# Therapeutic Suppression of FAK-AKT Signaling Overcomes Resistance to SHP2 Inhibition in Colorectal Carcinoma

**DOI:** 10.3389/fphar.2021.739501

**Published:** 2021-11-01

**Authors:** Ye Li, Yuncang Yuan, Fan Zhang, Aizhen Guo, Fuao Cao, Mengmeng Song, Yating Fu, Xiaowen Xu, Hao Shen, Shangyong Zheng, Yamin Pan, Wenjun Chang

**Affiliations:** ^1^ Department of Digestive Endoscopy, Shuguang Hospital, Shanghai University of Traditional Chinese Medicine, Shanghai, China; ^2^ Department of Environmental and Occupational Health, Second Military Medical University, Shanghai, China; ^3^ Laboratory of Animal Tumor Models, State Key Laboratory of Biotherapy and Cancer Center, West China Hospital, Sichuan University, Chengdu, China; ^4^ Department of General Practice, Yangpu Center Hospital, Medical School of Tongji University, Shanghai, China; ^5^ Department of Colorectal Surgery, Changhai Hospital, Second Military Medical University, Shanghai, China; ^6^ Department of Gastrointestinal Surgery/Clinical Nutrition, Beijing Shijitan Hospital, Capital Medical University, Beijing, China; ^7^ School of Medicine, Yunnan University, Kunming, China

**Keywords:** SHP2, AKT rebound, FAK, drug resistance, colorectal carcinoma

## Abstract

SHP2 mediates signaling from multiple receptor tyrosine kinases (RTKs) to extracellular signal-regulated kinase (ERK) and Ser and Thr kinase AKT, and its inhibitors offer an unprecedented opportunity for cancer treatment. Although the ERK signaling variation after SHP2 inhibition has been well investigated, the AKT signaling variation in colorectal carcinoma (CRC) is still unknown. Therefore, we performed immunohistochemistry and bioinformatics analyses to explore the significance of p-SHP2 in CRC. A panel of CRC cell lines with the SHP2 inhibitor, SHP099, was used to assess the effects on viability and signaling. The inhibitors of AKT and focal adhesion kinase (FAK) signaling were examined in combination with SHP099 as potential strategies to enhance the efficacy and overcome resistance. Frequent resistance to the SHP2 inhibitor was observed in CRC cells, even in those without RAS mutations. We observed rapid adaptive reactivation of the AKT pathway in response to SHP2 inhibition, possibly driven by the reactivation of RTKs or released *p*-FAK. High baseline p-FAK may also be associated with CRC cell resistance to SHP2 inhibition. Co-inhibition of FAK abrogated the feedback reactivation of AKT in response to SHP2 inhibition. Moreover, the combined inhibition of SHP2 with AKT or FAK resulted in sustained AKT pathway suppression and improved antitumor efficacy *in vitro* and *in vivo*. Our study found that reactivation of the AKT pathway is a key mechanism of adaptive resistance to SHP2 inhibition, highlighting the potential significance of AKT and FAK inhibition strategies to enhance the efficacy of SHP2 inhibitors in CRC treatment.

## Introduction

The non-receptor protein tyrosine phosphatase, SHP2, encoded by the gene of PTPN11, has a critical role in signal transduction downstream of growth factor receptor signaling and was the first reported oncogenic tyrosine phosphatase (Chan and Feng, 2007; Huang et al., 2014; Rehman et al., 2018). Activating mutations in this gene have been associated with developmental pathologies such as Noonan syndrome and are also frequently found in multiple cancers such as leukemia, lung and breast cancer, and neuroblastoma (Chan and Feng, 2007; Huang et al., 2014; Rehman et al., 2018; Martínez-Jiménez et al., 2020). SHP2 is ubiquitously expressed and regulates cell survival and proliferation primarily through the activation of the RAS–ERK and PI3K–AKT signaling pathways (Huang et al., 2014; Prahallad et al., 2015; Rehman et al., 2018). Additionally, it is a key mediator of the programmed cell death 1 (PD-1) and B- and T-lymphocyte attenuator immune checkpoint pathways (Prahallad et al., 2015; Zhao et al., 2019). Reduction in the SHP2 activity may suppress tumor cell growth and enhance the anti-tumor immune response (Prahallad et al., 2015; Zhao et al., 2019; Quintana et al., 2020). Thus, SHP2 is a potential target of cancer therapy, especially for many RTKs-driven tumors which depend on it for survival.

Epidermal growth factor receptor (EGFR) and multiple other RTKs are frequently over-expressed in CRC (García-Aranda and Redondo, 2019), which will usually result in the activation of SHP2 and its downstream signaling (Chen et al., 2016). Therefore, targeting SHP2 in CRC is a potential therapy (Prahallad et al., 2015; Chen et al., 2016; Rehman et al., 2018; García-Aranda and Redondo, 2019). However, KRAS or BRAF gain-of-function mutations are frequently observed in multiple cancer types, especially in CRC, pancreatic cancer, and non-small-cell lung cancers (NSCLC) (Network, 2012; Zehir et al., 2017), which may hijack the function of SHP2 as the key mediator of multiple RTKs to control the ERK and AKT signaling. Additionally, RAS-mutant tumors are insensitive to inhibition of upstream growth factor receptor signaling (Prahallad et al., 2015; Chen et al., 2016). Thus, SHP2 inhibition, which links RTKs to the RAS–RAF–MEK–ERK and RAS–PI3K–AKT–mTOR pathways, will be ineffective in KRAS-mutant or BRAF-mutant cancer cell lines. Previous data also indicate that SHP2 inhibition in KRAS-mutant NSCLC cell lines has little effect *in vitro* (Mainardi et al., 2018). However, inhibition of the RAS oncoproteins has been proven to be difficult, and attempts to target downstream effectors have been hampered by the activation of compensatory resistance mechanisms (Corcoran et al., 2012; Turke et al., 2012; Hirata et al., 2015; Kitai et al., 2016). Recently, SHP2 signaling response activation to the inhibitors of RAS downstream effectors has been reported in multiple cancer types, and combined targeting of RAS downstream effectors, especially for MEK inhibitors and SHP2, generated significant synthetic effects on tumor growth (Fedele et al., 2018; Wong et al., 2018; Ahmed et al., 2019; Lu et al., 2019). Thus, SHP2 is a promising target, especially as the combined therapy was used in RAS-mutant cancers.

RAS dominantly activates the ERK signaling and also controls the PI3K-AKT signaling by interacting with p110a (Zhang et al., 2002; Gupta et al., 2007; Castellano et al., 2013). However, the PI3K-AKT signaling may also be activated by SHP2 through moving p85, which is a suppressor for PI3K-AKT signaling independent of RAS mutation (Zhang et al., 2002). Recently, researchers have argued that the PI3K-AKT pathway requires RTK-induced activation, usually involving SHP2 as a critical mediator in KRAS-mutant cancers (Ebi et al., 2011; Navas et al., 2012; Hao et al., 2019). Thus, targeting the SHP2-PI3K-AKT pathway may still provide an attractive therapeutic strategy despite SHP2 downstream mutation. Moreover, SHP2 inhibition under growth factor-limiting conditions and in KRAS-mutant NSCLC xenografts provokes senescence responses (Mainardi et al., 2018). Therefore, the role and mechanism of SHP2 in CRC may be complex and require a profound study. The present study reported that most CRC cells are resistance to SHP2 inhibition, which is associated with a feedback reactivation of the AKT pathway. The underlying mechanism for AKT reactivation may be mediated by multiple RTKs and released *p*-FAK activation, followed by SHP2 inhibition. FAK co-inhibition prevented a more universal feedback reactivation after SHP2 inhibition, and the combined inhibition of SHP2 with AKT or FAK drove sustained AKT pathway suppression and improved antitumor efficacy both *in vitro* and *in vivo*. Therefore, the present study not only demonstrated the feedback reactivation of AKT pathway as a key mechanism for the resistance of CRC to SHP2 inhibition, but also provided the combination of SHP2 and each of the AKT and FAK pathway inhibition as potential strategies to enhance the efficacy of SHP2 inhibition.

## Materials and Methods

### Bioinformatics

The *p*-SHP2 expression data in 7694 cancer specimens and 277 cancer cell lines, which were examined by reverse phase protein array (RPPA), were downloaded and retrieved manually from the website of the cancer proteome atlas (TCPA) portal (https://tcpaportal.org/). The half maximal inhibitory concentrations (IC_50_) of 496 cancer cell lines including 41 CRC cell lines in response to SHP099 were also retrieved from a study (Hao et al., 2019) that studied the mutation status of KRAS and BRAF. All 496 cell lines past the information check of CCLE (https://portals.broadinstitute.org/ccle/about) and Cellosaurus (https://web.expasy.org/cellosaurus/). The cell lines with an IC_50_ value of SHP099 more than 30 μg/ ml were defined as the resistance phenotype (Hao et al., 2019). Then, the differences in *p*-SHP2 expression between CRC (*n* = 487) and other cancer types were ranked with the median and compared. The status of KRAS or BRAF mutation of all cancer cell lines were retrieved from CCLE and the *p*-SHP2 expression across the cancer cell lines was discriminated into high or low expression with a normalized RPPA expression of 0.1 as the cut-off value. Then, the distribution of the resistant cell lines concerning the subgroups of mutation status (KRAS or BRAF mutation), and p-SHP2 expression (high or low) was also investigated.

### Patients

The present study was conducted in 365 patients with localized CRC who received curative surgery in Changhai Hospital, Second Military Medical University (Shanghai, China) between January 2008 and October 2011. Less than 5% of patients with rectal cancer received preoperative radiotherapy in the cohort. The baseline information of patients, including age, gender, TNM stage (determined according to the American Joint Committee on Cancer Staging Manual, seventh edition), differentiation grades, carcinoembryonic antigen (CEA), and CA199 is presented in Supplementary Table S1. A written informed consent was obtained from each patient. The formalin-fixed paraffin-embedded specimens, including 365 cancerous and 75 noncancerous tissues, from the patients were collected and were used to construct tissue microarrays (TMAs) by a commercial company (Outdo Biotech, Shanghai, China). The TMA construction details were described in a previous study (Chang et al., 2014).

### Cells and Reagents

Cell lines were obtained from the American Type Culture Collection (ATCC), which routinely performs cell line authentication by short tandem repeat analysis, and maintained in Dulbecco’s modified eagle medium (CaCO2, CW2 and SW480) or RPMI-1640 (SW620, RKO, and Colo-205) supplemented with 10% heat-inactivated fetal calf serum (GIBCO), 100 U/ mL penicillin, and 100 mg/ ml streptomycin at 37°C in a humidified atmosphere containing 5% carbon dioxide (CO_2_). The SHP2 inhibitor (SHP099, #HY-100388) and the AKT-1/2/3 inhibitor (MK-2206, #HY10358) were purchased from MedChemExpress. The FAK inhibitors of PF-573228 (#S2013) and VS-4718 (#S7653) were purchased from Selleckchem.

### Cell Proliferation Assays

CRC cells were seeded in triplicate in 96-well plates at 4,000 cells per well and exposed to the inhibitors of SHP099 and MK-2206 both alone and in combination with indicated concentrations, and the dimethyl sulfoxide (DMSO) as control. The number of viable cells at 24, 48, and 72 h was assessed using Cell Counting Kit-8 (Dojindo, Kumamoto, Japan) according to the manufacturer’s instructions. The absorbance at 450 nm was measured to reflect the viable cell population. To determine the IC_50_ values, data were fitted using the dose response algorithm in Graphpad Prism as Y = Bottom + (Top − Bottom)/[1 + 10^(X-LogEC50)], in which the top and bottom are plateaus in the units of the Y axis, and EC50 is the inhibitor concentration that gives a response half-way between the bottom and top.

### Drug Combination Studies

The combination effect of SHP099 and MK-2206 on a panel of CRC cell growth was analyzed using CompuSyn 1.0 (Chou and Martin, 2005). The individual dose–effect of each drug was obtained by treating 5 CRC cell lines with SHP099 or MK-2206. The median effect dose (Dm) and linear correlation coefficient of the ME-plot (*r*) were analyzed. Optimal concentration ratios were obtained based on the Dm values, and six serial dilutions of the optimal ratio were used to measure the cytotoxic effect. Combination index (CI) of the combined use of different drugs was calculated using CompuSyn, which defined synergism (CI < 1), additive effect (CI = 1), and antagonism (CI > 1).

### Colony Formation Assays

The cells were initially cultured in 6-well plates for colony formation assay (Corning, NY, United States) at a density of 2.0 × 10^3^/well, and the regular medium supplemented with the inhibitors was refreshed every 2–3 days. After culturing for 2–3 weeks, the resulting colonies were fixed with ice-cold methanol and stained with a crystal violet solution for counting. The assay was performed in triplicate. The plates were scanned using a photo-scanner, and cell growth was quantified using ImageJ software.

### Animal Studies

Experiments were performed on 4-week-old nu/nu athymic BALB/c male mice obtained from the Shanghai JiHui experimental animal breeding company, Shanghai, China, and all the mice were maintained in pressurized ventilated cages under an Institutional Animal Care and Use Committee-approved protocol and institutional guidelines for the proper and humane use of animals in research. Subcutaneous tumors were generated by transplanting 0.5–1.0 × 10^7^ tumor cells (SW620 and Colo205) in phosphate buffered saline (PBS) into the right flank (200 μL/mouse) and randomized approximately 14 days post-implantation (size >100 mm^3^). The mice were treated with SHP099, MK-2206, VS-4718, or their combination with the indicated doses. SHP099 was formulated in 30% hydroxypropyl-β-cyclodextrin, whereas MK-2206 was dissolved in 30% Capitisol and administered by oral gavage. For the VS-4718 treatment, drug was prepared in 0.5% carboxymethyl cellulose and 0.1% Tween 80 and the mice were treated at 35 mg/ kg BID by gavage. All inhibitors were administered orally every day. The reagents which used to dissolve the inhibitors were taken as the vehicle control in the study. Tumor dimensions were measured with vernier calipers at an interval of 3 days, and tumor volumes were calculated as follows: π/6 × larger diameter × (smaller diameter)^2^. Animals were sacrificed by CO_2_ euthanasia when tumors reached the maximum-allowed size or when signs of ulceration were evident. After image analysis, the isolated tumor specimens were further processed for western blotting and immunohistochemistry (IHC) examination as corresponding manual.

### Western Blotting

The cells were washed with PBS once, disrupted on ice for 30 min by using radioimmunoprecipitation assay (RIPA) lysis and extraction buffer (Thermo, America). Pierce protease and phosphatase inhibitor mini tablets (Thermo, America) were added at one tablet per 10 ml solution and centrifuged for 15 min (14,000 ×g) at 4°C. Protein concentration was determined with bicinchoninic acid (BCA) reagent (Dingguo, Beijing). Equal amounts of protein (10–50 μg) in cell lysates were separated by 10% sodium dodecyl sulfate–polyacrylamide gel electrophoresis (SDS-PAGE), transferred to polyvinylidene difluoride membranes (Millipore), immunoblotted with specific primary and secondary antibodies, and detected through chemiluminescence by using the enhanced chemiluminescence (ECL) detection reagents from Millipore. Antibodies for western blotting against *p*-ERK1/2 (T202/Y204, 1:2000, #4370), ERK1/2 (1:1000, #4695), AKT (1:1000, #4691), *p*-AKT (S473, 1:1000, #4060), and *p*-FAK (Tyr397, 1:1000, #3283) were purchased from Cell Signaling Technologies (CST). GAPDH (1:5000, ab181602) and *p*-SHP2 (Y542, 1:1000, ab62322) were purchased from Abcam. The primary antibodies above are all from rabbit, so the secondary antibody of anti-rabbit IgG, HRP-linked Antibody (1:5000, #7074, CST) was used in the study.

### RTK Arrays

Human phospho-RTK arrays (R & D Systems) were utilized according to the manufacturer’s instructions. Cells were briefly washed with cold PBS, lysed in NP40 lysis buffer, and 100 mg of lysates was incubated overnight with blocked membranes. Membranes were subsequently washed and exposed to a chemiluminescent reagent and an X-ray film. Quantification of pixels was performed through densitometry by using Adobe CS2 and Fuji Film Multi Gauge software.

### IHC Assay

IHC antibodies for *p*-SHP2 (1:200, sc-280, Santa Cruz) were used in this study. All TMAs were stained simultaneously. The p-SHP2 expression was semi-quantitated using the H-score method as our previously reported (Yuan et al., 2019). The score of the *p*-SHP2 expression was defined as the staining intensity (0, negative; 1, weakly positive; 2, moderately positive; 3, strongly positive) multiplied by the percent tumor-positive area (0–100%). IHC scores were independently assessed by two observers (Y.Y and Z.F) who were blinded to the information of the specimen donors. IHC scores from the two observers was average for further analysis, and controversial cases (defined as a difference in IHC scores more than 10% of the average score) were jointly re-evaluated until a consensus was reached.

### Statistics

Expression levels of *p*-SHP2 in the CRC and adjacent normal tissues were compared using independent sample *t*-test for non-paired samples. The proportion of cancer cells with the resistance phenotypes between the subgroups concerning the mutation status of KRAS or BRAF or the levels of *p*-SHP2 was compared using chi-square test. All statistical tests were two-sided and were performed using SPSS version 22.0 for Windows (SPSS, Chicago, IL, United States). A *p* value of <0.05 was considered statistically significant.

## Results

### universal Resistance to SHP2 Inhibitor SHP099 in CRC

**FIGURE 1:**
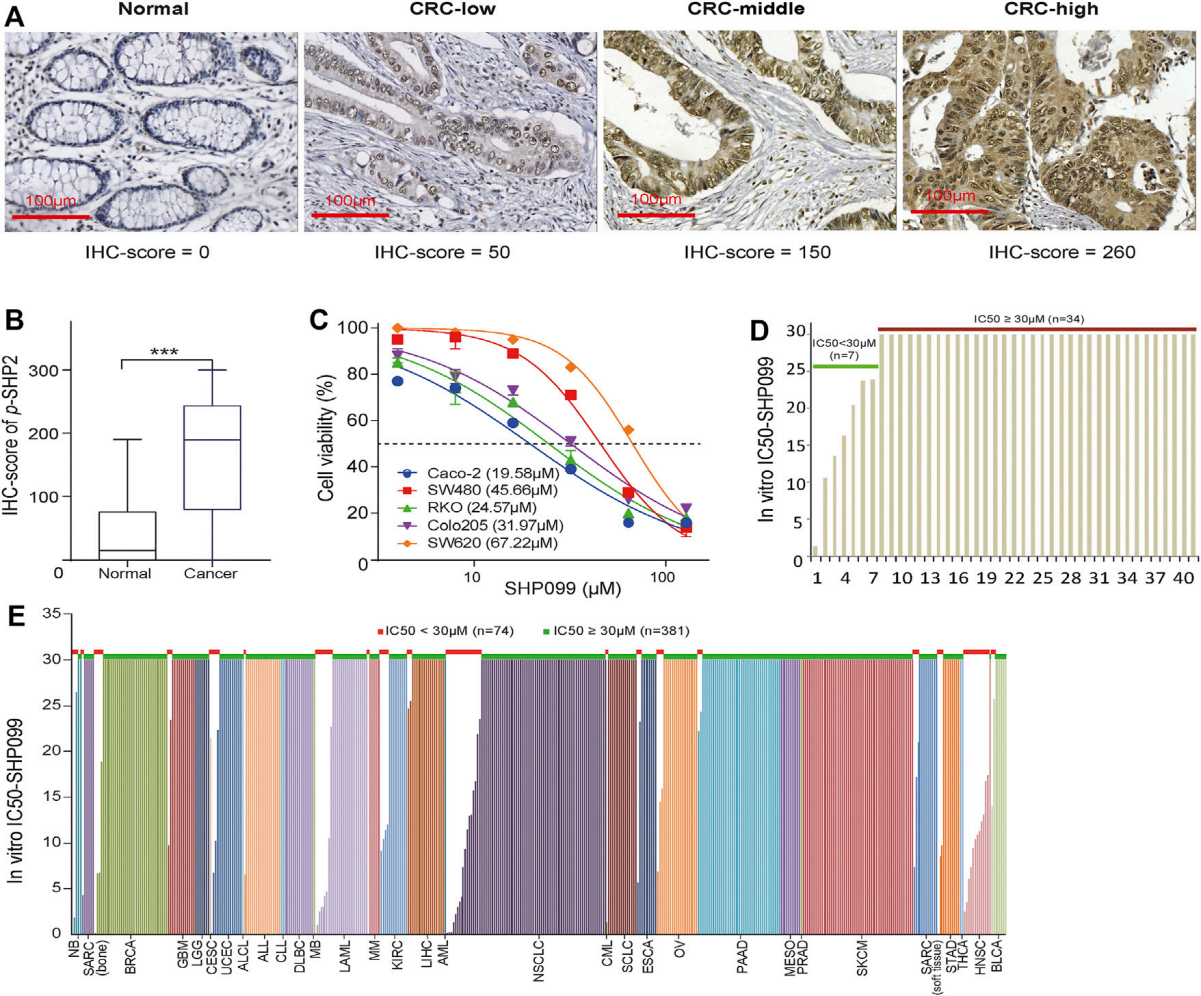
The expression of p-SHP2 in CRC and the activity of its inhibitor (SHP099) in cancer cells. **(A)** Representative images of p-SHP2 expression in colorectal tissues based on IHC. **(B)** Increased p-SHP2 expression in CRC. (^***^< 0.001) **(C)** Activity of SHP099 in 5 CRC cell lines with indicated IC50s. **(D)** Analyses of IC_50_ values of SHP099 in 41 CRC cell lines. **(E)** Analyses of IC_50_ values of SHP099 in 455 cancer cell lines excluding CRC cell lines. CRC: colorectal cancer; IHC: immunohistochemistry; IC_50_: the half maximal inhibitory concentration.

The IHC examination of *p*-SHP2 in 365 CRC and 75 noncancerous specimens indicated that the protein is located mainly in the cytoplasm of epithelial cells (Figure 1A) and is significantly elevated in CRC (Figure 1B). According to the *p*-SHP2 expression from the RPPA examination across TCGA cancers, as illustrated in Supplementary Figure S1, the median of *p*-SHP2 levels in CRC was located in the front of the list (ranked 12) among 31 cancer types. The data indicated that *p*-SHP2 may be a valuable target for CRC treatment. Next, we evaluated the effect of SHP2 inhibition with SHP099 on the growth of 5 CRC cell lines. IC_50_ values of more than 30 μM were observed in 3 of 5 tested CRC cells (Figure 1C), indicating a resistance to the blockage of SHP2 signaling. Moreover, bioinformatics analysis exhibited that only 17.1% (7/41) CRC cells (Figure 1D) and 16.3% (74/455) cell lines from other cancer types possessed IC_50_ values less than 30 μM (Figure 1E), confirming universal resistance to SHP099 among cancer cell lines. Although gain-of-function mutations of KRAS and BRAF have been identified as a contributor to SHP099 resistance (Mainardi et al., 2018), more than 77.4% (233/301) of all cancer cell lines with the wild types of both KRAS and BRAF were resistant to SHP099 treatment (Supplementary Figure S2A). We further considered the association between *p*-SHP2 status and the resistance to SHP099 in cells with both BRAF and KRAS wild types and observed only a marginal significance (*p* = 0.052) (Supplementary Figure S2B). Therefore, the potential mechanisms constraining the efficacy of SHP099 are still unclear and must be explored.

### Rapid Feedback Reactivation of AKT Pathway Following SHP2 Inhibition

**FIGURE 2:**
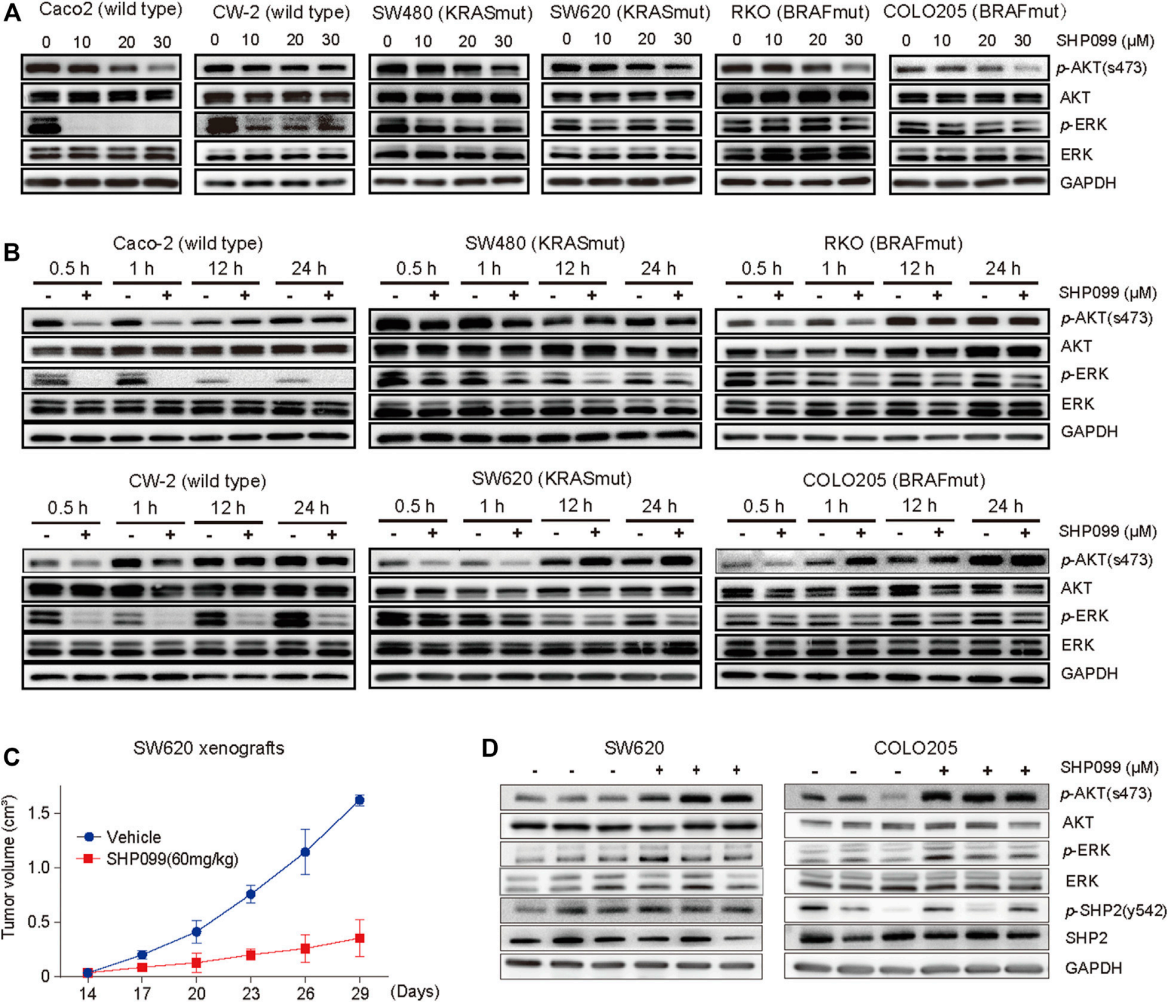
Feedback activation of AKT signaling followed by SHP2 inhibition with SHP099 in CRC cells. **(A)** SHP099 reduces the expression of p-AKT in indicated CRC cells. **(B)** A transient inhibition of p-AKT followed by feedback activation of the signaling across all indicated CRC cells. The same cell lines treated with different concentrations of SHP099 such as Caco-2 (20 μM), CW-2 (20 μM), RKO (30 μM), Colo205 (30 μM), SW480 (30 μM), and SW620 (40 μM) and collected at indicated times and analyzed by immunoblotting. **(C)** SHP099 reduces the growth of xenograft tumors derived from SW620 cells. **(D)** Increased activity of p-AKT signaling after SHP099 treatment from isolated xenograft tumors derived from CRC cells.

ERK and AKT signaling usually serve as the most crucial effectors for SHP2 inhibition. To investigate their changes in response to SHP2 inhibition, we evaluated the effects of SHP099 on a panel of CRC cell lines. As expected, we observed that the level of *p*-ERK in CaCO_2_ and CW-2 cells, which express wild types of both KRAS and BRAF, was sharply reduced at 45 min in response to SHP099, whereas it was only moderately or slightly reduced at a higher dosage of SHP099 in cells with KRAS or BRAF mutations (Figure 2A). The level of *p*-AKT was significantly suppressed by SHP099 with dosage-dependent trends across almost all tested cells (Figure 2A), independent of the mutation status of KRAS or BRAF. However, following the suppression, a significant rebound was observed in the level of *p*-AKT in response to SHP099 at 12 h (Figure 2B), although the rebound of *p*-AKT in RKO cells occurred at approximately 24 h. Additionally, the *p*-ERK levels in response to SHP099 were still sharply reduced in the CaCO2 and CW-2 cells and slightly or moderately reduced in the other cells (SW620, RKO, Colo205, and SW480) even after an extension of the observed time to 24 h (Figure 2B). Moreover, the *p-*AKT reactivation in CRC xenograft models following SHP2 inhibition also have be investigated. Although the isolated xenograft tumors (from SW620 or Colo205) from SHP099-treated animals exhibited smaller tumor sizes than the control group (Figure 2C), they exhibited consistently higher *p*-AKT levels than the controls (Figure 2D). Moreover, the *p*-ERK level in the SHP099 treatment groups was slightly elevated than that in the control groups (Figure 2D). Therefore, the AKT pathway exhibits a clear early repressed and then rebounded response to SHP099, indicating a dynamic and complex interaction between SHP2 and AKT signaling.

### Synergistic Suppression of CRC by SHP2 Inhibition and AKT Blockage

**FIGURE 3:**
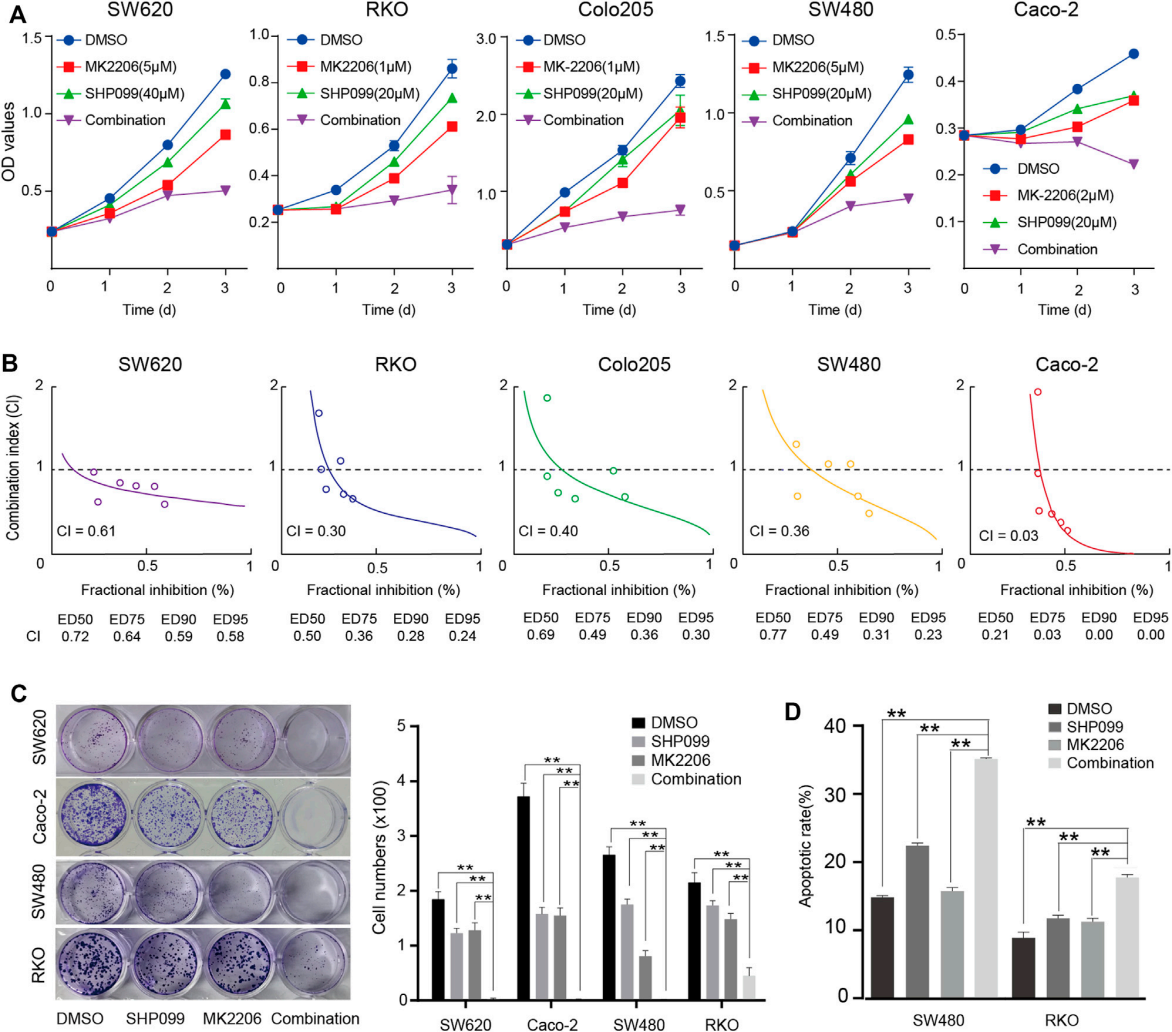
Synergistic suppression effect of SHP099 and MK-2206 on CRC cell growth. **(A)** The effect of SHP099 and MK-2206 on the proliferation of the 5 indicated cell lines. **(B)** Combination index of SHP099 and MK-2206 of the 5 involved CRC cell lines. **(C)** The effect of SHP099 and MK-2206 on the colony formation of the four indicated cell lines. **(D)** The effect of SHP099 and MK-2206 on the apoptosis of the 2 indicated cell lines. (^***^ *p* < 0.001, ^**^ *p* < 0.01, ^*^ *p* < 0.05).

The inhibition of AKT signaling may sensitize the role of SHP099 because the AKT signaling activation is always associated with drug resistance (Datta et al., 2017; Song et al., 2017; Vitiello et al., 2019). By using siRNAs targeting AKT1-3, we observed that SHP099 significantly reduces the proliferation and colony formation of CRC cells (CaCO2 and SW480) as knocking down AKT (Supplementary Figures S3A–3B). Furthermore, we assessed the combined effect of SHP099 and MK-2206 (a specific AKT inhibitor) on CRC. The proliferation curves consistently exhibited that the combined treatment is the most effective inhibition of CRC growth among all groups across tested cell lines, particularly the inhibition effect on the third day that strongly indicated a synergistic interaction between SHP099 and MK-2206 (Figure 3A). The CI was employed to examine the presence of a synergistic effect by the combination of SHP099 and MK-2206. The CI values from the 5 cell lines were all less than 0.70, which suggested a stable synergism in all tested cell lines (Figure 3B). The strongest synergism (CI ≤ 0.3) was observed in the RKO and CaCO2 cell models (Figure 3B). Moreover, the synergistic effect of SHP099 and MK-2206 was also supported by the results of colony formation (Figure 3C) and cell apoptosis assays (Figure 3D). Thus, the synergistic inhibitory effect of SHP2 and AKT on CRC growth exists universally.

### Combined SHP2 and AKT Inhibition Drives Tumor Regressions *in vivo*

**FIGURE 4:**
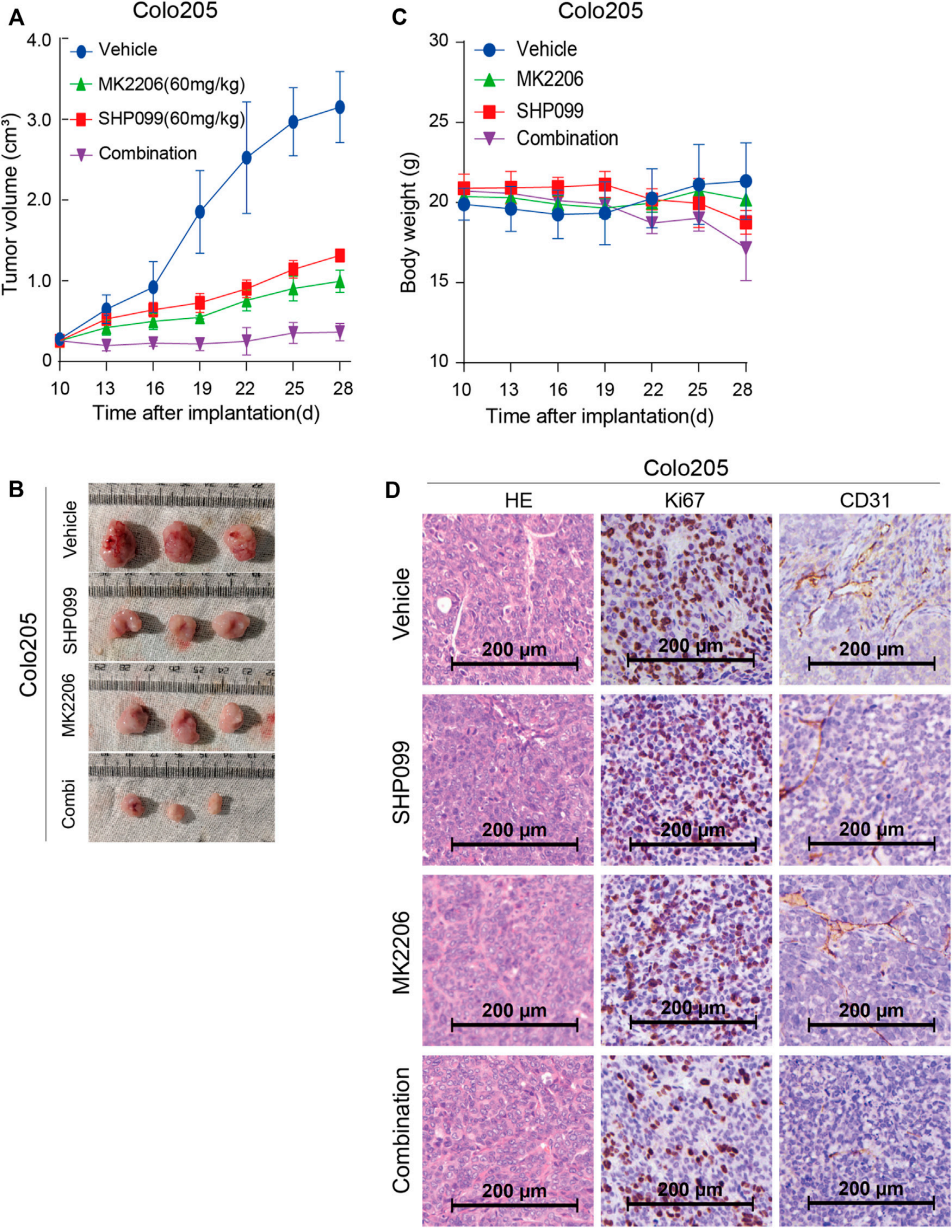
Effect of SHP099 and MK-2206 on the growth of xenograft CRC tumors. **(A)** Dynamic effect of SHP099 and MK-2206 on the CRC tumor volumes. **(B)** Represented images from isolated CRC tumors receiving SHP099 and MK-2206 treatment. **(C)** The body weight of animals during the treatment of SHP099 and MK-2206. **(D)** The effect of SHP099 and MK-2206 on the expression pattern of Ki67, and CD31 in isolated xenograft tumors examined by IHC.

Having established the effect of combined SHP099 and MK-2206 on CRC cells, we set out to validate the findings with *in vivo* models. Firstly, we injected the Colo205 and SW620 cells into nude mice until the tumors reached the required volumes at approximately the sixth day. Then, daily oral administration of a single agent SHP099 or MK-2206 and their combination was employed according to the designed regimen. The tumor volume difference among the four groups was not significant until the 19^th^ day (Figure 4A). Although both SHP099 and MK2206 exhibited significant inhibition of tumor growth in animal models (Figures 4A,B), the combination exhibited the maximum inhibition of tumor volumes among all groups, which almost retained the original sizes throughout the experiment period. Moreover, the difference in body weight between groups was statistically nonsignificant (Figure 4C). Consistent with at least some non-autonomous effects, SHP099 decreased tumor vascularity, as monitored by CD31 immunostaining, and the proliferation marker Ki67 also exhibited the weakest intensity in the group of combined inhibitors (Figure 4D). Therefore, the xenograft models consistently exhibited that the combination of SHP099 and MK-2206 may overcome the adaptive feedback resistance and may represent a promising therapeutic strategy.

### Induction of Phosphorylation of Multiple RTKs by SHP2 Inhibition

**FIGURE 5:**
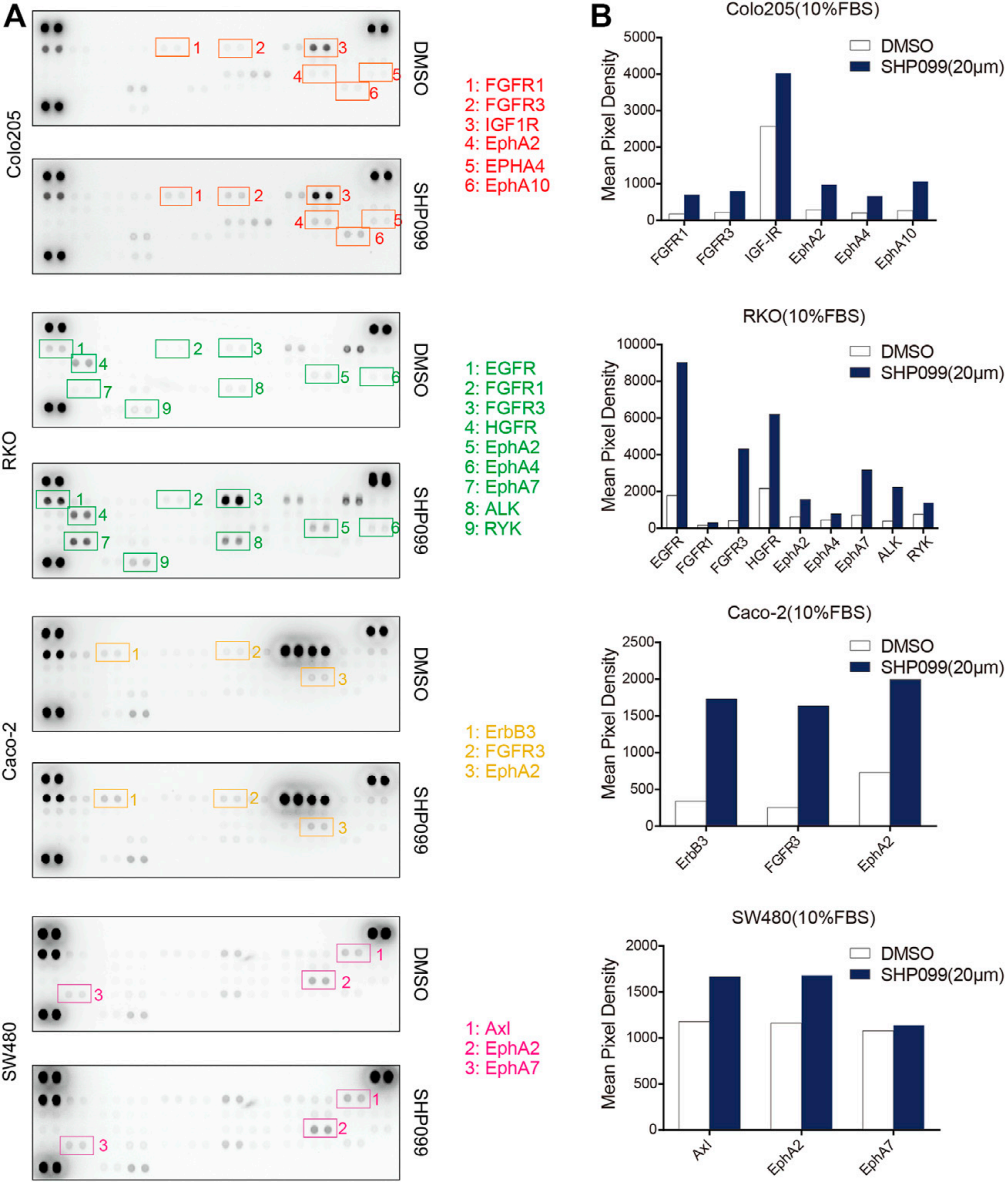
SHP2 inhibition with SHP099 induces several phosphorylated RTKs. **(A)** Induced expression profiles of RTKs by SHP099 for 24 h in CRC cells with the examination by phospho-RTK arrays. Spots are in duplicate, and each pair corresponds to a specific p-RTK. **(B)** Comparison of typical induced p-RTK expression by SHP099 across several CRC cell lines.

Multiple RTKs activated PI3K–AKT signaling in normal and tumor cells (García-Aranda and Redondo, 2019; Chandarlapaty et al., 2011). To investigate the role of RTKs in rebound of AKT signaling in response to SHP099, we employed an anti phosphotyrosine receptor antibody array to assess the levels of RTK activation at baseline and after 24 h of SHP099 treatment (Figure 5A). We observed that four phosphorylated RTKs (EGFR, IGF1R, Insulin R, and AXL) are the most prominent in at least 1 of the four tested cell lines. Additionally, following 24 h of SHP099 treatment, we observed that the phosphorylation of many RTKs (EGFR, ERBB3, FGFR1, FGFR3, EPHA2, EPHA4, EPHA7, EPHA10, HGFR, and ALK) is induced more than twofold in 24 h in at least 1 cell line (Figure 5B). However, the changed profile of the RTKs was extremely heterogenous across all indicated cell models, implying that multiple RTKs may play a role in the adaptive feedback of SHP099 treatment. The induction of tyrosine phosphorylation of EPHA2 following SHP099 treatment was consistently observed across four tested cell models, indicating that EPHA2 is a common target. However, the potential T594 site inhibited AKT activation (Miao et al., 2009). Therefore, the variable induction of multiple RTKs across different cell models following SHP099 treatment and different levels of phospho-RTKs at baseline suggest that distinct and multiple RTKs may drive adaptive feedback across different CRC cell lines and that the strategies targeting a single RTK may not be universally effective.

### FAK Mediation of the Feedback Reactivation of the AKT Pathway Following SHP2 Inhibition in a Subset of CRC Cells

**FIGURE 6:**
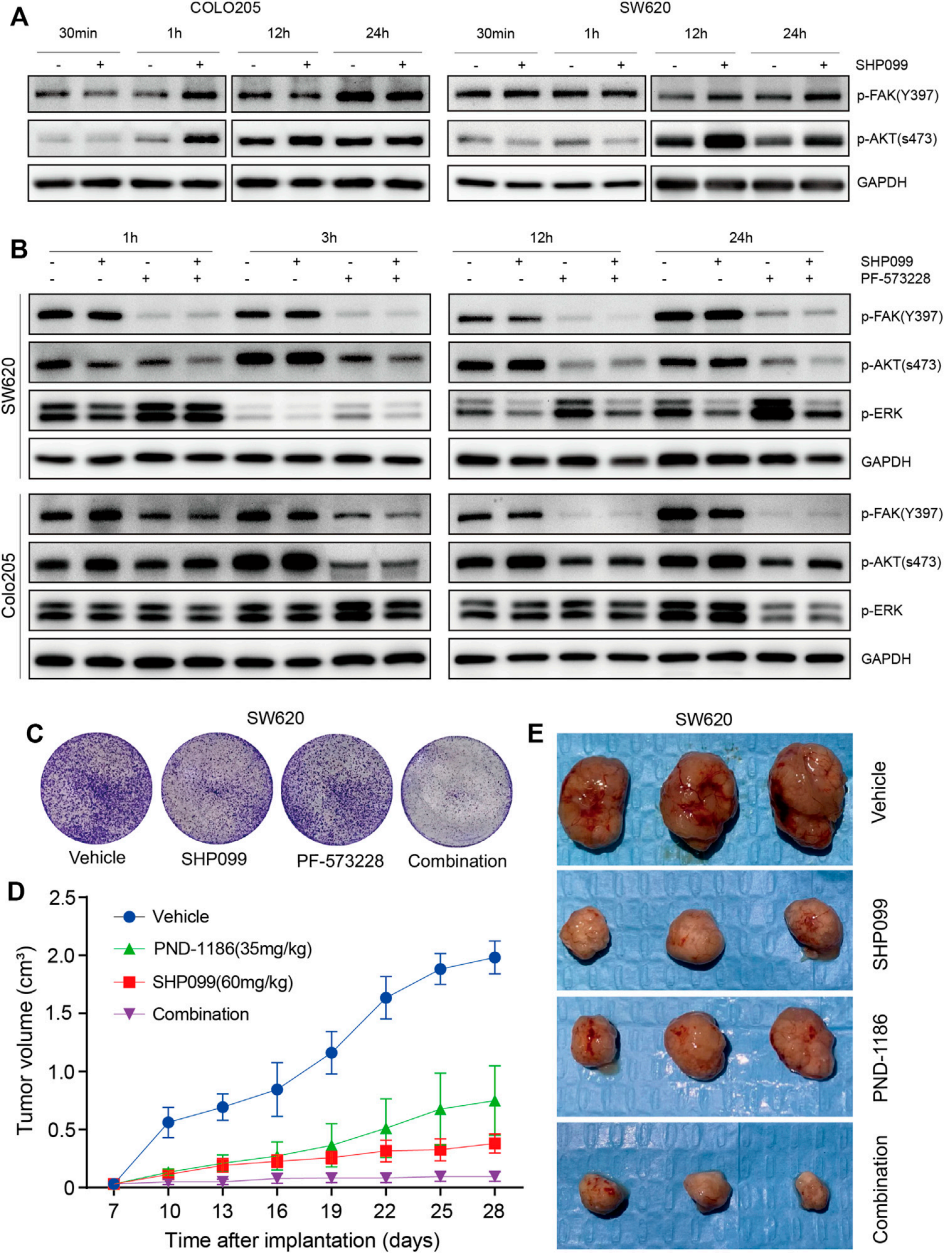
p-FAK inhibition improves antitumor efficacy when combined with SHP099. **(A)** Dynamic p-FAK is associated with the feedback activation of AKT signaling. **(B)** Dynamic effect of SHP099 and PF-573228 (10 μM) on the AKT and ERK signaling in CRC cells. **(C)** PF-573228 sensitizes the colony inhibition of SHP099 in SW620 cells. **(D)** PND-1186 enhances the growth inhibition of SW620 xenografts by SHP099. **(E)** Images of isolated tumors from SW620 xenografts treated by SHP099 and/or PND-1186.

Studies have reported the suppression of FAK signaling (Marin et al., 2008; Hartman et al., 2013; Lee et al., 2015) and activation of AKT–mTOR signaling (Ashton et al., 2010; Yoon et al., 2017) by SHP2. The present study evaluated the association between SHP2, FAK, and AKT signaling. The *p*-FAK induced by SHP099 treatment was significantly increased at 1 h for Colo205 and at 12 h for SW620 (Figure 6A), which is tightly correlated with the rebound of *p*-AKT, suggesting that FAK may be a key mediator for feedback reactivation of the AKT pathway following SHP2 inhibition. However, this phenomenon was not observed in other cell lines (Supplementary Figure S4A). The baselines of *p*-FAK were significantly higher in these cell lines than those in SW620 and Colo205 (Supplementary Figure S4B). To explore the role of induced FAK in the rebound of AKT pathway following SHP2 inhibition, we combined FAK inhibitor PF573228 and SHP099 to treat CRC cells. The combination of PF573228 and SHP099 not only eliminated the feedback reactivation of the AKT pathway but also generated stronger inhibition of *p*-AKT than SHP2 alone (Figure 6B). The result clearly demonstrated that released FAK activation following SHP2 inhibition may be a key mediator for the feedback reactivation of AKT pathway in SW620 and Colo205.

### Sensitization of the Suppression of SHP2 Inhibition on CRC by FAK Blockage

**FIGURE 7:**
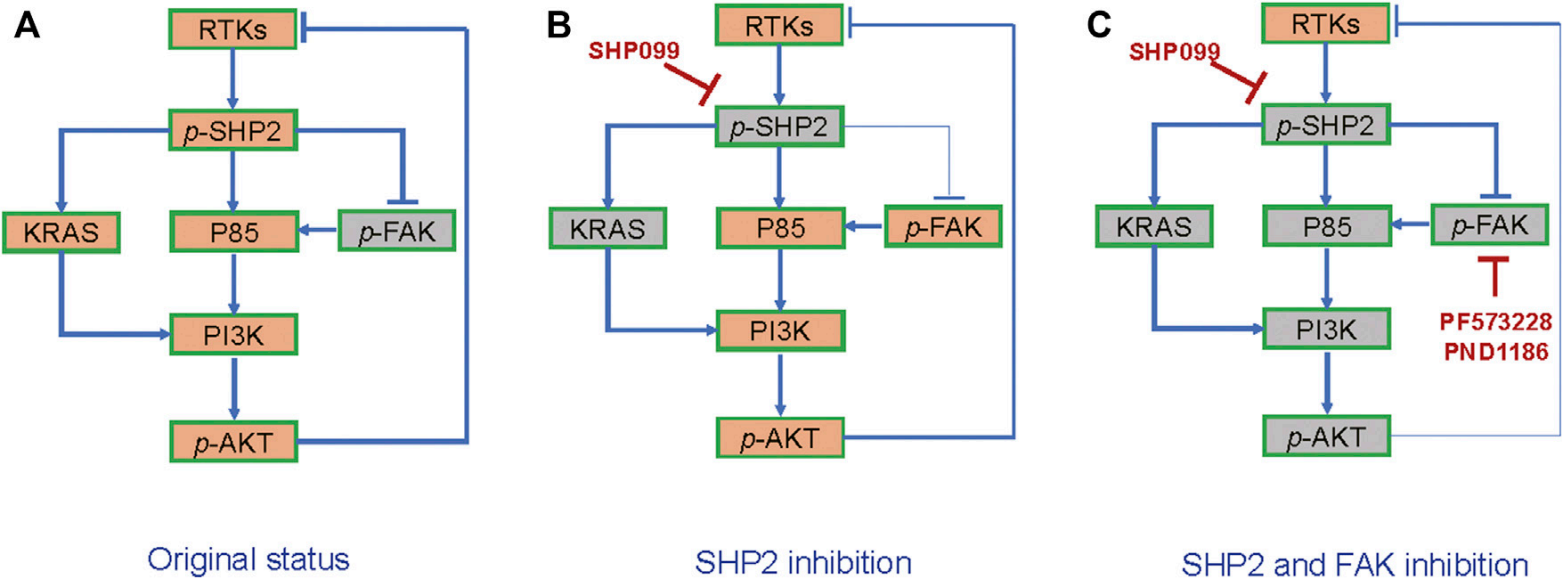
The schematic diagram of combined suppression of SHP2/FAK-AKT signaling. **(A)** RTKs-SHP2 signal activates AKT but suppresses FAK. **(B)** FAK-AKT signal is released in response to SHP2 inhibition. **(C)** Combination of SHP2 and FAK blockages generates stronger AKT inhibition than the inhibitor alone.

To explore the function of induced or baseline *p*-FAK in the resistance to SHP2 inhibition, we investigated the effects of SHP2 and FAK inhibitors, either alone or in combination, on CRC growth. The colony assays exhibited that the combined inhibition of SHP2 and FAK results in the strongest inhibition among all indicated groups, across not only SW620 (Figure 6C) but also the other four cell lines (Supplementary Figure S4C). Thus, the suppression of induced or baseline *p*-FAK can overcome the resistance to SHP099. With SW620-derived xenograft models, we further observed significant tumor suppression with a combination of SHP099 and FAK inhibitor PND-1186/VS4718 (Figures 6D,E). Thus, the findings indicate that the combination of SHP2 and a FAK inhibitor may be a board-spectrum and promising treatment regimen for CRC, and the potential working mechanism of the combination therapy on AKT pathway is represented in Figure 7.

## Discussion

The activation of AKT and ERK pathways by the multiple RTKs is critical in the pathogenesis of cancer. As a key mediator, SHP2 inhibition represents a potential opportunity to block these pathways (Huang et al., 2014; Prahallad et al., 2015; Chen et al., 2016; Rehman et al., 2018). In the present study, we observed that *p*-SHP2 expression is significantly elevated in the CRC epithelial cells as compared with normal tissues, which supported the use of SHP2 as a target for CRC treatment. However, the use of an SHP2 inhibitor alone may be ineffective in most CRC cells, especially for cells with gain-of-function mutations of KRAS or BRAF (Chen et al., 2016; Mainardi et al., 2018). We observed that most of the cancer cells with the wild types of both KRAS and BRAF still exhibit resistance to SHP2 inhibition, which cannot be fully explained by the *p*-SHP2 levels, indicating an unknown mechanism for the resistance.

The ERK pathway has been investigated widely as a key effector of SHP2 inhibition in studies (Chan and Feng, 2007; Huang et al., 2014; Prahallad et al., 2015; Chen et al., 2016; Fedele et al., 2018; Mainardi et al., 2018; Rehman et al., 2018; Wong et al., 2018; Ahmed et al., 2019; García-Aranda and Redondo, 2019; Lu et al., 2019; Zhao et al., 2019; Martínez-Jiménez et al., 2020; Quintana et al., 2020) and may not be affected by SHP2 inhibitors in cancer cells with KRAS or BRAF mutations (Chen et al., 2016; Mainardi et al., 2018). Similarly, the inhibition of SHP2 with SHP099 generated the obvious reduction of *p*-ERK only in those cells with both wild types of KRAS and BRAF. Researchers have observed higher levels of PI3K/AKT pathway activation in CRC than those in other cancer types (even in BRAF mutated specimens) (Ebi et al., 2011), indicating a critical role of PI3K/AKT pathway in cancer. However, the variation in the AKT pathway, a key effector of SHP2, has still not been well investigated in response to SHP2 inhibition. For CRC cells, we clearly observed a rapid reduction in the AKT pathway activation after SHP099 treatment, which is independent of KRAS or BRAF mutation. This result is consistent with a recent notion that SHP2 has a major effect on AKT pathway in CRC, even in those cells with KRAS and BRAF mutations (Ebi et al., 2011). An alternative explanation for this data is the alternative regulation of SHP2-p85-p110α and SHP2-KRAS-p110α, and the former of which is not depended on KRAS mutation. Unexpectedly, in 12–24 h after the reduction of the pathway, a reactivation of the AKT pathway was observed consistently across all tested CRC cells. Furthermore, we also confirmed that the reactivation of the AKT pathway occurs in xenograft models of CRC. Although the reactivation of the AKT pathway by its inhibitors has been reported (Chandarlapaty et al., 2011), the reactivation of AKT pathway induced by its upstream inhibition such as SHP099 is scarcely reported until now (Lauriol et al., 2016).

Concerning that the activation of the AKT pathway is usually associated with drug resistance (Datta et al., 2017; Song et al., 2017; Vitiello et al., 2019), we propose that the blockage of the reactivation of the AKT pathway in response to SHP2 inhibition may overcome the resistance of CRC cells to SHP2 inhibitors (Song et al., 2017; Sun et al., 2019; Leung et al., 2020). In line with this hypothesis, we observed that the combined inhibition of SHP2 and AKT pathways with SHP099 and MK-2206 generated a synthetic suppression of the growth of all tested CRC cells. The efficacy of the combined treatment was further confirmed with colonic assays and in animal xenograft models. Additionally, the combination of SHP2 and AKT inhibitors is almost comparable with the combined inhibition of SHP2 and ERK pathways, which has been reported in recent studies (Fedele et al., 2018; Wong et al., 2018; Ahmed et al., 2019; Lu et al., 2019). Moreover, SHP099 exhibited synergistic potential when combined with PI3K inhibitors (such as Pictilishib) (Chen et al., 2016; Sun et al., 2019), which supported our hypothesis. Therefore, we report that targeting the AKT pathway also has the potential to sensitize the efficacy of SHP2 inhibitor.

RTK-driven feedback reactivation of AKT or ERK signaling has been identified as a key driver of drug resistance in cancers treated with AKT and mTOR inhibitors or BRAF and MEK inhibitors (Corcoran et al., 2012; Turke et al., 2012; Sun and Bernards, 2014; Datta et al., 2017; Fedele et al., 2018; Wong et al., 2018; Ahmed et al., 2019; Lu et al., 2019; Leung et al., 2020). Thus, the reactivation of AKT following SHP2 inhibition may also be associated with RTKs. We observed that the levels of multiple RTKs is elevated in response to SHP099. However, the profile of RTKs was extremely heterogeneous among CRC cells. Similarly, the heterogeneity of the profile of RTKs induced by another inhibitor was also reported (Hao et al., 2019). Thus, the results indicate that the blockage of the reactivation of AKT pathway is difficult by targeting a dominant RTK.

When SHP2 is inhibited by SHP099, other mediators such as FAK and RSK can also take over the signaling from RTKs to AKT and ERK. Due to the FAK dephosphorylation by SHP2 (de Oliveira et al., 2009; Hartman et al., 2013), the SHP2 inhibition may result in the released activation of FAK signaling. We observed obvious elevation in FAK signaling following SHP2 inhibition in a subset of CRC cells, which is tightly correlated with the reactivation of AKT signaling. Interestingly, other cells without the proposed association exhibited consistently high baseline expression of *p*-FAK. The nuclear FAK expression is also associated with a poor prognosis in CRC (Albasri et al., 2014; Davis et al., 2017). The results promoted us to investigate the effect of the combined inhibition of SHP2 and FAK on all CRC cells. Surprisingly, the combined treatment resulted in obvious growth inhibition across all the tested cells. Moreover, the combination of SHP2 and FAK inhibitors resulted in a stronger reduction of AKT signaling in CRC cells, indicating that the role of FAK inhibitor as a sensitizer on SHP2 inhibitor depends at least partly on the blockage of AKT pathway reactivation induced by SHP2 inhibition. Therefore, FAK is a promising combination partner for SHP2 inhibitors, capable of preventing adaptive feedback reactivation from multiple RKTs to maintain AKT pathway suppression and enhance efficacy *in vitro* and *in vivo*. Additionally, the inhibition of SHP2 and FAK signaling may also contribute to enhanced antitumor immune response (Prahallad et al., 2015; Serrels et al., 2015; Jiang et al., 2016; Serrels et al., 2017; Zhao et al., 2019; Quintana et al., 2020), which must be explored further.

Our study has also some limitation. First, it is insufficient to clarify the effect of the mutation patterns of KRAS, BRAF and PIK3CA on the FAK-AKT signal. Second, only 2 cell lines were used *in vivo* models to verify the combination regimens, and more models should be used further. Third, the effect of tumor microenvironment, especially for multiple immune cells, is not involved in the study. However, we have described the adaptive feedback of the AKT pathway through multiple RTKs or released FAK activation after SHP2 inhibition and confirmed that the reactivation can drive the resistance to SHP2 inhibition in both *in vivo* and *in vitro* models. The combination of SHP2 inhibitors with the inhibitors of either FAK or AKT pathway may represent a promising therapeutic approach against CRC.

## Data Availability

The raw data supporting the conclusions of this article will be made available by the authors, without undue reservation.
