# Supplementary figures and images for: Therapeutic Suppression of FAK-AKT Signaling Overcomes Resistance to SHP2 Inhibition in Colorectal Carcinoma (part 1 of 2)
Source: Front Pharmacol. 2021 Nov 1;12:739501. doi: 10.3389/fphar.2021.739501 (PMC8591248; doi:10.3389/fphar.2021.739501)

FigureS1

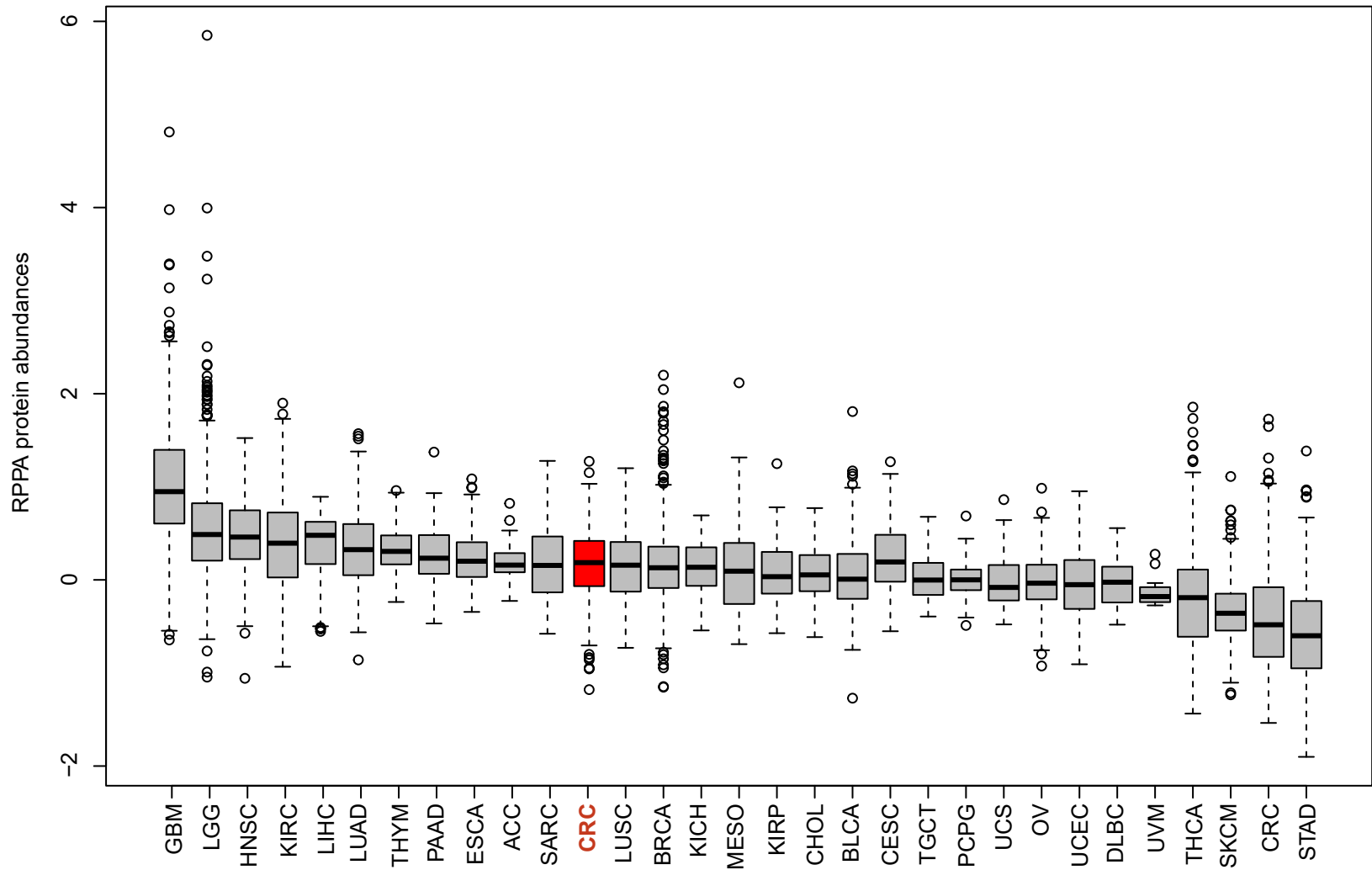

Supplement: Supplementary file 1 [file DataSheet2.PDF]

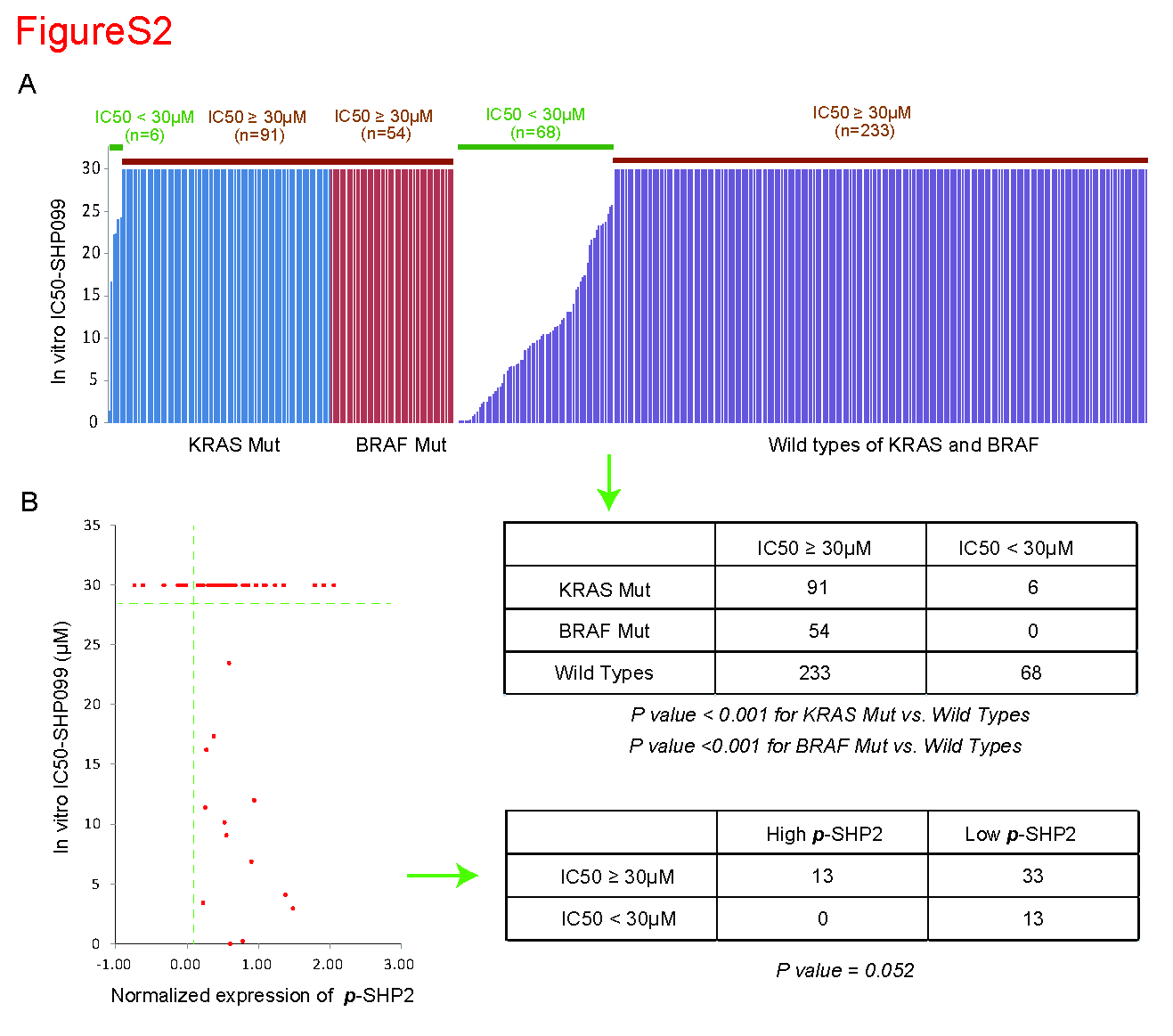

Supplement: Supplementary file 2 [file Image1.TIFF]

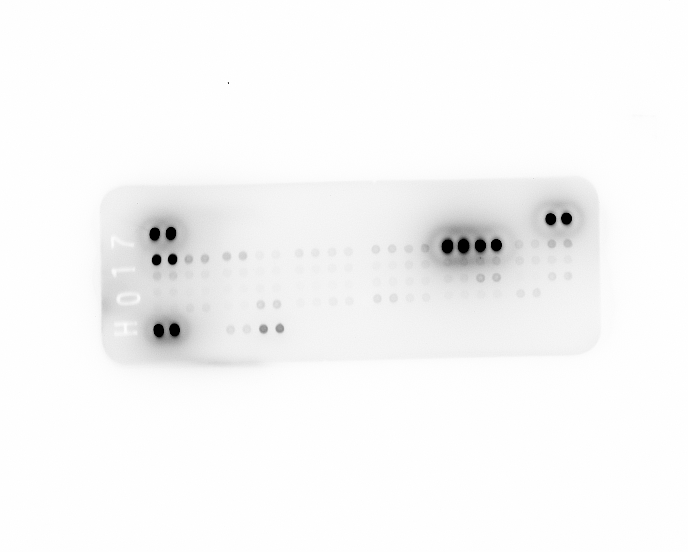

Supplement: Supplementary file 3 [file DataSheet8.ZIP › Figure5/A/Caco-2-DMSO-600S.tif]

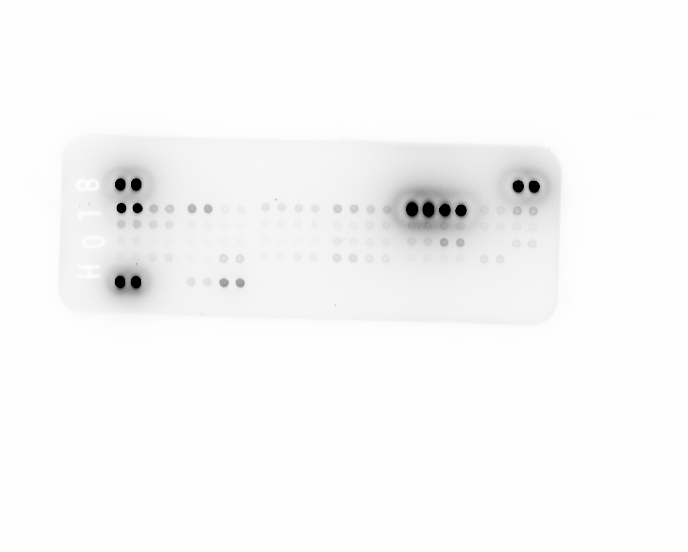

Supplement: Supplementary file 3 [file DataSheet8.ZIP › Figure5/A/Caco-2-SHP099-600S.tif]

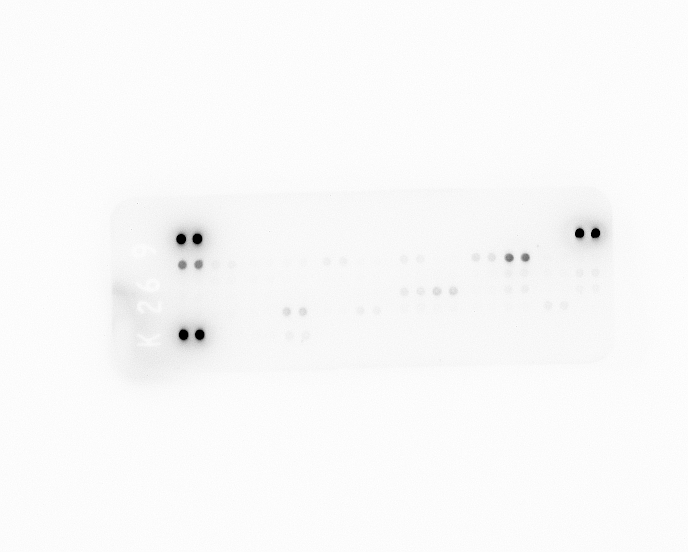

Supplement: Supplementary file 3 [file DataSheet8.ZIP › Figure5/A/Colo205-DMSO-300S.tif]

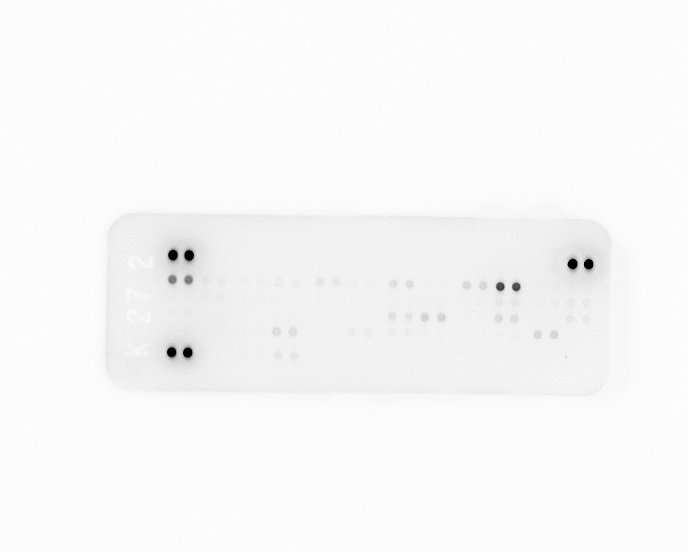

Supplement: Supplementary file 3 [file DataSheet8.ZIP › Figure5/A/Colo205-SHP099-300S.tif]

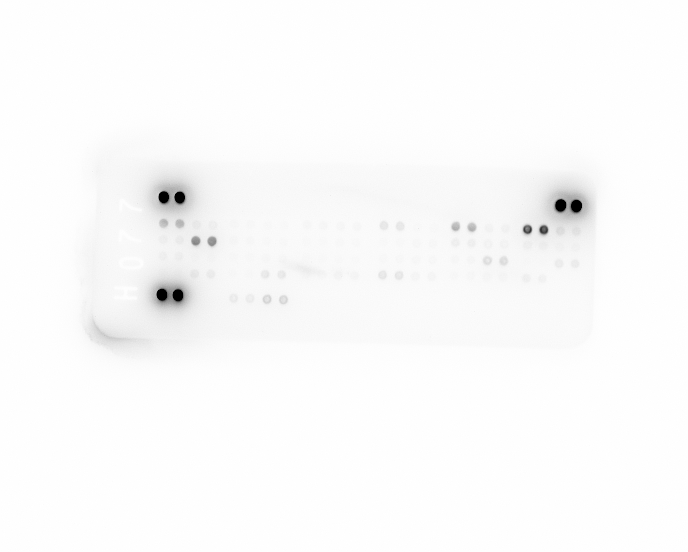

Supplement: Supplementary file 3 [file DataSheet8.ZIP › Figure5/A/RKO-DMSO-600S.tif]

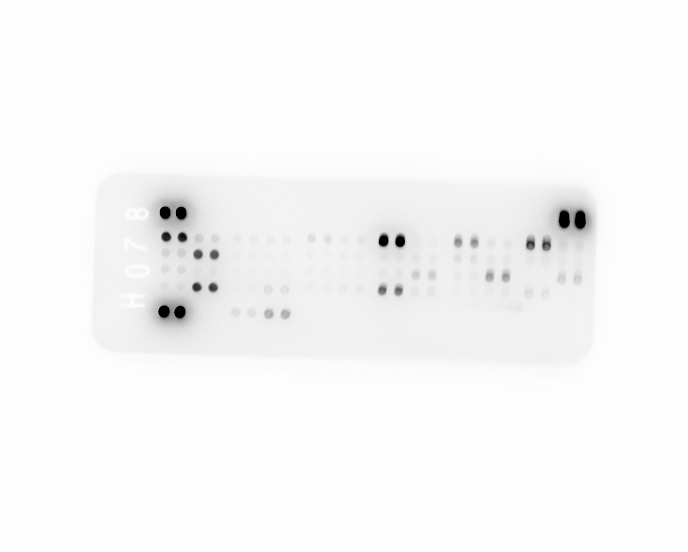

Supplement: Supplementary file 3 [file DataSheet8.ZIP › Figure5/A/RKO-SHP099-600S.tif]

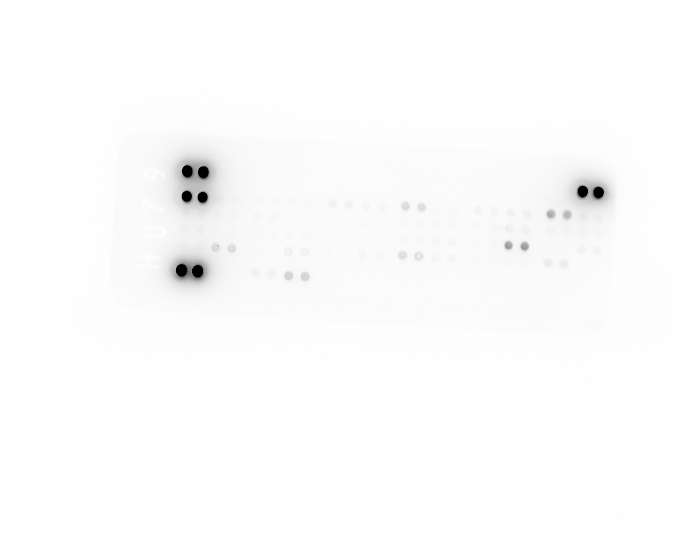

Supplement: Supplementary file 3 [file DataSheet8.ZIP › Figure5/A/SW480-DMSO-300S.tif]

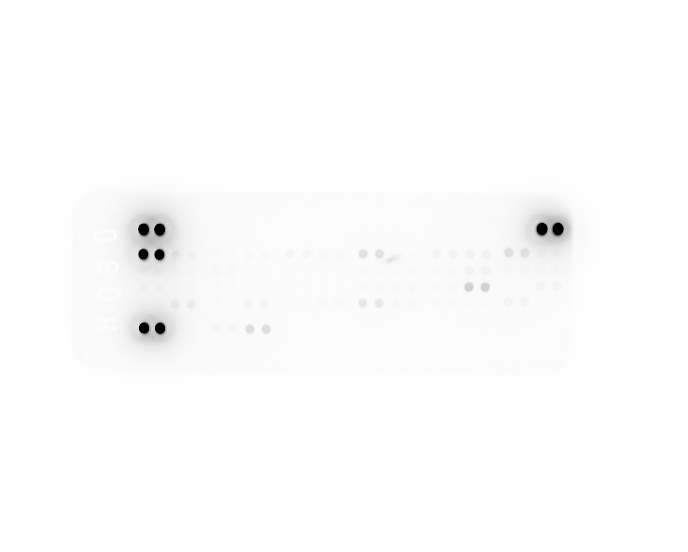

Supplement: Supplementary file 3 [file DataSheet8.ZIP › Figure5/A/SW480-SHP099-300S.tif]

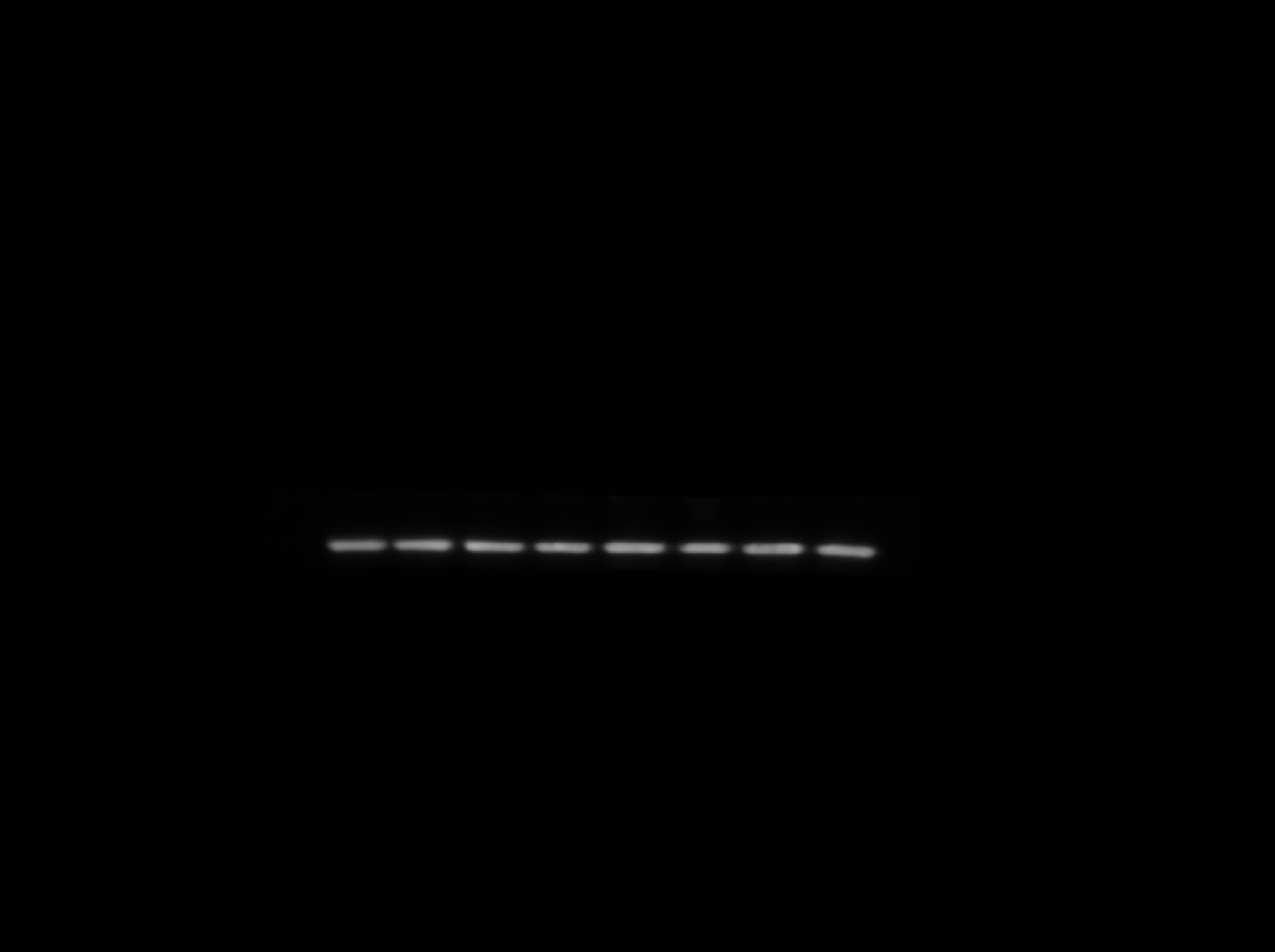

Supplement: Supplementary file 4 [file DataSheet9.ZIP › Figure6/Figure6A/GAPDH Colo205.jpg]

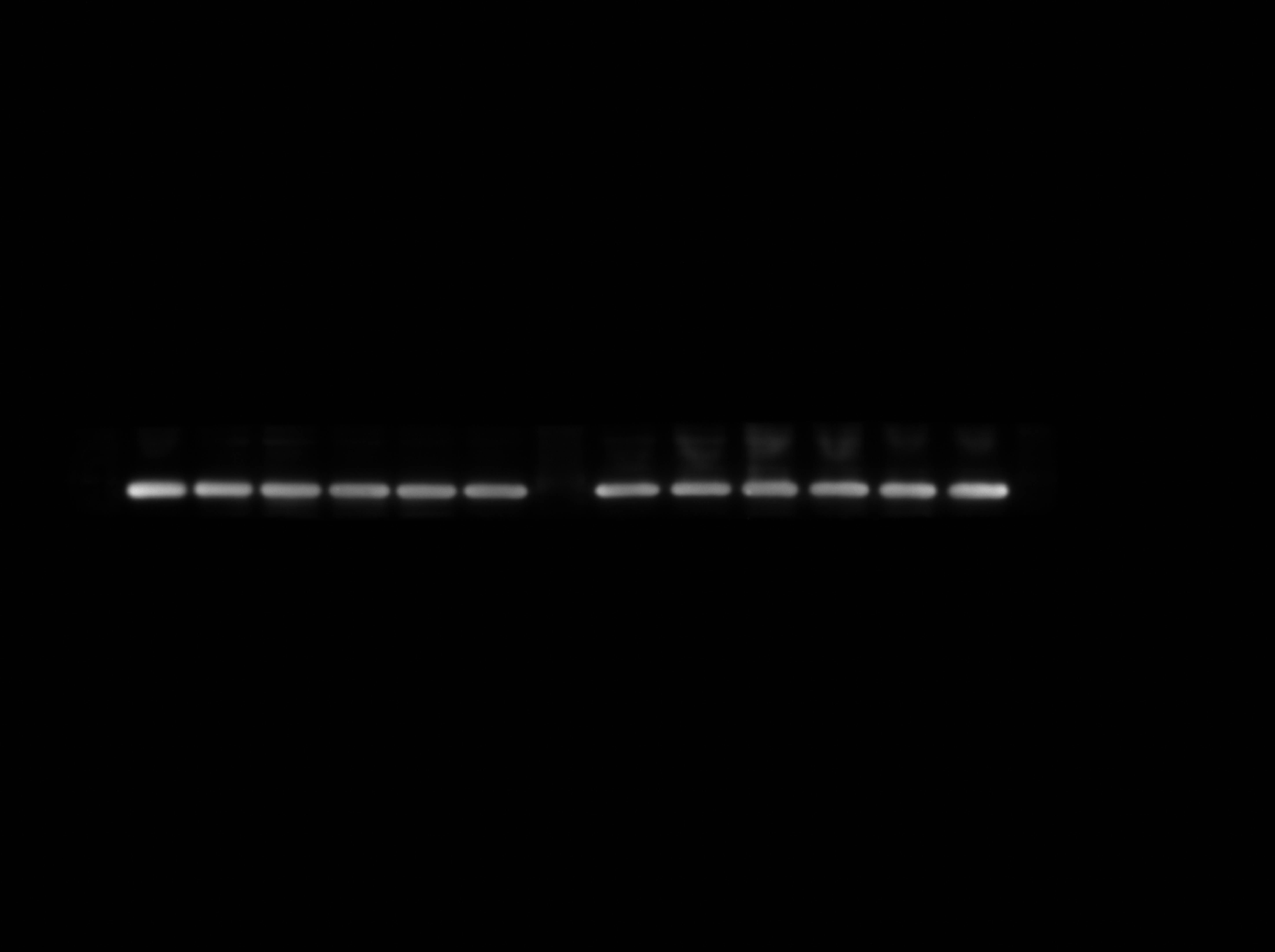

Supplement: Supplementary file 4 [file DataSheet9.ZIP › Figure6/Figure6A/GAPDH SW620 12h+24h.jpg]

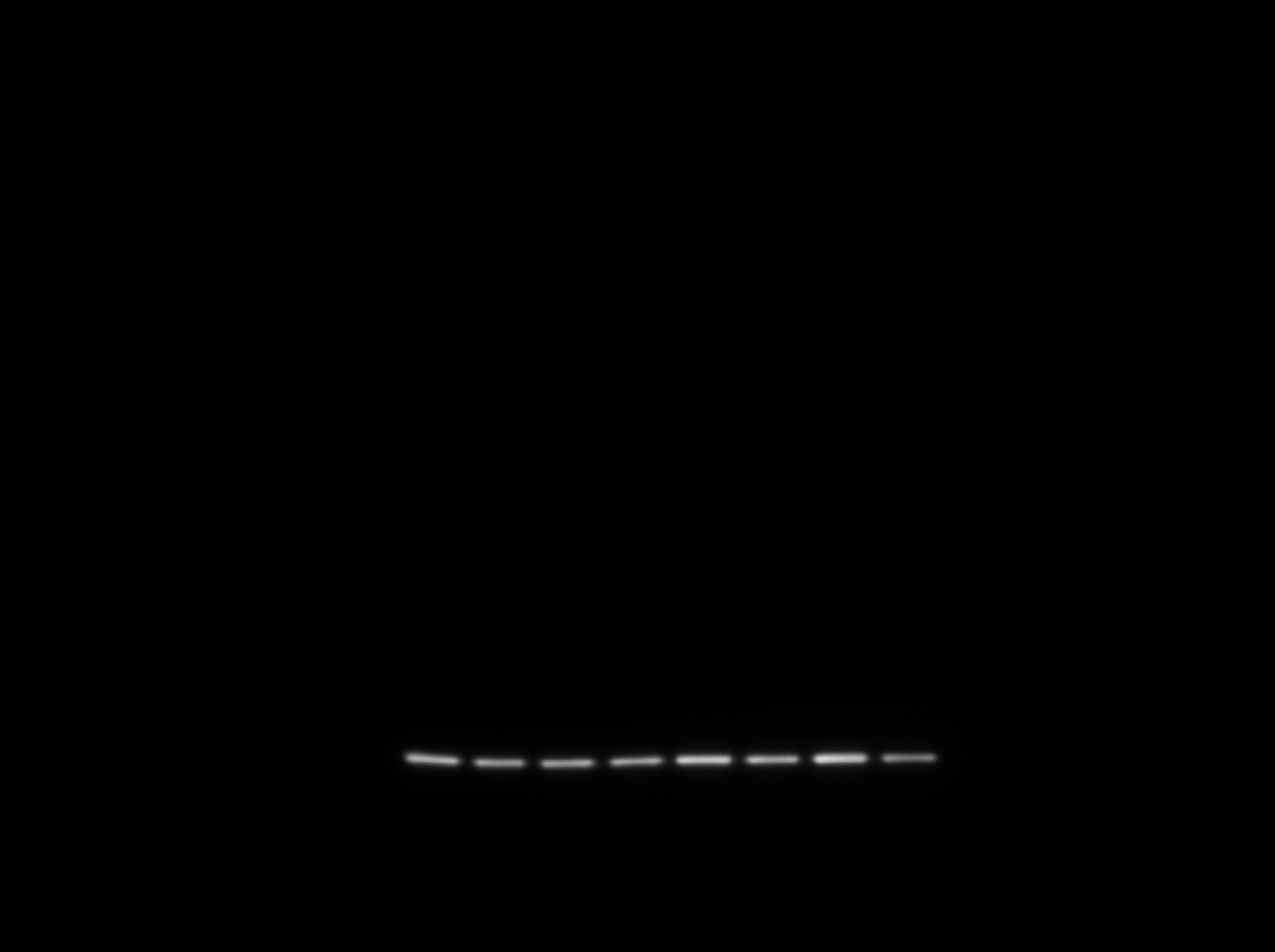

Supplement: Supplementary file 4 [file DataSheet9.ZIP › Figure6/Figure6A/GAPDH SW620 30min+1h.jpg]

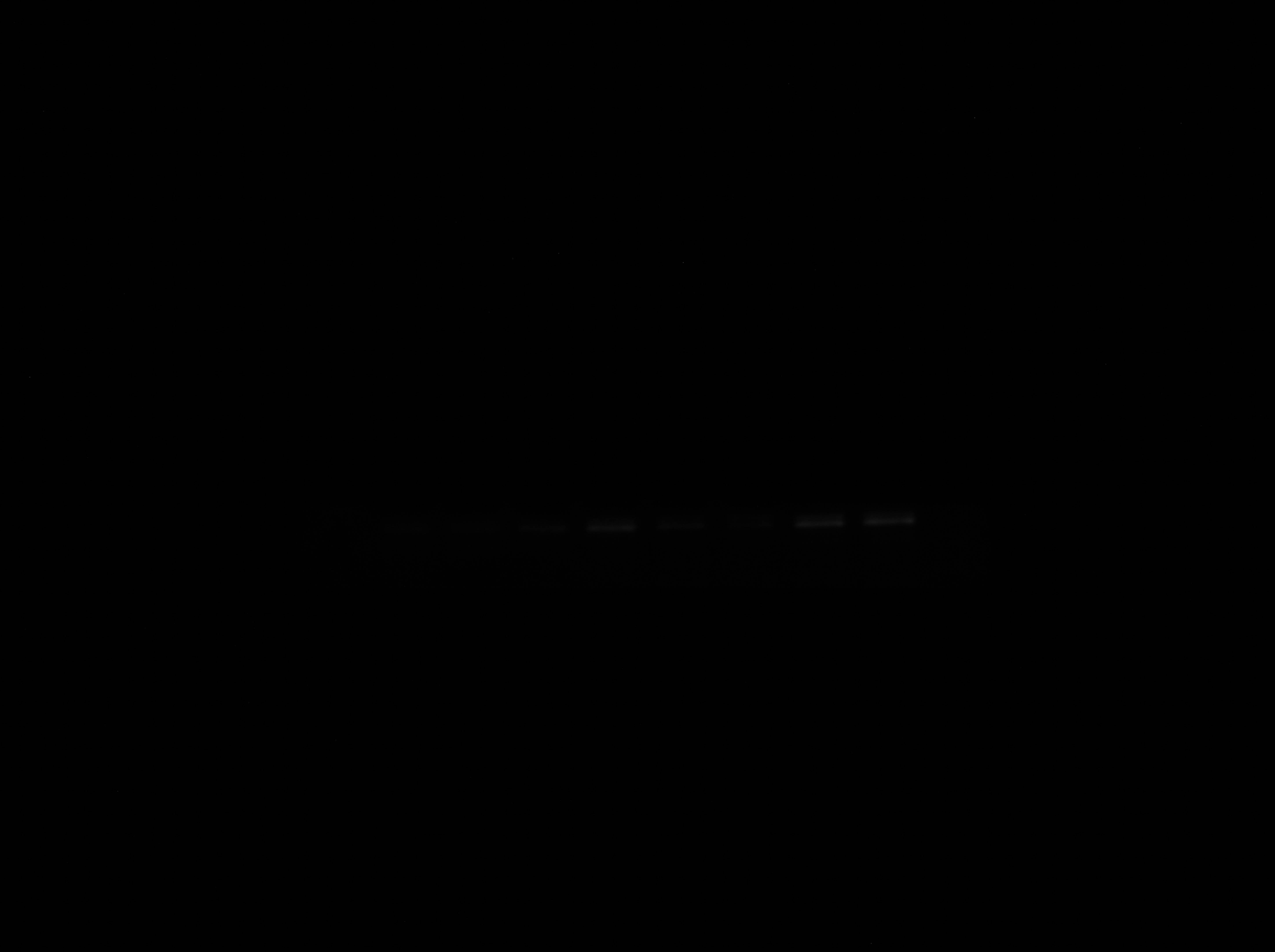

Supplement: Supplementary file 4 [file DataSheet9.ZIP › Figure6/Figure6A/P-AKT Colo205.jpg]

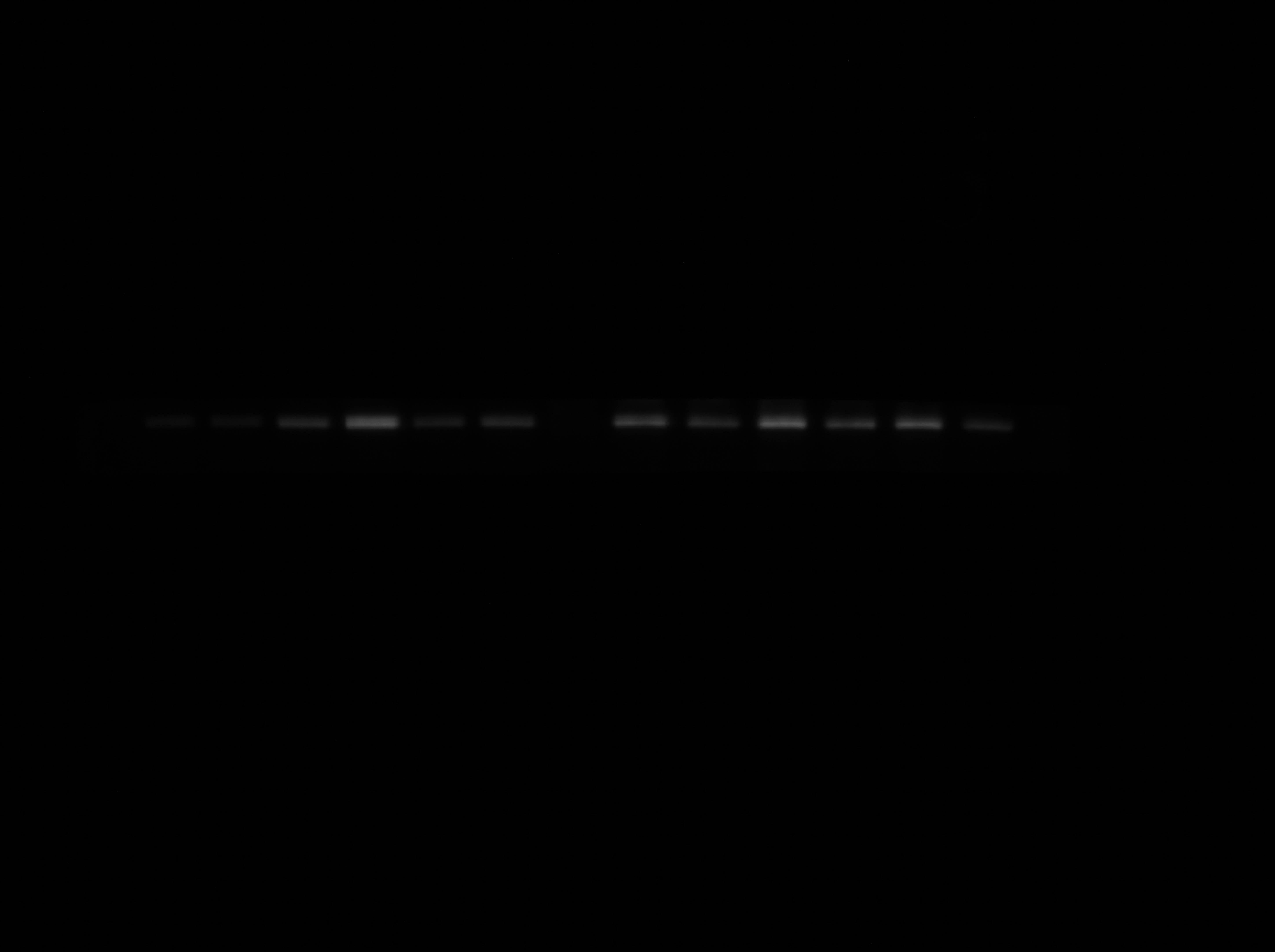

Supplement: Supplementary file 4 [file DataSheet9.ZIP › Figure6/Figure6A/P-AKT SW620 12h+24h.jpg]

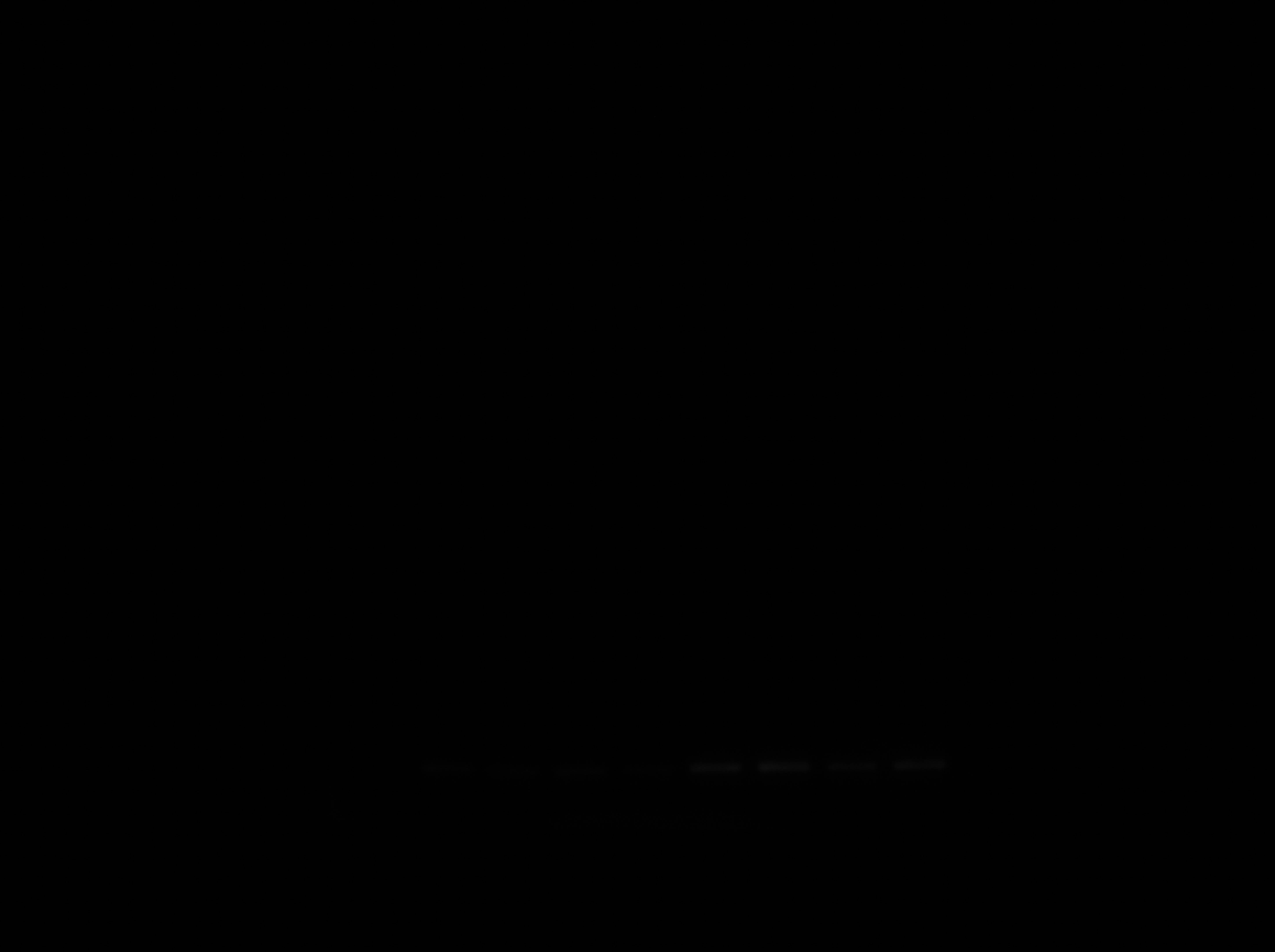

Supplement: Supplementary file 4 [file DataSheet9.ZIP › Figure6/Figure6A/P-AKT SW620 30min+1h.jpg]

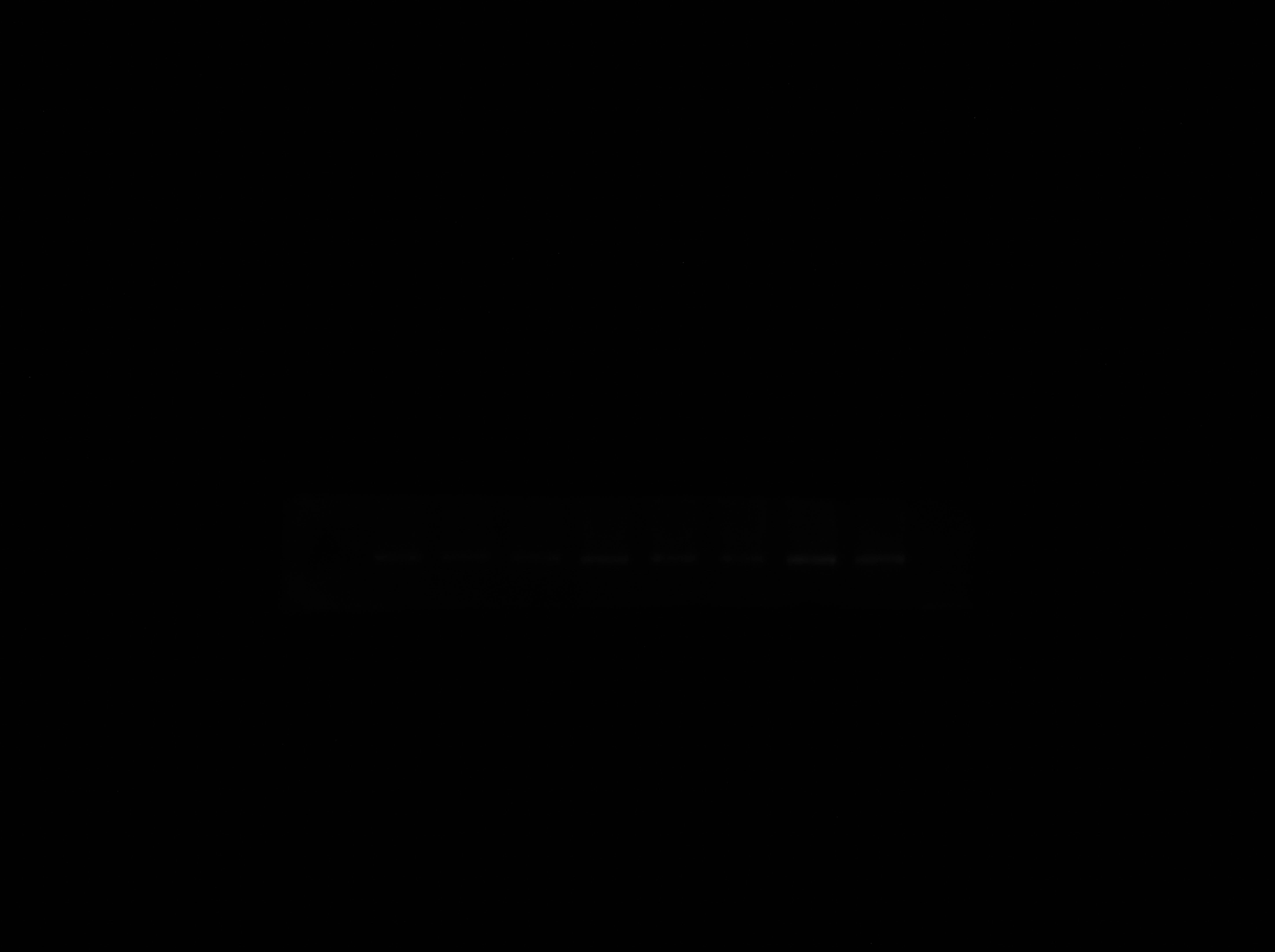

Supplement: Supplementary file 4 [file DataSheet9.ZIP › Figure6/Figure6A/P-FAK Colo205.jpg]

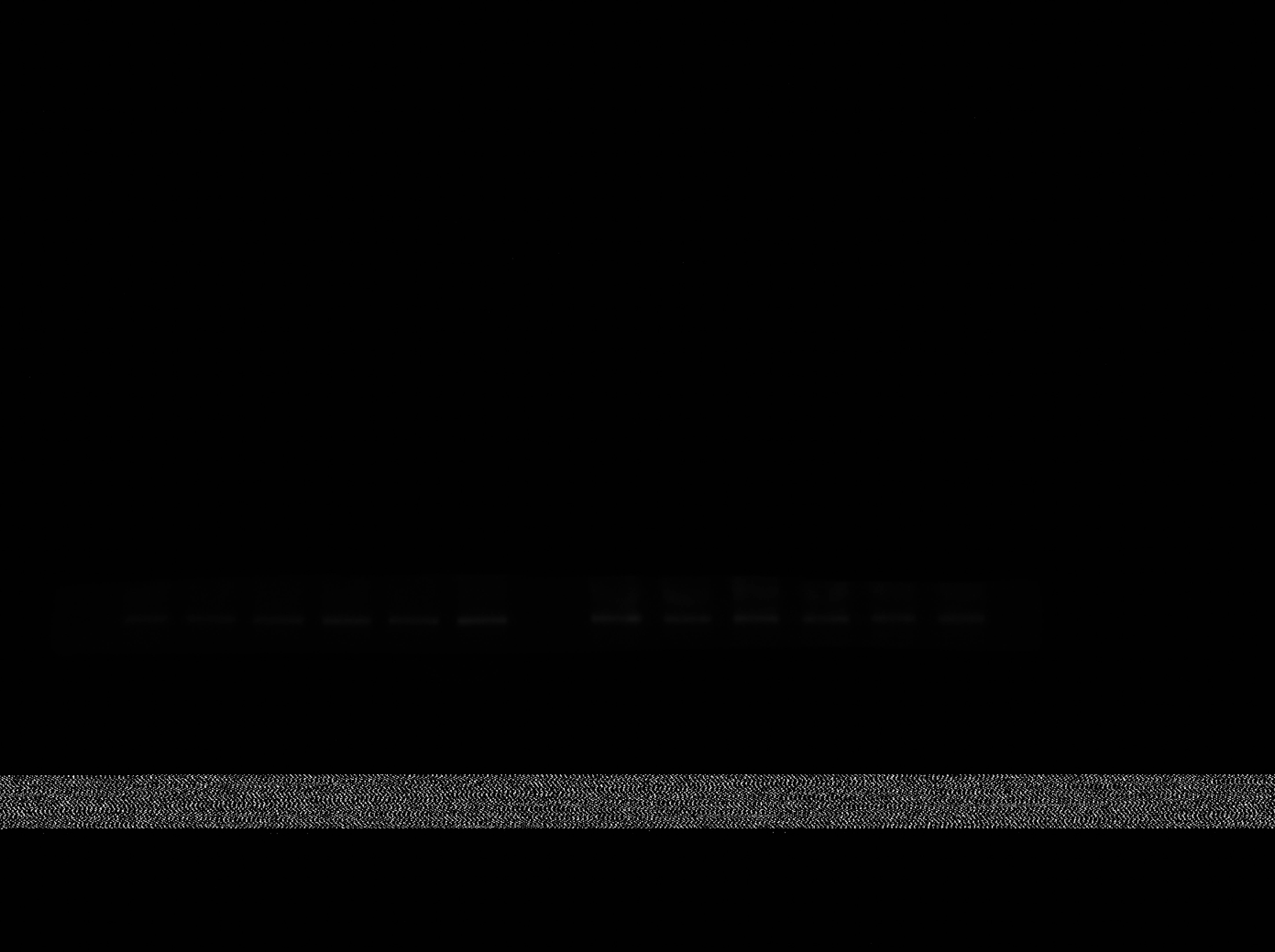

Supplement: Supplementary file 4 [file DataSheet9.ZIP › Figure6/Figure6A/P-FAK SW620 12h+24h.jpg]

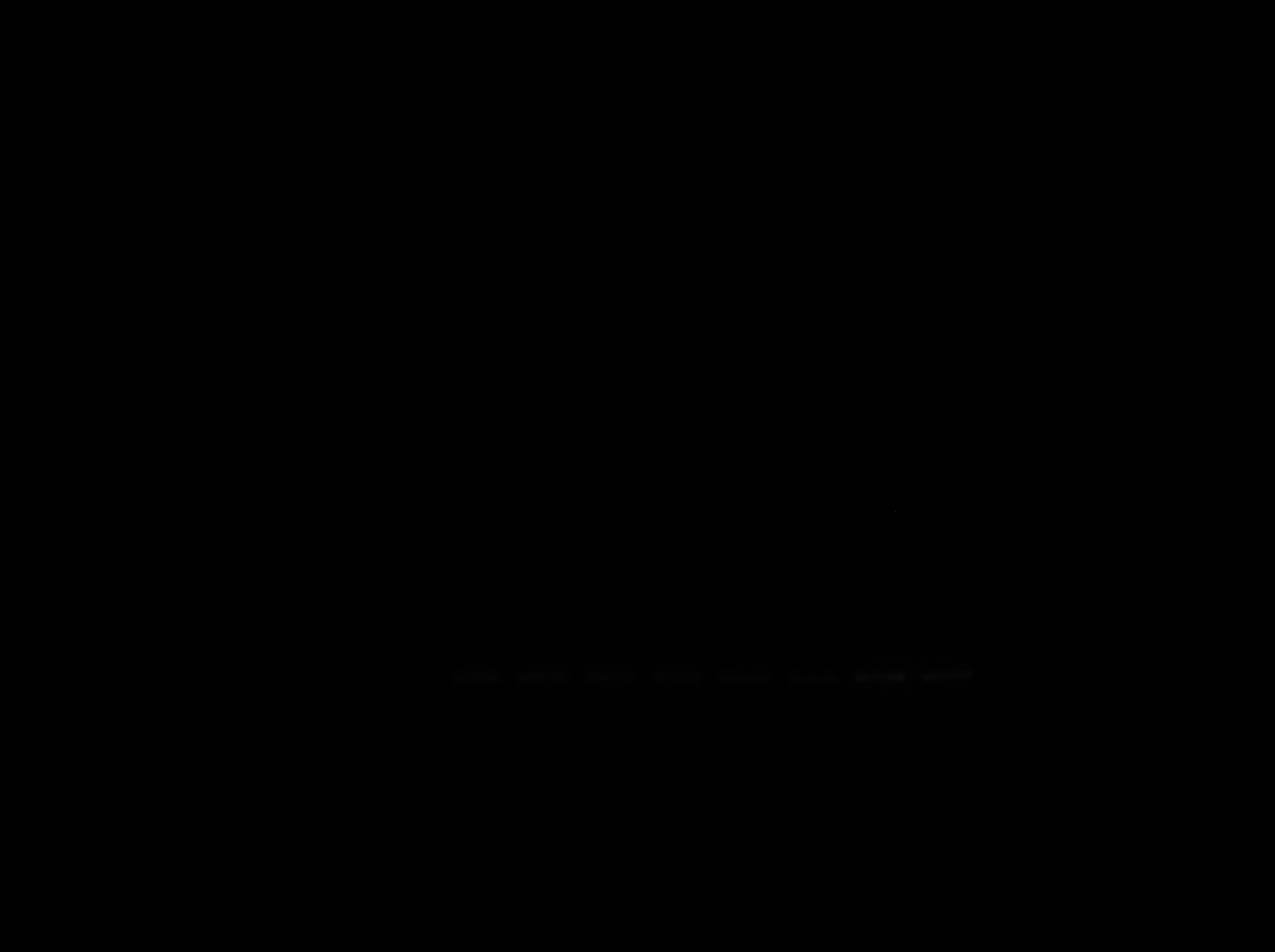

Supplement: Supplementary file 4 [file DataSheet9.ZIP › Figure6/Figure6A/P-FAK SW620 30min+1h.jpg]

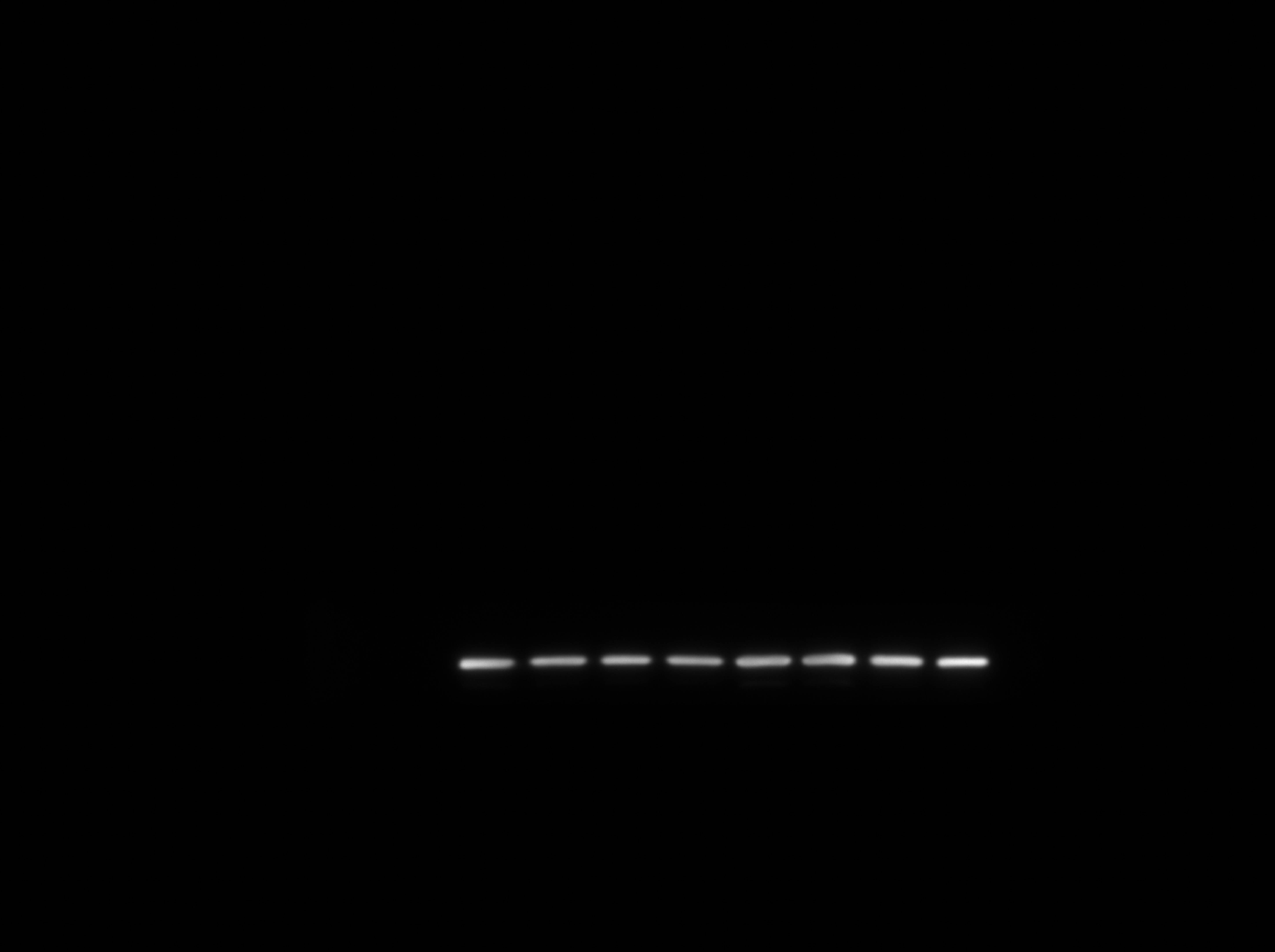

Supplement: Supplementary file 4 [file DataSheet9.ZIP › Figure6/Figure6B/GAPDH Colo205 12h+24h.jpg]

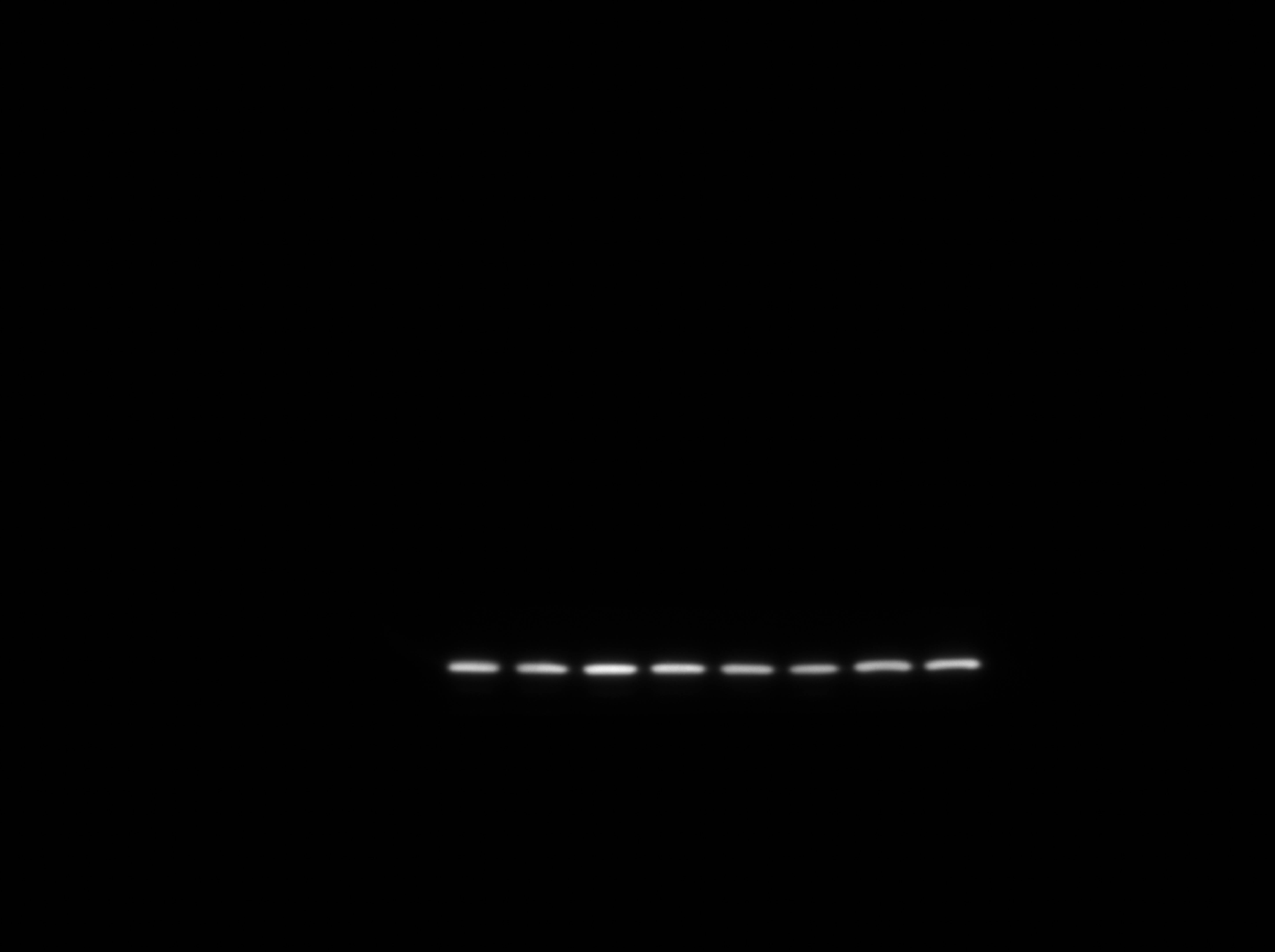

Supplement: Supplementary file 4 [file DataSheet9.ZIP › Figure6/Figure6B/GAPDH Colo205 1h+3h.jpg]

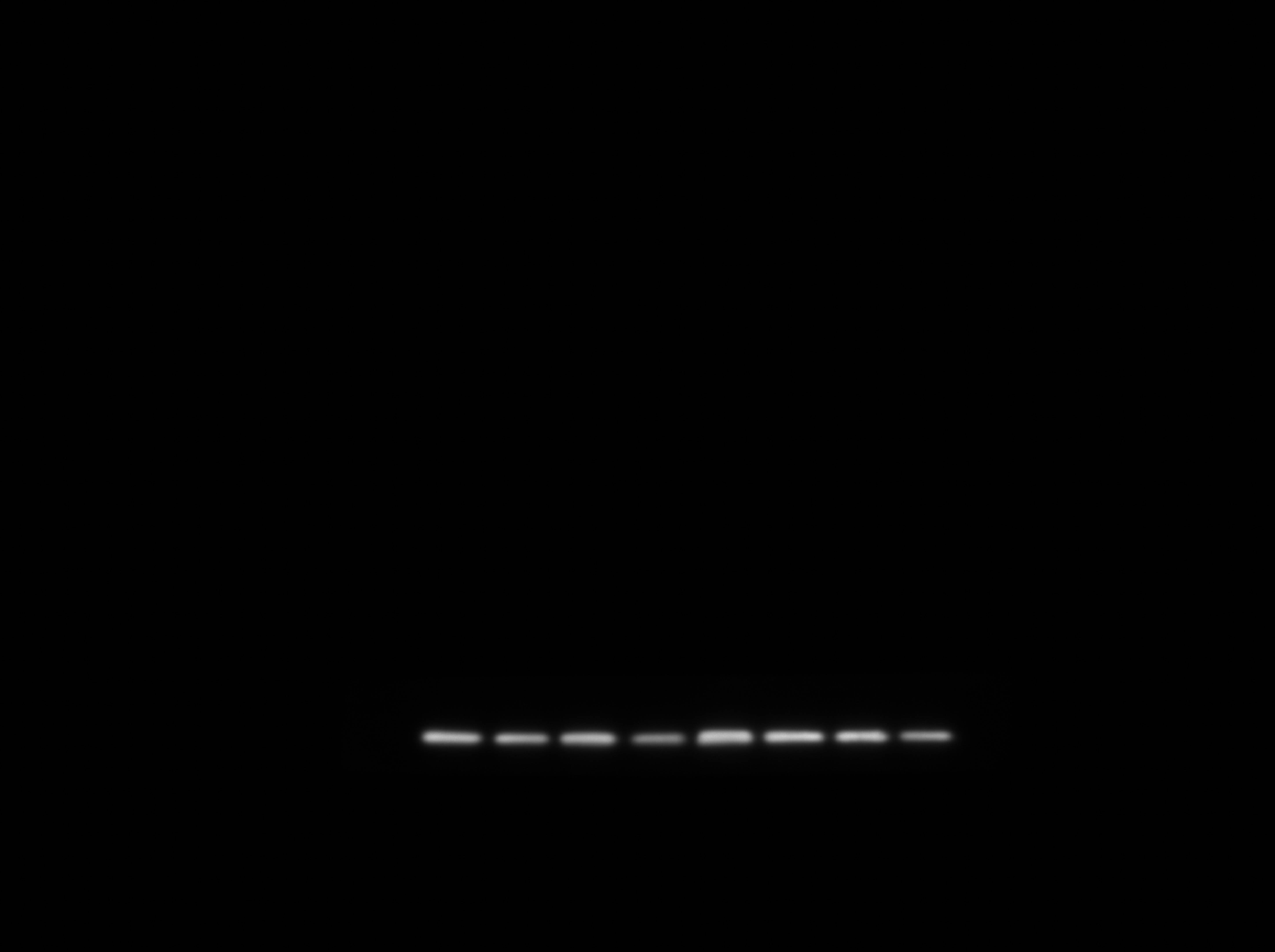

Supplement: Supplementary file 4 [file DataSheet9.ZIP › Figure6/Figure6B/GAPDH SW620 12h+24h.jpg]

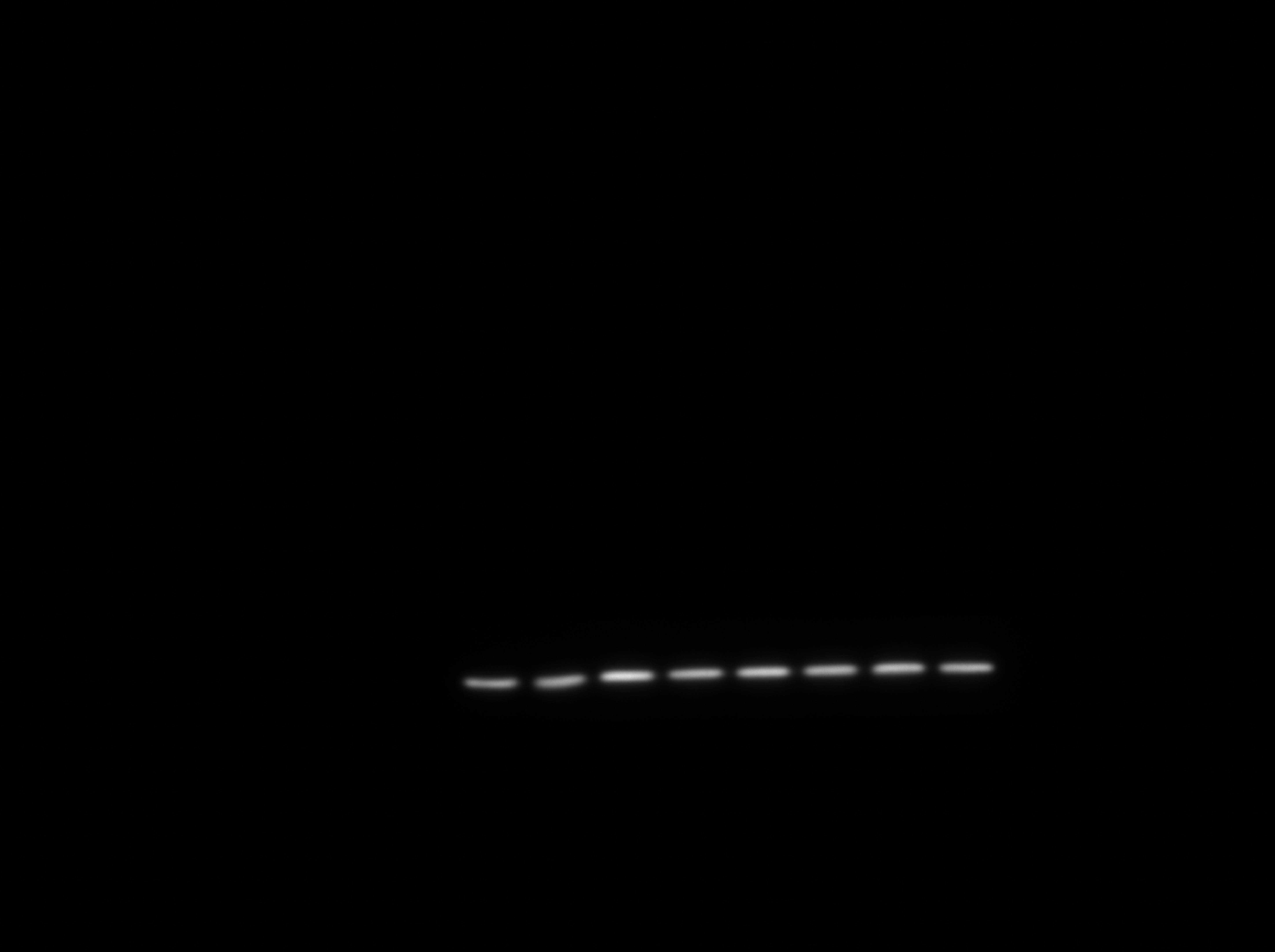

Supplement: Supplementary file 4 [file DataSheet9.ZIP › Figure6/Figure6B/GAPDH SW620 1h+3h.jpg]

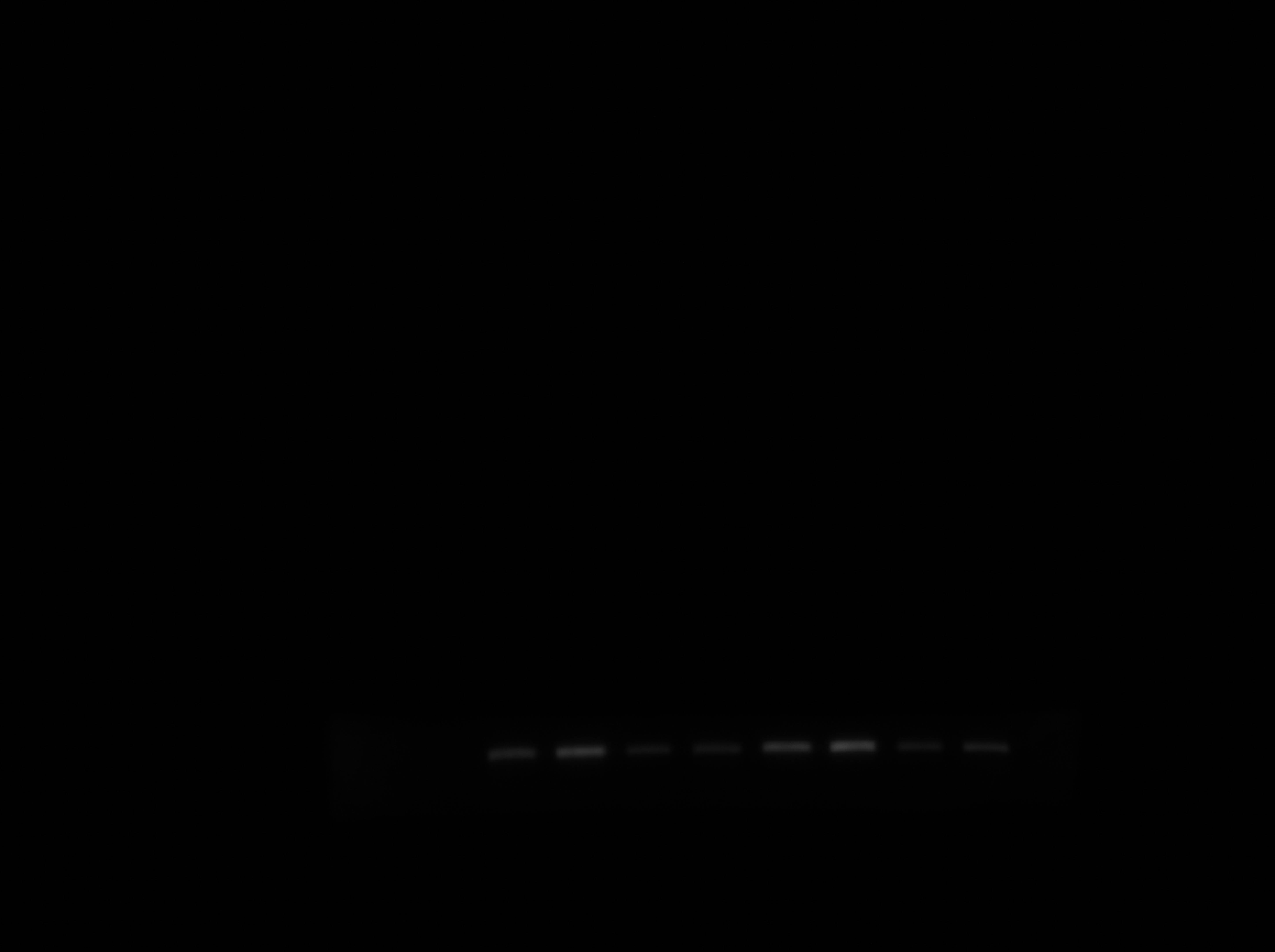

Supplement: Supplementary file 4 [file DataSheet9.ZIP › Figure6/Figure6B/P-AKT Colo205 12h+24h.jpg]

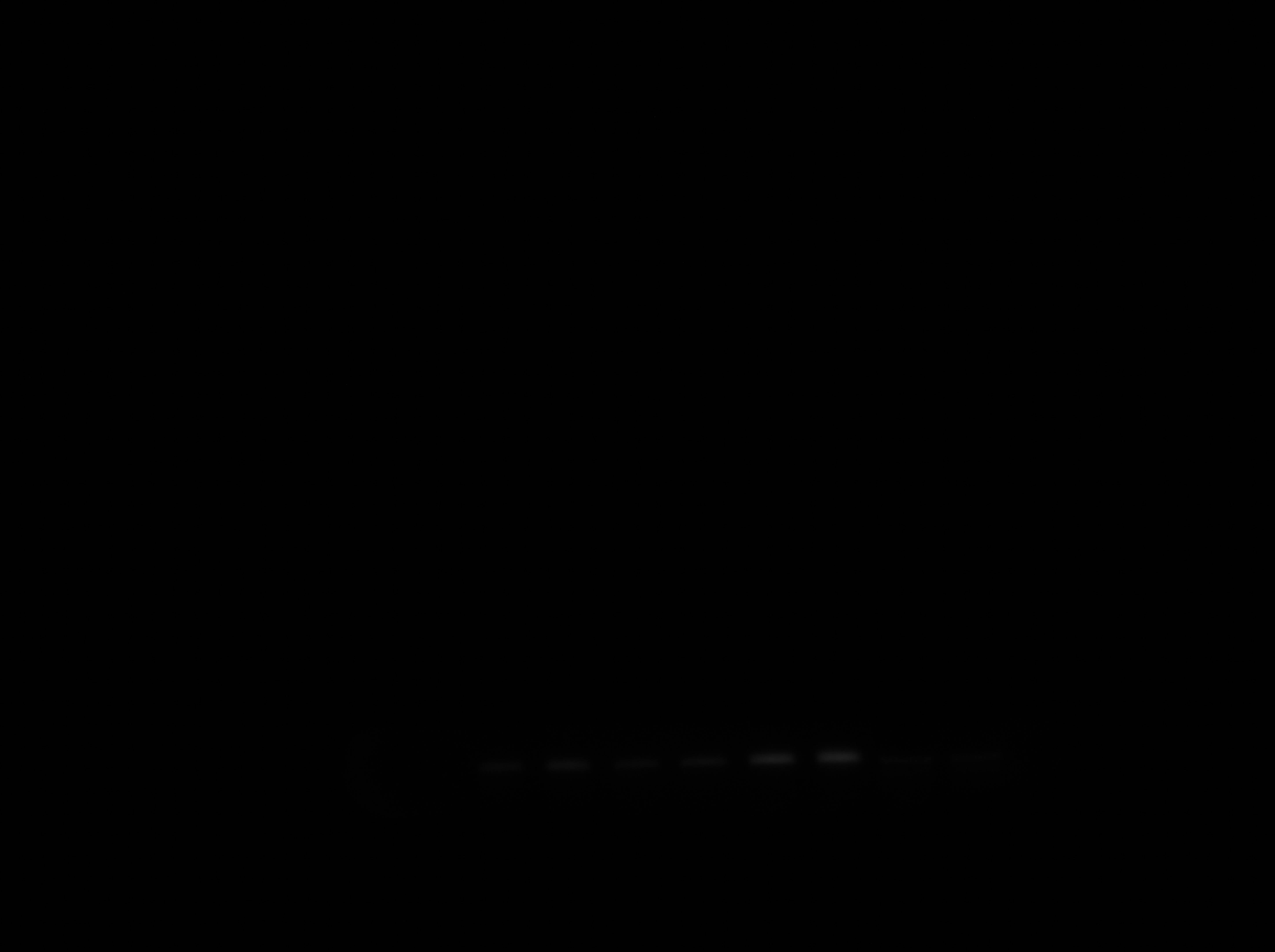

Supplement: Supplementary file 4 [file DataSheet9.ZIP › Figure6/Figure6B/P-AKT Colo205 1h+3h.jpg]

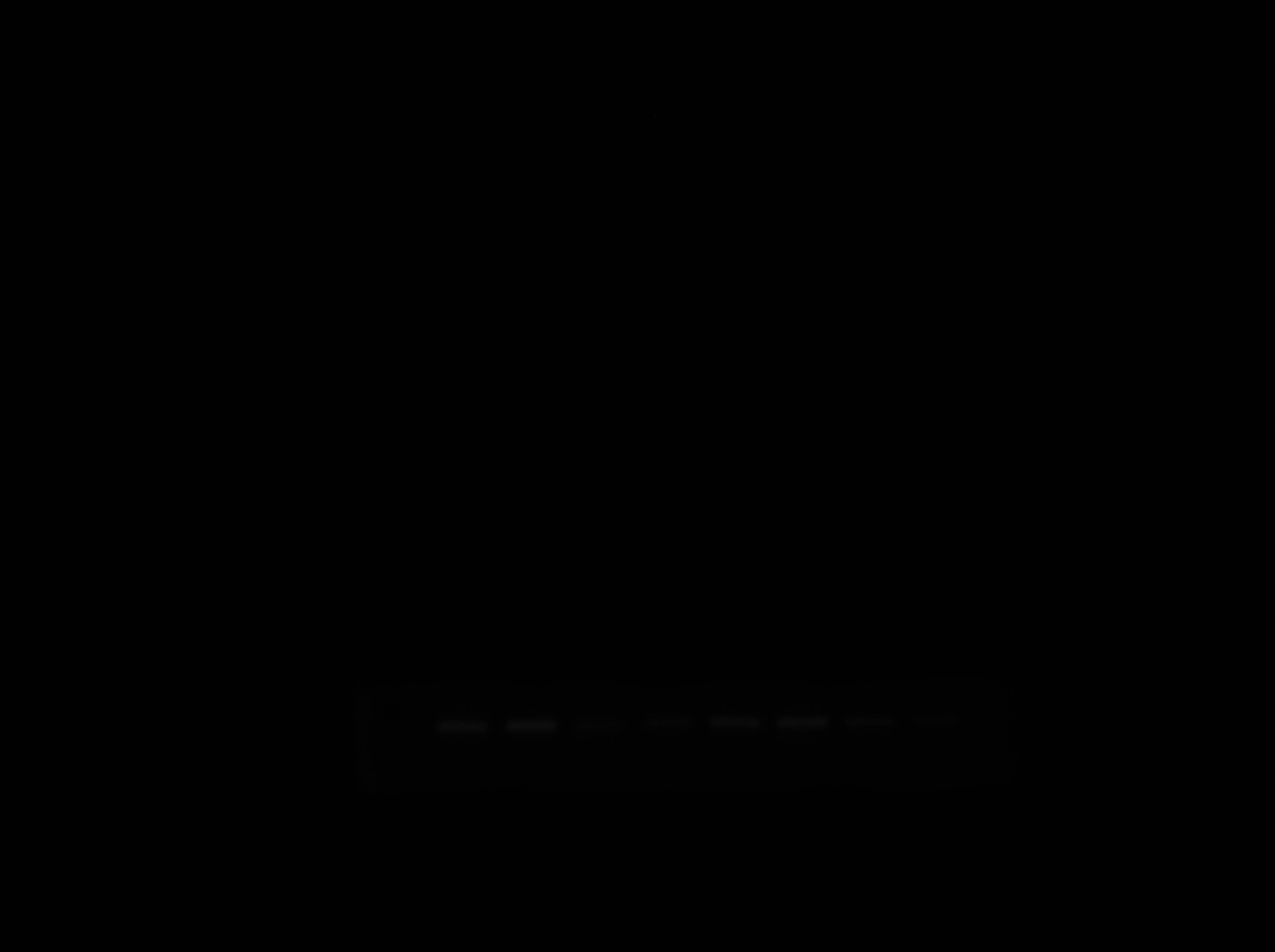

Supplement: Supplementary file 4 [file DataSheet9.ZIP › Figure6/Figure6B/P-AKT SW620 12h+24h.jpg]

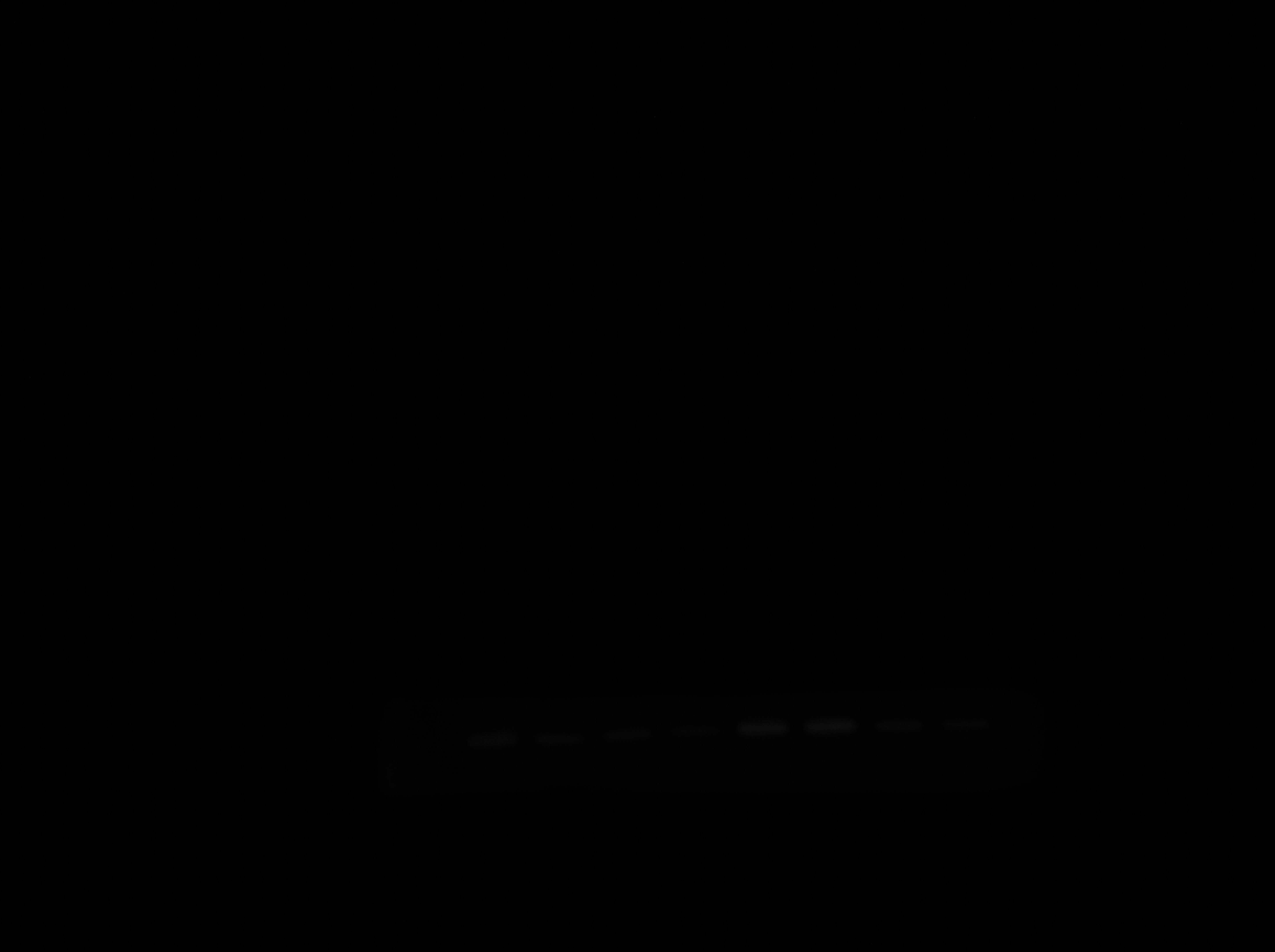

Supplement: Supplementary file 4 [file DataSheet9.ZIP › Figure6/Figure6B/P-AKT SW620 1h+3h.jpg]

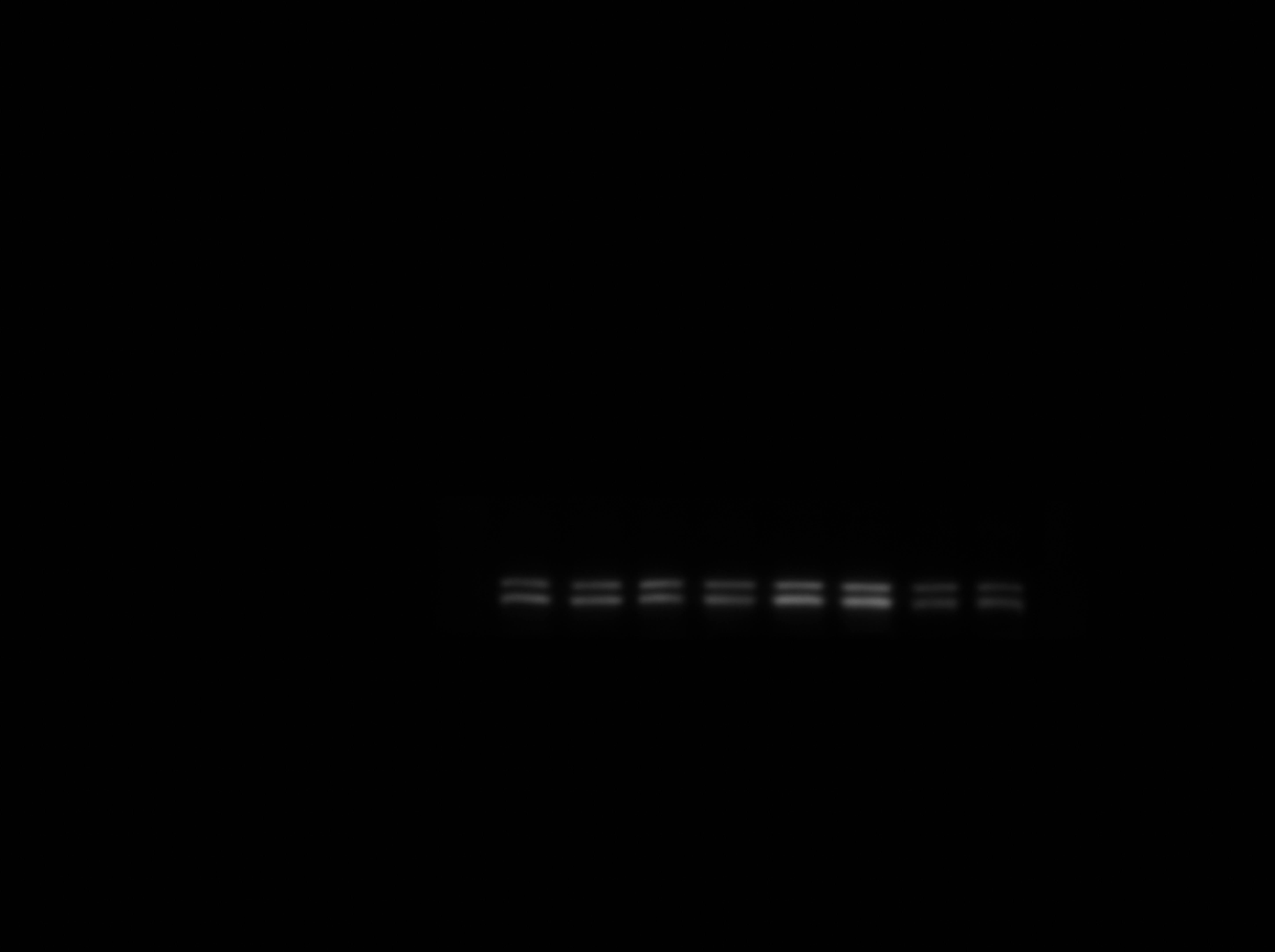

Supplement: Supplementary file 4 [file DataSheet9.ZIP › Figure6/Figure6B/P-ERK Colo205 12h+24h.jpg]

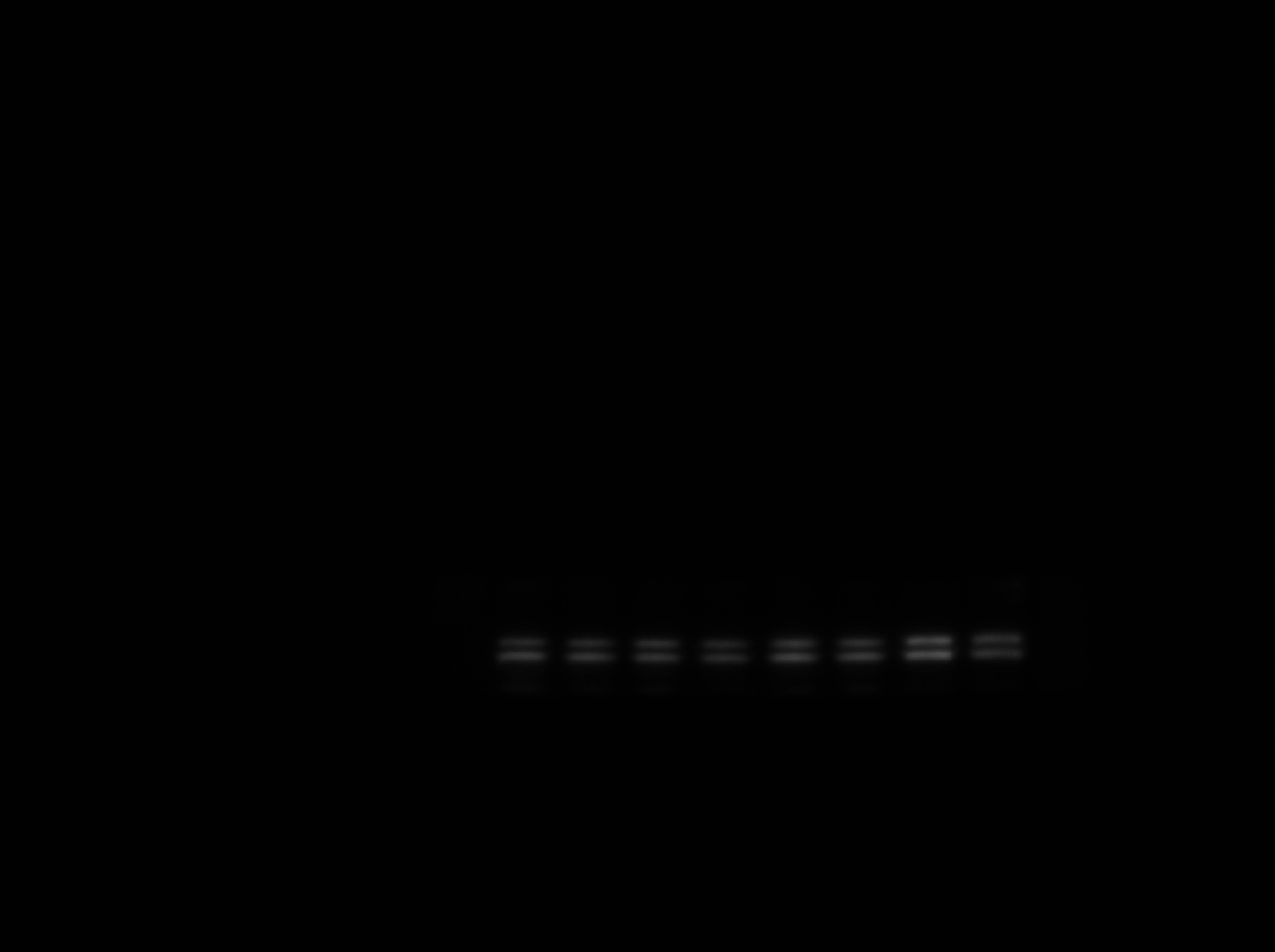

Supplement: Supplementary file 4 [file DataSheet9.ZIP › Figure6/Figure6B/P-ERK Colo205 1h+3h.jpg]

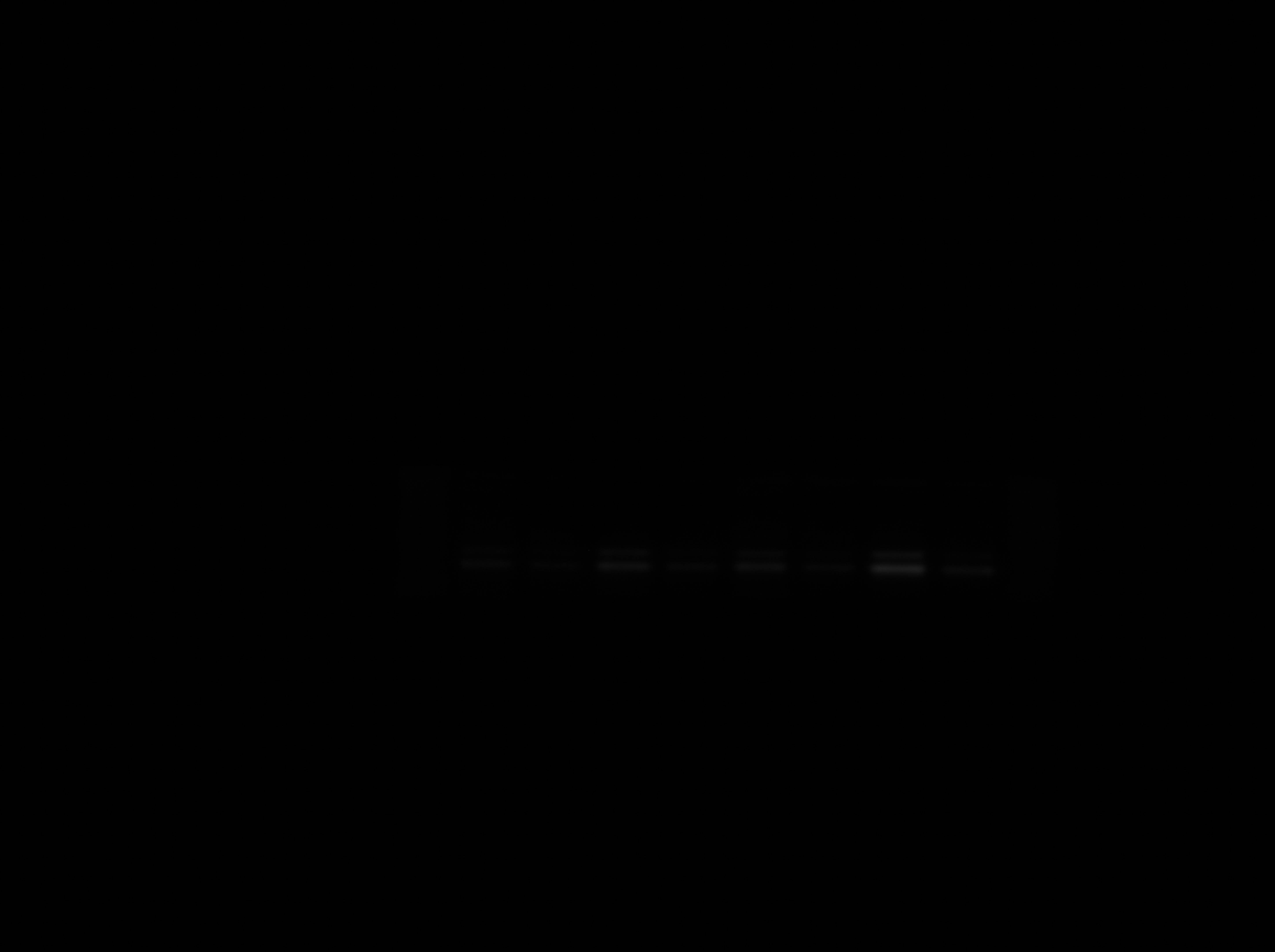

Supplement: Supplementary file 4 [file DataSheet9.ZIP › Figure6/Figure6B/P-ERK SW620 12h+24h.jpg]

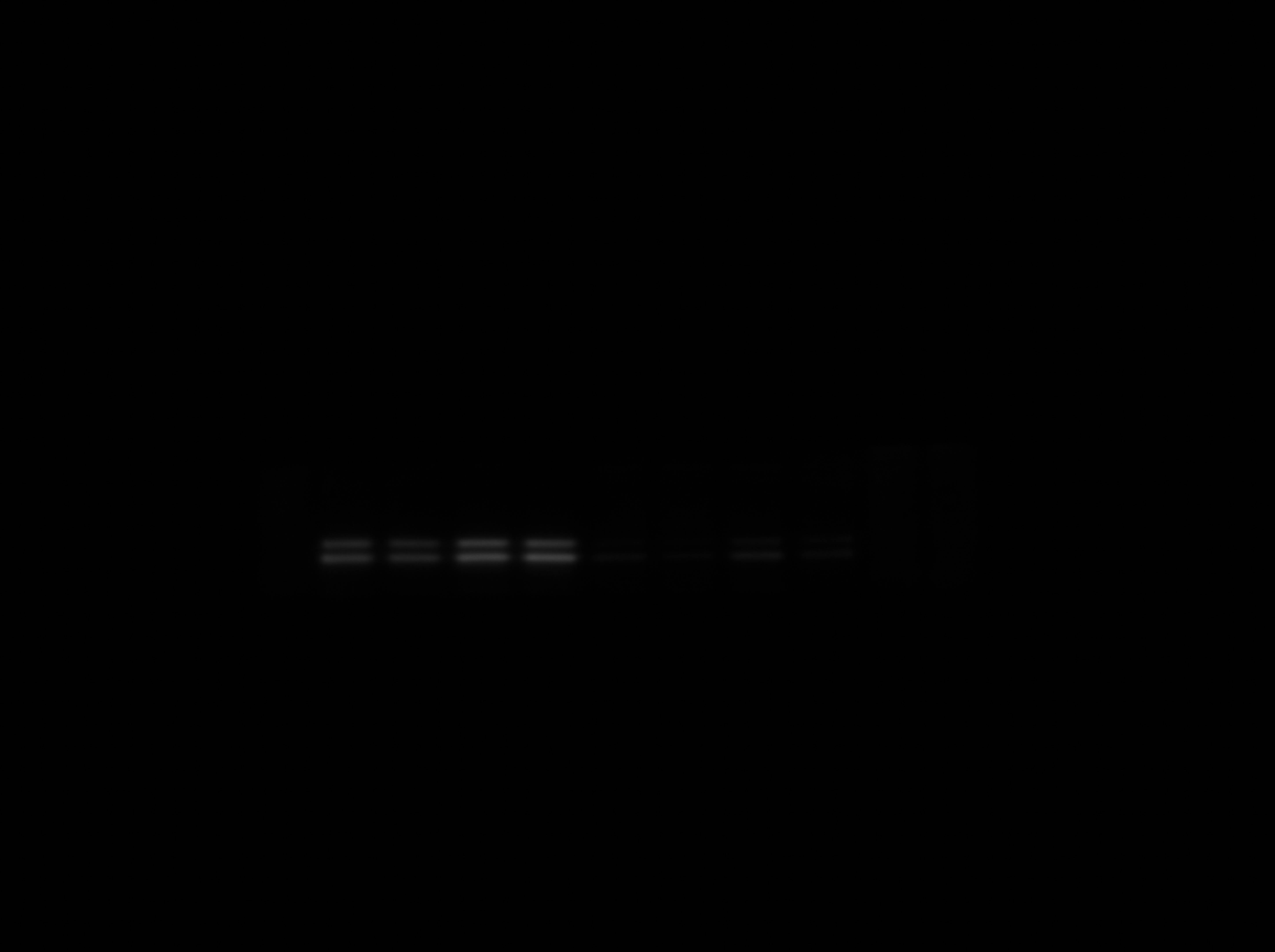

Supplement: Supplementary file 4 [file DataSheet9.ZIP › Figure6/Figure6B/P-ERK SW620 1h+3h.jpg]

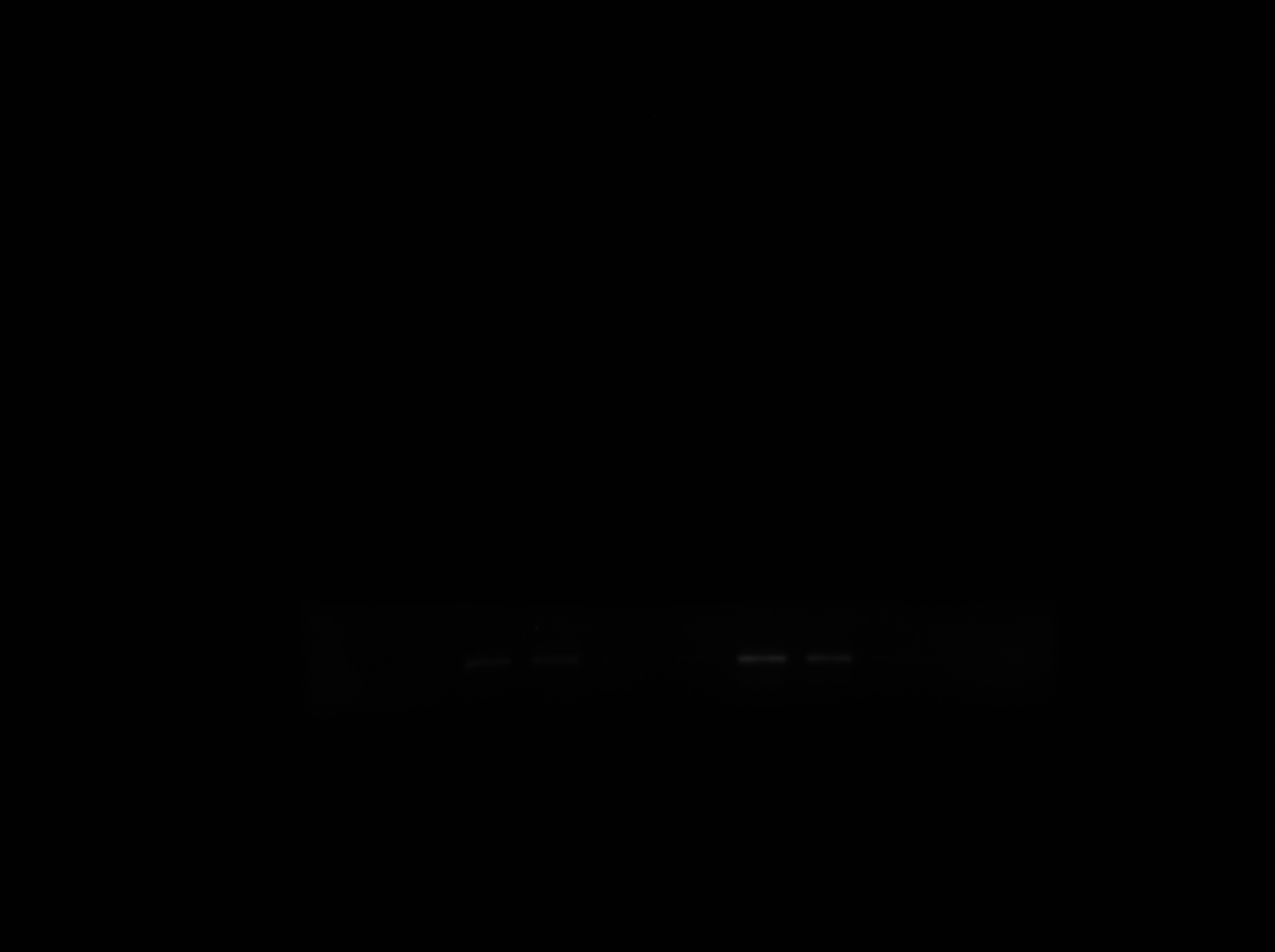

Supplement: Supplementary file 4 [file DataSheet9.ZIP › Figure6/Figure6B/P-FAK Colo205 12h+24h.jpg]

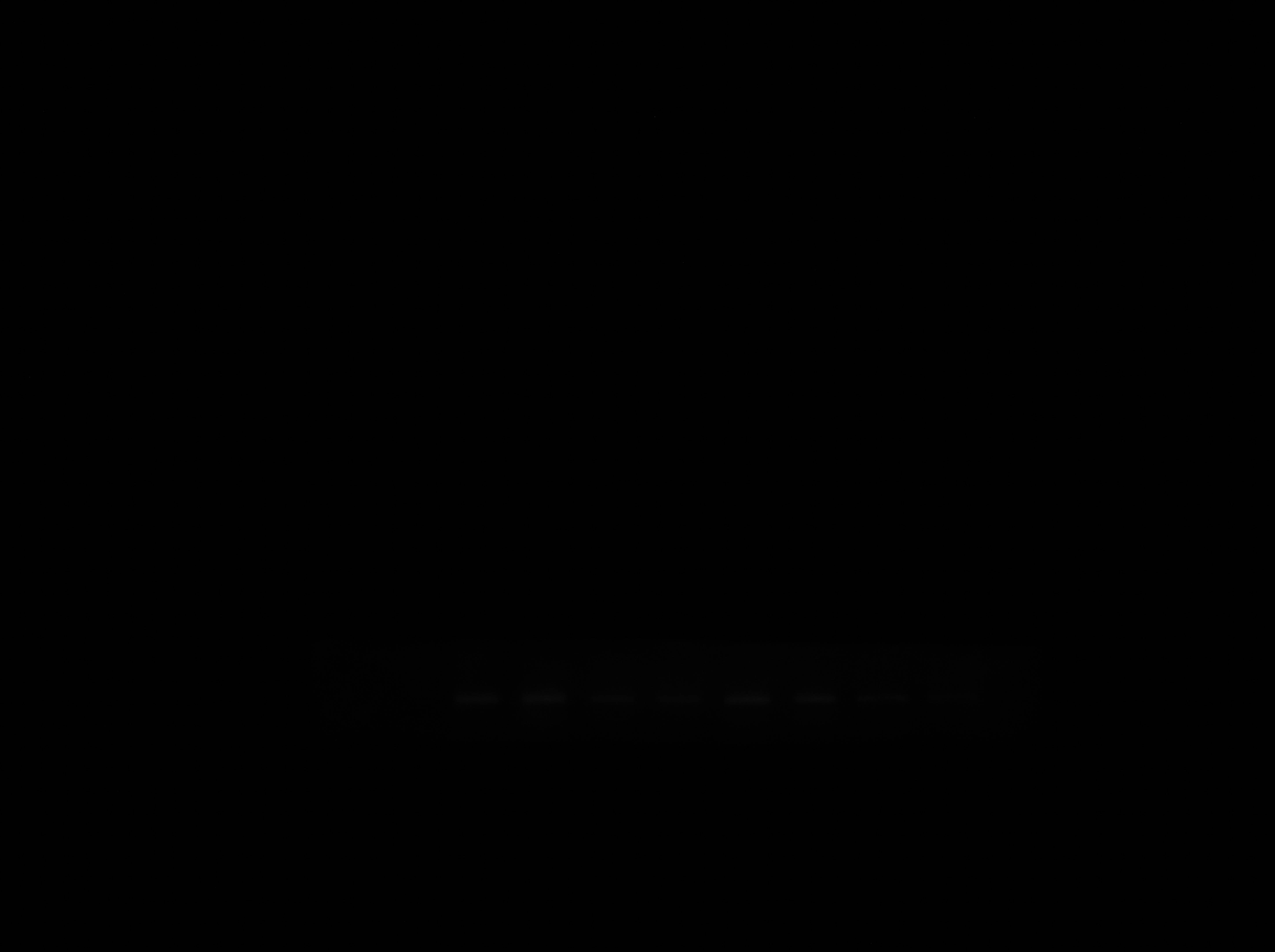

Supplement: Supplementary file 4 [file DataSheet9.ZIP › Figure6/Figure6B/P-FAK Colo205 1h+3h.jpg]

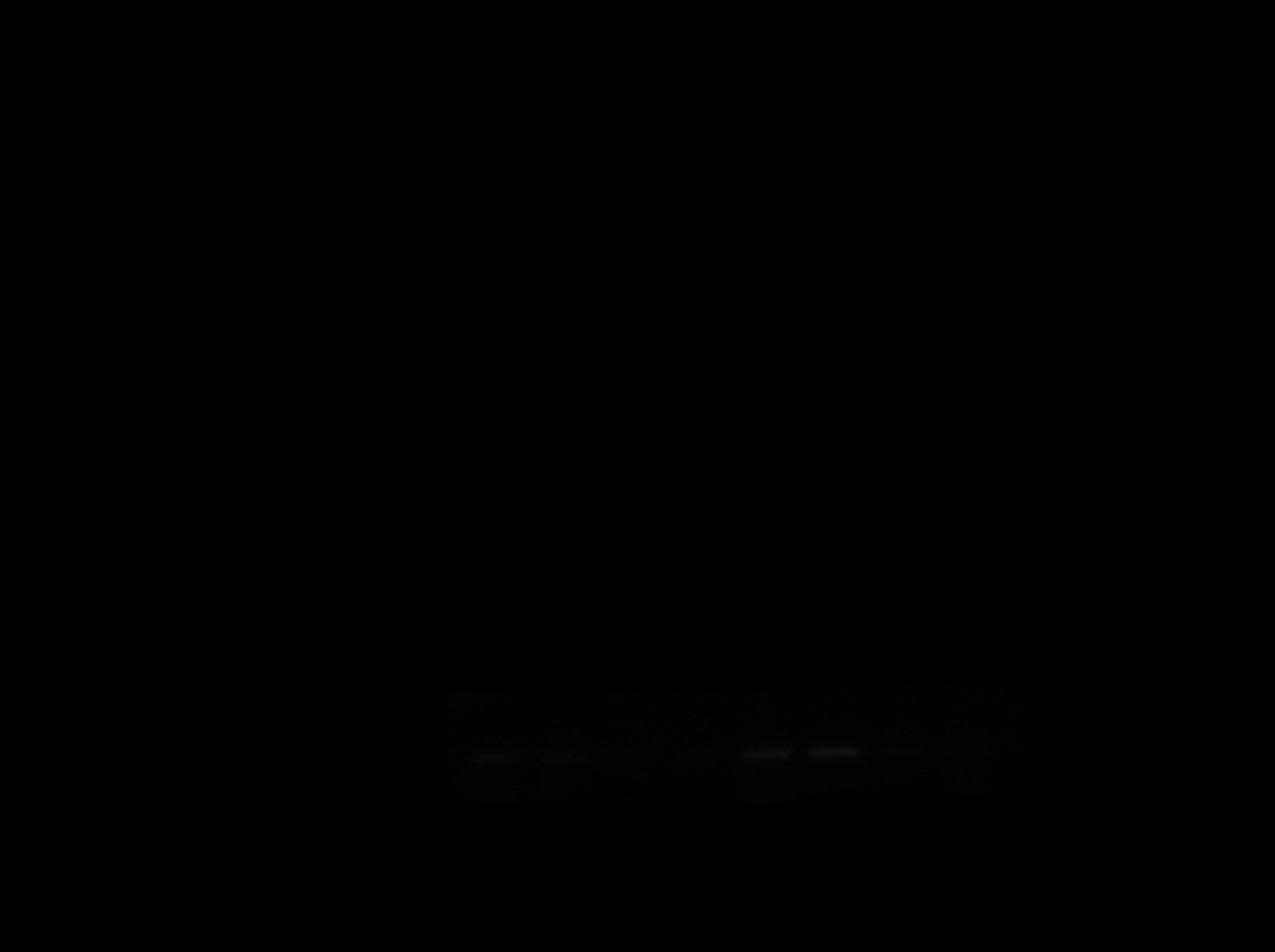

Supplement: Supplementary file 4 [file DataSheet9.ZIP › Figure6/Figure6B/P-FAK SW620 12h+24h.jpg]

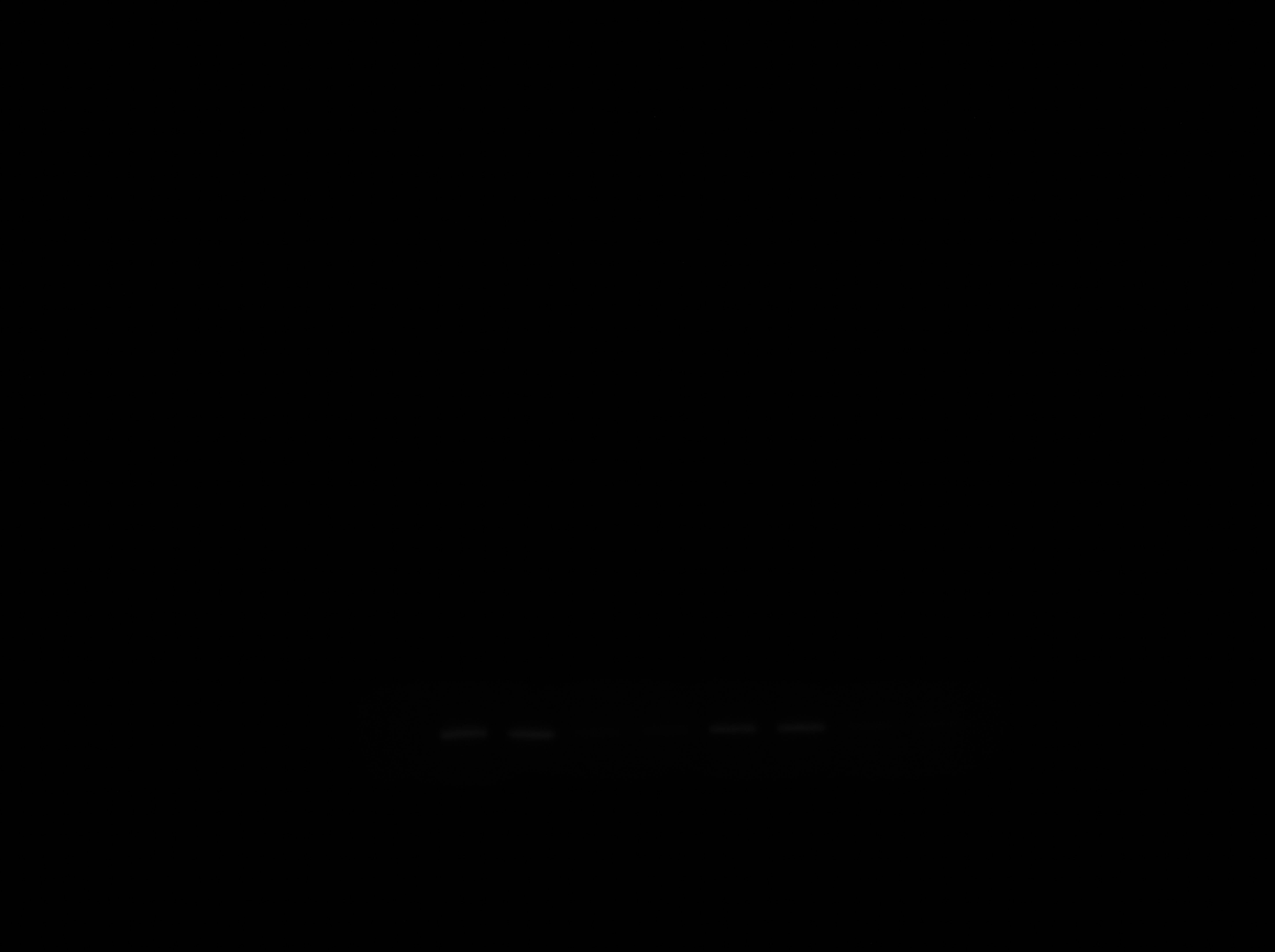

Supplement: Supplementary file 4 [file DataSheet9.ZIP › Figure6/Figure6B/P-FAK SW620 1h+3h.jpg]

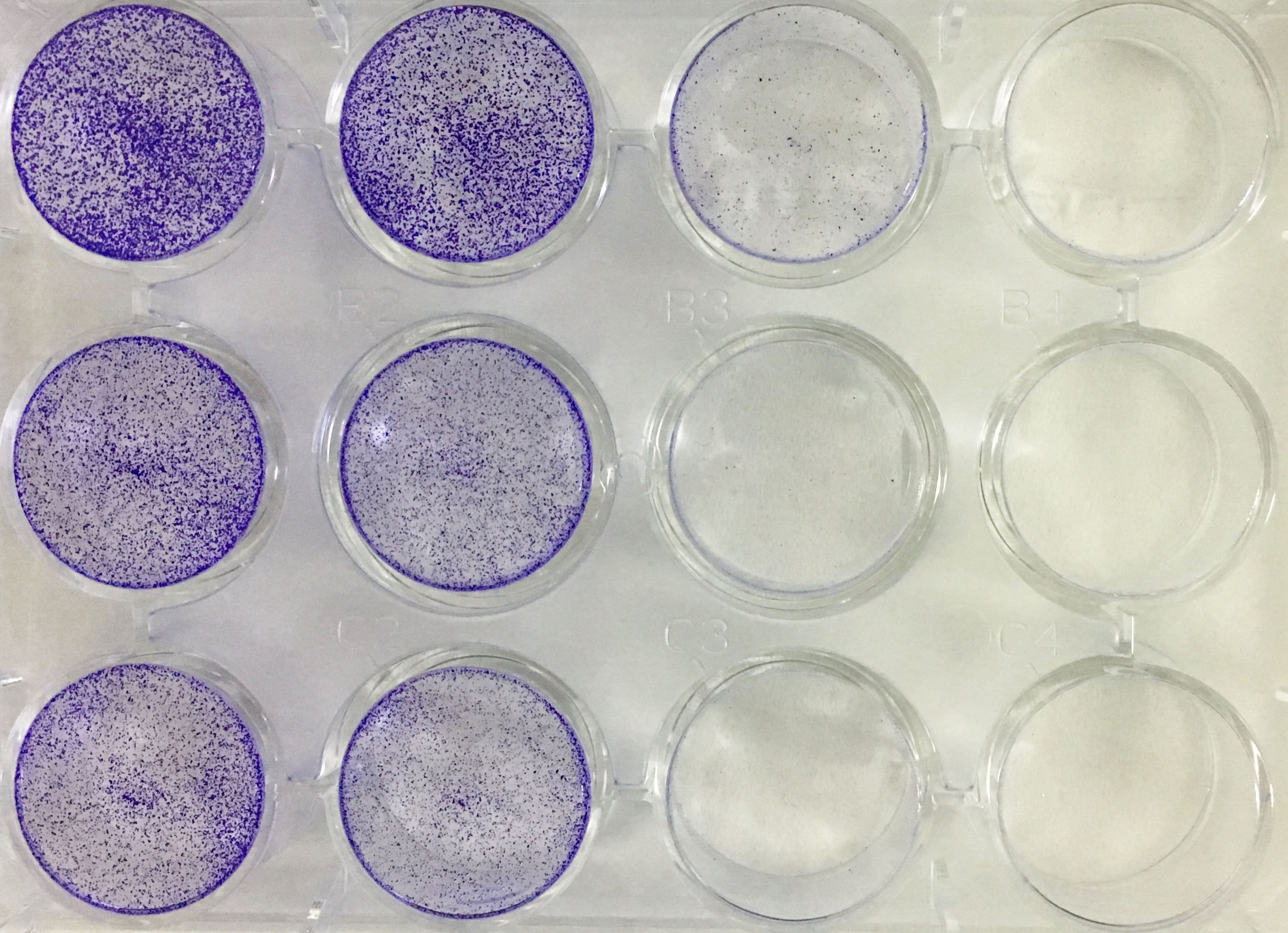

Supplement: Supplementary file 4 [file DataSheet9.ZIP › Figure6/Figure6C/SW620.jpg]

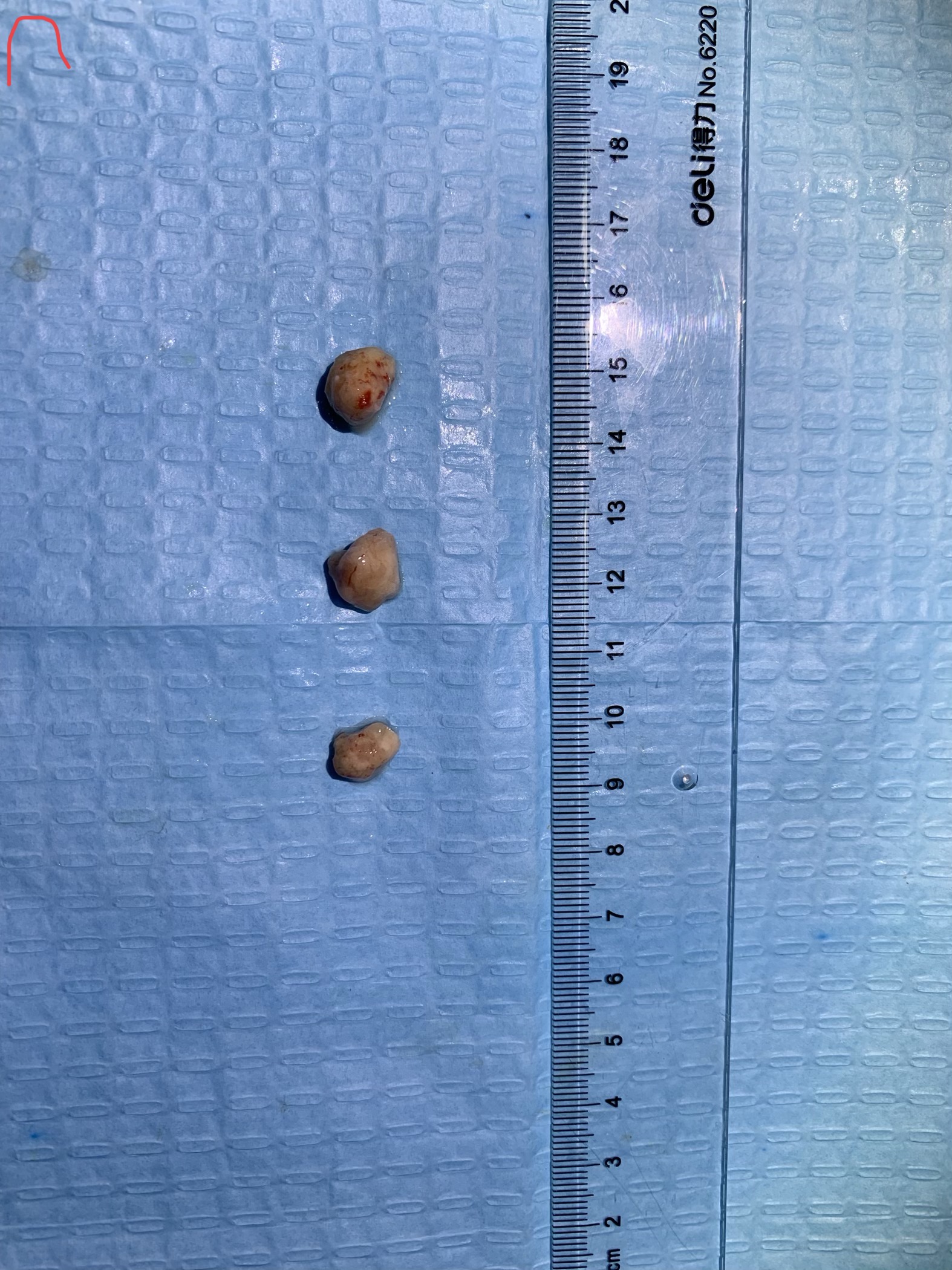

Supplement: Supplementary file 4 [file DataSheet9.ZIP › Figure6/Figure6E/Combination.jpg]

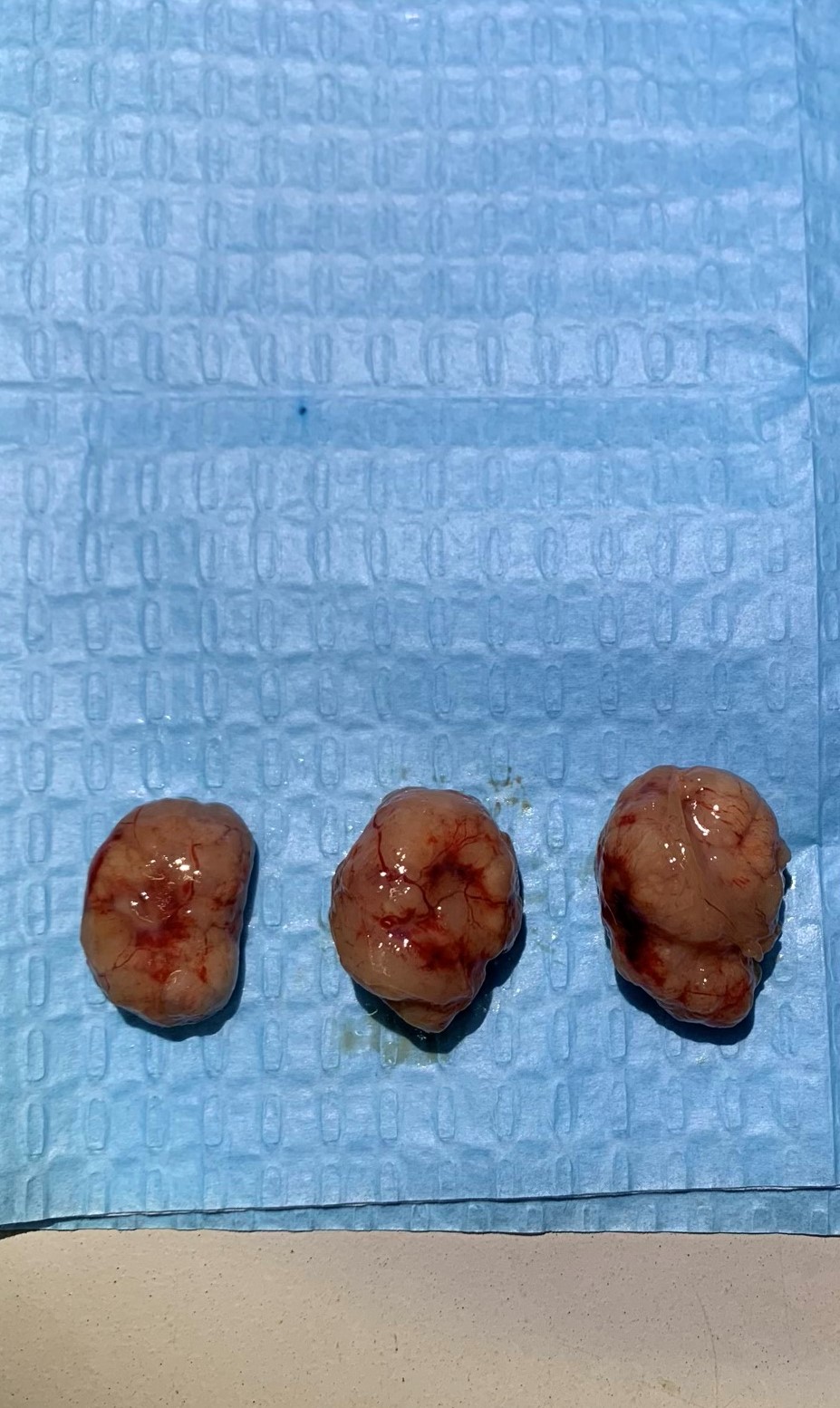

Supplement: Supplementary file 4 [file DataSheet9.ZIP › Figure6/Figure6E/NC.jpg]

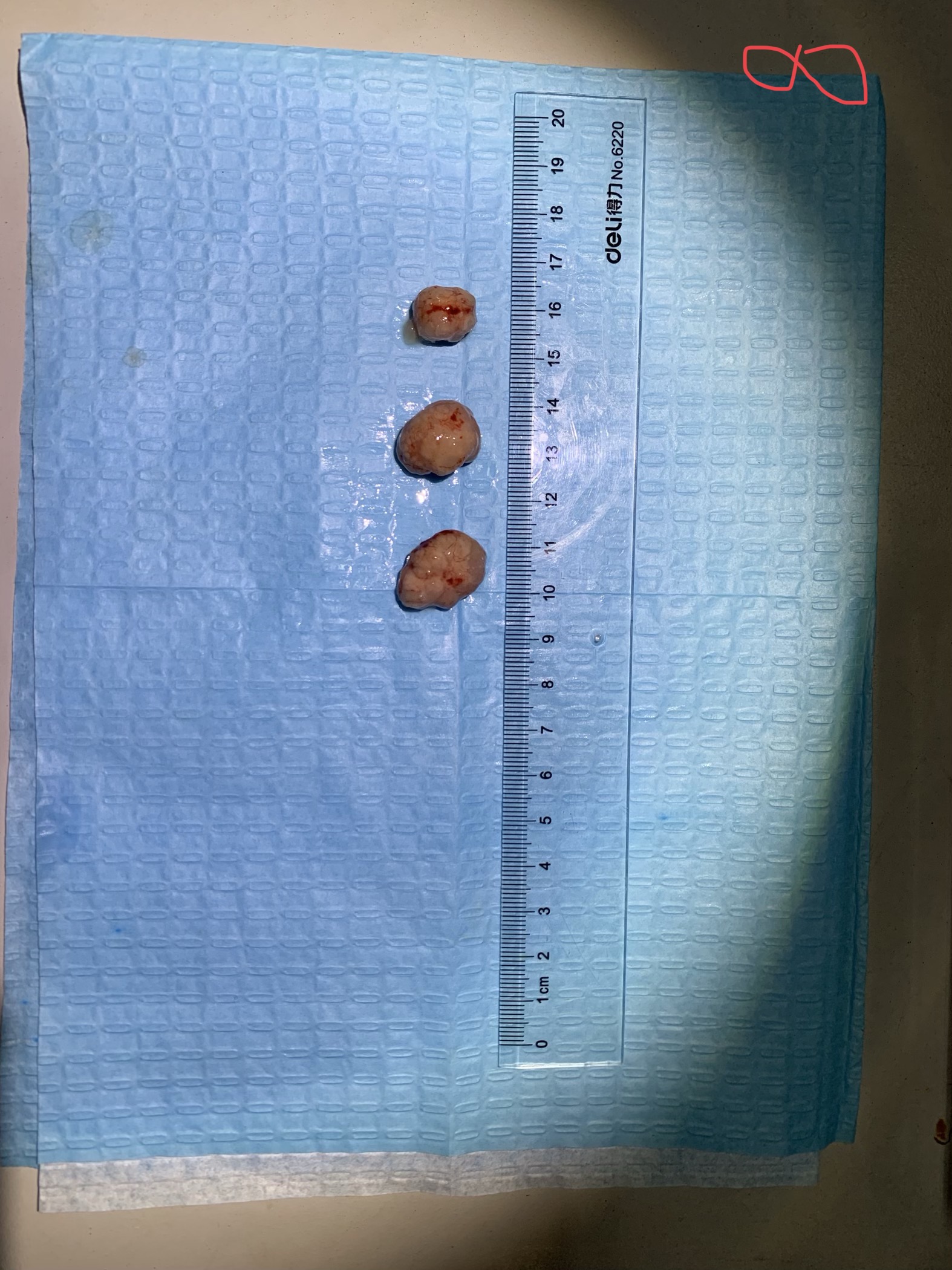

Supplement: Supplementary file 4 [file DataSheet9.ZIP › Figure6/Figure6E/PND-1186.jpg]

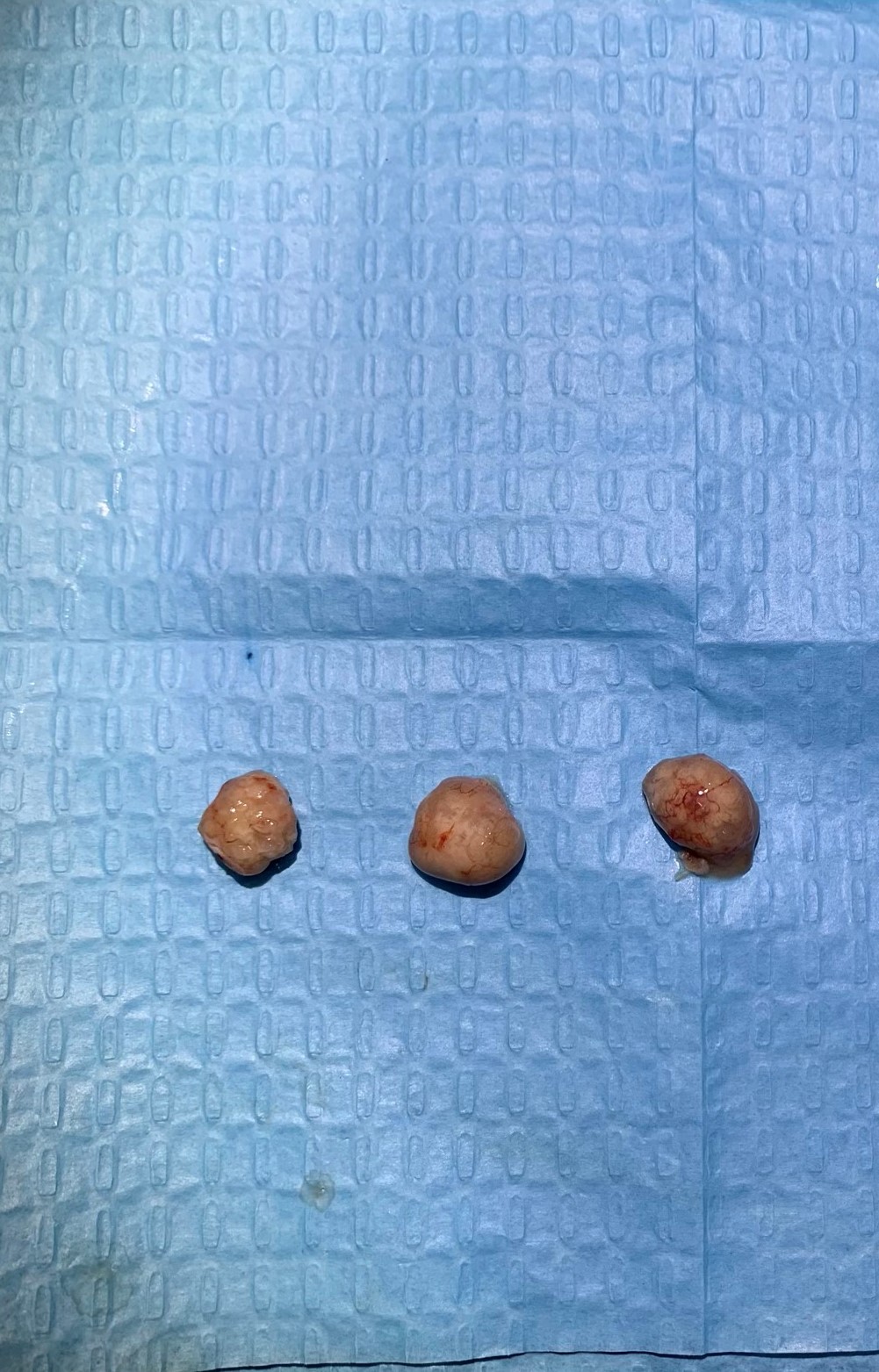

Supplement: Supplementary file 4 [file DataSheet9.ZIP › Figure6/Figure6E/SHP099.jpg]

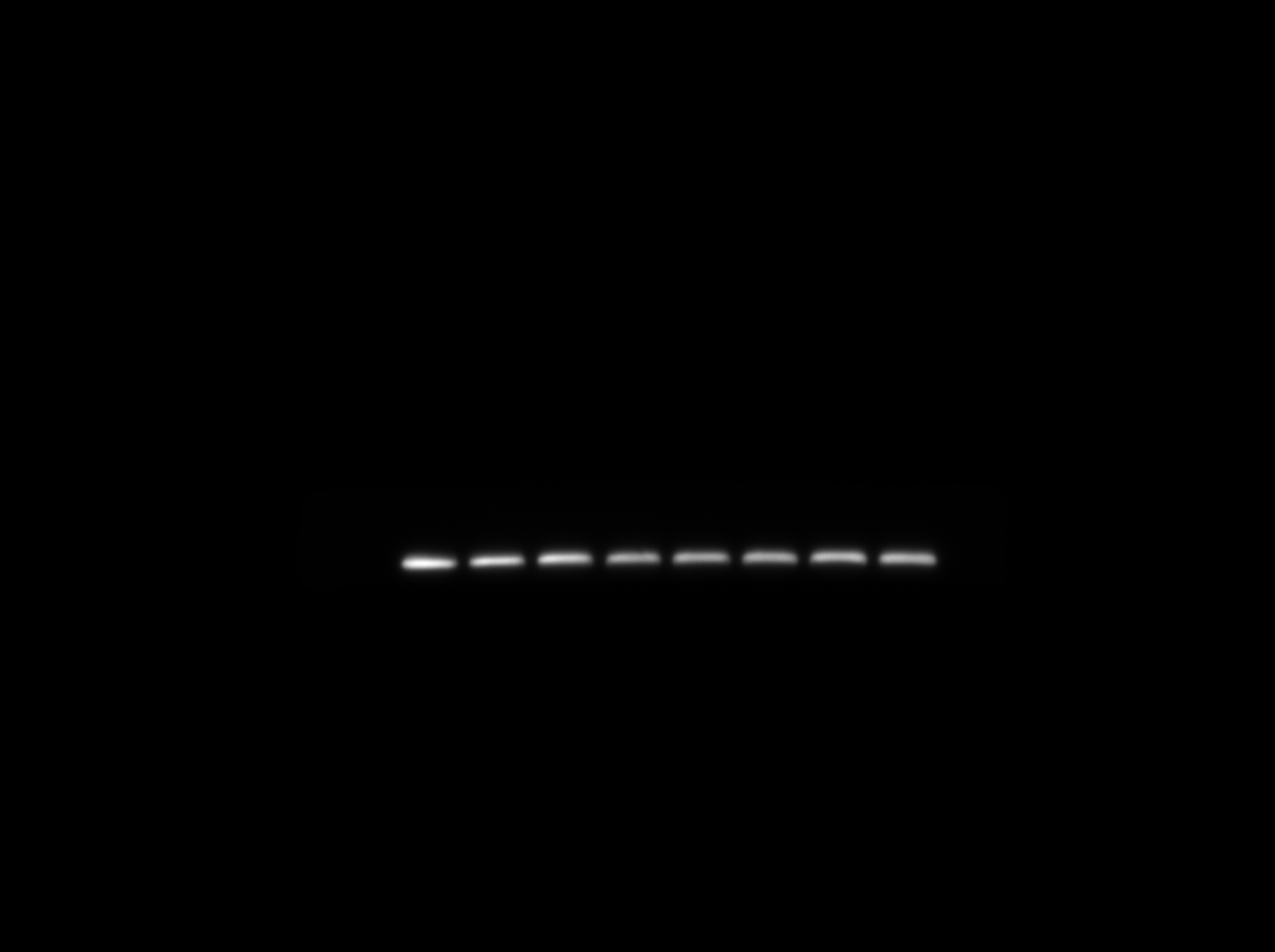

Supplement: Supplementary file 7 [file DataSheet1.zip › FigureS4/FigureS4A/GAPDH CW-2.tif]

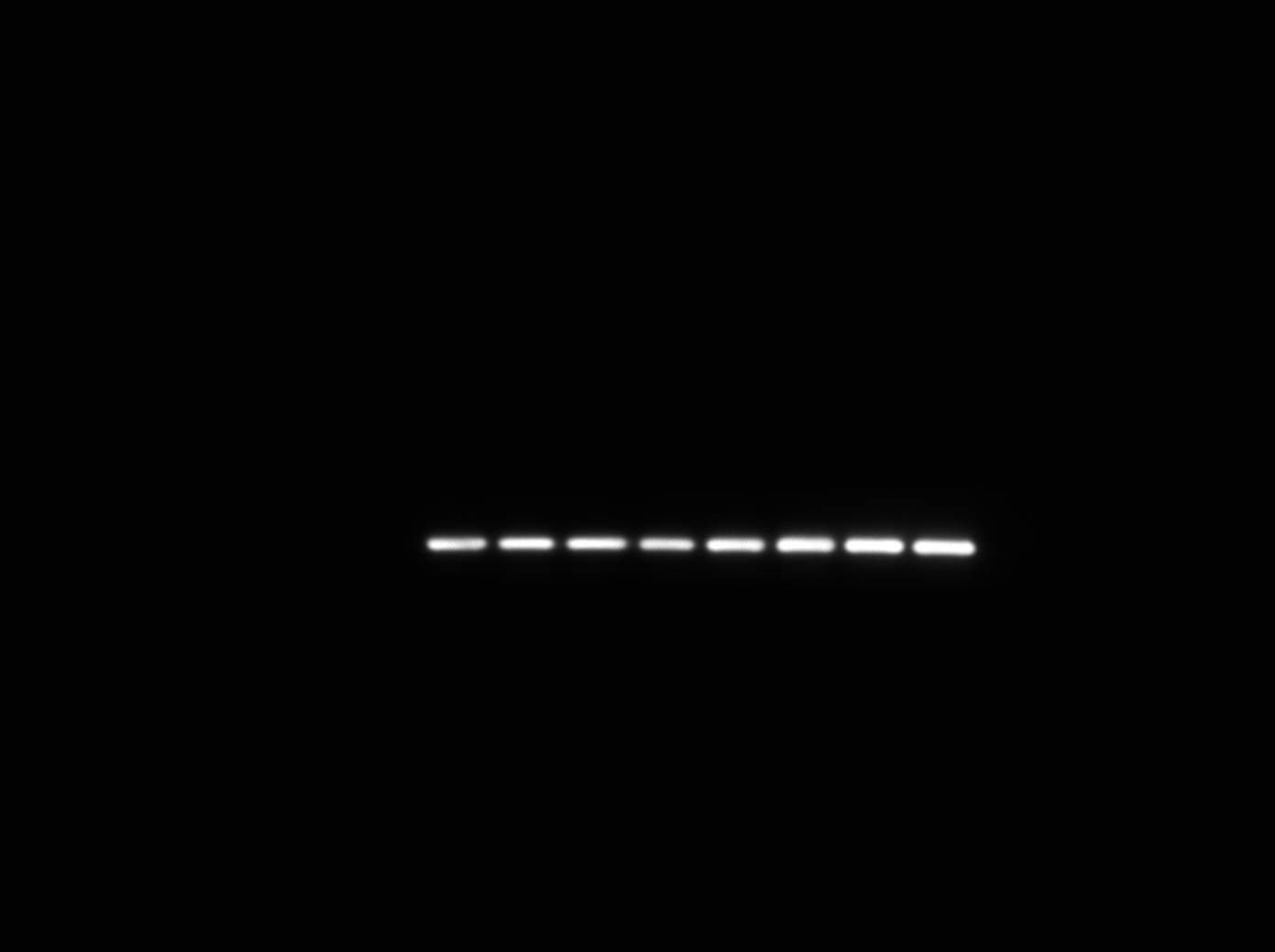

Supplement: Supplementary file 7 [file DataSheet1.zip › FigureS4/FigureS4A/GAPDH Caco-2.tif]

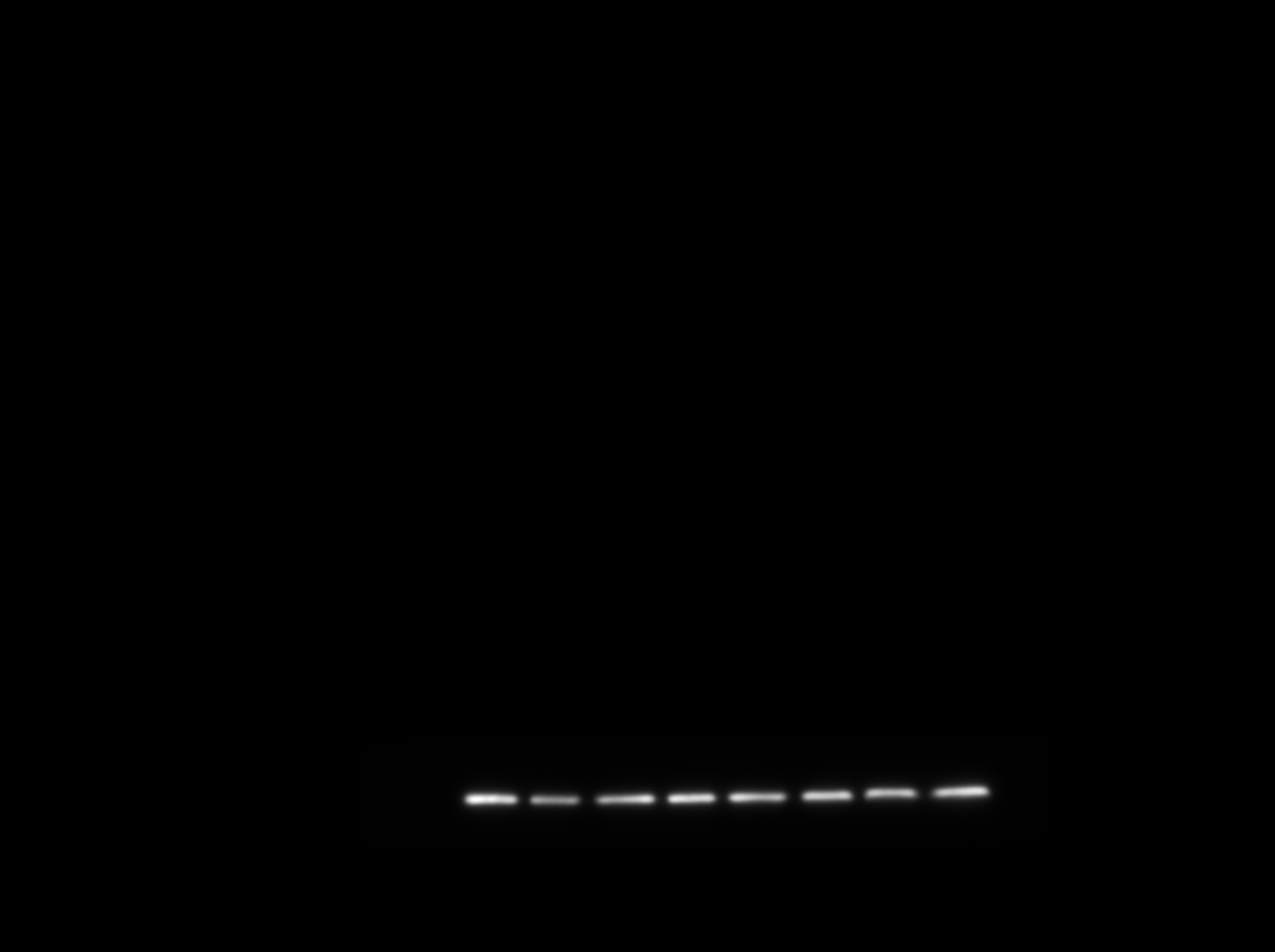

Supplement: Supplementary file 7 [file DataSheet1.zip › FigureS4/FigureS4A/GAPDH RKO.tif]

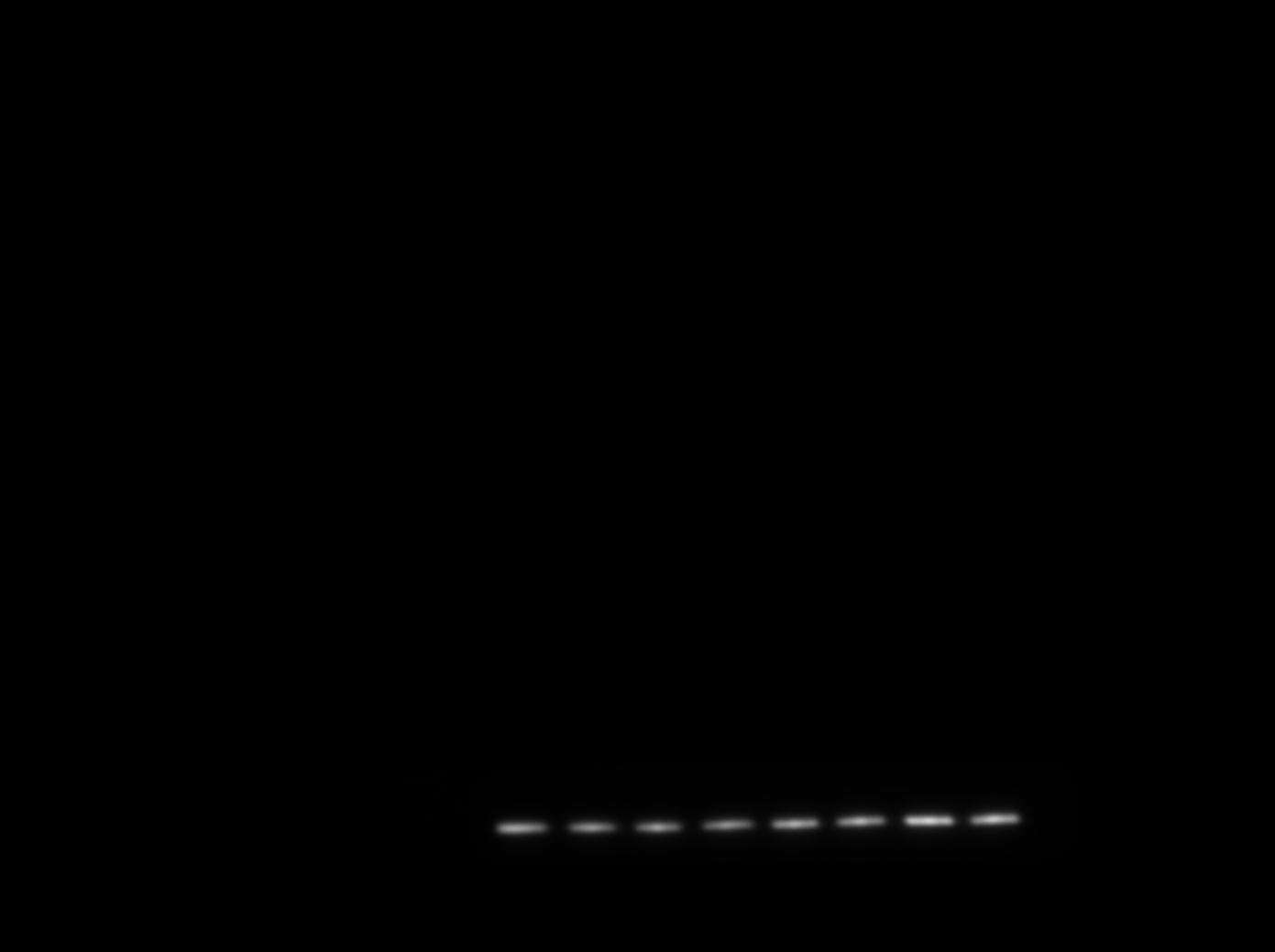

Supplement: Supplementary file 7 [file DataSheet1.zip › FigureS4/FigureS4A/GAPDH SW480.tif]

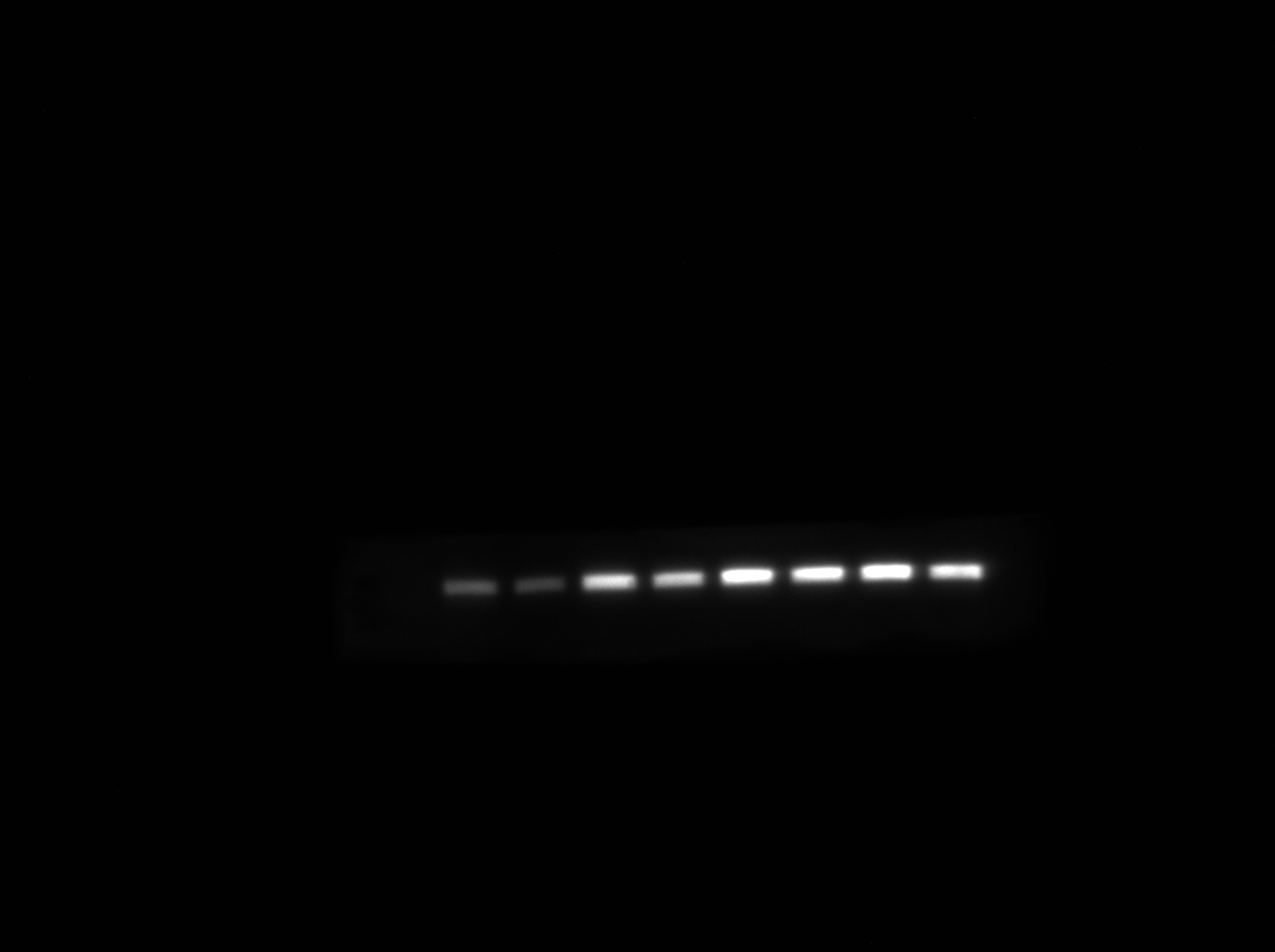

Supplement: Supplementary file 7 [file DataSheet1.zip › FigureS4/FigureS4A/P-AKT CW-2 12h+24h.tif]

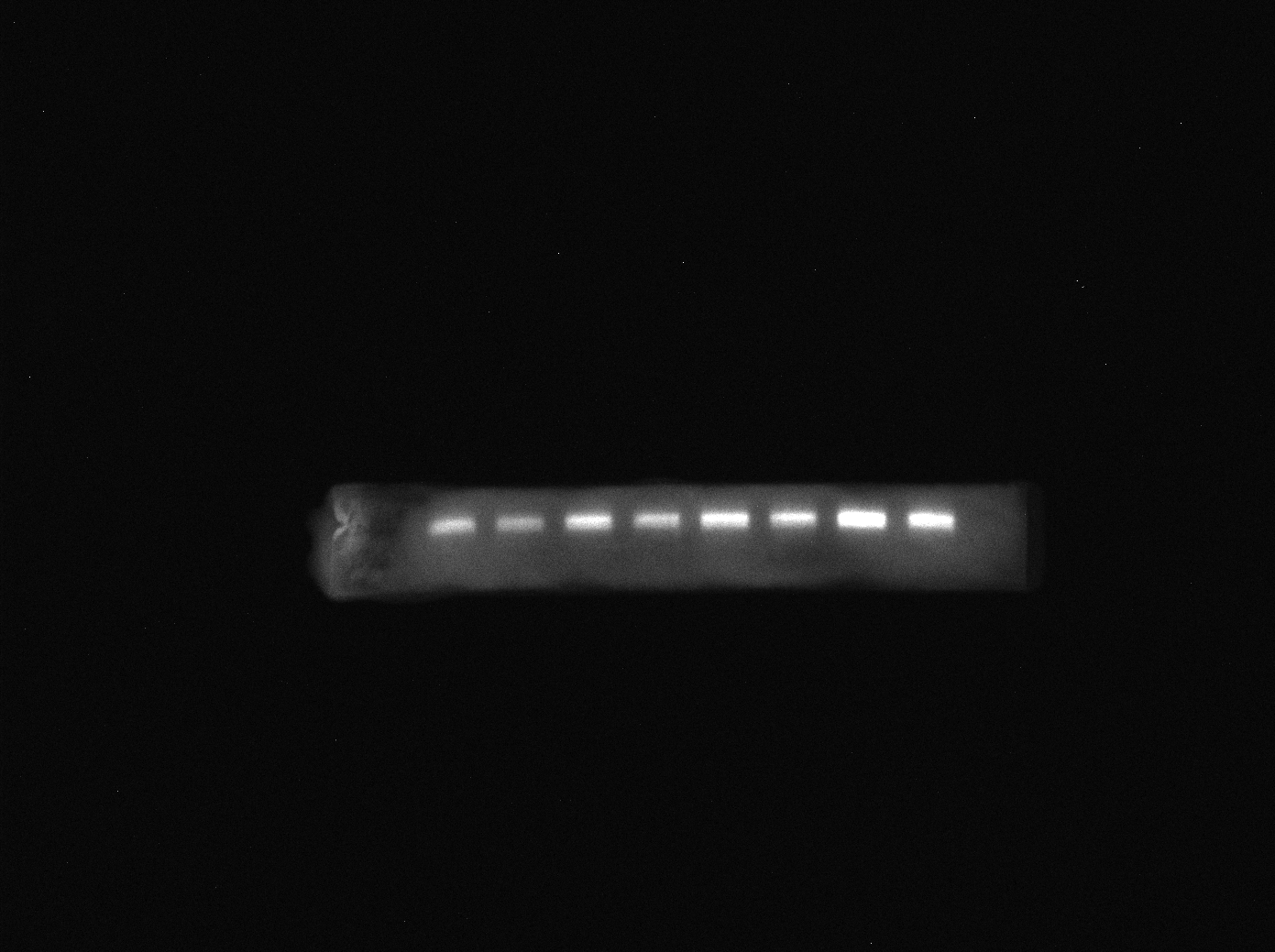

Supplement: Supplementary file 7 [file DataSheet1.zip › FigureS4/FigureS4A/P-AKT CW-2 30min+1h.tif]

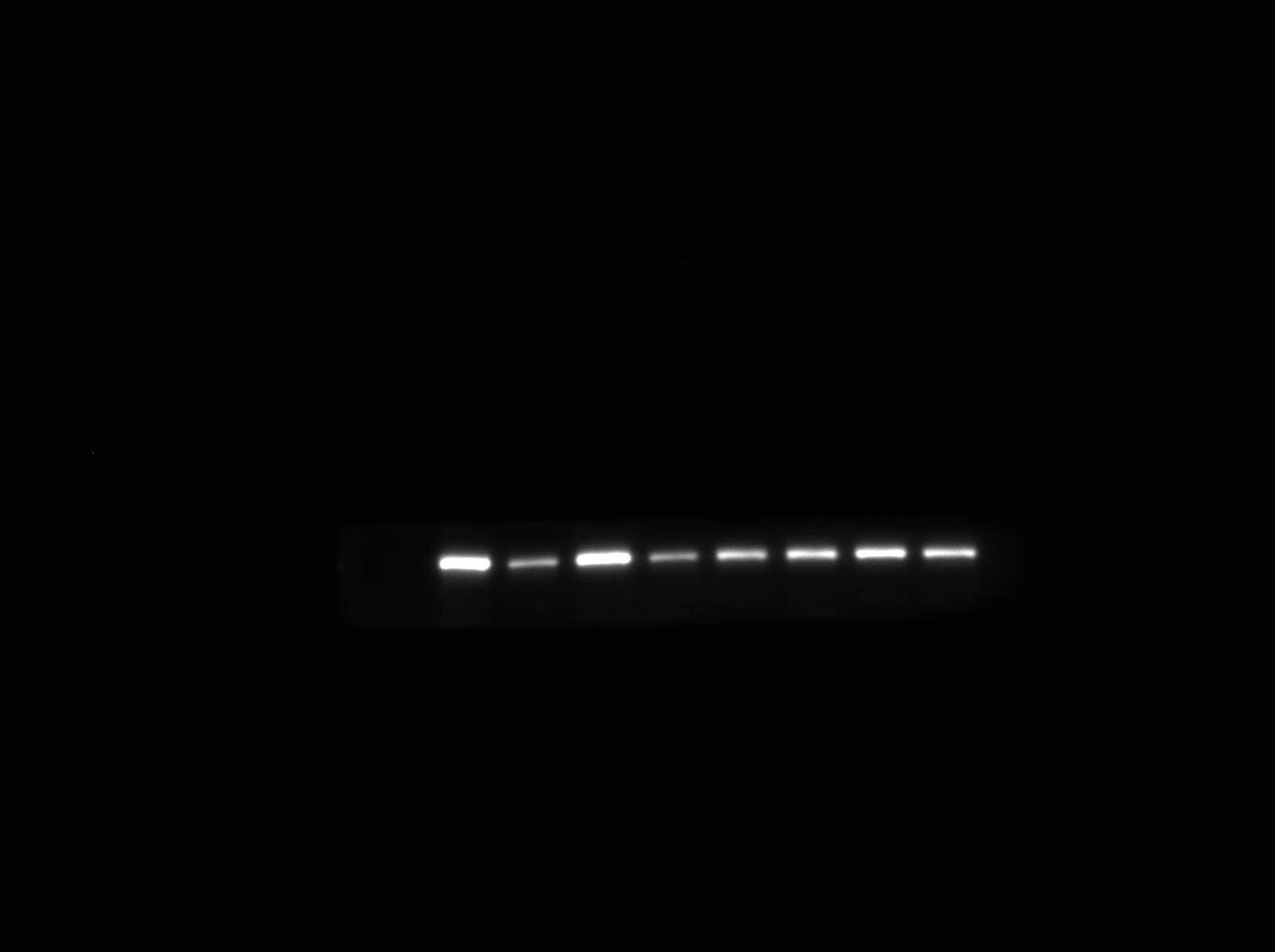

Supplement: Supplementary file 7 [file DataSheet1.zip › FigureS4/FigureS4A/P-AKT Caco-2.tif]

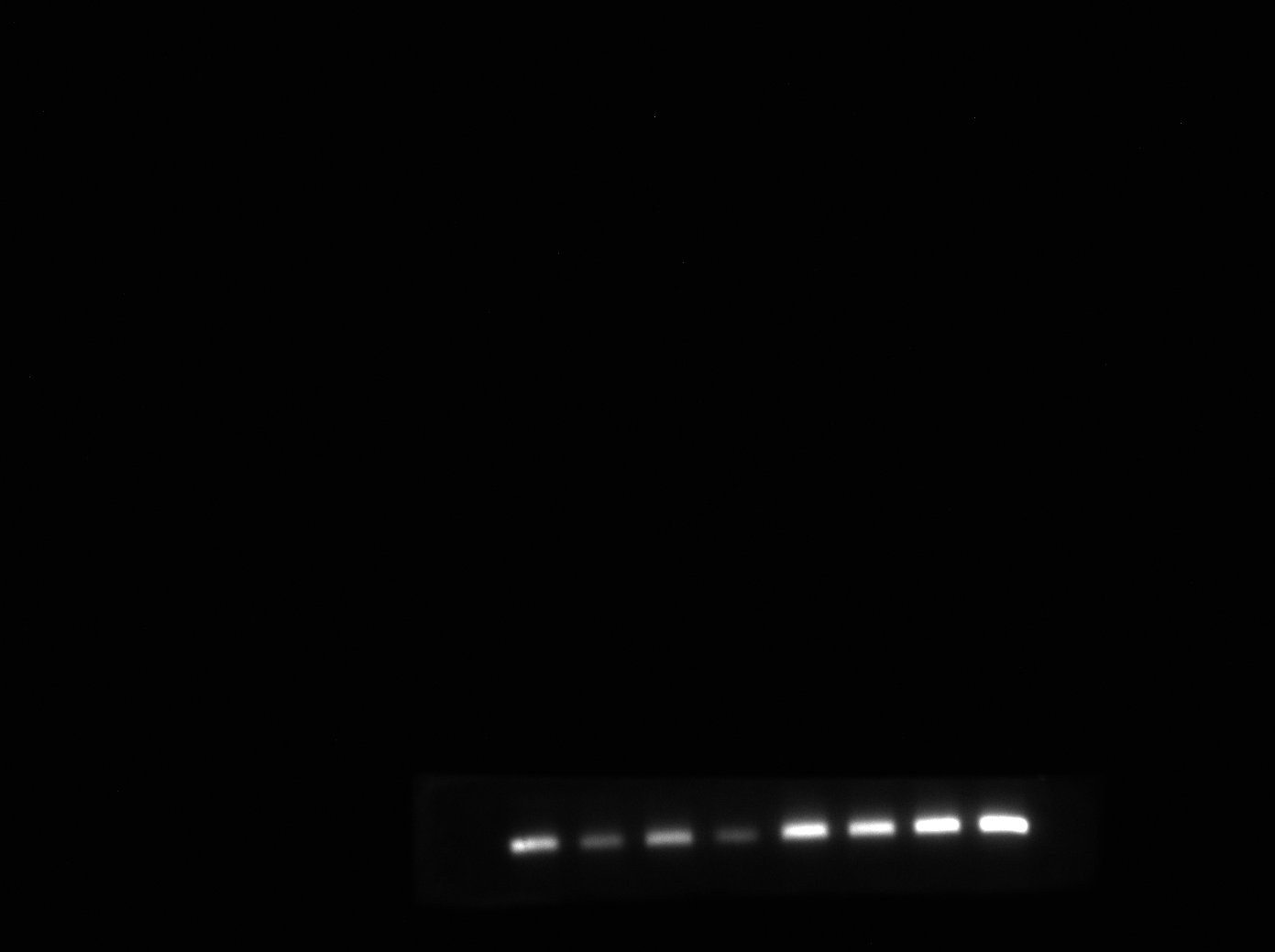

Supplement: Supplementary file 7 [file DataSheet1.zip › FigureS4/FigureS4A/P-AKT RKO.tif]

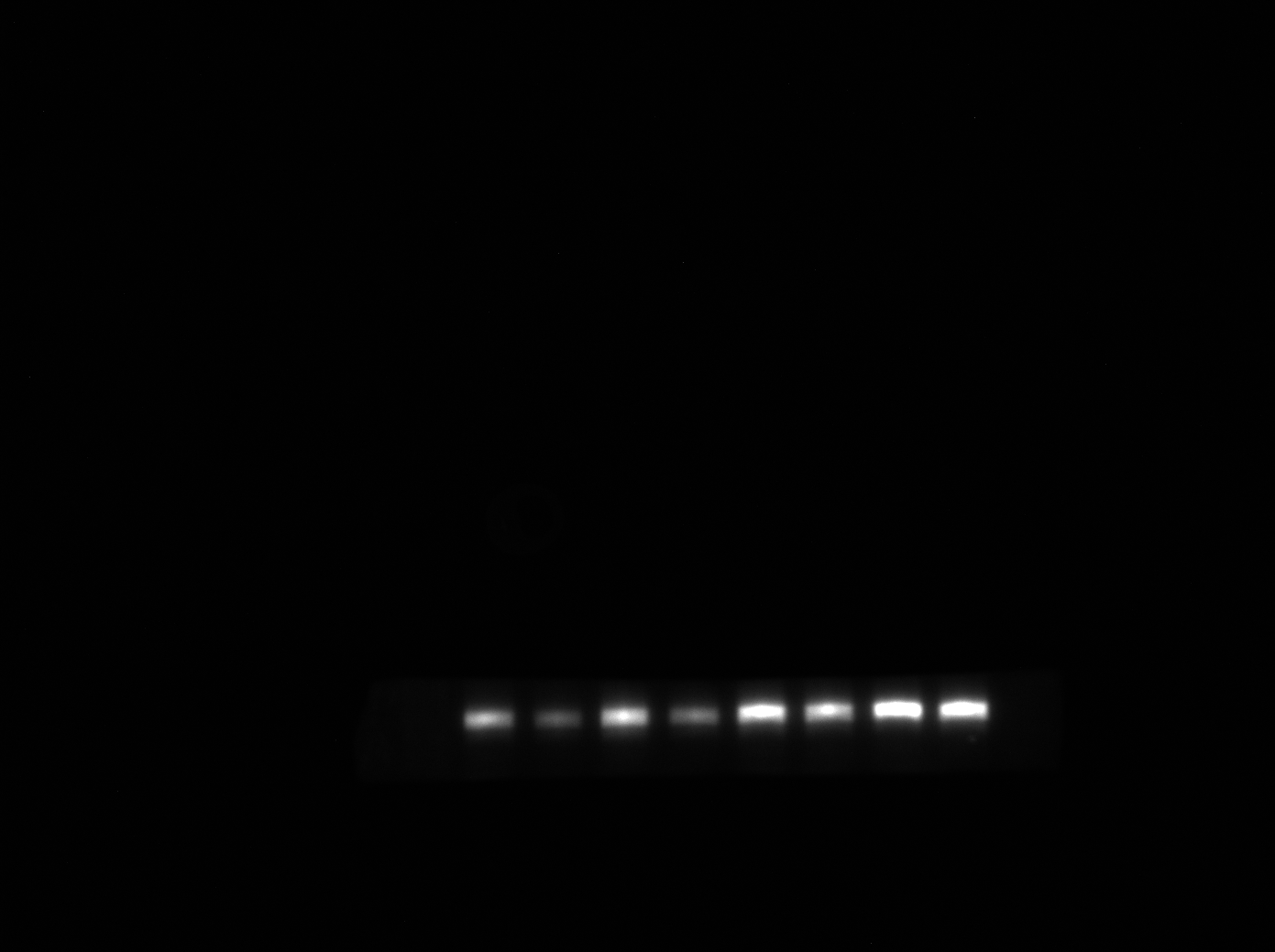

Supplement: Supplementary file 7 [file DataSheet1.zip › FigureS4/FigureS4A/P-AKT SW480.tif]

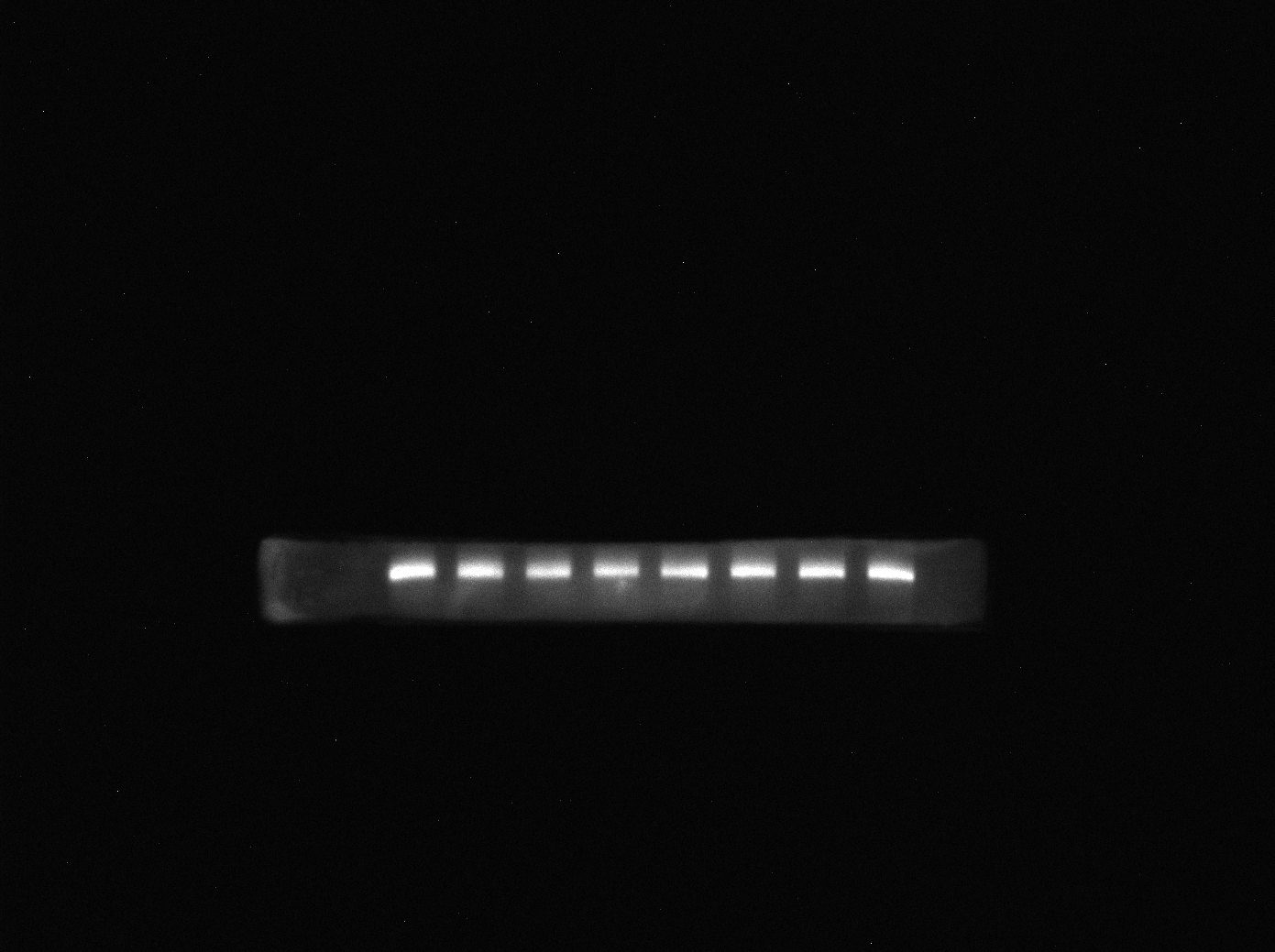

Supplement: Supplementary file 7 [file DataSheet1.zip › FigureS4/FigureS4A/P-FAK CW-2.tif]

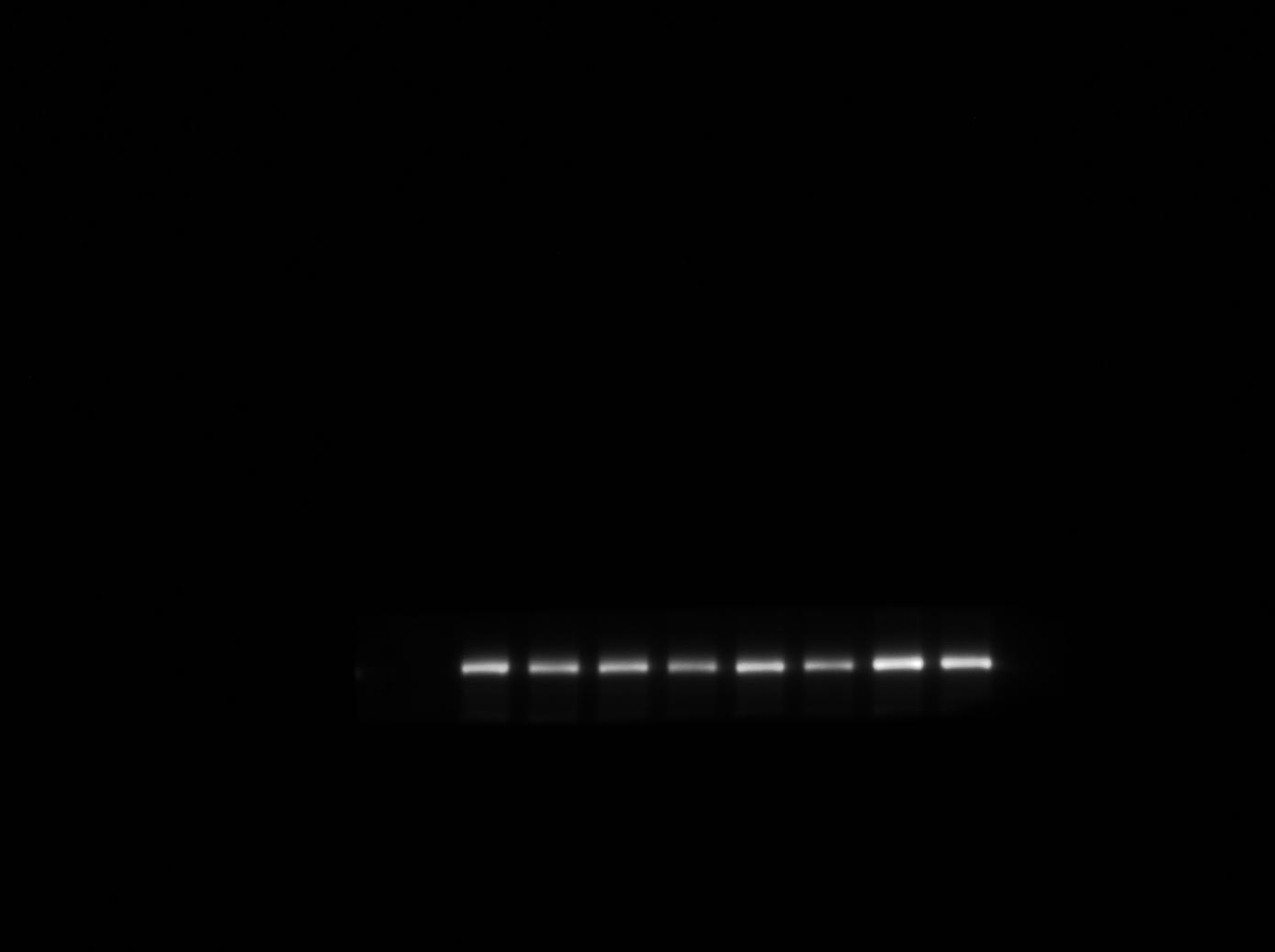

Supplement: Supplementary file 7 [file DataSheet1.zip › FigureS4/FigureS4A/P-FAK Caco-2.tif]

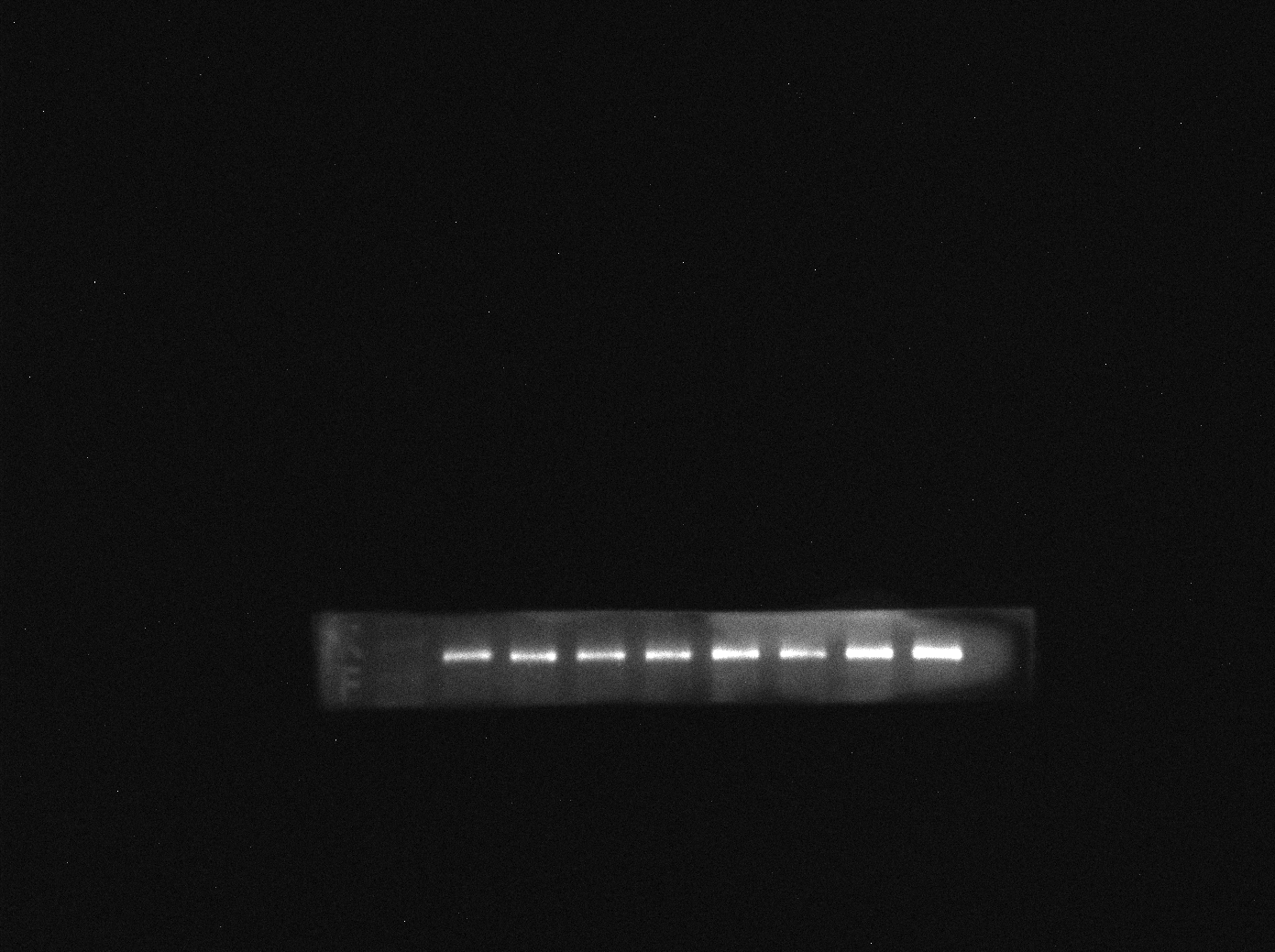

Supplement: Supplementary file 7 [file DataSheet1.zip › FigureS4/FigureS4A/P-FAK RKO.tif]

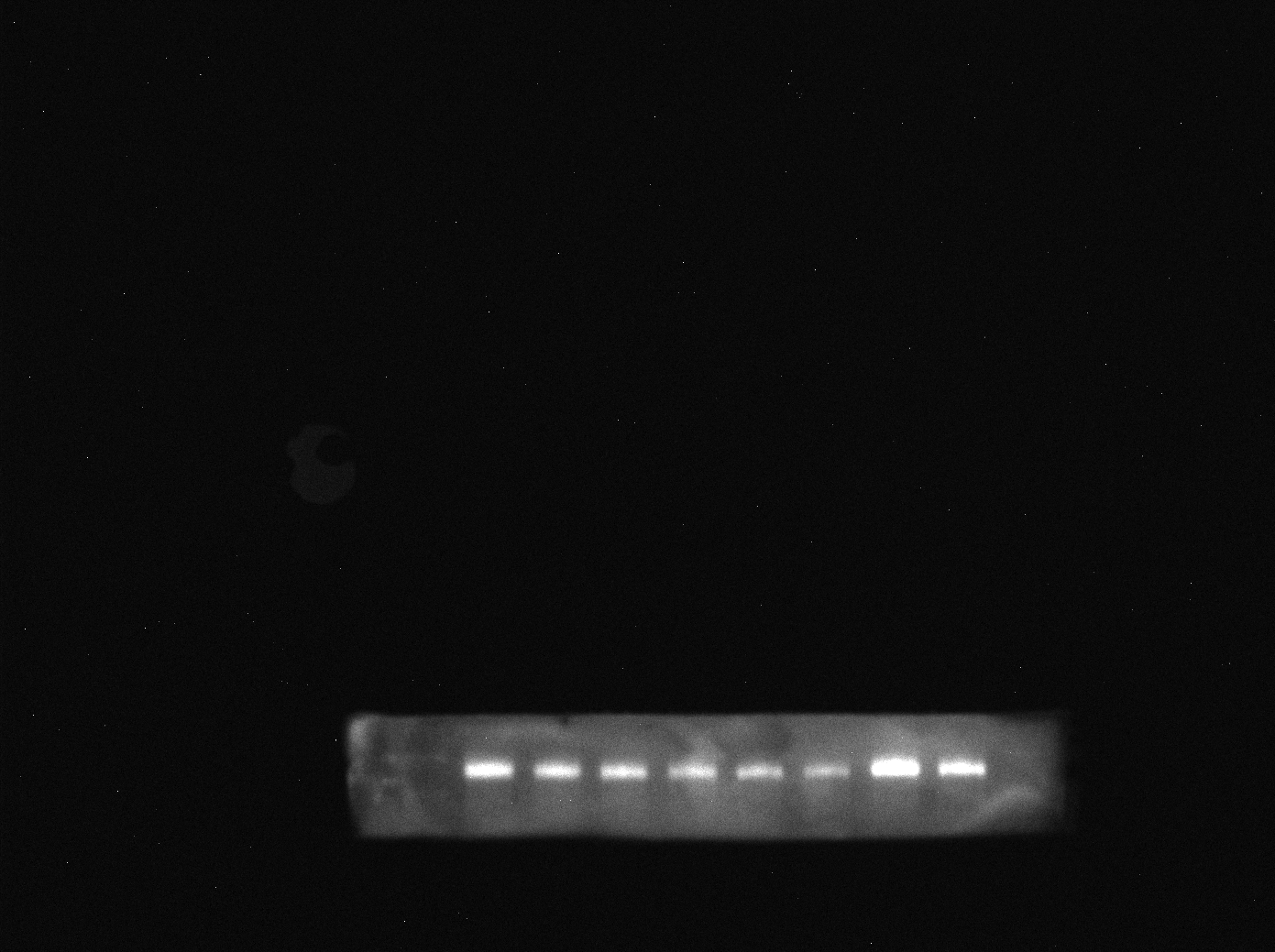

Supplement: Supplementary file 7 [file DataSheet1.zip › FigureS4/FigureS4A/P-FAK SW480.tif]

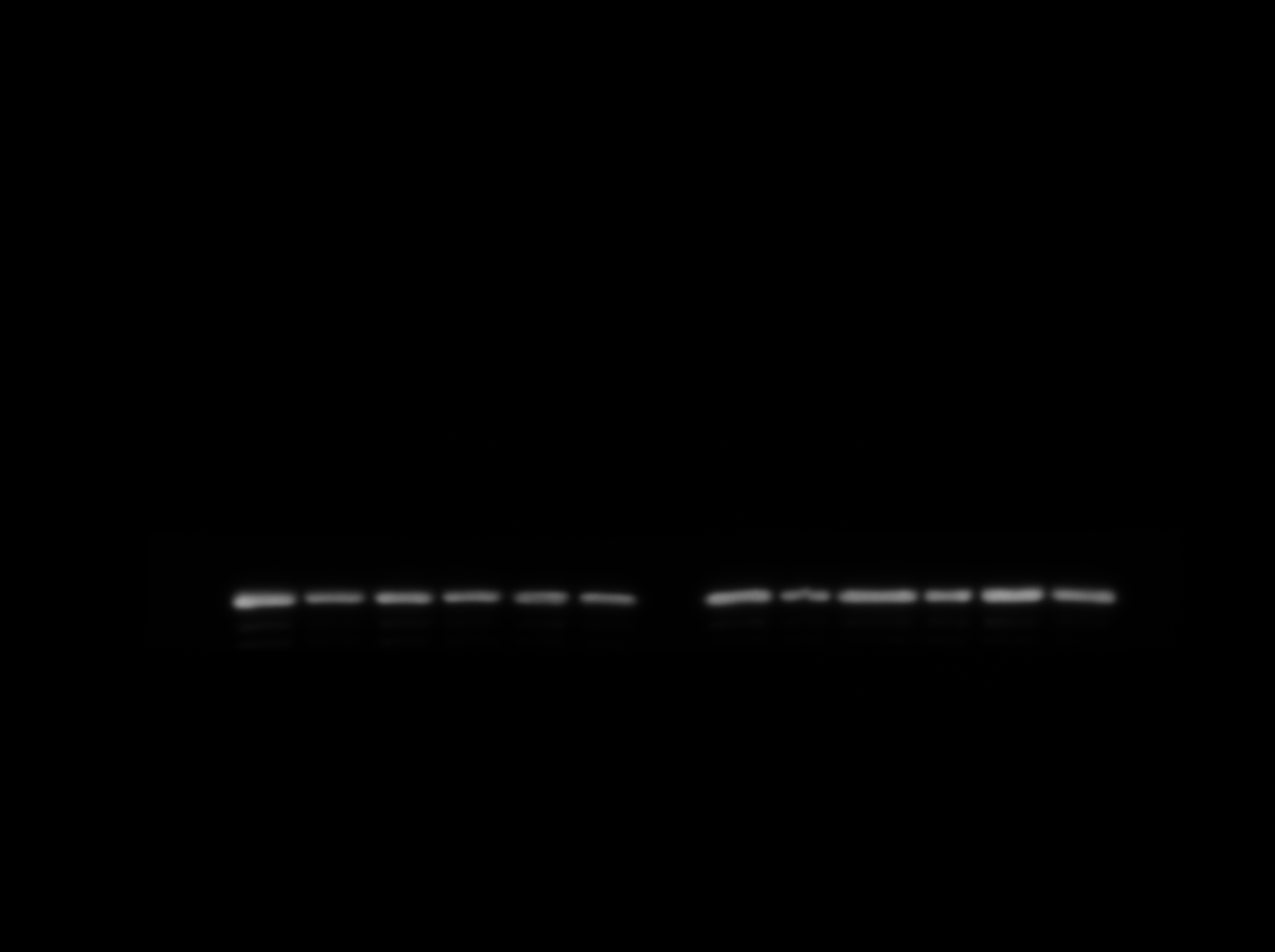

Supplement: Supplementary file 7 [file DataSheet1.zip › FigureS4/FigureS4B/GAPDH.tif]

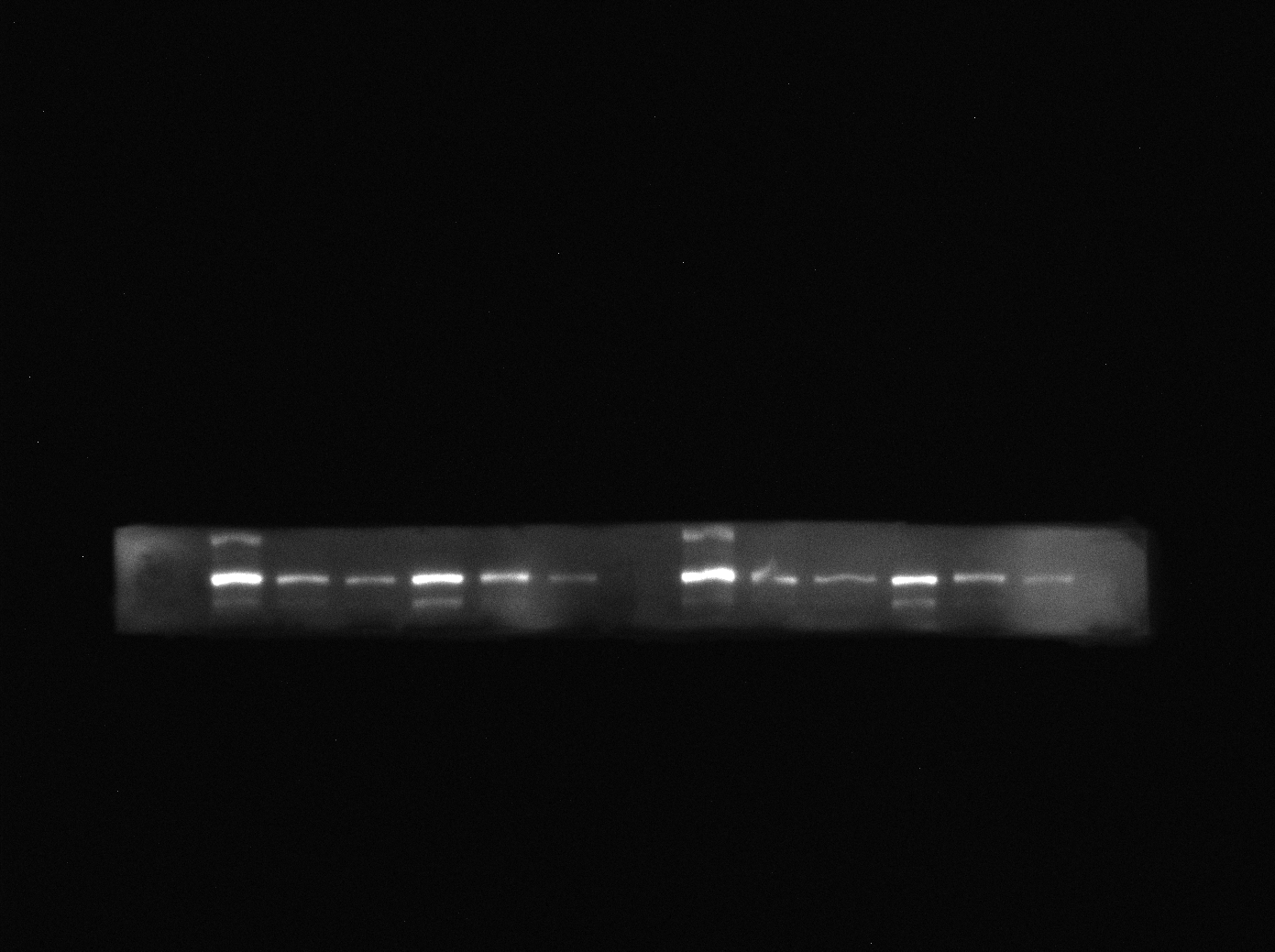

Supplement: Supplementary file 7 [file DataSheet1.zip › FigureS4/FigureS4B/P-FAK.tif]

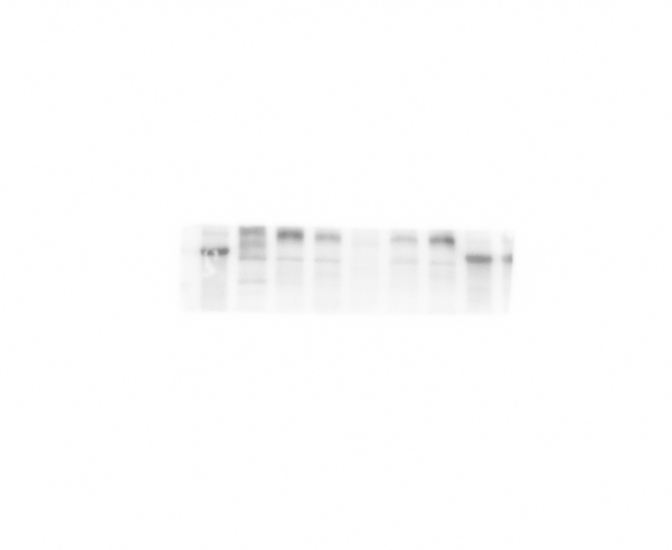

Supplement: Supplementary file 7 [file DataSheet1.zip › FigureS4/FigureS4B/P-SHP2.tif]

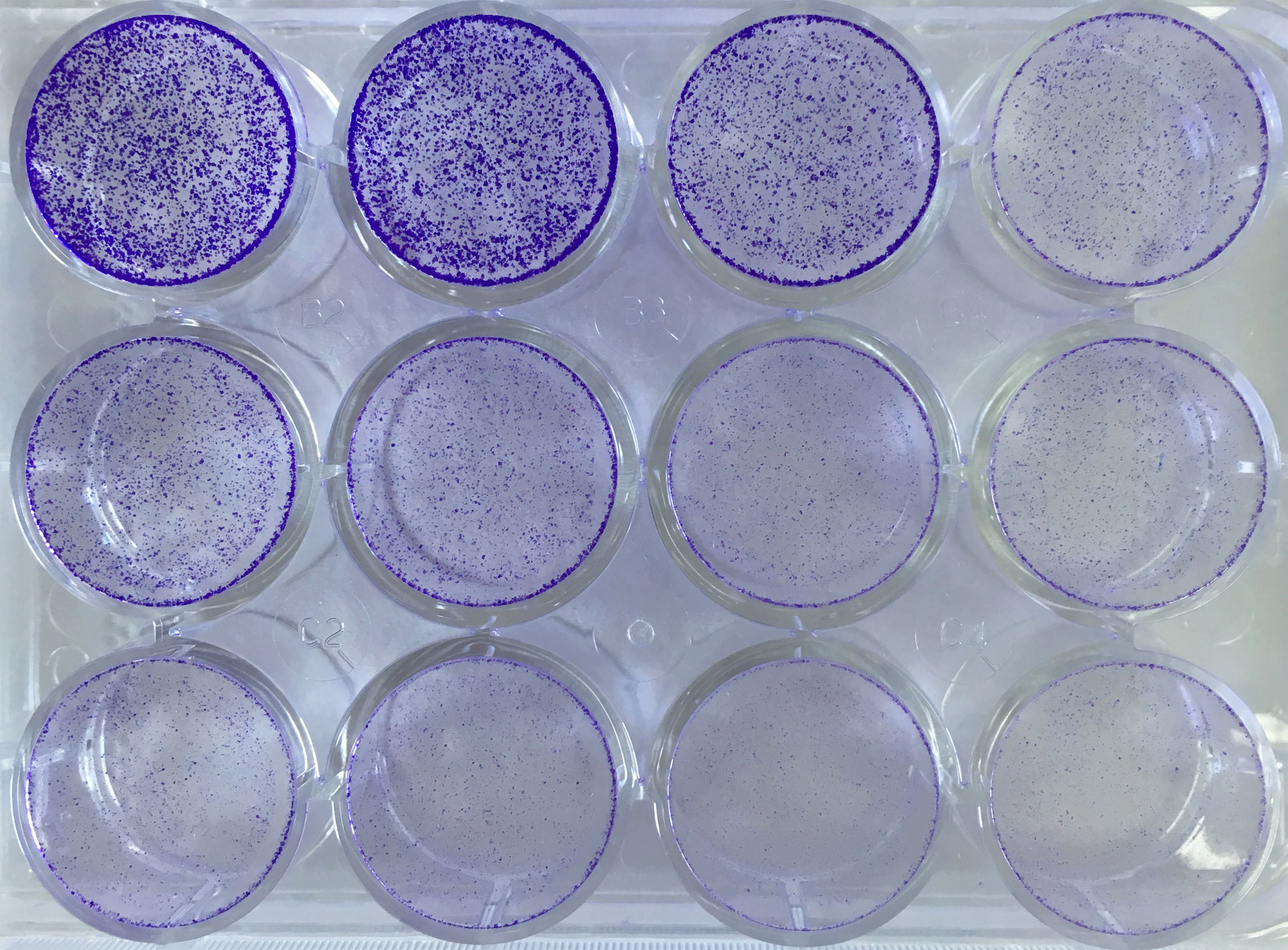

Supplement: Supplementary file 7 [file DataSheet1.zip › FigureS4/FigureS4C/CW-2.jpg]

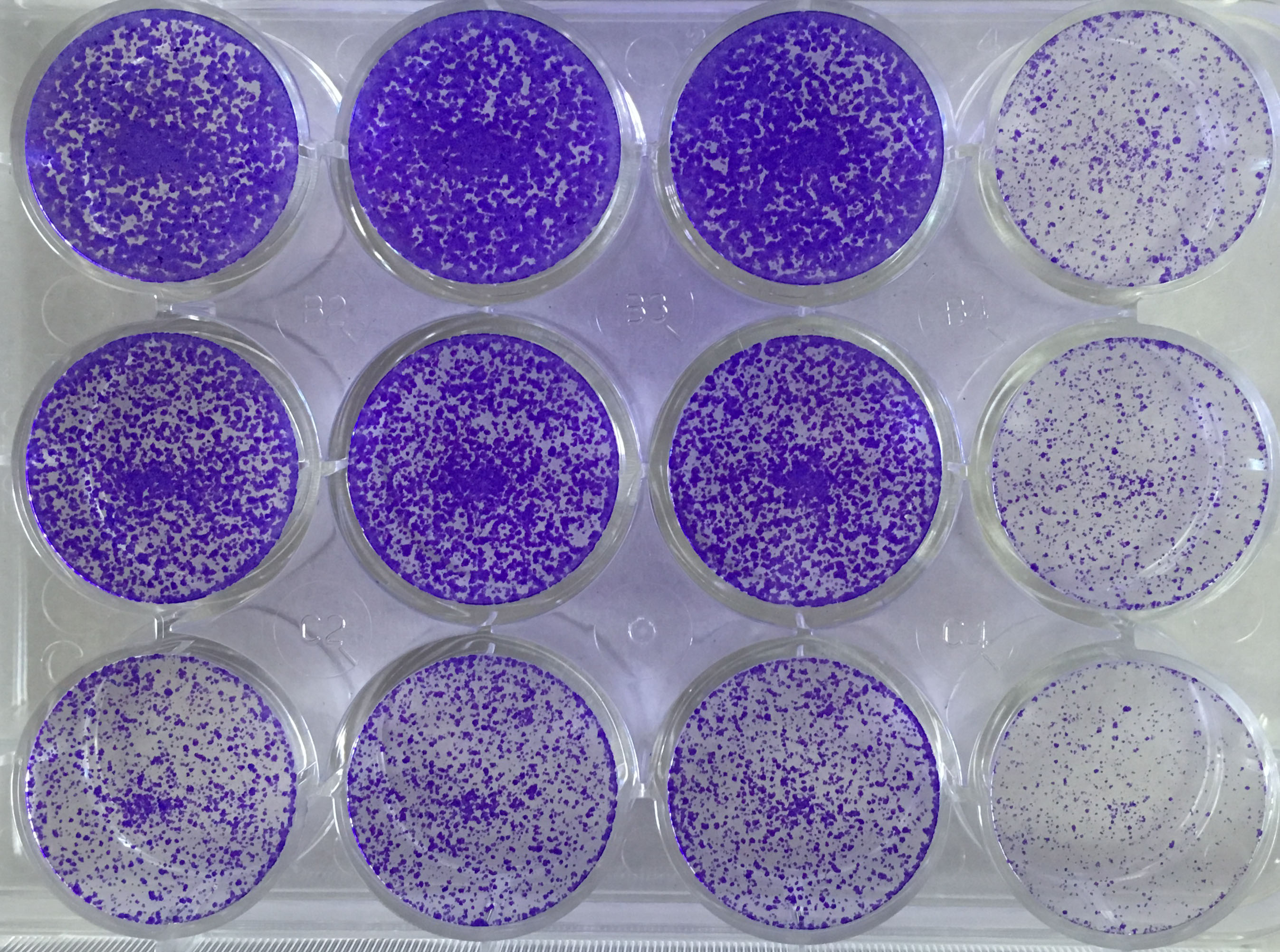

Supplement: Supplementary file 7 [file DataSheet1.zip › FigureS4/FigureS4C/Caco-2.jpg]

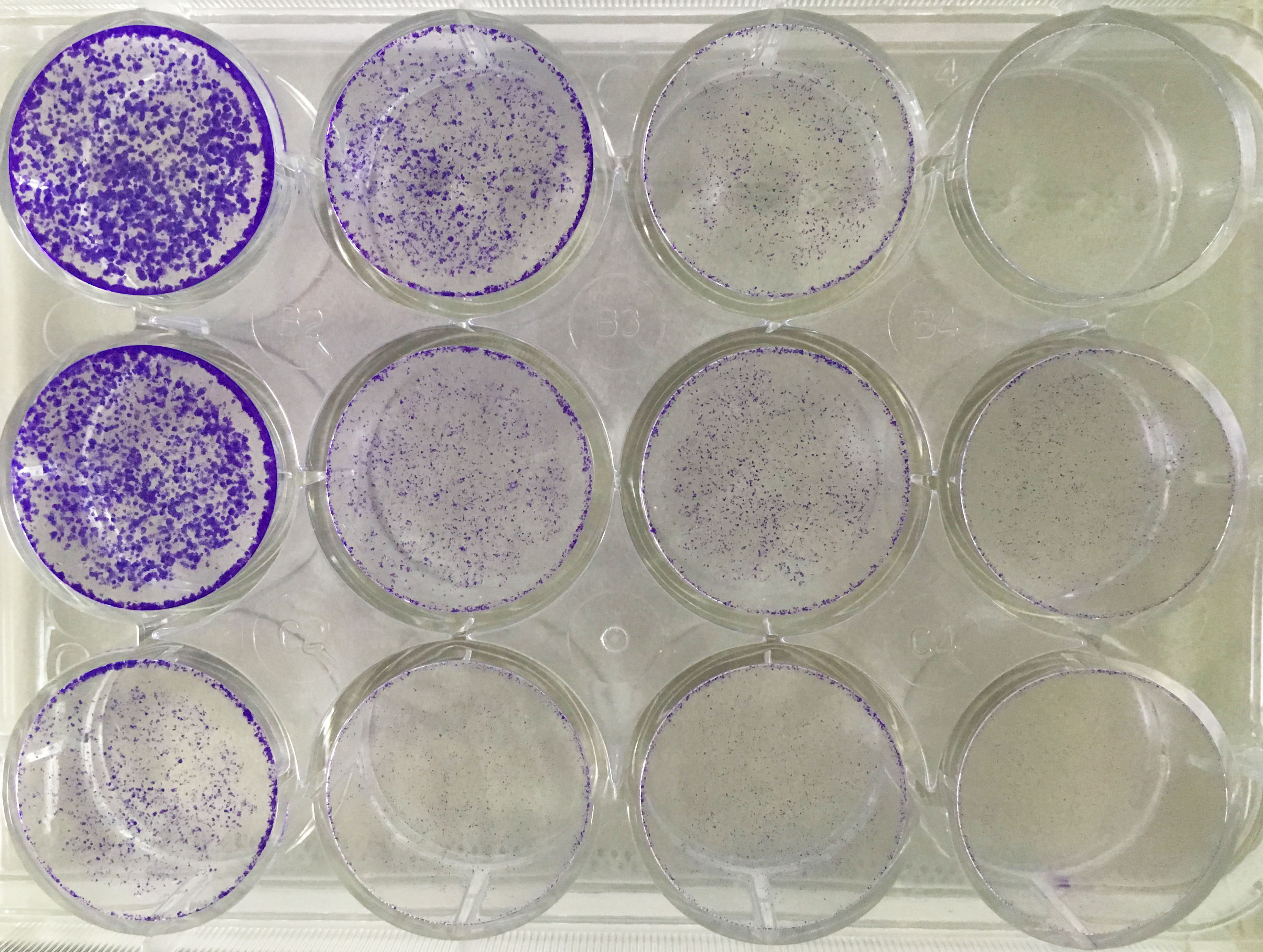

Supplement: Supplementary file 7 [file DataSheet1.zip › FigureS4/FigureS4C/RKO.jpg]

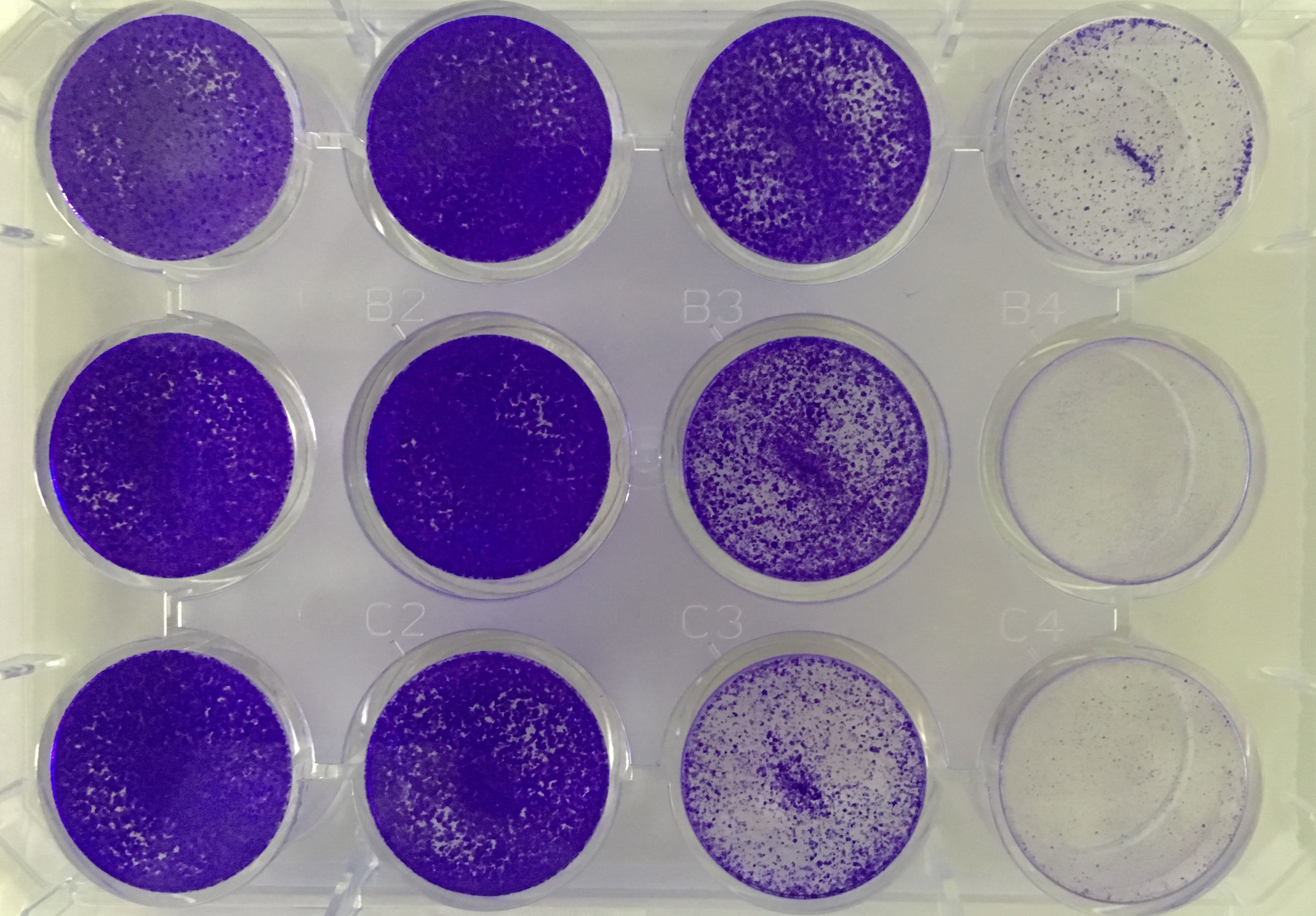

Supplement: Supplementary file 7 [file DataSheet1.zip › FigureS4/FigureS4C/SW480.jpg]

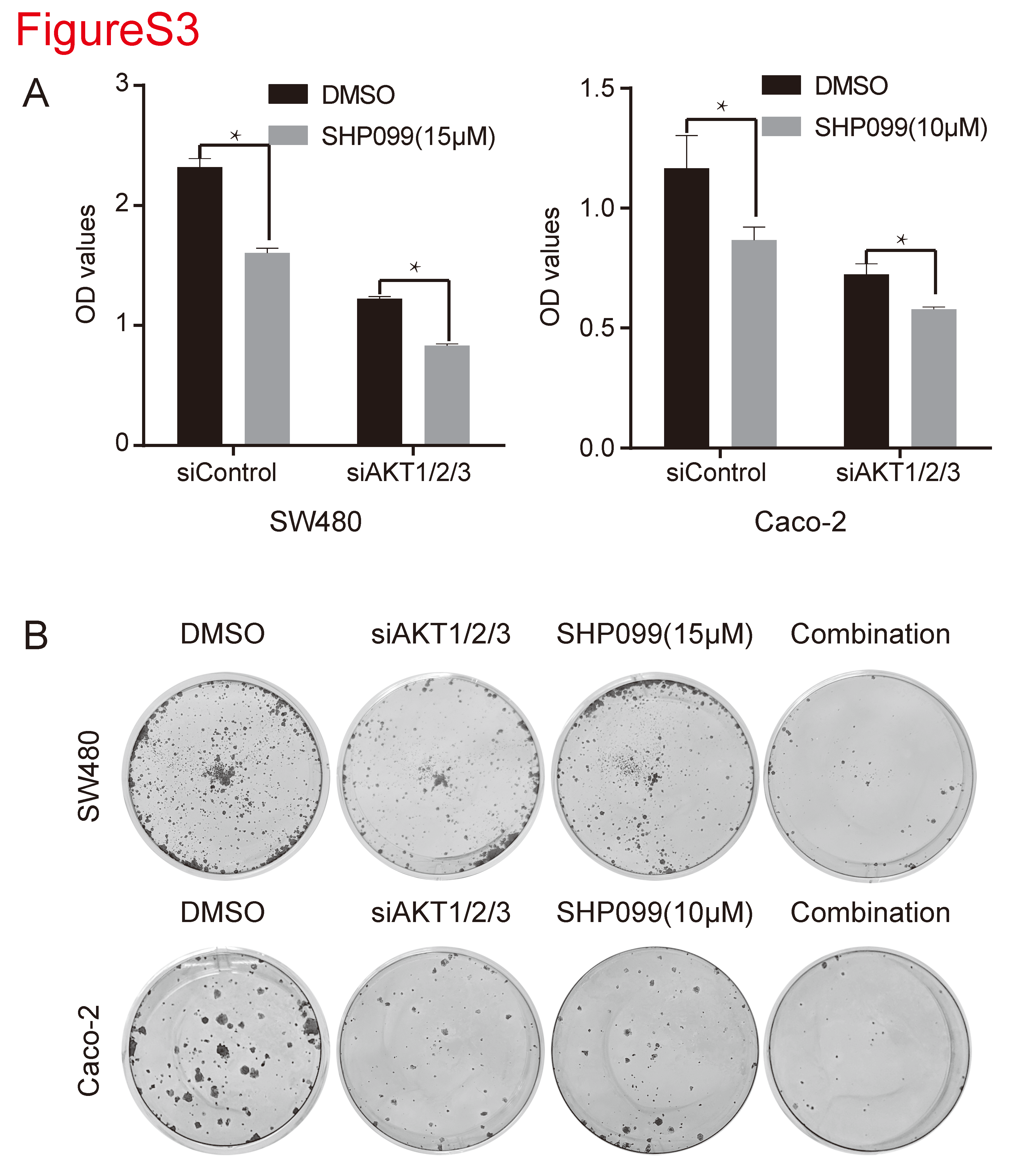

Supplement: Supplementary file 8 [file Image2.TIF]

FigureS4

A

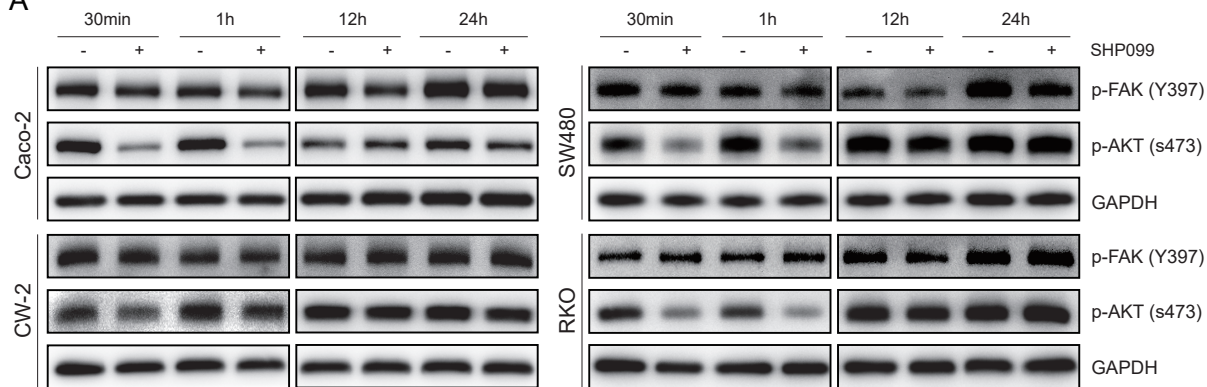

B

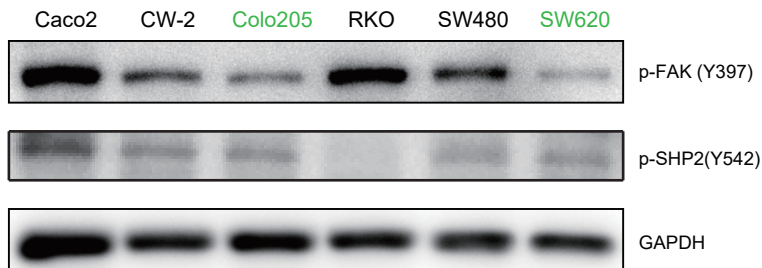

C

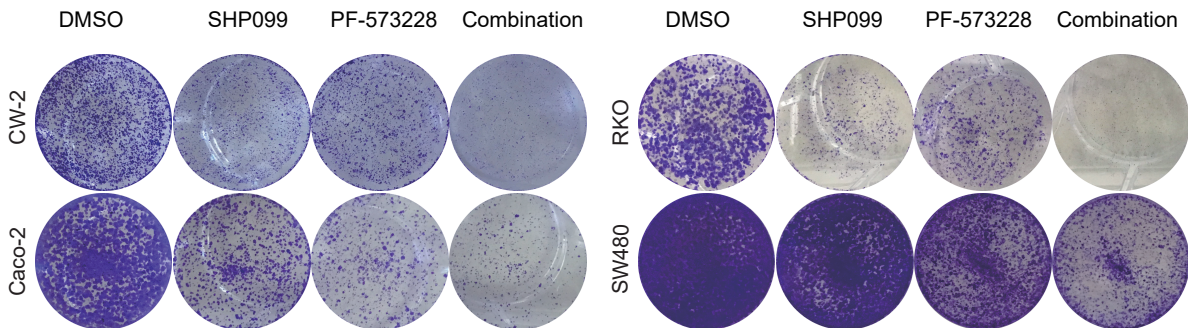

Supplement: Supplementary file 9 [file DataSheet3.PDF]

## Slide 1
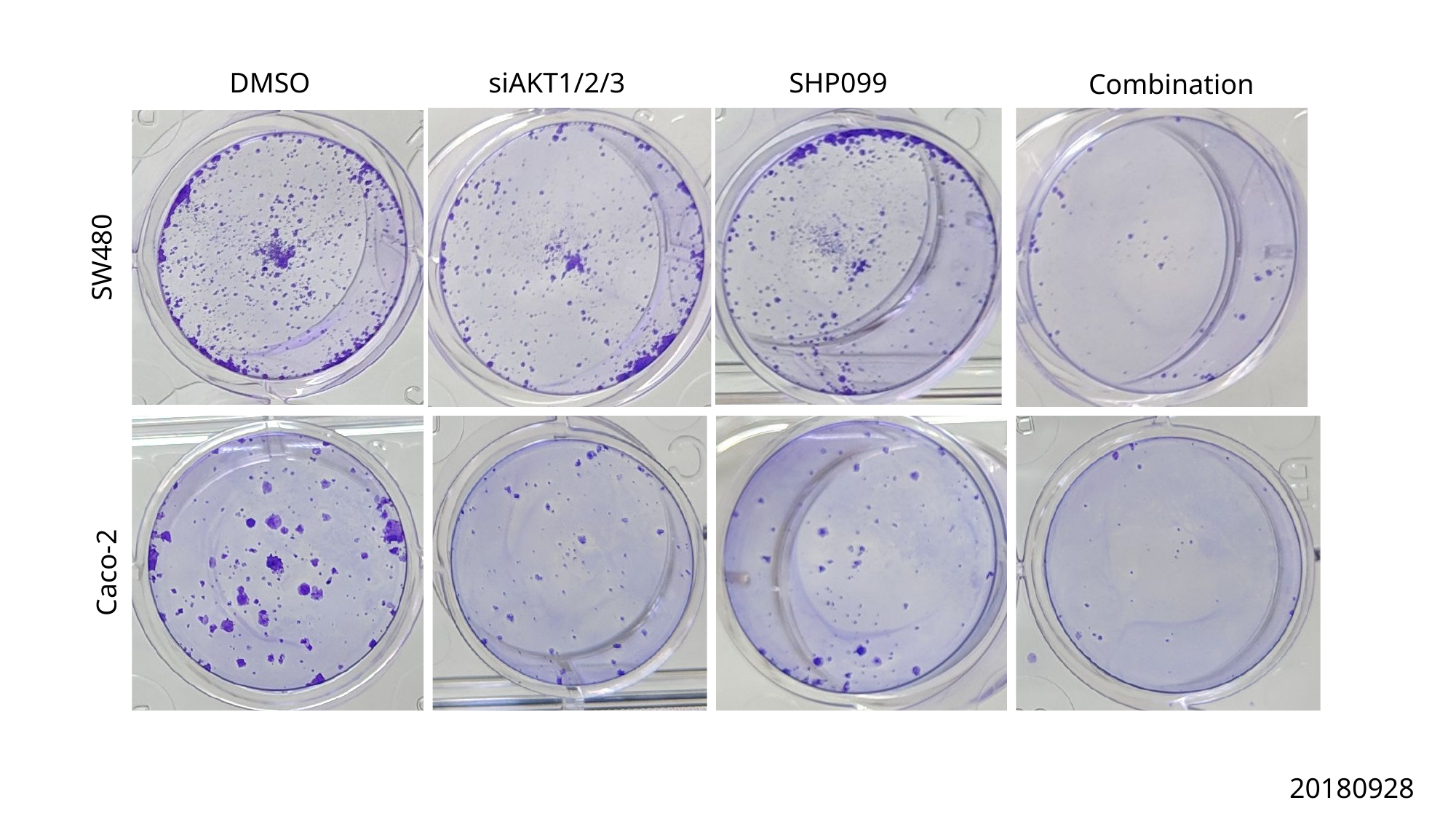

DMSO
siAKT1/2/3
SHP099
Combination
SW480
Caco-2
20180928

Supplement: Supplementary file 10 [file DataSheet10.ZIP › FigureS3/B/SW480 and Caco-2.pptx]

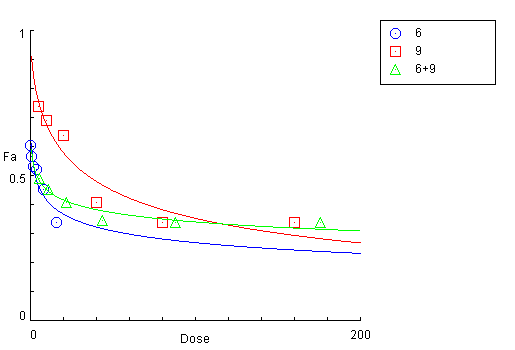

Supplement: Supplementary file 11 [file DataSheet6.ZIP › Figure3/Figure3B/CACO2/Caco2/Caco2-report/doseeffect.gif]

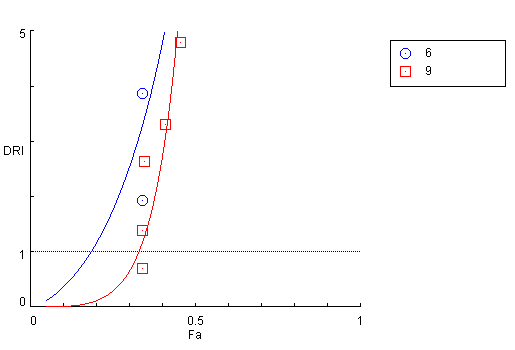

Supplement: Supplementary file 11 [file DataSheet6.ZIP › Figure3/Figure3B/CACO2/Caco2/Caco2-report/driplotcombo1.gif]

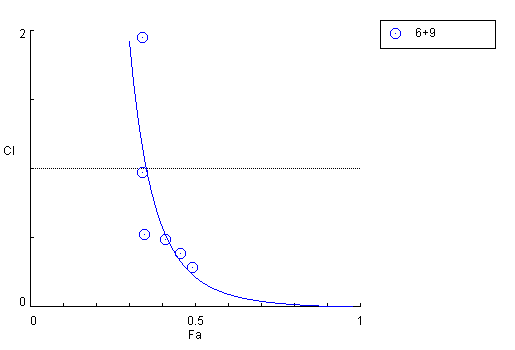

Supplement: Supplementary file 11 [file DataSheet6.ZIP › Figure3/Figure3B/CACO2/Caco2/Caco2-report/faci.gif]

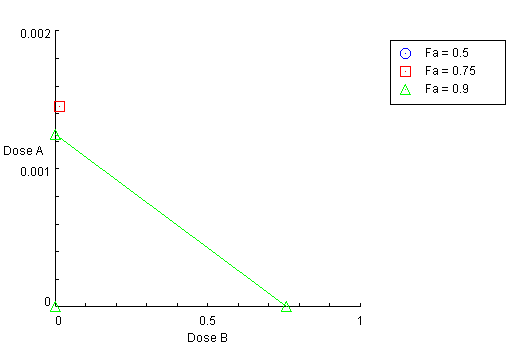

Supplement: Supplementary file 11 [file DataSheet6.ZIP › Figure3/Figure3B/CACO2/Caco2/Caco2-report/isocombo1.gif]

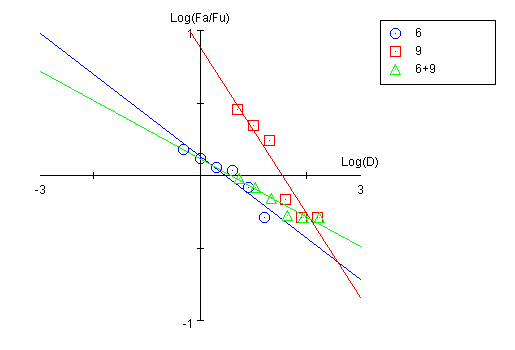

Supplement: Supplementary file 11 [file DataSheet6.ZIP › Figure3/Figure3B/CACO2/Caco2/Caco2-report/medianeffect.gif]

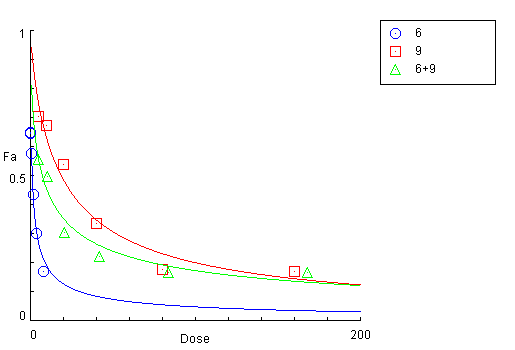

Supplement: Supplementary file 11 [file DataSheet6.ZIP › Figure3/Figure3B/Colo205/COLO205/COLO205-report/doseeffect.gif]

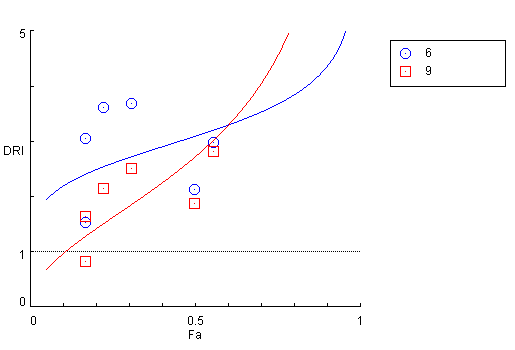

Supplement: Supplementary file 11 [file DataSheet6.ZIP › Figure3/Figure3B/Colo205/COLO205/COLO205-report/driplotcombo1.gif]

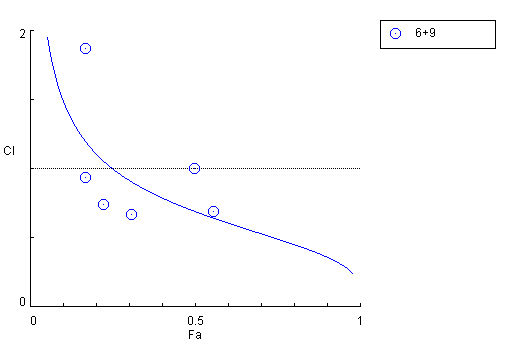

Supplement: Supplementary file 11 [file DataSheet6.ZIP › Figure3/Figure3B/Colo205/COLO205/COLO205-report/faci.gif]

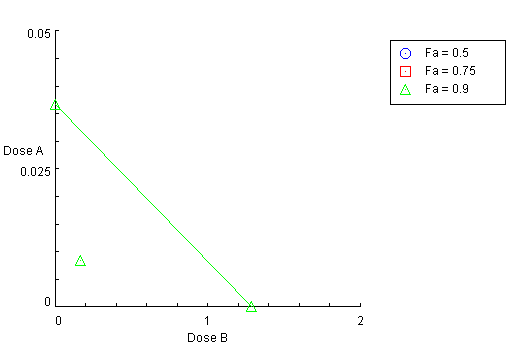

Supplement: Supplementary file 11 [file DataSheet6.ZIP › Figure3/Figure3B/Colo205/COLO205/COLO205-report/isocombo1.gif]

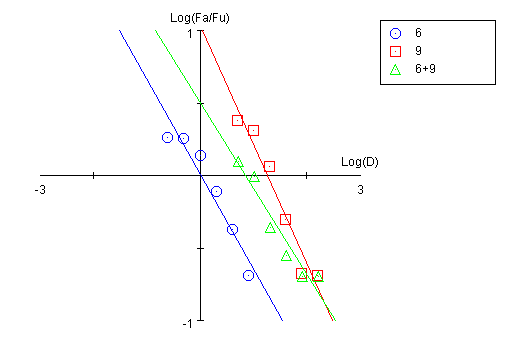

Supplement: Supplementary file 11 [file DataSheet6.ZIP › Figure3/Figure3B/Colo205/COLO205/COLO205-report/medianeffect.gif]

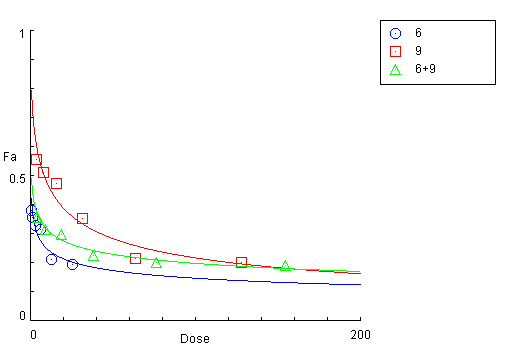

Supplement: Supplementary file 11 [file DataSheet6.ZIP › Figure3/Figure3B/RKO/RKO-report/doseeffect.gif]

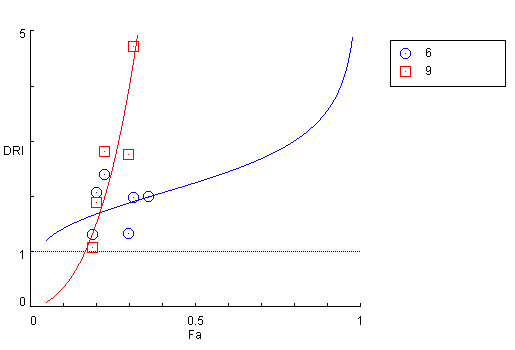

Supplement: Supplementary file 11 [file DataSheet6.ZIP › Figure3/Figure3B/RKO/RKO-report/driplotcombo1.gif]

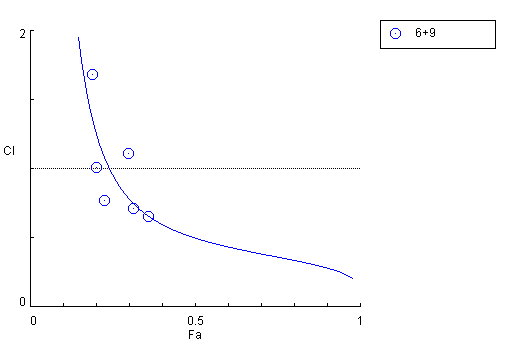

Supplement: Supplementary file 11 [file DataSheet6.ZIP › Figure3/Figure3B/RKO/RKO-report/faci.gif]

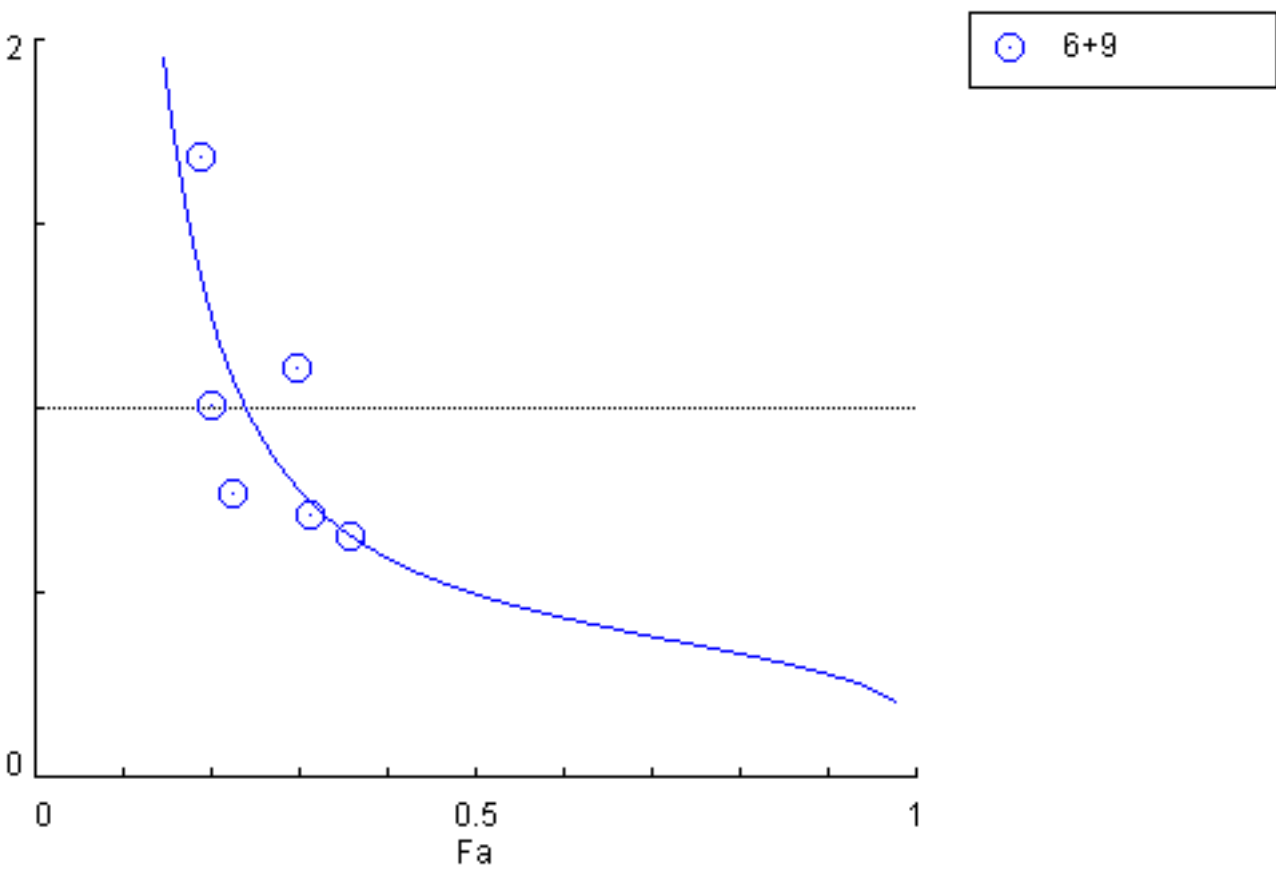

Supplement: Supplementary file 11 [file DataSheet6.ZIP › Figure3/Figure3B/RKO/RKO-report/faci.pdf]

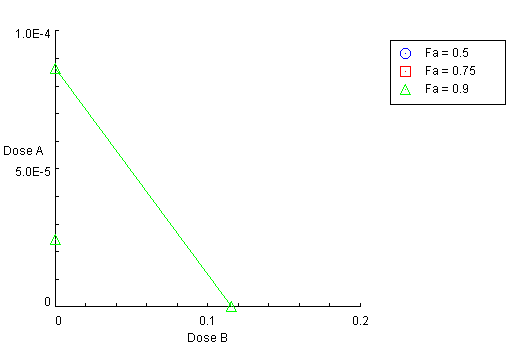

Supplement: Supplementary file 11 [file DataSheet6.ZIP › Figure3/Figure3B/RKO/RKO-report/isocombo1.gif]

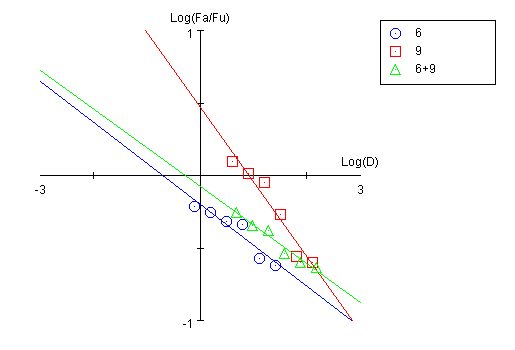

Supplement: Supplementary file 11 [file DataSheet6.ZIP › Figure3/Figure3B/RKO/RKO-report/medianeffect.gif]

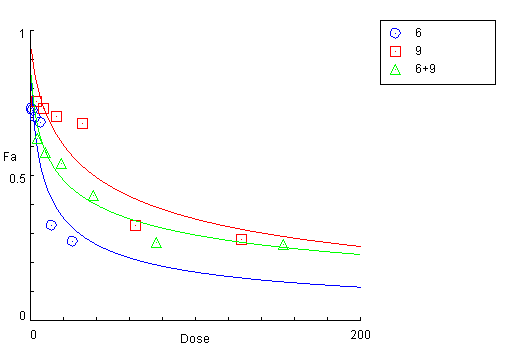

Supplement: Supplementary file 11 [file DataSheet6.ZIP › Figure3/Figure3B/SW480/480-report/doseeffect.gif]

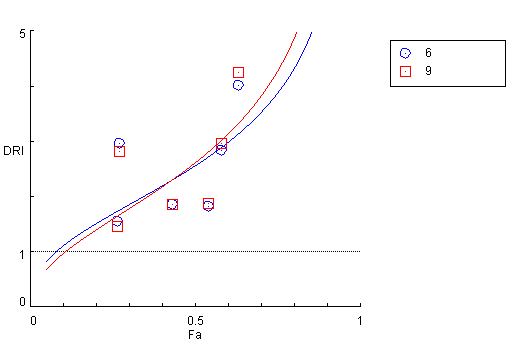

Supplement: Supplementary file 11 [file DataSheet6.ZIP › Figure3/Figure3B/SW480/480-report/driplotcombo1.gif]

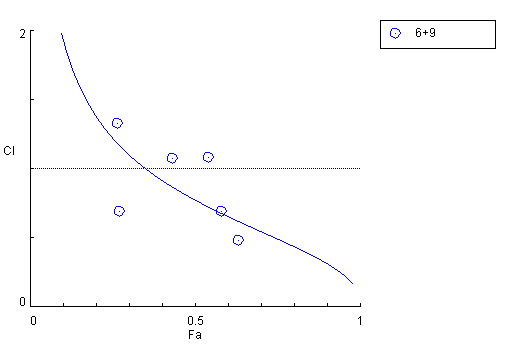

Supplement: Supplementary file 11 [file DataSheet6.ZIP › Figure3/Figure3B/SW480/480-report/faci.gif]

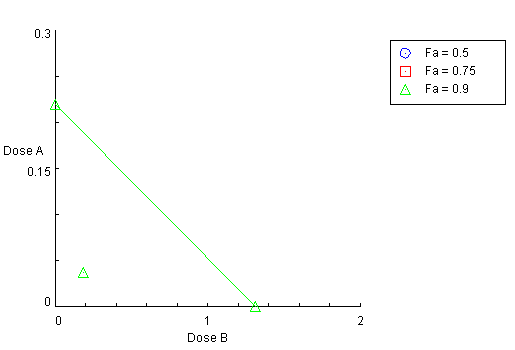

Supplement: Supplementary file 11 [file DataSheet6.ZIP › Figure3/Figure3B/SW480/480-report/isocombo1.gif]

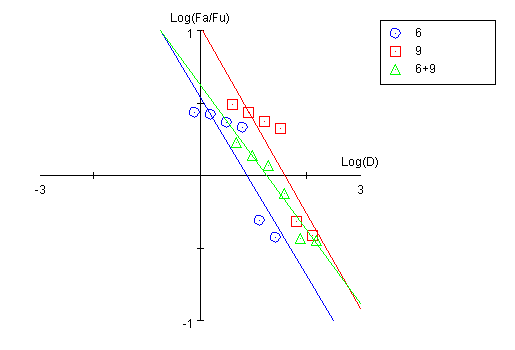

Supplement: Supplementary file 11 [file DataSheet6.ZIP › Figure3/Figure3B/SW480/480-report/medianeffect.gif]

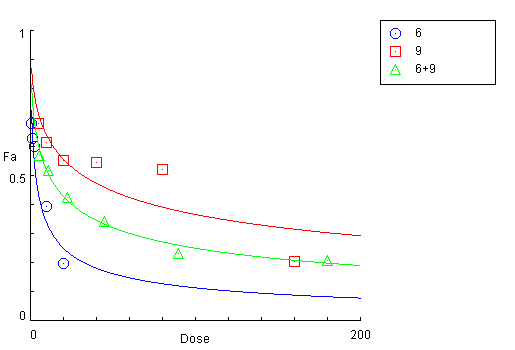

Supplement: Supplementary file 11 [file DataSheet6.ZIP › Figure3/Figure3B/SW620/SW620-report/doseeffect.gif]

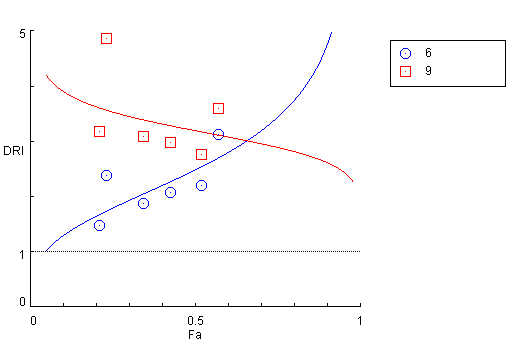

Supplement: Supplementary file 11 [file DataSheet6.ZIP › Figure3/Figure3B/SW620/SW620-report/driplotcombo1.gif]

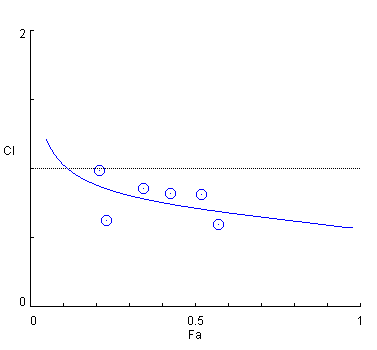

Supplement: Supplementary file 11 [file DataSheet6.ZIP › Figure3/Figure3B/SW620/SW620-report/faci.gif]

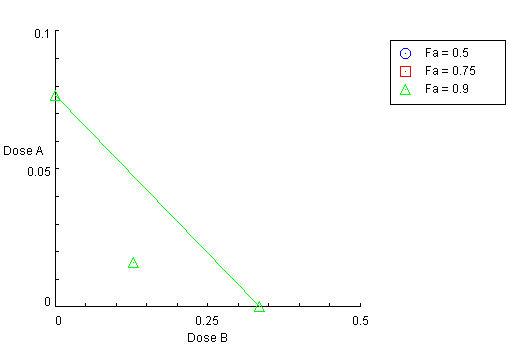

Supplement: Supplementary file 11 [file DataSheet6.ZIP › Figure3/Figure3B/SW620/SW620-report/isocombo1.gif]

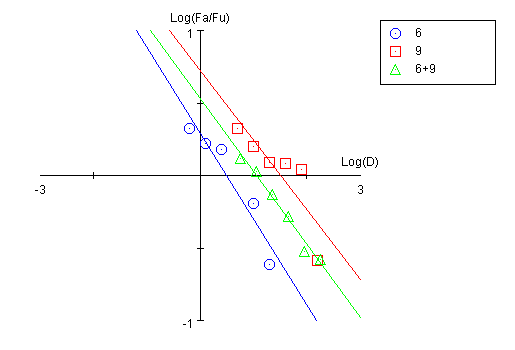

Supplement: Supplementary file 11 [file DataSheet6.ZIP › Figure3/Figure3B/SW620/SW620-report/medianeffect.gif]

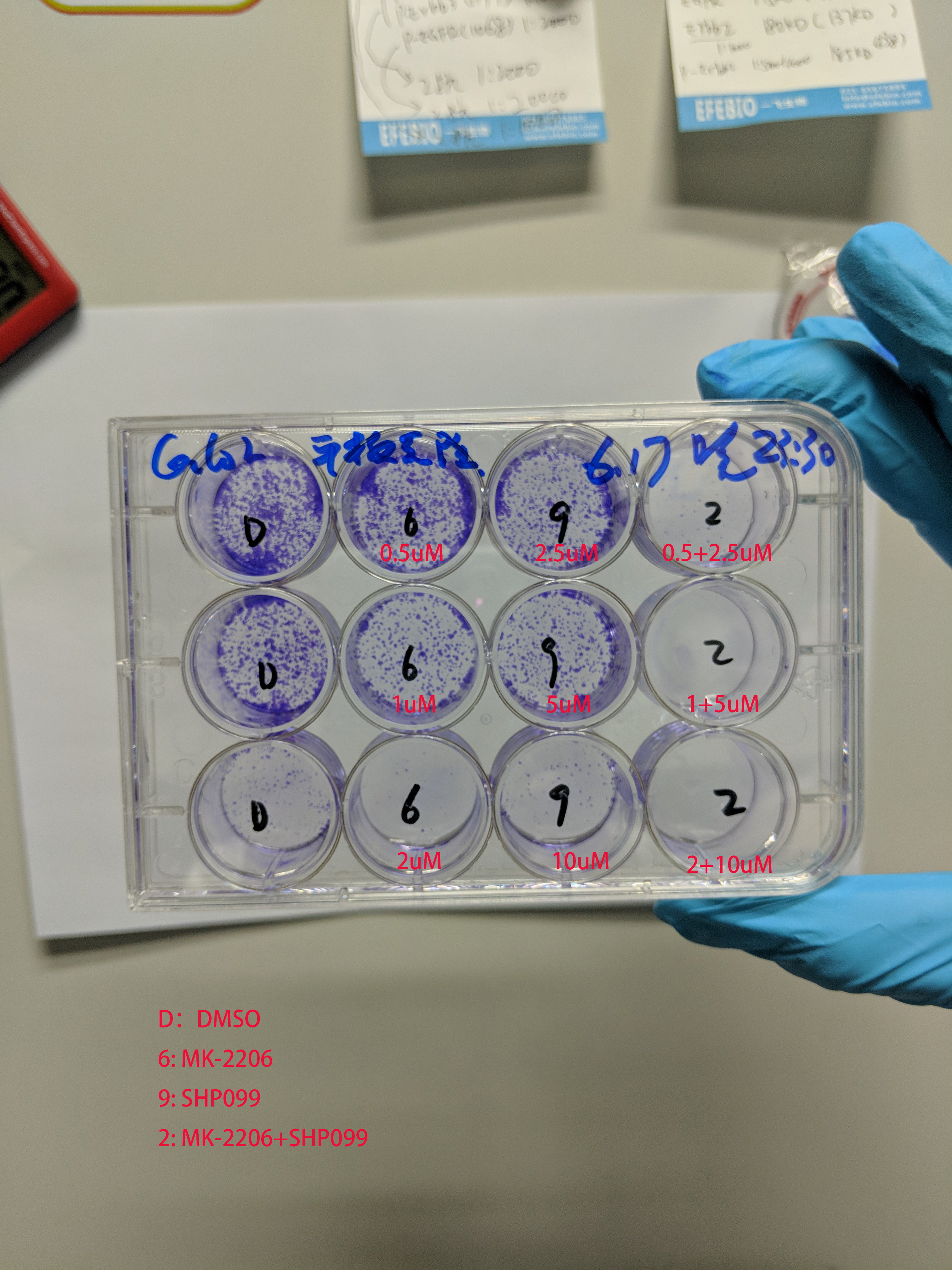

Supplement: Supplementary file 11 [file DataSheet6.ZIP › Figure3/Figure3C/CACO2/IMG_20180625_210822.jpg]

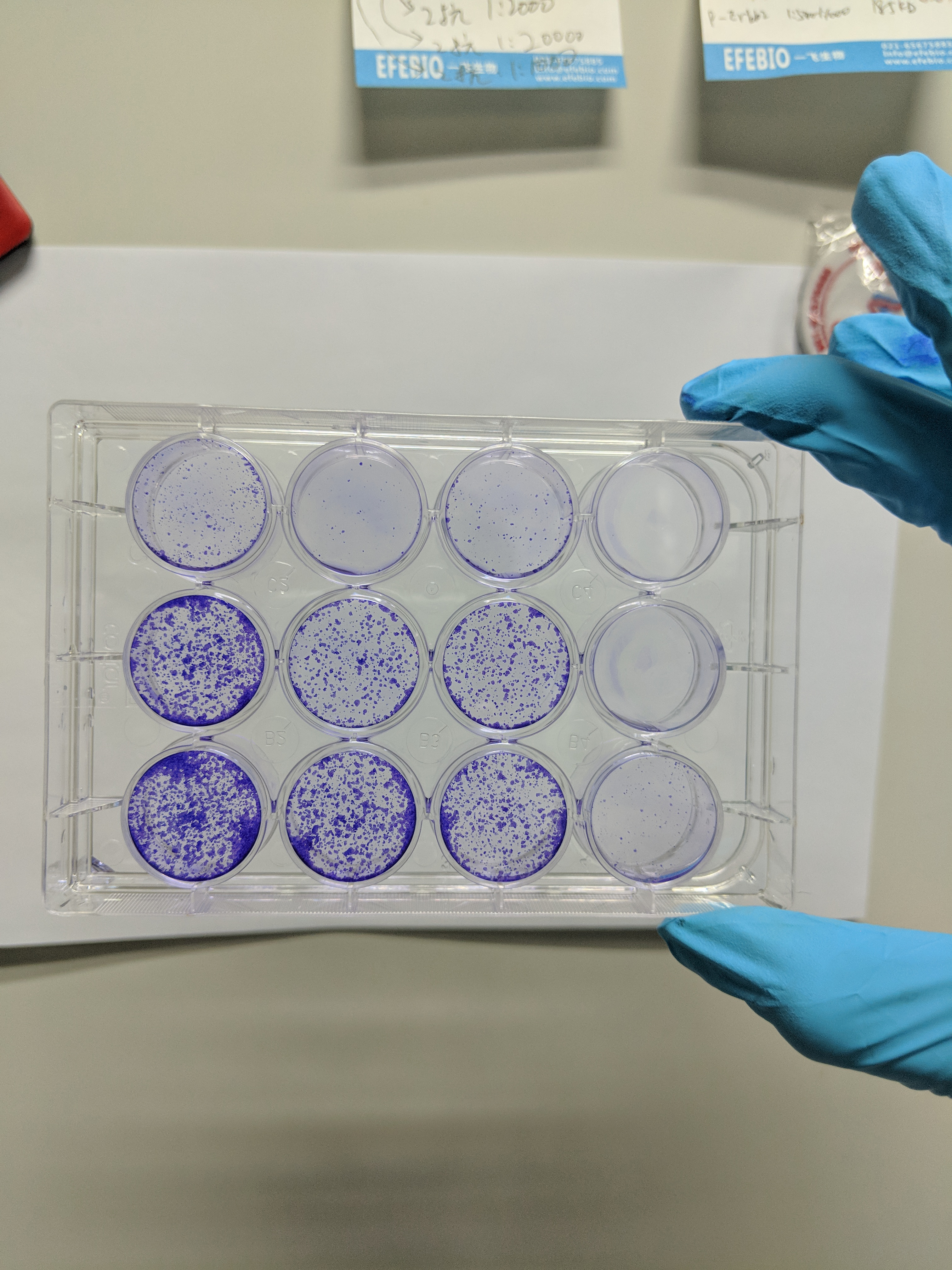

Supplement: Supplementary file 11 [file DataSheet6.ZIP › Figure3/Figure3C/CACO2/IMG_20180625_210945.jpg]

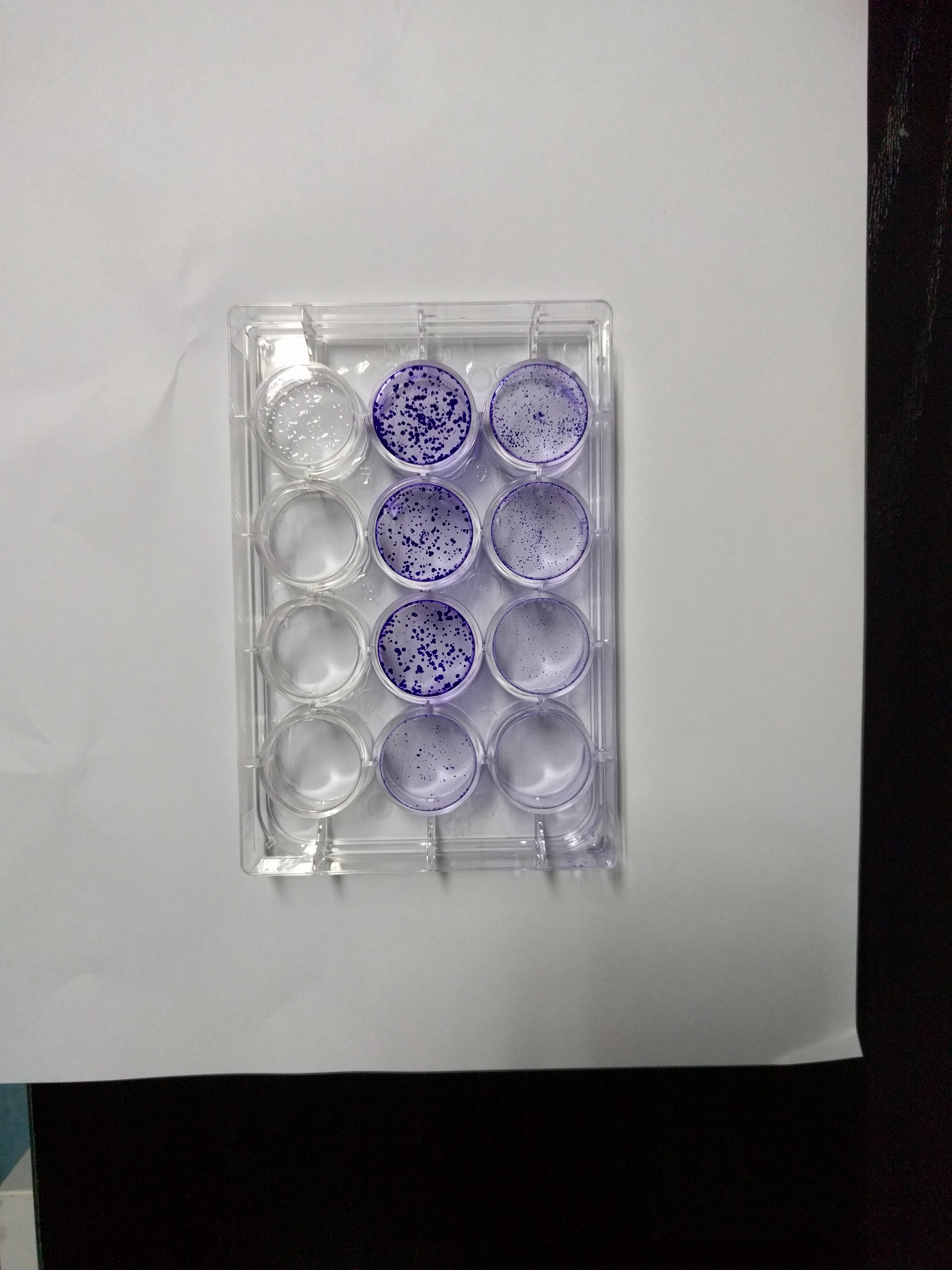

Supplement: Supplementary file 11 [file DataSheet6.ZIP › Figure3/Figure3C/RKO AND SW480/IMG_20171016_100813.jpg]

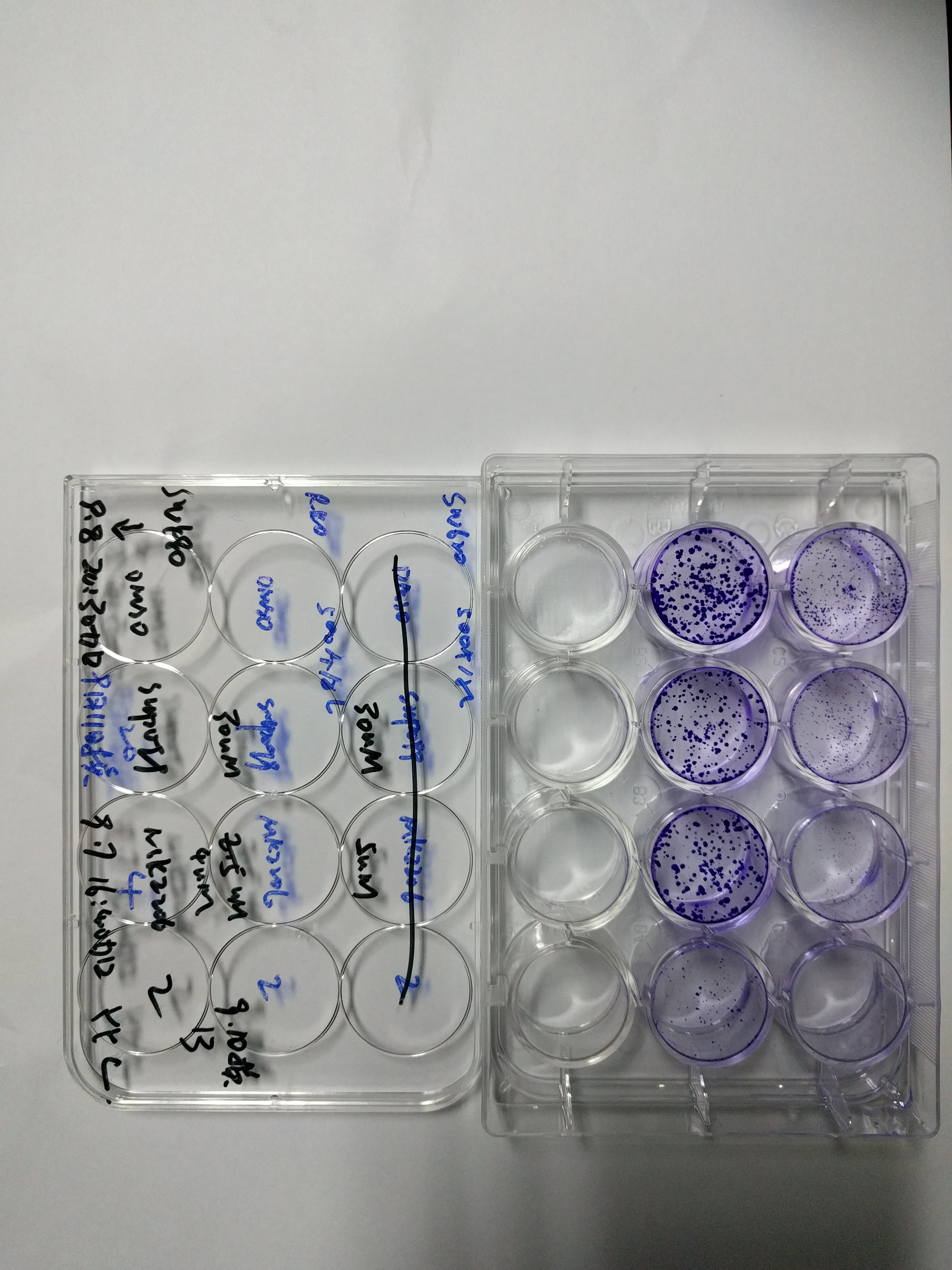

Supplement: Supplementary file 11 [file DataSheet6.ZIP › Figure3/Figure3C/RKO AND SW480/IMG_20171016_100902.jpg]

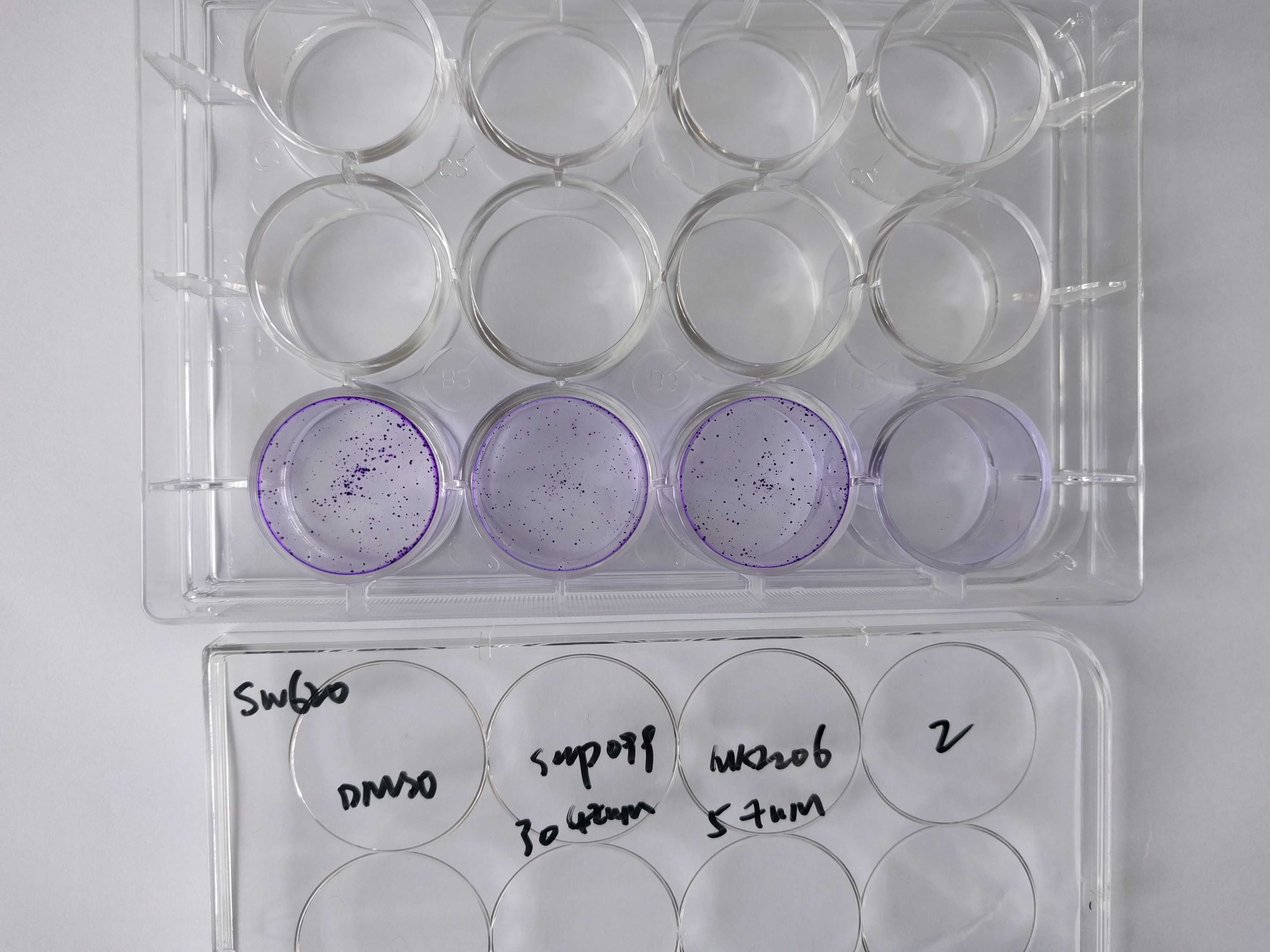

Supplement: Supplementary file 11 [file DataSheet6.ZIP › Figure3/Figure3C/SW620/IMG_20171022_123300.jpg]

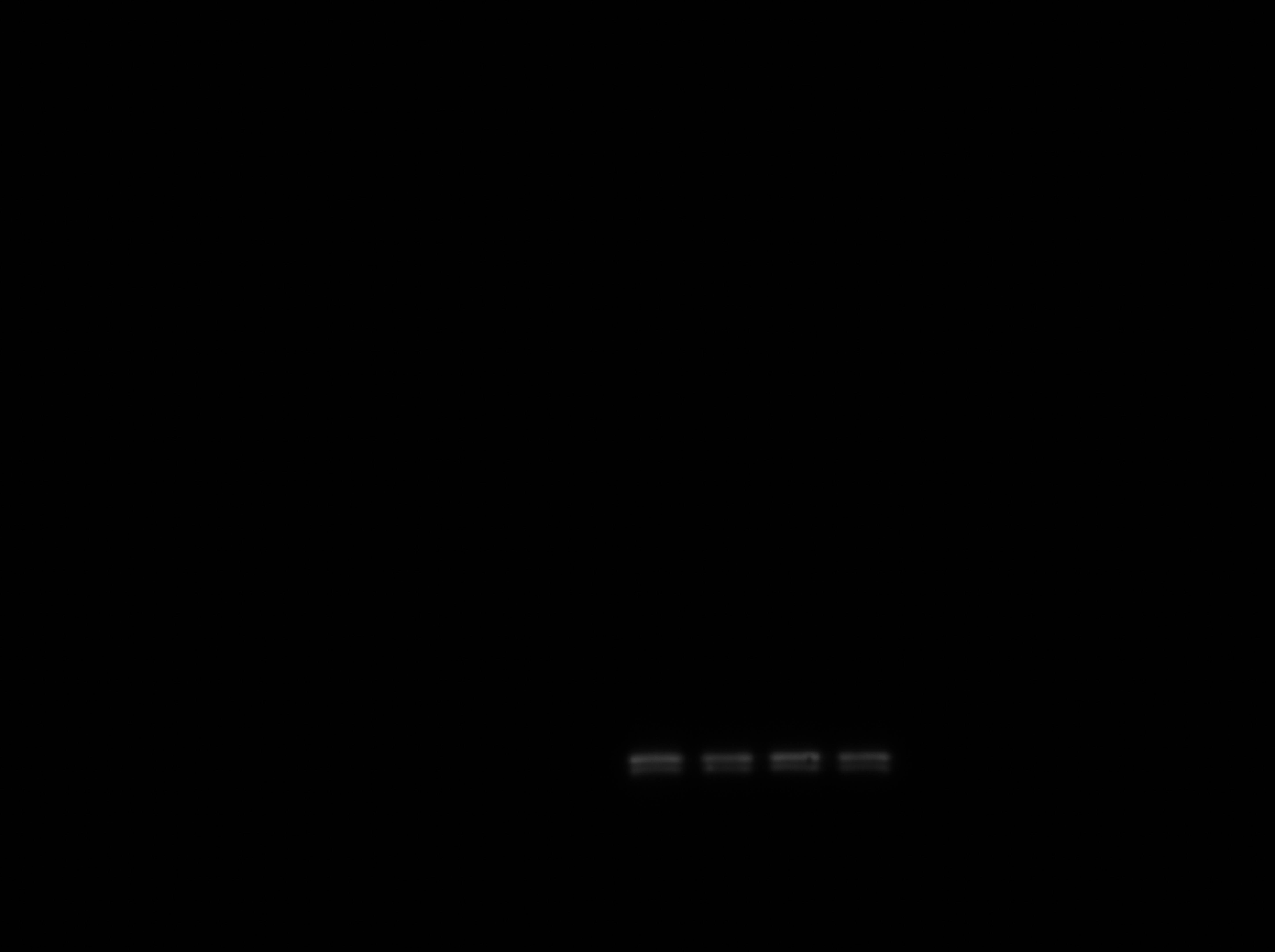

Supplement: Supplementary file 12 [file DataSheet5.ZIP › Figure2/Figure2A/AKT CW-2.jpg]

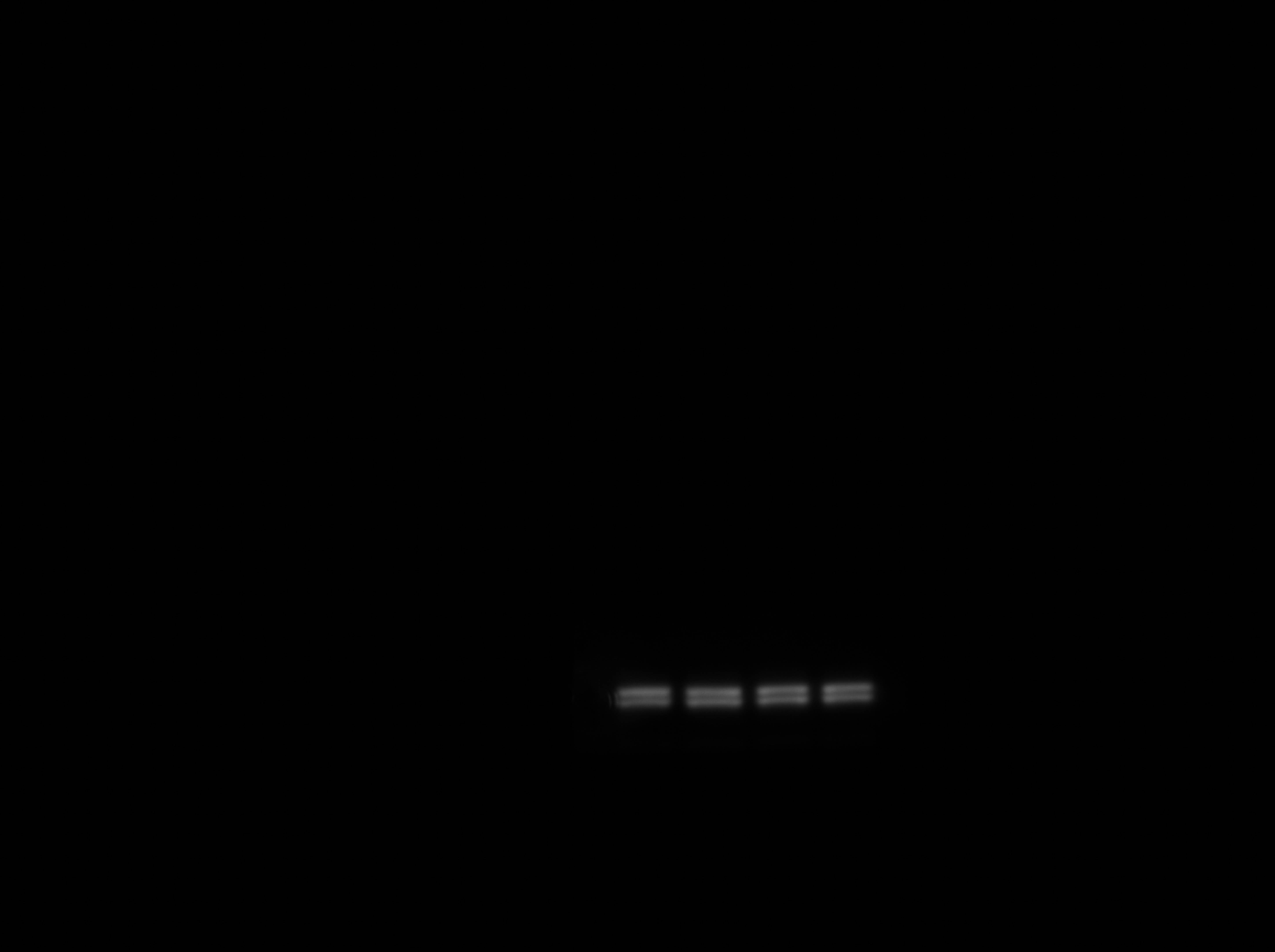

Supplement: Supplementary file 12 [file DataSheet5.ZIP › Figure2/Figure2A/AKT Caco-2.jpg]

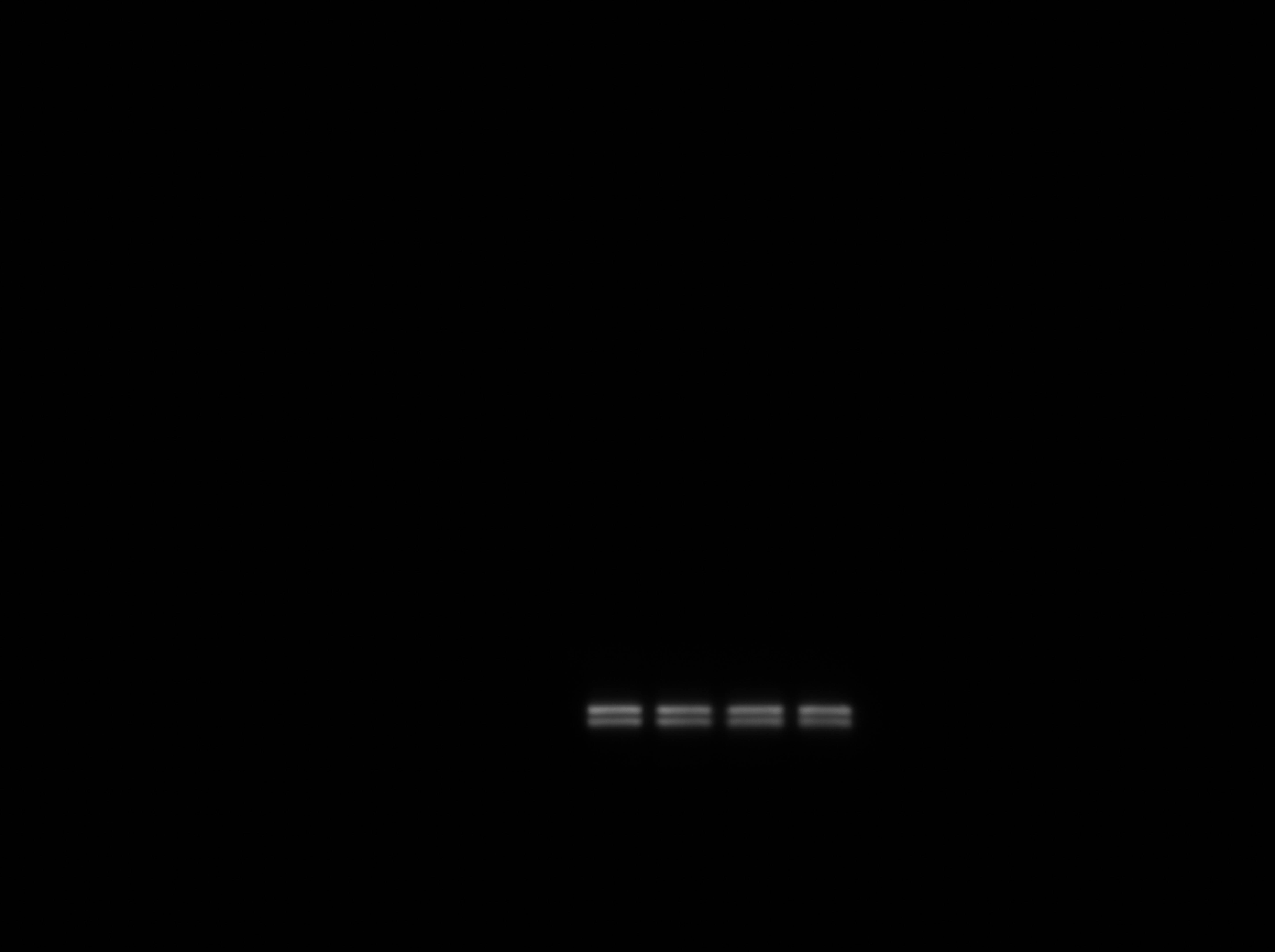

Supplement: Supplementary file 12 [file DataSheet5.ZIP › Figure2/Figure2A/AKT Colo205.jpg]

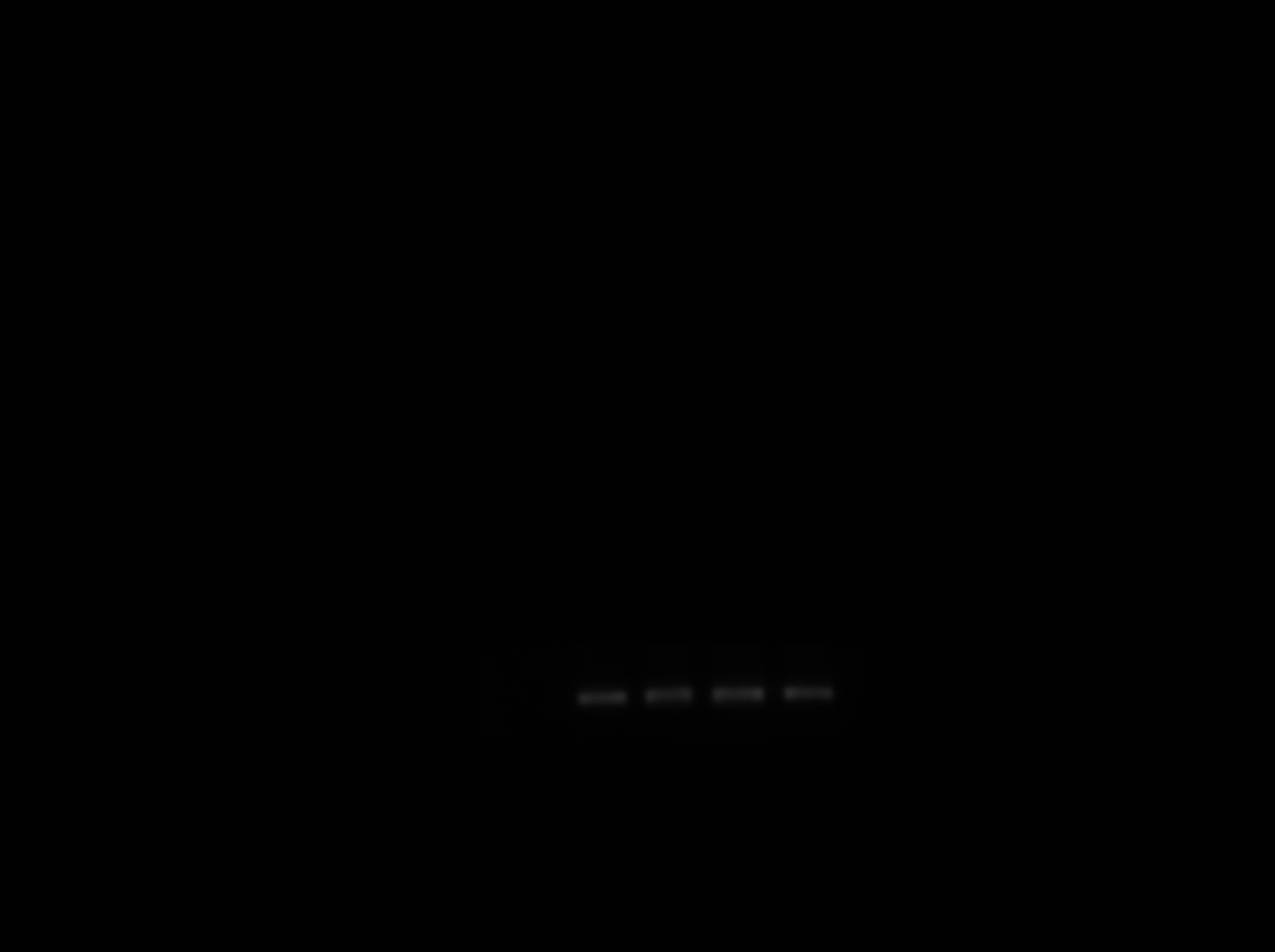

Supplement: Supplementary file 12 [file DataSheet5.ZIP › Figure2/Figure2A/AKT RKO.jpg]

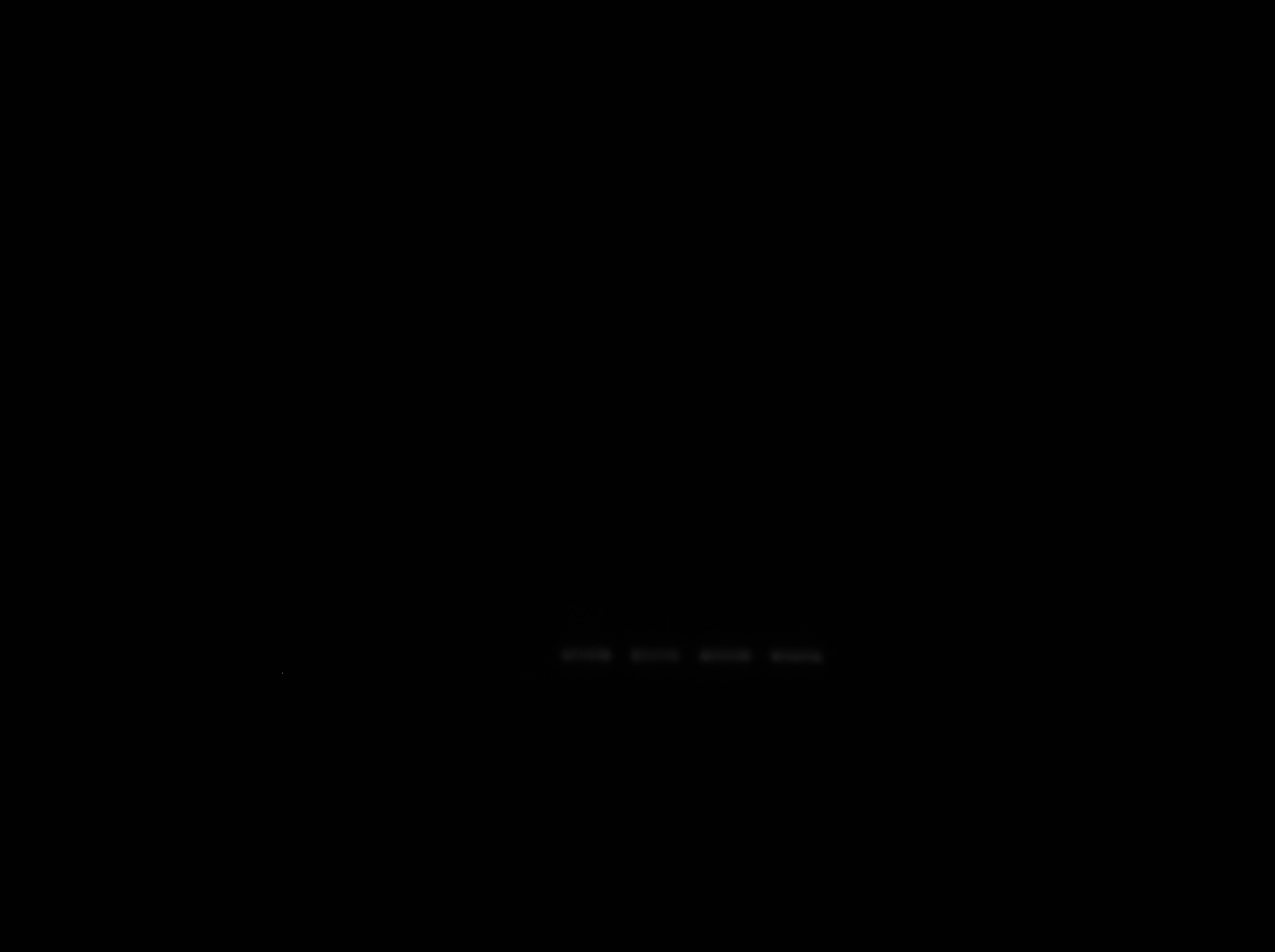

Supplement: Supplementary file 12 [file DataSheet5.ZIP › Figure2/Figure2A/AKT SW480.jpg]

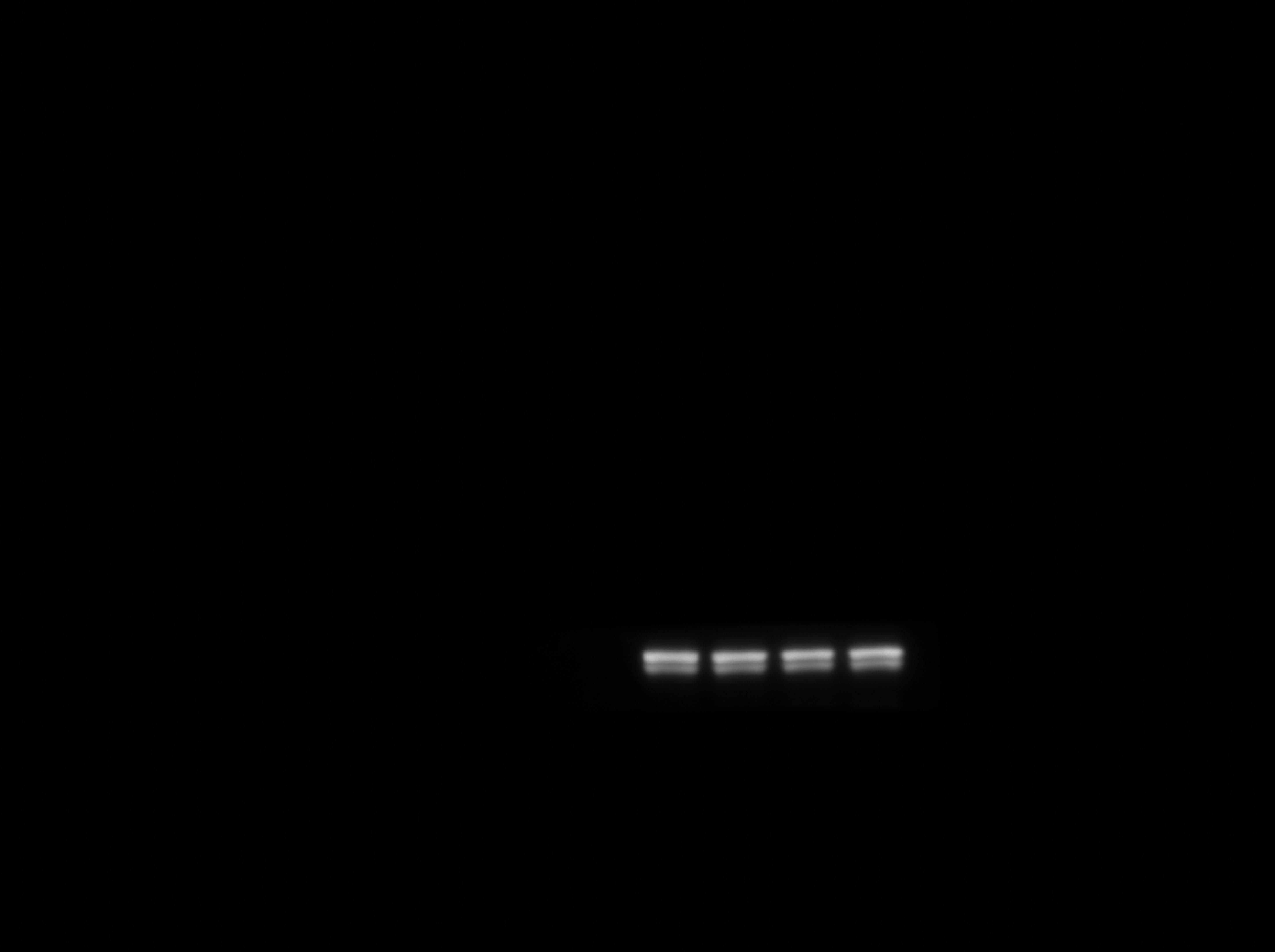

Supplement: Supplementary file 12 [file DataSheet5.ZIP › Figure2/Figure2A/AKT SW620.jpg]
