# Supplementary material for: Therapeutic Suppression of FAK-AKT Signaling Overcomes Resistance to SHP2 Inhibition in Colorectal Carcinoma
Source: Front Pharmacol. 2021 Nov 1;12:739501. doi: 10.3389/fphar.2021.739501 (PMC8591248; doi:10.3389/fphar.2021.739501)
Supplement: Supplementary file 5 [file DataSheet11.pdf]

Data Supplement

Title:

Therapeutic suppression of FAK-AKT signaling overcomes resistance to SHP2 inhibition in Colorectal Carcinoma

Supplementary Table 1. The baseline information of 365 CRC patients used in immunohistochemical exanimation of *p*-SHP2.

Supplementary Table 2. Sequences of siAKT1-3 and PCR primers of AKT1-3 used in the study.

Supplementary Figure 1. Expression pattern of *p*-SHP2 in cancer specimens according to multiple cancer types from TCGA specimens.

Supplementary Figure 2. The association between SHP099 resistance and the status of KRAS mutation, BRAF mutation, or *p*-SHP2 expression across different cancer cell lines.

Supplementary Figure 3. The effect of SHP099 and AKT1-3 inhibition with siRNAs on the proliferation and colony formation of CRC cells.

Supplementary Figure 4. The baselines of *p*-FAK and *p*-SHP2 were in 6 cell lines. *p*-FAK inhibition reduces the colony formation efficacy when combined with SHP099 in *p*-FAK high cells.

Supplementary Table 1. The baseline features of 365 patients used in immunohistochemical exanimation of *p*-SHP2.

Features	n (%)
**Age (yrs)**	
≤60	180 (49.3)
＞60	185 (50.7)
**Sex**	
Male	209 (57.3)
Female	156 (42.7)
**Tumor location**	
Colon	218 (59.7)
Rectum	147 (40.3)
**Differential grade**	
Well	18 (4.9)
Moderate	222 (60.8)
Poor	105 (28.8)
Missing	20 (5.5)
**Lymph nodes**	
<12	182 (49.9)
≥12	183 (50.1)
**TNM stage**	
I	16 (4.4)
II	230 (63.0)
III	119 (32.6)
**Chemotherapy**	
Yes	308 (84.4)
No	57 (15.6)
**Serum CEA**	
<5 ng/mL	217 (59.5)
≥5 ng/mL	148 (40.5)
**Serum CA199**	
<37 U/mL	301 (85.3)
≥37 U/mL	64 (17.5)

Supplementary Table 2. Sequences of siAKT1-3 and PCR primers of AKT1-3 used in the study.

**Names**	**Sequences (5'-3')**
AKT1 siRNA	TAATGTGCCCGTCCTTGTCTT
AKT2 siRNA	GGTGTCTGTCATCAAAGAATT
AKT3 siRNA	GGCAAGATGTATATGATAATT
AKT1-F	GTCATCGAACGCACCTTCCAT
AKT1-R	AGCTTCAGGTACTCAAACTCGT
AKT2-F	ACCACAGTCATCGAGAGGACC
AKT2-R	GGAGCCACACTTGTAGTCCA
AKT3-F	TGAAGTGGCACACACTCTAACT
AKT3-R	CCGCTCTCTCGACAAATGGA

FigureS1

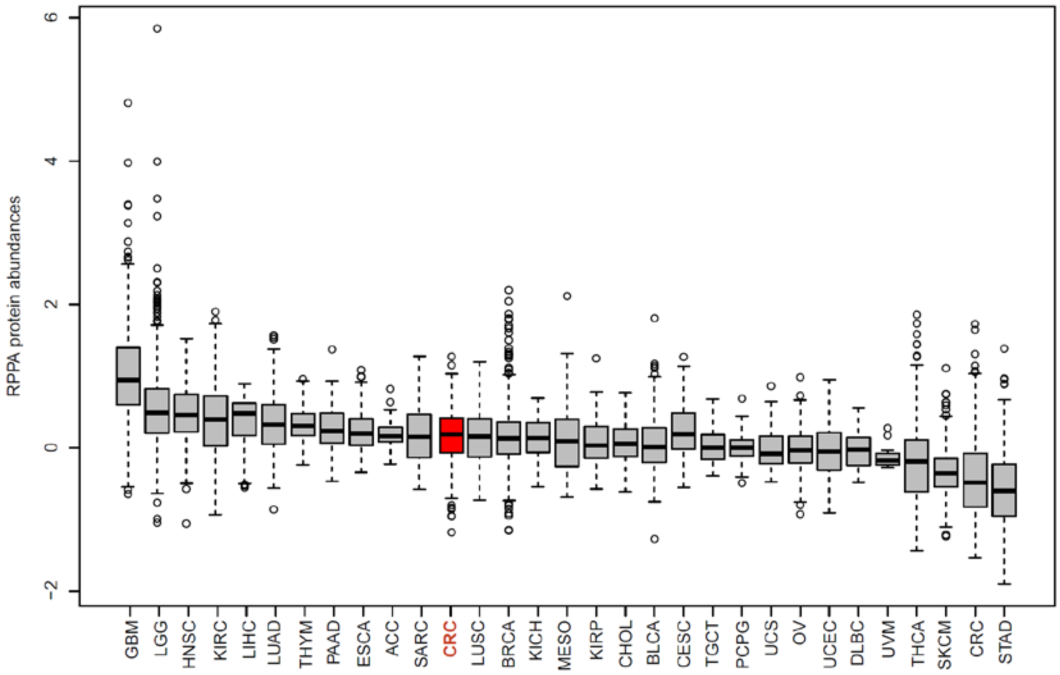

Supplementary Figure 1. Expression pattern of *p*-SHP2 in cancer specimens according to multiple cancer types from TCGA specimens. BLCA, Bladder Cancer; BRCA, Breast Cancer; CESC, Cervical Cancer; CHOL, Bile Duct Cancer; ESCA, Esophageal Cancer; HNSC, Head and Neck Cancer; KICH, Kidney Chromophobe; KIRC, Kidney Clear Cell Carcinoma; KIRP, Kidney Papillary Cell Carcinoma; LIHC, Liver Cancer; LUAD, Lung Adenocarcinoma; LUSC, Lung Squamous Cell Carcinoma; PAAD, Pancreatic Cancer; PCPG, Pheochromocytoma & Paraganglioma; PRAD, Prostate Cancer; SARC, Sarcoma; SKCM, Melanoma; STAD, Stomach Cancer; THCA, Thyroid Cancer; THYM, Thymoma; UCEC, Endometrioid Cancer.

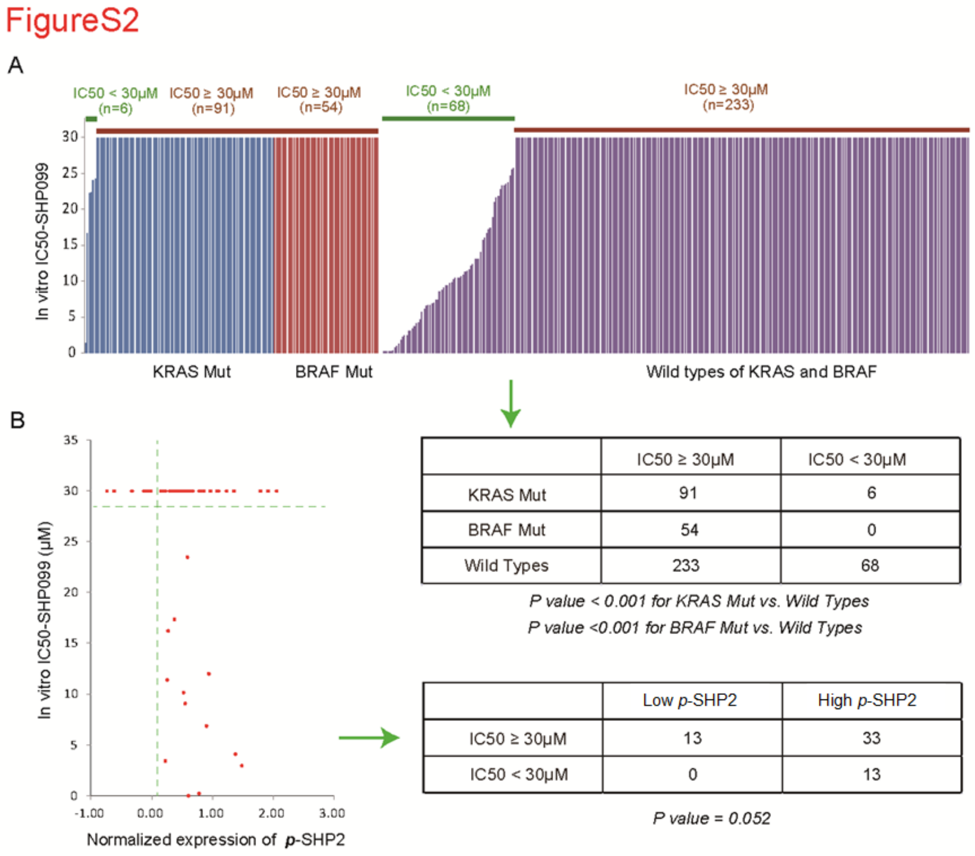

	IC50 ≥ 30μM	IC50 < 30μM
KRAS Mut	91	6
BRAF Mut	54	0
Wild Types	233	68

	Low *p*-SHP2	High *p*-SHP2
IC50 ≥ 30μM	13	33
IC50 < 30μM	0	13

Supplementary Figure 2. The association between SHP099 resistance and the status of KRAS mutation, BRAF mutation, or p-SHP2 expression across diffrent cancer cell lines.

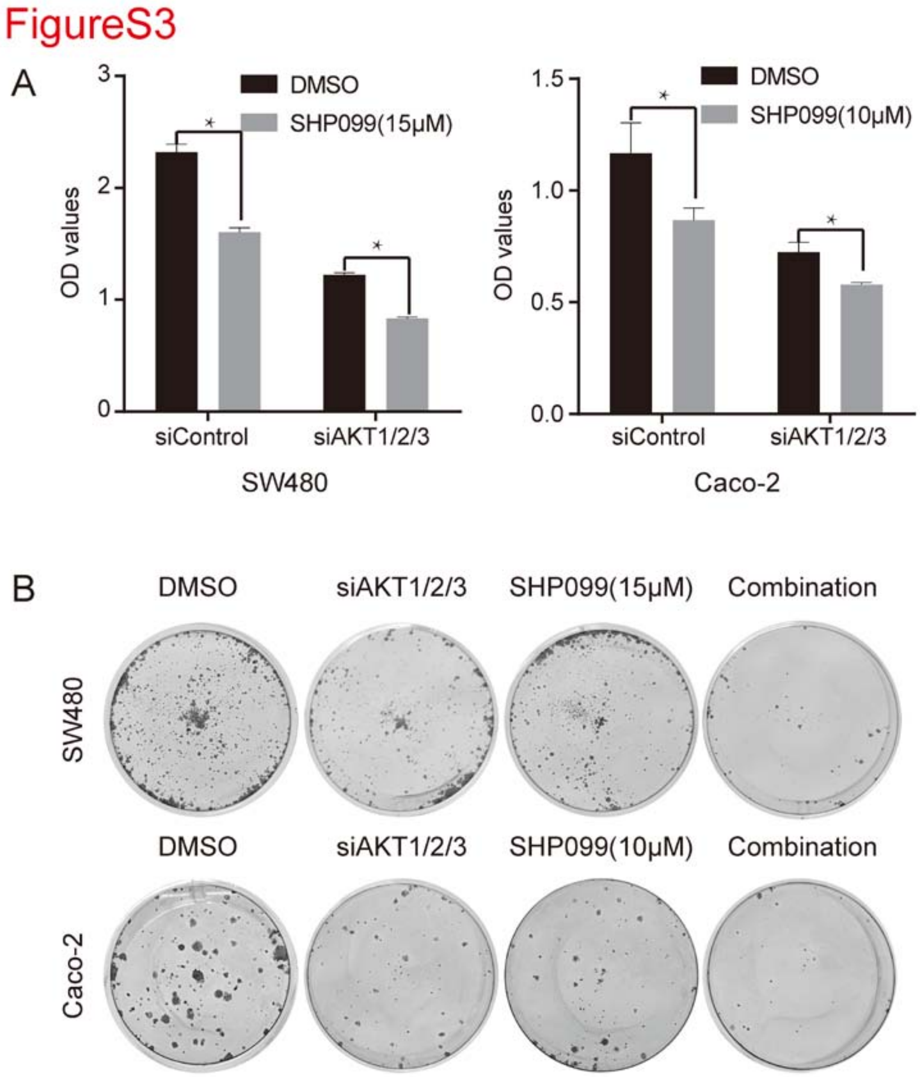

Supplementary Figure 3. The effect of SHP099 and AKT1-3 inhibition with siRNAs on the proliferation and colony formation of CRC cells.

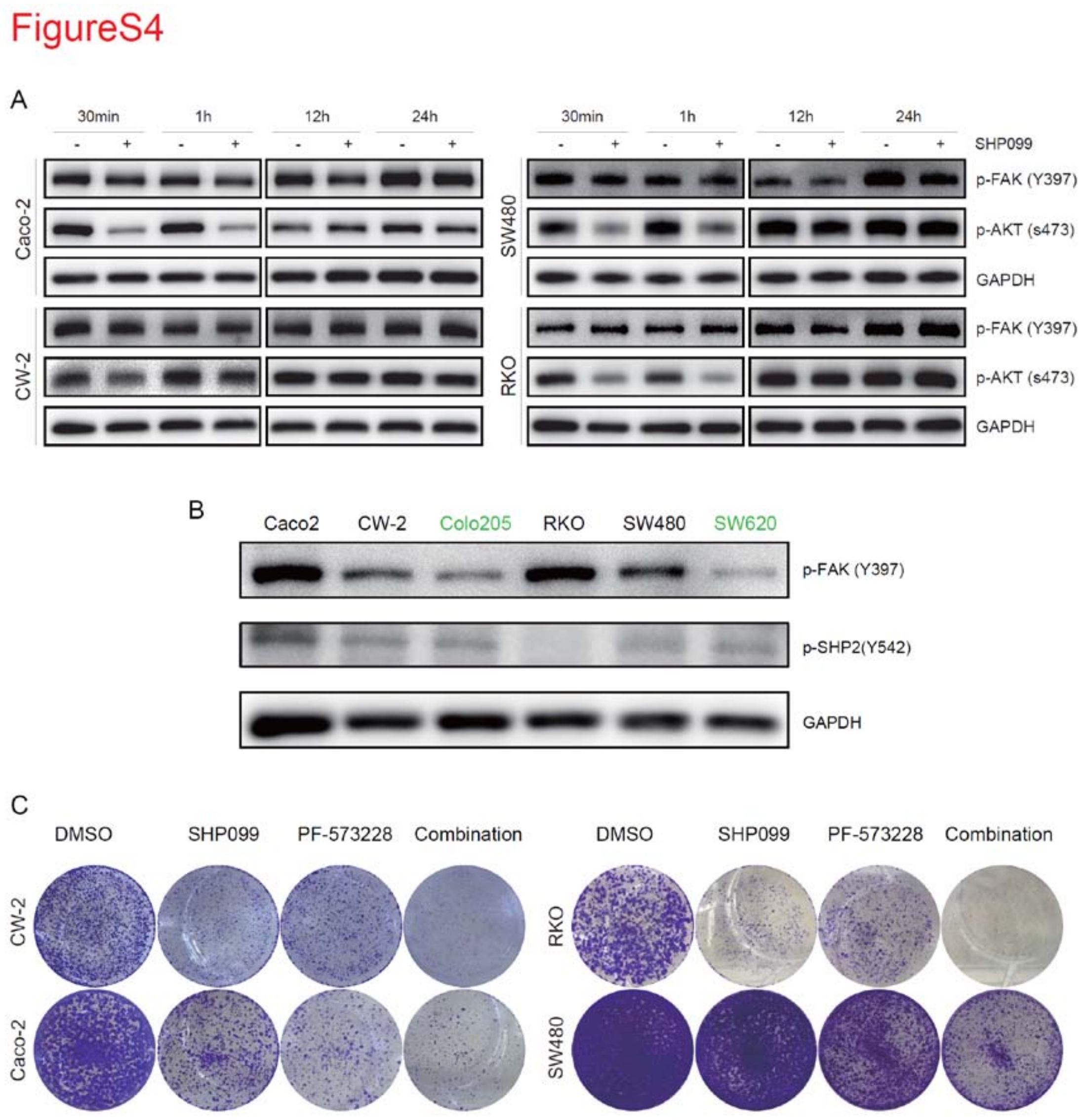

Supplementary Figure 4. *p*-FAK inhibition reduces the colony formation efficacy when given in combination with SHP099 in *p*-FAK high cells.
