# Supplementary material for: Therapeutic Suppression of FAK-AKT Signaling Overcomes Resistance to SHP2 Inhibition in Colorectal Carcinoma
Source: Front Pharmacol. 2021 Nov 1;12:739501. doi: 10.3389/fphar.2021.739501 (PMC8591248; doi:10.3389/fphar.2021.739501)
Supplement: Supplementary file 11 [file DataSheet6.ZIP › Figure3/Figure3B/CACO2/Caco2/Caco2-report/report.html]

CompuSyn Report


CompuSyn Report

|  |  |  |  |  |  |  |  |
| --- | --- | --- | --- | --- | --- | --- | --- |
| Experiment Name: CACO2|  |  |  |  |  |  | | --- | --- | --- | --- | --- | --- | | Date: |  |  |  |  | | --- | --- | --- | --- | | File Name: L:\Ô¬Ê¦ÐÖÊý¾Ý\20180523 coca2 CI\Caco2\Caco2.cse|  |  | | --- | --- | | Description  | | | | | | | |

|  |  |  |  |  |  |
| --- | --- | --- | --- | --- | --- |
| Drug: MK-2206 (6) [uM]|  |  |  |  | | --- | --- | --- | --- | | Drug: SHP099 (9) [uM]|  |  | | --- | --- | | Drug Combo: MK-2206+SHP099 (6+9) (6+9 [1:10]) | | | | | |

---

Data for Drug: 6 [uM]

| Dose Effect | |
| --- | --- |
| 0.5 0.605|  |  |  |  |  |  |  |  |  |  | | --- | --- | --- | --- | --- | --- | --- | --- | --- | --- | | 1.0 0.568|  |  |  |  |  |  |  |  | | --- | --- | --- | --- | --- | --- | --- | --- | | 2.0 0.534|  |  |  |  |  |  | | --- | --- | --- | --- | --- | --- | | 4.0 0.523|  |  |  |  | | --- | --- | --- | --- | | 8.0 0.453|  |  | | --- | --- | | 16.0 0.341 | | | | | | | | | | | |

6 data points entered.

|  |  |  |  |  |  |  |  |  |  |
| --- | --- | --- | --- | --- | --- | --- | --- | --- | --- |
| X-int: 0.47227|  |  |  |  |  |  |  |  | | --- | --- | --- | --- | --- | --- | --- | --- | | Y-int: 0.13348 +/- 0.03349|  |  |  |  |  |  | | --- | --- | --- | --- | --- | --- | | m: -0.2826 +/- 0.04895|  |  |  |  | | --- | --- | --- | --- | | Dm: 2.96666|  |  | | --- | --- | | r: -0.9449 | | | | | | | | | |

---

Data for Drug: 9 [uM]

| Dose Effect | |
| --- | --- |
| 5.0 0.741|  |  |  |  |  |  |  |  |  |  | | --- | --- | --- | --- | --- | --- | --- | --- | --- | --- | | 10.0 0.691|  |  |  |  |  |  |  |  | | --- | --- | --- | --- | --- | --- | --- | --- | | 20.0 0.639|  |  |  |  |  |  | | --- | --- | --- | --- | --- | --- | | 40.0 0.408|  |  |  |  | | --- | --- | --- | --- | | 80.0 0.341|  |  | | --- | --- | | 160.0 0.34 | | | | | | | | | | | |

6 data points entered.

|  |  |  |  |  |  |  |  |  |  |
| --- | --- | --- | --- | --- | --- | --- | --- | --- | --- |
| X-int: 1.54405|  |  |  |  |  |  |  |  | | --- | --- | --- | --- | --- | --- | --- | --- | | Y-int: 0.88509 +/- 0.12985|  |  |  |  |  |  | | --- | --- | --- | --- | --- | --- | | m: -0.5732 +/- 0.08432|  |  |  |  | | --- | --- | --- | --- | | Dm: 34.9989|  |  | | --- | --- | | r: -0.9593 | | | | | | | | | |

---

Data for Drug Combo: 6+9 (6+9 [1:10])

| Dose A Effect | |
| --- | --- |
| 0.5+ 0.492|  |  |  |  |  |  |  |  |  |  | | --- | --- | --- | --- | --- | --- | --- | --- | --- | --- | | 1.0+ 0.455|  |  |  |  |  |  |  |  | | --- | --- | --- | --- | --- | --- | --- | --- | | 2.0+ 0.41|  |  |  |  |  |  | | --- | --- | --- | --- | --- | --- | | 4.0+ 0.347|  |  |  |  | | --- | --- | --- | --- | | 8.0+ 0.34|  |  | | --- | --- | | 16.0+ 0.34 | | | | | | | | | | | |

6 data points entered.

|  |  |  |  |  |  |  |  |  |  |
| --- | --- | --- | --- | --- | --- | --- | --- | --- | --- |
| X-int: 0.57936|  |  |  |  |  |  |  |  | | --- | --- | --- | --- | --- | --- | --- | --- | | Y-int: 0.11638 +/- 0.05108|  |  |  |  |  |  | | --- | --- | --- | --- | --- | --- | | m: -0.2009 +/- 0.03235|  |  |  |  | | --- | --- | --- | --- | | Dm: 3.79631|  |  | | --- | --- | | r: -0.9518 | | | | | | | | | |

---

Dose-Effect Curve  


---

Median-Effect Plot  


---

CI Data for Drug Combo: 6+9 (6+9 [1:10])

| Fa CI Value Total Dose | | |
| --- | --- | --- |
| 0.05 1354.40 8818695|  |  |  |  |  |  |  |  |  |  |  |  |  |  |  |  |  |  |  |  |  |  |  |  |  |  |  |  |  |  |  |  |  |  |  |  |  |  |  |  |  |  |  |  |  |  |  |  |  |  |  |  |  |  |  |  |  | | --- | --- | --- | --- | --- | --- | --- | --- | --- | --- | --- | --- | --- | --- | --- | --- | --- | --- | --- | --- | --- | --- | --- | --- | --- | --- | --- | --- | --- | --- | --- | --- | --- | --- | --- | --- | --- | --- | --- | --- | --- | --- | --- | --- | --- | --- | --- | --- | --- | --- | --- | --- | --- | --- | --- | --- | --- | | 0.1 122.910 213740.|  |  |  |  |  |  |  |  |  |  |  |  |  |  |  |  |  |  |  |  |  |  |  |  |  |  |  |  |  |  |  |  |  |  |  |  |  |  |  |  |  |  |  |  |  |  |  |  |  |  |  |  |  |  | | --- | --- | --- | --- | --- | --- | --- | --- | --- | --- | --- | --- | --- | --- | --- | --- | --- | --- | --- | --- | --- | --- | --- | --- | --- | --- | --- | --- | --- | --- | --- | --- | --- | --- | --- | --- | --- | --- | --- | --- | --- | --- | --- | --- | --- | --- | --- | --- | --- | --- | --- | --- | --- | --- | | 0.15 28.3317 21363.2|  |  |  |  |  |  |  |  |  |  |  |  |  |  |  |  |  |  |  |  |  |  |  |  |  |  |  |  |  |  |  |  |  |  |  |  |  |  |  |  |  |  |  |  |  |  |  |  |  |  |  | | --- | --- | --- | --- | --- | --- | --- | --- | --- | --- | --- | --- | --- | --- | --- | --- | --- | --- | --- | --- | --- | --- | --- | --- | --- | --- | --- | --- | --- | --- | --- | --- | --- | --- | --- | --- | --- | --- | --- | --- | --- | --- | --- | --- | --- | --- | --- | --- | --- | --- | --- | | 0.2 9.58347 3772.32|  |  |  |  |  |  |  |  |  |  |  |  |  |  |  |  |  |  |  |  |  |  |  |  |  |  |  |  |  |  |  |  |  |  |  |  |  |  |  |  |  |  |  |  |  |  |  |  | | --- | --- | --- | --- | --- | --- | --- | --- | --- | --- | --- | --- | --- | --- | --- | --- | --- | --- | --- | --- | --- | --- | --- | --- | --- | --- | --- | --- | --- | --- | --- | --- | --- | --- | --- | --- | --- | --- | --- | --- | --- | --- | --- | --- | --- | --- | --- | --- | | 0.25 4.00820 900.789|  |  |  |  |  |  |  |  |  |  |  |  |  |  |  |  |  |  |  |  |  |  |  |  |  |  |  |  |  |  |  |  |  |  |  |  |  |  |  |  |  |  |  |  |  | | --- | --- | --- | --- | --- | --- | --- | --- | --- | --- | --- | --- | --- | --- | --- | --- | --- | --- | --- | --- | --- | --- | --- | --- | --- | --- | --- | --- | --- | --- | --- | --- | --- | --- | --- | --- | --- | --- | --- | --- | --- | --- | --- | --- | --- | | 0.3 1.92129 257.791|  |  |  |  |  |  |  |  |  |  |  |  |  |  |  |  |  |  |  |  |  |  |  |  |  |  |  |  |  |  |  |  |  |  |  |  |  |  |  |  |  |  | | --- | --- | --- | --- | --- | --- | --- | --- | --- | --- | --- | --- | --- | --- | --- | --- | --- | --- | --- | --- | --- | --- | --- | --- | --- | --- | --- | --- | --- | --- | --- | --- | --- | --- | --- | --- | --- | --- | --- | --- | --- | --- | | 0.35 1.01370 82.7484|  |  |  |  |  |  |  |  |  |  |  |  |  |  |  |  |  |  |  |  |  |  |  |  |  |  |  |  |  |  |  |  |  |  |  |  |  |  |  | | --- | --- | --- | --- | --- | --- | --- | --- | --- | --- | --- | --- | --- | --- | --- | --- | --- | --- | --- | --- | --- | --- | --- | --- | --- | --- | --- | --- | --- | --- | --- | --- | --- | --- | --- | --- | --- | --- | --- | | 0.4 0.57449 28.5759|  |  |  |  |  |  |  |  |  |  |  |  |  |  |  |  |  |  |  |  |  |  |  |  |  |  |  |  |  |  |  |  |  |  |  |  | | --- | --- | --- | --- | --- | --- | --- | --- | --- | --- | --- | --- | --- | --- | --- | --- | --- | --- | --- | --- | --- | --- | --- | --- | --- | --- | --- | --- | --- | --- | --- | --- | --- | --- | --- | --- | | 0.45 0.34400 10.3092|  |  |  |  |  |  |  |  |  |  |  |  |  |  |  |  |  |  |  |  |  |  |  |  |  |  |  |  |  |  |  |  |  | | --- | --- | --- | --- | --- | --- | --- | --- | --- | --- | --- | --- | --- | --- | --- | --- | --- | --- | --- | --- | --- | --- | --- | --- | --- | --- | --- | --- | --- | --- | --- | --- | --- | | 0.5 0.21494 3.79631|  |  |  |  |  |  |  |  |  |  |  |  |  |  |  |  |  |  |  |  |  |  |  |  |  |  |  |  |  |  | | --- | --- | --- | --- | --- | --- | --- | --- | --- | --- | --- | --- | --- | --- | --- | --- | --- | --- | --- | --- | --- | --- | --- | --- | --- | --- | --- | --- | --- | --- | | 0.55 0.13867 1.39798|  |  |  |  |  |  |  |  |  |  |  |  |  |  |  |  |  |  |  |  |  |  |  |  |  |  |  | | --- | --- | --- | --- | --- | --- | --- | --- | --- | --- | --- | --- | --- | --- | --- | --- | --- | --- | --- | --- | --- | --- | --- | --- | --- | --- | --- | | 0.6 0.09145 0.50434|  |  |  |  |  |  |  |  |  |  |  |  |  |  |  |  |  |  |  |  |  |  |  |  | | --- | --- | --- | --- | --- | --- | --- | --- | --- | --- | --- | --- | --- | --- | --- | --- | --- | --- | --- | --- | --- | --- | --- | --- | | 0.65 0.06102 0.17417|  |  |  |  |  |  |  |  |  |  |  |  |  |  |  |  |  |  |  |  |  | | --- | --- | --- | --- | --- | --- | --- | --- | --- | --- | --- | --- | --- | --- | --- | --- | --- | --- | --- | --- | --- | | 0.7 0.04070 0.05591|  |  |  |  |  |  |  |  |  |  |  |  |  |  |  |  |  |  | | --- | --- | --- | --- | --- | --- | --- | --- | --- | --- | --- | --- | --- | --- | --- | --- | --- | --- | | 0.75 0.02673 0.01600|  |  |  |  |  |  |  |  |  |  |  |  |  |  |  | | --- | --- | --- | --- | --- | --- | --- | --- | --- | --- | --- | --- | --- | --- | --- | | 0.8 0.01691 0.00382|  |  |  |  |  |  |  |  |  |  |  |  | | --- | --- | --- | --- | --- | --- | --- | --- | --- | --- | --- | --- | | 0.85 0.00993 6.75E-4|  |  |  |  |  |  |  |  |  | | --- | --- | --- | --- | --- | --- | --- | --- | --- | | 0.9 0.00499 6.74E-5|  |  |  |  |  |  | | --- | --- | --- | --- | --- | --- | | 0.95 0.00168 1.63E-6|  |  |  | | --- | --- | --- | | 0.97 7.80E-4 1.16E-7 | | | | | | | | | | | | | | | | | | | | | | | | | | | | | | | | | | | | | | | | | | | | | | | | | | | | | | | | | | | |

CI values for actual experimental points:

Total Dose Fa CI Value | | || 5.5 0.492 0.28560|  |  |  |  |  |  |  |  |  |  |  |  |  |  |  | | --- | --- | --- | --- | --- | --- | --- | --- | --- | --- | --- | --- | --- | --- | --- | | 11.0 0.455 0.38654|  |  |  |  |  |  |  |  |  |  |  |  | | --- | --- | --- | --- | --- | --- | --- | --- | --- | --- | --- | --- | | 22.0 0.41 0.48884|  |  |  |  |  |  |  |  |  | | --- | --- | --- | --- | --- | --- | --- | --- | --- | | 44.0 0.347 0.52328|  |  |  |  |  |  | | --- | --- | --- | --- | --- | --- | | 88.0 0.34 0.97662|  |  |  | | --- | --- | --- | | 176.0 0.34 1.95324 | | | | | | | | | | | | | | | | | |

---

Combination Index Plot  


---

DRI Data for Drug Combo: 6+9 (6+9 [1:10])

| Fa Dose 6 Dose 9 DRI 6 DRI 9 | | | | |
| --- | --- | --- | --- | --- |
| 0.05 99227.3 5954.73 0.12377 7.43E-4|  |  |  |  |  |  |  |  |  |  |  |  |  |  |  |  |  |  |  |  |  |  |  |  |  |  |  |  |  |  |  |  |  |  |  |  |  |  |  |  |  |  |  |  |  |  |  |  |  |  |  |  |  |  |  |  |  |  |  |  |  |  |  |  |  |  |  |  |  |  |  |  |  |  |  |  |  |  |  |  |  |  |  |  |  |  |  |  |  |  |  |  |  |  |  | | --- | --- | --- | --- | --- | --- | --- | --- | --- | --- | --- | --- | --- | --- | --- | --- | --- | --- | --- | --- | --- | --- | --- | --- | --- | --- | --- | --- | --- | --- | --- | --- | --- | --- | --- | --- | --- | --- | --- | --- | --- | --- | --- | --- | --- | --- | --- | --- | --- | --- | --- | --- | --- | --- | --- | --- | --- | --- | --- | --- | --- | --- | --- | --- | --- | --- | --- | --- | --- | --- | --- | --- | --- | --- | --- | --- | --- | --- | --- | --- | --- | --- | --- | --- | --- | --- | --- | --- | --- | --- | --- | --- | --- | --- | --- | | 0.1 7054.68 1617.14 0.36307 0.00832|  |  |  |  |  |  |  |  |  |  |  |  |  |  |  |  |  |  |  |  |  |  |  |  |  |  |  |  |  |  |  |  |  |  |  |  |  |  |  |  |  |  |  |  |  |  |  |  |  |  |  |  |  |  |  |  |  |  |  |  |  |  |  |  |  |  |  |  |  |  |  |  |  |  |  |  |  |  |  |  |  |  |  |  |  |  |  |  |  |  | | --- | --- | --- | --- | --- | --- | --- | --- | --- | --- | --- | --- | --- | --- | --- | --- | --- | --- | --- | --- | --- | --- | --- | --- | --- | --- | --- | --- | --- | --- | --- | --- | --- | --- | --- | --- | --- | --- | --- | --- | --- | --- | --- | --- | --- | --- | --- | --- | --- | --- | --- | --- | --- | --- | --- | --- | --- | --- | --- | --- | --- | --- | --- | --- | --- | --- | --- | --- | --- | --- | --- | --- | --- | --- | --- | --- | --- | --- | --- | --- | --- | --- | --- | --- | --- | --- | --- | --- | --- | --- | | 0.15 1372.84 721.519 0.70688 0.03715|  |  |  |  |  |  |  |  |  |  |  |  |  |  |  |  |  |  |  |  |  |  |  |  |  |  |  |  |  |  |  |  |  |  |  |  |  |  |  |  |  |  |  |  |  |  |  |  |  |  |  |  |  |  |  |  |  |  |  |  |  |  |  |  |  |  |  |  |  |  |  |  |  |  |  |  |  |  |  |  |  |  |  |  |  | | --- | --- | --- | --- | --- | --- | --- | --- | --- | --- | --- | --- | --- | --- | --- | --- | --- | --- | --- | --- | --- | --- | --- | --- | --- | --- | --- | --- | --- | --- | --- | --- | --- | --- | --- | --- | --- | --- | --- | --- | --- | --- | --- | --- | --- | --- | --- | --- | --- | --- | --- | --- | --- | --- | --- | --- | --- | --- | --- | --- | --- | --- | --- | --- | --- | --- | --- | --- | --- | --- | --- | --- | --- | --- | --- | --- | --- | --- | --- | --- | --- | --- | --- | --- | --- | | 0.2 400.330 392.969 1.16736 0.11459|  |  |  |  |  |  |  |  |  |  |  |  |  |  |  |  |  |  |  |  |  |  |  |  |  |  |  |  |  |  |  |  |  |  |  |  |  |  |  |  |  |  |  |  |  |  |  |  |  |  |  |  |  |  |  |  |  |  |  |  |  |  |  |  |  |  |  |  |  |  |  |  |  |  |  |  |  |  |  |  | | --- | --- | --- | --- | --- | --- | --- | --- | --- | --- | --- | --- | --- | --- | --- | --- | --- | --- | --- | --- | --- | --- | --- | --- | --- | --- | --- | --- | --- | --- | --- | --- | --- | --- | --- | --- | --- | --- | --- | --- | --- | --- | --- | --- | --- | --- | --- | --- | --- | --- | --- | --- | --- | --- | --- | --- | --- | --- | --- | --- | --- | --- | --- | --- | --- | --- | --- | --- | --- | --- | --- | --- | --- | --- | --- | --- | --- | --- | --- | --- | | 0.25 144.668 237.904 1.76661 0.29052|  |  |  |  |  |  |  |  |  |  |  |  |  |  |  |  |  |  |  |  |  |  |  |  |  |  |  |  |  |  |  |  |  |  |  |  |  |  |  |  |  |  |  |  |  |  |  |  |  |  |  |  |  |  |  |  |  |  |  |  |  |  |  |  |  |  |  |  |  |  |  |  |  |  |  | | --- | --- | --- | --- | --- | --- | --- | --- | --- | --- | --- | --- | --- | --- | --- | --- | --- | --- | --- | --- | --- | --- | --- | --- | --- | --- | --- | --- | --- | --- | --- | --- | --- | --- | --- | --- | --- | --- | --- | --- | --- | --- | --- | --- | --- | --- | --- | --- | --- | --- | --- | --- | --- | --- | --- | --- | --- | --- | --- | --- | --- | --- | --- | --- | --- | --- | --- | --- | --- | --- | --- | --- | --- | --- | --- | | 0.3 59.4576 153.460 2.53707 0.65482|  |  |  |  |  |  |  |  |  |  |  |  |  |  |  |  |  |  |  |  |  |  |  |  |  |  |  |  |  |  |  |  |  |  |  |  |  |  |  |  |  |  |  |  |  |  |  |  |  |  |  |  |  |  |  |  |  |  |  |  |  |  |  |  |  |  |  |  |  |  | | --- | --- | --- | --- | --- | --- | --- | --- | --- | --- | --- | --- | --- | --- | --- | --- | --- | --- | --- | --- | --- | --- | --- | --- | --- | --- | --- | --- | --- | --- | --- | --- | --- | --- | --- | --- | --- | --- | --- | --- | --- | --- | --- | --- | --- | --- | --- | --- | --- | --- | --- | --- | --- | --- | --- | --- | --- | --- | --- | --- | --- | --- | --- | --- | --- | --- | --- | --- | --- | --- | | 0.35 26.5137 103.053 3.52455 1.36991|  |  |  |  |  |  |  |  |  |  |  |  |  |  |  |  |  |  |  |  |  |  |  |  |  |  |  |  |  |  |  |  |  |  |  |  |  |  |  |  |  |  |  |  |  |  |  |  |  |  |  |  |  |  |  |  |  |  |  |  |  |  |  |  |  | | --- | --- | --- | --- | --- | --- | --- | --- | --- | --- | --- | --- | --- | --- | --- | --- | --- | --- | --- | --- | --- | --- | --- | --- | --- | --- | --- | --- | --- | --- | --- | --- | --- | --- | --- | --- | --- | --- | --- | --- | --- | --- | --- | --- | --- | --- | --- | --- | --- | --- | --- | --- | --- | --- | --- | --- | --- | --- | --- | --- | --- | --- | --- | --- | --- | | 0.4 12.4536 70.9985 4.79390 2.73301|  |  |  |  |  |  |  |  |  |  |  |  |  |  |  |  |  |  |  |  |  |  |  |  |  |  |  |  |  |  |  |  |  |  |  |  |  |  |  |  |  |  |  |  |  |  |  |  |  |  |  |  |  |  |  |  |  |  |  |  | | --- | --- | --- | --- | --- | --- | --- | --- | --- | --- | --- | --- | --- | --- | --- | --- | --- | --- | --- | --- | --- | --- | --- | --- | --- | --- | --- | --- | --- | --- | --- | --- | --- | --- | --- | --- | --- | --- | --- | --- | --- | --- | --- | --- | --- | --- | --- | --- | --- | --- | --- | --- | --- | --- | --- | --- | --- | --- | --- | --- | | 0.45 6.03412 49.6695 6.43848 5.29980|  |  |  |  |  |  |  |  |  |  |  |  |  |  |  |  |  |  |  |  |  |  |  |  |  |  |  |  |  |  |  |  |  |  |  |  |  |  |  |  |  |  |  |  |  |  |  |  |  |  |  |  |  |  |  | | --- | --- | --- | --- | --- | --- | --- | --- | --- | --- | --- | --- | --- | --- | --- | --- | --- | --- | --- | --- | --- | --- | --- | --- | --- | --- | --- | --- | --- | --- | --- | --- | --- | --- | --- | --- | --- | --- | --- | --- | --- | --- | --- | --- | --- | --- | --- | --- | --- | --- | --- | --- | --- | --- | --- | | 0.5 2.96666 34.9989 8.59603 10.1411|  |  |  |  |  |  |  |  |  |  |  |  |  |  |  |  |  |  |  |  |  |  |  |  |  |  |  |  |  |  |  |  |  |  |  |  |  |  |  |  |  |  |  |  |  |  |  |  |  |  | | --- | --- | --- | --- | --- | --- | --- | --- | --- | --- | --- | --- | --- | --- | --- | --- | --- | --- | --- | --- | --- | --- | --- | --- | --- | --- | --- | --- | --- | --- | --- | --- | --- | --- | --- | --- | --- | --- | --- | --- | --- | --- | --- | --- | --- | --- | --- | --- | --- | --- | | 0.55 1.45855 24.6615 11.4766 19.4049|  |  |  |  |  |  |  |  |  |  |  |  |  |  |  |  |  |  |  |  |  |  |  |  |  |  |  |  |  |  |  |  |  |  |  |  |  |  |  |  |  |  |  |  |  | | --- | --- | --- | --- | --- | --- | --- | --- | --- | --- | --- | --- | --- | --- | --- | --- | --- | --- | --- | --- | --- | --- | --- | --- | --- | --- | --- | --- | --- | --- | --- | --- | --- | --- | --- | --- | --- | --- | --- | --- | --- | --- | --- | --- | --- | | 0.6 0.70670 17.2529 15.4137 37.6296|  |  |  |  |  |  |  |  |  |  |  |  |  |  |  |  |  |  |  |  |  |  |  |  |  |  |  |  |  |  |  |  |  |  |  |  |  |  |  |  | | --- | --- | --- | --- | --- | --- | --- | --- | --- | --- | --- | --- | --- | --- | --- | --- | --- | --- | --- | --- | --- | --- | --- | --- | --- | --- | --- | --- | --- | --- | --- | --- | --- | --- | --- | --- | --- | --- | --- | --- | | 0.65 0.33194 11.8864 20.9649 75.0721|  |  |  |  |  |  |  |  |  |  |  |  |  |  |  |  |  |  |  |  |  |  |  |  |  |  |  |  |  |  |  |  |  |  |  | | --- | --- | --- | --- | --- | --- | --- | --- | --- | --- | --- | --- | --- | --- | --- | --- | --- | --- | --- | --- | --- | --- | --- | --- | --- | --- | --- | --- | --- | --- | --- | --- | --- | --- | --- | | 0.7 0.14802 7.98203 29.1248 157.054|  |  |  |  |  |  |  |  |  |  |  |  |  |  |  |  |  |  |  |  |  |  |  |  |  |  |  |  |  |  | | --- | --- | --- | --- | --- | --- | --- | --- | --- | --- | --- | --- | --- | --- | --- | --- | --- | --- | --- | --- | --- | --- | --- | --- | --- | --- | --- | --- | --- | --- | | 0.75 0.06084 5.14884 41.8268 353.998|  |  |  |  |  |  |  |  |  |  |  |  |  |  |  |  |  |  |  |  |  |  |  |  |  | | --- | --- | --- | --- | --- | --- | --- | --- | --- | --- | --- | --- | --- | --- | --- | --- | --- | --- | --- | --- | --- | --- | --- | --- | --- | | 0.8 0.02198 3.11710 63.2985 897.488|  |  |  |  |  |  |  |  |  |  |  |  |  |  |  |  |  |  |  |  | | --- | --- | --- | --- | --- | --- | --- | --- | --- | --- | --- | --- | --- | --- | --- | --- | --- | --- | --- | --- | | 0.85 0.00641 1.69771 104.532 2768.20|  |  |  |  |  |  |  |  |  |  |  |  |  |  |  | | --- | --- | --- | --- | --- | --- | --- | --- | --- | --- | --- | --- | --- | --- | --- | | 0.9 0.00125 0.75747 203.522 12357.1|  |  |  |  |  |  |  |  |  |  | | --- | --- | --- | --- | --- | --- | --- | --- | --- | --- | | 0.95 8.87E-5 0.20571 597.003 138459.|  |  |  |  |  | | --- | --- | --- | --- | --- | | 0.97 1.35E-5 0.08137 1283.86 772674. | | | | | | | | | | | | | | | | | | | | | | | | | | | | | | | | | | | | | | | | | | | | | | | | | | | | | | | | | | | | | | | | | | | | | | | | | | | | | | | | | | | | | | | | | | | | | | | | | | | |

DRI values calculated at experimental points

| Fa Dose 6 Dose 9 DRI 6 DRI 9 | | | | |
| --- | --- | --- | --- | --- |
| 0.492 3.32232 37.0085 6.64465 7.40170|  |  |  |  |  |  |  |  |  |  |  |  |  |  |  |  |  |  |  |  |  |  |  |  |  | | --- | --- | --- | --- | --- | --- | --- | --- | --- | --- | --- | --- | --- | --- | --- | --- | --- | --- | --- | --- | --- | --- | --- | --- | --- | | 0.455 5.61826 47.9512 5.61826 4.79512|  |  |  |  |  |  |  |  |  |  |  |  |  |  |  |  |  |  |  |  | | --- | --- | --- | --- | --- | --- | --- | --- | --- | --- | --- | --- | --- | --- | --- | --- | --- | --- | --- | --- | | 0.41 10.7530 66.0401 5.37649 3.30200|  |  |  |  |  |  |  |  |  |  |  |  |  |  |  | | --- | --- | --- | --- | --- | --- | --- | --- | --- | --- | --- | --- | --- | --- | --- | | 0.347 27.7826 105.456 6.94565 2.63640|  |  |  |  |  |  |  |  |  |  | | --- | --- | --- | --- | --- | --- | --- | --- | --- | --- | | 0.34 31.0078 111.324 3.87598 1.39155|  |  |  |  |  | | --- | --- | --- | --- | --- | | 0.34 31.0078 111.324 1.93799 0.69578 | | | | | | | | | | | | | | | | | | | | | | | | | | | | | |

---

DRI Plot for Combo: 6+9 (6+9 [1:10])  


---

Isobologram for Combo: 6+9 (6+9 [1:10])  


---

Summary Table

|  |  |  |  |  |  |  |  |
| --- | --- | --- | --- | --- | --- | --- | --- |
| Experiment Name: CACO2|  |  |  |  |  |  | | --- | --- | --- | --- | --- | --- | | Date: |  |  |  |  | | --- | --- | --- | --- | | File Name: L:\Ô¬Ê¦ÐÖÊý¾Ý\20180523 coca2 CI\Caco2\Caco2.cse|  |  | | --- | --- | | Description  | | | | | | | |

|  |  |  |  |  |  |
| --- | --- | --- | --- | --- | --- |
| Drug: MK-2206 (6) [uM]|  |  |  |  | | --- | --- | --- | --- | | Drug: SHP099 (9) [uM]|  |  | | --- | --- | | Drug Combo: MK-2206+SHP099 (6+9) (6+9 [1:10]) | | | | | |

---

| Drug/Combo Dm m r | | | |
| --- | --- | --- | --- |
| 6 2.96666 -0.2826 -0.9449|  |  |  |  |  |  |  |  | | --- | --- | --- | --- | --- | --- | --- | --- | | 9 34.9989 -0.5732 -0.9593|  |  |  |  | | --- | --- | --- | --- | | 6+9 3.79631 -0.2009 -0.9518 | | | | | | | | | | | |

---

|  |  |  |  |  |  |  |  |  |  |
| --- | --- | --- | --- | --- | --- | --- | --- | --- | --- |
| CI values at:| Combo ED50 ED75 ED90 ED95 | | | | | | --- | --- | --- | --- | --- | | | | | |
| 6+9 0.21494 0.02673 0.00499 0.00168 | | | | |

---

Data for Fa = 0.5

| Drug/Combo CI value Dose 6 Dose 9 | | | |
| --- | --- | --- | --- |
| 6 2.96666|  |  |  |  |  |  |  |  | | --- | --- | --- | --- | --- | --- | --- | --- | | 9 34.9989|  |  |  |  | | --- | --- | --- | --- | | 6+9 0.21494 0.34512 3.45119 | | | | | | | | | | |

---

Data for Fa = 0.75

| Drug/Combo CI value Dose 6 Dose 9 | | | |
| --- | --- | --- | --- |
| 6 0.06084|  |  |  |  |  |  |  |  | | --- | --- | --- | --- | --- | --- | --- | --- | | 9 5.14884|  |  |  |  | | --- | --- | --- | --- | | 6+9 0.02673 0.00145 0.01454 | | | | | | | | | | |

---

Data for Fa = 0.9

| Drug/Combo CI value Dose 6 Dose 9 | | | |
| --- | --- | --- | --- |
| 6 0.00125|  |  |  |  |  |  |  |  | | --- | --- | --- | --- | --- | --- | --- | --- | | 9 0.75747|  |  |  |  | | --- | --- | --- | --- | | 6+9 0.00499 6.13E-6 6.13E-5 | | | | | | | | | | |

---

Data for Fa = 0.95

| Drug/Combo CI value Dose 6 Dose 9 | | | |
| --- | --- | --- | --- |
| 6 8.87E-5|  |  |  |  |  |  |  |  | | --- | --- | --- | --- | --- | --- | --- | --- | | 9 0.20571|  |  |  |  | | --- | --- | --- | --- | | 6+9 0.00168 1.49E-7 1.49E-6 | | | | | | | | | | |

---

Data for Fa = 0.97

| Drug/Combo CI value Dose 6 Dose 9 | | | |
| --- | --- | --- | --- |
| 6 1.35E-5|  |  |  |  |  |  |  |  | | --- | --- | --- | --- | --- | --- | --- | --- | | 9 0.08137|  |  |  |  | | --- | --- | --- | --- | | 6+9 7.80E-4 1.05E-8 1.05E-7 | | | | | | | | | | |
