# Supplementary material for: Therapeutic Suppression of FAK-AKT Signaling Overcomes Resistance to SHP2 Inhibition in Colorectal Carcinoma
Source: Front Pharmacol. 2021 Nov 1;12:739501. doi: 10.3389/fphar.2021.739501 (PMC8591248; doi:10.3389/fphar.2021.739501)
Supplement: Supplementary file 11 [file DataSheet6.ZIP › Figure3/Figure3B/Colo205/COLO205/COLO205-report/report.html]

CompuSyn Report


CompuSyn Report

|  |  |  |  |  |  |  |  |
| --- | --- | --- | --- | --- | --- | --- | --- |
| Experiment Name: COLO205|  |  |  |  |  |  | | --- | --- | --- | --- | --- | --- | | Date: |  |  |  |  | | --- | --- | --- | --- | | File Name: L:\20180520 205 CI\COLO205\COLO205.cse|  |  | | --- | --- | | Description  | | | | | | | |

|  |  |  |  |  |  |
| --- | --- | --- | --- | --- | --- |
| Drug: MK-2206 (6) [uM]|  |  |  |  | | --- | --- | --- | --- | | Drug: SHP099 (9) [uM]|  |  | | --- | --- | | Drug Combo: MK-2206+SHP099 (6+9) (6+9 [1:20]) | | | | | |

---

Data for Drug: 6 [uM]

| Dose Effect | |
| --- | --- |
| 0.25 0.65|  |  |  |  |  |  |  |  |  |  | | --- | --- | --- | --- | --- | --- | --- | --- | --- | --- | | 0.5 0.645|  |  |  |  |  |  |  |  | | --- | --- | --- | --- | --- | --- | --- | --- | | 1.0 0.579|  |  |  |  |  |  | | --- | --- | --- | --- | --- | --- | | 2.0 0.4375|  |  |  |  | | --- | --- | --- | --- | | 4.0 0.301|  |  | | --- | --- | | 8.0 0.1715 | | | | | | | | | | | |

6 data points entered.

|  |  |  |  |  |  |  |  |  |  |
| --- | --- | --- | --- | --- | --- | --- | --- | --- | --- |
| X-int: 0.02495|  |  |  |  |  |  |  |  | | --- | --- | --- | --- | --- | --- | --- | --- | | Y-int: 0.01631 +/- 0.04954|  |  |  |  |  |  | | --- | --- | --- | --- | --- | --- | | m: -0.6537 +/- 0.09248|  |  |  |  | | --- | --- | --- | --- | | Dm: 1.05913|  |  | | --- | --- | | r: -0.9622 | | | | | | | | | |

---

Data for Drug: 9 [uM]

| Dose Effect | |
| --- | --- |
| 5.0 0.706|  |  |  |  |  |  |  |  |  |  | | --- | --- | --- | --- | --- | --- | --- | --- | --- | --- | | 10.0 0.674|  |  |  |  |  |  |  |  | | --- | --- | --- | --- | --- | --- | --- | --- | | 20.0 0.539|  |  |  |  |  |  | | --- | --- | --- | --- | --- | --- | | 40.0 0.3345|  |  |  |  | | --- | --- | --- | --- | | 80.0 0.176|  |  | | --- | --- | | 160.0 0.1705 | | | | | | | | | | | |

6 data points entered.

|  |  |  |  |  |  |  |  |  |  |
| --- | --- | --- | --- | --- | --- | --- | --- | --- | --- |
| X-int: 1.27062|  |  |  |  |  |  |  |  | | --- | --- | --- | --- | --- | --- | --- | --- | | Y-int: 1.04461 +/- 0.14347|  |  |  |  |  |  | | --- | --- | --- | --- | --- | --- | | m: -0.8221 +/- 0.09317|  |  |  |  | | --- | --- | --- | --- | | Dm: 18.6474|  |  | | --- | --- | | r: -0.9753 | | | | | | | | | |

---

Data for Drug Combo: 6+9 (6+9 [1:20])

| Dose A Effect | |
| --- | --- |
| 0.25+ 0.557|  |  |  |  |  |  |  |  |  |  | | --- | --- | --- | --- | --- | --- | --- | --- | --- | --- | | 0.5+ 0.4995|  |  |  |  |  |  |  |  | | --- | --- | --- | --- | --- | --- | --- | --- | | 1.0+ 0.3065|  |  |  |  |  |  | | --- | --- | --- | --- | --- | --- | | 2.0+ 0.2215|  |  |  |  | | --- | --- | --- | --- | | 4.0+ 0.168|  |  | | --- | --- | | 8.0+ 0.1678 | | | | | | | | | | | |

6 data points entered.

|  |  |  |  |  |  |  |  |  |  |
| --- | --- | --- | --- | --- | --- | --- | --- | --- | --- |
| X-int: 0.85658|  |  |  |  |  |  |  |  | | --- | --- | --- | --- | --- | --- | --- | --- | | Y-int: 0.50792 +/- 0.12617|  |  |  |  |  |  | | --- | --- | --- | --- | --- | --- | | m: -0.5930 +/- 0.08089|  |  |  |  | | --- | --- | --- | --- | | Dm: 7.18749|  |  | | --- | --- | | r: -0.9647 | | | | | | | | | |

---

Dose-Effect Curve  


---

Median-Effect Plot  


---

CI Data for Drug Combo: 6+9 (6+9 [1:20])

| Fa CI Value Total Dose | | |
| --- | --- | --- |
| 0.05 1.97779 1030.64|  |  |  |  |  |  |  |  |  |  |  |  |  |  |  |  |  |  |  |  |  |  |  |  |  |  |  |  |  |  |  |  |  |  |  |  |  |  |  |  |  |  |  |  |  |  |  |  |  |  |  |  |  |  |  |  |  | | --- | --- | --- | --- | --- | --- | --- | --- | --- | --- | --- | --- | --- | --- | --- | --- | --- | --- | --- | --- | --- | --- | --- | --- | --- | --- | --- | --- | --- | --- | --- | --- | --- | --- | --- | --- | --- | --- | --- | --- | --- | --- | --- | --- | --- | --- | --- | --- | --- | --- | --- | --- | --- | --- | --- | --- | --- | | 0.1 1.48718 292.309|  |  |  |  |  |  |  |  |  |  |  |  |  |  |  |  |  |  |  |  |  |  |  |  |  |  |  |  |  |  |  |  |  |  |  |  |  |  |  |  |  |  |  |  |  |  |  |  |  |  |  |  |  |  | | --- | --- | --- | --- | --- | --- | --- | --- | --- | --- | --- | --- | --- | --- | --- | --- | --- | --- | --- | --- | --- | --- | --- | --- | --- | --- | --- | --- | --- | --- | --- | --- | --- | --- | --- | --- | --- | --- | --- | --- | --- | --- | --- | --- | --- | --- | --- | --- | --- | --- | --- | --- | --- | --- | | 0.15 1.25375 133.972|  |  |  |  |  |  |  |  |  |  |  |  |  |  |  |  |  |  |  |  |  |  |  |  |  |  |  |  |  |  |  |  |  |  |  |  |  |  |  |  |  |  |  |  |  |  |  |  |  |  |  | | --- | --- | --- | --- | --- | --- | --- | --- | --- | --- | --- | --- | --- | --- | --- | --- | --- | --- | --- | --- | --- | --- | --- | --- | --- | --- | --- | --- | --- | --- | --- | --- | --- | --- | --- | --- | --- | --- | --- | --- | --- | --- | --- | --- | --- | --- | --- | --- | --- | --- | --- | | 0.2 1.10592 74.4578|  |  |  |  |  |  |  |  |  |  |  |  |  |  |  |  |  |  |  |  |  |  |  |  |  |  |  |  |  |  |  |  |  |  |  |  |  |  |  |  |  |  |  |  |  |  |  |  | | --- | --- | --- | --- | --- | --- | --- | --- | --- | --- | --- | --- | --- | --- | --- | --- | --- | --- | --- | --- | --- | --- | --- | --- | --- | --- | --- | --- | --- | --- | --- | --- | --- | --- | --- | --- | --- | --- | --- | --- | --- | --- | --- | --- | --- | --- | --- | --- | | 0.25 0.99912 45.8363|  |  |  |  |  |  |  |  |  |  |  |  |  |  |  |  |  |  |  |  |  |  |  |  |  |  |  |  |  |  |  |  |  |  |  |  |  |  |  |  |  |  |  |  |  | | --- | --- | --- | --- | --- | --- | --- | --- | --- | --- | --- | --- | --- | --- | --- | --- | --- | --- | --- | --- | --- | --- | --- | --- | --- | --- | --- | --- | --- | --- | --- | --- | --- | --- | --- | --- | --- | --- | --- | --- | --- | --- | --- | --- | --- | | 0.3 0.91574 30.0016|  |  |  |  |  |  |  |  |  |  |  |  |  |  |  |  |  |  |  |  |  |  |  |  |  |  |  |  |  |  |  |  |  |  |  |  |  |  |  |  |  |  | | --- | --- | --- | --- | --- | --- | --- | --- | --- | --- | --- | --- | --- | --- | --- | --- | --- | --- | --- | --- | --- | --- | --- | --- | --- | --- | --- | --- | --- | --- | --- | --- | --- | --- | --- | --- | --- | --- | --- | --- | --- | --- | | 0.35 0.84715 20.4158|  |  |  |  |  |  |  |  |  |  |  |  |  |  |  |  |  |  |  |  |  |  |  |  |  |  |  |  |  |  |  |  |  |  |  |  |  |  |  | | --- | --- | --- | --- | --- | --- | --- | --- | --- | --- | --- | --- | --- | --- | --- | --- | --- | --- | --- | --- | --- | --- | --- | --- | --- | --- | --- | --- | --- | --- | --- | --- | --- | --- | --- | --- | --- | --- | --- | | 0.4 0.78852 14.2411|  |  |  |  |  |  |  |  |  |  |  |  |  |  |  |  |  |  |  |  |  |  |  |  |  |  |  |  |  |  |  |  |  |  |  |  | | --- | --- | --- | --- | --- | --- | --- | --- | --- | --- | --- | --- | --- | --- | --- | --- | --- | --- | --- | --- | --- | --- | --- | --- | --- | --- | --- | --- | --- | --- | --- | --- | --- | --- | --- | --- | | 0.45 0.73688 10.0821|  |  |  |  |  |  |  |  |  |  |  |  |  |  |  |  |  |  |  |  |  |  |  |  |  |  |  |  |  |  |  |  |  | | --- | --- | --- | --- | --- | --- | --- | --- | --- | --- | --- | --- | --- | --- | --- | --- | --- | --- | --- | --- | --- | --- | --- | --- | --- | --- | --- | --- | --- | --- | --- | --- | --- | | 0.5 0.69024 7.18749|  |  |  |  |  |  |  |  |  |  |  |  |  |  |  |  |  |  |  |  |  |  |  |  |  |  |  |  |  |  | | --- | --- | --- | --- | --- | --- | --- | --- | --- | --- | --- | --- | --- | --- | --- | --- | --- | --- | --- | --- | --- | --- | --- | --- | --- | --- | --- | --- | --- | --- | | 0.55 0.64719 5.12395|  |  |  |  |  |  |  |  |  |  |  |  |  |  |  |  |  |  |  |  |  |  |  |  |  |  |  | | --- | --- | --- | --- | --- | --- | --- | --- | --- | --- | --- | --- | --- | --- | --- | --- | --- | --- | --- | --- | --- | --- | --- | --- | --- | --- | --- | | 0.6 0.60664 3.62754|  |  |  |  |  |  |  |  |  |  |  |  |  |  |  |  |  |  |  |  |  |  |  |  | | --- | --- | --- | --- | --- | --- | --- | --- | --- | --- | --- | --- | --- | --- | --- | --- | --- | --- | --- | --- | --- | --- | --- | --- | | 0.65 0.56768 2.53040|  |  |  |  |  |  |  |  |  |  |  |  |  |  |  |  |  |  |  |  |  | | --- | --- | --- | --- | --- | --- | --- | --- | --- | --- | --- | --- | --- | --- | --- | --- | --- | --- | --- | --- | --- | | 0.7 0.52946 1.72191|  |  |  |  |  |  |  |  |  |  |  |  |  |  |  |  |  |  | | --- | --- | --- | --- | --- | --- | --- | --- | --- | --- | --- | --- | --- | --- | --- | --- | --- | --- | | 0.75 0.49106 1.12706|  |  |  |  |  |  |  |  |  |  |  |  |  |  |  | | --- | --- | --- | --- | --- | --- | --- | --- | --- | --- | --- | --- | --- | --- | --- | | 0.8 0.45137 0.69382|  |  |  |  |  |  |  |  |  |  |  |  | | --- | --- | --- | --- | --- | --- | --- | --- | --- | --- | --- | --- | | 0.85 0.40866 0.38560|  |  |  |  |  |  |  |  |  | | --- | --- | --- | --- | --- | --- | --- | --- | --- | | 0.9 0.35969 0.17673|  |  |  |  |  |  | | --- | --- | --- | --- | --- | --- | | 0.95 0.29568 0.05012|  |  |  | | --- | --- | --- | | 0.97 0.25905 0.02045 | | | | | | | | | | | | | | | | | | | | | | | | | | | | | | | | | | | | | | | | | | | | | | | | | | | | | | | | | | | |

CI values for actual experimental points:

Total Dose Fa CI Value | | || 5.25 0.557 0.68932|  |  |  |  |  |  |  |  |  |  |  |  |  |  |  | | --- | --- | --- | --- | --- | --- | --- | --- | --- | --- | --- | --- | --- | --- | --- | | 10.5 0.4995 1.00561|  |  |  |  |  |  |  |  |  |  |  |  | | --- | --- | --- | --- | --- | --- | --- | --- | --- | --- | --- | --- | | 21.0 0.3065 0.66802|  |  |  |  |  |  |  |  |  | | --- | --- | --- | --- | --- | --- | --- | --- | --- | | 42.0 0.2215 0.74108|  |  |  |  |  |  | | --- | --- | --- | --- | --- | --- | | 84.0 0.168 0.93959|  |  |  | | --- | --- | --- | | 168.0 0.1678 1.87562 | | | | | | | | | | | | | | | | | |

---

Combination Index Plot  


---

DRI Data for Drug Combo: 6+9 (6+9 [1:20])

| Fa Dose 6 Dose 9 DRI 6 DRI 9 | | | | |
| --- | --- | --- | --- | --- |
| 0.05 95.7337 669.948 1.95064 0.68253|  |  |  |  |  |  |  |  |  |  |  |  |  |  |  |  |  |  |  |  |  |  |  |  |  |  |  |  |  |  |  |  |  |  |  |  |  |  |  |  |  |  |  |  |  |  |  |  |  |  |  |  |  |  |  |  |  |  |  |  |  |  |  |  |  |  |  |  |  |  |  |  |  |  |  |  |  |  |  |  |  |  |  |  |  |  |  |  |  |  |  |  |  |  |  | | --- | --- | --- | --- | --- | --- | --- | --- | --- | --- | --- | --- | --- | --- | --- | --- | --- | --- | --- | --- | --- | --- | --- | --- | --- | --- | --- | --- | --- | --- | --- | --- | --- | --- | --- | --- | --- | --- | --- | --- | --- | --- | --- | --- | --- | --- | --- | --- | --- | --- | --- | --- | --- | --- | --- | --- | --- | --- | --- | --- | --- | --- | --- | --- | --- | --- | --- | --- | --- | --- | --- | --- | --- | --- | --- | --- | --- | --- | --- | --- | --- | --- | --- | --- | --- | --- | --- | --- | --- | --- | --- | --- | --- | --- | --- | | 0.1 30.5252 269.972 2.19299 0.96976|  |  |  |  |  |  |  |  |  |  |  |  |  |  |  |  |  |  |  |  |  |  |  |  |  |  |  |  |  |  |  |  |  |  |  |  |  |  |  |  |  |  |  |  |  |  |  |  |  |  |  |  |  |  |  |  |  |  |  |  |  |  |  |  |  |  |  |  |  |  |  |  |  |  |  |  |  |  |  |  |  |  |  |  |  |  |  |  |  |  | | --- | --- | --- | --- | --- | --- | --- | --- | --- | --- | --- | --- | --- | --- | --- | --- | --- | --- | --- | --- | --- | --- | --- | --- | --- | --- | --- | --- | --- | --- | --- | --- | --- | --- | --- | --- | --- | --- | --- | --- | --- | --- | --- | --- | --- | --- | --- | --- | --- | --- | --- | --- | --- | --- | --- | --- | --- | --- | --- | --- | --- | --- | --- | --- | --- | --- | --- | --- | --- | --- | --- | --- | --- | --- | --- | --- | --- | --- | --- | --- | --- | --- | --- | --- | --- | --- | --- | --- | --- | --- | | 0.15 15.0424 153.792 2.35789 1.20534|  |  |  |  |  |  |  |  |  |  |  |  |  |  |  |  |  |  |  |  |  |  |  |  |  |  |  |  |  |  |  |  |  |  |  |  |  |  |  |  |  |  |  |  |  |  |  |  |  |  |  |  |  |  |  |  |  |  |  |  |  |  |  |  |  |  |  |  |  |  |  |  |  |  |  |  |  |  |  |  |  |  |  |  |  | | --- | --- | --- | --- | --- | --- | --- | --- | --- | --- | --- | --- | --- | --- | --- | --- | --- | --- | --- | --- | --- | --- | --- | --- | --- | --- | --- | --- | --- | --- | --- | --- | --- | --- | --- | --- | --- | --- | --- | --- | --- | --- | --- | --- | --- | --- | --- | --- | --- | --- | --- | --- | --- | --- | --- | --- | --- | --- | --- | --- | --- | --- | --- | --- | --- | --- | --- | --- | --- | --- | --- | --- | --- | --- | --- | --- | --- | --- | --- | --- | --- | --- | --- | --- | --- | | 0.2 8.82924 100.679 2.49019 1.41977|  |  |  |  |  |  |  |  |  |  |  |  |  |  |  |  |  |  |  |  |  |  |  |  |  |  |  |  |  |  |  |  |  |  |  |  |  |  |  |  |  |  |  |  |  |  |  |  |  |  |  |  |  |  |  |  |  |  |  |  |  |  |  |  |  |  |  |  |  |  |  |  |  |  |  |  |  |  |  |  | | --- | --- | --- | --- | --- | --- | --- | --- | --- | --- | --- | --- | --- | --- | --- | --- | --- | --- | --- | --- | --- | --- | --- | --- | --- | --- | --- | --- | --- | --- | --- | --- | --- | --- | --- | --- | --- | --- | --- | --- | --- | --- | --- | --- | --- | --- | --- | --- | --- | --- | --- | --- | --- | --- | --- | --- | --- | --- | --- | --- | --- | --- | --- | --- | --- | --- | --- | --- | --- | --- | --- | --- | --- | --- | --- | --- | --- | --- | --- | --- | | 0.25 5.68596 70.9526 2.60503 1.62535|  |  |  |  |  |  |  |  |  |  |  |  |  |  |  |  |  |  |  |  |  |  |  |  |  |  |  |  |  |  |  |  |  |  |  |  |  |  |  |  |  |  |  |  |  |  |  |  |  |  |  |  |  |  |  |  |  |  |  |  |  |  |  |  |  |  |  |  |  |  |  |  |  |  |  | | --- | --- | --- | --- | --- | --- | --- | --- | --- | --- | --- | --- | --- | --- | --- | --- | --- | --- | --- | --- | --- | --- | --- | --- | --- | --- | --- | --- | --- | --- | --- | --- | --- | --- | --- | --- | --- | --- | --- | --- | --- | --- | --- | --- | --- | --- | --- | --- | --- | --- | --- | --- | --- | --- | --- | --- | --- | --- | --- | --- | --- | --- | --- | --- | --- | --- | --- | --- | --- | --- | --- | --- | --- | --- | --- | | 0.3 3.87119 52.2648 2.70969 1.82917|  |  |  |  |  |  |  |  |  |  |  |  |  |  |  |  |  |  |  |  |  |  |  |  |  |  |  |  |  |  |  |  |  |  |  |  |  |  |  |  |  |  |  |  |  |  |  |  |  |  |  |  |  |  |  |  |  |  |  |  |  |  |  |  |  |  |  |  |  |  | | --- | --- | --- | --- | --- | --- | --- | --- | --- | --- | --- | --- | --- | --- | --- | --- | --- | --- | --- | --- | --- | --- | --- | --- | --- | --- | --- | --- | --- | --- | --- | --- | --- | --- | --- | --- | --- | --- | --- | --- | --- | --- | --- | --- | --- | --- | --- | --- | --- | --- | --- | --- | --- | --- | --- | --- | --- | --- | --- | --- | --- | --- | --- | --- | --- | --- | --- | --- | --- | --- | | 0.35 2.73025 39.5940 2.80838 2.03635|  |  |  |  |  |  |  |  |  |  |  |  |  |  |  |  |  |  |  |  |  |  |  |  |  |  |  |  |  |  |  |  |  |  |  |  |  |  |  |  |  |  |  |  |  |  |  |  |  |  |  |  |  |  |  |  |  |  |  |  |  |  |  |  |  | | --- | --- | --- | --- | --- | --- | --- | --- | --- | --- | --- | --- | --- | --- | --- | --- | --- | --- | --- | --- | --- | --- | --- | --- | --- | --- | --- | --- | --- | --- | --- | --- | --- | --- | --- | --- | --- | --- | --- | --- | --- | --- | --- | --- | --- | --- | --- | --- | --- | --- | --- | --- | --- | --- | --- | --- | --- | --- | --- | --- | --- | --- | --- | --- | --- | | 0.4 1.96932 30.5357 2.90397 2.25140|  |  |  |  |  |  |  |  |  |  |  |  |  |  |  |  |  |  |  |  |  |  |  |  |  |  |  |  |  |  |  |  |  |  |  |  |  |  |  |  |  |  |  |  |  |  |  |  |  |  |  |  |  |  |  |  |  |  |  |  | | --- | --- | --- | --- | --- | --- | --- | --- | --- | --- | --- | --- | --- | --- | --- | --- | --- | --- | --- | --- | --- | --- | --- | --- | --- | --- | --- | --- | --- | --- | --- | --- | --- | --- | --- | --- | --- | --- | --- | --- | --- | --- | --- | --- | --- | --- | --- | --- | --- | --- | --- | --- | --- | --- | --- | --- | --- | --- | --- | --- | | 0.45 1.43967 23.8025 2.99869 2.47892|  |  |  |  |  |  |  |  |  |  |  |  |  |  |  |  |  |  |  |  |  |  |  |  |  |  |  |  |  |  |  |  |  |  |  |  |  |  |  |  |  |  |  |  |  |  |  |  |  |  |  |  |  |  |  | | --- | --- | --- | --- | --- | --- | --- | --- | --- | --- | --- | --- | --- | --- | --- | --- | --- | --- | --- | --- | --- | --- | --- | --- | --- | --- | --- | --- | --- | --- | --- | --- | --- | --- | --- | --- | --- | --- | --- | --- | --- | --- | --- | --- | --- | --- | --- | --- | --- | --- | --- | --- | --- | --- | --- | | 0.5 1.05913 18.6474 3.09450 2.72414|  |  |  |  |  |  |  |  |  |  |  |  |  |  |  |  |  |  |  |  |  |  |  |  |  |  |  |  |  |  |  |  |  |  |  |  |  |  |  |  |  |  |  |  |  |  |  |  |  |  | | --- | --- | --- | --- | --- | --- | --- | --- | --- | --- | --- | --- | --- | --- | --- | --- | --- | --- | --- | --- | --- | --- | --- | --- | --- | --- | --- | --- | --- | --- | --- | --- | --- | --- | --- | --- | --- | --- | --- | --- | --- | --- | --- | --- | --- | --- | --- | --- | --- | --- | | 0.55 0.77918 14.6087 3.19337 2.99362|  |  |  |  |  |  |  |  |  |  |  |  |  |  |  |  |  |  |  |  |  |  |  |  |  |  |  |  |  |  |  |  |  |  |  |  |  |  |  |  |  |  |  |  |  | | --- | --- | --- | --- | --- | --- | --- | --- | --- | --- | --- | --- | --- | --- | --- | --- | --- | --- | --- | --- | --- | --- | --- | --- | --- | --- | --- | --- | --- | --- | --- | --- | --- | --- | --- | --- | --- | --- | --- | --- | --- | --- | --- | --- | --- | | 0.6 0.56961 11.3875 3.29753 3.29613|  |  |  |  |  |  |  |  |  |  |  |  |  |  |  |  |  |  |  |  |  |  |  |  |  |  |  |  |  |  |  |  |  |  |  |  |  |  |  |  | | --- | --- | --- | --- | --- | --- | --- | --- | --- | --- | --- | --- | --- | --- | --- | --- | --- | --- | --- | --- | --- | --- | --- | --- | --- | --- | --- | --- | --- | --- | --- | --- | --- | --- | --- | --- | --- | --- | --- | --- | | 0.65 0.41086 8.78223 3.40978 3.64423|  |  |  |  |  |  |  |  |  |  |  |  |  |  |  |  |  |  |  |  |  |  |  |  |  |  |  |  |  |  |  |  |  |  |  | | --- | --- | --- | --- | --- | --- | --- | --- | --- | --- | --- | --- | --- | --- | --- | --- | --- | --- | --- | --- | --- | --- | --- | --- | --- | --- | --- | --- | --- | --- | --- | --- | --- | --- | --- | | 0.7 0.28977 6.65312 3.53397 4.05700|  |  |  |  |  |  |  |  |  |  |  |  |  |  |  |  |  |  |  |  |  |  |  |  |  |  |  |  |  |  | | --- | --- | --- | --- | --- | --- | --- | --- | --- | --- | --- | --- | --- | --- | --- | --- | --- | --- | --- | --- | --- | --- | --- | --- | --- | --- | --- | --- | --- | --- | | 0.75 0.19729 4.90080 3.67594 4.56574|  |  |  |  |  |  |  |  |  |  |  |  |  |  |  |  |  |  |  |  |  |  |  |  |  | | --- | --- | --- | --- | --- | --- | --- | --- | --- | --- | --- | --- | --- | --- | --- | --- | --- | --- | --- | --- | --- | --- | --- | --- | --- | | 0.8 0.12705 3.45379 3.84547 5.22686|  |  |  |  |  |  |  |  |  |  |  |  |  |  |  |  |  |  |  |  | | --- | --- | --- | --- | --- | --- | --- | --- | --- | --- | --- | --- | --- | --- | --- | --- | --- | --- | --- | --- | | 0.85 0.07457 2.26100 4.06123 6.15670|  |  |  |  |  |  |  |  |  |  |  |  |  |  |  | | --- | --- | --- | --- | --- | --- | --- | --- | --- | --- | --- | --- | --- | --- | --- | | 0.9 0.03675 1.28800 4.36663 7.65230|  |  |  |  |  |  |  |  |  |  | | --- | --- | --- | --- | --- | --- | --- | --- | --- | --- | | 0.95 0.01172 0.51903 4.90914 10.8727|  |  |  |  |  | | --- | --- | --- | --- | --- | | 0.97 0.00520 0.27186 5.33572 13.9596 | | | | | | | | | | | | | | | | | | | | | | | | | | | | | | | | | | | | | | | | | | | | | | | | | | | | | | | | | | | | | | | | | | | | | | | | | | | | | | | | | | | | | | | | | | | | | | | | | | | |

DRI values calculated at experimental points

| Fa Dose 6 Dose 9 DRI 6 DRI 9 | | | | |
| --- | --- | --- | --- | --- |
| 0.557 0.74614 14.1140 2.98454 2.82279|  |  |  |  |  |  |  |  |  |  |  |  |  |  |  |  |  |  |  |  |  |  |  |  |  | | --- | --- | --- | --- | --- | --- | --- | --- | --- | --- | --- | --- | --- | --- | --- | --- | --- | --- | --- | --- | --- | --- | --- | --- | --- | | 0.4995 1.06237 18.6928 2.12475 1.86928|  |  |  |  |  |  |  |  |  |  |  |  |  |  |  |  |  |  |  |  | | --- | --- | --- | --- | --- | --- | --- | --- | --- | --- | --- | --- | --- | --- | --- | --- | --- | --- | --- | --- | | 0.3065 3.69323 50.3452 3.69323 2.51726|  |  |  |  |  |  |  |  |  |  |  |  |  |  |  | | --- | --- | --- | --- | --- | --- | --- | --- | --- | --- | --- | --- | --- | --- | --- | | 0.2215 7.24422 86.0220 3.62211 2.15055|  |  |  |  |  |  |  |  |  |  | | --- | --- | --- | --- | --- | --- | --- | --- | --- | --- | | 0.168 12.2408 130.545 3.06020 1.63181|  |  |  |  |  | | --- | --- | --- | --- | --- | | 0.1678 12.2676 130.773 1.53345 0.81733 | | | | | | | | | | | | | | | | | | | | | | | | | | | | | |

---

DRI Plot for Combo: 6+9 (6+9 [1:20])  


---

Isobologram for Combo: 6+9 (6+9 [1:20])  


---

Summary Table

|  |  |  |  |  |  |  |  |
| --- | --- | --- | --- | --- | --- | --- | --- |
| Experiment Name: COLO205|  |  |  |  |  |  | | --- | --- | --- | --- | --- | --- | | Date: |  |  |  |  | | --- | --- | --- | --- | | File Name: L:\20180520 205 CI\COLO205\COLO205.cse|  |  | | --- | --- | | Description  | | | | | | | |

|  |  |  |  |  |  |
| --- | --- | --- | --- | --- | --- |
| Drug: MK-2206 (6) [uM]|  |  |  |  | | --- | --- | --- | --- | | Drug: SHP099 (9) [uM]|  |  | | --- | --- | | Drug Combo: MK-2206+SHP099 (6+9) (6+9 [1:20]) | | | | | |

---

| Drug/Combo Dm m r | | | |
| --- | --- | --- | --- |
| 6 1.05913 -0.6537 -0.9622|  |  |  |  |  |  |  |  | | --- | --- | --- | --- | --- | --- | --- | --- | | 9 18.6474 -0.8221 -0.9753|  |  |  |  | | --- | --- | --- | --- | | 6+9 7.18749 -0.5930 -0.9647 | | | | | | | | | | | |

---

|  |  |  |  |  |  |  |  |  |  |
| --- | --- | --- | --- | --- | --- | --- | --- | --- | --- |
| CI values at:| Combo ED50 ED75 ED90 ED95 | | | | | | --- | --- | --- | --- | --- | | | | | |
| 6+9 0.69024 0.49106 0.35969 0.29568 | | | | |

---

Data for Fa = 0.5

| Drug/Combo CI value Dose 6 Dose 9 | | | |
| --- | --- | --- | --- |
| 6 1.05913|  |  |  |  |  |  |  |  | | --- | --- | --- | --- | --- | --- | --- | --- | | 9 18.6474|  |  |  |  | | --- | --- | --- | --- | | 6+9 0.69024 0.34226 6.84523 | | | | | | | | | | |

---

Data for Fa = 0.75

| Drug/Combo CI value Dose 6 Dose 9 | | | |
| --- | --- | --- | --- |
| 6 0.19729|  |  |  |  |  |  |  |  | | --- | --- | --- | --- | --- | --- | --- | --- | | 9 4.90080|  |  |  |  | | --- | --- | --- | --- | | 6+9 0.49106 0.05367 1.07339 | | | | | | | | | | |

---

Data for Fa = 0.9

| Drug/Combo CI value Dose 6 Dose 9 | | | |
| --- | --- | --- | --- |
| 6 0.03675|  |  |  |  |  |  |  |  | | --- | --- | --- | --- | --- | --- | --- | --- | | 9 1.28800|  |  |  |  | | --- | --- | --- | --- | | 6+9 0.35969 0.00842 0.16832 | | | | | | | | | | |

---

Data for Fa = 0.95

| Drug/Combo CI value Dose 6 Dose 9 | | | |
| --- | --- | --- | --- |
| 6 0.01172|  |  |  |  |  |  |  |  | | --- | --- | --- | --- | --- | --- | --- | --- | | 9 0.51903|  |  |  |  | | --- | --- | --- | --- | | 6+9 0.29568 0.00239 0.04774 | | | | | | | | | | |

---

Data for Fa = 0.97

| Drug/Combo CI value Dose 6 Dose 9 | | | |
| --- | --- | --- | --- |
| 6 0.00520|  |  |  |  |  |  |  |  | | --- | --- | --- | --- | --- | --- | --- | --- | | 9 0.27186|  |  |  |  | | --- | --- | --- | --- | | 6+9 0.25905 9.74E-4 0.01947 | | | | | | | | | | |
