# Supplementary material for: Therapeutic Suppression of FAK-AKT Signaling Overcomes Resistance to SHP2 Inhibition in Colorectal Carcinoma
Source: Front Pharmacol. 2021 Nov 1;12:739501. doi: 10.3389/fphar.2021.739501 (PMC8591248; doi:10.3389/fphar.2021.739501)
Supplement: Supplementary file 11 [file DataSheet6.ZIP › Figure3/Figure3B/RKO/RKO-report/report.html]

CompuSyn Report


CompuSyn Report

|  |  |  |  |  |  |  |  |
| --- | --- | --- | --- | --- | --- | --- | --- |
| Experiment Name: RKO|  |  |  |  |  |  | | --- | --- | --- | --- | --- | --- | | Date: |  |  |  |  | | --- | --- | --- | --- | | File Name: K:\20180502 CI\20180510\RKO\RKO.cse|  |  | | --- | --- | | Description  | | | | | | | |

|  |  |  |  |  |  |
| --- | --- | --- | --- | --- | --- |
| Drug: MK-2206 (6) [uM]|  |  |  |  | | --- | --- | --- | --- | | Drug: SHP099 (9) [uM]|  |  | | --- | --- | | Drug Combo: MK-2206+SHP099 (6+9) (6+9 [1:5]) | | | | | |

---

Data for Drug: 6 [uM]

| Dose Effect | |
| --- | --- |
| 0.8 0.381|  |  |  |  |  |  |  |  |  |  | | --- | --- | --- | --- | --- | --- | --- | --- | --- | --- | | 1.6 0.3585|  |  |  |  |  |  |  |  | | --- | --- | --- | --- | --- | --- | --- | --- | | 3.2 0.3285|  |  |  |  |  |  | | --- | --- | --- | --- | --- | --- | | 6.4 0.3165|  |  |  |  | | --- | --- | --- | --- | | 12.8 0.2135|  |  | | --- | --- | | 25.6 0.1955 | | | | | | | | | | | |

6 data points entered.

|  |  |  |  |  |  |  |  |  |  |
| --- | --- | --- | --- | --- | --- | --- | --- | --- | --- |
| X-int: -0.6920|  |  |  |  |  |  |  |  | | --- | --- | --- | --- | --- | --- | --- | --- | | Y-int: -0.1959 +/- 0.03955|  |  |  |  |  |  | | --- | --- | --- | --- | --- | --- | | m: -0.2831 +/- 0.04746|  |  |  |  | | --- | --- | --- | --- | | Dm: 0.20326|  |  | | --- | --- | | r: -0.9481 | | | | | | | | | |

---

Data for Drug: 9 [uM]

| Dose Effect | |
| --- | --- |
| 4.0 0.557|  |  |  |  |  |  |  |  |  |  | | --- | --- | --- | --- | --- | --- | --- | --- | --- | --- | | 8.0 0.511|  |  |  |  |  |  |  |  | | --- | --- | --- | --- | --- | --- | --- | --- | | 16.0 0.474|  |  |  |  |  |  | | --- | --- | --- | --- | --- | --- | | 32.0 0.352|  |  |  |  | | --- | --- | --- | --- | | 64.0 0.2165|  |  | | --- | --- | | 128.0 0.203 | | | | | | | | | | | |

6 data points entered.

|  |  |  |  |  |  |  |  |  |  |
| --- | --- | --- | --- | --- | --- | --- | --- | --- | --- |
| X-int: 0.91912|  |  |  |  |  |  |  |  | | --- | --- | --- | --- | --- | --- | --- | --- | | Y-int: 0.47282 +/- 0.09371|  |  |  |  |  |  | | --- | --- | --- | --- | --- | --- | | m: -0.5144 +/- 0.06467|  |  |  |  | | --- | --- | --- | --- | | Dm: 8.30087|  |  | | --- | --- | | r: -0.9698 | | | | | | | | | |

---

Data for Drug Combo: 6+9 (6+9 [1:5])

| Dose A Effect | |
| --- | --- |
| 0.80000+ 0.358|  |  |  |  |  |  |  |  |  |  | | --- | --- | --- | --- | --- | --- | --- | --- | --- | --- | | 1.60000+ 0.3145|  |  |  |  |  |  |  |  | | --- | --- | --- | --- | --- | --- | --- | --- | | 3.20000+ 0.297|  |  |  |  |  |  | | --- | --- | --- | --- | --- | --- | | 6.40000+ 0.227|  |  |  |  | | --- | --- | --- | --- | | 12.8000+ 0.201|  |  | | --- | --- | | 25.8+ 0.19 | | | | | | | | | | | |

6 data points entered.

|  |  |  |  |  |  |  |  |  |  |
| --- | --- | --- | --- | --- | --- | --- | --- | --- | --- |
| X-int: -0.2663|  |  |  |  |  |  |  |  | | --- | --- | --- | --- | --- | --- | --- | --- | | Y-int: -0.0712 +/- 0.03976|  |  |  |  |  |  | | --- | --- | --- | --- | --- | --- | | m: -0.2673 +/- 0.02609|  |  |  |  | | --- | --- | --- | --- | | Dm: 0.54157|  |  | | --- | --- | | r: -0.9815 | | | | | | | | | |

---

Dose-Effect Curve  


---

Median-Effect Plot  


---

CI Data for Drug Combo: 6+9 (6+9 [1:5])

| Fa CI Value Total Dose | | |
| --- | --- | --- |
| 0.05 11.6303 32952.4|  |  |  |  |  |  |  |  |  |  |  |  |  |  |  |  |  |  |  |  |  |  |  |  |  |  |  |  |  |  |  |  |  |  |  |  |  |  |  |  |  |  |  |  |  |  |  |  |  |  |  |  |  |  |  |  |  | | --- | --- | --- | --- | --- | --- | --- | --- | --- | --- | --- | --- | --- | --- | --- | --- | --- | --- | --- | --- | --- | --- | --- | --- | --- | --- | --- | --- | --- | --- | --- | --- | --- | --- | --- | --- | --- | --- | --- | --- | --- | --- | --- | --- | --- | --- | --- | --- | --- | --- | --- | --- | --- | --- | --- | --- | --- | | 0.1 3.52443 2012.73|  |  |  |  |  |  |  |  |  |  |  |  |  |  |  |  |  |  |  |  |  |  |  |  |  |  |  |  |  |  |  |  |  |  |  |  |  |  |  |  |  |  |  |  |  |  |  |  |  |  |  |  |  |  | | --- | --- | --- | --- | --- | --- | --- | --- | --- | --- | --- | --- | --- | --- | --- | --- | --- | --- | --- | --- | --- | --- | --- | --- | --- | --- | --- | --- | --- | --- | --- | --- | --- | --- | --- | --- | --- | --- | --- | --- | --- | --- | --- | --- | --- | --- | --- | --- | --- | --- | --- | --- | --- | --- | | 0.15 1.86656 356.530|  |  |  |  |  |  |  |  |  |  |  |  |  |  |  |  |  |  |  |  |  |  |  |  |  |  |  |  |  |  |  |  |  |  |  |  |  |  |  |  |  |  |  |  |  |  |  |  |  |  |  | | --- | --- | --- | --- | --- | --- | --- | --- | --- | --- | --- | --- | --- | --- | --- | --- | --- | --- | --- | --- | --- | --- | --- | --- | --- | --- | --- | --- | --- | --- | --- | --- | --- | --- | --- | --- | --- | --- | --- | --- | --- | --- | --- | --- | --- | --- | --- | --- | --- | --- | --- | | 0.2 1.25015 96.8622|  |  |  |  |  |  |  |  |  |  |  |  |  |  |  |  |  |  |  |  |  |  |  |  |  |  |  |  |  |  |  |  |  |  |  |  |  |  |  |  |  |  |  |  |  |  |  |  | | --- | --- | --- | --- | --- | --- | --- | --- | --- | --- | --- | --- | --- | --- | --- | --- | --- | --- | --- | --- | --- | --- | --- | --- | --- | --- | --- | --- | --- | --- | --- | --- | --- | --- | --- | --- | --- | --- | --- | --- | --- | --- | --- | --- | --- | --- | --- | --- | | 0.25 0.95033 33.0155|  |  |  |  |  |  |  |  |  |  |  |  |  |  |  |  |  |  |  |  |  |  |  |  |  |  |  |  |  |  |  |  |  |  |  |  |  |  |  |  |  |  |  |  |  | | --- | --- | --- | --- | --- | --- | --- | --- | --- | --- | --- | --- | --- | --- | --- | --- | --- | --- | --- | --- | --- | --- | --- | --- | --- | --- | --- | --- | --- | --- | --- | --- | --- | --- | --- | --- | --- | --- | --- | --- | --- | --- | --- | --- | --- | | 0.3 0.77940 12.8936|  |  |  |  |  |  |  |  |  |  |  |  |  |  |  |  |  |  |  |  |  |  |  |  |  |  |  |  |  |  |  |  |  |  |  |  |  |  |  |  |  |  | | --- | --- | --- | --- | --- | --- | --- | --- | --- | --- | --- | --- | --- | --- | --- | --- | --- | --- | --- | --- | --- | --- | --- | --- | --- | --- | --- | --- | --- | --- | --- | --- | --- | --- | --- | --- | --- | --- | --- | --- | --- | --- | | 0.35 0.67081 5.48895|  |  |  |  |  |  |  |  |  |  |  |  |  |  |  |  |  |  |  |  |  |  |  |  |  |  |  |  |  |  |  |  |  |  |  |  |  |  |  | | --- | --- | --- | --- | --- | --- | --- | --- | --- | --- | --- | --- | --- | --- | --- | --- | --- | --- | --- | --- | --- | --- | --- | --- | --- | --- | --- | --- | --- | --- | --- | --- | --- | --- | --- | --- | --- | --- | --- | | 0.4 0.59602 2.46869|  |  |  |  |  |  |  |  |  |  |  |  |  |  |  |  |  |  |  |  |  |  |  |  |  |  |  |  |  |  |  |  |  |  |  |  | | --- | --- | --- | --- | --- | --- | --- | --- | --- | --- | --- | --- | --- | --- | --- | --- | --- | --- | --- | --- | --- | --- | --- | --- | --- | --- | --- | --- | --- | --- | --- | --- | --- | --- | --- | --- | | 0.45 0.54107 1.14738|  |  |  |  |  |  |  |  |  |  |  |  |  |  |  |  |  |  |  |  |  |  |  |  |  |  |  |  |  |  |  |  |  | | --- | --- | --- | --- | --- | --- | --- | --- | --- | --- | --- | --- | --- | --- | --- | --- | --- | --- | --- | --- | --- | --- | --- | --- | --- | --- | --- | --- | --- | --- | --- | --- | --- | | 0.5 0.49844 0.54157|  |  |  |  |  |  |  |  |  |  |  |  |  |  |  |  |  |  |  |  |  |  |  |  |  |  |  |  |  |  | | --- | --- | --- | --- | --- | --- | --- | --- | --- | --- | --- | --- | --- | --- | --- | --- | --- | --- | --- | --- | --- | --- | --- | --- | --- | --- | --- | --- | --- | --- | | 0.55 0.46375 0.25562|  |  |  |  |  |  |  |  |  |  |  |  |  |  |  |  |  |  |  |  |  |  |  |  |  |  |  | | --- | --- | --- | --- | --- | --- | --- | --- | --- | --- | --- | --- | --- | --- | --- | --- | --- | --- | --- | --- | --- | --- | --- | --- | --- | --- | --- | | 0.6 0.43424 0.11881|  |  |  |  |  |  |  |  |  |  |  |  |  |  |  |  |  |  |  |  |  |  |  |  | | --- | --- | --- | --- | --- | --- | --- | --- | --- | --- | --- | --- | --- | --- | --- | --- | --- | --- | --- | --- | --- | --- | --- | --- | | 0.65 0.40807 0.05343|  |  |  |  |  |  |  |  |  |  |  |  |  |  |  |  |  |  |  |  |  | | --- | --- | --- | --- | --- | --- | --- | --- | --- | --- | --- | --- | --- | --- | --- | --- | --- | --- | --- | --- | --- | | 0.7 0.38388 0.02275|  |  |  |  |  |  |  |  |  |  |  |  |  |  |  |  |  |  | | --- | --- | --- | --- | --- | --- | --- | --- | --- | --- | --- | --- | --- | --- | --- | --- | --- | --- | | 0.75 0.36054 0.00888|  |  |  |  |  |  |  |  |  |  |  |  |  |  |  | | --- | --- | --- | --- | --- | --- | --- | --- | --- | --- | --- | --- | --- | --- | --- | | 0.8 0.33690 0.00303|  |  |  |  |  |  |  |  |  |  |  |  | | --- | --- | --- | --- | --- | --- | --- | --- | --- | --- | --- | --- | | 0.85 0.31147 8.23E-4|  |  |  |  |  |  |  |  |  | | --- | --- | --- | --- | --- | --- | --- | --- | --- | | 0.9 0.28164 1.46E-4|  |  |  |  |  |  | | --- | --- | --- | --- | --- | --- | | 0.95 0.24031 8.90E-6|  |  |  | | --- | --- | --- | | 0.97 0.21490 1.22E-6 | | | | | | | | | | | | | | | | | | | | | | | | | | | | | | | | | | | | | | | | | | | | | | | | | | | | | | | | | | | |

CI values for actual experimental points:

Total Dose Fa CI Value | | || 4.8 0.358 0.65492|  |  |  |  |  |  |  |  |  |  |  |  |  |  |  | | --- | --- | --- | --- | --- | --- | --- | --- | --- | --- | --- | --- | --- | --- | --- | | 9.6 0.3145 0.71399|  |  |  |  |  |  |  |  |  |  |  |  | | --- | --- | --- | --- | --- | --- | --- | --- | --- | --- | --- | --- | | 19.2 0.297 1.11146|  |  |  |  |  |  |  |  |  | | --- | --- | --- | --- | --- | --- | --- | --- | --- | | 38.4 0.227 0.77139|  |  |  |  |  |  | | --- | --- | --- | --- | --- | --- | | 76.8 0.201 1.00805|  |  |  | | --- | --- | --- | | 154.8 0.19 1.68454 | | | | | | | | | | | | | | | | | |

---

Combination Index Plot  


---

DRI Data for Drug Combo: 6+9 (6+9 [1:5])

| Fa Dose 6 Dose 9 DRI 6 DRI 9 | | | | |
| --- | --- | --- | --- | --- |
| 0.05 6684.86 2540.57 1.21719 0.09252|  |  |  |  |  |  |  |  |  |  |  |  |  |  |  |  |  |  |  |  |  |  |  |  |  |  |  |  |  |  |  |  |  |  |  |  |  |  |  |  |  |  |  |  |  |  |  |  |  |  |  |  |  |  |  |  |  |  |  |  |  |  |  |  |  |  |  |  |  |  |  |  |  |  |  |  |  |  |  |  |  |  |  |  |  |  |  |  |  |  |  |  |  |  |  | | --- | --- | --- | --- | --- | --- | --- | --- | --- | --- | --- | --- | --- | --- | --- | --- | --- | --- | --- | --- | --- | --- | --- | --- | --- | --- | --- | --- | --- | --- | --- | --- | --- | --- | --- | --- | --- | --- | --- | --- | --- | --- | --- | --- | --- | --- | --- | --- | --- | --- | --- | --- | --- | --- | --- | --- | --- | --- | --- | --- | --- | --- | --- | --- | --- | --- | --- | --- | --- | --- | --- | --- | --- | --- | --- | --- | --- | --- | --- | --- | --- | --- | --- | --- | --- | --- | --- | --- | --- | --- | --- | --- | --- | --- | --- | | 0.1 477.303 594.435 1.42286 0.35441|  |  |  |  |  |  |  |  |  |  |  |  |  |  |  |  |  |  |  |  |  |  |  |  |  |  |  |  |  |  |  |  |  |  |  |  |  |  |  |  |  |  |  |  |  |  |  |  |  |  |  |  |  |  |  |  |  |  |  |  |  |  |  |  |  |  |  |  |  |  |  |  |  |  |  |  |  |  |  |  |  |  |  |  |  |  |  |  |  |  | | --- | --- | --- | --- | --- | --- | --- | --- | --- | --- | --- | --- | --- | --- | --- | --- | --- | --- | --- | --- | --- | --- | --- | --- | --- | --- | --- | --- | --- | --- | --- | --- | --- | --- | --- | --- | --- | --- | --- | --- | --- | --- | --- | --- | --- | --- | --- | --- | --- | --- | --- | --- | --- | --- | --- | --- | --- | --- | --- | --- | --- | --- | --- | --- | --- | --- | --- | --- | --- | --- | --- | --- | --- | --- | --- | --- | --- | --- | --- | --- | --- | --- | --- | --- | --- | --- | --- | --- | --- | --- | | 0.15 93.1293 241.847 1.56726 0.81400|  |  |  |  |  |  |  |  |  |  |  |  |  |  |  |  |  |  |  |  |  |  |  |  |  |  |  |  |  |  |  |  |  |  |  |  |  |  |  |  |  |  |  |  |  |  |  |  |  |  |  |  |  |  |  |  |  |  |  |  |  |  |  |  |  |  |  |  |  |  |  |  |  |  |  |  |  |  |  |  |  |  |  |  |  | | --- | --- | --- | --- | --- | --- | --- | --- | --- | --- | --- | --- | --- | --- | --- | --- | --- | --- | --- | --- | --- | --- | --- | --- | --- | --- | --- | --- | --- | --- | --- | --- | --- | --- | --- | --- | --- | --- | --- | --- | --- | --- | --- | --- | --- | --- | --- | --- | --- | --- | --- | --- | --- | --- | --- | --- | --- | --- | --- | --- | --- | --- | --- | --- | --- | --- | --- | --- | --- | --- | --- | --- | --- | --- | --- | --- | --- | --- | --- | --- | --- | --- | --- | --- | --- | | 0.2 27.2114 122.881 1.68557 1.52234|  |  |  |  |  |  |  |  |  |  |  |  |  |  |  |  |  |  |  |  |  |  |  |  |  |  |  |  |  |  |  |  |  |  |  |  |  |  |  |  |  |  |  |  |  |  |  |  |  |  |  |  |  |  |  |  |  |  |  |  |  |  |  |  |  |  |  |  |  |  |  |  |  |  |  |  |  |  |  |  | | --- | --- | --- | --- | --- | --- | --- | --- | --- | --- | --- | --- | --- | --- | --- | --- | --- | --- | --- | --- | --- | --- | --- | --- | --- | --- | --- | --- | --- | --- | --- | --- | --- | --- | --- | --- | --- | --- | --- | --- | --- | --- | --- | --- | --- | --- | --- | --- | --- | --- | --- | --- | --- | --- | --- | --- | --- | --- | --- | --- | --- | --- | --- | --- | --- | --- | --- | --- | --- | --- | --- | --- | --- | --- | --- | --- | --- | --- | --- | --- | | 0.25 9.84961 70.2448 1.79000 2.55316|  |  |  |  |  |  |  |  |  |  |  |  |  |  |  |  |  |  |  |  |  |  |  |  |  |  |  |  |  |  |  |  |  |  |  |  |  |  |  |  |  |  |  |  |  |  |  |  |  |  |  |  |  |  |  |  |  |  |  |  |  |  |  |  |  |  |  |  |  |  |  |  |  |  |  | | --- | --- | --- | --- | --- | --- | --- | --- | --- | --- | --- | --- | --- | --- | --- | --- | --- | --- | --- | --- | --- | --- | --- | --- | --- | --- | --- | --- | --- | --- | --- | --- | --- | --- | --- | --- | --- | --- | --- | --- | --- | --- | --- | --- | --- | --- | --- | --- | --- | --- | --- | --- | --- | --- | --- | --- | --- | --- | --- | --- | --- | --- | --- | --- | --- | --- | --- | --- | --- | --- | --- | --- | --- | --- | --- | | 0.3 4.05395 43.0968 1.88650 4.01101|  |  |  |  |  |  |  |  |  |  |  |  |  |  |  |  |  |  |  |  |  |  |  |  |  |  |  |  |  |  |  |  |  |  |  |  |  |  |  |  |  |  |  |  |  |  |  |  |  |  |  |  |  |  |  |  |  |  |  |  |  |  |  |  |  |  |  |  |  |  | | --- | --- | --- | --- | --- | --- | --- | --- | --- | --- | --- | --- | --- | --- | --- | --- | --- | --- | --- | --- | --- | --- | --- | --- | --- | --- | --- | --- | --- | --- | --- | --- | --- | --- | --- | --- | --- | --- | --- | --- | --- | --- | --- | --- | --- | --- | --- | --- | --- | --- | --- | --- | --- | --- | --- | --- | --- | --- | --- | --- | --- | --- | --- | --- | --- | --- | --- | --- | --- | --- | | 0.35 1.81012 27.6529 1.97866 6.04550|  |  |  |  |  |  |  |  |  |  |  |  |  |  |  |  |  |  |  |  |  |  |  |  |  |  |  |  |  |  |  |  |  |  |  |  |  |  |  |  |  |  |  |  |  |  |  |  |  |  |  |  |  |  |  |  |  |  |  |  |  |  |  |  |  | | --- | --- | --- | --- | --- | --- | --- | --- | --- | --- | --- | --- | --- | --- | --- | --- | --- | --- | --- | --- | --- | --- | --- | --- | --- | --- | --- | --- | --- | --- | --- | --- | --- | --- | --- | --- | --- | --- | --- | --- | --- | --- | --- | --- | --- | --- | --- | --- | --- | --- | --- | --- | --- | --- | --- | --- | --- | --- | --- | --- | --- | --- | --- | --- | --- | | 0.4 0.85127 18.2571 2.06895 8.87458|  |  |  |  |  |  |  |  |  |  |  |  |  |  |  |  |  |  |  |  |  |  |  |  |  |  |  |  |  |  |  |  |  |  |  |  |  |  |  |  |  |  |  |  |  |  |  |  |  |  |  |  |  |  |  |  |  |  |  |  | | --- | --- | --- | --- | --- | --- | --- | --- | --- | --- | --- | --- | --- | --- | --- | --- | --- | --- | --- | --- | --- | --- | --- | --- | --- | --- | --- | --- | --- | --- | --- | --- | --- | --- | --- | --- | --- | --- | --- | --- | --- | --- | --- | --- | --- | --- | --- | --- | --- | --- | --- | --- | --- | --- | --- | --- | --- | --- | --- | --- | | 0.45 0.41294 12.2613 2.15941 12.8236|  |  |  |  |  |  |  |  |  |  |  |  |  |  |  |  |  |  |  |  |  |  |  |  |  |  |  |  |  |  |  |  |  |  |  |  |  |  |  |  |  |  |  |  |  |  |  |  |  |  |  |  |  |  |  | | --- | --- | --- | --- | --- | --- | --- | --- | --- | --- | --- | --- | --- | --- | --- | --- | --- | --- | --- | --- | --- | --- | --- | --- | --- | --- | --- | --- | --- | --- | --- | --- | --- | --- | --- | --- | --- | --- | --- | --- | --- | --- | --- | --- | --- | --- | --- | --- | --- | --- | --- | --- | --- | --- | --- | | 0.5 0.20326 8.30087 2.25187 18.3930|  |  |  |  |  |  |  |  |  |  |  |  |  |  |  |  |  |  |  |  |  |  |  |  |  |  |  |  |  |  |  |  |  |  |  |  |  |  |  |  |  |  |  |  |  |  |  |  |  |  | | --- | --- | --- | --- | --- | --- | --- | --- | --- | --- | --- | --- | --- | --- | --- | --- | --- | --- | --- | --- | --- | --- | --- | --- | --- | --- | --- | --- | --- | --- | --- | --- | --- | --- | --- | --- | --- | --- | --- | --- | --- | --- | --- | --- | --- | --- | --- | --- | --- | --- | | 0.55 0.10005 5.61965 2.34830 26.3814|  |  |  |  |  |  |  |  |  |  |  |  |  |  |  |  |  |  |  |  |  |  |  |  |  |  |  |  |  |  |  |  |  |  |  |  |  |  |  |  |  |  |  |  |  | | --- | --- | --- | --- | --- | --- | --- | --- | --- | --- | --- | --- | --- | --- | --- | --- | --- | --- | --- | --- | --- | --- | --- | --- | --- | --- | --- | --- | --- | --- | --- | --- | --- | --- | --- | --- | --- | --- | --- | --- | --- | --- | --- | --- | --- | | 0.6 0.04853 3.77411 2.45096 38.1206|  |  |  |  |  |  |  |  |  |  |  |  |  |  |  |  |  |  |  |  |  |  |  |  |  |  |  |  |  |  |  |  |  |  |  |  |  |  |  |  | | --- | --- | --- | --- | --- | --- | --- | --- | --- | --- | --- | --- | --- | --- | --- | --- | --- | --- | --- | --- | --- | --- | --- | --- | --- | --- | --- | --- | --- | --- | --- | --- | --- | --- | --- | --- | --- | --- | --- | --- | | 0.65 0.02282 2.49176 2.56282 55.9597|  |  |  |  |  |  |  |  |  |  |  |  |  |  |  |  |  |  |  |  |  |  |  |  |  |  |  |  |  |  |  |  |  |  |  | | --- | --- | --- | --- | --- | --- | --- | --- | --- | --- | --- | --- | --- | --- | --- | --- | --- | --- | --- | --- | --- | --- | --- | --- | --- | --- | --- | --- | --- | --- | --- | --- | --- | --- | --- | | 0.7 0.01019 1.59883 2.68801 84.3440|  |  |  |  |  |  |  |  |  |  |  |  |  |  |  |  |  |  |  |  |  |  |  |  |  |  |  |  |  |  | | --- | --- | --- | --- | --- | --- | --- | --- | --- | --- | --- | --- | --- | --- | --- | --- | --- | --- | --- | --- | --- | --- | --- | --- | --- | --- | --- | --- | --- | --- | | 0.75 0.00419 0.98092 2.83293 132.504|  |  |  |  |  |  |  |  |  |  |  |  |  |  |  |  |  |  |  |  |  |  |  |  |  | | --- | --- | --- | --- | --- | --- | --- | --- | --- | --- | --- | --- | --- | --- | --- | --- | --- | --- | --- | --- | --- | --- | --- | --- | --- | | 0.8 0.00152 0.56074 3.00843 222.226|  |  |  |  |  |  |  |  |  |  |  |  |  |  |  |  |  |  |  |  | | --- | --- | --- | --- | --- | --- | --- | --- | --- | --- | --- | --- | --- | --- | --- | --- | --- | --- | --- | --- | | 0.85 4.44E-4 0.28491 3.23554 415.607|  |  |  |  |  |  |  |  |  |  |  |  |  |  |  | | --- | --- | --- | --- | --- | --- | --- | --- | --- | --- | --- | --- | --- | --- | --- | | 0.9 8.66E-5 0.11592 3.56391 954.567|  |  |  |  |  |  |  |  |  |  | | --- | --- | --- | --- | --- | --- | --- | --- | --- | --- | | 0.95 6.18E-6 0.02712 4.16611 3656.64|  |  |  |  |  | | --- | --- | --- | --- | --- | | 0.97 9.45E-7 0.00965 4.65560 9508.13 | | | | | | | | | | | | | | | | | | | | | | | | | | | | | | | | | | | | | | | | | | | | | | | | | | | | | | | | | | | | | | | | | | | | | | | | | | | | | | | | | | | | | | | | | | | | | | | | | | | |

DRI values calculated at experimental points

| Fa Dose 6 Dose 9 DRI 6 DRI 9 | | | | |
| --- | --- | --- | --- | --- |
| 0.358 1.59970 25.8348 1.99963 6.45870|  |  |  |  |  |  |  |  |  |  |  |  |  |  |  |  |  |  |  |  |  |  |  |  |  | | --- | --- | --- | --- | --- | --- | --- | --- | --- | --- | --- | --- | --- | --- | --- | --- | --- | --- | --- | --- | --- | --- | --- | --- | --- | | 0.3145 3.18681 37.7506 1.99175 4.71882|  |  |  |  |  |  |  |  |  |  |  |  |  |  |  |  |  |  |  |  | | --- | --- | --- | --- | --- | --- | --- | --- | --- | --- | --- | --- | --- | --- | --- | --- | --- | --- | --- | --- | | 0.297 4.26440 44.3139 1.33262 2.76962|  |  |  |  |  |  |  |  |  |  |  |  |  |  |  | | --- | --- | --- | --- | --- | --- | --- | --- | --- | --- | --- | --- | --- | --- | --- | | 0.227 15.4104 89.8655 2.40788 2.80830|  |  |  |  |  |  |  |  |  |  | | --- | --- | --- | --- | --- | --- | --- | --- | --- | --- | | 0.201 26.6183 121.400 2.07956 1.89688|  |  |  |  |  | | --- | --- | --- | --- | --- | | 0.19 34.0798 139.084 1.32092 1.07817 | | | | | | | | | | | | | | | | | | | | | | | | | | | | | |

---

DRI Plot for Combo: 6+9 (6+9 [1:5])  


---

Isobologram for Combo: 6+9 (6+9 [1:5])  


---

Summary Table

|  |  |  |  |  |  |  |  |
| --- | --- | --- | --- | --- | --- | --- | --- |
| Experiment Name: RKO|  |  |  |  |  |  | | --- | --- | --- | --- | --- | --- | | Date: |  |  |  |  | | --- | --- | --- | --- | | File Name: K:\20180502 CI\20180510\RKO\RKO.cse|  |  | | --- | --- | | Description  | | | | | | | |

|  |  |  |  |  |  |
| --- | --- | --- | --- | --- | --- |
| Drug: MK-2206 (6) [uM]|  |  |  |  | | --- | --- | --- | --- | | Drug: SHP099 (9) [uM]|  |  | | --- | --- | | Drug Combo: MK-2206+SHP099 (6+9) (6+9 [1:5]) | | | | | |

---

| Drug/Combo Dm m r | | | |
| --- | --- | --- | --- |
| 6 0.20326 -0.2831 -0.9481|  |  |  |  |  |  |  |  | | --- | --- | --- | --- | --- | --- | --- | --- | | 9 8.30087 -0.5144 -0.9698|  |  |  |  | | --- | --- | --- | --- | | 6+9 0.54157 -0.2673 -0.9815 | | | | | | | | | | | |

---

|  |  |  |  |  |  |  |  |  |  |
| --- | --- | --- | --- | --- | --- | --- | --- | --- | --- |
| CI values at:| Combo ED50 ED75 ED90 ED95 | | | | | | --- | --- | --- | --- | --- | | | | | |
| 6+9 0.49844 0.36054 0.28164 0.24031 | | | | |

---

Data for Fa = 0.5

| Drug/Combo CI value Dose 6 Dose 9 | | | |
| --- | --- | --- | --- |
| 6 0.20326|  |  |  |  |  |  |  |  | | --- | --- | --- | --- | --- | --- | --- | --- | | 9 8.30087|  |  |  |  | | --- | --- | --- | --- | | 6+9 0.49844 0.09026 0.45130 | | | | | | | | | | |

---

Data for Fa = 0.75

| Drug/Combo CI value Dose 6 Dose 9 | | | |
| --- | --- | --- | --- |
| 6 0.00419|  |  |  |  |  |  |  |  | | --- | --- | --- | --- | --- | --- | --- | --- | | 9 0.98092|  |  |  |  | | --- | --- | --- | --- | | 6+9 0.36054 0.00148 0.00740 | | | | | | | | | | |

---

Data for Fa = 0.9

| Drug/Combo CI value Dose 6 Dose 9 | | | |
| --- | --- | --- | --- |
| 6 8.66E-5|  |  |  |  |  |  |  |  | | --- | --- | --- | --- | --- | --- | --- | --- | | 9 0.11592|  |  |  |  | | --- | --- | --- | --- | | 6+9 0.28164 2.43E-5 1.21E-4 | | | | | | | | | | |

---

Data for Fa = 0.95

| Drug/Combo CI value Dose 6 Dose 9 | | | |
| --- | --- | --- | --- |
| 6 6.18E-6|  |  |  |  |  |  |  |  | | --- | --- | --- | --- | --- | --- | --- | --- | | 9 0.02712|  |  |  |  | | --- | --- | --- | --- | | 6+9 0.24031 1.48E-6 7.42E-6 | | | | | | | | | | |

---

Data for Fa = 0.97

| Drug/Combo CI value Dose 6 Dose 9 | | | |
| --- | --- | --- | --- |
| 6 9.45E-7|  |  |  |  |  |  |  |  | | --- | --- | --- | --- | --- | --- | --- | --- | | 9 0.00965|  |  |  |  | | --- | --- | --- | --- | | 6+9 0.21490 2.03E-7 1.01E-6 | | | | | | | | | | |
