# Supplementary material for: Therapeutic Suppression of FAK-AKT Signaling Overcomes Resistance to SHP2 Inhibition in Colorectal Carcinoma
Source: Front Pharmacol. 2021 Nov 1;12:739501. doi: 10.3389/fphar.2021.739501 (PMC8591248; doi:10.3389/fphar.2021.739501)
Supplement: Supplementary file 11 [file DataSheet6.ZIP › Figure3/Figure3B/SW480/480-report/report.html]

CompuSyn Report


CompuSyn Report

|  |  |  |  |  |  |  |  |
| --- | --- | --- | --- | --- | --- | --- | --- |
| Experiment Name: SW480|  |  |  |  |  |  | | --- | --- | --- | --- | --- | --- | | Date: |  |  |  |  | | --- | --- | --- | --- | | File Name: E:\SHP2\SHP099+MK-2206Í¶¸å\Êý¾Ý¼°´¦Àí\4-CI\20180510 RKO 480 CI\SW480\480.cse|  |  | | --- | --- | | Description  | | | | | | | |

|  |  |  |  |  |  |
| --- | --- | --- | --- | --- | --- |
| Drug: MK-2206 (6) [uM]|  |  |  |  | | --- | --- | --- | --- | | Drug: SHP099 (9) [uM]|  |  | | --- | --- | | Drug Combo: MK-2206+SHP099 (6+9) (6+9 [1:5]) | | | | | |

---

Data for Drug: 6 [uM]

| Dose Effect | |
| --- | --- |
| 0.8 0.7335|  |  |  |  |  |  |  |  |  |  | | --- | --- | --- | --- | --- | --- | --- | --- | --- | --- | | 1.6 0.7255|  |  |  |  |  |  |  |  | | --- | --- | --- | --- | --- | --- | --- | --- | | 3.2 0.7015|  |  |  |  |  |  | | --- | --- | --- | --- | --- | --- | | 6.4 0.685|  |  |  |  | | --- | --- | --- | --- | | 12.8 0.3295|  |  | | --- | --- | | 25.6 0.275 | | | | | | | | | | | |

6 data points entered.

|  |  |  |  |  |  |  |  |  |  |
| --- | --- | --- | --- | --- | --- | --- | --- | --- | --- |
| X-int: 0.88177|  |  |  |  |  |  |  |  | | --- | --- | --- | --- | --- | --- | --- | --- | | Y-int: 0.54643 +/- 0.13587|  |  |  |  |  |  | | --- | --- | --- | --- | --- | --- | | m: -0.6197 +/- 0.16307|  |  |  |  | | --- | --- | --- | --- | | Dm: 7.61682|  |  | | --- | --- | | r: -0.8849 | | | | | | | | | |

---

Data for Drug: 9 [uM]

| Dose Effect | |
| --- | --- |
| 4.0 0.758|  |  |  |  |  |  |  |  |  |  | | --- | --- | --- | --- | --- | --- | --- | --- | --- | --- | | 8.0 0.732|  |  |  |  |  |  |  |  | | --- | --- | --- | --- | --- | --- | --- | --- | | 16.0 0.705|  |  |  |  |  |  | | --- | --- | --- | --- | --- | --- | | 32.0 0.68|  |  |  |  | | --- | --- | --- | --- | | 64.0 0.328|  |  | | --- | --- | | 128.0 0.2795 | | | | | | | | | | | |

6 data points entered.

|  |  |  |  |  |  |  |  |  |  |
| --- | --- | --- | --- | --- | --- | --- | --- | --- | --- |
| X-int: 1.58993|  |  |  |  |  |  |  |  | | --- | --- | --- | --- | --- | --- | --- | --- | | Y-int: 1.03070 +/- 0.22037|  |  |  |  |  |  | | --- | --- | --- | --- | --- | --- | | m: -0.6483 +/- 0.15209|  |  |  |  | | --- | --- | --- | --- | | Dm: 38.8984|  |  | | --- | --- | | r: -0.9053 | | | | | | | | | |

---

Data for Drug Combo: 6+9 (6+9 [1:5])

| Dose A Effect | |
| --- | --- |
| 0.80000+ 0.631|  |  |  |  |  |  |  |  |  |  | | --- | --- | --- | --- | --- | --- | --- | --- | --- | --- | | 1.60000+ 0.5795|  |  |  |  |  |  |  |  | | --- | --- | --- | --- | --- | --- | --- | --- | | 3.20000+ 0.542|  |  |  |  |  |  | | --- | --- | --- | --- | --- | --- | | 6.40000+ 0.432|  |  |  |  | | --- | --- | --- | --- | | 12.8000+ 0.27|  |  | | --- | --- | | 25.6000+ 0.265 | | | | | | | | | | | |

6 data points entered.

|  |  |  |  |  |  |  |  |  |  |
| --- | --- | --- | --- | --- | --- | --- | --- | --- | --- |
| X-int: 1.25162|  |  |  |  |  |  |  |  | | --- | --- | --- | --- | --- | --- | --- | --- | | Y-int: 0.62794 +/- 0.09891|  |  |  |  |  |  | | --- | --- | --- | --- | --- | --- | | m: -0.5017 +/- 0.06494|  |  |  |  | | --- | --- | --- | --- | | Dm: 17.8494|  |  | | --- | --- | | r: -0.9681 | | | | | | | | | |

---

Dose-Effect Curve  


---

Median-Effect Plot  


---

CI Data for Drug Combo: 6+9 (6+9 [1:5])

| Fa CI Value Total Dose | | |
| --- | --- | --- |
| 0.05 2.63535 6316.15|  |  |  |  |  |  |  |  |  |  |  |  |  |  |  |  |  |  |  |  |  |  |  |  |  |  |  |  |  |  |  |  |  |  |  |  |  |  |  |  |  |  |  |  |  |  |  |  |  |  |  |  |  |  |  |  |  | | --- | --- | --- | --- | --- | --- | --- | --- | --- | --- | --- | --- | --- | --- | --- | --- | --- | --- | --- | --- | --- | --- | --- | --- | --- | --- | --- | --- | --- | --- | --- | --- | --- | --- | --- | --- | --- | --- | --- | --- | --- | --- | --- | --- | --- | --- | --- | --- | --- | --- | --- | --- | --- | --- | --- | --- | --- | | 0.1 1.92846 1424.40|  |  |  |  |  |  |  |  |  |  |  |  |  |  |  |  |  |  |  |  |  |  |  |  |  |  |  |  |  |  |  |  |  |  |  |  |  |  |  |  |  |  |  |  |  |  |  |  |  |  |  |  |  |  | | --- | --- | --- | --- | --- | --- | --- | --- | --- | --- | --- | --- | --- | --- | --- | --- | --- | --- | --- | --- | --- | --- | --- | --- | --- | --- | --- | --- | --- | --- | --- | --- | --- | --- | --- | --- | --- | --- | --- | --- | --- | --- | --- | --- | --- | --- | --- | --- | --- | --- | --- | --- | --- | --- | | 0.15 1.58998 566.456|  |  |  |  |  |  |  |  |  |  |  |  |  |  |  |  |  |  |  |  |  |  |  |  |  |  |  |  |  |  |  |  |  |  |  |  |  |  |  |  |  |  |  |  |  |  |  |  |  |  |  | | --- | --- | --- | --- | --- | --- | --- | --- | --- | --- | --- | --- | --- | --- | --- | --- | --- | --- | --- | --- | --- | --- | --- | --- | --- | --- | --- | --- | --- | --- | --- | --- | --- | --- | --- | --- | --- | --- | --- | --- | --- | --- | --- | --- | --- | --- | --- | --- | --- | --- | --- | | 0.2 1.37519 282.916|  |  |  |  |  |  |  |  |  |  |  |  |  |  |  |  |  |  |  |  |  |  |  |  |  |  |  |  |  |  |  |  |  |  |  |  |  |  |  |  |  |  |  |  |  |  |  |  | | --- | --- | --- | --- | --- | --- | --- | --- | --- | --- | --- | --- | --- | --- | --- | --- | --- | --- | --- | --- | --- | --- | --- | --- | --- | --- | --- | --- | --- | --- | --- | --- | --- | --- | --- | --- | --- | --- | --- | --- | --- | --- | --- | --- | --- | --- | --- | --- | | 0.25 1.21998 159.451|  |  |  |  |  |  |  |  |  |  |  |  |  |  |  |  |  |  |  |  |  |  |  |  |  |  |  |  |  |  |  |  |  |  |  |  |  |  |  |  |  |  |  |  |  | | --- | --- | --- | --- | --- | --- | --- | --- | --- | --- | --- | --- | --- | --- | --- | --- | --- | --- | --- | --- | --- | --- | --- | --- | --- | --- | --- | --- | --- | --- | --- | --- | --- | --- | --- | --- | --- | --- | --- | --- | --- | --- | --- | --- | --- | | 0.3 1.09890 96.6227|  |  |  |  |  |  |  |  |  |  |  |  |  |  |  |  |  |  |  |  |  |  |  |  |  |  |  |  |  |  |  |  |  |  |  |  |  |  |  |  |  |  | | --- | --- | --- | --- | --- | --- | --- | --- | --- | --- | --- | --- | --- | --- | --- | --- | --- | --- | --- | --- | --- | --- | --- | --- | --- | --- | --- | --- | --- | --- | --- | --- | --- | --- | --- | --- | --- | --- | --- | --- | --- | --- | | 0.35 0.99943 61.3040|  |  |  |  |  |  |  |  |  |  |  |  |  |  |  |  |  |  |  |  |  |  |  |  |  |  |  |  |  |  |  |  |  |  |  |  |  |  |  | | --- | --- | --- | --- | --- | --- | --- | --- | --- | --- | --- | --- | --- | --- | --- | --- | --- | --- | --- | --- | --- | --- | --- | --- | --- | --- | --- | --- | --- | --- | --- | --- | --- | --- | --- | --- | --- | --- | --- | | 0.4 0.91460 40.0507|  |  |  |  |  |  |  |  |  |  |  |  |  |  |  |  |  |  |  |  |  |  |  |  |  |  |  |  |  |  |  |  |  |  |  |  | | --- | --- | --- | --- | --- | --- | --- | --- | --- | --- | --- | --- | --- | --- | --- | --- | --- | --- | --- | --- | --- | --- | --- | --- | --- | --- | --- | --- | --- | --- | --- | --- | --- | --- | --- | --- | | 0.45 0.84006 26.6276|  |  |  |  |  |  |  |  |  |  |  |  |  |  |  |  |  |  |  |  |  |  |  |  |  |  |  |  |  |  |  |  |  | | --- | --- | --- | --- | --- | --- | --- | --- | --- | --- | --- | --- | --- | --- | --- | --- | --- | --- | --- | --- | --- | --- | --- | --- | --- | --- | --- | --- | --- | --- | --- | --- | --- | | 0.5 0.77296 17.8494|  |  |  |  |  |  |  |  |  |  |  |  |  |  |  |  |  |  |  |  |  |  |  |  |  |  |  |  |  |  | | --- | --- | --- | --- | --- | --- | --- | --- | --- | --- | --- | --- | --- | --- | --- | --- | --- | --- | --- | --- | --- | --- | --- | --- | --- | --- | --- | --- | --- | --- | | 0.55 0.71126 11.9650|  |  |  |  |  |  |  |  |  |  |  |  |  |  |  |  |  |  |  |  |  |  |  |  |  |  |  | | --- | --- | --- | --- | --- | --- | --- | --- | --- | --- | --- | --- | --- | --- | --- | --- | --- | --- | --- | --- | --- | --- | --- | --- | --- | --- | --- | | 0.6 0.65340 7.95491|  |  |  |  |  |  |  |  |  |  |  |  |  |  |  |  |  |  |  |  |  |  |  |  | | --- | --- | --- | --- | --- | --- | --- | --- | --- | --- | --- | --- | --- | --- | --- | --- | --- | --- | --- | --- | --- | --- | --- | --- | | 0.65 0.59810 5.19705|  |  |  |  |  |  |  |  |  |  |  |  |  |  |  |  |  |  |  |  |  | | --- | --- | --- | --- | --- | --- | --- | --- | --- | --- | --- | --- | --- | --- | --- | --- | --- | --- | --- | --- | --- | | 0.7 0.54419 3.29736|  |  |  |  |  |  |  |  |  |  |  |  |  |  |  |  |  |  | | --- | --- | --- | --- | --- | --- | --- | --- | --- | --- | --- | --- | --- | --- | --- | --- | --- | --- | | 0.75 0.49049 1.99810|  |  |  |  |  |  |  |  |  |  |  |  |  |  |  | | --- | --- | --- | --- | --- | --- | --- | --- | --- | --- | --- | --- | --- | --- | --- | | 0.8 0.43552 1.12613|  |  |  |  |  |  |  |  |  |  |  |  | | --- | --- | --- | --- | --- | --- | --- | --- | --- | --- | --- | --- | | 0.85 0.37720 0.56244|  |  |  |  |  |  |  |  |  | | --- | --- | --- | --- | --- | --- | --- | --- | --- | | 0.9 0.31171 0.22367|  |  |  |  |  |  | | --- | --- | --- | --- | --- | --- | | 0.95 0.22921 0.05044|  |  |  | | --- | --- | --- | | 0.97 0.18425 0.01748 | | | | | | | | | | | | | | | | | | | | | | | | | | | | | | | | | | | | | | | | | | | | | | | | | | | | | | | | | | | |

CI values for actual experimental points:

Total Dose Fa CI Value | | || 4.8 0.631 0.48490|  |  |  |  |  |  |  |  |  |  |  |  |  |  |  | | --- | --- | --- | --- | --- | --- | --- | --- | --- | --- | --- | --- | --- | --- | --- | | 9.6 0.5795 0.68977|  |  |  |  |  |  |  |  |  |  |  |  | | --- | --- | --- | --- | --- | --- | --- | --- | --- | --- | --- | --- | | 19.2 0.542 1.08464|  |  |  |  |  |  |  |  |  | | --- | --- | --- | --- | --- | --- | --- | --- | --- | | 38.4 0.432 1.07959|  |  |  |  |  |  | | --- | --- | --- | --- | --- | --- | | 76.8 0.27 0.69233|  |  |  | | --- | --- | --- | | 153.6 0.265 1.33004 | | | | | | | | | | | | | | | | | |

---

Combination Index Plot  


---

DRI Data for Drug Combo: 6+9 (6+9 [1:5])

| Fa Dose 6 Dose 9 DRI 6 DRI 9 | | | | |
| --- | --- | --- | --- | --- |
| 0.05 881.632 3651.83 0.83750 0.69381|  |  |  |  |  |  |  |  |  |  |  |  |  |  |  |  |  |  |  |  |  |  |  |  |  |  |  |  |  |  |  |  |  |  |  |  |  |  |  |  |  |  |  |  |  |  |  |  |  |  |  |  |  |  |  |  |  |  |  |  |  |  |  |  |  |  |  |  |  |  |  |  |  |  |  |  |  |  |  |  |  |  |  |  |  |  |  |  |  |  |  |  |  |  |  | | --- | --- | --- | --- | --- | --- | --- | --- | --- | --- | --- | --- | --- | --- | --- | --- | --- | --- | --- | --- | --- | --- | --- | --- | --- | --- | --- | --- | --- | --- | --- | --- | --- | --- | --- | --- | --- | --- | --- | --- | --- | --- | --- | --- | --- | --- | --- | --- | --- | --- | --- | --- | --- | --- | --- | --- | --- | --- | --- | --- | --- | --- | --- | --- | --- | --- | --- | --- | --- | --- | --- | --- | --- | --- | --- | --- | --- | --- | --- | --- | --- | --- | --- | --- | --- | --- | --- | --- | --- | --- | --- | --- | --- | --- | --- | | 0.1 264.014 1153.26 1.11210 0.97157|  |  |  |  |  |  |  |  |  |  |  |  |  |  |  |  |  |  |  |  |  |  |  |  |  |  |  |  |  |  |  |  |  |  |  |  |  |  |  |  |  |  |  |  |  |  |  |  |  |  |  |  |  |  |  |  |  |  |  |  |  |  |  |  |  |  |  |  |  |  |  |  |  |  |  |  |  |  |  |  |  |  |  |  |  |  |  |  |  |  | | --- | --- | --- | --- | --- | --- | --- | --- | --- | --- | --- | --- | --- | --- | --- | --- | --- | --- | --- | --- | --- | --- | --- | --- | --- | --- | --- | --- | --- | --- | --- | --- | --- | --- | --- | --- | --- | --- | --- | --- | --- | --- | --- | --- | --- | --- | --- | --- | --- | --- | --- | --- | --- | --- | --- | --- | --- | --- | --- | --- | --- | --- | --- | --- | --- | --- | --- | --- | --- | --- | --- | --- | --- | --- | --- | --- | --- | --- | --- | --- | --- | --- | --- | --- | --- | --- | --- | --- | --- | --- | | 0.15 125.145 564.936 1.32555 1.19678|  |  |  |  |  |  |  |  |  |  |  |  |  |  |  |  |  |  |  |  |  |  |  |  |  |  |  |  |  |  |  |  |  |  |  |  |  |  |  |  |  |  |  |  |  |  |  |  |  |  |  |  |  |  |  |  |  |  |  |  |  |  |  |  |  |  |  |  |  |  |  |  |  |  |  |  |  |  |  |  |  |  |  |  |  | | --- | --- | --- | --- | --- | --- | --- | --- | --- | --- | --- | --- | --- | --- | --- | --- | --- | --- | --- | --- | --- | --- | --- | --- | --- | --- | --- | --- | --- | --- | --- | --- | --- | --- | --- | --- | --- | --- | --- | --- | --- | --- | --- | --- | --- | --- | --- | --- | --- | --- | --- | --- | --- | --- | --- | --- | --- | --- | --- | --- | --- | --- | --- | --- | --- | --- | --- | --- | --- | --- | --- | --- | --- | --- | --- | --- | --- | --- | --- | --- | --- | --- | --- | --- | --- | | 0.2 71.3366 330.109 1.51289 1.40017|  |  |  |  |  |  |  |  |  |  |  |  |  |  |  |  |  |  |  |  |  |  |  |  |  |  |  |  |  |  |  |  |  |  |  |  |  |  |  |  |  |  |  |  |  |  |  |  |  |  |  |  |  |  |  |  |  |  |  |  |  |  |  |  |  |  |  |  |  |  |  |  |  |  |  |  |  |  |  |  | | --- | --- | --- | --- | --- | --- | --- | --- | --- | --- | --- | --- | --- | --- | --- | --- | --- | --- | --- | --- | --- | --- | --- | --- | --- | --- | --- | --- | --- | --- | --- | --- | --- | --- | --- | --- | --- | --- | --- | --- | --- | --- | --- | --- | --- | --- | --- | --- | --- | --- | --- | --- | --- | --- | --- | --- | --- | --- | --- | --- | --- | --- | --- | --- | --- | --- | --- | --- | --- | --- | --- | --- | --- | --- | --- | --- | --- | --- | --- | --- | | 0.25 44.8436 211.801 1.68742 1.59398|  |  |  |  |  |  |  |  |  |  |  |  |  |  |  |  |  |  |  |  |  |  |  |  |  |  |  |  |  |  |  |  |  |  |  |  |  |  |  |  |  |  |  |  |  |  |  |  |  |  |  |  |  |  |  |  |  |  |  |  |  |  |  |  |  |  |  |  |  |  |  |  |  |  |  | | --- | --- | --- | --- | --- | --- | --- | --- | --- | --- | --- | --- | --- | --- | --- | --- | --- | --- | --- | --- | --- | --- | --- | --- | --- | --- | --- | --- | --- | --- | --- | --- | --- | --- | --- | --- | --- | --- | --- | --- | --- | --- | --- | --- | --- | --- | --- | --- | --- | --- | --- | --- | --- | --- | --- | --- | --- | --- | --- | --- | --- | --- | --- | --- | --- | --- | --- | --- | --- | --- | --- | --- | --- | --- | --- | | 0.3 29.8933 143.736 1.85629 1.78512|  |  |  |  |  |  |  |  |  |  |  |  |  |  |  |  |  |  |  |  |  |  |  |  |  |  |  |  |  |  |  |  |  |  |  |  |  |  |  |  |  |  |  |  |  |  |  |  |  |  |  |  |  |  |  |  |  |  |  |  |  |  |  |  |  |  |  |  |  |  | | --- | --- | --- | --- | --- | --- | --- | --- | --- | --- | --- | --- | --- | --- | --- | --- | --- | --- | --- | --- | --- | --- | --- | --- | --- | --- | --- | --- | --- | --- | --- | --- | --- | --- | --- | --- | --- | --- | --- | --- | --- | --- | --- | --- | --- | --- | --- | --- | --- | --- | --- | --- | --- | --- | --- | --- | --- | --- | --- | --- | --- | --- | --- | --- | --- | --- | --- | --- | --- | --- | | 0.35 20.6827 101.076 2.02427 1.97851|  |  |  |  |  |  |  |  |  |  |  |  |  |  |  |  |  |  |  |  |  |  |  |  |  |  |  |  |  |  |  |  |  |  |  |  |  |  |  |  |  |  |  |  |  |  |  |  |  |  |  |  |  |  |  |  |  |  |  |  |  |  |  |  |  | | --- | --- | --- | --- | --- | --- | --- | --- | --- | --- | --- | --- | --- | --- | --- | --- | --- | --- | --- | --- | --- | --- | --- | --- | --- | --- | --- | --- | --- | --- | --- | --- | --- | --- | --- | --- | --- | --- | --- | --- | --- | --- | --- | --- | --- | --- | --- | --- | --- | --- | --- | --- | --- | --- | --- | --- | --- | --- | --- | --- | --- | --- | --- | --- | --- | | 0.4 14.6531 72.7054 2.19519 2.17840|  |  |  |  |  |  |  |  |  |  |  |  |  |  |  |  |  |  |  |  |  |  |  |  |  |  |  |  |  |  |  |  |  |  |  |  |  |  |  |  |  |  |  |  |  |  |  |  |  |  |  |  |  |  |  |  |  |  |  |  | | --- | --- | --- | --- | --- | --- | --- | --- | --- | --- | --- | --- | --- | --- | --- | --- | --- | --- | --- | --- | --- | --- | --- | --- | --- | --- | --- | --- | --- | --- | --- | --- | --- | --- | --- | --- | --- | --- | --- | --- | --- | --- | --- | --- | --- | --- | --- | --- | --- | --- | --- | --- | --- | --- | --- | --- | --- | --- | --- | --- | | 0.45 10.5295 53.0112 2.37261 2.38901|  |  |  |  |  |  |  |  |  |  |  |  |  |  |  |  |  |  |  |  |  |  |  |  |  |  |  |  |  |  |  |  |  |  |  |  |  |  |  |  |  |  |  |  |  |  |  |  |  |  |  |  |  |  |  | | --- | --- | --- | --- | --- | --- | --- | --- | --- | --- | --- | --- | --- | --- | --- | --- | --- | --- | --- | --- | --- | --- | --- | --- | --- | --- | --- | --- | --- | --- | --- | --- | --- | --- | --- | --- | --- | --- | --- | --- | --- | --- | --- | --- | --- | --- | --- | --- | --- | --- | --- | --- | --- | --- | --- | | 0.5 7.61682 38.8984 2.56036 2.61511|  |  |  |  |  |  |  |  |  |  |  |  |  |  |  |  |  |  |  |  |  |  |  |  |  |  |  |  |  |  |  |  |  |  |  |  |  |  |  |  |  |  |  |  |  |  |  |  |  |  | | --- | --- | --- | --- | --- | --- | --- | --- | --- | --- | --- | --- | --- | --- | --- | --- | --- | --- | --- | --- | --- | --- | --- | --- | --- | --- | --- | --- | --- | --- | --- | --- | --- | --- | --- | --- | --- | --- | --- | --- | --- | --- | --- | --- | --- | --- | --- | --- | --- | --- | | 0.55 5.50985 28.5428 2.76298 2.86262|  |  |  |  |  |  |  |  |  |  |  |  |  |  |  |  |  |  |  |  |  |  |  |  |  |  |  |  |  |  |  |  |  |  |  |  |  |  |  |  |  |  |  |  |  | | --- | --- | --- | --- | --- | --- | --- | --- | --- | --- | --- | --- | --- | --- | --- | --- | --- | --- | --- | --- | --- | --- | --- | --- | --- | --- | --- | --- | --- | --- | --- | --- | --- | --- | --- | --- | --- | --- | --- | --- | --- | --- | --- | --- | --- | | 0.6 3.95928 20.8112 2.98629 3.13937|  |  |  |  |  |  |  |  |  |  |  |  |  |  |  |  |  |  |  |  |  |  |  |  |  |  |  |  |  |  |  |  |  |  |  |  |  |  |  |  | | --- | --- | --- | --- | --- | --- | --- | --- | --- | --- | --- | --- | --- | --- | --- | --- | --- | --- | --- | --- | --- | --- | --- | --- | --- | --- | --- | --- | --- | --- | --- | --- | --- | --- | --- | --- | --- | --- | --- | --- | | 0.65 2.80505 14.9698 3.23843 3.45654|  |  |  |  |  |  |  |  |  |  |  |  |  |  |  |  |  |  |  |  |  |  |  |  |  |  |  |  |  |  |  |  |  |  |  | | --- | --- | --- | --- | --- | --- | --- | --- | --- | --- | --- | --- | --- | --- | --- | --- | --- | --- | --- | --- | --- | --- | --- | --- | --- | --- | --- | --- | --- | --- | --- | --- | --- | --- | --- | | 0.7 1.94076 10.5269 3.53148 3.83101|  |  |  |  |  |  |  |  |  |  |  |  |  |  |  |  |  |  |  |  |  |  |  |  |  |  |  |  |  |  | | --- | --- | --- | --- | --- | --- | --- | --- | --- | --- | --- | --- | --- | --- | --- | --- | --- | --- | --- | --- | --- | --- | --- | --- | --- | --- | --- | --- | --- | --- | | 0.75 1.29374 7.14389 3.88490 4.29040|  |  |  |  |  |  |  |  |  |  |  |  |  |  |  |  |  |  |  |  |  |  |  |  |  | | --- | --- | --- | --- | --- | --- | --- | --- | --- | --- | --- | --- | --- | --- | --- | --- | --- | --- | --- | --- | --- | --- | --- | --- | --- | | 0.8 0.81327 4.58360 4.33308 4.88427|  |  |  |  |  |  |  |  |  |  |  |  |  |  |  |  |  |  |  |  | | --- | --- | --- | --- | --- | --- | --- | --- | --- | --- | --- | --- | --- | --- | --- | --- | --- | --- | --- | --- | | 0.85 0.46359 2.67833 4.94546 5.71434|  |  |  |  |  |  |  |  |  |  |  |  |  |  |  | | --- | --- | --- | --- | --- | --- | --- | --- | --- | --- | --- | --- | --- | --- | --- | | 0.9 0.21975 1.31201 5.89465 7.03891|  |  |  |  |  |  |  |  |  |  | | --- | --- | --- | --- | --- | --- | --- | --- | --- | --- | | 0.95 0.06581 0.41434 7.82740 9.85693|  |  |  |  |  | | --- | --- | --- | --- | --- | | 0.97 0.02790 0.18246 9.57741 12.5254 | | | | | | | | | | | | | | | | | | | | | | | | | | | | | | | | | | | | | | | | | | | | | | | | | | | | | | | | | | | | | | | | | | | | | | | | | | | | | | | | | | | | | | | | | | | | | | | | | | | |

DRI values calculated at experimental points

| Fa Dose 6 Dose 9 DRI 6 DRI 9 | | | | |
| --- | --- | --- | --- | --- |
| 0.631 3.20463 17.0022 4.00579 4.25056|  |  |  |  |  |  |  |  |  |  |  |  |  |  |  |  |  |  |  |  |  |  |  |  |  | | --- | --- | --- | --- | --- | --- | --- | --- | --- | --- | --- | --- | --- | --- | --- | --- | --- | --- | --- | --- | --- | --- | --- | --- | --- | | 0.5795 4.53948 23.7176 2.83718 2.96470|  |  |  |  |  |  |  |  |  |  |  |  |  |  |  |  |  |  |  |  | | --- | --- | --- | --- | --- | --- | --- | --- | --- | --- | --- | --- | --- | --- | --- | --- | --- | --- | --- | --- | | 0.542 5.80441 29.9997 1.81388 1.87498|  |  |  |  |  |  |  |  |  |  |  |  |  |  |  | | --- | --- | --- | --- | --- | --- | --- | --- | --- | --- | --- | --- | --- | --- | --- | | 0.432 11.8463 59.3321 1.85099 1.85413|  |  |  |  |  |  |  |  |  |  | | --- | --- | --- | --- | --- | --- | --- | --- | --- | --- | | 0.27 37.9160 180.411 2.96219 2.81892|  |  |  |  |  | | --- | --- | --- | --- | --- | | 0.265 39.5099 187.654 1.54335 1.46605 | | | | | | | | | | | | | | | | | | | | | | | | | | | | | |

---

DRI Plot for Combo: 6+9 (6+9 [1:5])  


---

Isobologram for Combo: 6+9 (6+9 [1:5])  


---

Summary Table

|  |  |  |  |  |  |  |  |
| --- | --- | --- | --- | --- | --- | --- | --- |
| Experiment Name: SW480|  |  |  |  |  |  | | --- | --- | --- | --- | --- | --- | | Date: |  |  |  |  | | --- | --- | --- | --- | | File Name: E:\SHP2\SHP099+MK-2206Í¶¸å\Êý¾Ý¼°´¦Àí\4-CI\20180510 RKO 480 CI\SW480\480.cse|  |  | | --- | --- | | Description  | | | | | | | |

|  |  |  |  |  |  |
| --- | --- | --- | --- | --- | --- |
| Drug: MK-2206 (6) [uM]|  |  |  |  | | --- | --- | --- | --- | | Drug: SHP099 (9) [uM]|  |  | | --- | --- | | Drug Combo: MK-2206+SHP099 (6+9) (6+9 [1:5]) | | | | | |

---

| Drug/Combo Dm m r | | | |
| --- | --- | --- | --- |
| 6 7.61682 -0.6197 -0.8849|  |  |  |  |  |  |  |  | | --- | --- | --- | --- | --- | --- | --- | --- | | 9 38.8984 -0.6483 -0.9053|  |  |  |  | | --- | --- | --- | --- | | 6+9 17.8494 -0.5017 -0.9681 | | | | | | | | | | | |

---

|  |  |  |  |  |  |  |  |  |  |
| --- | --- | --- | --- | --- | --- | --- | --- | --- | --- |
| CI values at:| Combo ED50 ED75 ED90 ED95 | | | | | | --- | --- | --- | --- | --- | | | | | |
| 6+9 0.77296 0.49049 0.31171 0.22921 | | | | |

---

Data for Fa = 0.5

| Drug/Combo CI value Dose 6 Dose 9 | | | |
| --- | --- | --- | --- |
| 6 7.61682|  |  |  |  |  |  |  |  | | --- | --- | --- | --- | --- | --- | --- | --- | | 9 38.8984|  |  |  |  | | --- | --- | --- | --- | | 6+9 0.77296 2.97489 14.8745 | | | | | | | | | | |

---

Data for Fa = 0.75

| Drug/Combo CI value Dose 6 Dose 9 | | | |
| --- | --- | --- | --- |
| 6 1.29374|  |  |  |  |  |  |  |  | | --- | --- | --- | --- | --- | --- | --- | --- | | 9 7.14389|  |  |  |  | | --- | --- | --- | --- | | 6+9 0.49049 0.33302 1.66509 | | | | | | | | | | |

---

Data for Fa = 0.9

| Drug/Combo CI value Dose 6 Dose 9 | | | |
| --- | --- | --- | --- |
| 6 0.21975|  |  |  |  |  |  |  |  | | --- | --- | --- | --- | --- | --- | --- | --- | | 9 1.31201|  |  |  |  | | --- | --- | --- | --- | | 6+9 0.31171 0.03728 0.18639 | | | | | | | | | | |

---

Data for Fa = 0.95

| Drug/Combo CI value Dose 6 Dose 9 | | | |
| --- | --- | --- | --- |
| 6 0.06581|  |  |  |  |  |  |  |  | | --- | --- | --- | --- | --- | --- | --- | --- | | 9 0.41434|  |  |  |  | | --- | --- | --- | --- | | 6+9 0.22921 0.00841 0.04204 | | | | | | | | | | |

---

Data for Fa = 0.97

| Drug/Combo CI value Dose 6 Dose 9 | | | |
| --- | --- | --- | --- |
| 6 0.02790|  |  |  |  |  |  |  |  | | --- | --- | --- | --- | --- | --- | --- | --- | | 9 0.18246|  |  |  |  | | --- | --- | --- | --- | | 6+9 0.18425 0.00291 0.01457 | | | | | | | | | | |
