# Supplementary material for: Therapeutic Suppression of FAK-AKT Signaling Overcomes Resistance to SHP2 Inhibition in Colorectal Carcinoma
Source: Front Pharmacol. 2021 Nov 1;12:739501. doi: 10.3389/fphar.2021.739501 (PMC8591248; doi:10.3389/fphar.2021.739501)
Supplement: Supplementary file 11 [file DataSheet6.ZIP › Figure3/Figure3B/SW620/SW620-report/report.html]

CompuSyn Report


CompuSyn Report

|  |  |  |  |  |  |  |  |
| --- | --- | --- | --- | --- | --- | --- | --- |
| Experiment Name: SW620|  |  |  |  |  |  | | --- | --- | --- | --- | --- | --- | | Date: |  |  |  |  | | --- | --- | --- | --- | | File Name: K:\20180601 620 ci\SW620.cse|  |  | | --- | --- | | Description  | | | | | | | |

|  |  |  |  |  |  |
| --- | --- | --- | --- | --- | --- |
| Drug: MK2206 (6) [uM]|  |  |  |  | | --- | --- | --- | --- | | Drug: SHP099 (9) [uM]|  |  | | --- | --- | | Drug Combo: MK2206+SHP099 (6+9) (6+9 [1:8]) | | | | | |

---

Data for Drug: 6 [uM]

| Dose Effect | |
| --- | --- |
| 0.625 0.68|  |  |  |  |  |  |  |  | | --- | --- | --- | --- | --- | --- | --- | --- | | 1.25 0.628|  |  |  |  |  |  | | --- | --- | --- | --- | --- | --- | | 2.5 0.602|  |  |  |  | | --- | --- | --- | --- | | 10.0 0.394|  |  | | --- | --- | | 20.0 0.197 | | | | | | | | | |

5 data points entered.

|  |  |  |  |  |  |  |  |  |  |
| --- | --- | --- | --- | --- | --- | --- | --- | --- | --- |
| X-int: 0.49713|  |  |  |  |  |  |  |  | | --- | --- | --- | --- | --- | --- | --- | --- | | Y-int: 0.29397 +/- 0.08091|  |  |  |  |  |  | | --- | --- | --- | --- | --- | --- | | m: -0.5913 +/- 0.10620|  |  |  |  | | --- | --- | --- | --- | | Dm: 3.14147|  |  | | --- | --- | | r: -0.9549 | | | | | | | | | |

---

Data for Drug: 9 [uM]

| Dose Effect | |
| --- | --- |
| 5.0 0.68|  |  |  |  |  |  |  |  |  |  | | --- | --- | --- | --- | --- | --- | --- | --- | --- | --- | | 10.0 0.616|  |  |  |  |  |  |  |  | | --- | --- | --- | --- | --- | --- | --- | --- | | 20.0 0.554|  |  |  |  |  |  | | --- | --- | --- | --- | --- | --- | | 40.0 0.548|  |  |  |  | | --- | --- | --- | --- | | 160.0 0.206|  |  | | --- | --- | | 80.0 0.524 | | | | | | | | | | | |

6 data points entered.

|  |  |  |  |  |  |  |  |  |  |
| --- | --- | --- | --- | --- | --- | --- | --- | --- | --- |
| X-int: 1.50913|  |  |  |  |  |  |  |  | | --- | --- | --- | --- | --- | --- | --- | --- | | Y-int: 0.72587 +/- 0.22775|  |  |  |  |  |  | | --- | --- | --- | --- | --- | --- | | m: -0.4810 +/- 0.14790|  |  |  |  | | --- | --- | --- | --- | | Dm: 32.2949|  |  | | --- | --- | | r: -0.8518 | | | | | | | | | |

---

Data for Drug Combo: 6+9 (6+9 [1:8])

| Dose A Effect | |
| --- | --- |
| 0.625+ 0.57|  |  |  |  |  |  |  |  |  |  | | --- | --- | --- | --- | --- | --- | --- | --- | --- | --- | | 1.25+ 0.519|  |  |  |  |  |  |  |  | | --- | --- | --- | --- | --- | --- | --- | --- | | 2.5+ 0.427|  |  |  |  |  |  | | --- | --- | --- | --- | --- | --- | | 5.0+ 0.344|  |  |  |  | | --- | --- | --- | --- | | 10.0+ 0.232|  |  | | --- | --- | | 20.0+ 0.21 | | | | | | | | | | | |

6 data points entered.

|  |  |  |  |  |  |  |  |  |  |
| --- | --- | --- | --- | --- | --- | --- | --- | --- | --- |
| X-int: 1.05613|  |  |  |  |  |  |  |  | | --- | --- | --- | --- | --- | --- | --- | --- | | Y-int: 0.53131 +/- 0.05705|  |  |  |  |  |  | | --- | --- | --- | --- | --- | --- | | m: -0.5031 +/- 0.03592|  |  |  |  | | --- | --- | --- | --- | | Dm: 11.3797|  |  | | --- | --- | | r: -0.9900 | | | | | | | | | |

---

Dose-Effect Curve  


---

Median-Effect Plot  


---

CI Data for Drug Combo: 6+9 (6+9 [1:8])

| Fa CI Value Total Dose | | |
| --- | --- | --- |
| 0.05 1.20356 3963.04|  |  |  |  |  |  |  |  |  |  |  |  |  |  |  |  |  |  |  |  |  |  |  |  |  |  |  |  |  |  |  |  |  |  |  |  |  |  |  |  |  |  |  |  |  |  |  |  |  |  |  |  |  |  |  |  |  | | --- | --- | --- | --- | --- | --- | --- | --- | --- | --- | --- | --- | --- | --- | --- | --- | --- | --- | --- | --- | --- | --- | --- | --- | --- | --- | --- | --- | --- | --- | --- | --- | --- | --- | --- | --- | --- | --- | --- | --- | --- | --- | --- | --- | --- | --- | --- | --- | --- | --- | --- | --- | --- | --- | --- | --- | --- | | 0.1 1.02875 897.363|  |  |  |  |  |  |  |  |  |  |  |  |  |  |  |  |  |  |  |  |  |  |  |  |  |  |  |  |  |  |  |  |  |  |  |  |  |  |  |  |  |  |  |  |  |  |  |  |  |  |  |  |  |  | | --- | --- | --- | --- | --- | --- | --- | --- | --- | --- | --- | --- | --- | --- | --- | --- | --- | --- | --- | --- | --- | --- | --- | --- | --- | --- | --- | --- | --- | --- | --- | --- | --- | --- | --- | --- | --- | --- | --- | --- | --- | --- | --- | --- | --- | --- | --- | --- | --- | --- | --- | --- | --- | --- | | 0.15 0.94073 357.759|  |  |  |  |  |  |  |  |  |  |  |  |  |  |  |  |  |  |  |  |  |  |  |  |  |  |  |  |  |  |  |  |  |  |  |  |  |  |  |  |  |  |  |  |  |  |  |  |  |  |  | | --- | --- | --- | --- | --- | --- | --- | --- | --- | --- | --- | --- | --- | --- | --- | --- | --- | --- | --- | --- | --- | --- | --- | --- | --- | --- | --- | --- | --- | --- | --- | --- | --- | --- | --- | --- | --- | --- | --- | --- | --- | --- | --- | --- | --- | --- | --- | --- | --- | --- | --- | | 0.2 0.88326 179.020|  |  |  |  |  |  |  |  |  |  |  |  |  |  |  |  |  |  |  |  |  |  |  |  |  |  |  |  |  |  |  |  |  |  |  |  |  |  |  |  |  |  |  |  |  |  |  |  | | --- | --- | --- | --- | --- | --- | --- | --- | --- | --- | --- | --- | --- | --- | --- | --- | --- | --- | --- | --- | --- | --- | --- | --- | --- | --- | --- | --- | --- | --- | --- | --- | --- | --- | --- | --- | --- | --- | --- | --- | --- | --- | --- | --- | --- | --- | --- | --- | | 0.25 0.84092 101.053|  |  |  |  |  |  |  |  |  |  |  |  |  |  |  |  |  |  |  |  |  |  |  |  |  |  |  |  |  |  |  |  |  |  |  |  |  |  |  |  |  |  |  |  |  | | --- | --- | --- | --- | --- | --- | --- | --- | --- | --- | --- | --- | --- | --- | --- | --- | --- | --- | --- | --- | --- | --- | --- | --- | --- | --- | --- | --- | --- | --- | --- | --- | --- | --- | --- | --- | --- | --- | --- | --- | --- | --- | --- | --- | --- | | 0.3 0.80743 61.3188|  |  |  |  |  |  |  |  |  |  |  |  |  |  |  |  |  |  |  |  |  |  |  |  |  |  |  |  |  |  |  |  |  |  |  |  |  |  |  |  |  |  | | --- | --- | --- | --- | --- | --- | --- | --- | --- | --- | --- | --- | --- | --- | --- | --- | --- | --- | --- | --- | --- | --- | --- | --- | --- | --- | --- | --- | --- | --- | --- | --- | --- | --- | --- | --- | --- | --- | --- | --- | --- | --- | | 0.35 0.77965 38.9530|  |  |  |  |  |  |  |  |  |  |  |  |  |  |  |  |  |  |  |  |  |  |  |  |  |  |  |  |  |  |  |  |  |  |  |  |  |  |  | | --- | --- | --- | --- | --- | --- | --- | --- | --- | --- | --- | --- | --- | --- | --- | --- | --- | --- | --- | --- | --- | --- | --- | --- | --- | --- | --- | --- | --- | --- | --- | --- | --- | --- | --- | --- | --- | --- | --- | | 0.4 0.75578 25.4780|  |  |  |  |  |  |  |  |  |  |  |  |  |  |  |  |  |  |  |  |  |  |  |  |  |  |  |  |  |  |  |  |  |  |  |  | | --- | --- | --- | --- | --- | --- | --- | --- | --- | --- | --- | --- | --- | --- | --- | --- | --- | --- | --- | --- | --- | --- | --- | --- | --- | --- | --- | --- | --- | --- | --- | --- | --- | --- | --- | --- | | 0.45 0.73471 16.9578|  |  |  |  |  |  |  |  |  |  |  |  |  |  |  |  |  |  |  |  |  |  |  |  |  |  |  |  |  |  |  |  |  | | --- | --- | --- | --- | --- | --- | --- | --- | --- | --- | --- | --- | --- | --- | --- | --- | --- | --- | --- | --- | --- | --- | --- | --- | --- | --- | --- | --- | --- | --- | --- | --- | --- | | 0.5 0.71571 11.3797|  |  |  |  |  |  |  |  |  |  |  |  |  |  |  |  |  |  |  |  |  |  |  |  |  |  |  |  |  |  | | --- | --- | --- | --- | --- | --- | --- | --- | --- | --- | --- | --- | --- | --- | --- | --- | --- | --- | --- | --- | --- | --- | --- | --- | --- | --- | --- | --- | --- | --- | | 0.55 0.69823 7.63652|  |  |  |  |  |  |  |  |  |  |  |  |  |  |  |  |  |  |  |  |  |  |  |  |  |  |  | | --- | --- | --- | --- | --- | --- | --- | --- | --- | --- | --- | --- | --- | --- | --- | --- | --- | --- | --- | --- | --- | --- | --- | --- | --- | --- | --- | | 0.6 0.68190 5.08275|  |  |  |  |  |  |  |  |  |  |  |  |  |  |  |  |  |  |  |  |  |  |  |  | | --- | --- | --- | --- | --- | --- | --- | --- | --- | --- | --- | --- | --- | --- | --- | --- | --- | --- | --- | --- | --- | --- | --- | --- | | 0.65 0.66638 3.32447|  |  |  |  |  |  |  |  |  |  |  |  |  |  |  |  |  |  |  |  |  | | --- | --- | --- | --- | --- | --- | --- | --- | --- | --- | --- | --- | --- | --- | --- | --- | --- | --- | --- | --- | --- | | 0.7 0.65143 2.11188|  |  |  |  |  |  |  |  |  |  |  |  |  |  |  |  |  |  | | --- | --- | --- | --- | --- | --- | --- | --- | --- | --- | --- | --- | --- | --- | --- | --- | --- | --- | | 0.75 0.63679 1.28148|  |  |  |  |  |  |  |  |  |  |  |  |  |  |  | | --- | --- | --- | --- | --- | --- | --- | --- | --- | --- | --- | --- | --- | --- | --- | | 0.8 0.62223 0.72337|  |  |  |  |  |  |  |  |  |  |  |  | | --- | --- | --- | --- | --- | --- | --- | --- | --- | --- | --- | --- | | 0.85 0.60753 0.36197|  |  |  |  |  |  |  |  |  | | --- | --- | --- | --- | --- | --- | --- | --- | --- | | 0.9 0.59250 0.14431|  |  |  |  |  |  | | --- | --- | --- | --- | --- | --- | | 0.95 0.57782 0.03268|  |  |  | | --- | --- | --- | | 0.97 0.57368 0.01136 | | | | | | | | | | | | | | | | | | | | | | | | | | | | | | | | | | | | | | | | | | | | | | | | | | | | | | | | | | | |

CI values for actual experimental points:

Total Dose Fa CI Value | | || 5.625 0.57 0.59862|  |  |  |  |  |  |  |  |  |  |  |  |  |  |  | | --- | --- | --- | --- | --- | --- | --- | --- | --- | --- | --- | --- | --- | --- | --- | | 11.25 0.519 0.81518|  |  |  |  |  |  |  |  |  |  |  |  | | --- | --- | --- | --- | --- | --- | --- | --- | --- | --- | --- | --- | | 22.5 0.427 0.81996|  |  |  |  |  |  |  |  |  | | --- | --- | --- | --- | --- | --- | --- | --- | --- | | 45.0 0.344 0.85789|  |  |  |  |  |  | | --- | --- | --- | --- | --- | --- | | 90.0 0.232 0.62608|  |  |  | | --- | --- | --- | | 180.0 0.21 0.99263 | | | | | | | | | | | | | | | | | |

---

Combination Index Plot  


---

DRI Data for Drug Combo: 6+9 (6+9 [1:8])

| Fa Dose 6 Dose 9 DRI 6 DRI 9 | | | | |
| --- | --- | --- | --- | --- |
| 0.05 456.705 14714.8 1.03717 4.17714|  |  |  |  |  |  |  |  |  |  |  |  |  |  |  |  |  |  |  |  |  |  |  |  |  |  |  |  |  |  |  |  |  |  |  |  |  |  |  |  |  |  |  |  |  |  |  |  |  |  |  |  |  |  |  |  |  |  |  |  |  |  |  |  |  |  |  |  |  |  |  |  |  |  |  |  |  |  |  |  |  |  |  |  |  |  |  |  |  |  |  |  |  |  |  | | --- | --- | --- | --- | --- | --- | --- | --- | --- | --- | --- | --- | --- | --- | --- | --- | --- | --- | --- | --- | --- | --- | --- | --- | --- | --- | --- | --- | --- | --- | --- | --- | --- | --- | --- | --- | --- | --- | --- | --- | --- | --- | --- | --- | --- | --- | --- | --- | --- | --- | --- | --- | --- | --- | --- | --- | --- | --- | --- | --- | --- | --- | --- | --- | --- | --- | --- | --- | --- | --- | --- | --- | --- | --- | --- | --- | --- | --- | --- | --- | --- | --- | --- | --- | --- | --- | --- | --- | --- | --- | --- | --- | --- | --- | --- | | 0.1 129.079 3112.24 1.29458 3.90173|  |  |  |  |  |  |  |  |  |  |  |  |  |  |  |  |  |  |  |  |  |  |  |  |  |  |  |  |  |  |  |  |  |  |  |  |  |  |  |  |  |  |  |  |  |  |  |  |  |  |  |  |  |  |  |  |  |  |  |  |  |  |  |  |  |  |  |  |  |  |  |  |  |  |  |  |  |  |  |  |  |  |  |  |  |  |  |  |  |  | | --- | --- | --- | --- | --- | --- | --- | --- | --- | --- | --- | --- | --- | --- | --- | --- | --- | --- | --- | --- | --- | --- | --- | --- | --- | --- | --- | --- | --- | --- | --- | --- | --- | --- | --- | --- | --- | --- | --- | --- | --- | --- | --- | --- | --- | --- | --- | --- | --- | --- | --- | --- | --- | --- | --- | --- | --- | --- | --- | --- | --- | --- | --- | --- | --- | --- | --- | --- | --- | --- | --- | --- | --- | --- | --- | --- | --- | --- | --- | --- | --- | --- | --- | --- | --- | --- | --- | --- | --- | --- | | 0.15 59.0320 1189.48 1.48504 3.74040|  |  |  |  |  |  |  |  |  |  |  |  |  |  |  |  |  |  |  |  |  |  |  |  |  |  |  |  |  |  |  |  |  |  |  |  |  |  |  |  |  |  |  |  |  |  |  |  |  |  |  |  |  |  |  |  |  |  |  |  |  |  |  |  |  |  |  |  |  |  |  |  |  |  |  |  |  |  |  |  |  |  |  |  |  | | --- | --- | --- | --- | --- | --- | --- | --- | --- | --- | --- | --- | --- | --- | --- | --- | --- | --- | --- | --- | --- | --- | --- | --- | --- | --- | --- | --- | --- | --- | --- | --- | --- | --- | --- | --- | --- | --- | --- | --- | --- | --- | --- | --- | --- | --- | --- | --- | --- | --- | --- | --- | --- | --- | --- | --- | --- | --- | --- | --- | --- | --- | --- | --- | --- | --- | --- | --- | --- | --- | --- | --- | --- | --- | --- | --- | --- | --- | --- | --- | --- | --- | --- | --- | --- | | 0.2 32.7550 576.581 1.64671 3.62335|  |  |  |  |  |  |  |  |  |  |  |  |  |  |  |  |  |  |  |  |  |  |  |  |  |  |  |  |  |  |  |  |  |  |  |  |  |  |  |  |  |  |  |  |  |  |  |  |  |  |  |  |  |  |  |  |  |  |  |  |  |  |  |  |  |  |  |  |  |  |  |  |  |  |  |  |  |  |  |  | | --- | --- | --- | --- | --- | --- | --- | --- | --- | --- | --- | --- | --- | --- | --- | --- | --- | --- | --- | --- | --- | --- | --- | --- | --- | --- | --- | --- | --- | --- | --- | --- | --- | --- | --- | --- | --- | --- | --- | --- | --- | --- | --- | --- | --- | --- | --- | --- | --- | --- | --- | --- | --- | --- | --- | --- | --- | --- | --- | --- | --- | --- | --- | --- | --- | --- | --- | --- | --- | --- | --- | --- | --- | --- | --- | --- | --- | --- | --- | --- | | 0.25 20.1370 317.032 1.79344 3.52944|  |  |  |  |  |  |  |  |  |  |  |  |  |  |  |  |  |  |  |  |  |  |  |  |  |  |  |  |  |  |  |  |  |  |  |  |  |  |  |  |  |  |  |  |  |  |  |  |  |  |  |  |  |  |  |  |  |  |  |  |  |  |  |  |  |  |  |  |  |  |  |  |  |  |  | | --- | --- | --- | --- | --- | --- | --- | --- | --- | --- | --- | --- | --- | --- | --- | --- | --- | --- | --- | --- | --- | --- | --- | --- | --- | --- | --- | --- | --- | --- | --- | --- | --- | --- | --- | --- | --- | --- | --- | --- | --- | --- | --- | --- | --- | --- | --- | --- | --- | --- | --- | --- | --- | --- | --- | --- | --- | --- | --- | --- | --- | --- | --- | --- | --- | --- | --- | --- | --- | --- | --- | --- | --- | --- | --- | | 0.3 13.1650 188.011 1.93227 3.44940|  |  |  |  |  |  |  |  |  |  |  |  |  |  |  |  |  |  |  |  |  |  |  |  |  |  |  |  |  |  |  |  |  |  |  |  |  |  |  |  |  |  |  |  |  |  |  |  |  |  |  |  |  |  |  |  |  |  |  |  |  |  |  |  |  |  |  |  |  |  | | --- | --- | --- | --- | --- | --- | --- | --- | --- | --- | --- | --- | --- | --- | --- | --- | --- | --- | --- | --- | --- | --- | --- | --- | --- | --- | --- | --- | --- | --- | --- | --- | --- | --- | --- | --- | --- | --- | --- | --- | --- | --- | --- | --- | --- | --- | --- | --- | --- | --- | --- | --- | --- | --- | --- | --- | --- | --- | --- | --- | --- | --- | --- | --- | --- | --- | --- | --- | --- | --- | | 0.35 8.94907 116.972 2.06766 3.37827|  |  |  |  |  |  |  |  |  |  |  |  |  |  |  |  |  |  |  |  |  |  |  |  |  |  |  |  |  |  |  |  |  |  |  |  |  |  |  |  |  |  |  |  |  |  |  |  |  |  |  |  |  |  |  |  |  |  |  |  |  |  |  |  |  | | --- | --- | --- | --- | --- | --- | --- | --- | --- | --- | --- | --- | --- | --- | --- | --- | --- | --- | --- | --- | --- | --- | --- | --- | --- | --- | --- | --- | --- | --- | --- | --- | --- | --- | --- | --- | --- | --- | --- | --- | --- | --- | --- | --- | --- | --- | --- | --- | --- | --- | --- | --- | --- | --- | --- | --- | --- | --- | --- | --- | --- | --- | --- | --- | --- | | 0.4 6.23622 75.0310 2.20292 3.31305|  |  |  |  |  |  |  |  |  |  |  |  |  |  |  |  |  |  |  |  |  |  |  |  |  |  |  |  |  |  |  |  |  |  |  |  |  |  |  |  |  |  |  |  |  |  |  |  |  |  |  |  |  |  |  |  |  |  |  |  | | --- | --- | --- | --- | --- | --- | --- | --- | --- | --- | --- | --- | --- | --- | --- | --- | --- | --- | --- | --- | --- | --- | --- | --- | --- | --- | --- | --- | --- | --- | --- | --- | --- | --- | --- | --- | --- | --- | --- | --- | --- | --- | --- | --- | --- | --- | --- | --- | --- | --- | --- | --- | --- | --- | --- | --- | --- | --- | --- | --- | | 0.45 4.41076 49.0146 2.34092 3.25170|  |  |  |  |  |  |  |  |  |  |  |  |  |  |  |  |  |  |  |  |  |  |  |  |  |  |  |  |  |  |  |  |  |  |  |  |  |  |  |  |  |  |  |  |  |  |  |  |  |  |  |  |  |  |  | | --- | --- | --- | --- | --- | --- | --- | --- | --- | --- | --- | --- | --- | --- | --- | --- | --- | --- | --- | --- | --- | --- | --- | --- | --- | --- | --- | --- | --- | --- | --- | --- | --- | --- | --- | --- | --- | --- | --- | --- | --- | --- | --- | --- | --- | --- | --- | --- | --- | --- | --- | --- | --- | --- | --- | | 0.5 3.14147 32.2949 2.48453 3.19268|  |  |  |  |  |  |  |  |  |  |  |  |  |  |  |  |  |  |  |  |  |  |  |  |  |  |  |  |  |  |  |  |  |  |  |  |  |  |  |  |  |  |  |  |  |  |  |  |  |  | | --- | --- | --- | --- | --- | --- | --- | --- | --- | --- | --- | --- | --- | --- | --- | --- | --- | --- | --- | --- | --- | --- | --- | --- | --- | --- | --- | --- | --- | --- | --- | --- | --- | --- | --- | --- | --- | --- | --- | --- | --- | --- | --- | --- | --- | --- | --- | --- | --- | --- | | 0.55 2.23745 21.2786 2.63694 3.13473|  |  |  |  |  |  |  |  |  |  |  |  |  |  |  |  |  |  |  |  |  |  |  |  |  |  |  |  |  |  |  |  |  |  |  |  |  |  |  |  |  |  |  |  |  | | --- | --- | --- | --- | --- | --- | --- | --- | --- | --- | --- | --- | --- | --- | --- | --- | --- | --- | --- | --- | --- | --- | --- | --- | --- | --- | --- | --- | --- | --- | --- | --- | --- | --- | --- | --- | --- | --- | --- | --- | --- | --- | --- | --- | --- | | 0.6 1.58250 13.9004 2.80213 3.07667|  |  |  |  |  |  |  |  |  |  |  |  |  |  |  |  |  |  |  |  |  |  |  |  |  |  |  |  |  |  |  |  |  |  |  |  |  |  |  |  | | --- | --- | --- | --- | --- | --- | --- | --- | --- | --- | --- | --- | --- | --- | --- | --- | --- | --- | --- | --- | --- | --- | --- | --- | --- | --- | --- | --- | --- | --- | --- | --- | --- | --- | --- | --- | --- | --- | --- | --- | | 0.65 1.10278 8.91631 2.98544 3.01727|  |  |  |  |  |  |  |  |  |  |  |  |  |  |  |  |  |  |  |  |  |  |  |  |  |  |  |  |  |  |  |  |  |  |  | | --- | --- | --- | --- | --- | --- | --- | --- | --- | --- | --- | --- | --- | --- | --- | --- | --- | --- | --- | --- | --- | --- | --- | --- | --- | --- | --- | --- | --- | --- | --- | --- | --- | --- | --- | | 0.7 0.74963 5.54733 3.19462 2.95506|  |  |  |  |  |  |  |  |  |  |  |  |  |  |  |  |  |  |  |  |  |  |  |  |  |  |  |  |  |  | | --- | --- | --- | --- | --- | --- | --- | --- | --- | --- | --- | --- | --- | --- | --- | --- | --- | --- | --- | --- | --- | --- | --- | --- | --- | --- | --- | --- | --- | --- | | 0.75 0.49009 3.28976 3.44193 2.88804|  |  |  |  |  |  |  |  |  |  |  |  |  |  |  |  |  |  |  |  |  |  |  |  |  | | --- | --- | --- | --- | --- | --- | --- | --- | --- | --- | --- | --- | --- | --- | --- | --- | --- | --- | --- | --- | --- | --- | --- | --- | --- | | 0.8 0.30129 1.80887 3.74860 2.81319|  |  |  |  |  |  |  |  |  |  |  |  |  |  |  |  |  |  |  |  | | --- | --- | --- | --- | --- | --- | --- | --- | --- | --- | --- | --- | --- | --- | --- | --- | --- | --- | --- | --- | | 0.85 0.16718 0.87682 4.15671 2.72516|  |  |  |  |  |  |  |  |  |  |  |  |  |  |  | | --- | --- | --- | --- | --- | --- | --- | --- | --- | --- | --- | --- | --- | --- | --- | | 0.9 0.07646 0.33512 4.76825 2.61248|  |  |  |  |  |  |  |  |  |  | | --- | --- | --- | --- | --- | --- | --- | --- | --- | --- | | 0.95 0.02161 0.07088 5.95166 2.44023|  |  |  |  |  | | --- | --- | --- | --- | --- | | 0.97 0.00879 0.02347 6.96857 2.32464 | | | | | | | | | | | | | | | | | | | | | | | | | | | | | | | | | | | | | | | | | | | | | | | | | | | | | | | | | | | | | | | | | | | | | | | | | | | | | | | | | | | | | | | | | | | | | | | | | | | |

DRI values calculated at experimental points

| Fa Dose 6 Dose 9 DRI 6 DRI 9 | | | | |
| --- | --- | --- | --- | --- |
| 0.57 1.95043 17.9739 3.12070 3.59478|  |  |  |  |  |  |  |  |  |  |  |  |  |  |  |  |  |  |  |  |  |  |  |  |  | | --- | --- | --- | --- | --- | --- | --- | --- | --- | --- | --- | --- | --- | --- | --- | --- | --- | --- | --- | --- | --- | --- | --- | --- | --- | | 0.519 2.76242 27.5726 2.20993 2.75726|  |  |  |  |  |  |  |  |  |  |  |  |  |  |  |  |  |  |  |  | | --- | --- | --- | --- | --- | --- | --- | --- | --- | --- | --- | --- | --- | --- | --- | --- | --- | --- | --- | --- | | 0.427 5.16574 59.5233 2.06629 2.97617|  |  |  |  |  |  |  |  |  |  |  |  |  |  |  | | --- | --- | --- | --- | --- | --- | --- | --- | --- | --- | --- | --- | --- | --- | --- | | 0.344 9.35892 123.593 1.87178 3.08981|  |  |  |  |  |  |  |  |  |  | | --- | --- | --- | --- | --- | --- | --- | --- | --- | --- | | 0.232 23.7844 389.034 2.37844 4.86293|  |  |  |  |  | | --- | --- | --- | --- | --- | | 0.21 29.5261 507.514 1.47631 3.17196 | | | | | | | | | | | | | | | | | | | | | | | | | | | | | |

---

DRI Plot for Combo: 6+9 (6+9 [1:8])  


---

Isobologram for Combo: 6+9 (6+9 [1:8])  


---

Summary Table

|  |  |  |  |  |  |  |  |
| --- | --- | --- | --- | --- | --- | --- | --- |
| Experiment Name: SW620|  |  |  |  |  |  | | --- | --- | --- | --- | --- | --- | | Date: |  |  |  |  | | --- | --- | --- | --- | | File Name: K:\20180601 620 ci\SW620.cse|  |  | | --- | --- | | Description  | | | | | | | |

|  |  |  |  |  |  |
| --- | --- | --- | --- | --- | --- |
| Drug: MK2206 (6) [uM]|  |  |  |  | | --- | --- | --- | --- | | Drug: SHP099 (9) [uM]|  |  | | --- | --- | | Drug Combo: MK2206+SHP099 (6+9) (6+9 [1:8]) | | | | | |

---

| Drug/Combo Dm m r | | | |
| --- | --- | --- | --- |
| 6 3.14147 -0.5913 -0.9549|  |  |  |  |  |  |  |  | | --- | --- | --- | --- | --- | --- | --- | --- | | 9 32.2949 -0.4810 -0.8518|  |  |  |  | | --- | --- | --- | --- | | 6+9 11.3797 -0.5031 -0.9900 | | | | | | | | | | | |

---

|  |  |  |  |  |  |  |  |  |  |
| --- | --- | --- | --- | --- | --- | --- | --- | --- | --- |
| CI values at:| Combo ED50 ED75 ED90 ED95 | | | | | | --- | --- | --- | --- | --- | | | | | |
| 6+9 0.71571 0.63679 0.59250 0.57782 | | | | |

---

Data for Fa = 0.5

| Drug/Combo CI value Dose 6 Dose 9 | | | |
| --- | --- | --- | --- |
| 6 3.14147|  |  |  |  |  |  |  |  | | --- | --- | --- | --- | --- | --- | --- | --- | | 9 32.2949|  |  |  |  | | --- | --- | --- | --- | | 6+9 0.71571 1.26441 10.1153 | | | | | | | | | | |

---

Data for Fa = 0.75

| Drug/Combo CI value Dose 6 Dose 9 | | | |
| --- | --- | --- | --- |
| 6 0.49009|  |  |  |  |  |  |  |  | | --- | --- | --- | --- | --- | --- | --- | --- | | 9 3.28976|  |  |  |  | | --- | --- | --- | --- | | 6+9 0.63679 0.14239 1.13910 | | | | | | | | | | |

---

Data for Fa = 0.9

| Drug/Combo CI value Dose 6 Dose 9 | | | |
| --- | --- | --- | --- |
| 6 0.07646|  |  |  |  |  |  |  |  | | --- | --- | --- | --- | --- | --- | --- | --- | | 9 0.33512|  |  |  |  | | --- | --- | --- | --- | | 6+9 0.59250 0.01603 0.12828 | | | | | | | | | | |

---

Data for Fa = 0.95

| Drug/Combo CI value Dose 6 Dose 9 | | | |
| --- | --- | --- | --- |
| 6 0.02161|  |  |  |  |  |  |  |  | | --- | --- | --- | --- | --- | --- | --- | --- | | 9 0.07088|  |  |  |  | | --- | --- | --- | --- | | 6+9 0.57782 0.00363 0.02905 | | | | | | | | | | |

---

Data for Fa = 0.97

| Drug/Combo CI value Dose 6 Dose 9 | | | |
| --- | --- | --- | --- |
| 6 0.00879|  |  |  |  |  |  |  |  | | --- | --- | --- | --- | --- | --- | --- | --- | | 9 0.02347|  |  |  |  | | --- | --- | --- | --- | | 6+9 0.57368 0.00126 0.01010 | | | | | | | | | | |
