# Supplementary material for: Therapeutic Suppression of FAK-AKT Signaling Overcomes Resistance to SHP2 Inhibition in Colorectal Carcinoma
Source: Front Pharmacol. 2021 Nov 1;12:739501. doi: 10.3389/fphar.2021.739501 (PMC8591248; doi:10.3389/fphar.2021.739501)
Supplement: Supplementary file 11 [file DataSheet6.ZIP › Figure3/Figure3D/RKO.pdf]

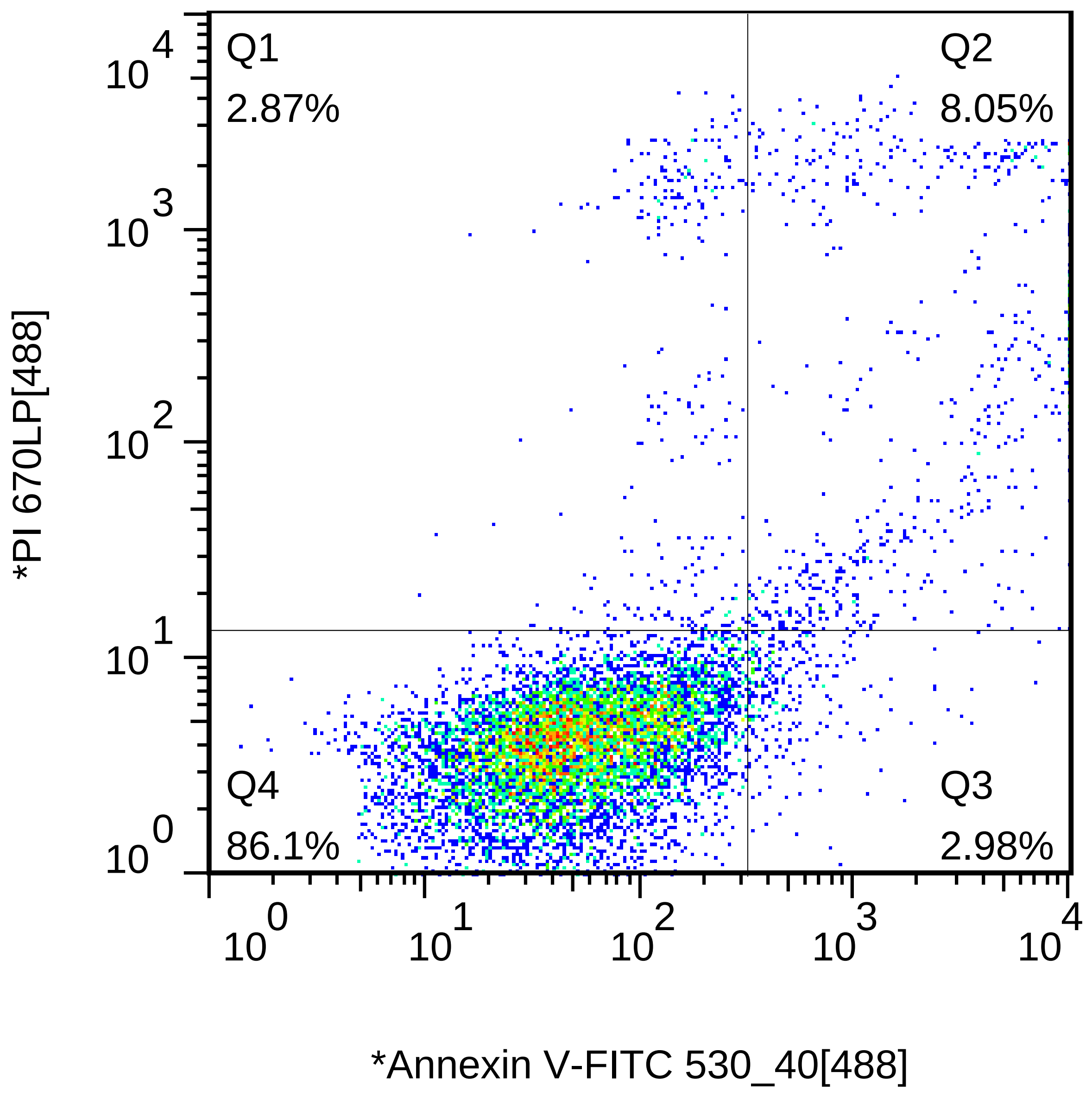

RKO DMSO.fcs
FSC, SSC subset
9608

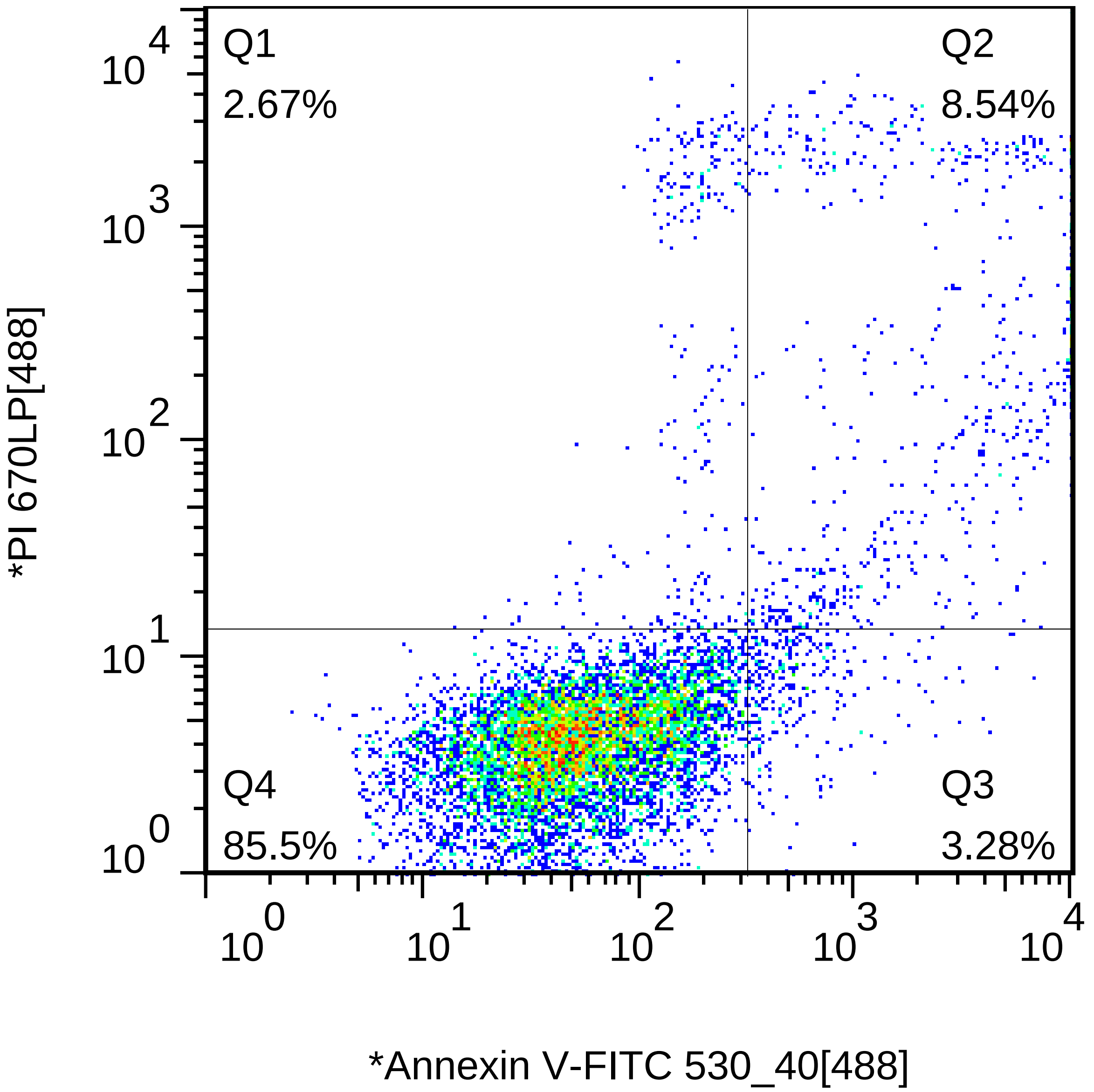

RKO DMSO_001.fcs
FSC, SSC subset
9630

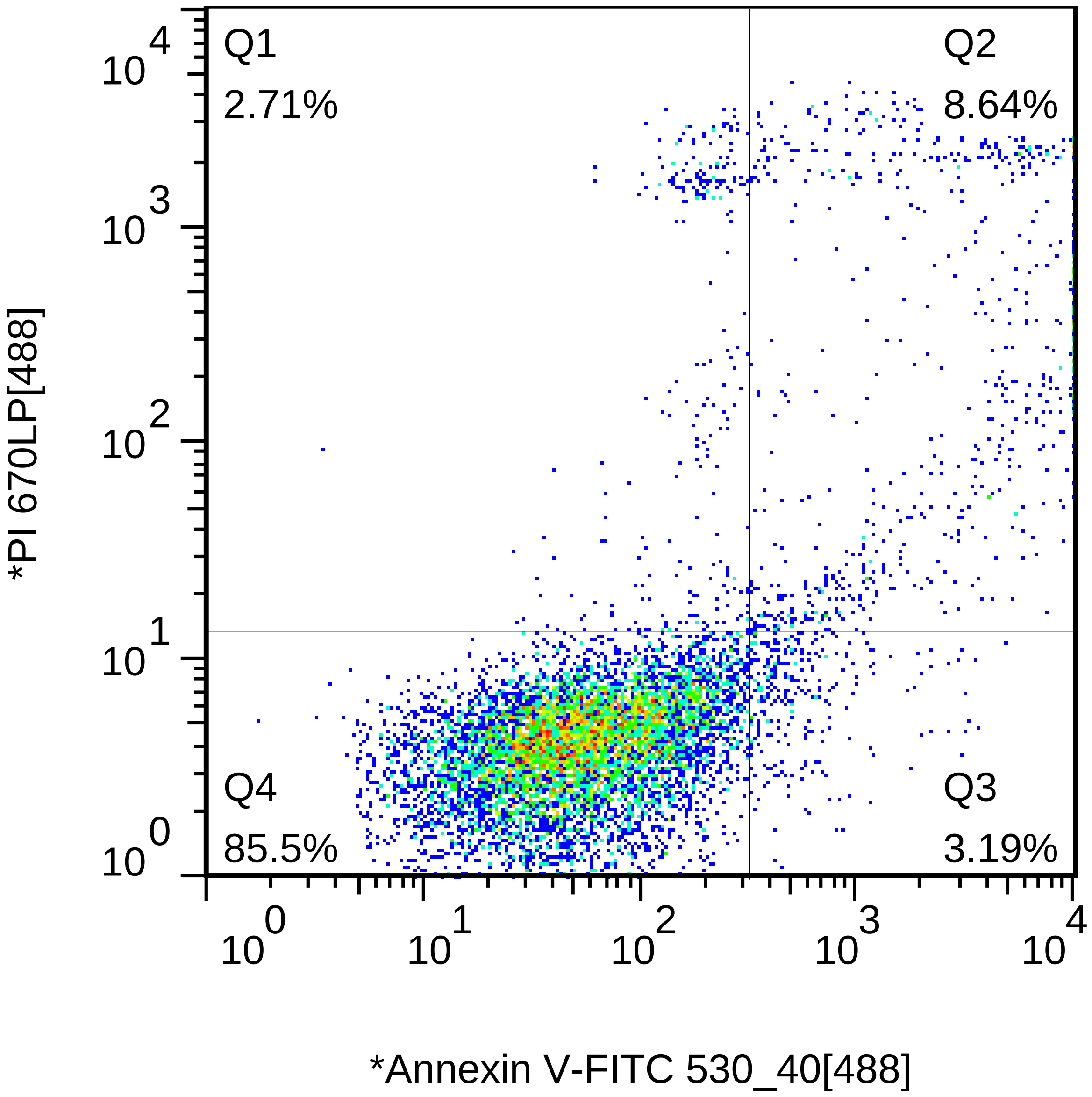

RKO DMSO_002.fcs
FSC, SSC subset
9668

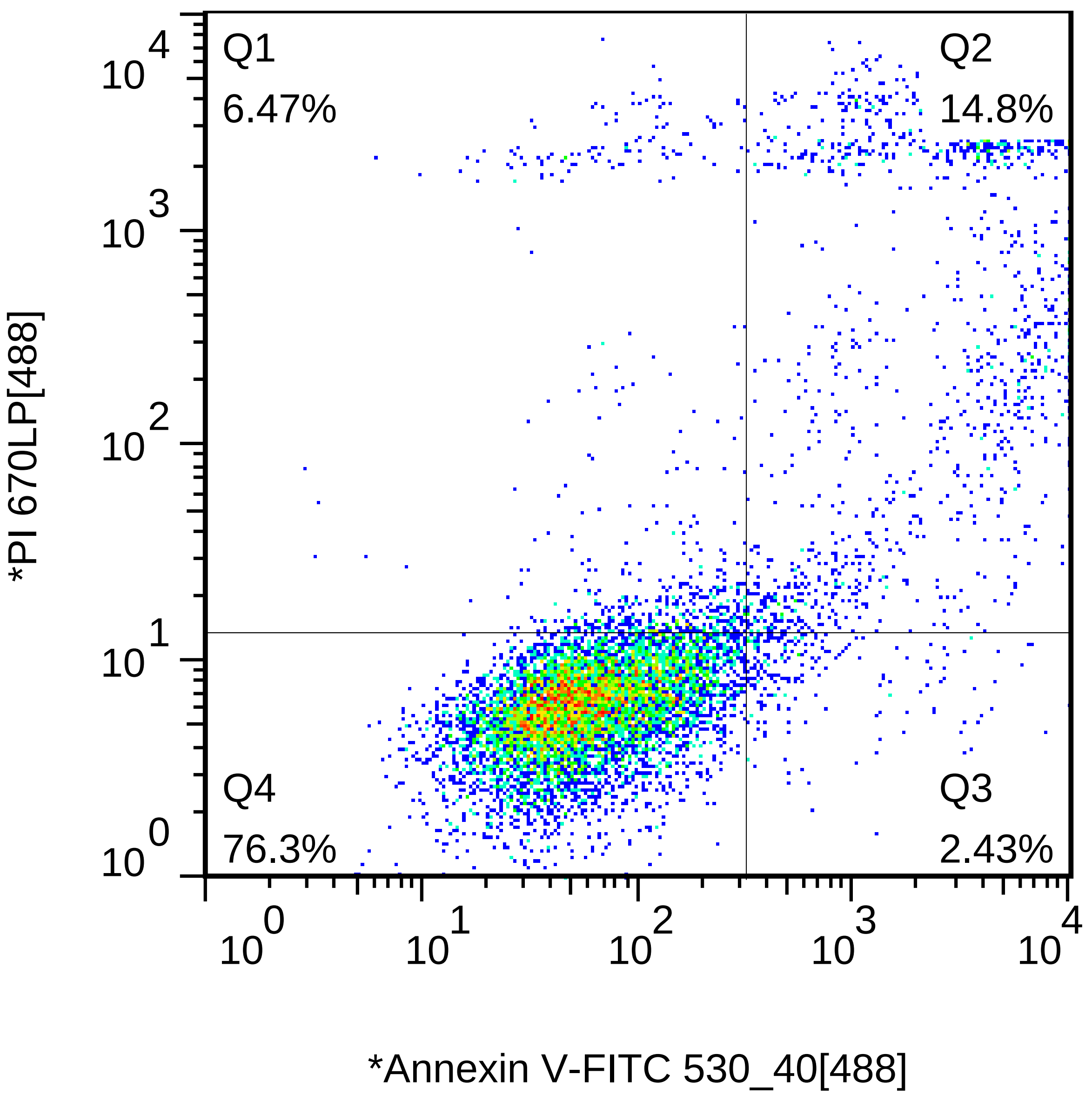

RKO-9+6_001.fcs
FSC, SSC subset
9491

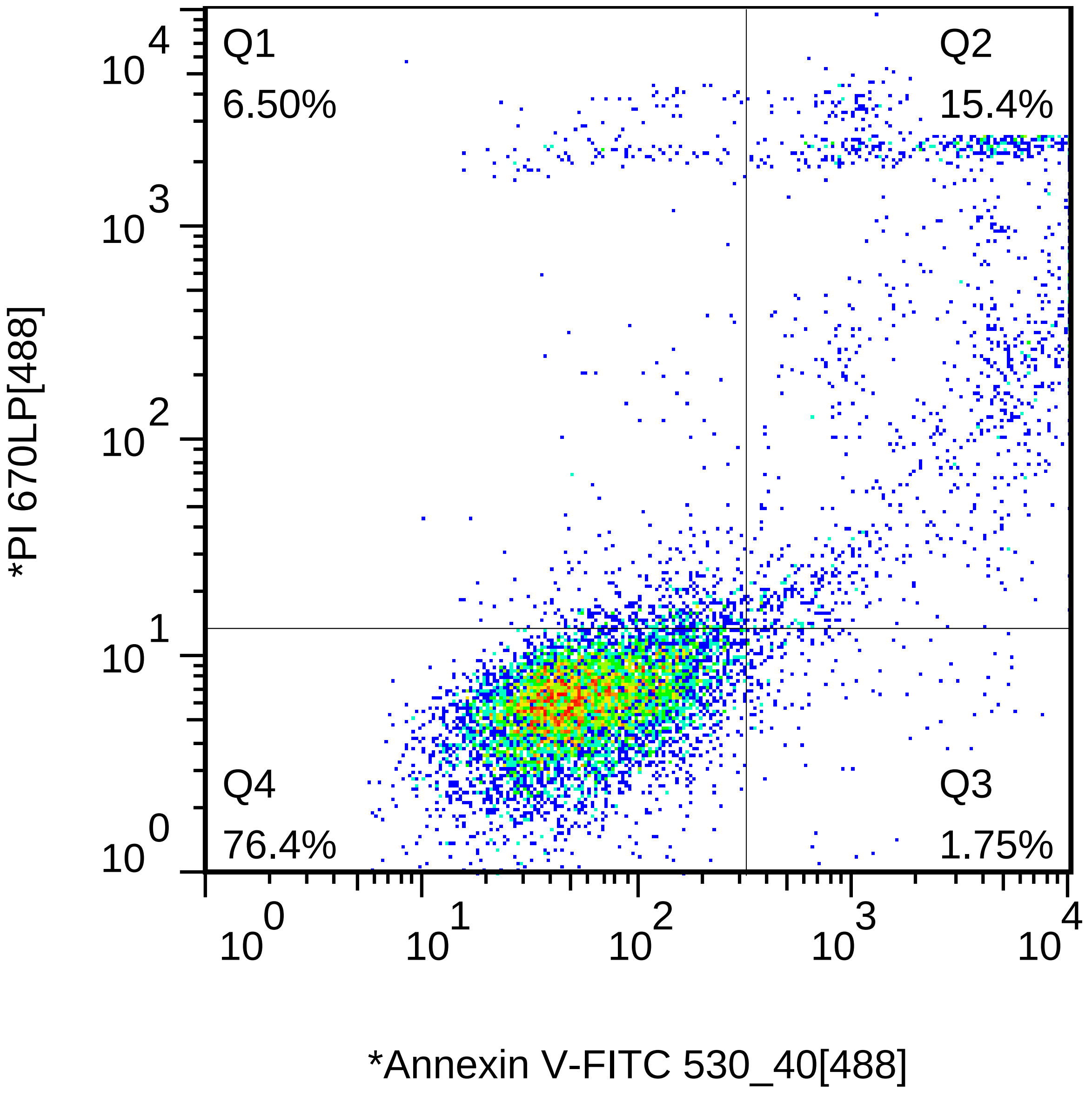

RKO-9+6_002.fcs
FSC, SSC subset
9556

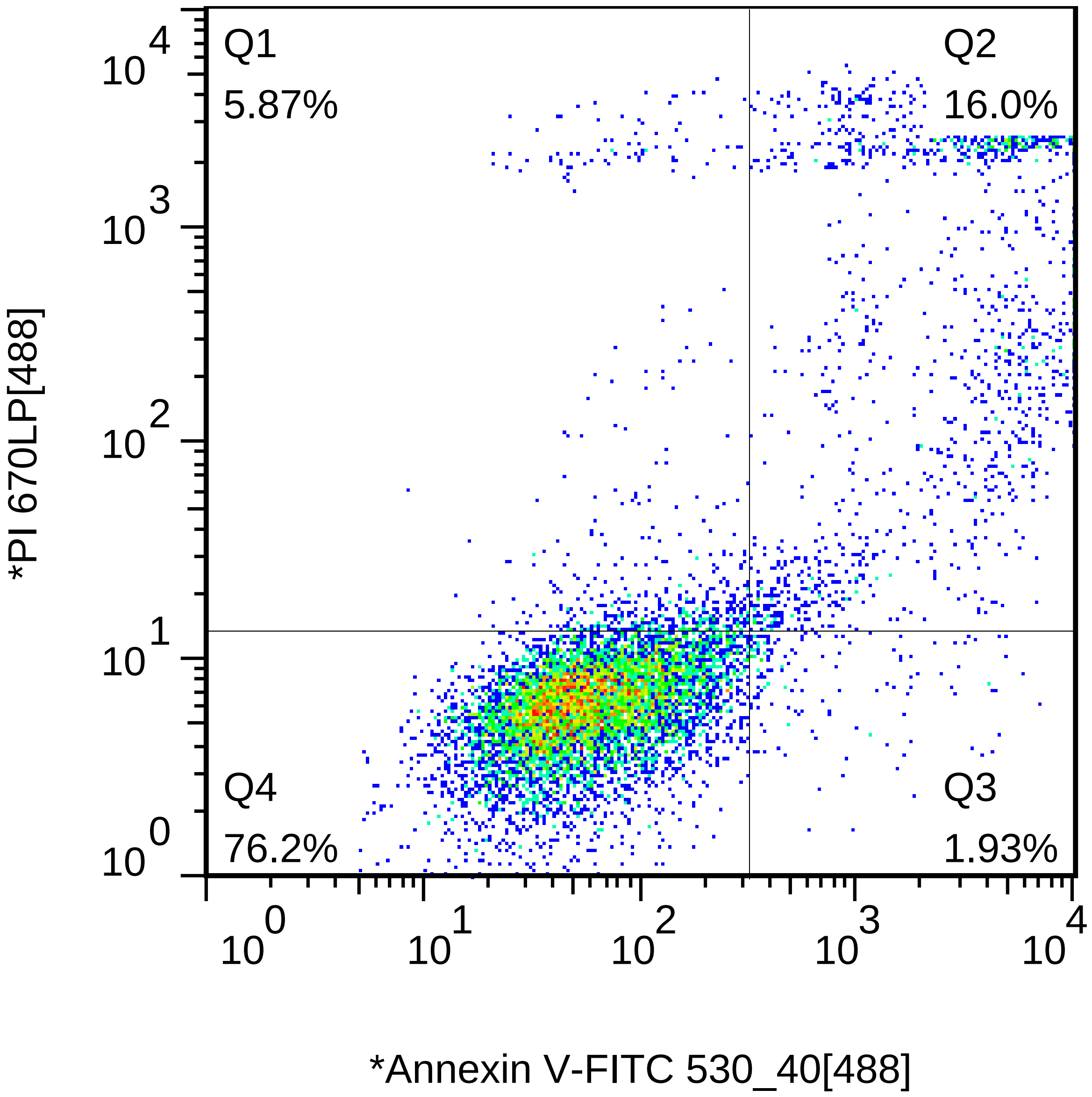

RKO-9+6_003.fcs
FSC, SSC subset
9516

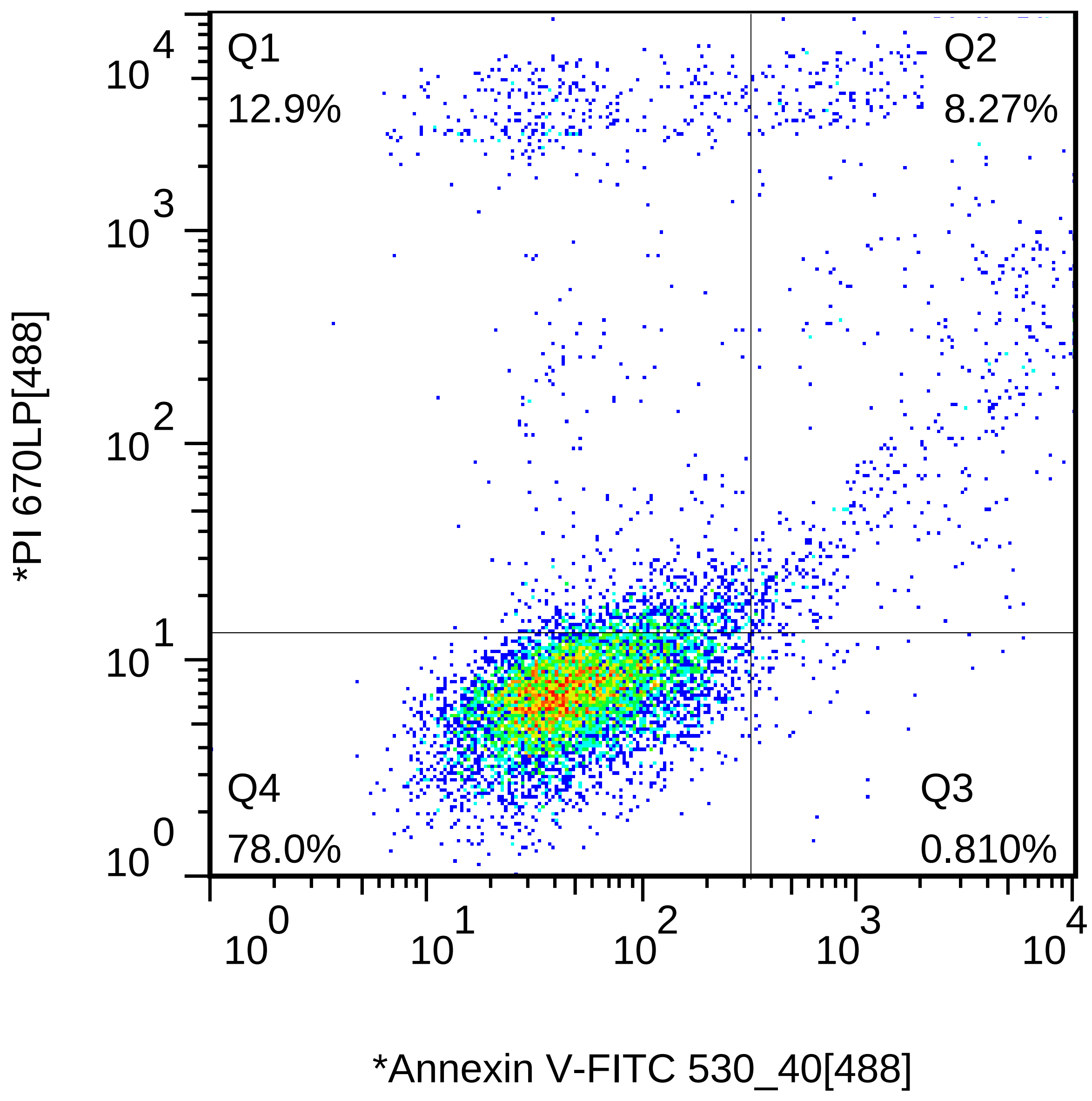

RKO-099_001.fcs
FSC, SSC subset
9506

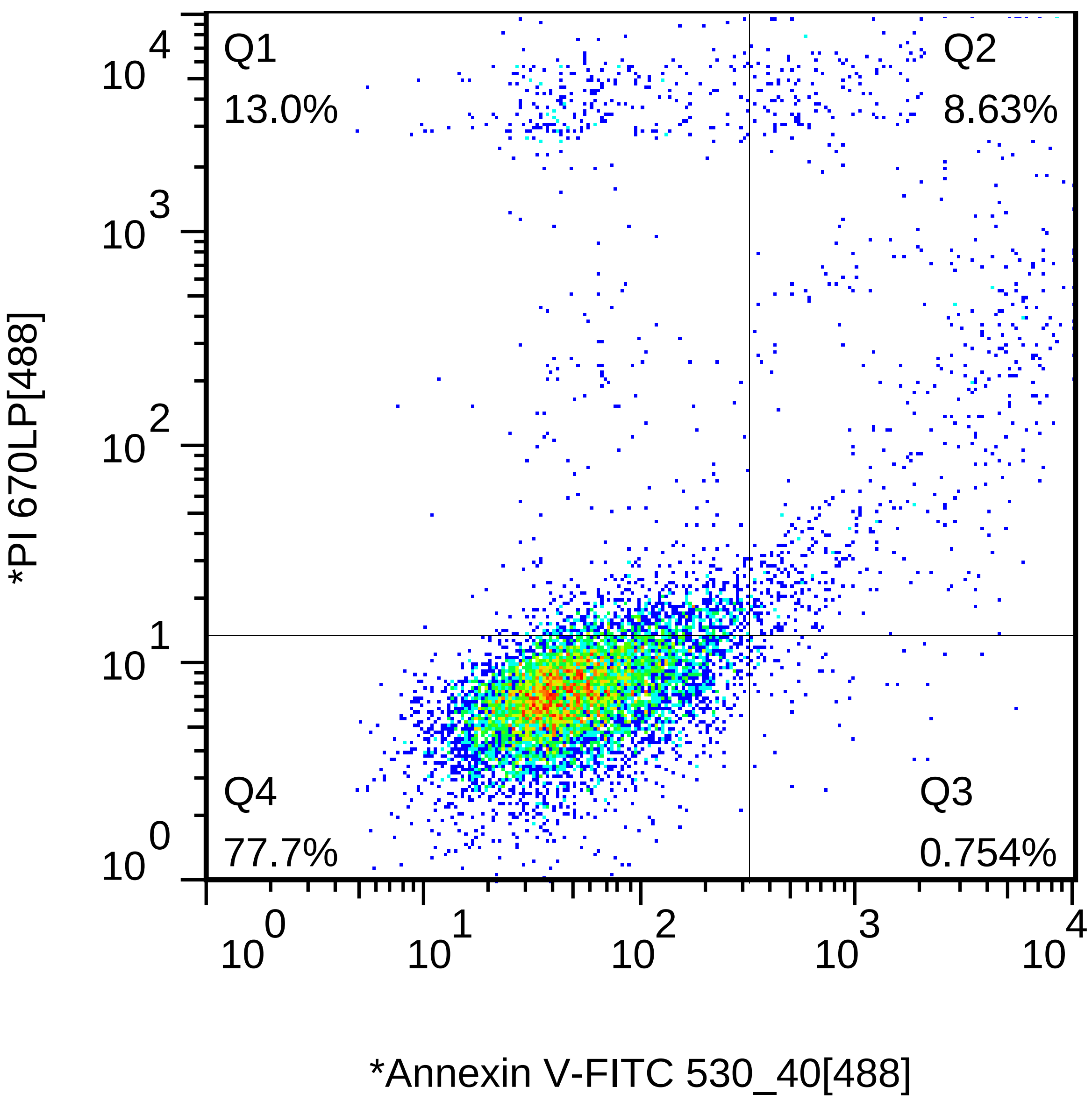

RKO-099_003.fcs
FSC, SSC subset
9544

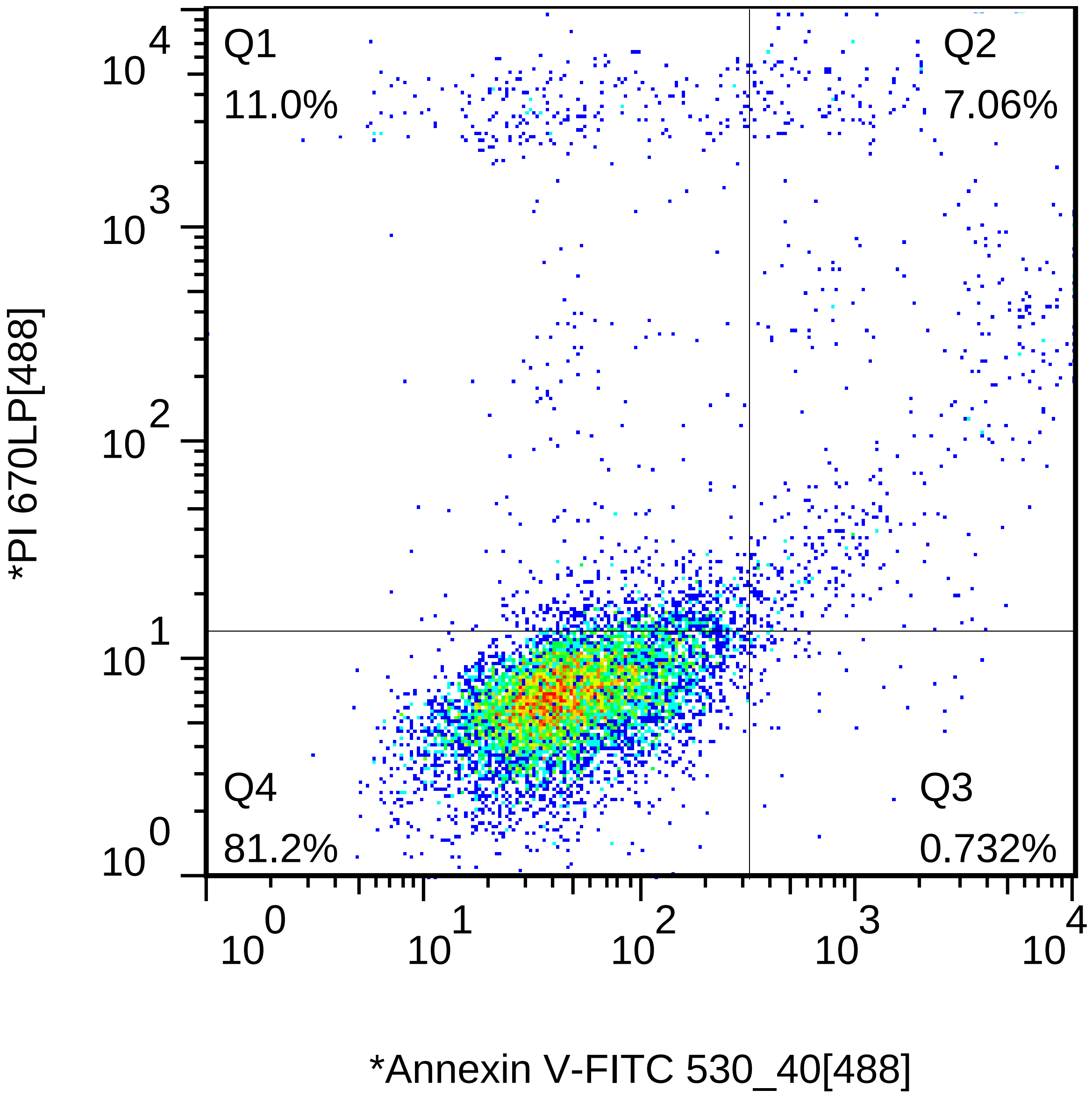

RKO-099.fcs
FSC, SSC subset
9431

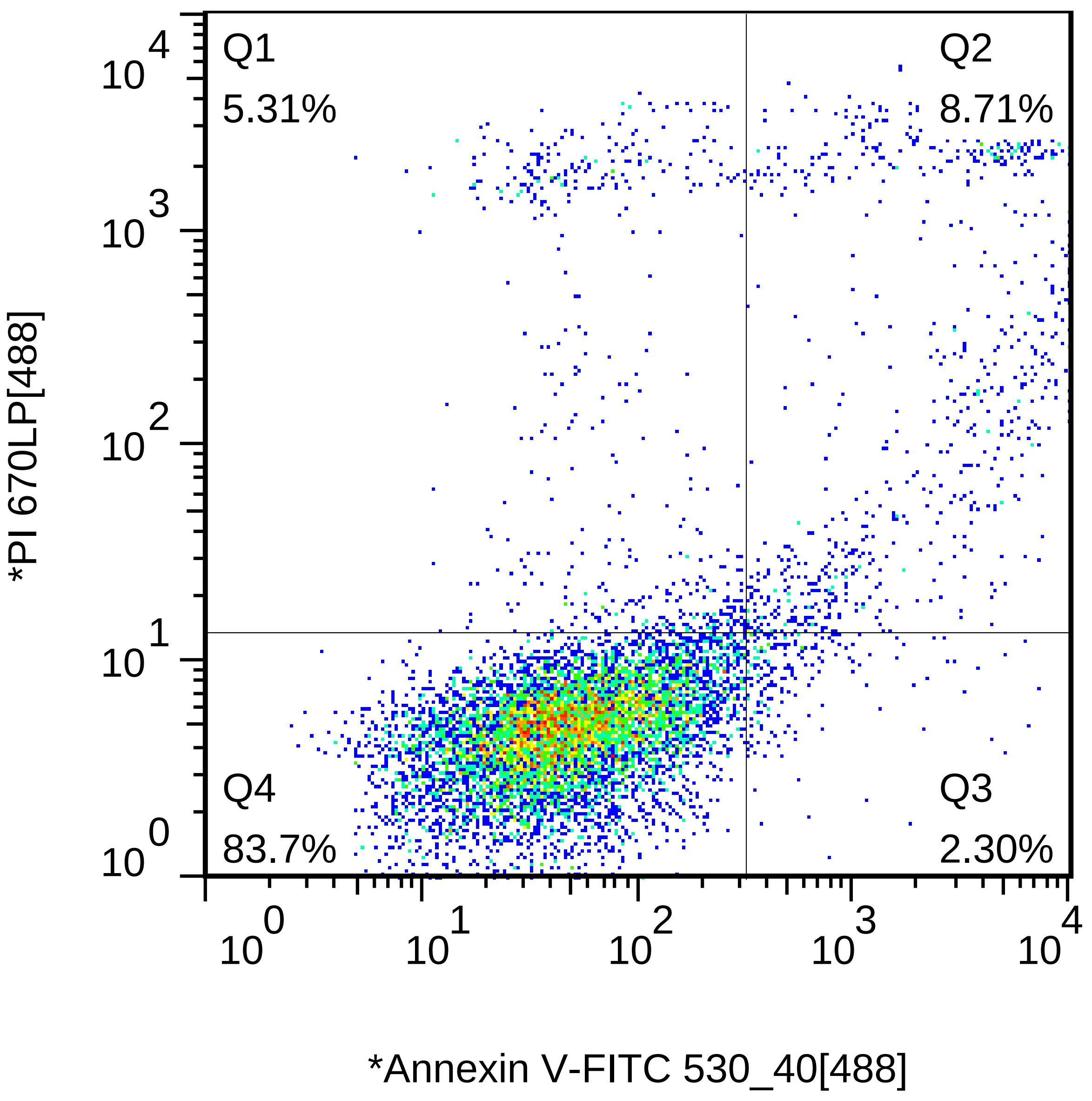

RKO-2206_001.fcs
FSC, SSC subset
9353

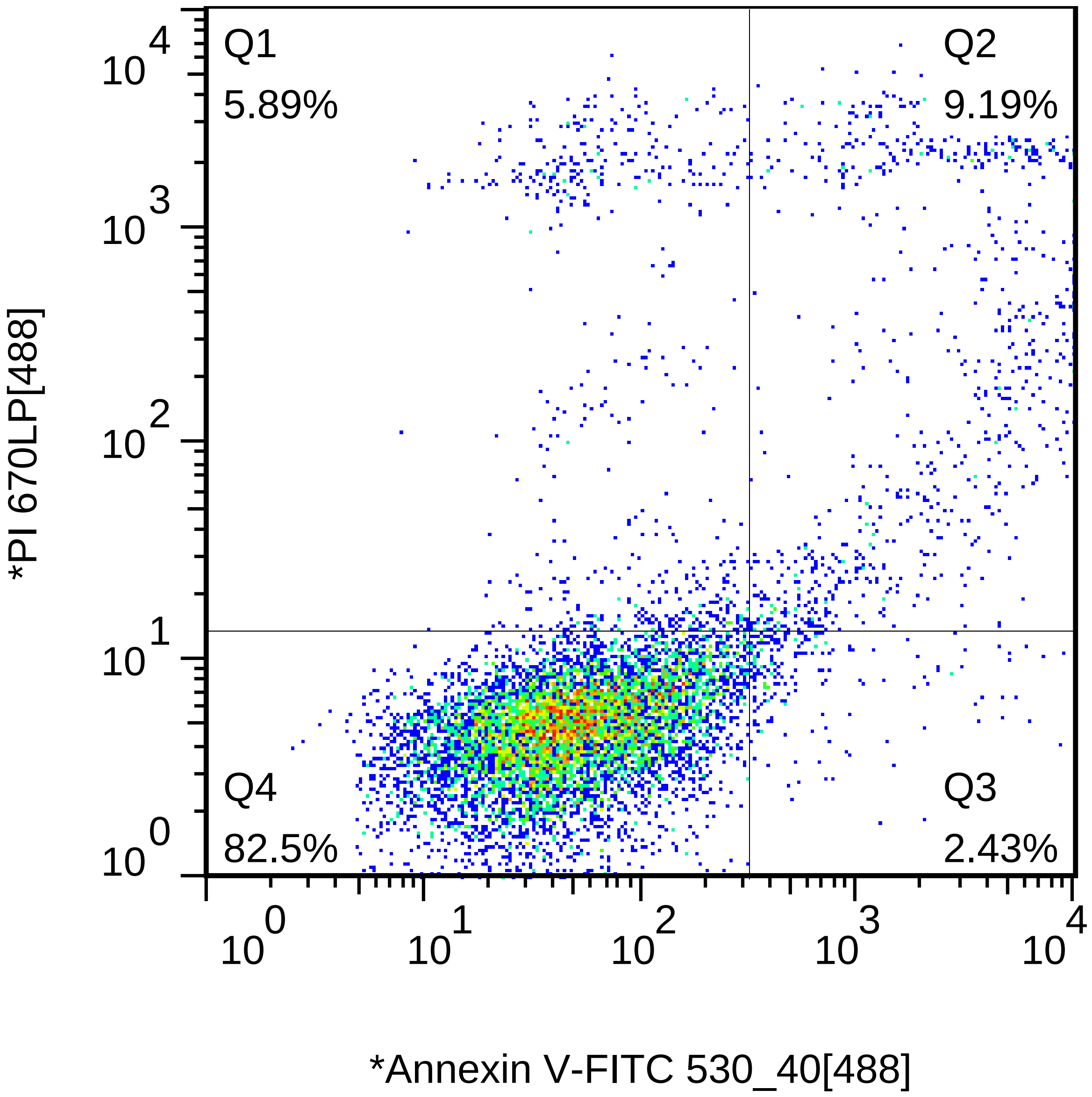

RKO-2206_002.fcs
FSC, SSC subset
9402

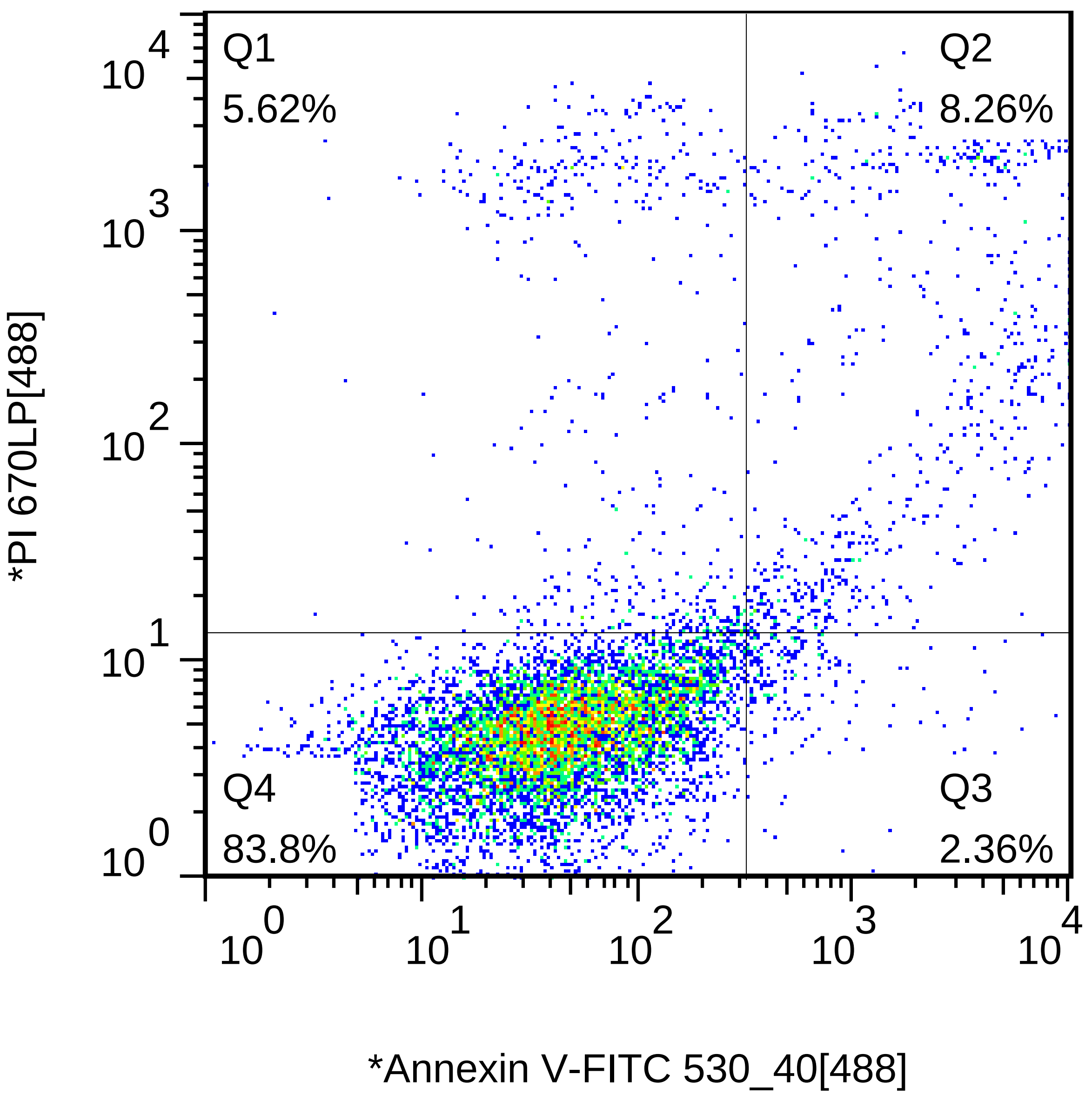

RKO-2206.fcs
FSC, SSC subset
9234
