# Supplementary material for: Therapeutic Suppression of FAK-AKT Signaling Overcomes Resistance to SHP2 Inhibition in Colorectal Carcinoma
Source: Front Pharmacol. 2021 Nov 1;12:739501. doi: 10.3389/fphar.2021.739501 (PMC8591248; doi:10.3389/fphar.2021.739501)
Supplement: Supplementary file 11 [file DataSheet6.ZIP › Figure3/Figure3D/SW620.pdf]

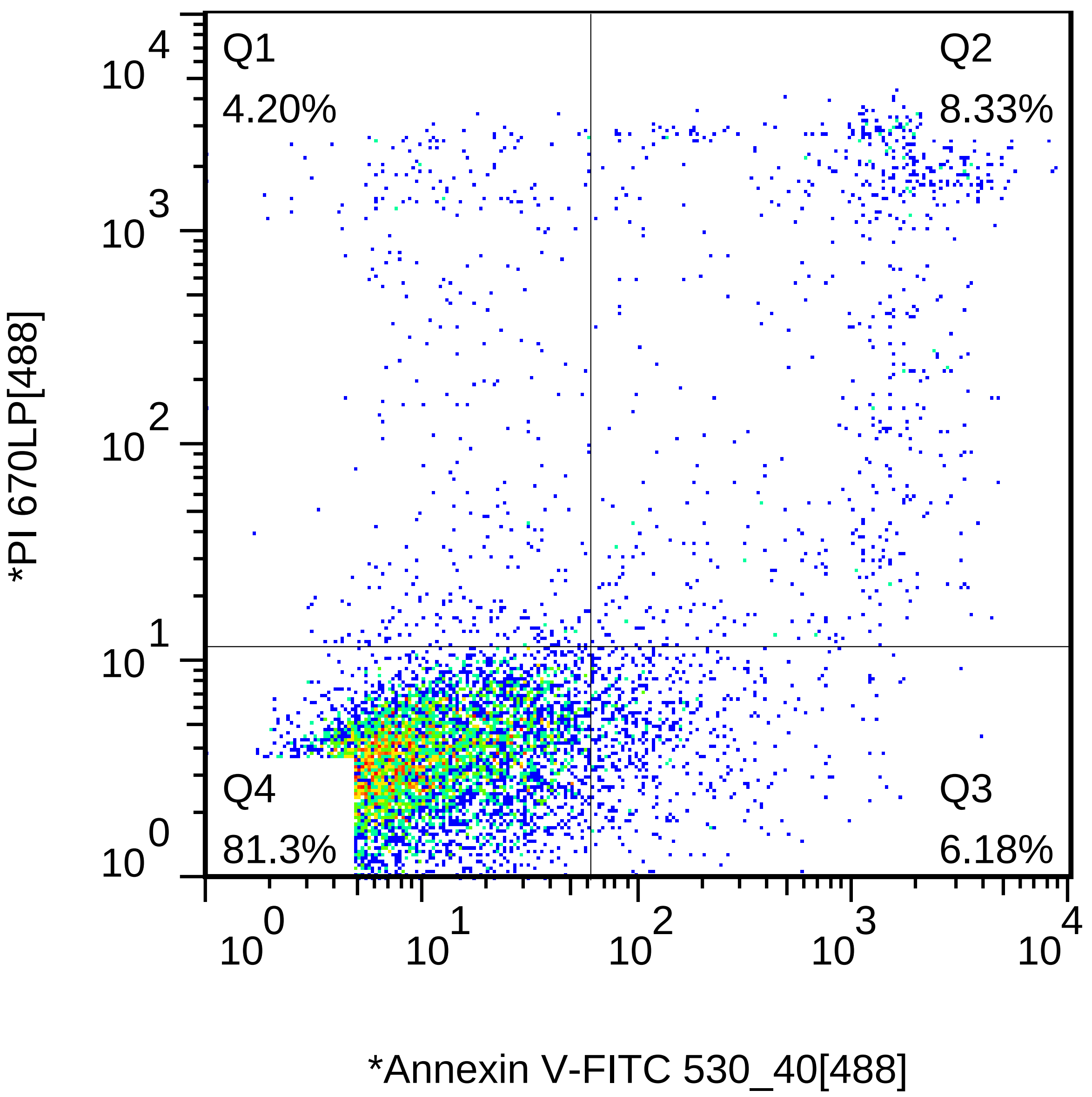

620DMSO_001.fcs
FSC, SSC subset
9175

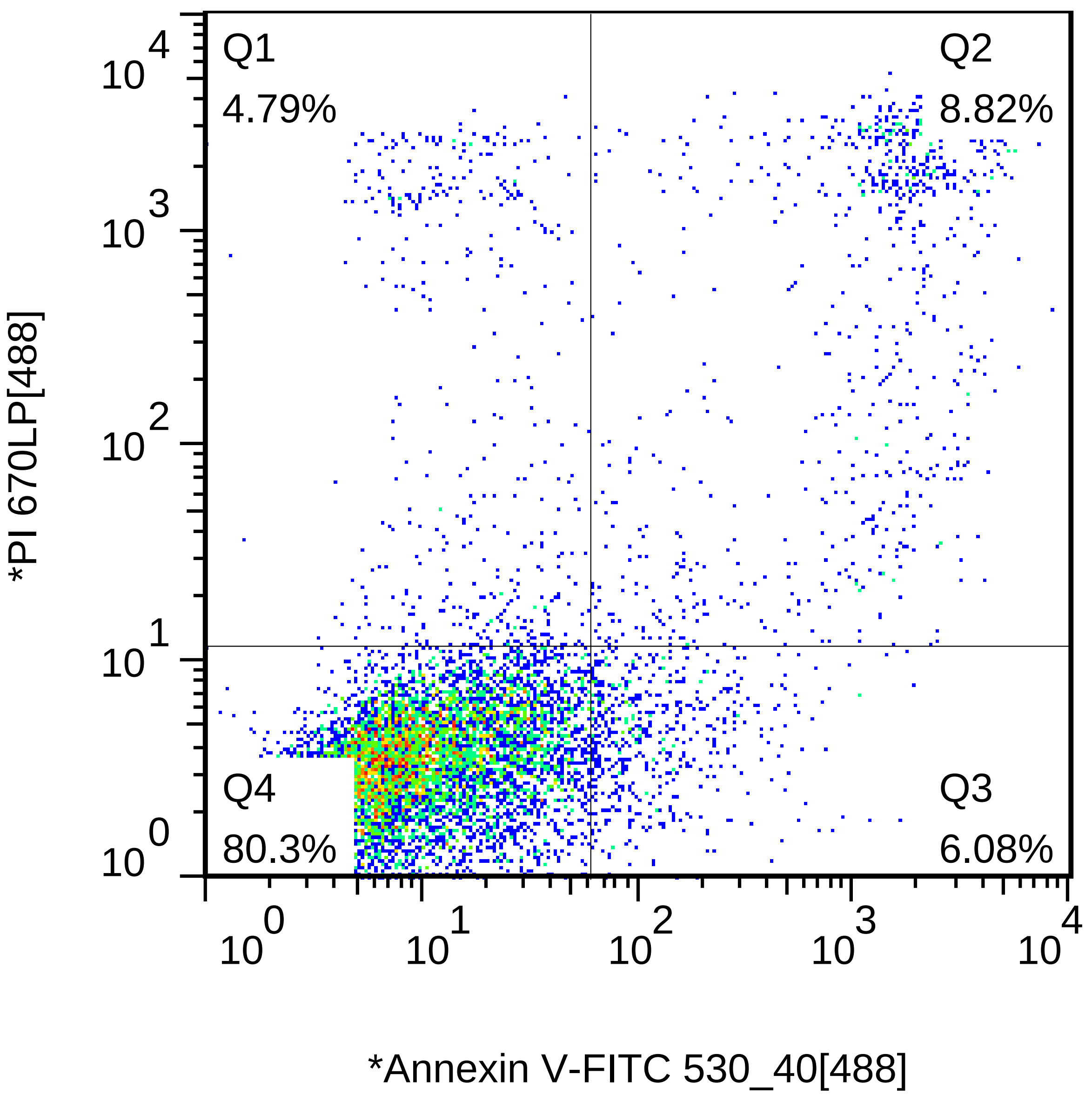

620DMSO_002.fcs
FSC, SSC subset
9253

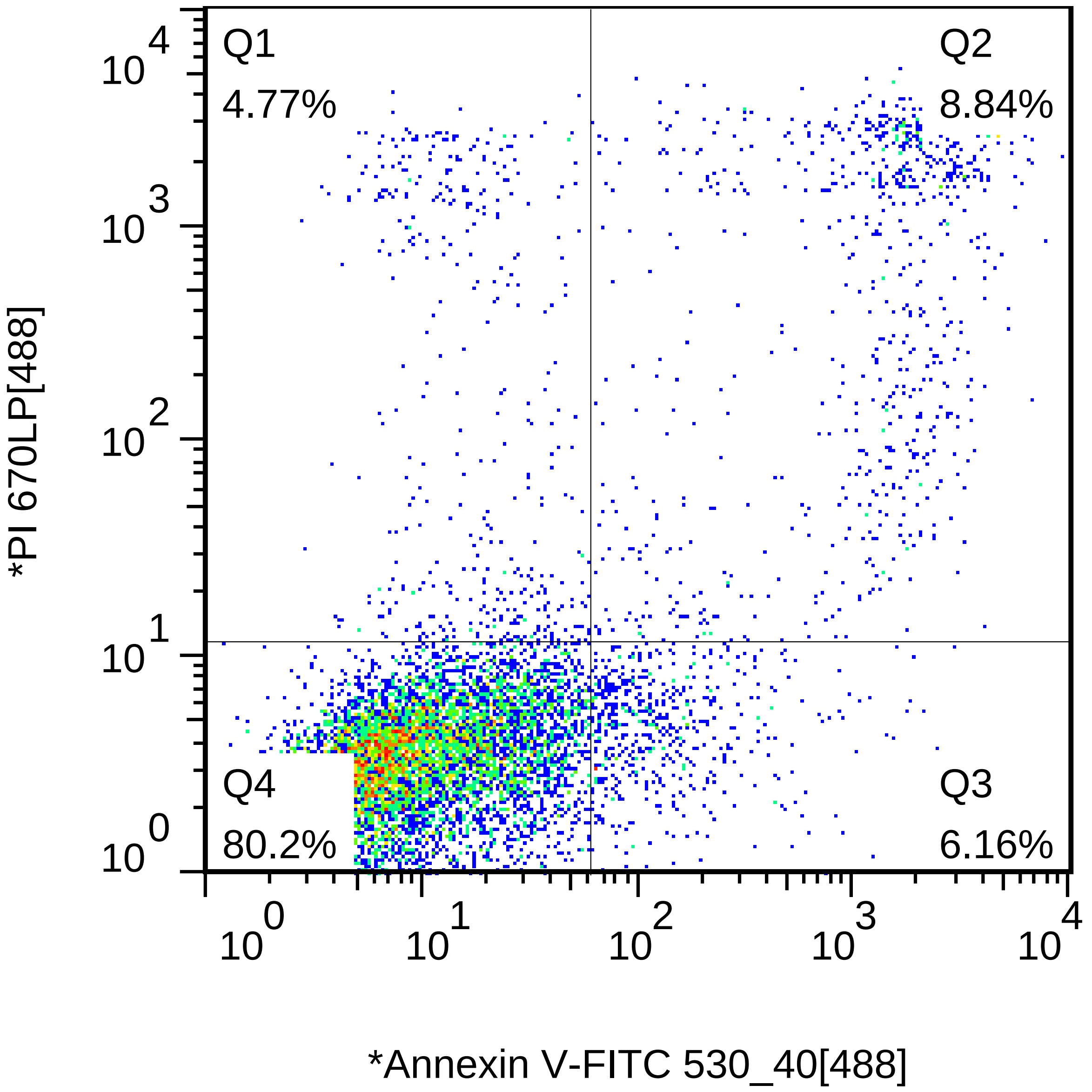

620DMSO_003.fcs
FSC, SSC subset
9170

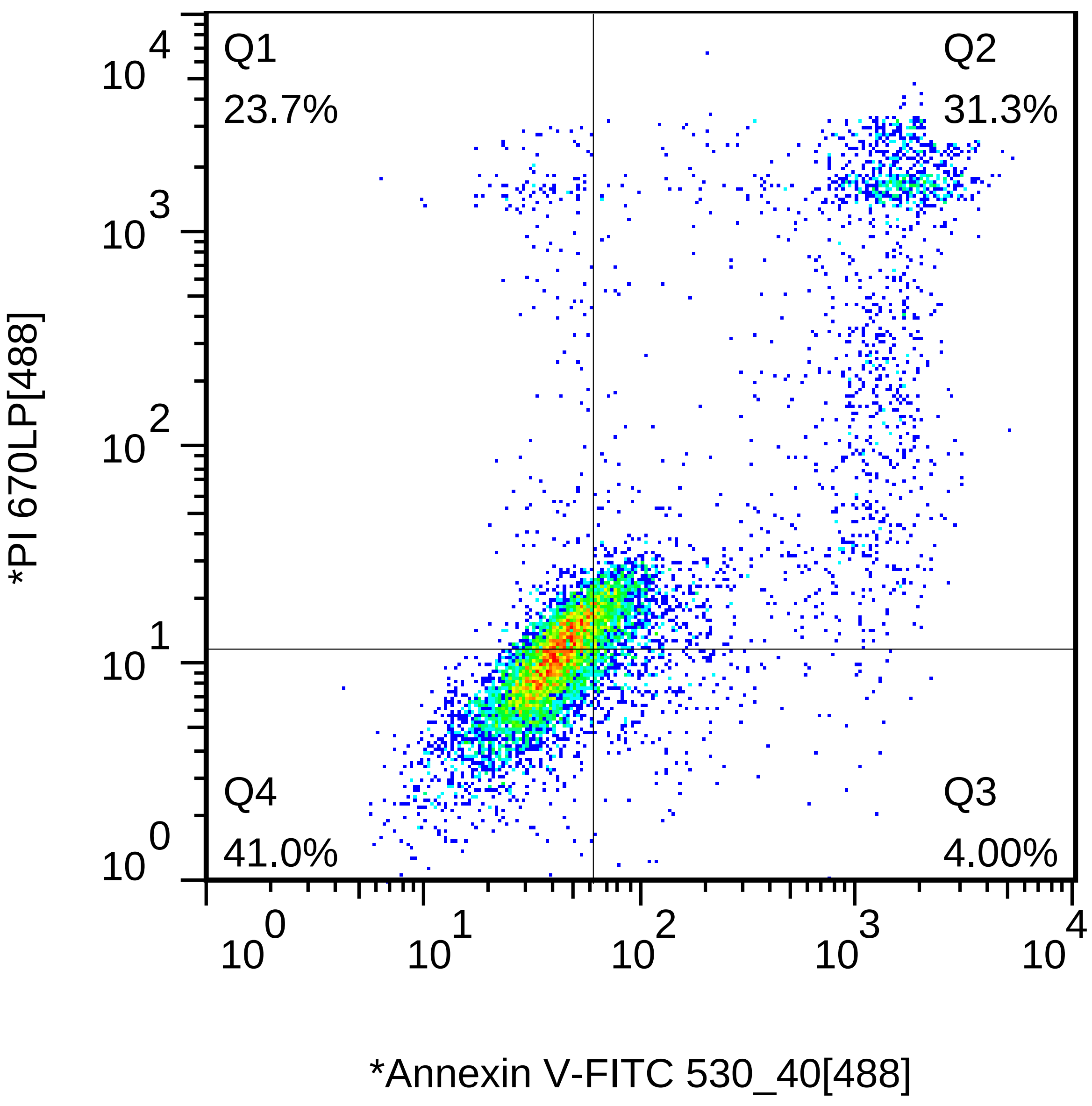

6209+6_002.fcs
FSC, SSC subset
9299

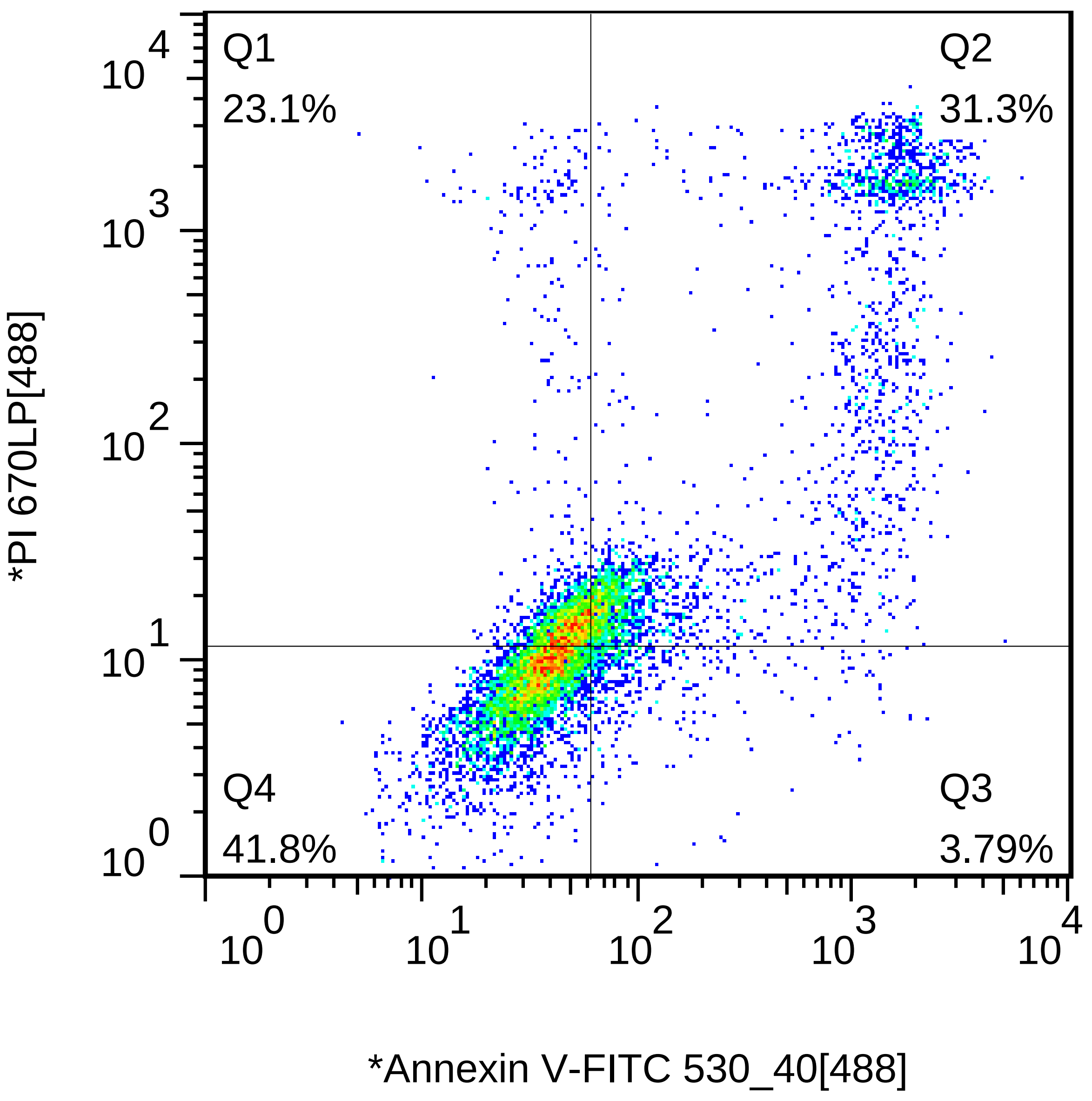

6209+6_003.fcs
FSC, SSC subset
9293

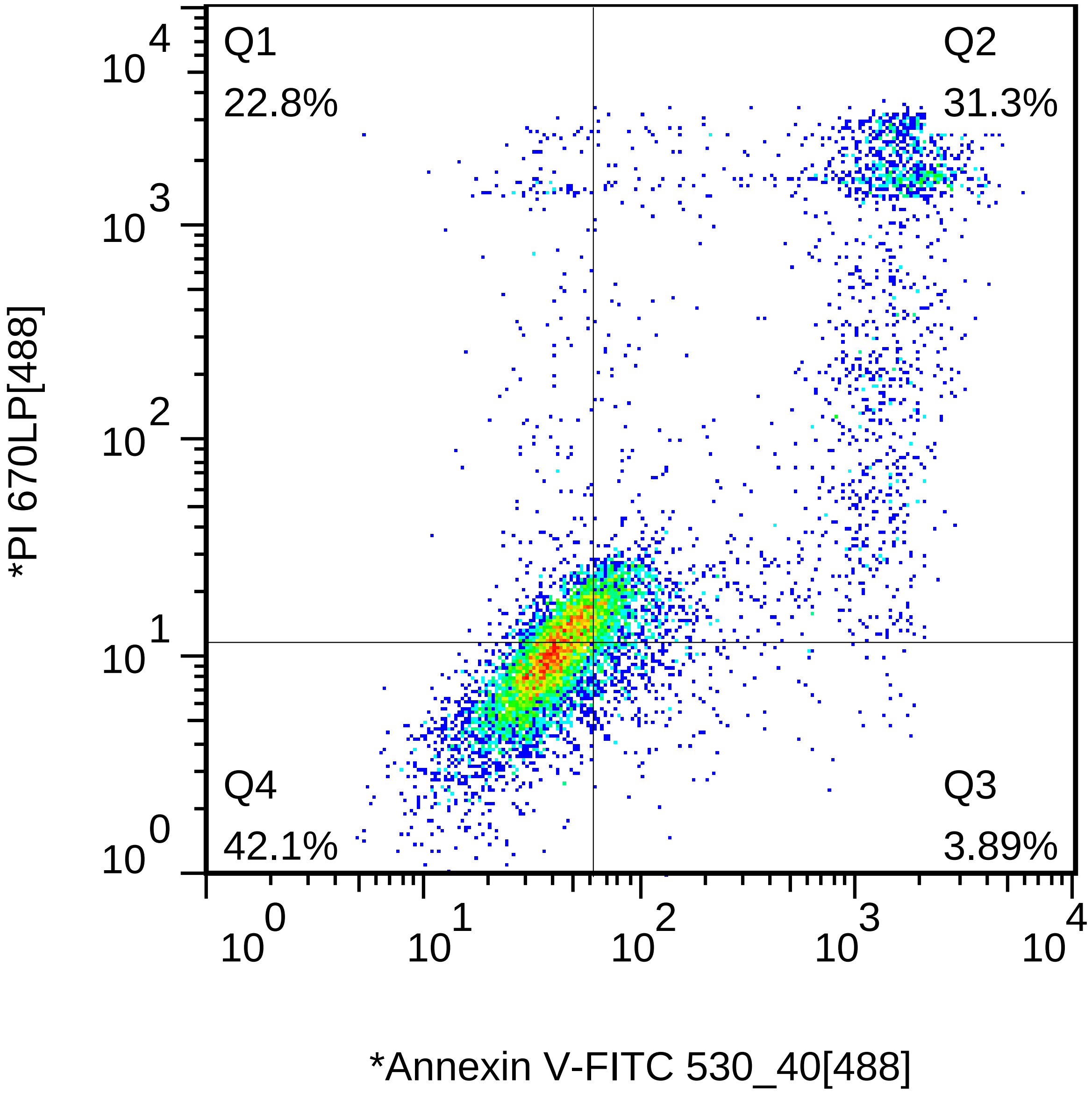

6209+6.fcs
FSC, SSC subset
9307

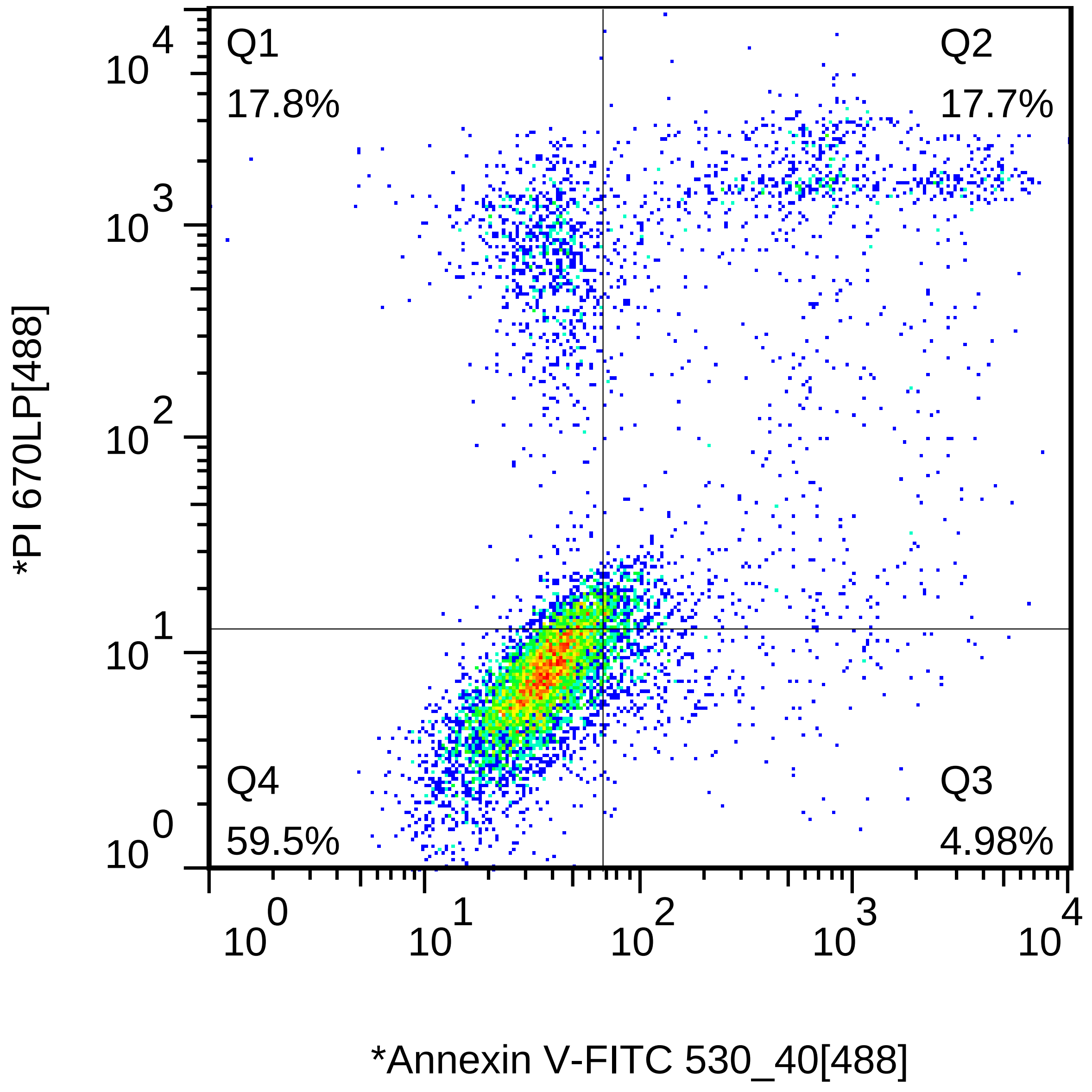

SW620 099_001.fcs
FSC, SSC subset
8911

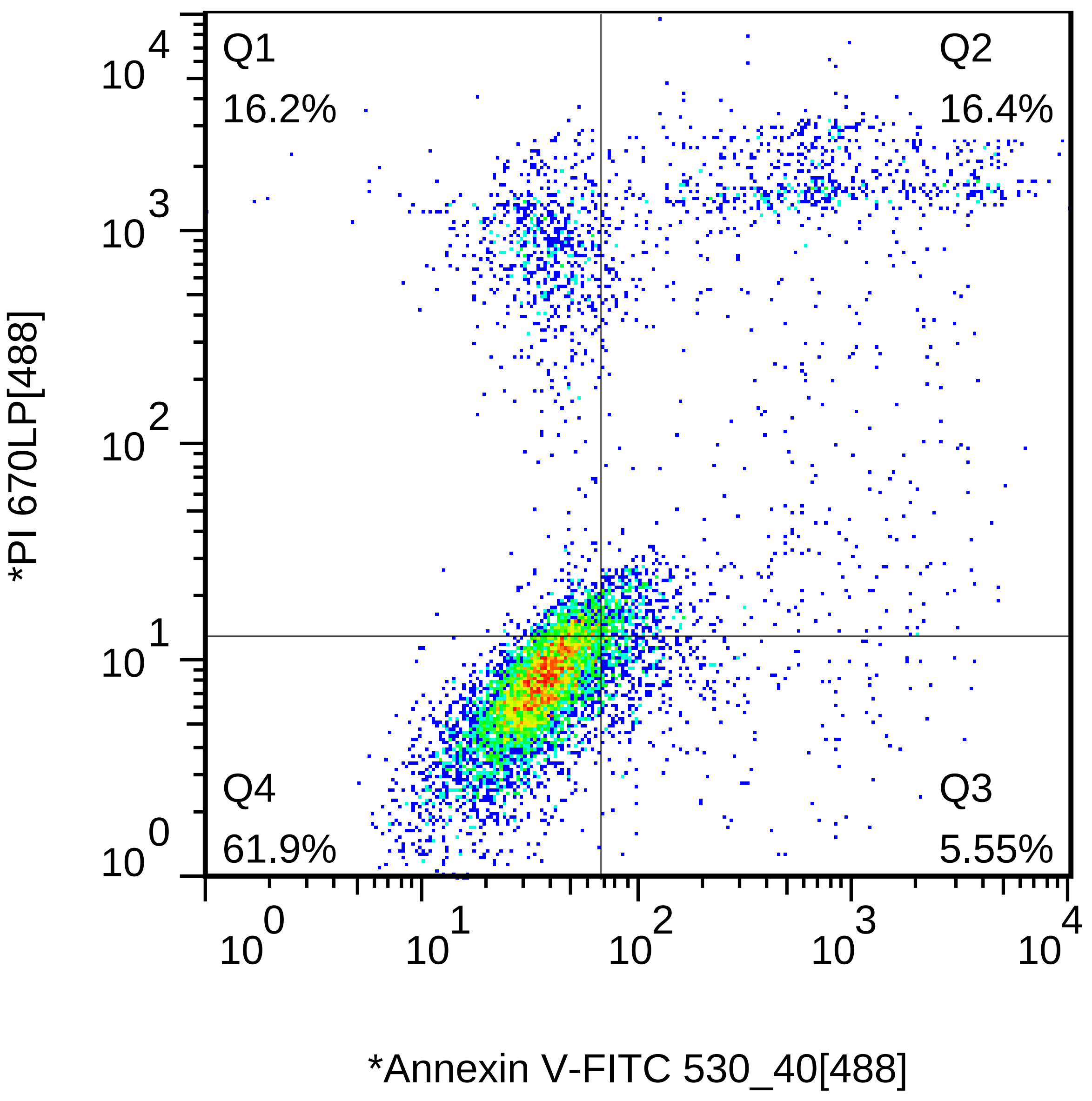

SW620 099_002.fcs
FSC, SSC subset
9029

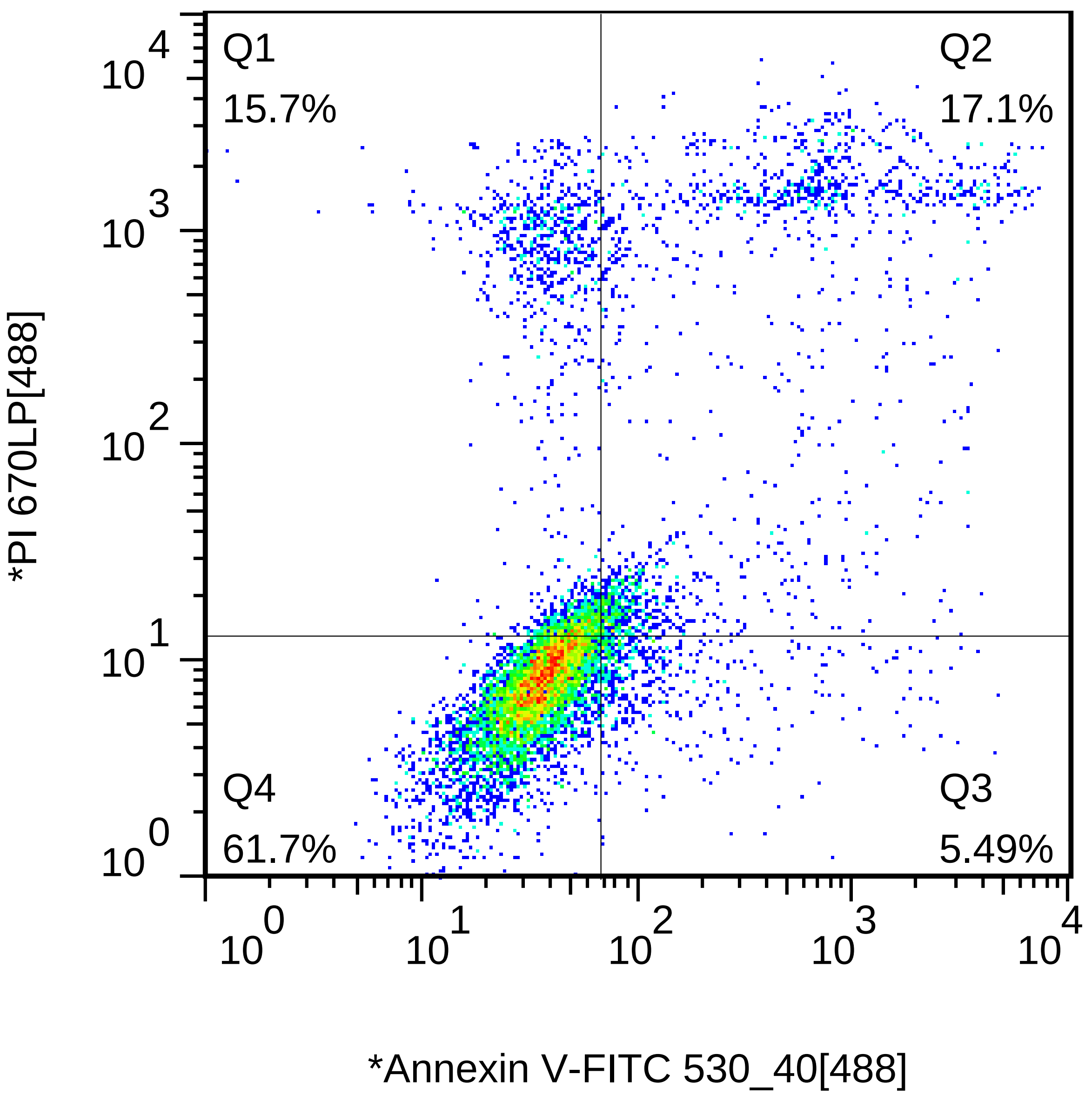

SW620 099_003.fcs
FSC, SSC subset
8994

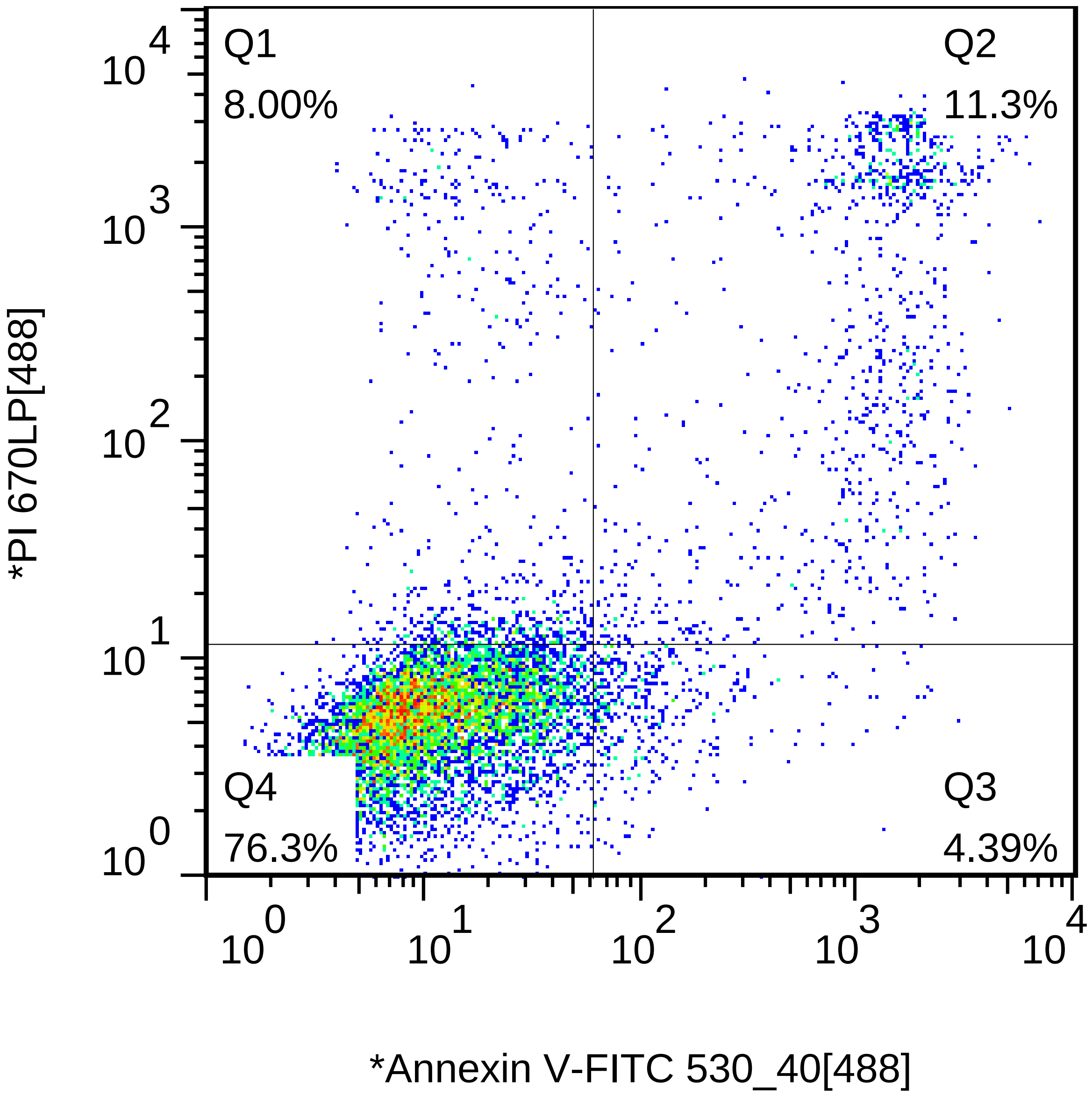

6202206_002.fcs
FSC, SSC subset
9441

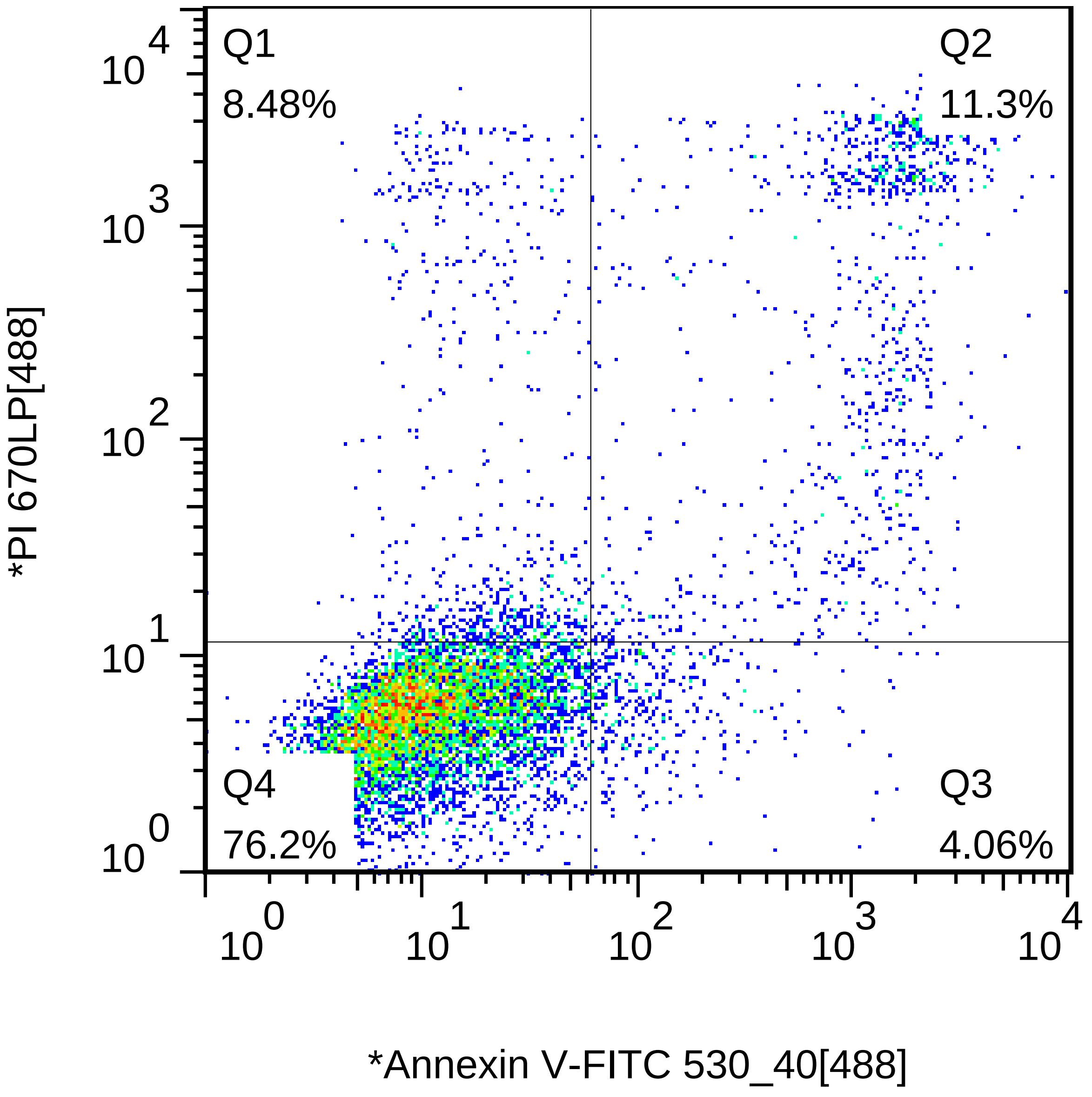

6202206_003.fcs
FSC, SSC subset
9473

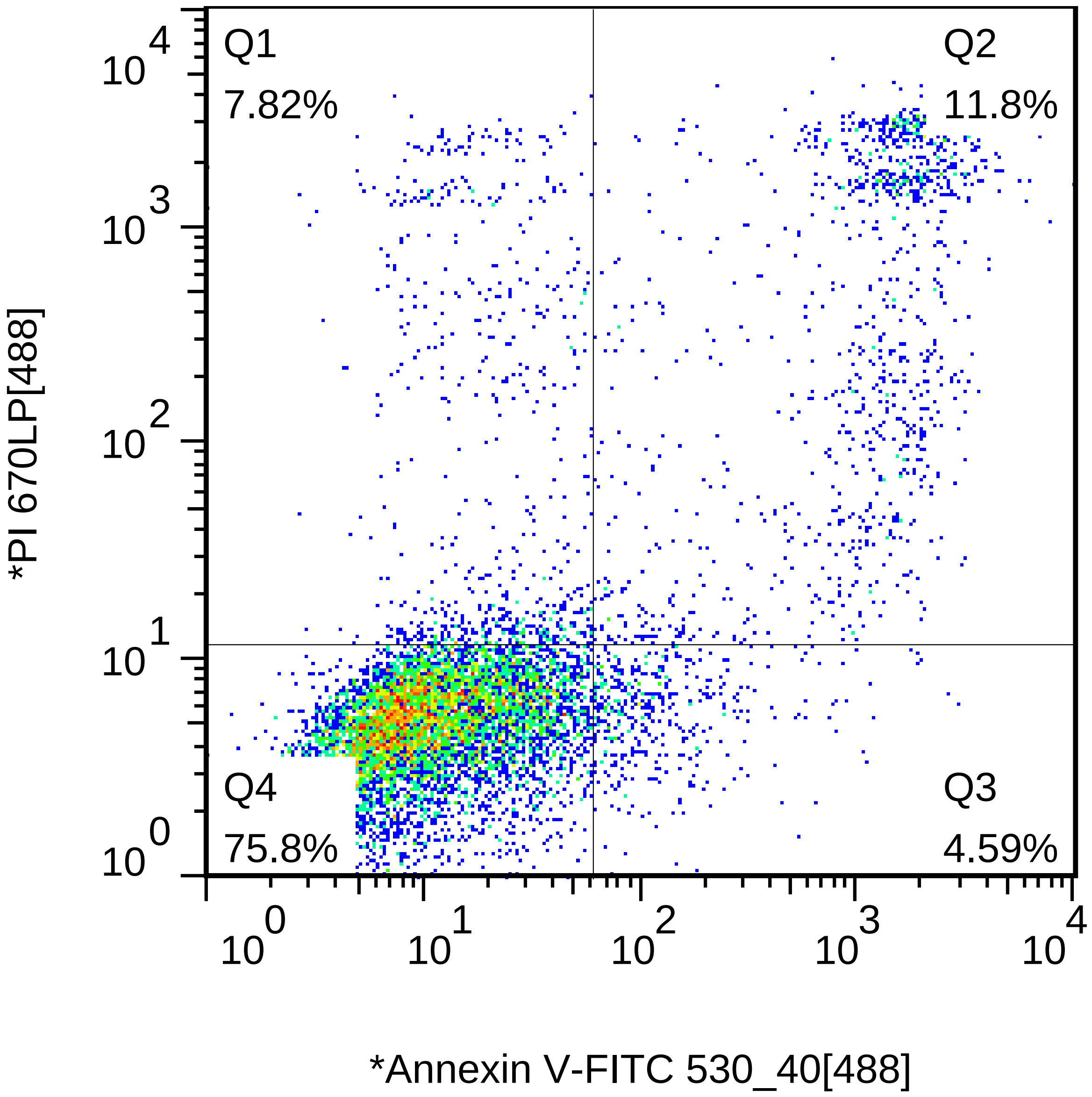

6202206.fcs
FSC, SSC subset
9399
