# Supplementary material for: Therapeutic Suppression of FAK-AKT Signaling Overcomes Resistance to SHP2 Inhibition in Colorectal Carcinoma
Source: Front Pharmacol. 2021 Nov 1;12:739501. doi: 10.3389/fphar.2021.739501 (PMC8591248; doi:10.3389/fphar.2021.739501)
Supplement: Supplementary file 12 [file DataSheet5.ZIP › Figure2/Figure2D/COLO205/instruction.pptx]

## Slide 1
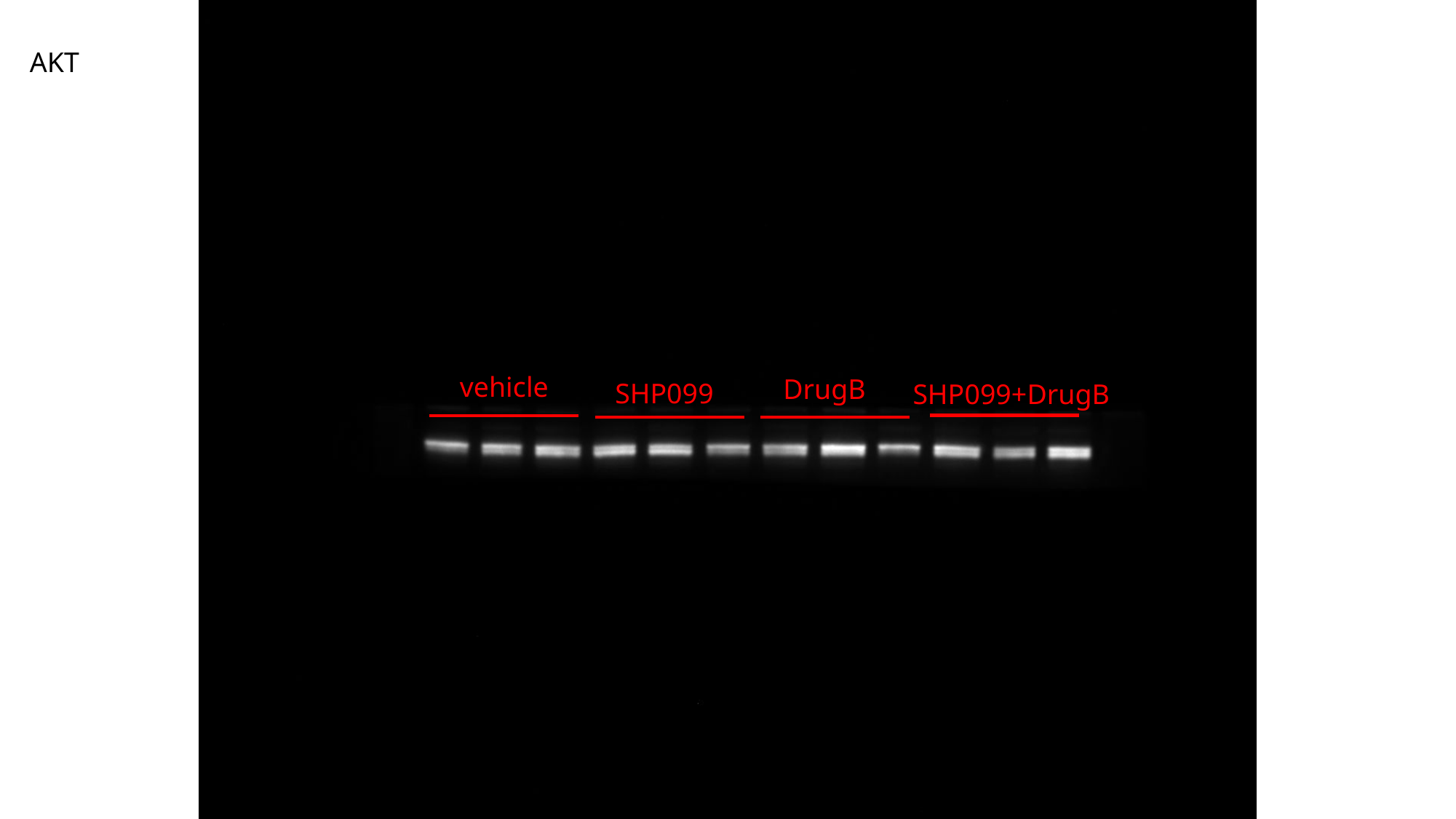

AKT
vehicle
DrugB
SHP099
SHP099+DrugB

## Slide 2
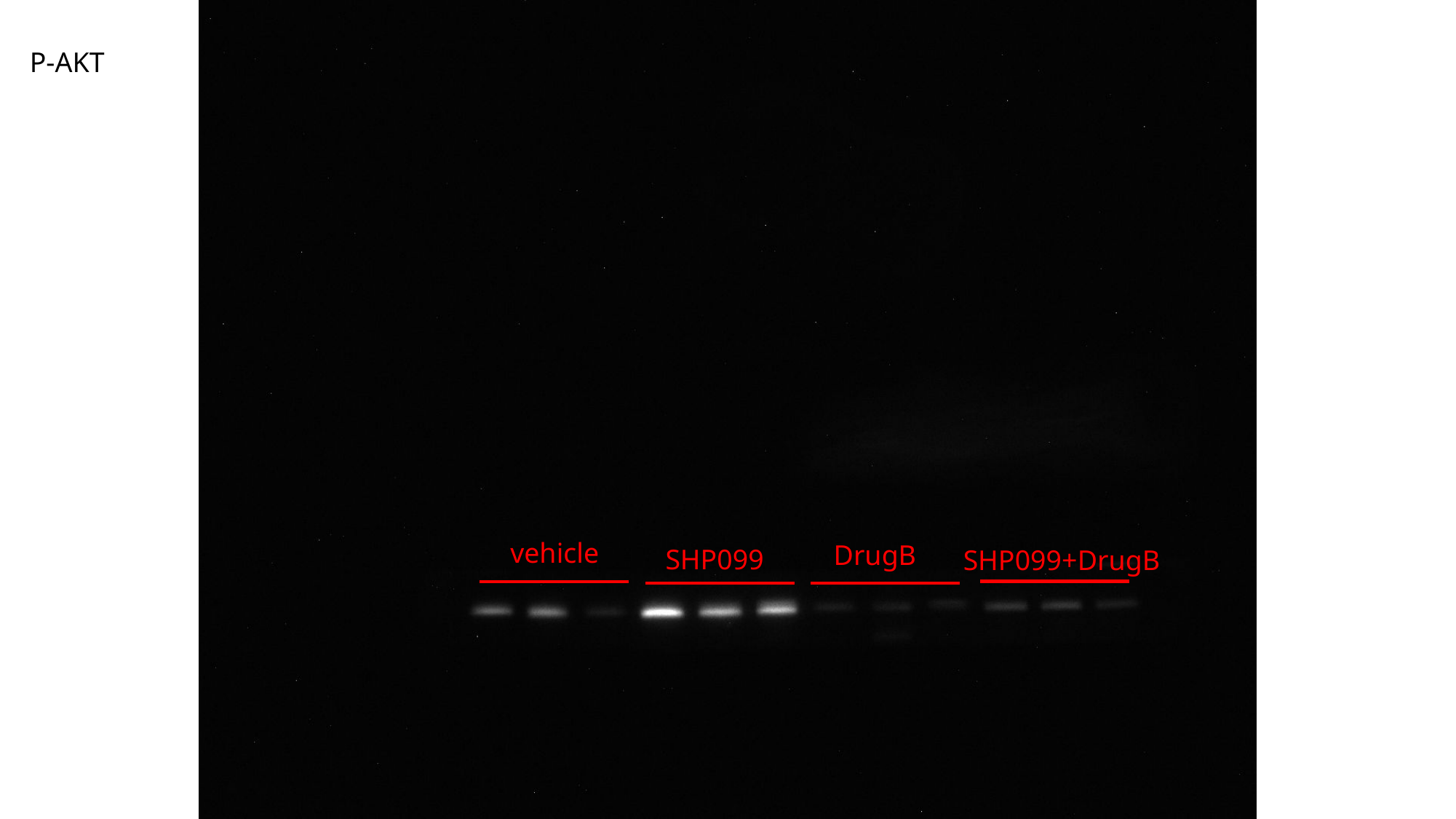

P-AKT
vehicle
DrugB
SHP099
SHP099+DrugB

## Slide 3
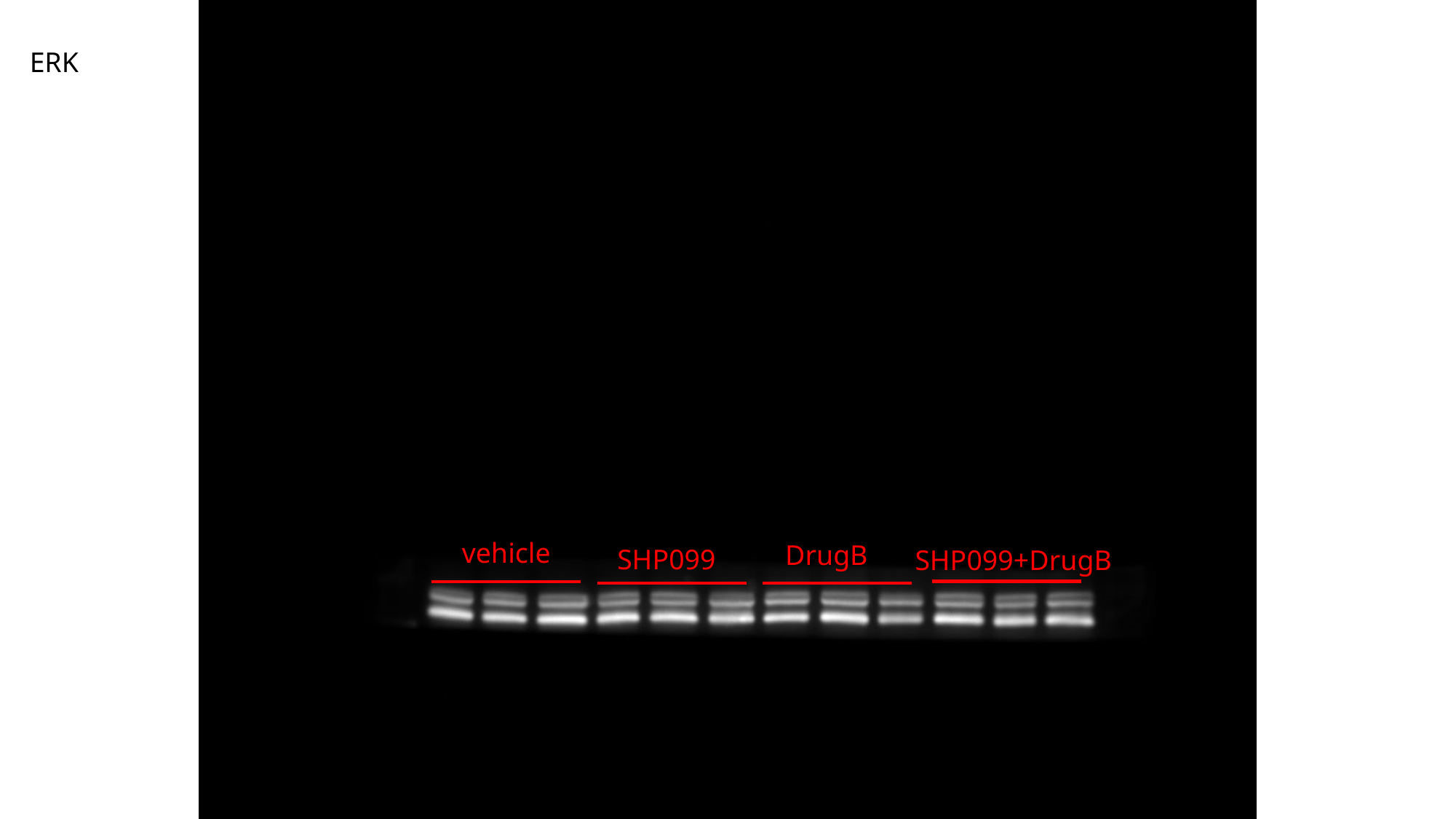

ERK
vehicle
DrugB
SHP099
SHP099+DrugB

## Slide 4
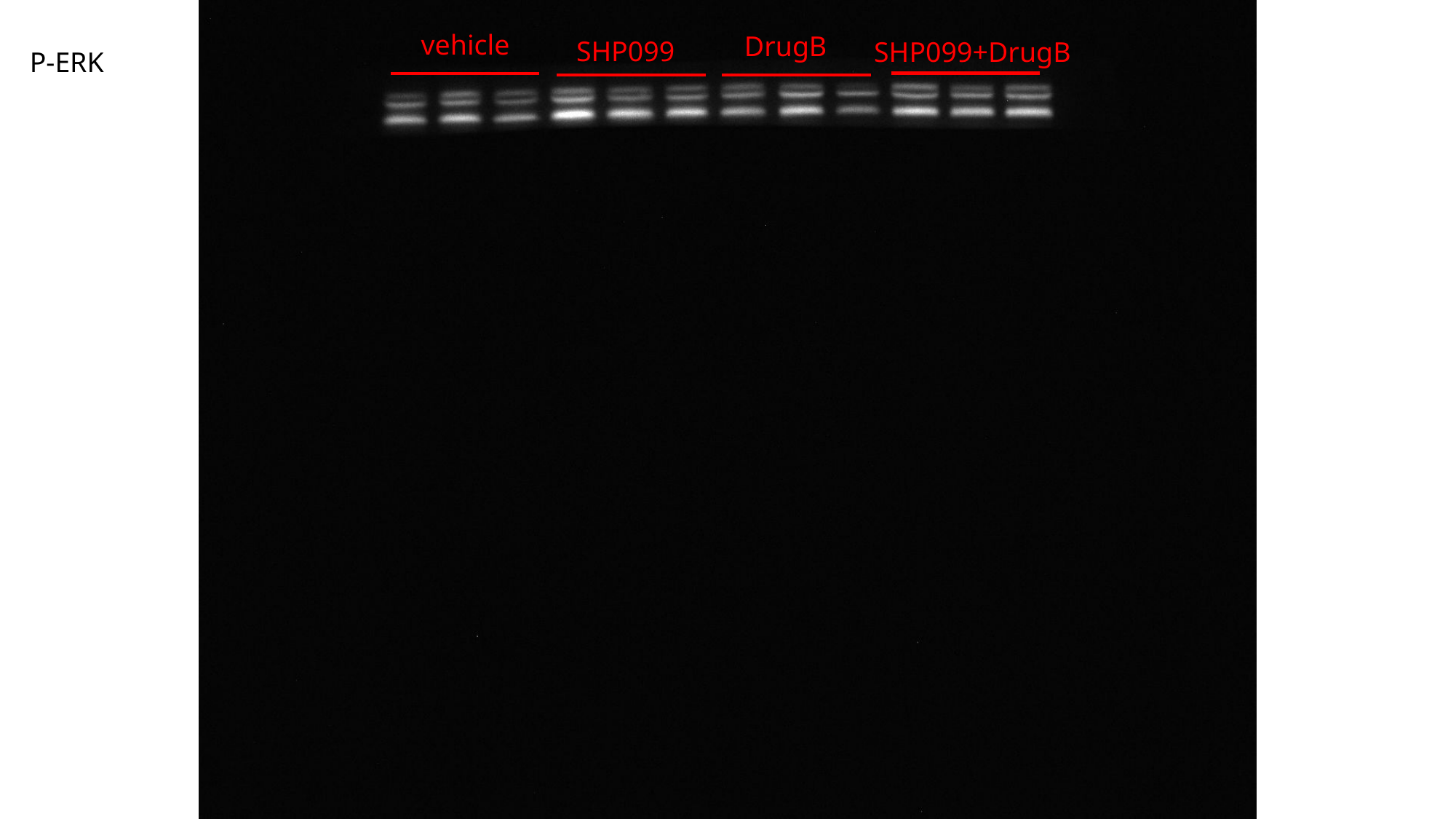

vehicle
DrugB
SHP099
SHP099+DrugB
P-ERK

## Slide 5
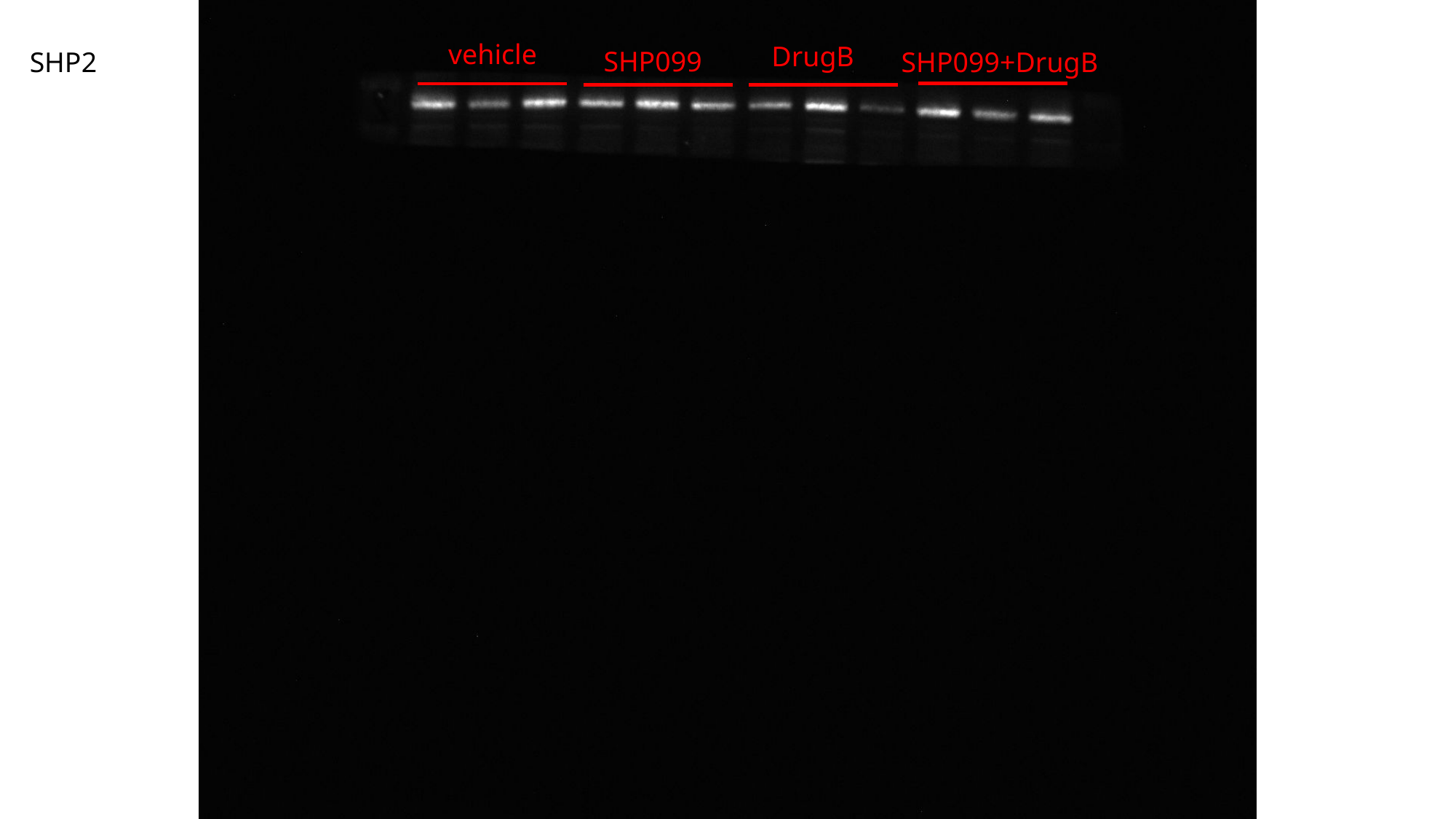

vehicle
DrugB
SHP099
SHP2
SHP099+DrugB

## Slide 6
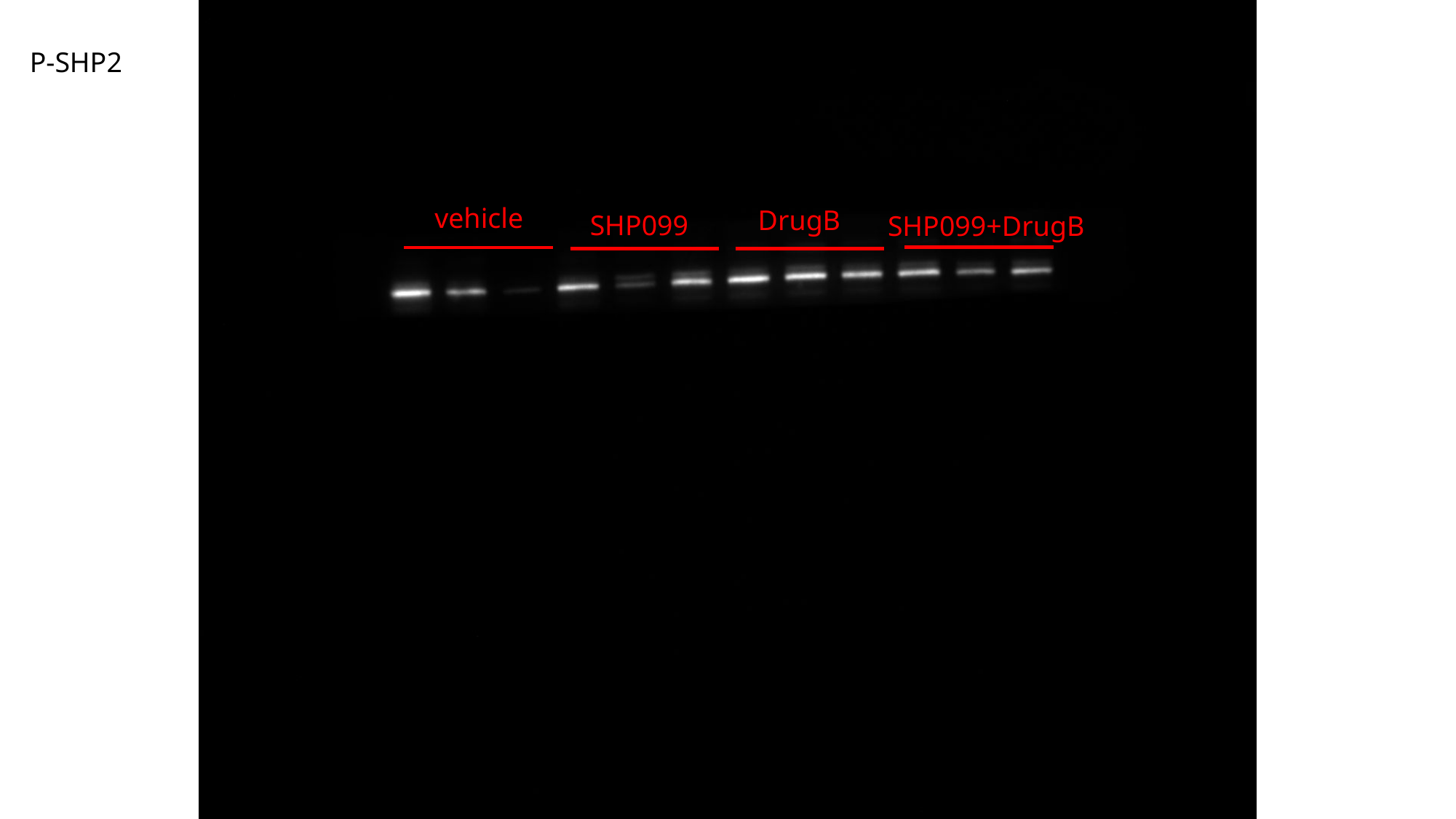

P-SHP2
vehicle
DrugB
SHP099
SHP099+DrugB

## Slide 7
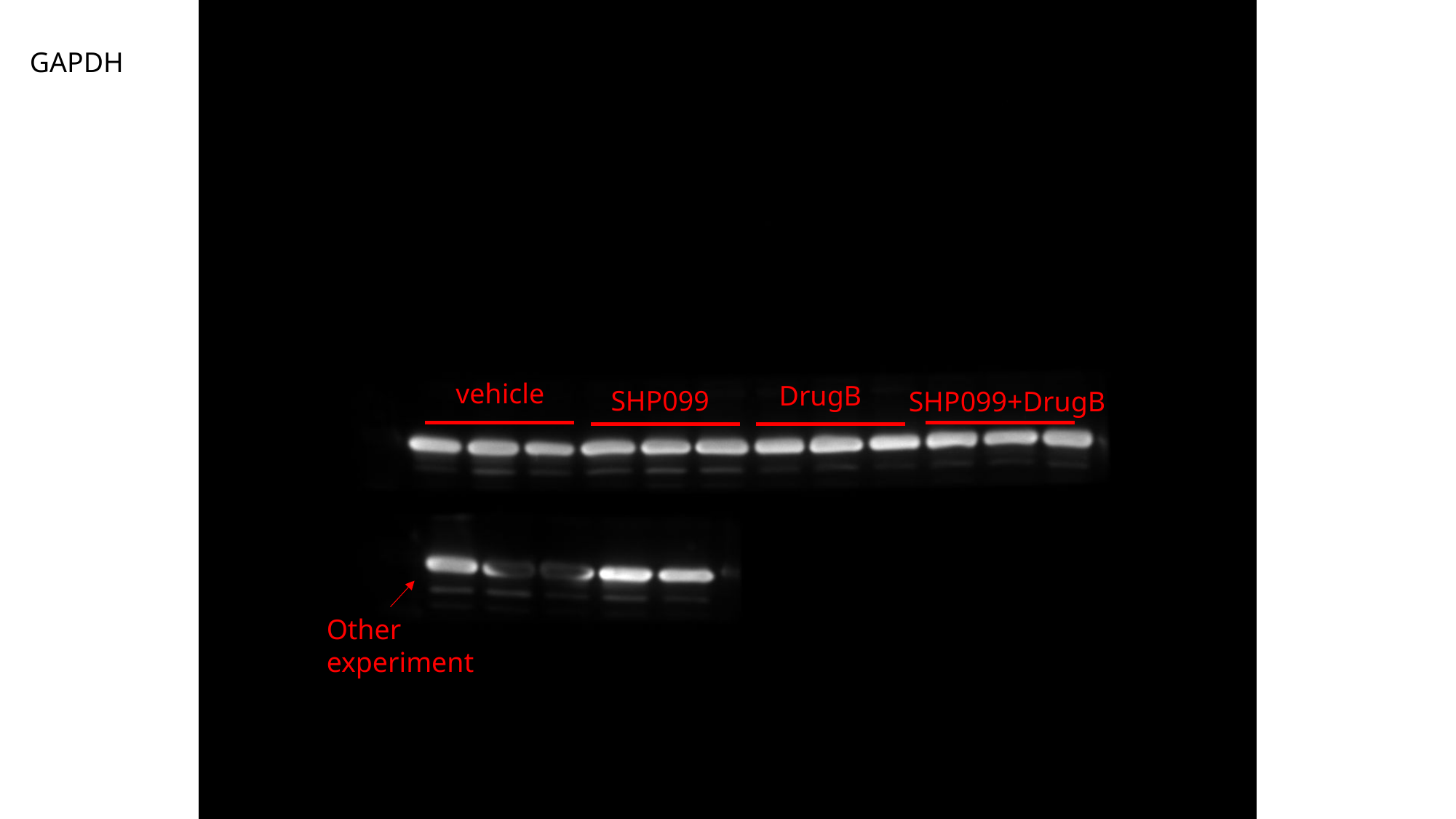

GAPDH
vehicle
DrugB
SHP099
SHP099+DrugB
Other experiment
