# Supplementary figures and images for: Therapeutic Suppression of FAK-AKT Signaling Overcomes Resistance to SHP2 Inhibition in Colorectal Carcinoma (part 2 of 2)
Source: Front Pharmacol. 2021 Nov 1;12:739501. doi: 10.3389/fphar.2021.739501 (PMC8591248; doi:10.3389/fphar.2021.739501)

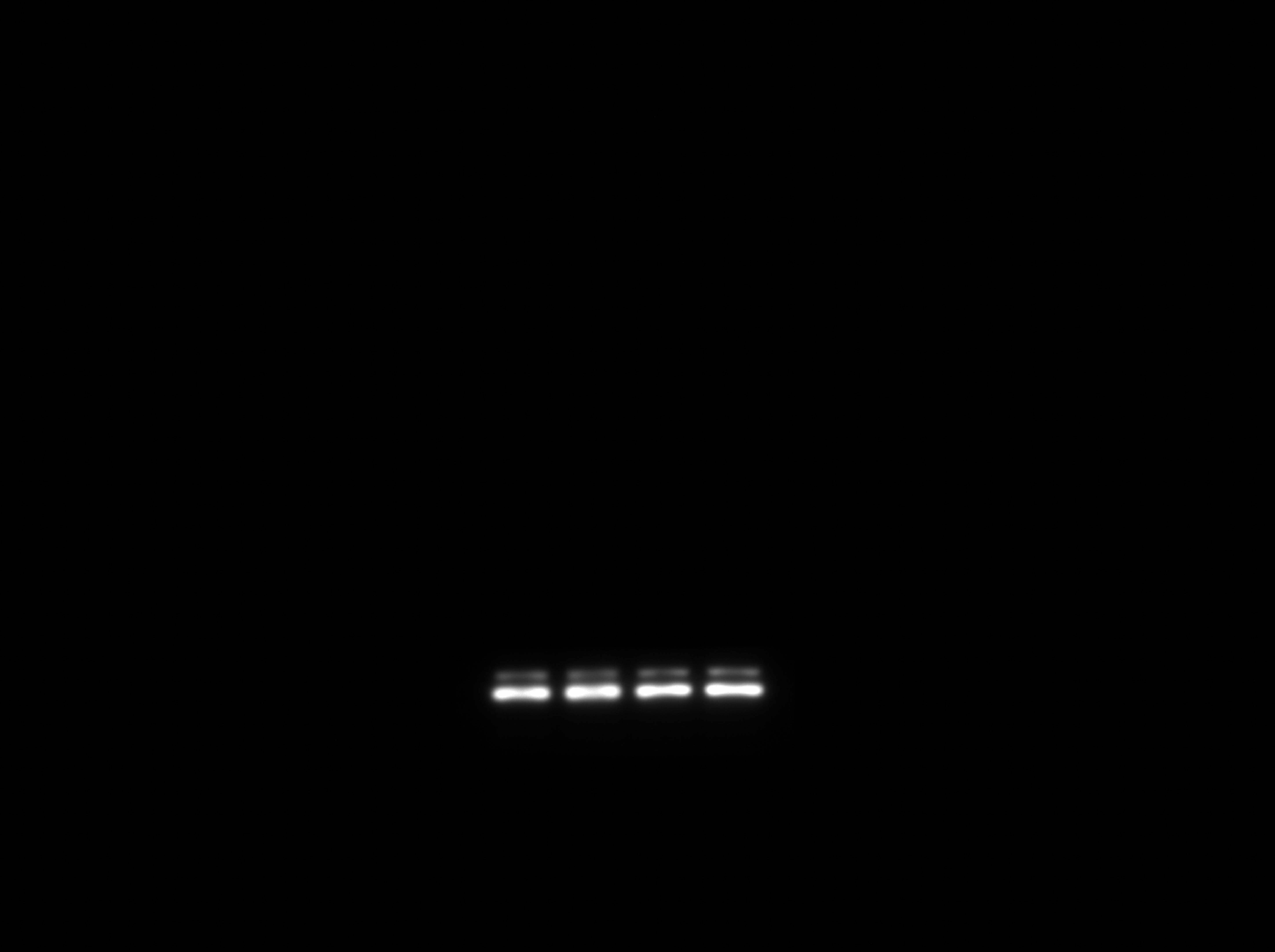

Supplement: Supplementary file 12 [file DataSheet5.ZIP › Figure2/Figure2A/ERK CW-2.jpg]

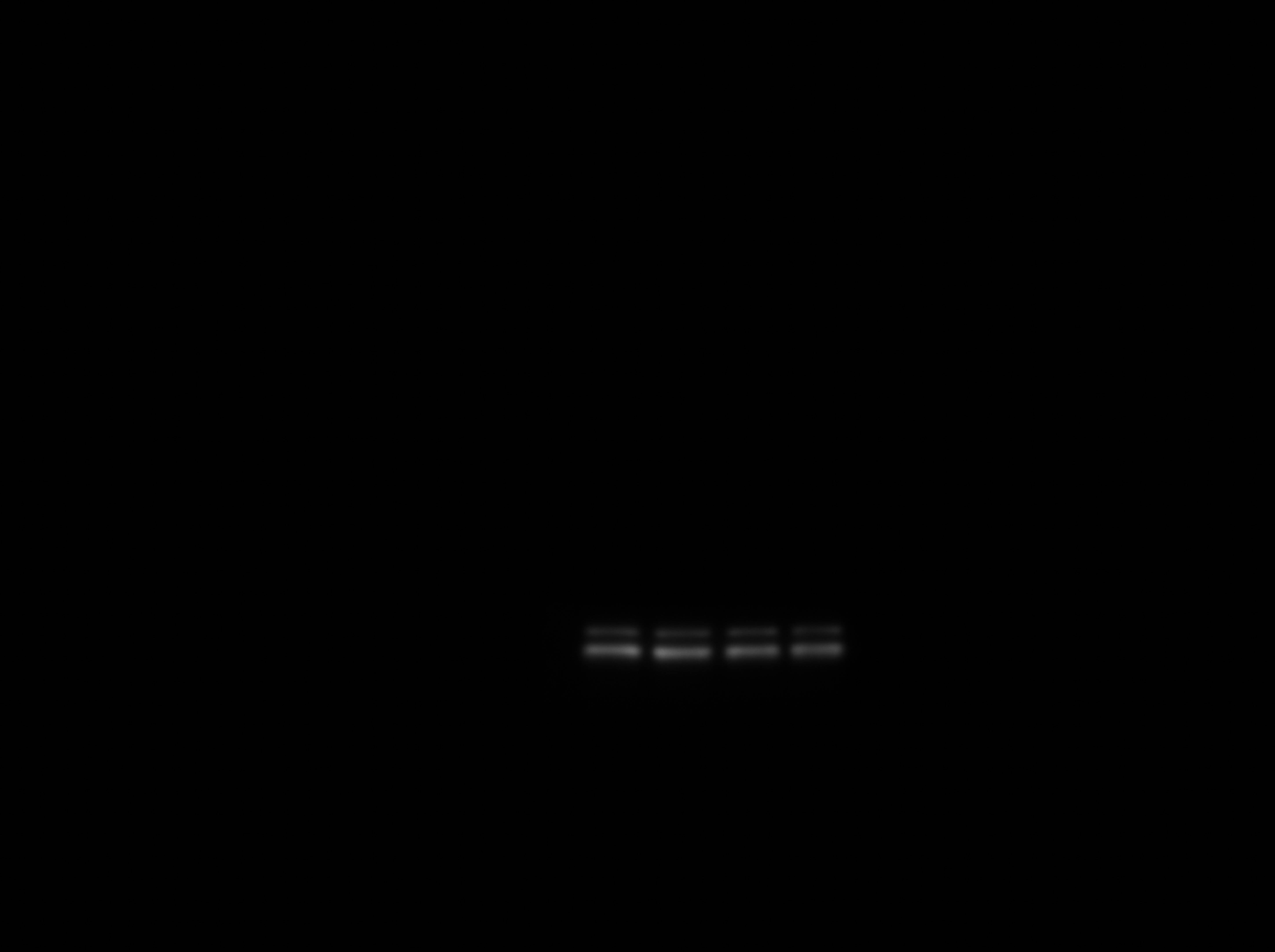

Supplement: Supplementary file 12 [file DataSheet5.ZIP › Figure2/Figure2A/ERK Caco-2.jpg]

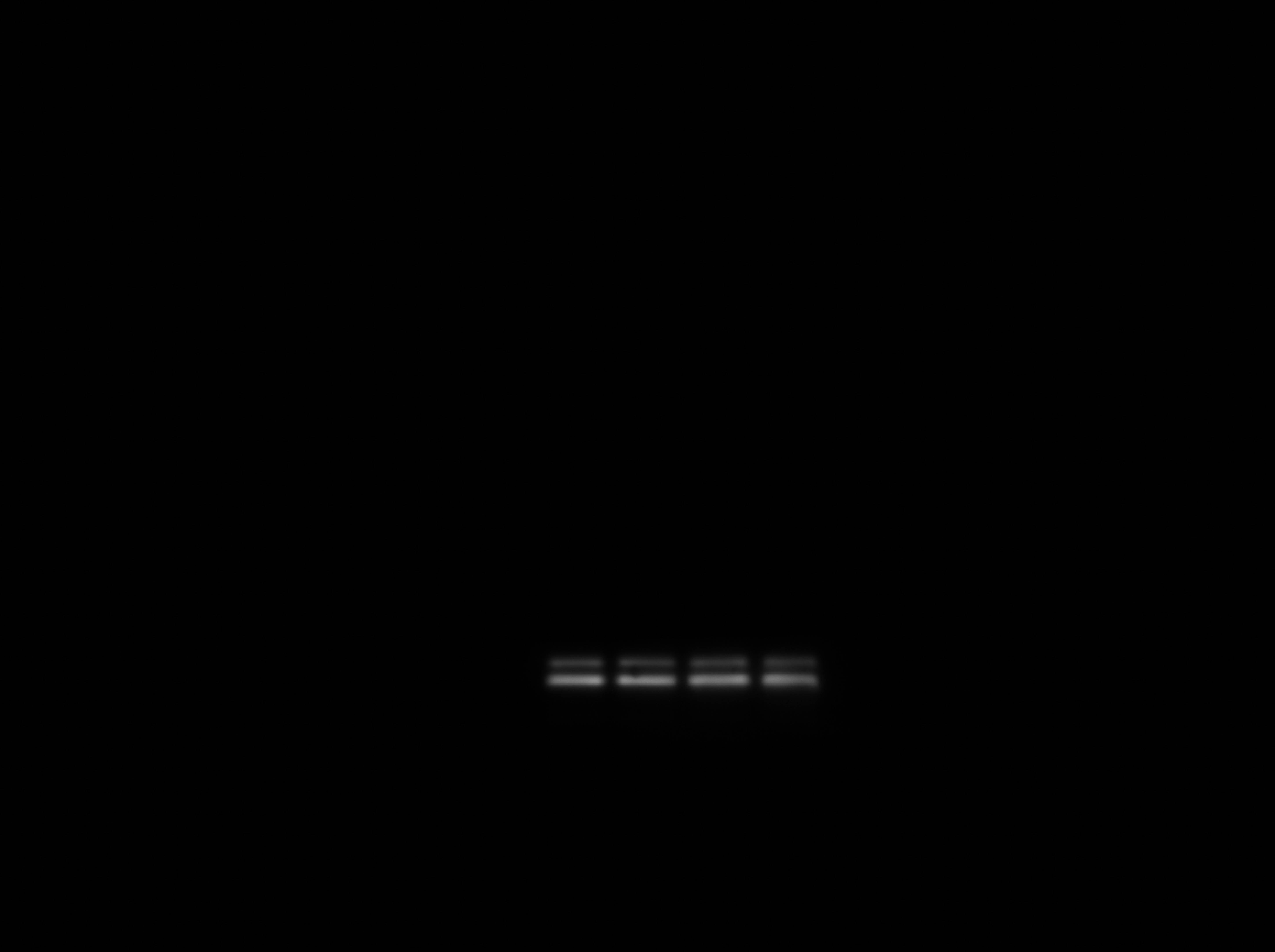

Supplement: Supplementary file 12 [file DataSheet5.ZIP › Figure2/Figure2A/ERK Colo205.jpg]

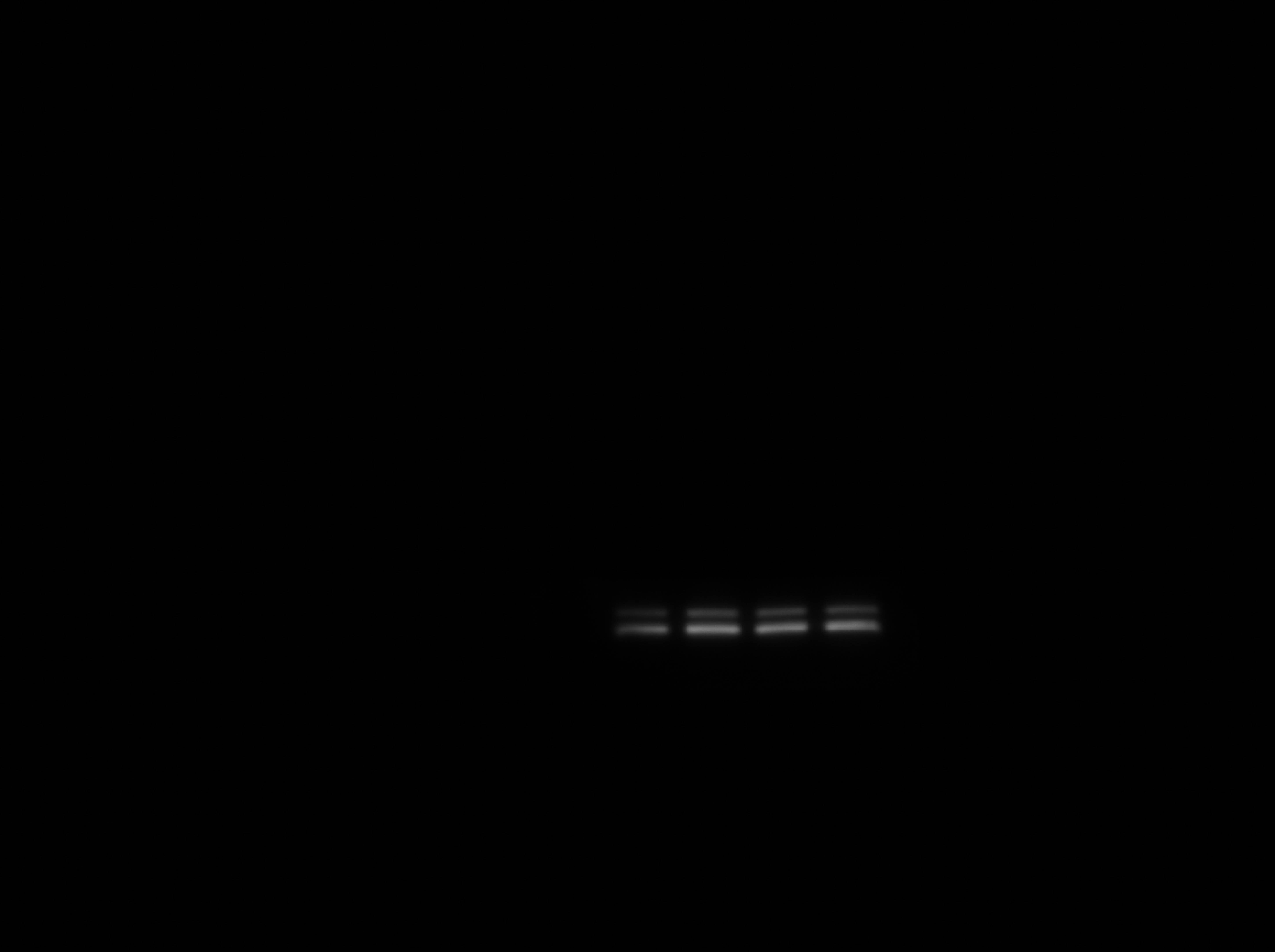

Supplement: Supplementary file 12 [file DataSheet5.ZIP › Figure2/Figure2A/ERK RKO.jpg]

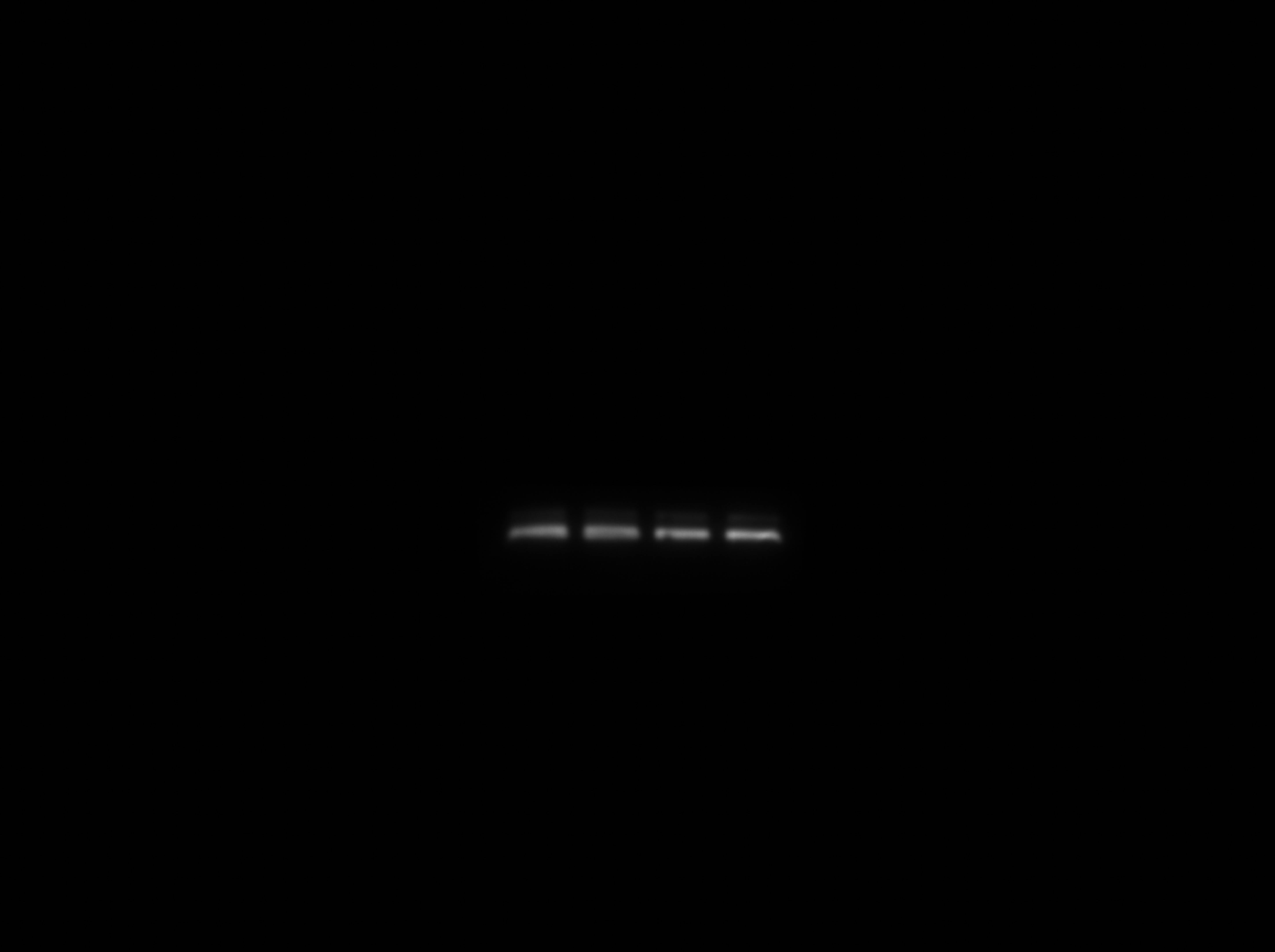

Supplement: Supplementary file 12 [file DataSheet5.ZIP › Figure2/Figure2A/ERK SW480.jpg]

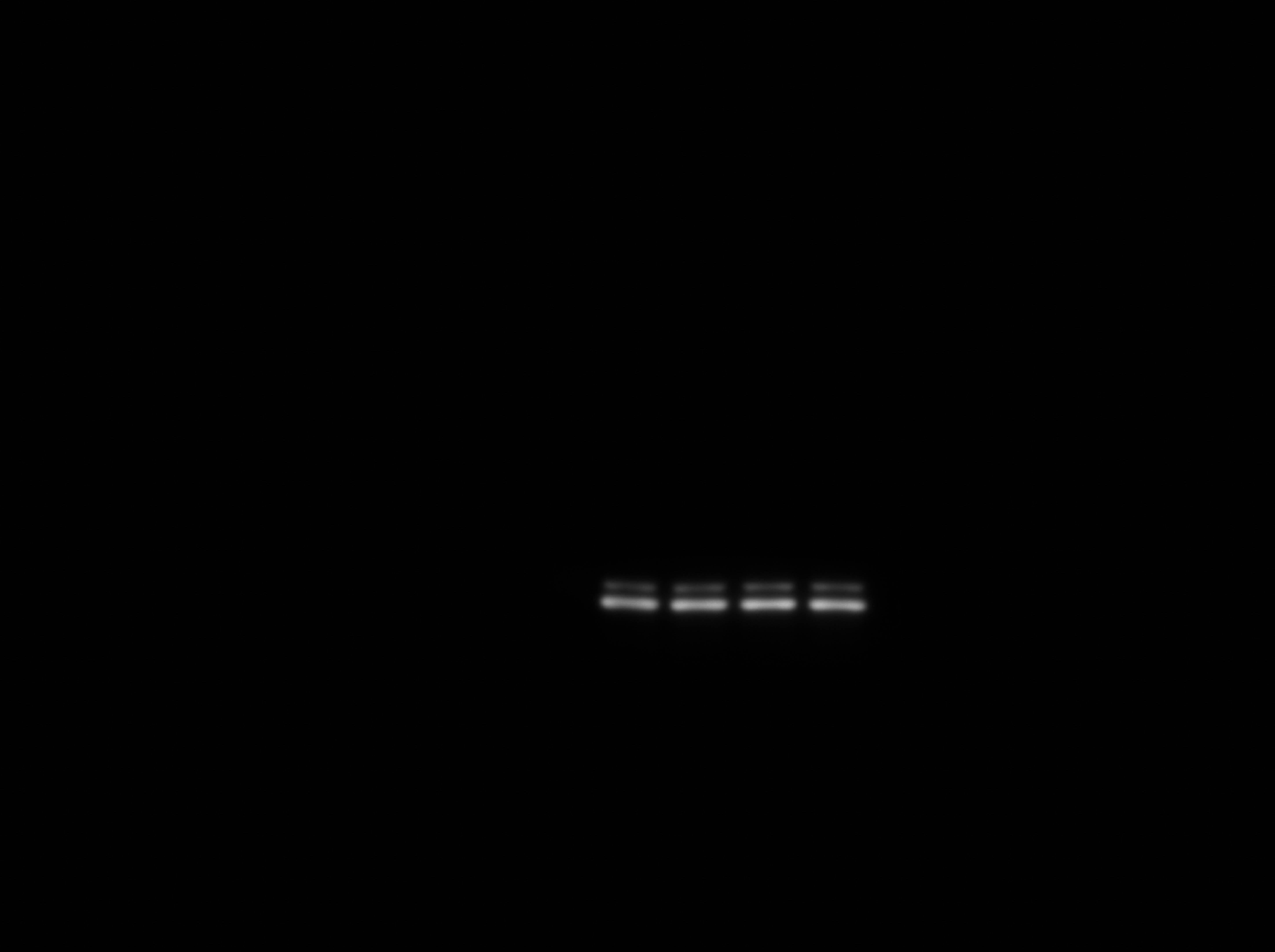

Supplement: Supplementary file 12 [file DataSheet5.ZIP › Figure2/Figure2A/ERK SW620.jpg]

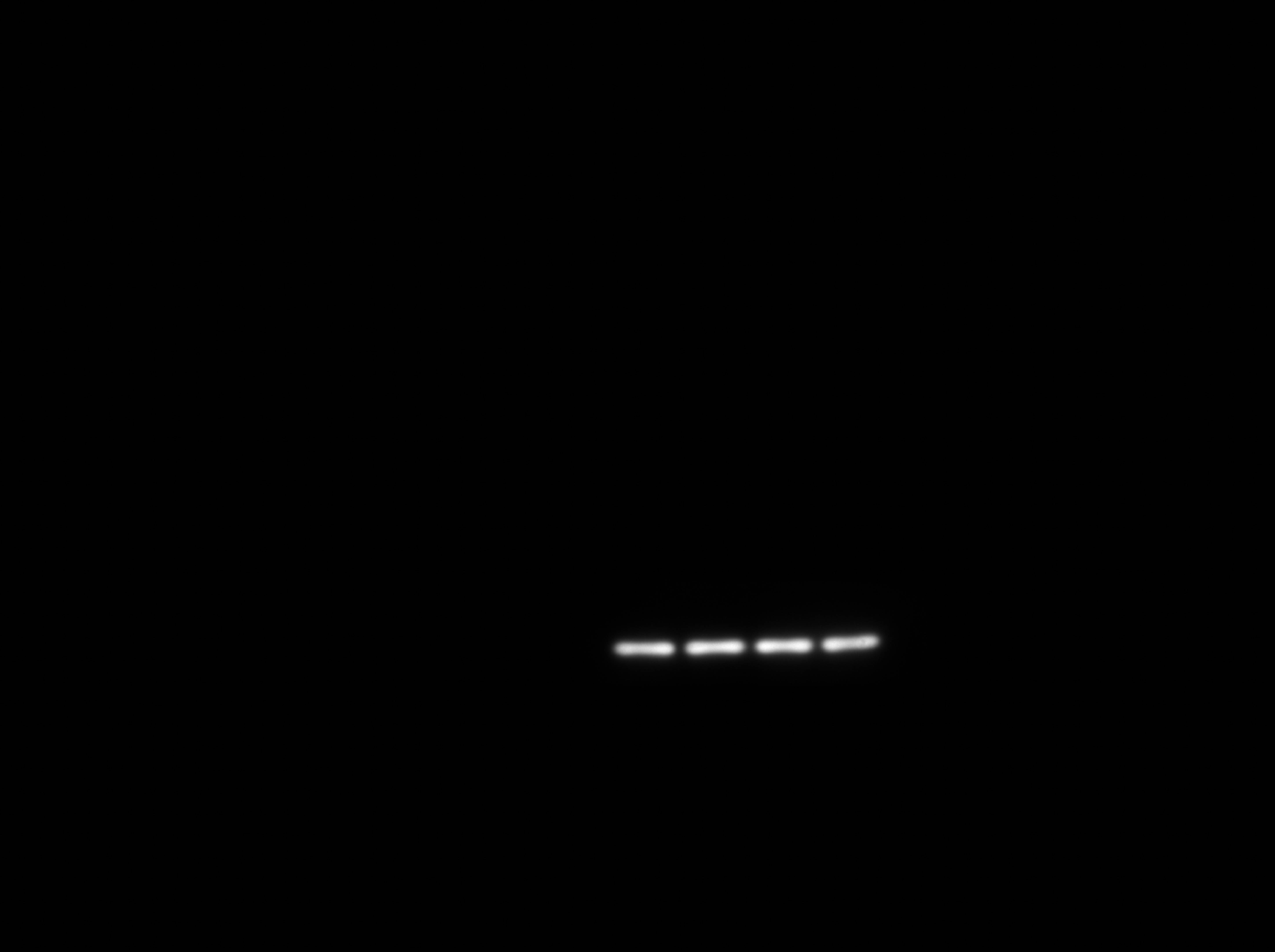

Supplement: Supplementary file 12 [file DataSheet5.ZIP › Figure2/Figure2A/GAPDH CW-2.jpg]

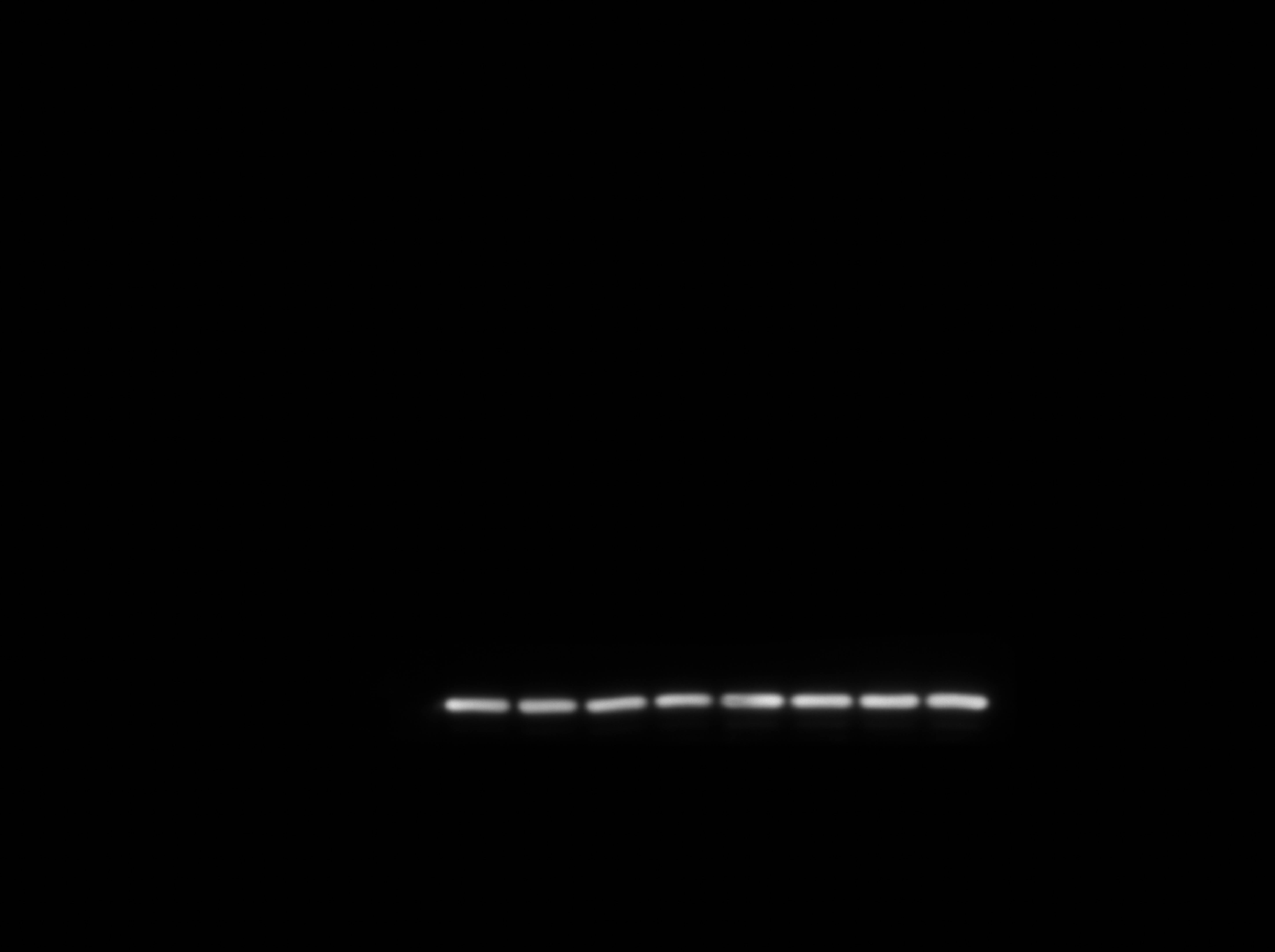

Supplement: Supplementary file 12 [file DataSheet5.ZIP › Figure2/Figure2A/GAPDH Colo205+Caco-2.jpg]

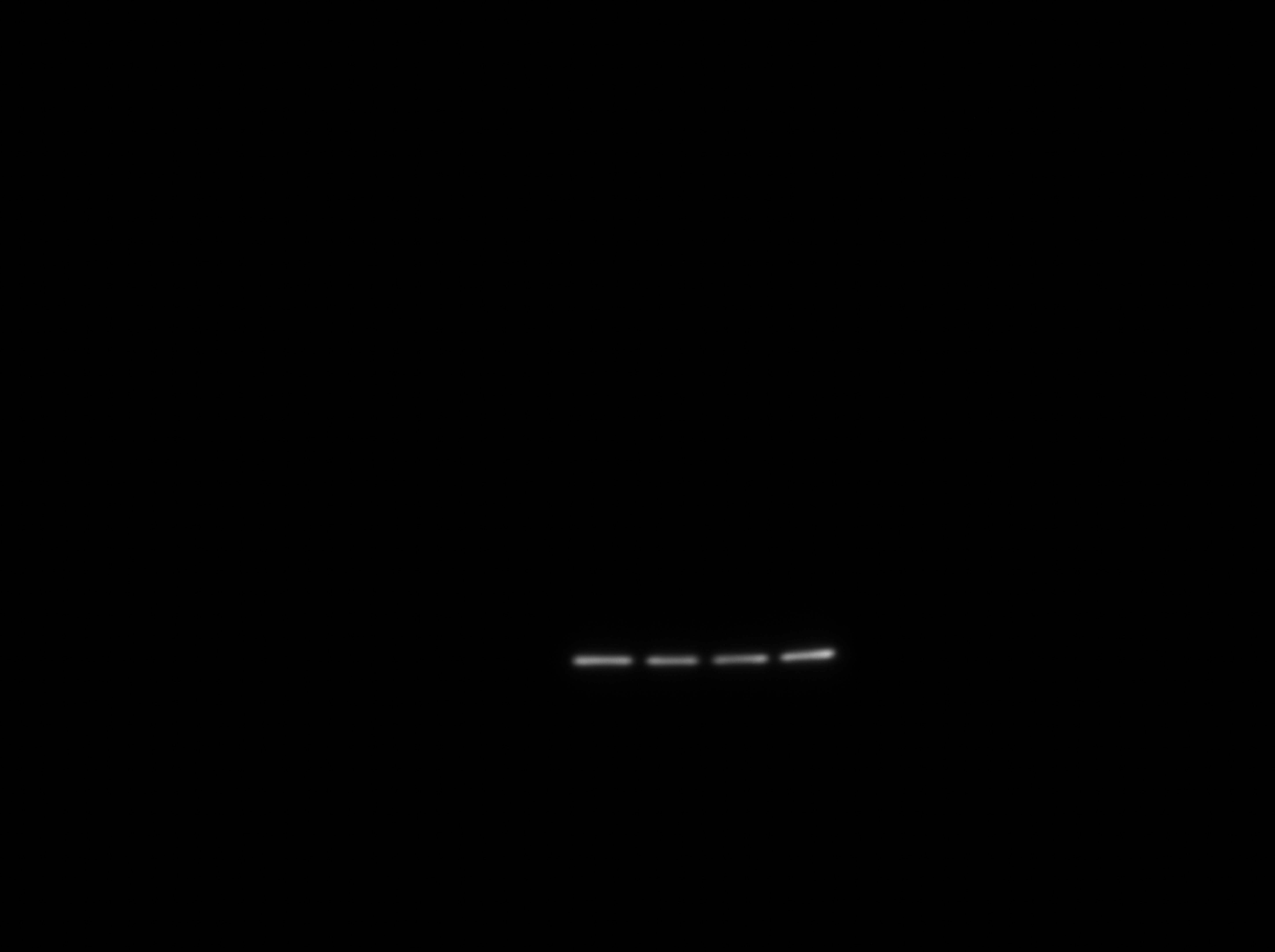

Supplement: Supplementary file 12 [file DataSheet5.ZIP › Figure2/Figure2A/GAPDH RKO.jpg]

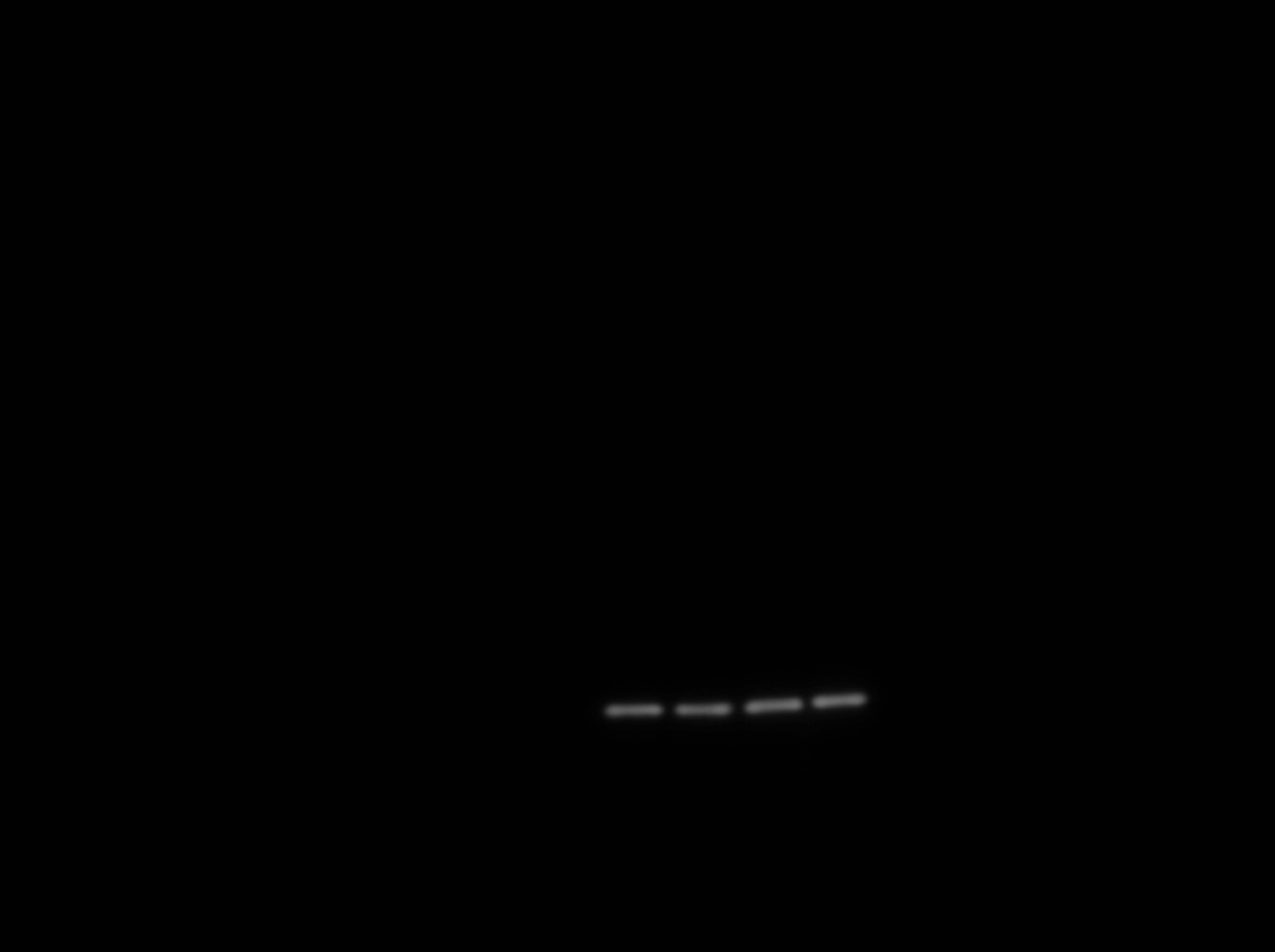

Supplement: Supplementary file 12 [file DataSheet5.ZIP › Figure2/Figure2A/GAPDH SW480.jpg]

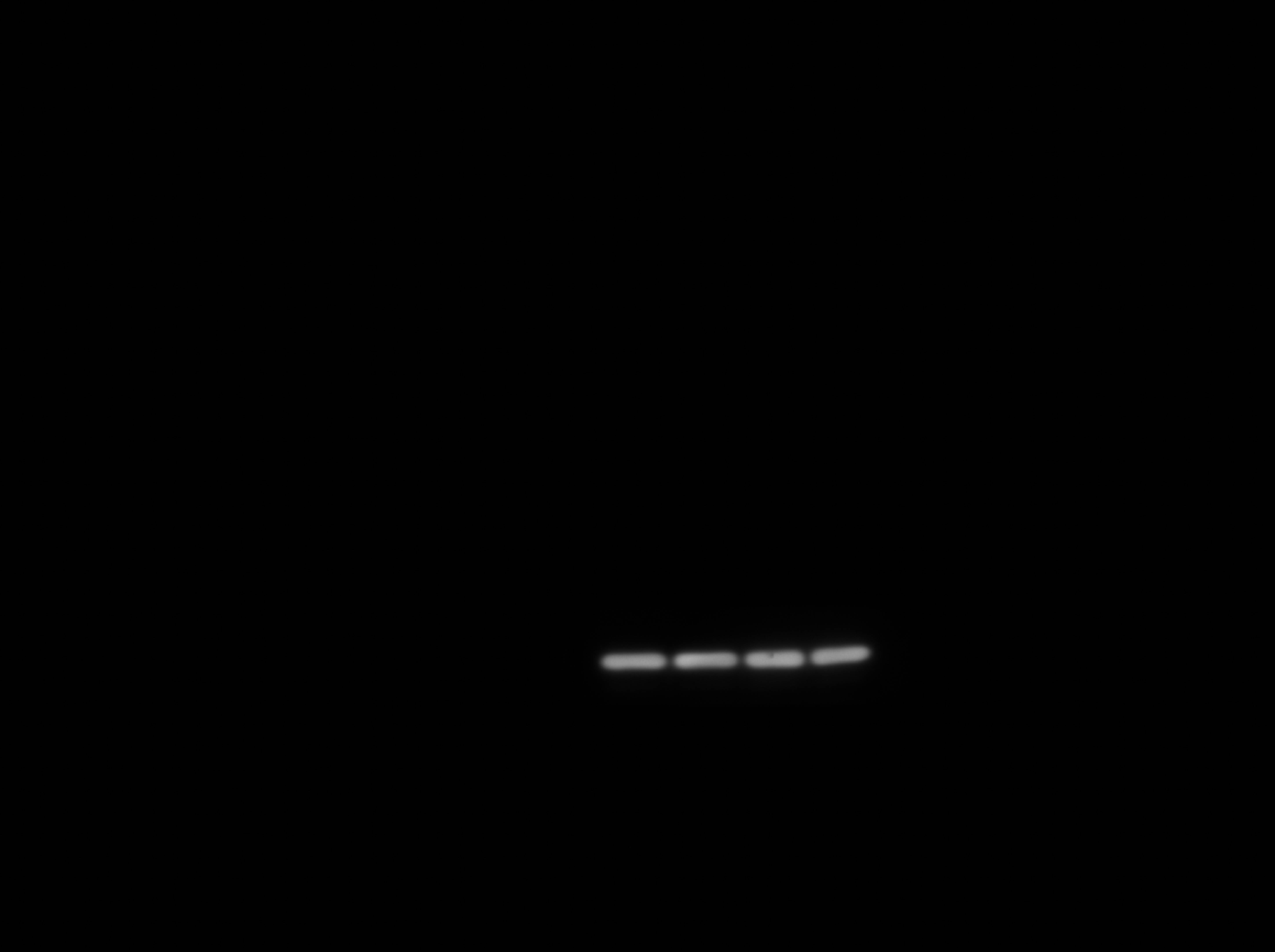

Supplement: Supplementary file 12 [file DataSheet5.ZIP › Figure2/Figure2A/GAPDH SW620.jpg]

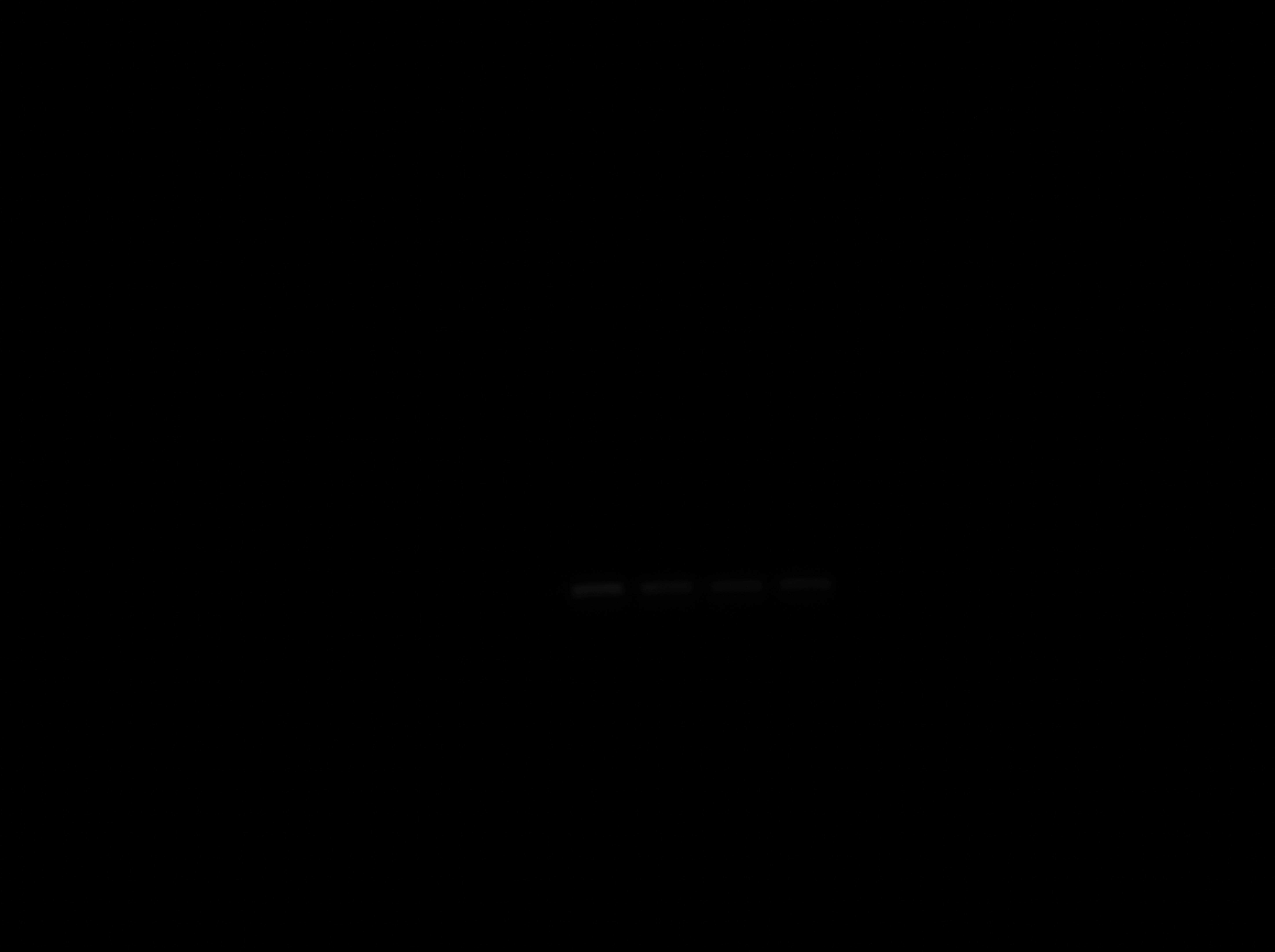

Supplement: Supplementary file 12 [file DataSheet5.ZIP › Figure2/Figure2A/P-AKT CW-2.jpg]

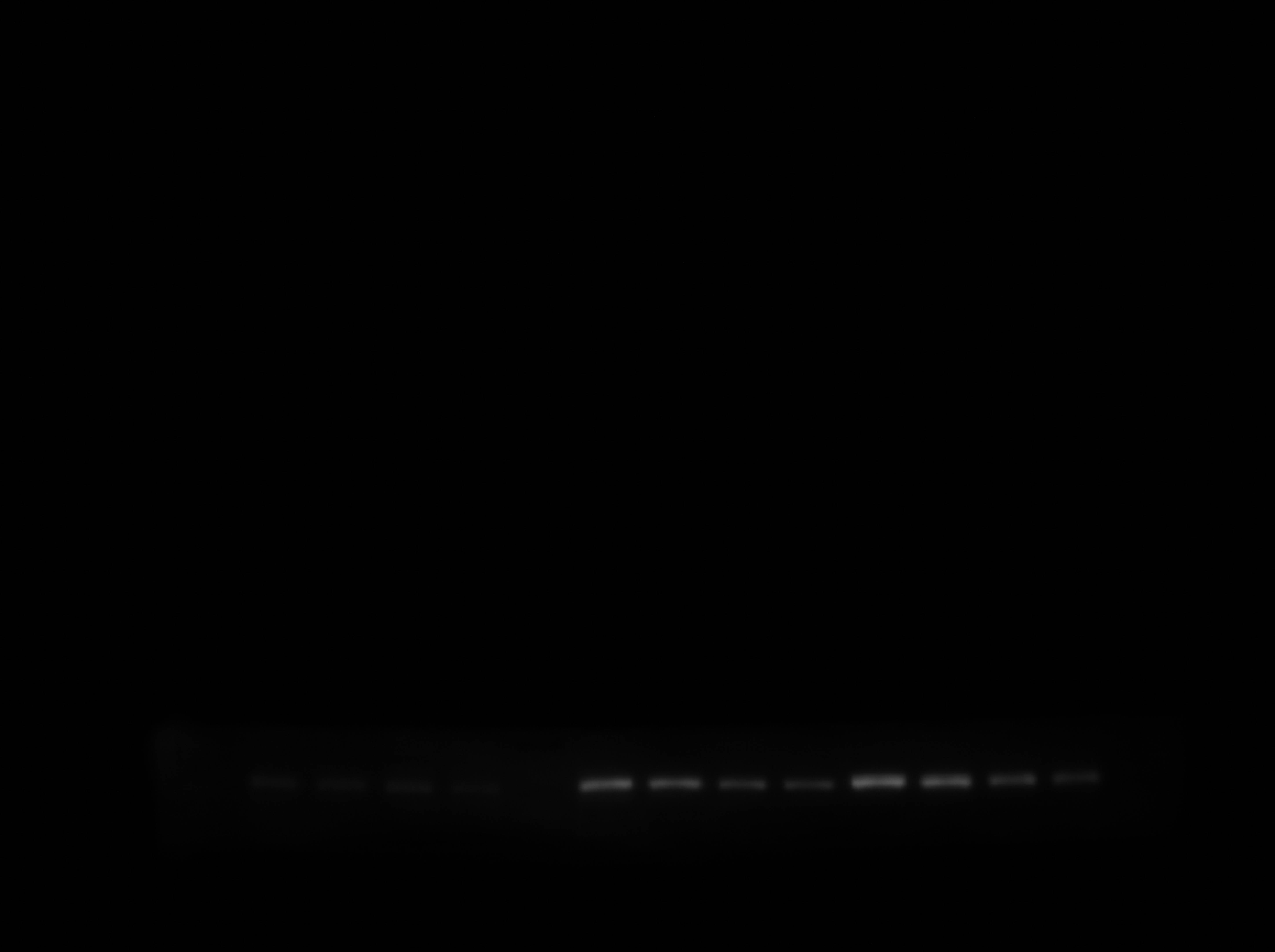

Supplement: Supplementary file 12 [file DataSheet5.ZIP › Figure2/Figure2A/P-AKT Caco-2.jpg]

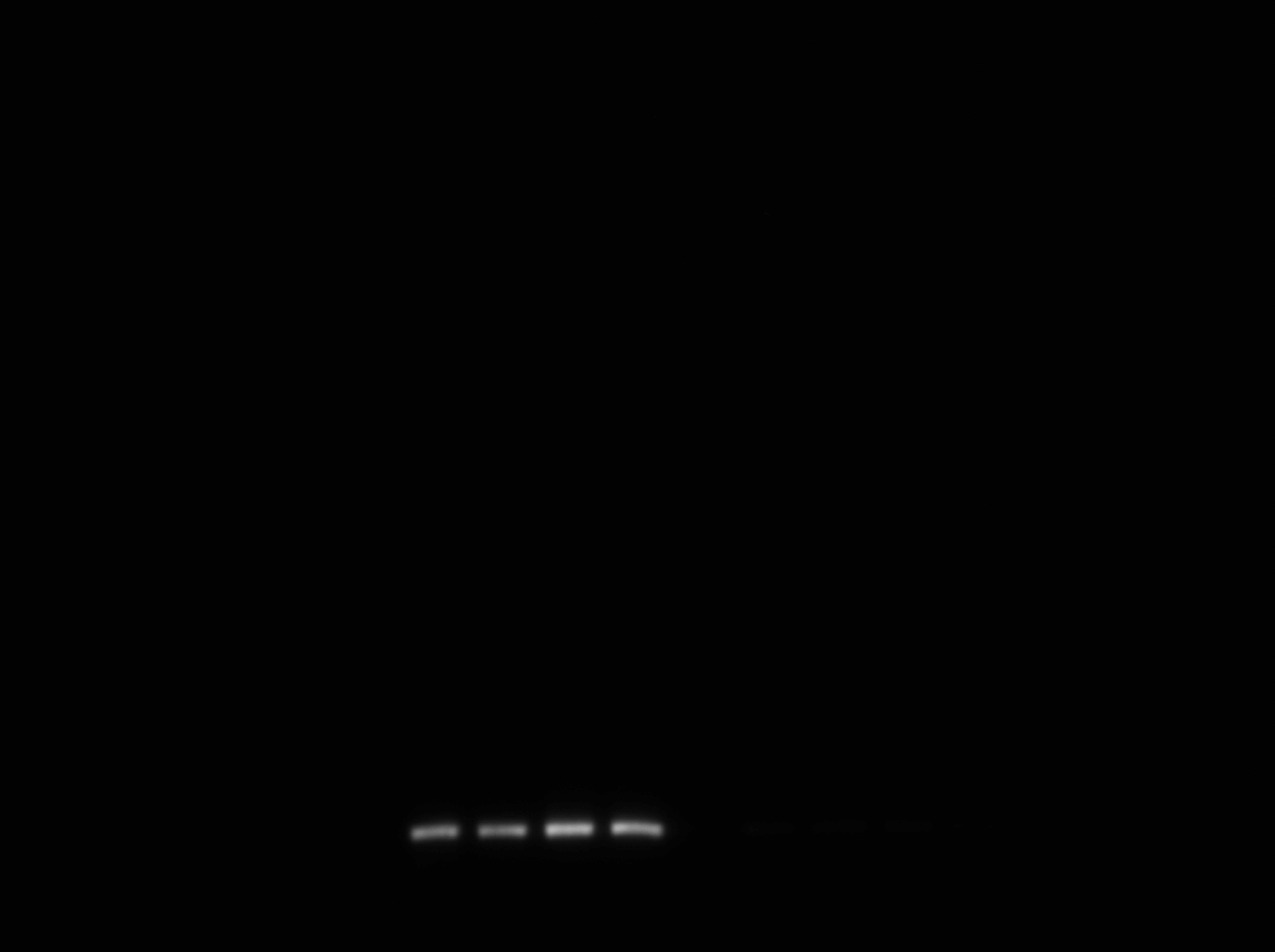

Supplement: Supplementary file 12 [file DataSheet5.ZIP › Figure2/Figure2A/P-AKT Colo205.jpg]

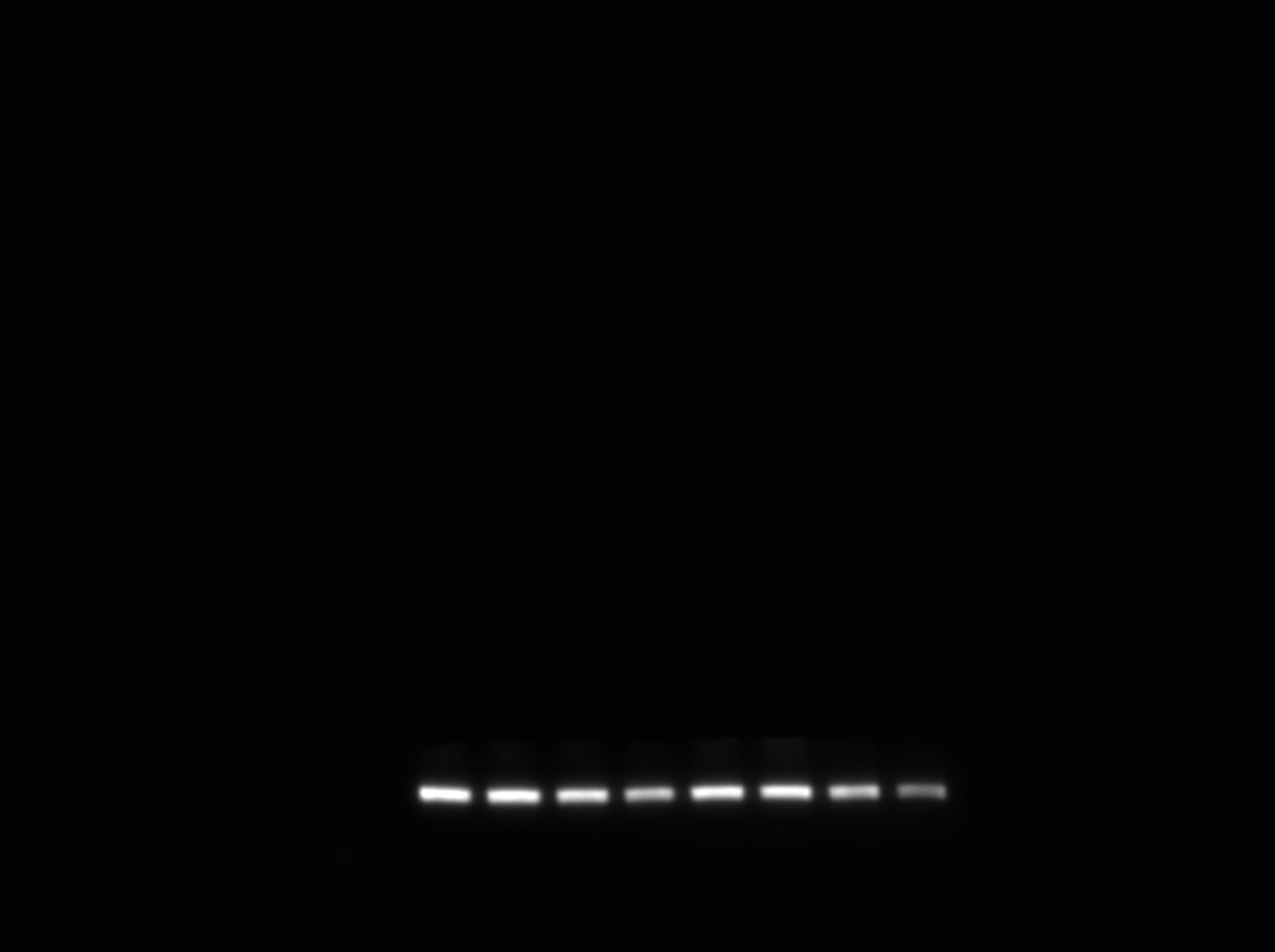

Supplement: Supplementary file 12 [file DataSheet5.ZIP › Figure2/Figure2A/P-AKT RKO.jpg]

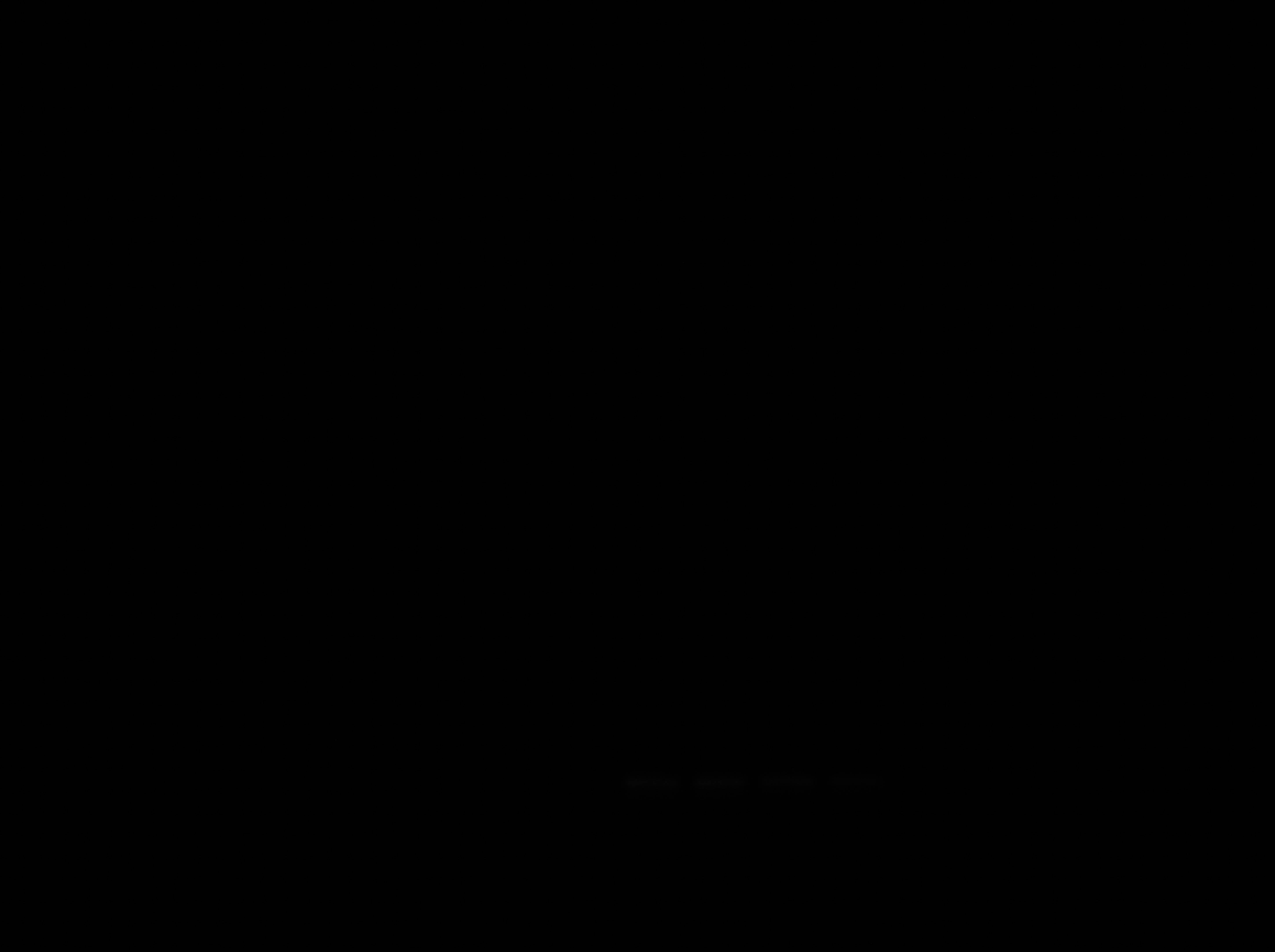

Supplement: Supplementary file 12 [file DataSheet5.ZIP › Figure2/Figure2A/P-AKT SW620.jpg]

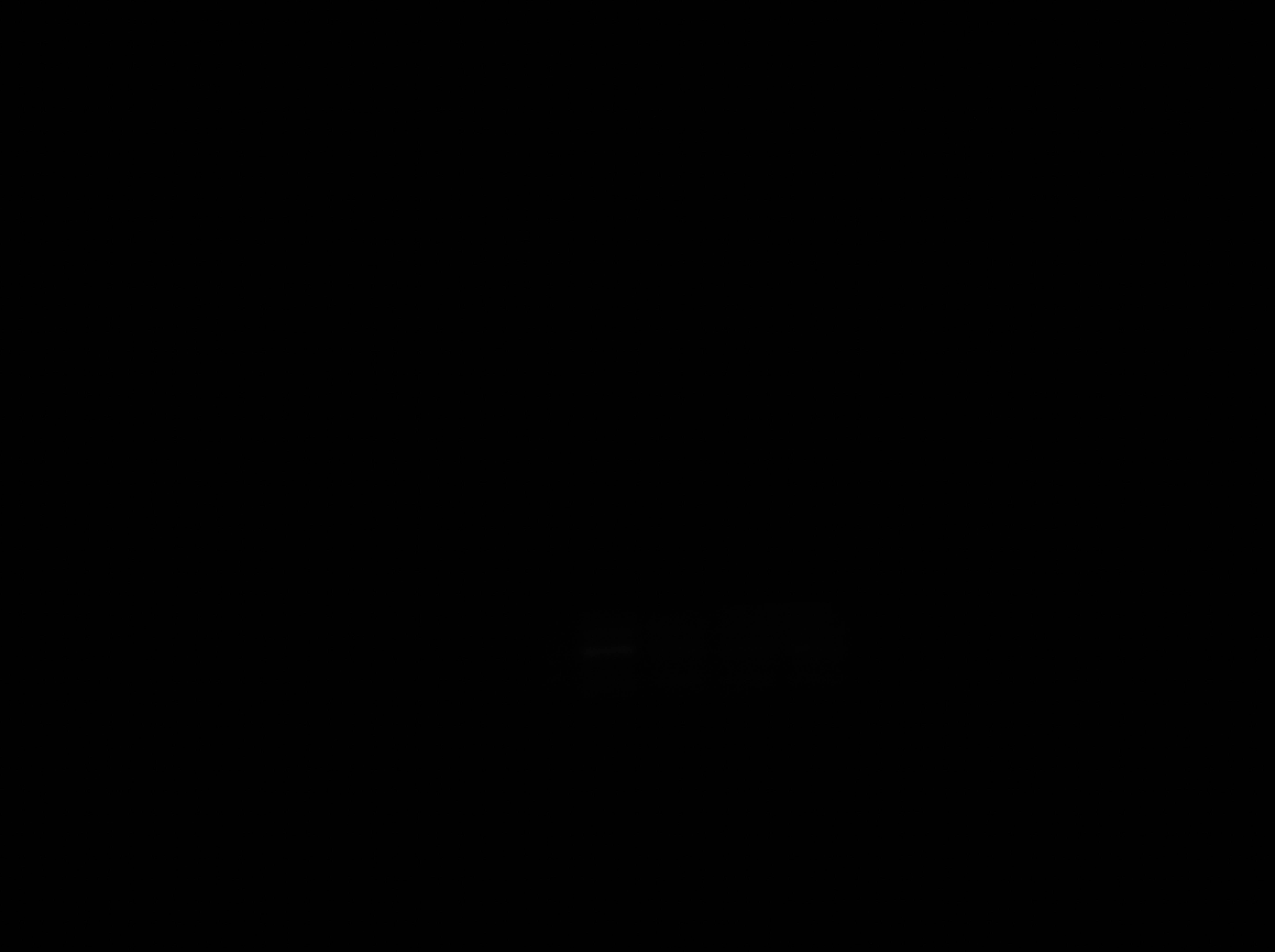

Supplement: Supplementary file 12 [file DataSheet5.ZIP › Figure2/Figure2A/P-ERK CW-2.jpg]

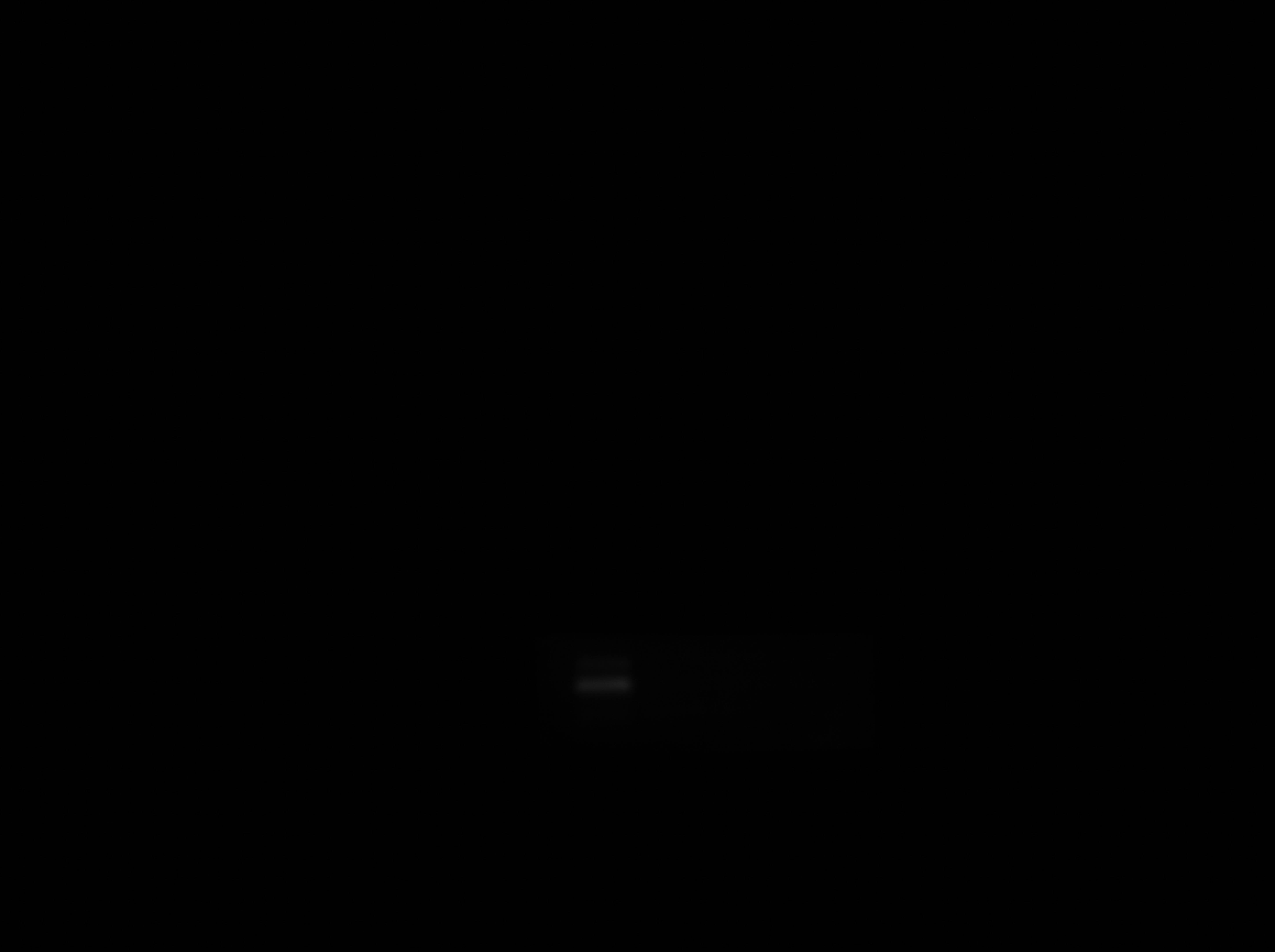

Supplement: Supplementary file 12 [file DataSheet5.ZIP › Figure2/Figure2A/P-ERK Caco-2.jpg]

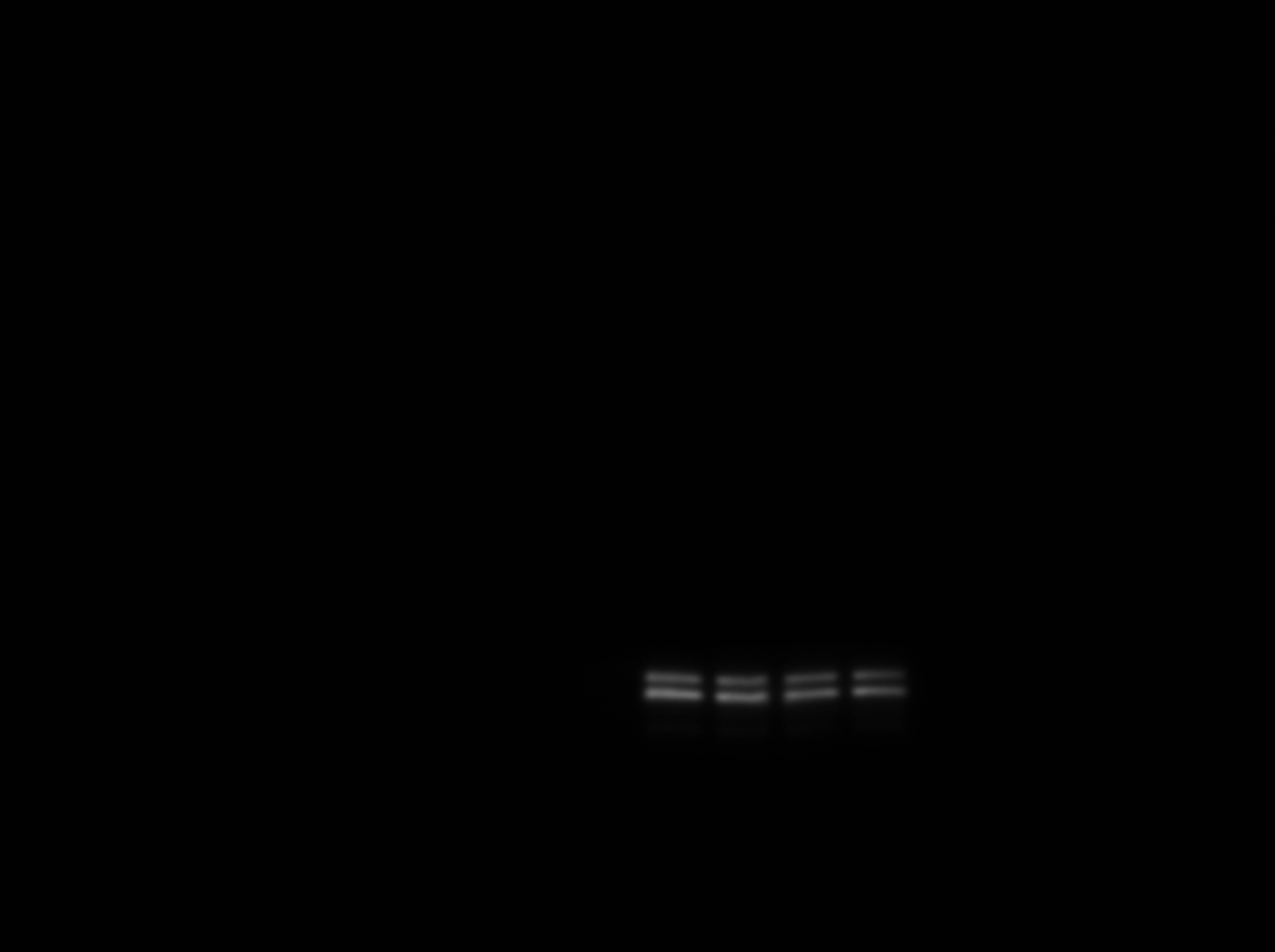

Supplement: Supplementary file 12 [file DataSheet5.ZIP › Figure2/Figure2A/P-ERK Colo205.jpg]

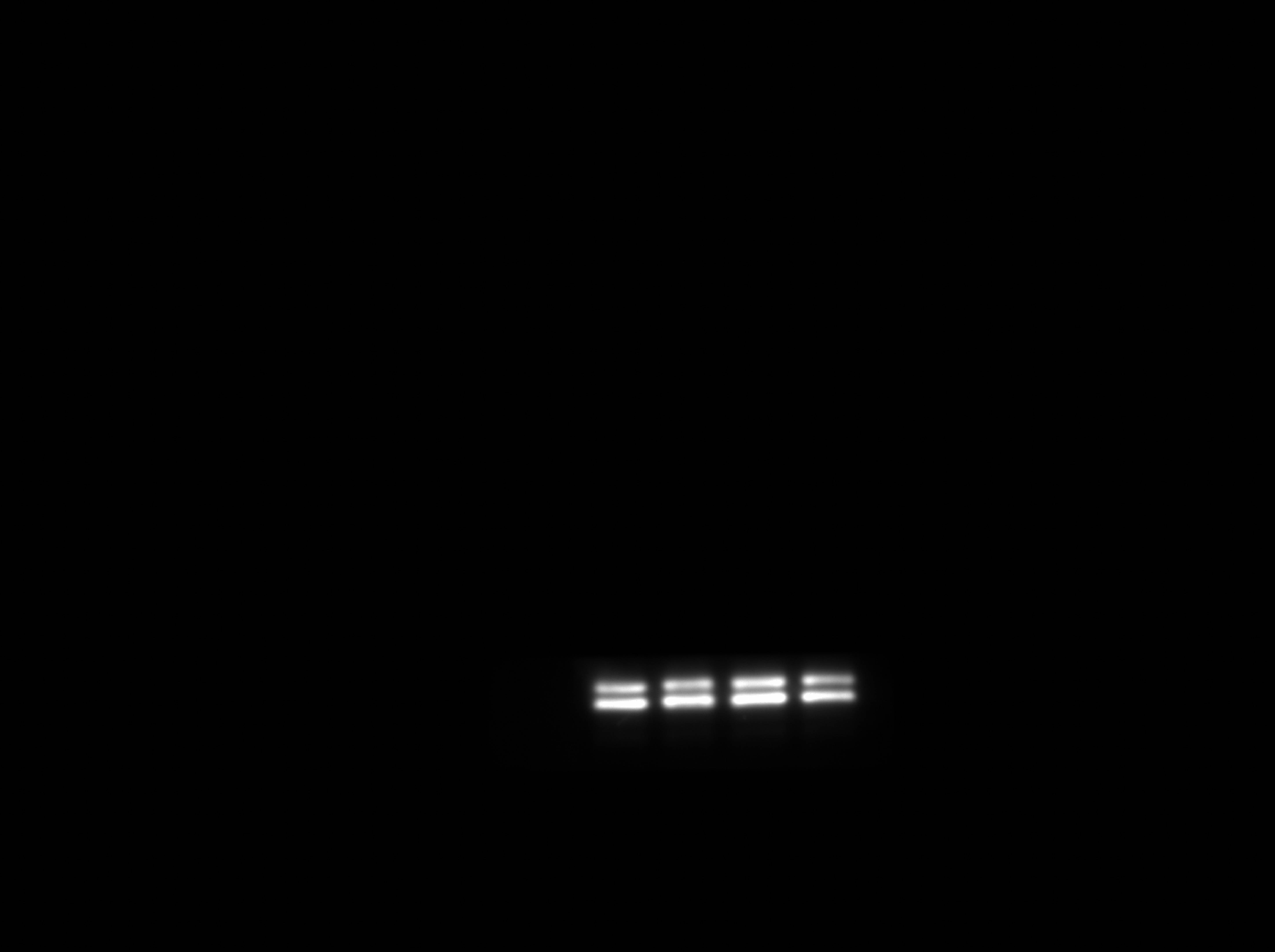

Supplement: Supplementary file 12 [file DataSheet5.ZIP › Figure2/Figure2A/P-ERK RKO.jpg]

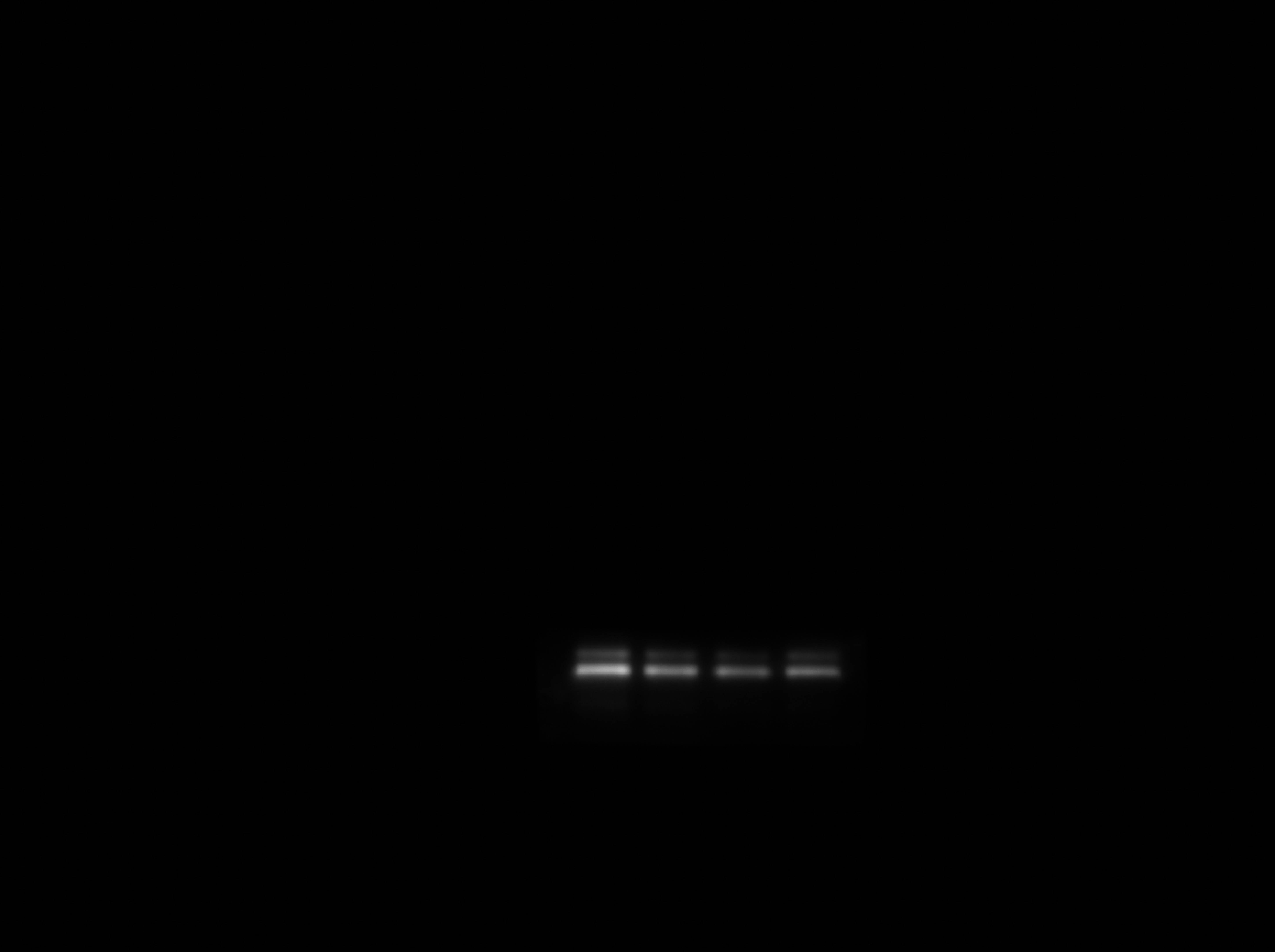

Supplement: Supplementary file 12 [file DataSheet5.ZIP › Figure2/Figure2A/P-ERK SW480.jpg]

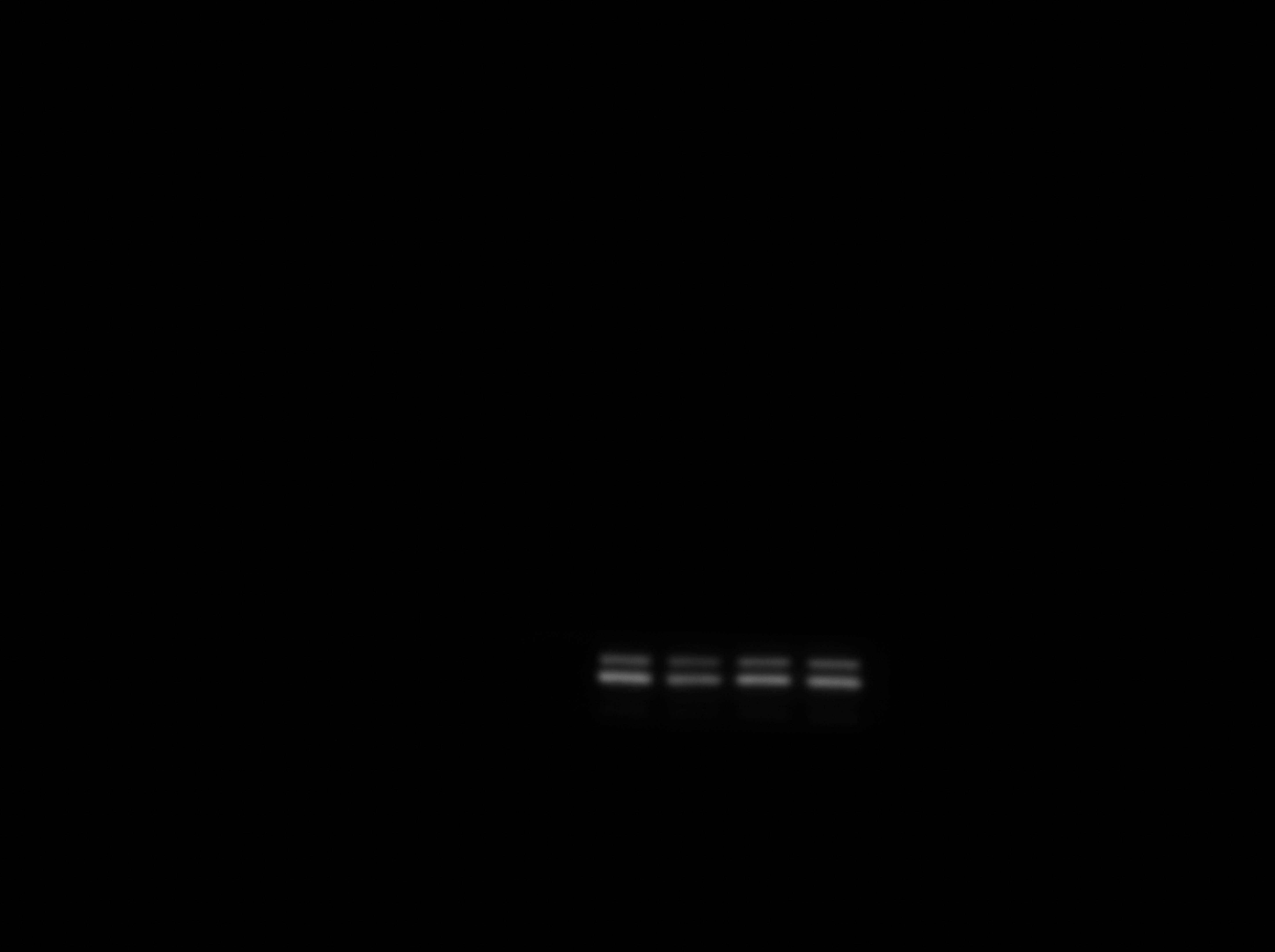

Supplement: Supplementary file 12 [file DataSheet5.ZIP › Figure2/Figure2A/P-ERK SW620.jpg]

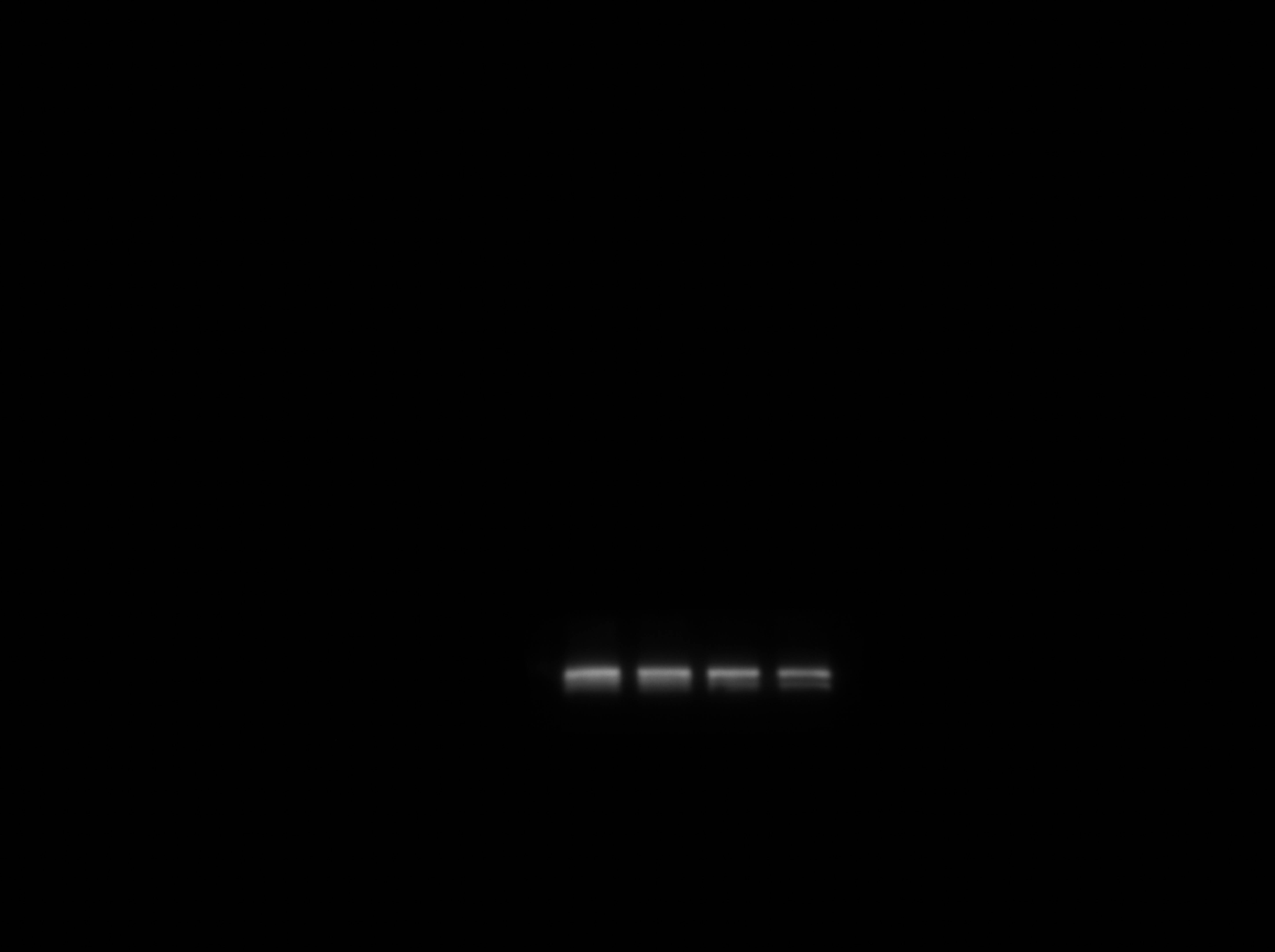

Supplement: Supplementary file 12 [file DataSheet5.ZIP › Figure2/Figure2A/p-AKT SW480.jpg]

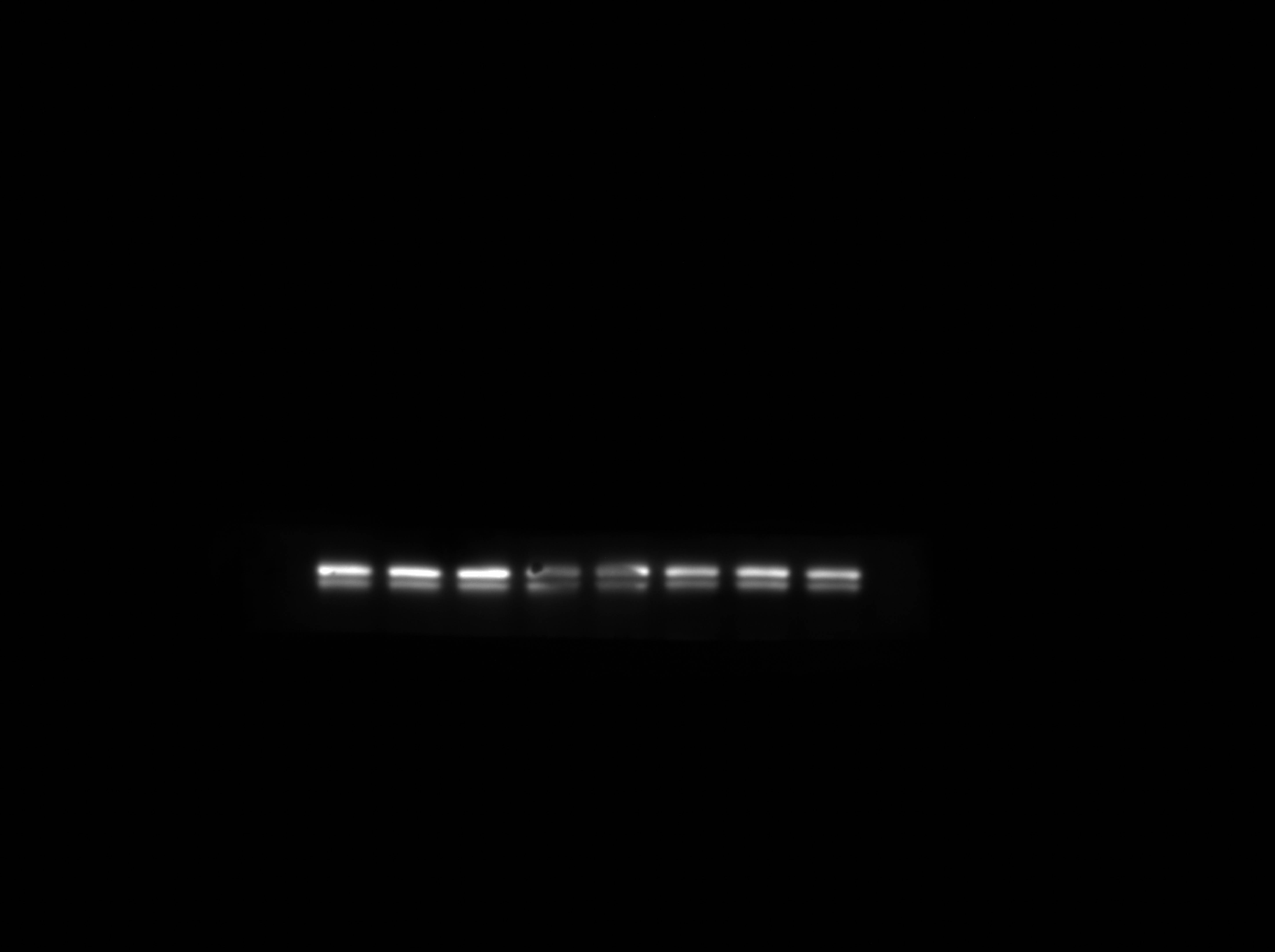

Supplement: Supplementary file 12 [file DataSheet5.ZIP › Figure2/Figure2B/AKT CW-2.jpg]

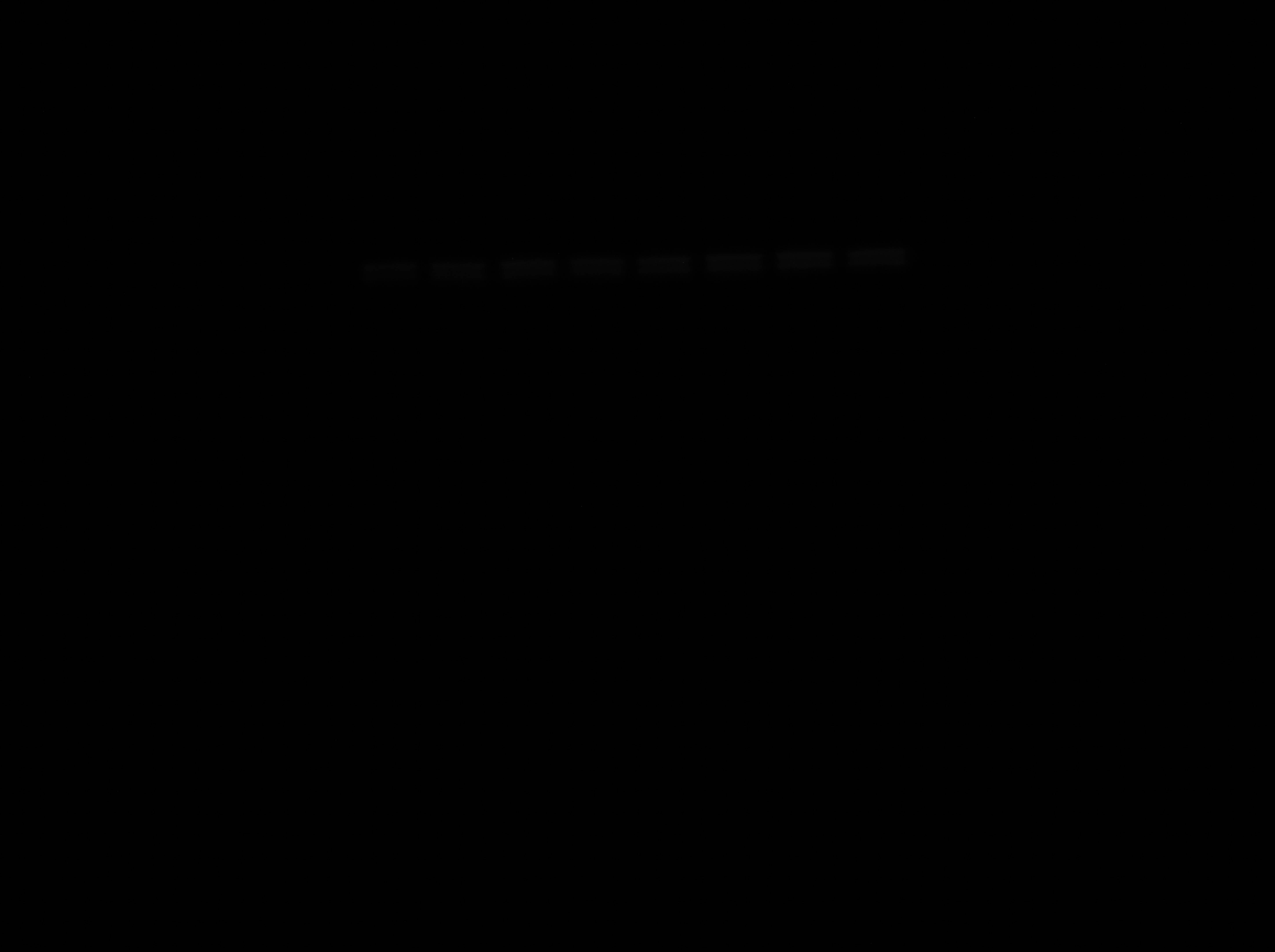

Supplement: Supplementary file 12 [file DataSheet5.ZIP › Figure2/Figure2B/AKT Caco-2.jpg]

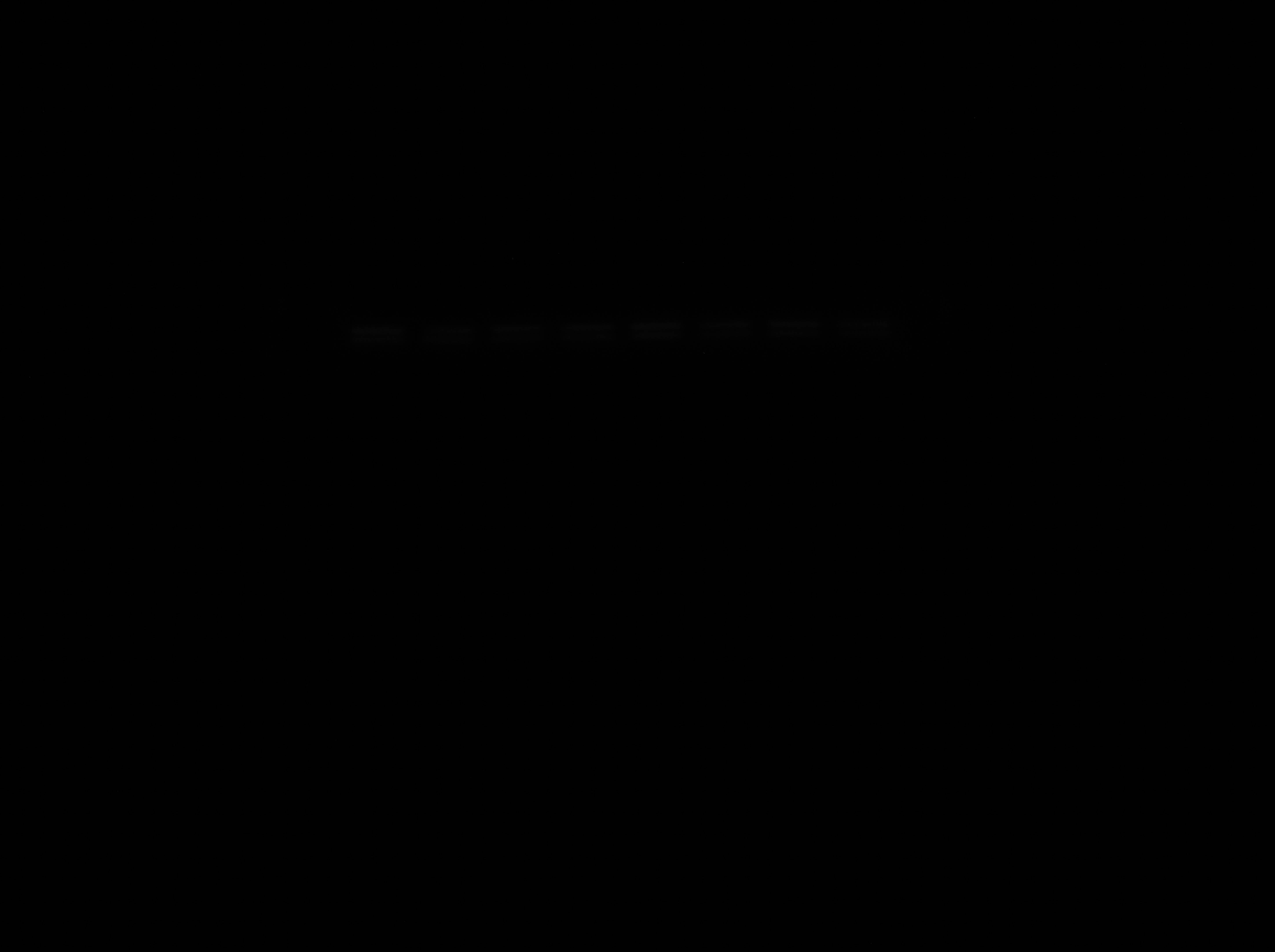

Supplement: Supplementary file 12 [file DataSheet5.ZIP › Figure2/Figure2B/AKT Colo205.jpg]

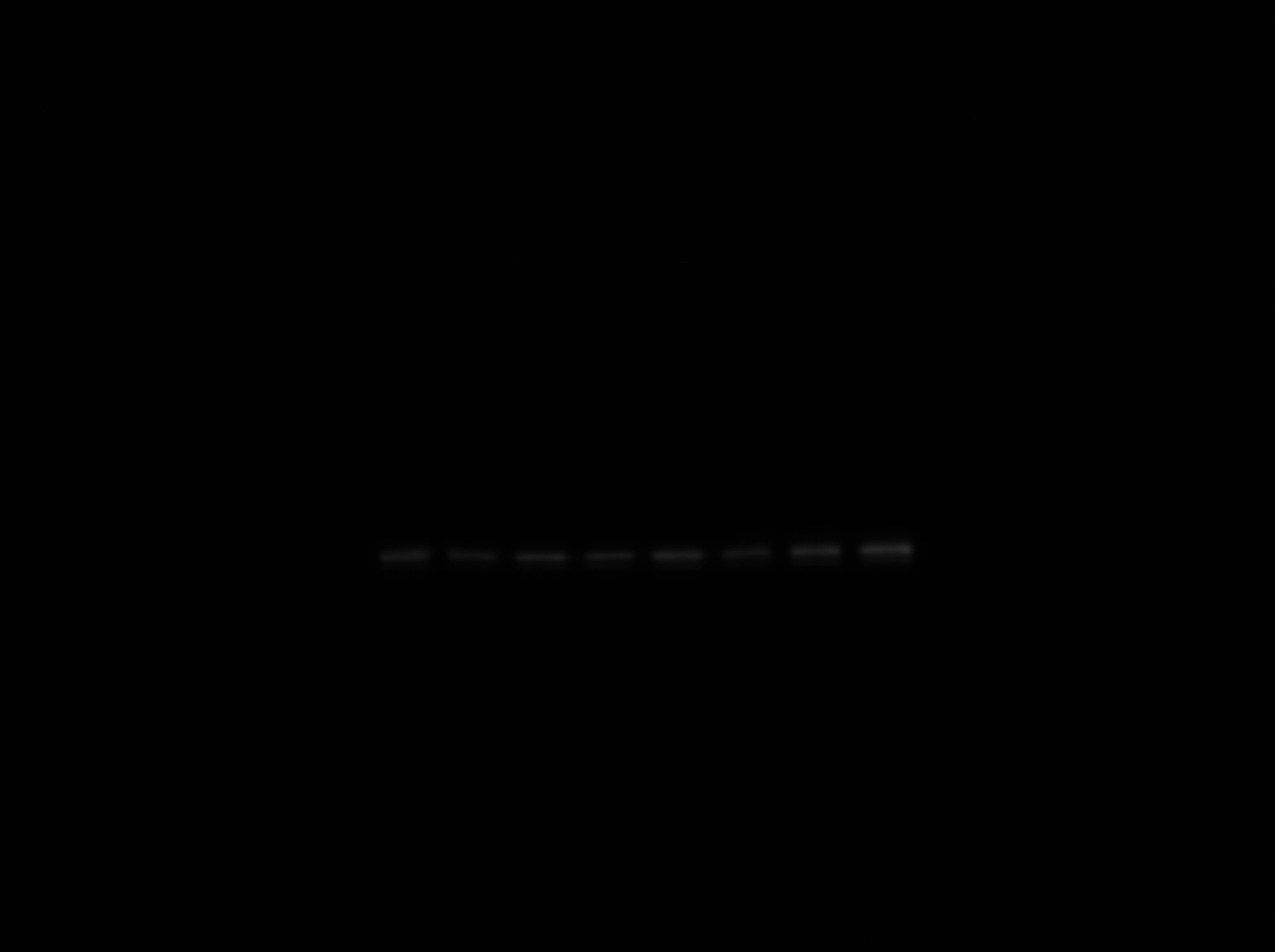

Supplement: Supplementary file 12 [file DataSheet5.ZIP › Figure2/Figure2B/AKT RKO.jpg]

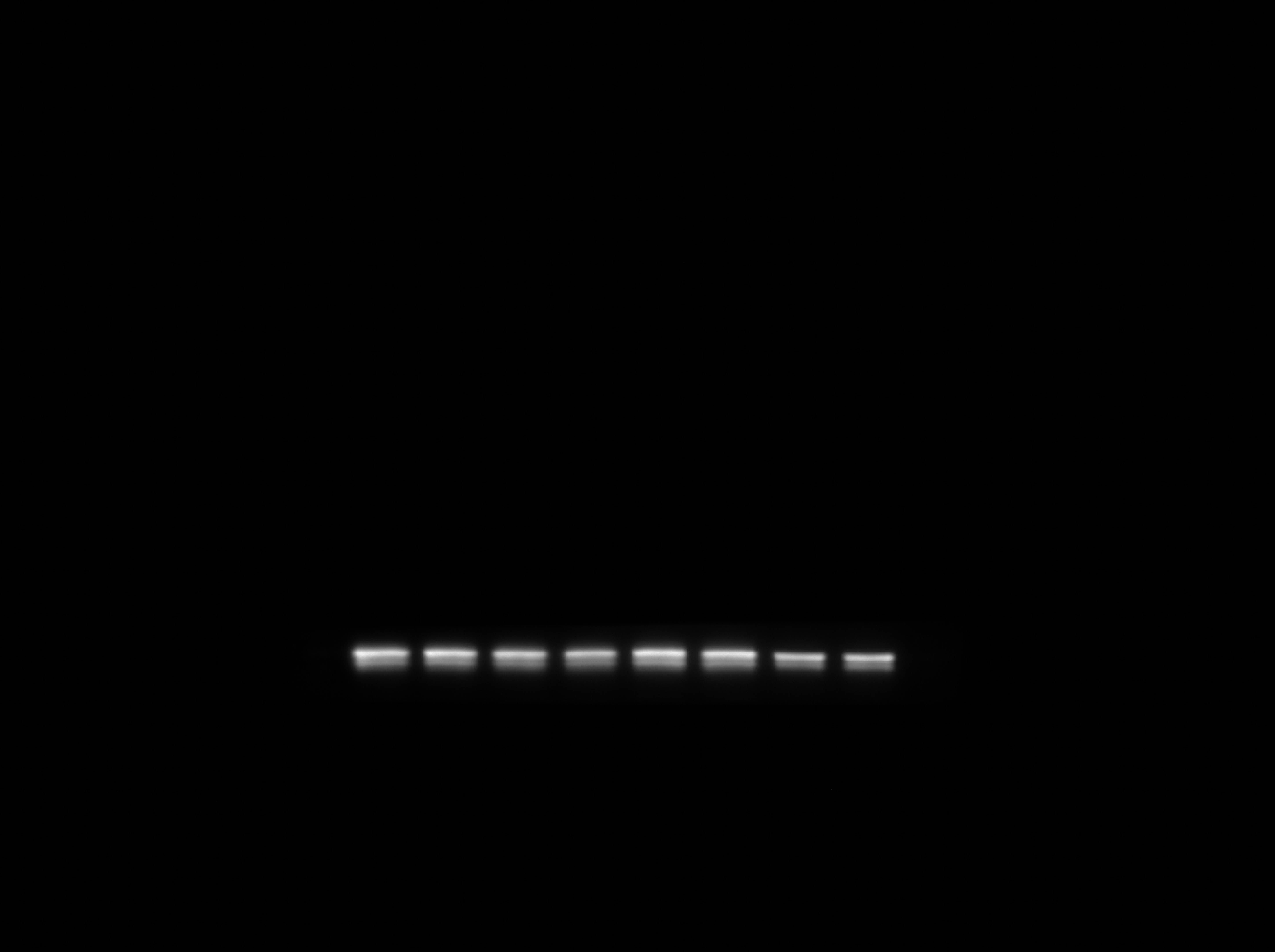

Supplement: Supplementary file 12 [file DataSheet5.ZIP › Figure2/Figure2B/AKT SW480.jpg]

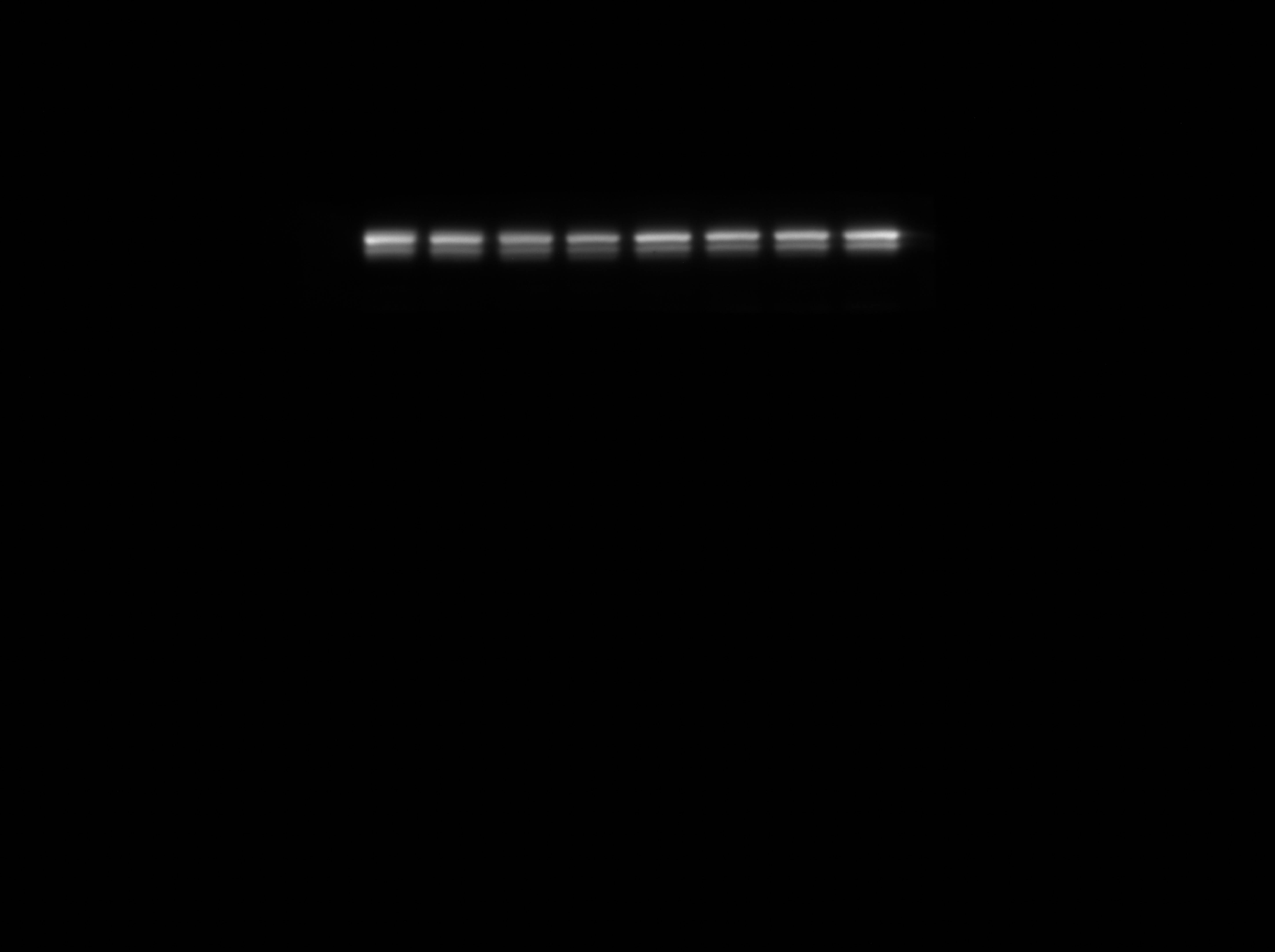

Supplement: Supplementary file 12 [file DataSheet5.ZIP › Figure2/Figure2B/AKT SW620.jpg]

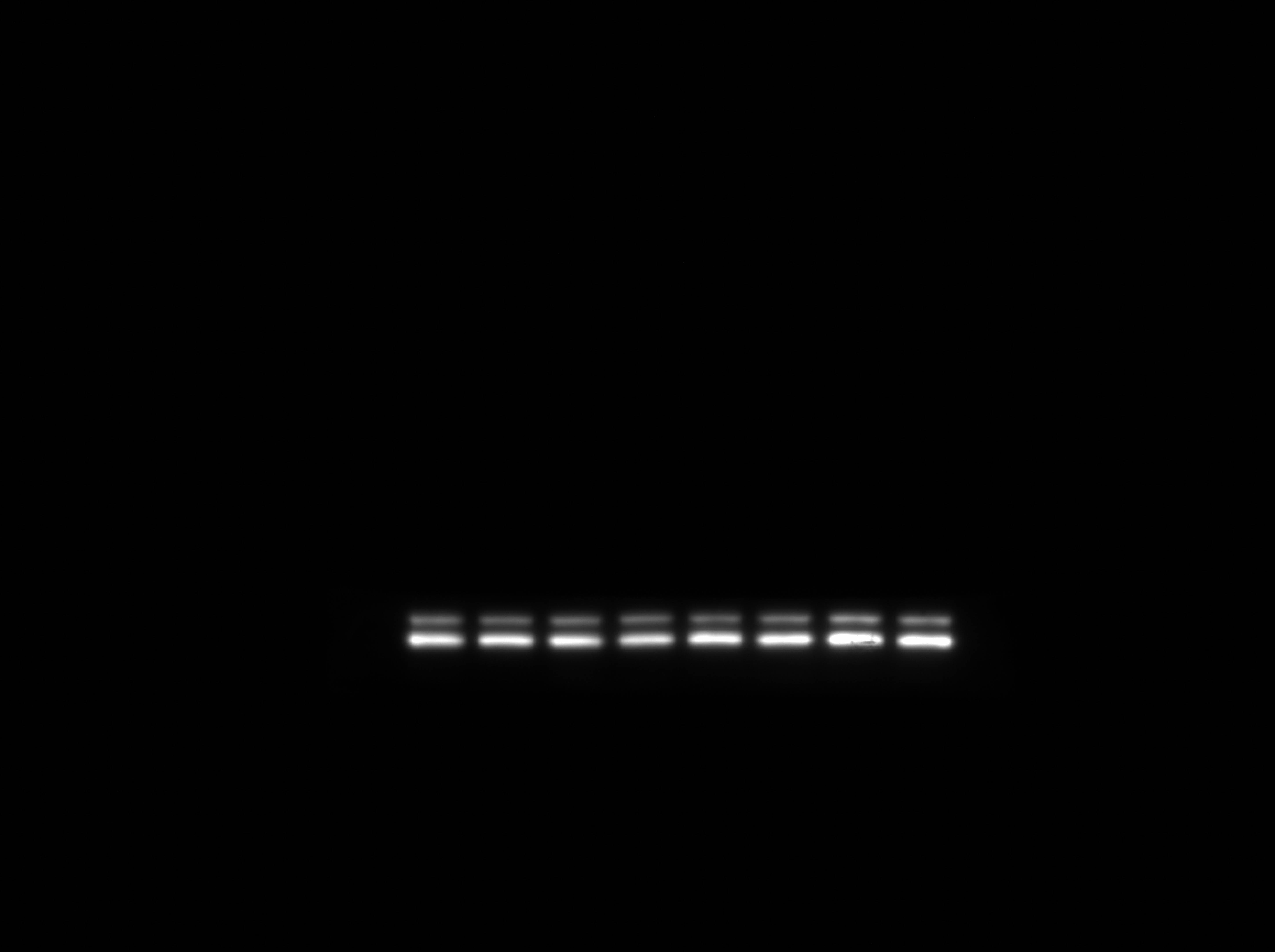

Supplement: Supplementary file 12 [file DataSheet5.ZIP › Figure2/Figure2B/ERK CW-2.jpg]

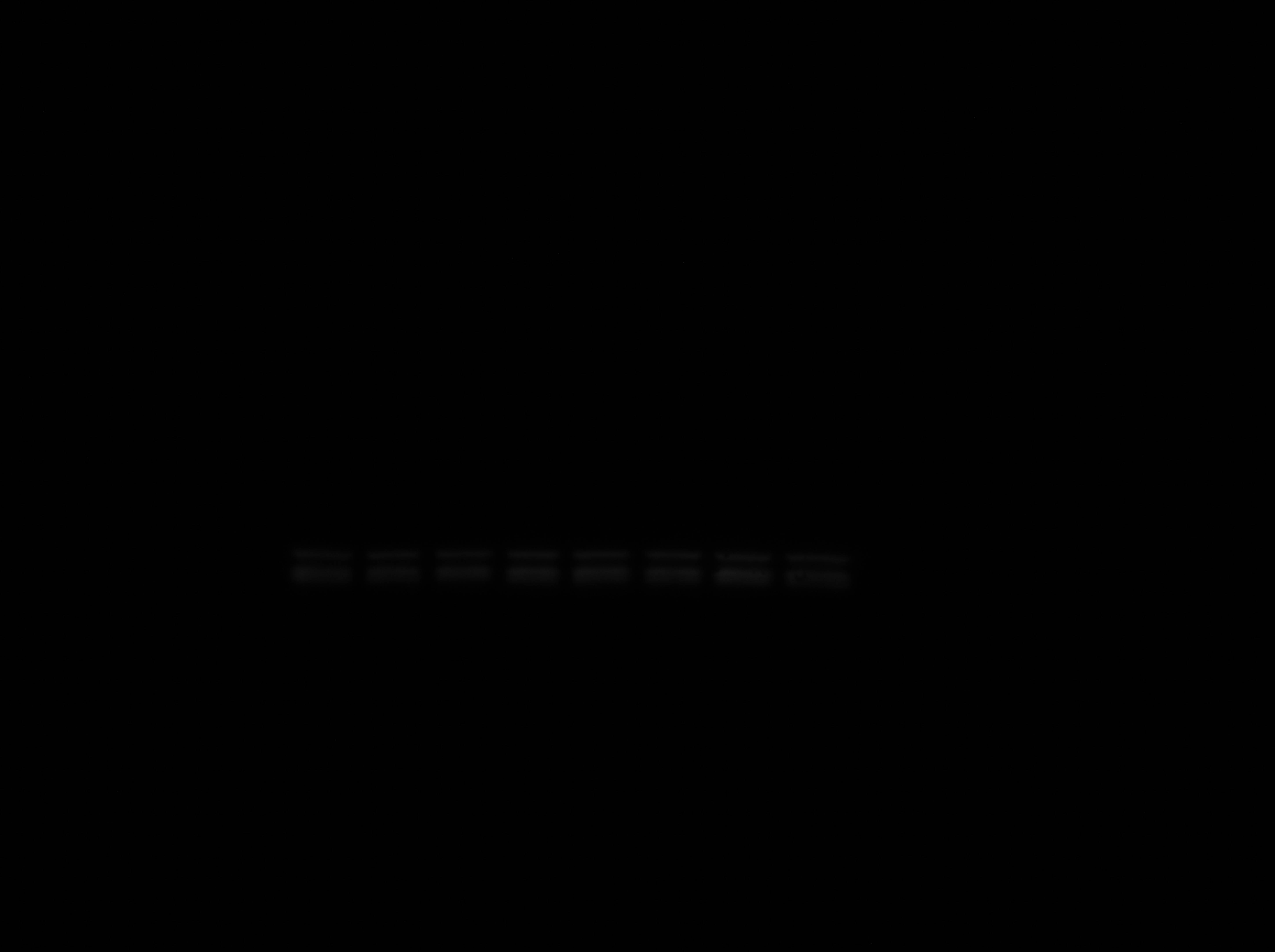

Supplement: Supplementary file 12 [file DataSheet5.ZIP › Figure2/Figure2B/ERK Caco-2.jpg]

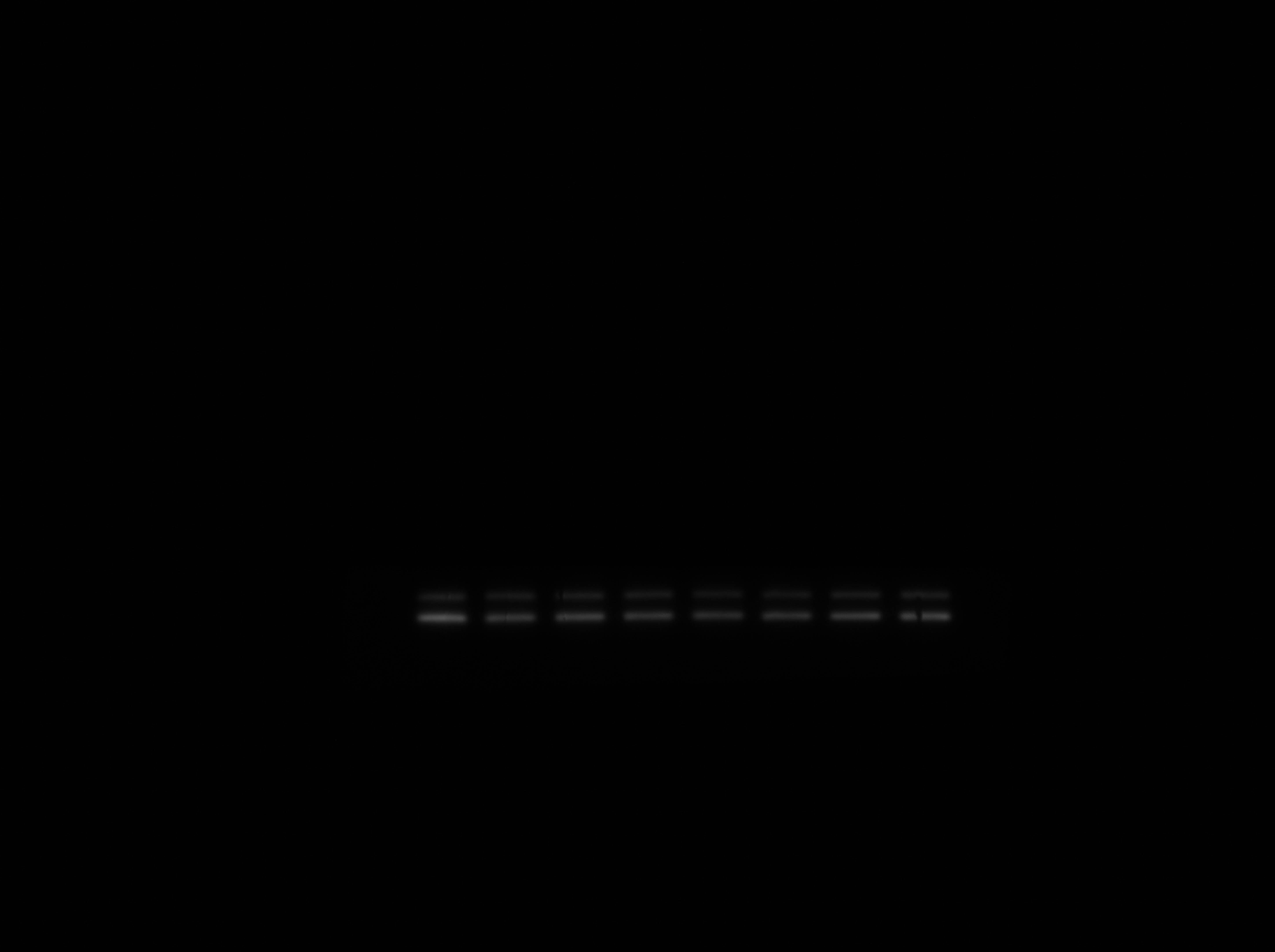

Supplement: Supplementary file 12 [file DataSheet5.ZIP › Figure2/Figure2B/ERK Colo205.jpg]

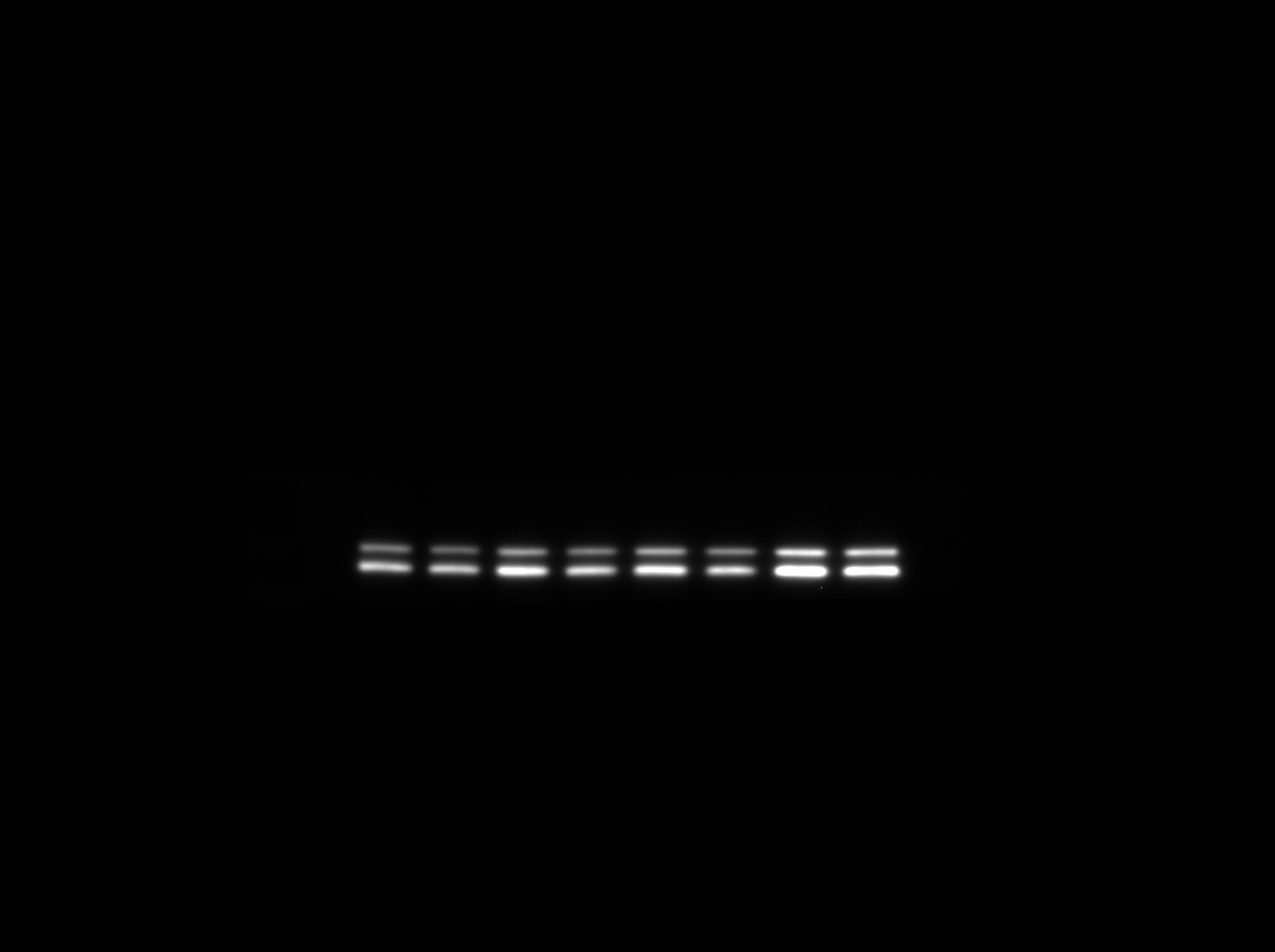

Supplement: Supplementary file 12 [file DataSheet5.ZIP › Figure2/Figure2B/ERK RKO.jpg]

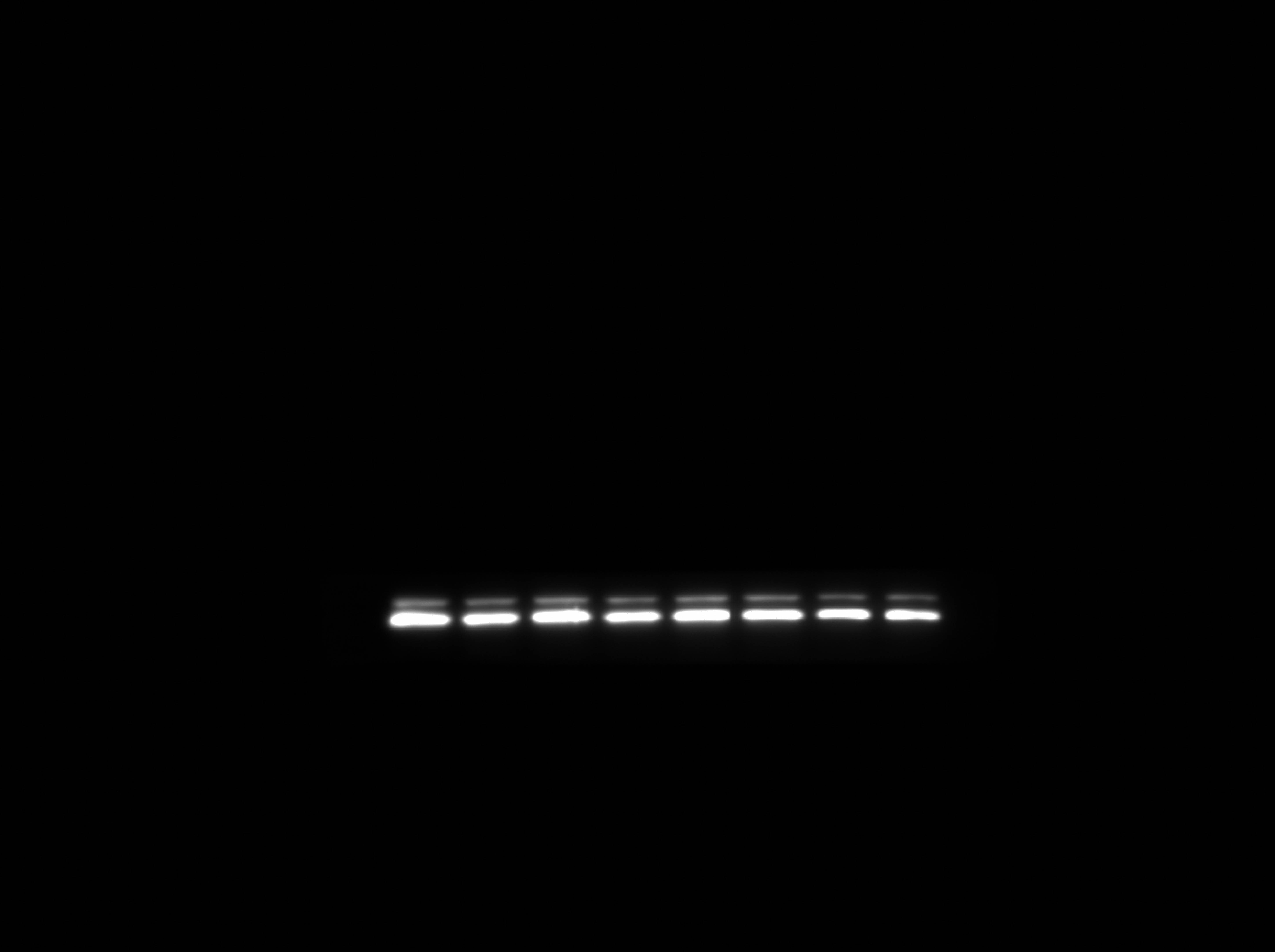

Supplement: Supplementary file 12 [file DataSheet5.ZIP › Figure2/Figure2B/ERK SW480.jpg]

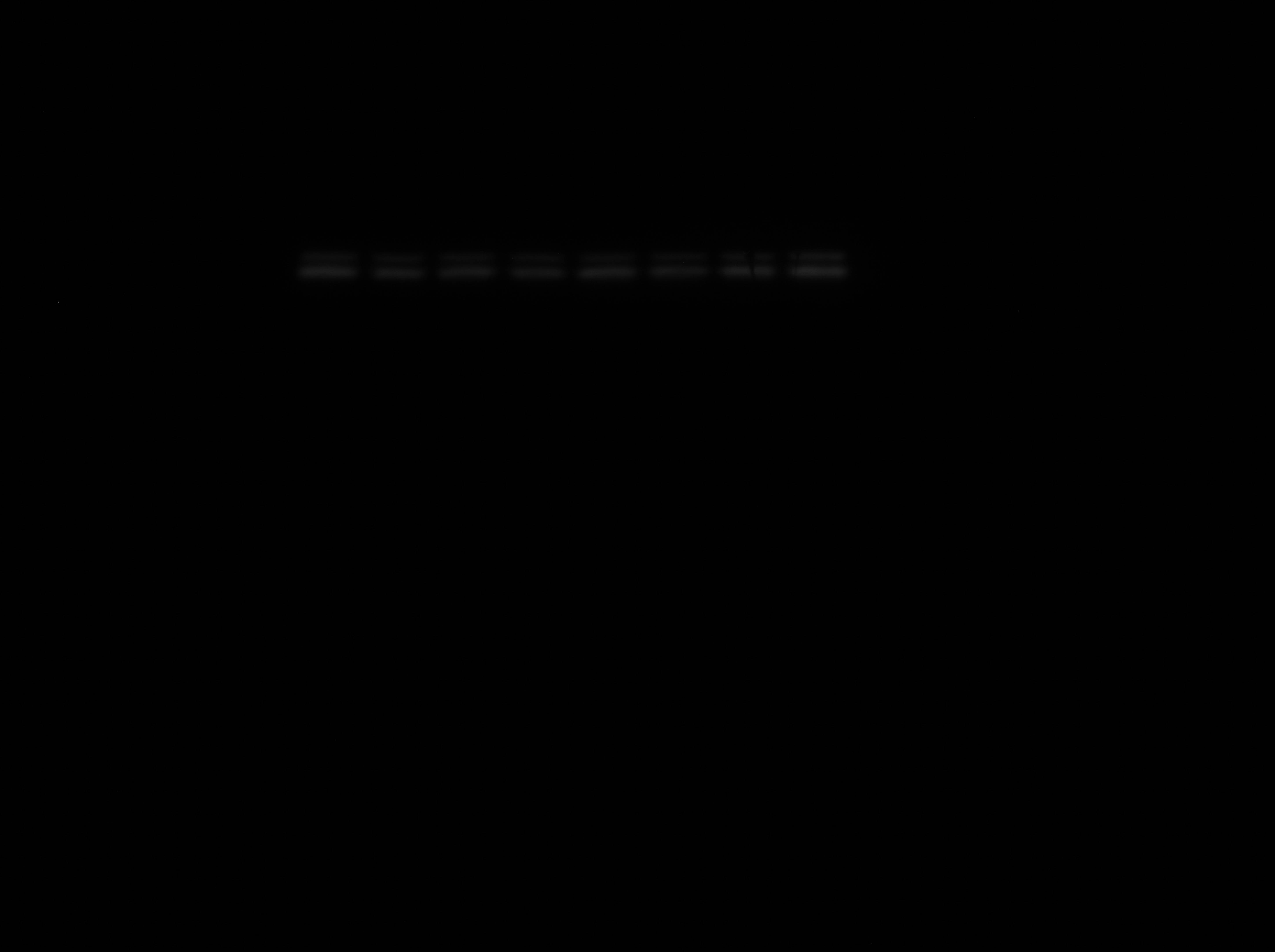

Supplement: Supplementary file 12 [file DataSheet5.ZIP › Figure2/Figure2B/ERK SW620.jpg]

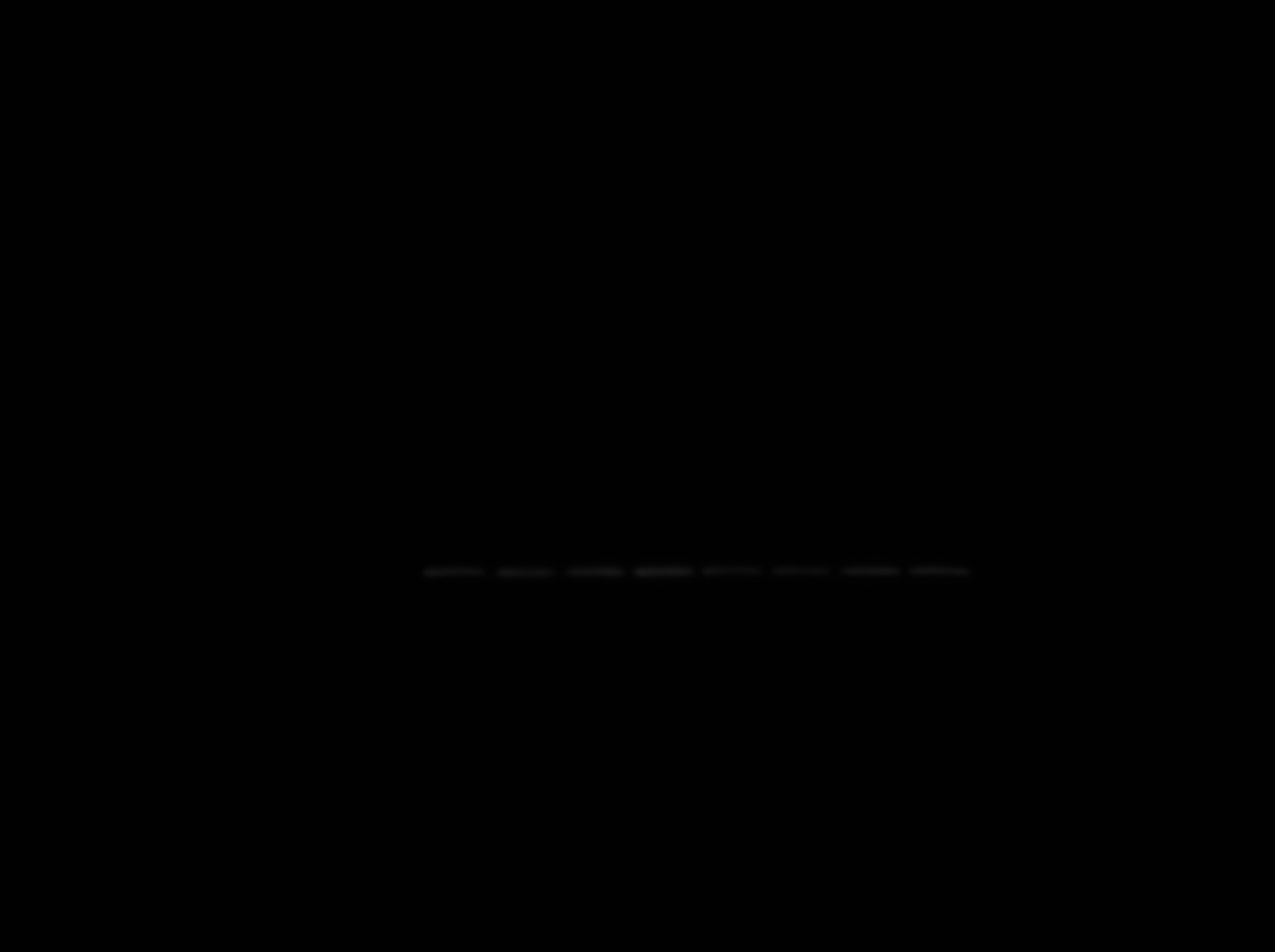

Supplement: Supplementary file 12 [file DataSheet5.ZIP › Figure2/Figure2B/GAPDH CW-2.jpg]

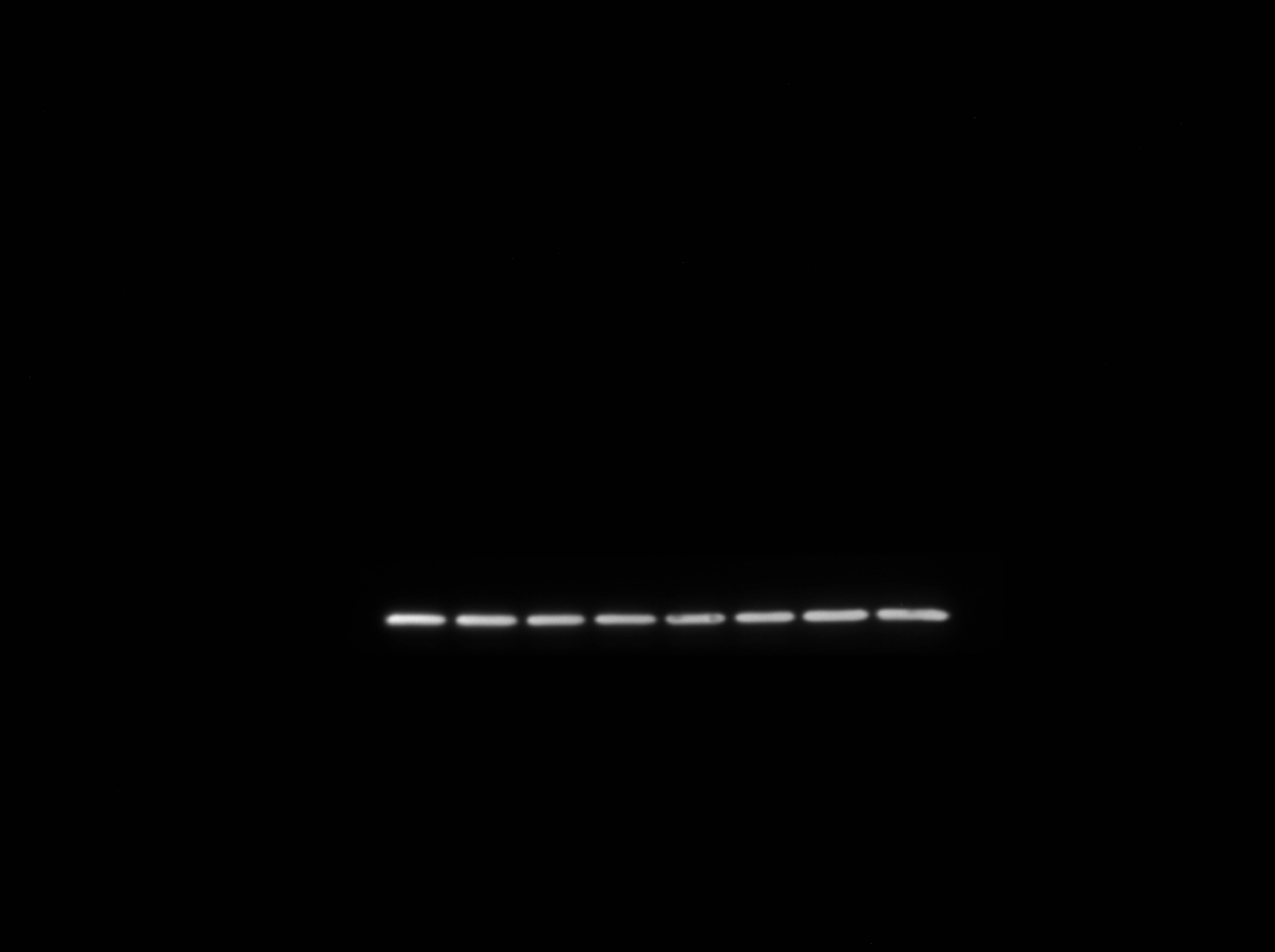

Supplement: Supplementary file 12 [file DataSheet5.ZIP › Figure2/Figure2B/GAPDH Caco-2.jpg]

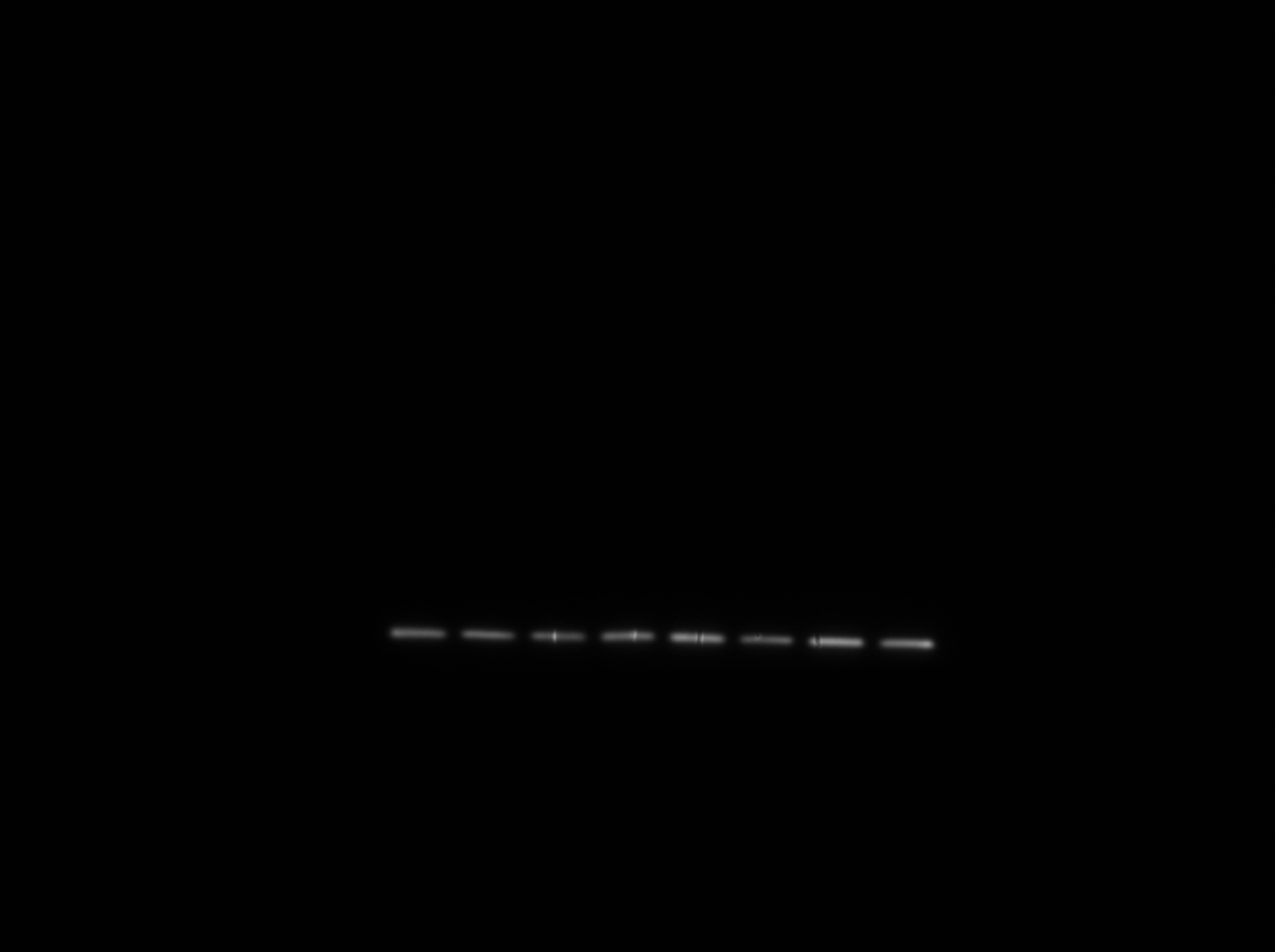

Supplement: Supplementary file 12 [file DataSheet5.ZIP › Figure2/Figure2B/GAPDH Colo205.jpg]

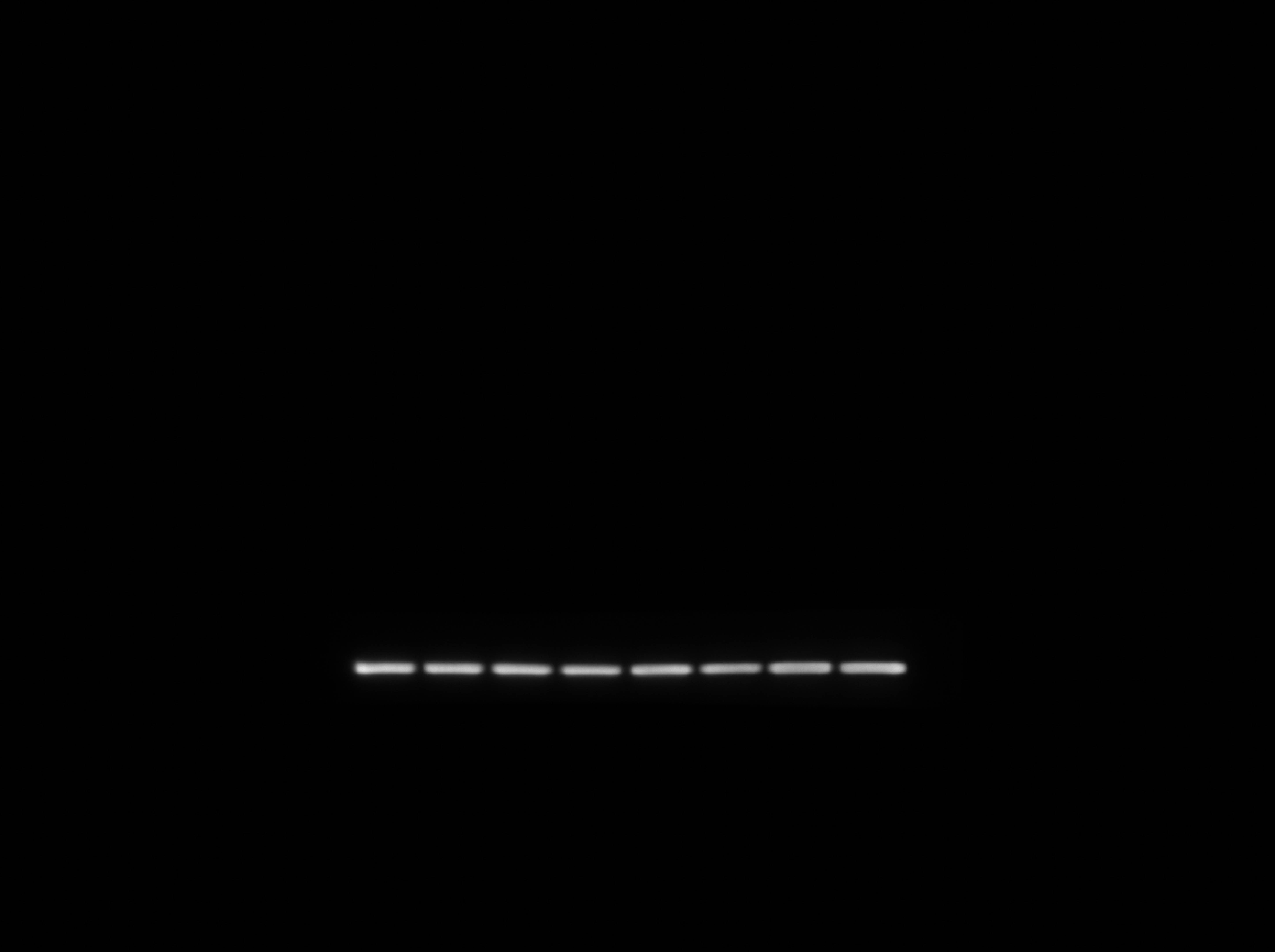

Supplement: Supplementary file 12 [file DataSheet5.ZIP › Figure2/Figure2B/GAPDH RKO.jpg]

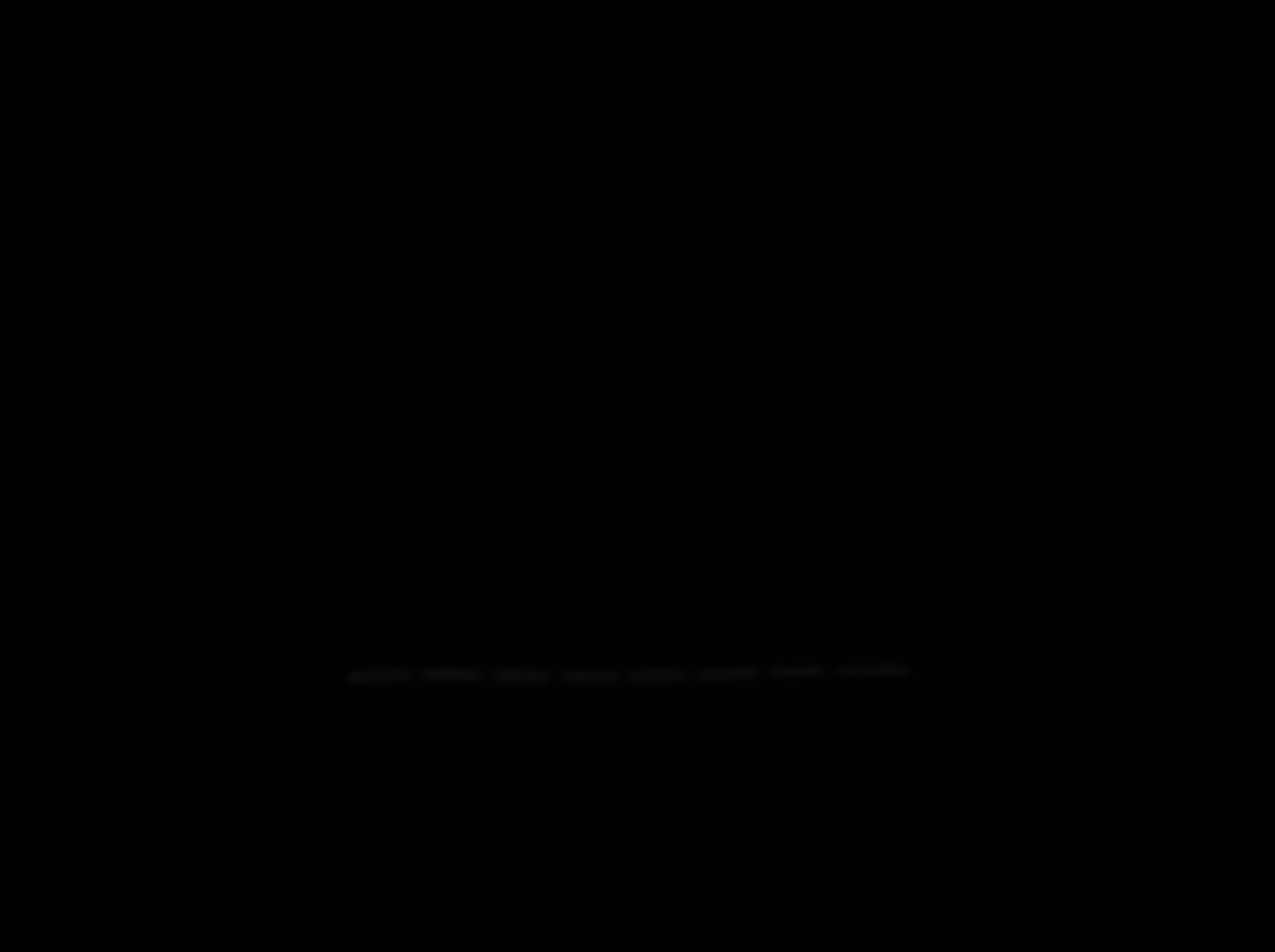

Supplement: Supplementary file 12 [file DataSheet5.ZIP › Figure2/Figure2B/GAPDH SW480.jpg]

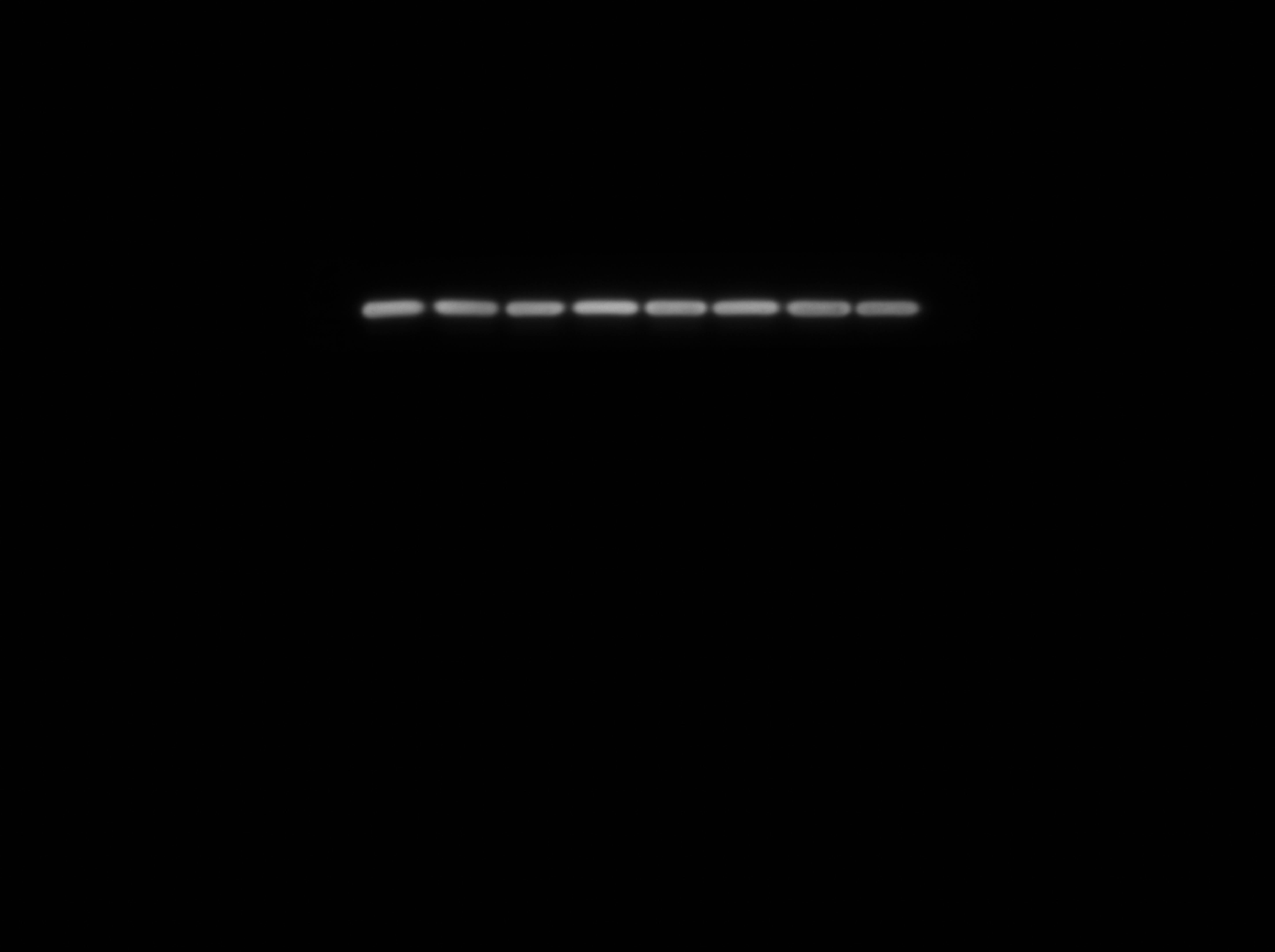

Supplement: Supplementary file 12 [file DataSheet5.ZIP › Figure2/Figure2B/GAPDH SW620.jpg]

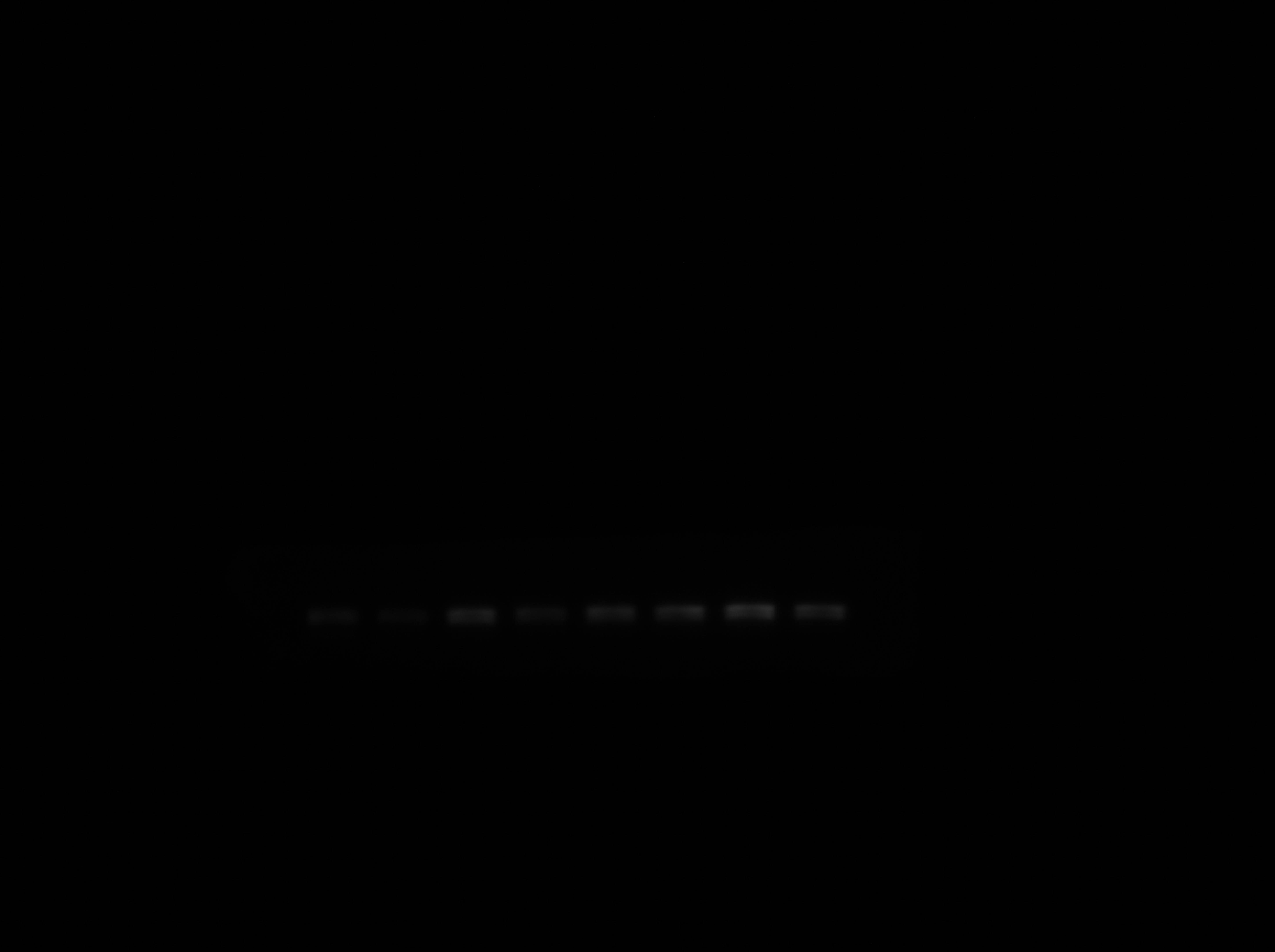

Supplement: Supplementary file 12 [file DataSheet5.ZIP › Figure2/Figure2B/P-AKT CW-2.jpg]

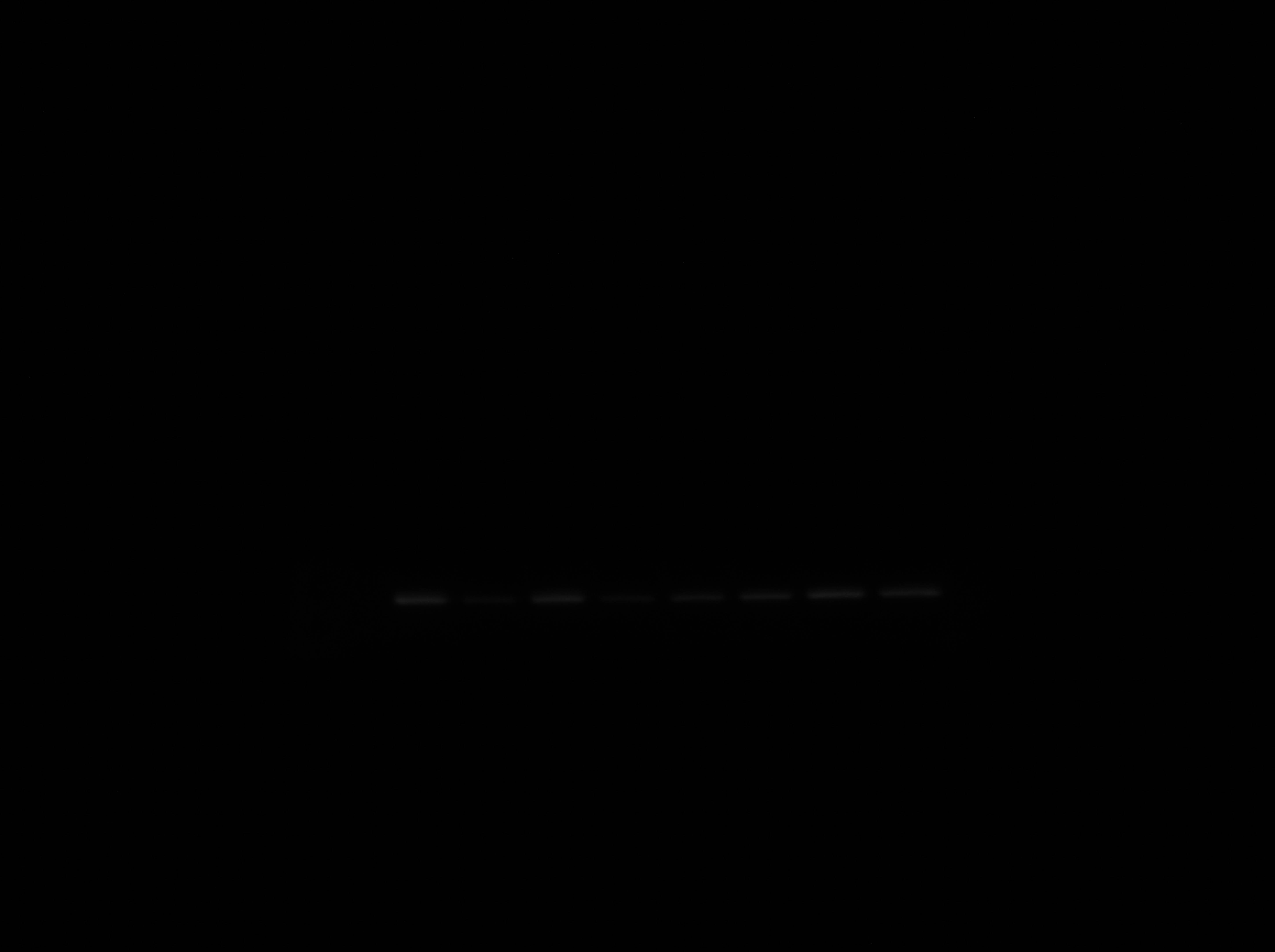

Supplement: Supplementary file 12 [file DataSheet5.ZIP › Figure2/Figure2B/P-AKT Caco-2.jpg]

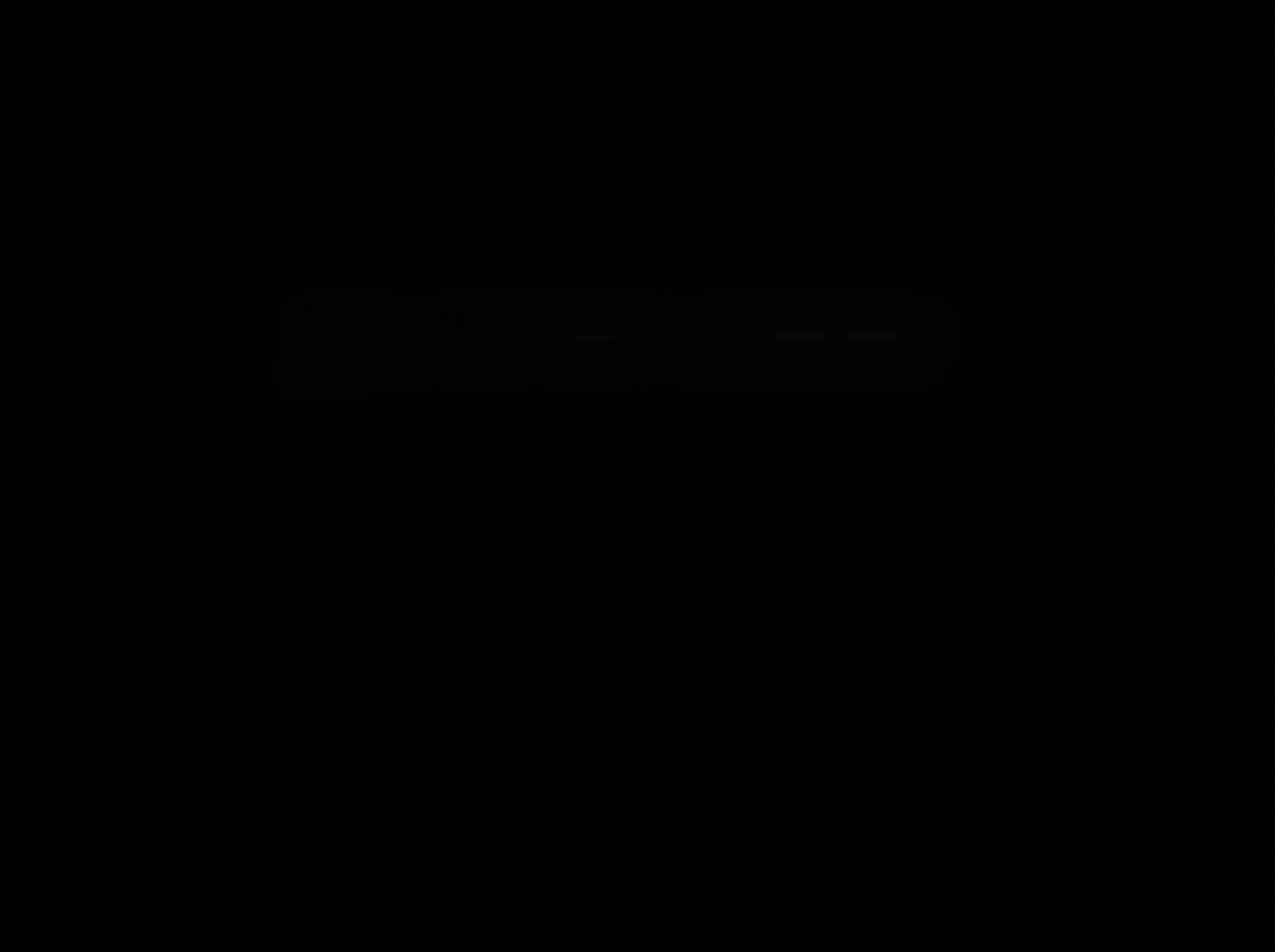

Supplement: Supplementary file 12 [file DataSheet5.ZIP › Figure2/Figure2B/P-AKT Colo205.jpg]

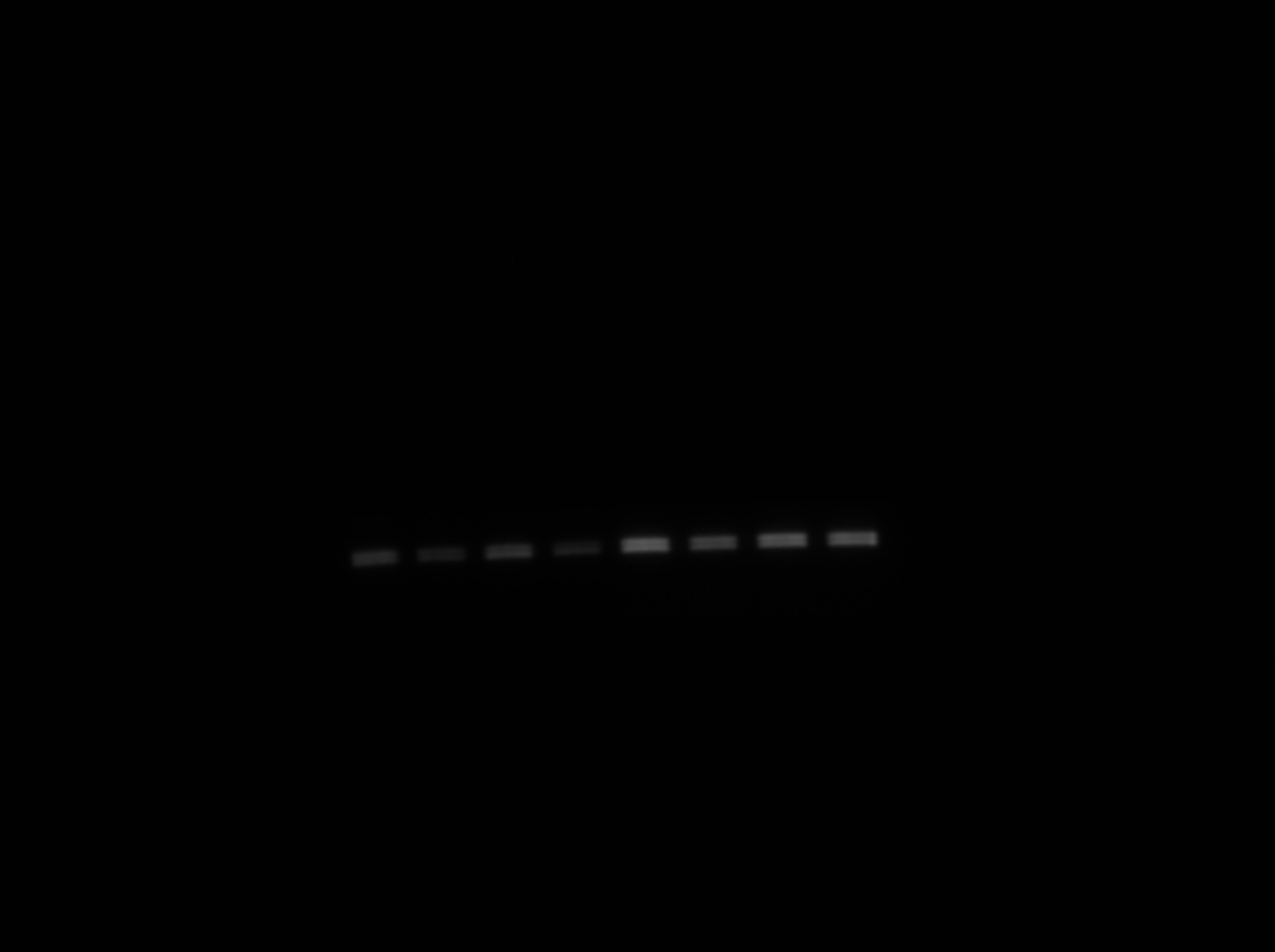

Supplement: Supplementary file 12 [file DataSheet5.ZIP › Figure2/Figure2B/P-AKT RKO.jpg]

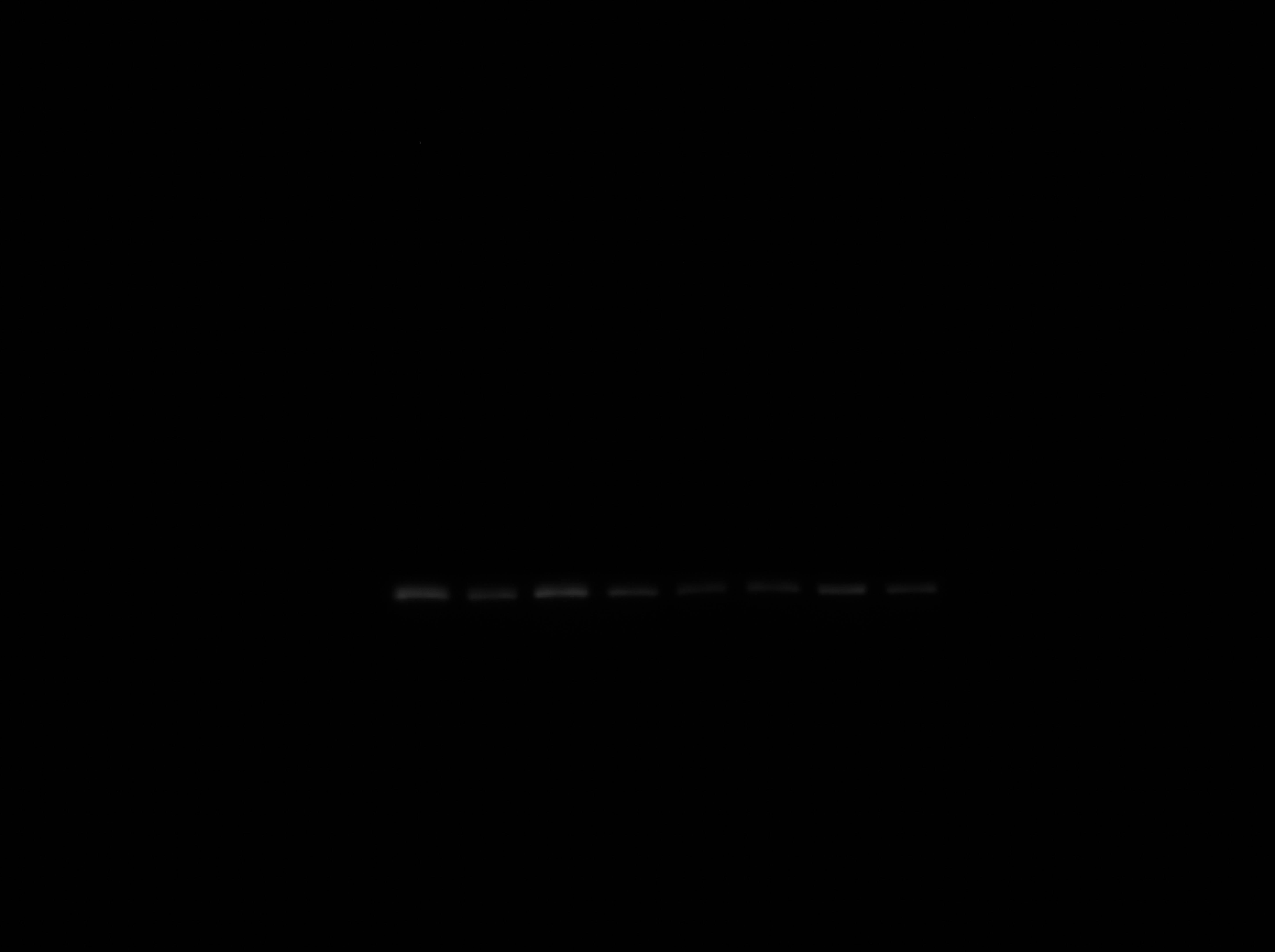

Supplement: Supplementary file 12 [file DataSheet5.ZIP › Figure2/Figure2B/P-AKT SW480.jpg]

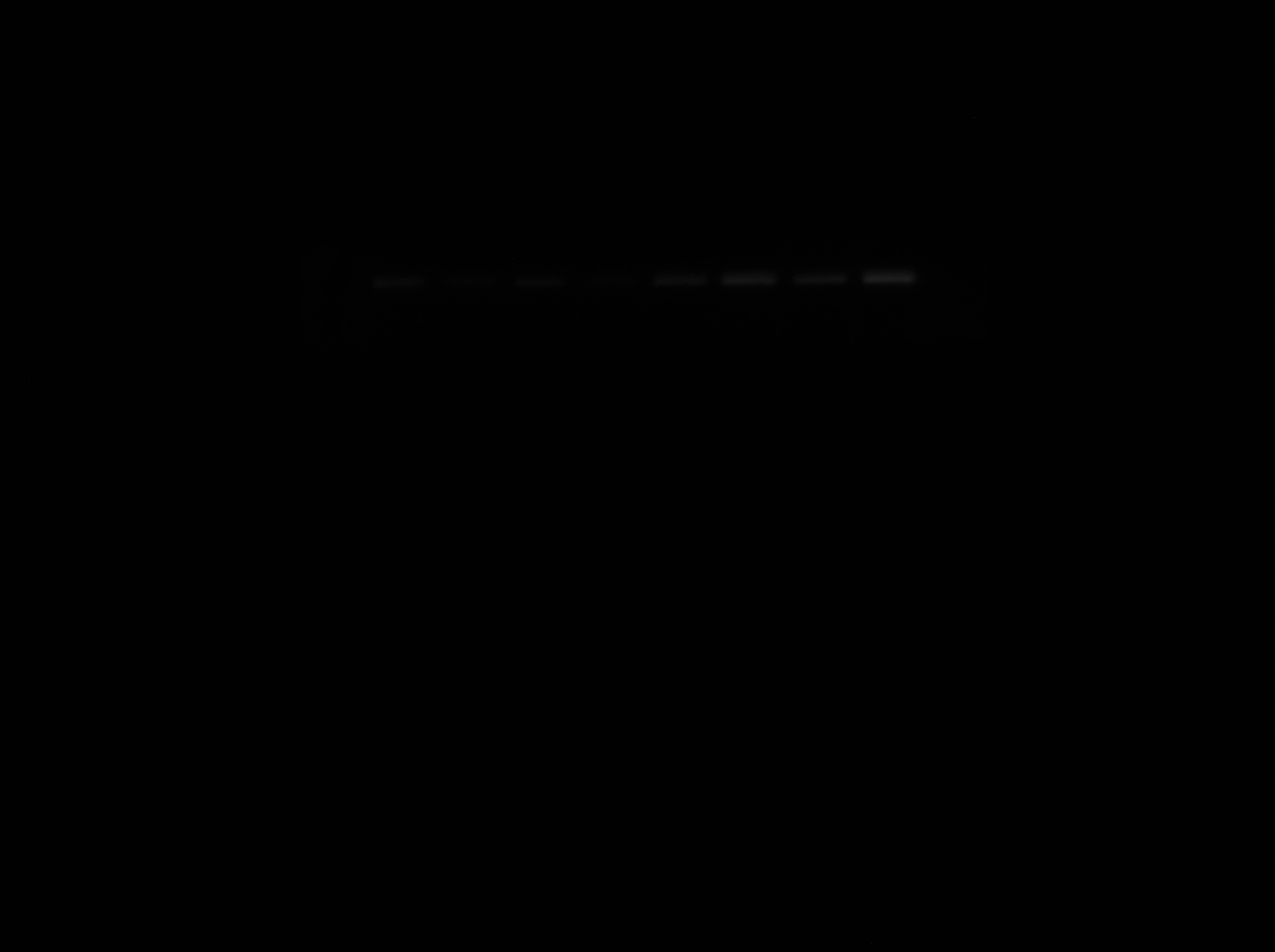

Supplement: Supplementary file 12 [file DataSheet5.ZIP › Figure2/Figure2B/P-AKT SW620.jpg]

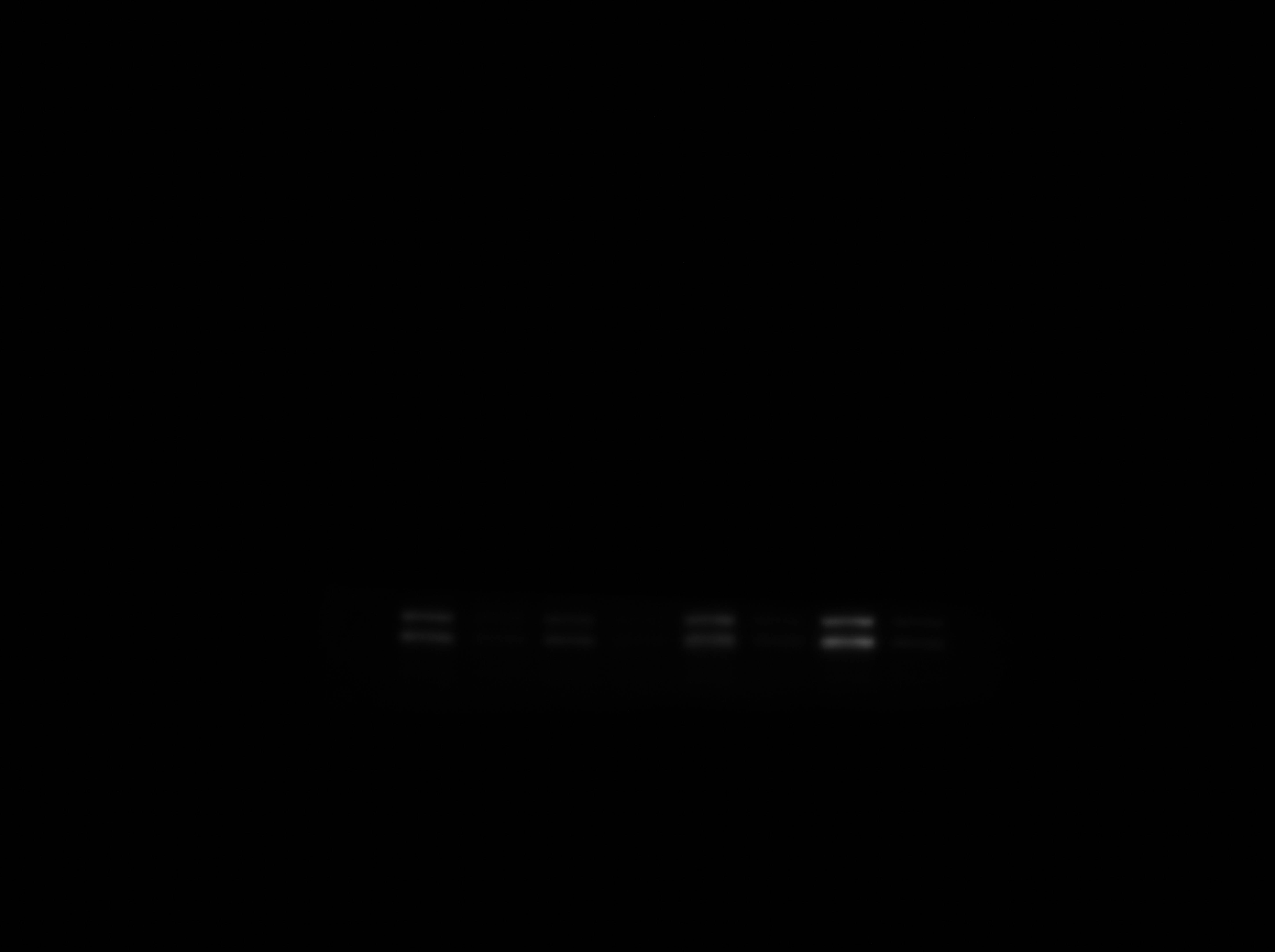

Supplement: Supplementary file 12 [file DataSheet5.ZIP › Figure2/Figure2B/P-ERK CW-2.jpg]

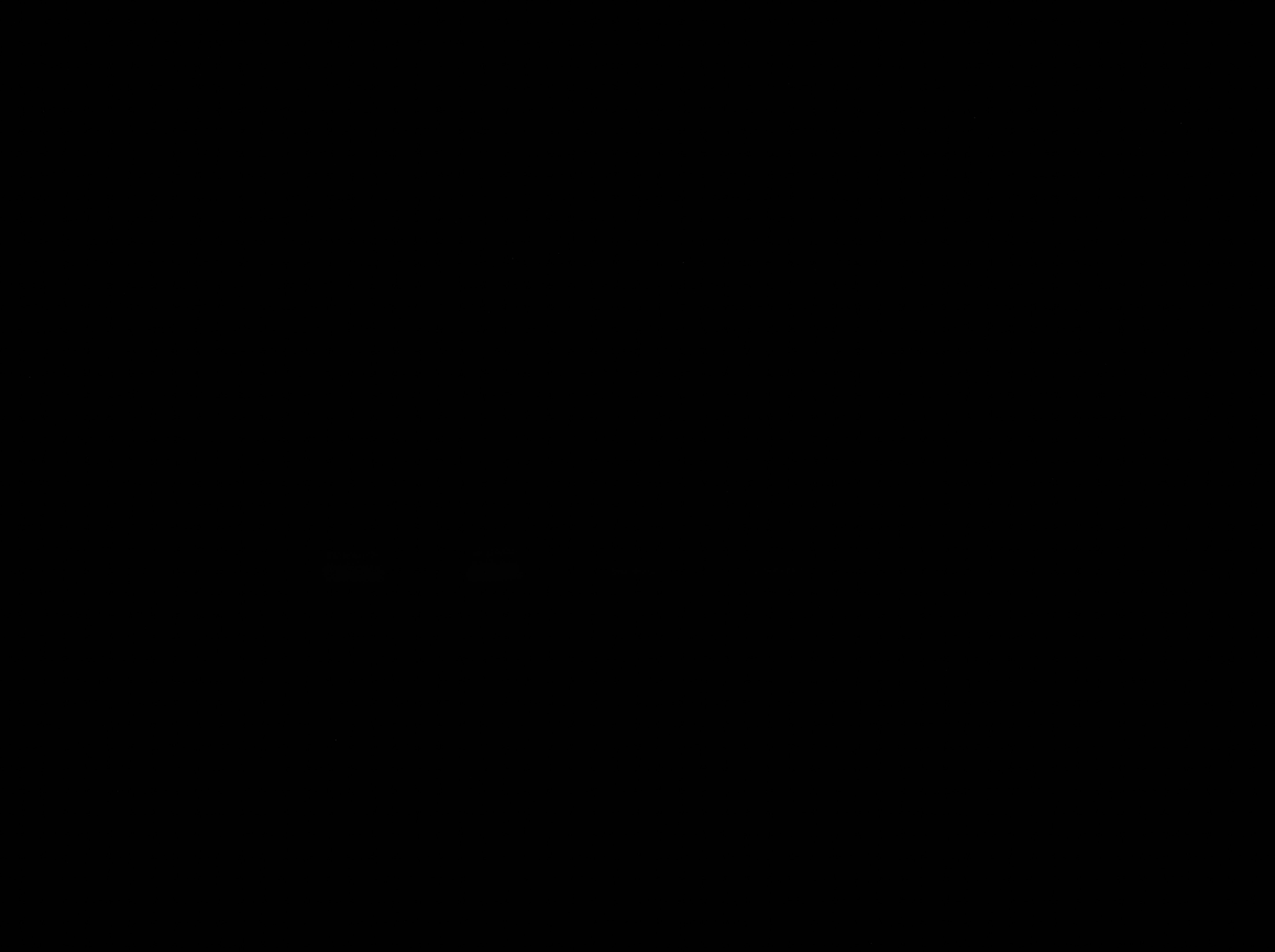

Supplement: Supplementary file 12 [file DataSheet5.ZIP › Figure2/Figure2B/P-ERK Caco-2.jpg]

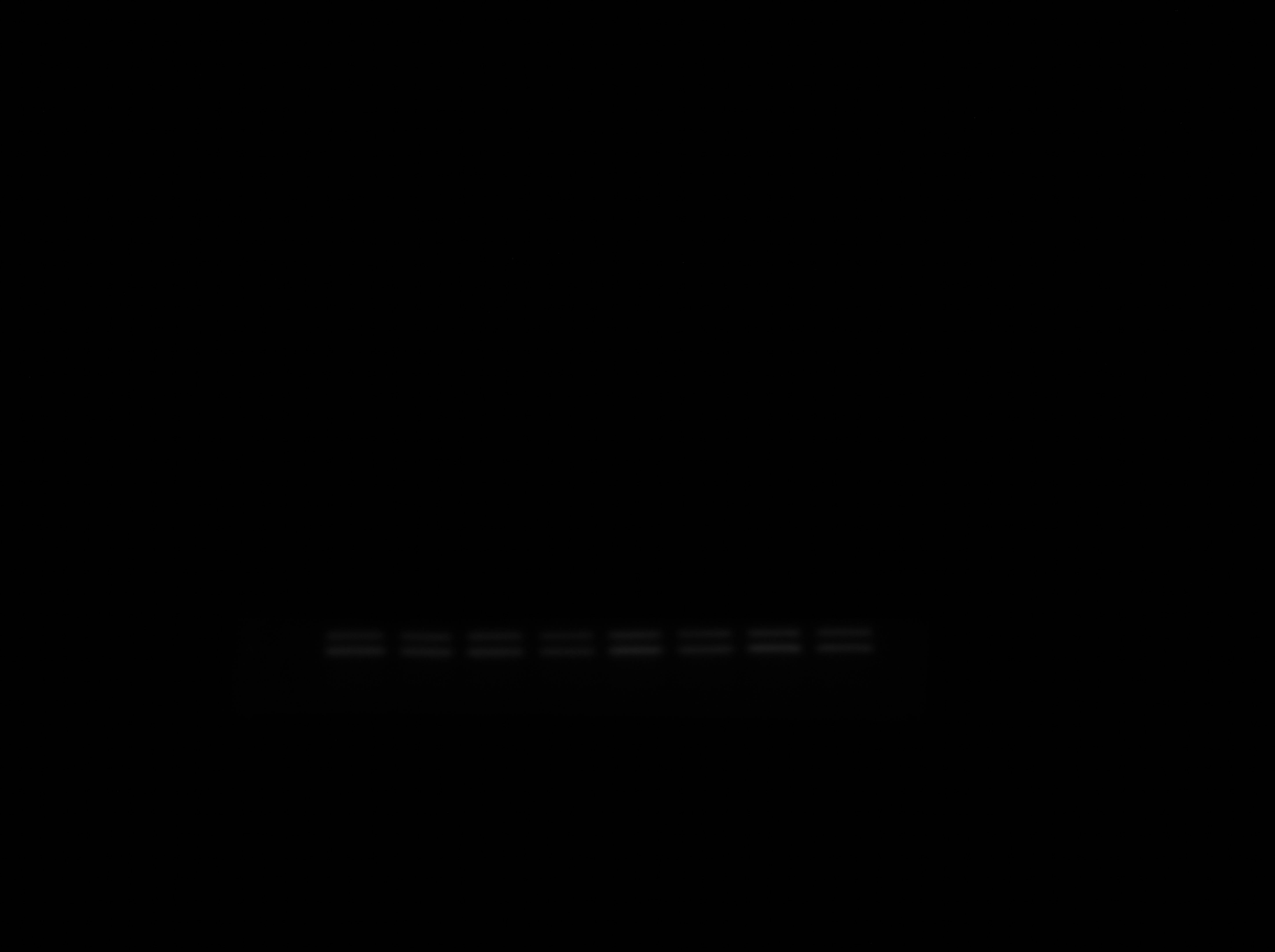

Supplement: Supplementary file 12 [file DataSheet5.ZIP › Figure2/Figure2B/P-ERK Colo205.jpg]

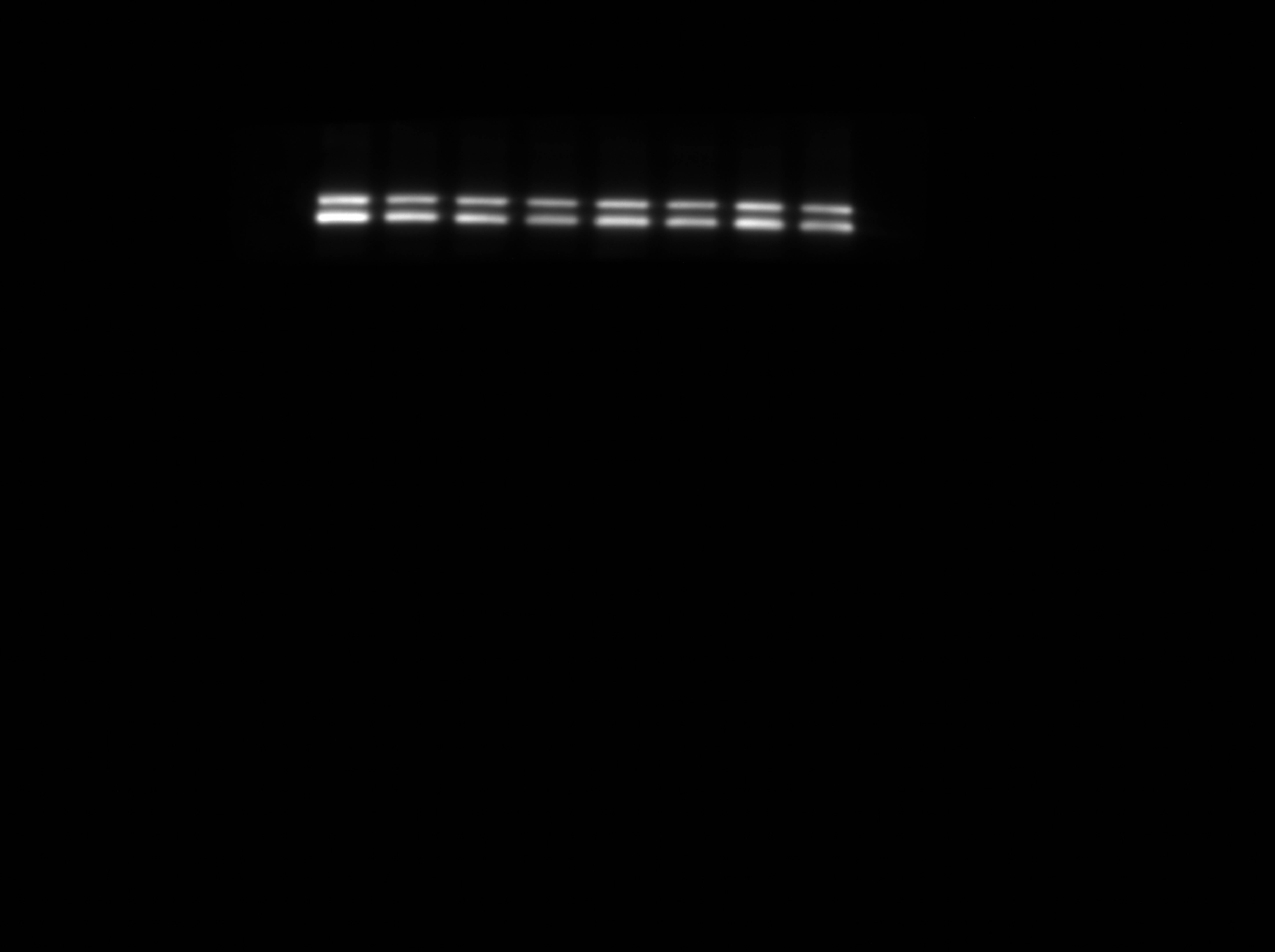

Supplement: Supplementary file 12 [file DataSheet5.ZIP › Figure2/Figure2B/P-ERK RKO.jpg]

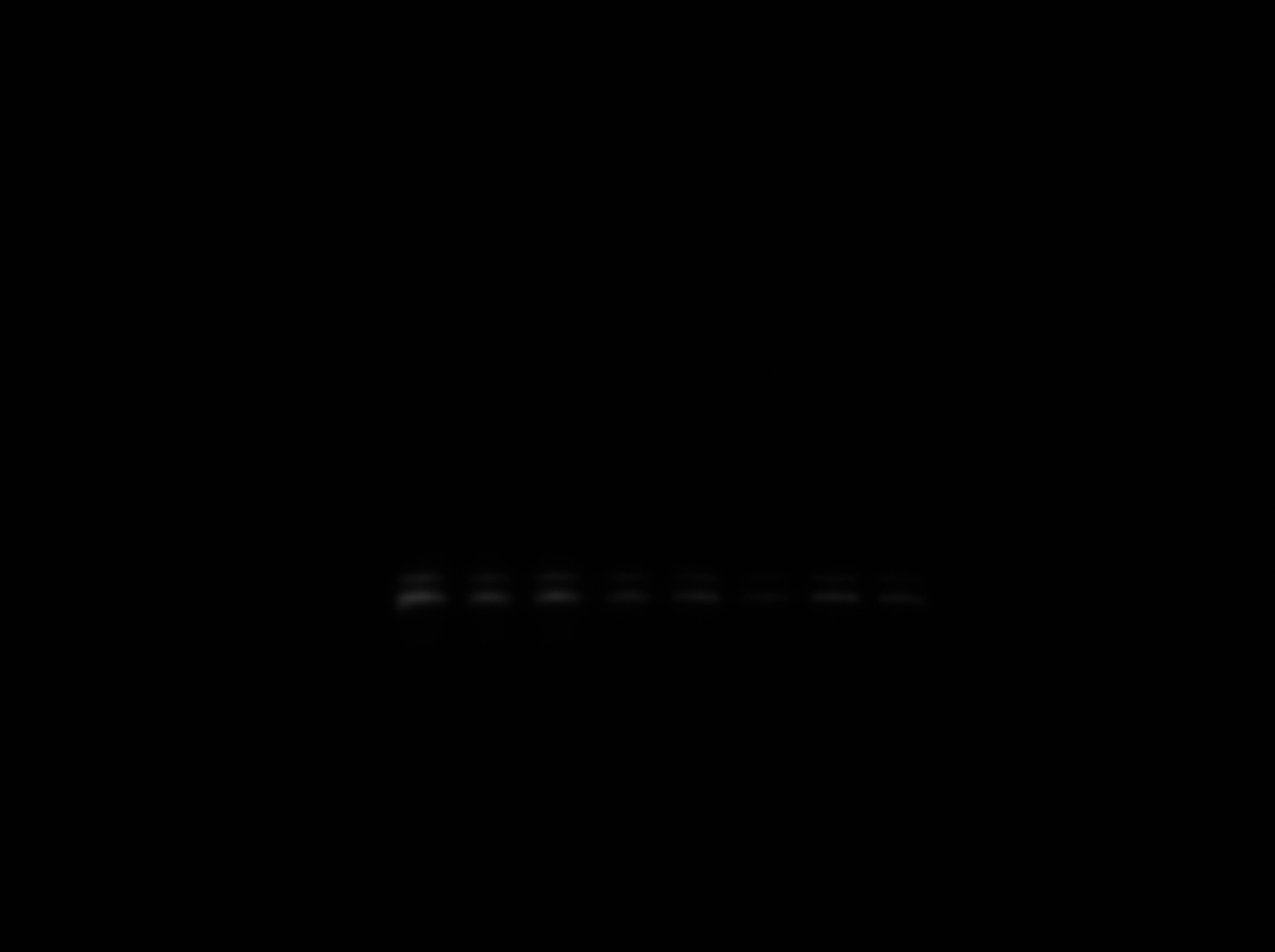

Supplement: Supplementary file 12 [file DataSheet5.ZIP › Figure2/Figure2B/P-ERK SW480.jpg]

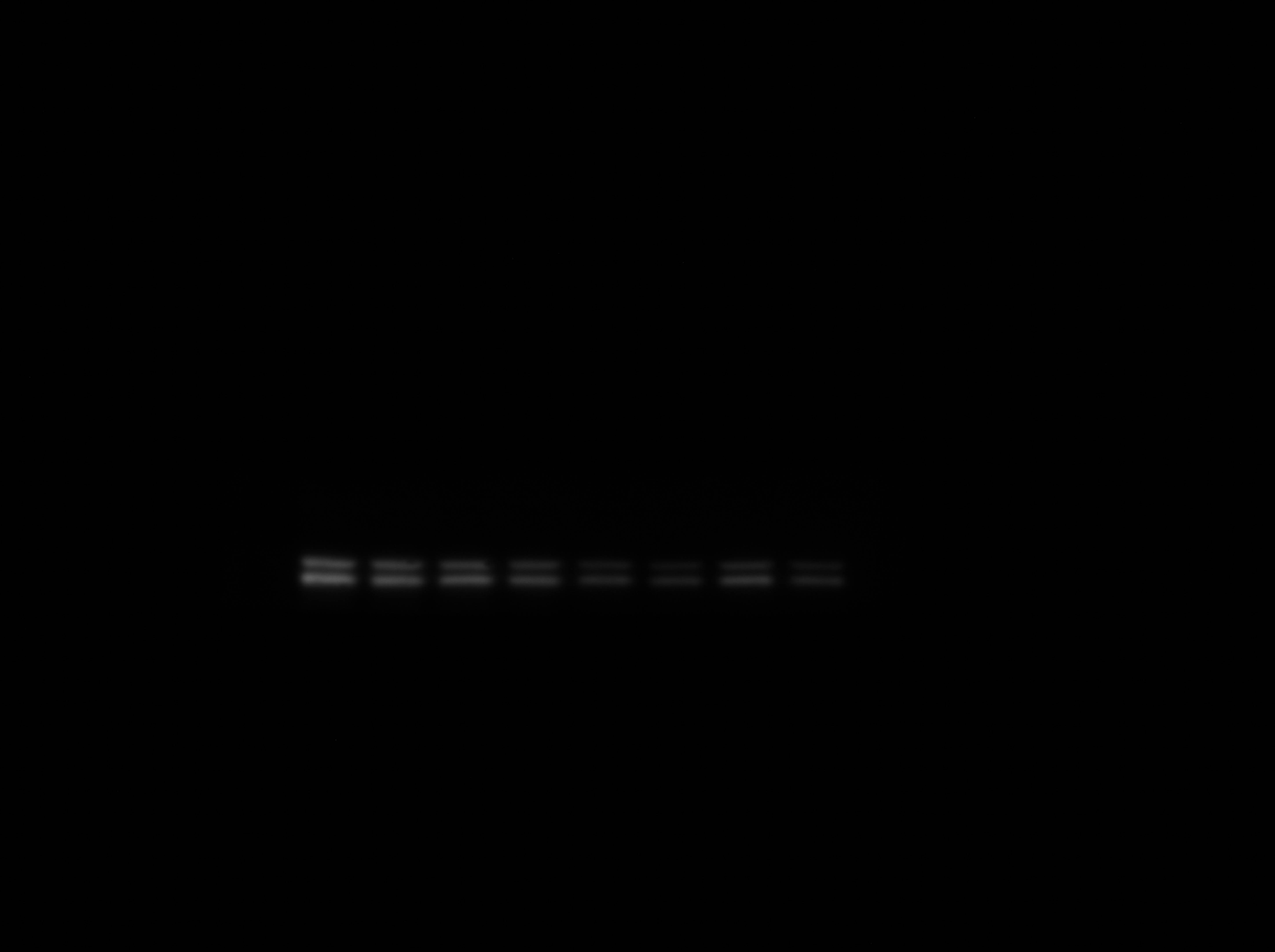

Supplement: Supplementary file 12 [file DataSheet5.ZIP › Figure2/Figure2B/P-ERK SW620.jpg]

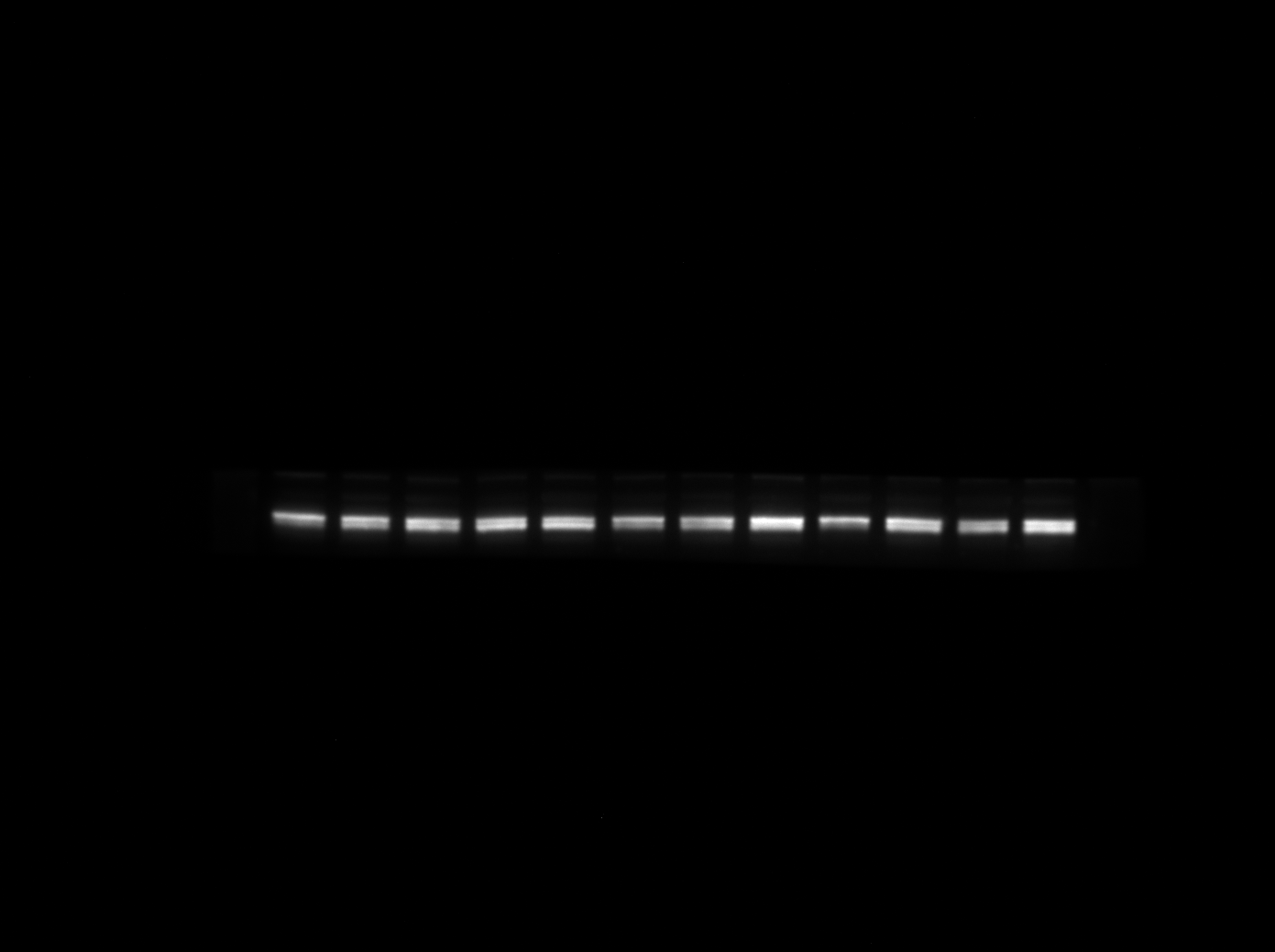

Supplement: Supplementary file 12 [file DataSheet5.ZIP › Figure2/Figure2D/COLO205/AKT - instruction.tif]

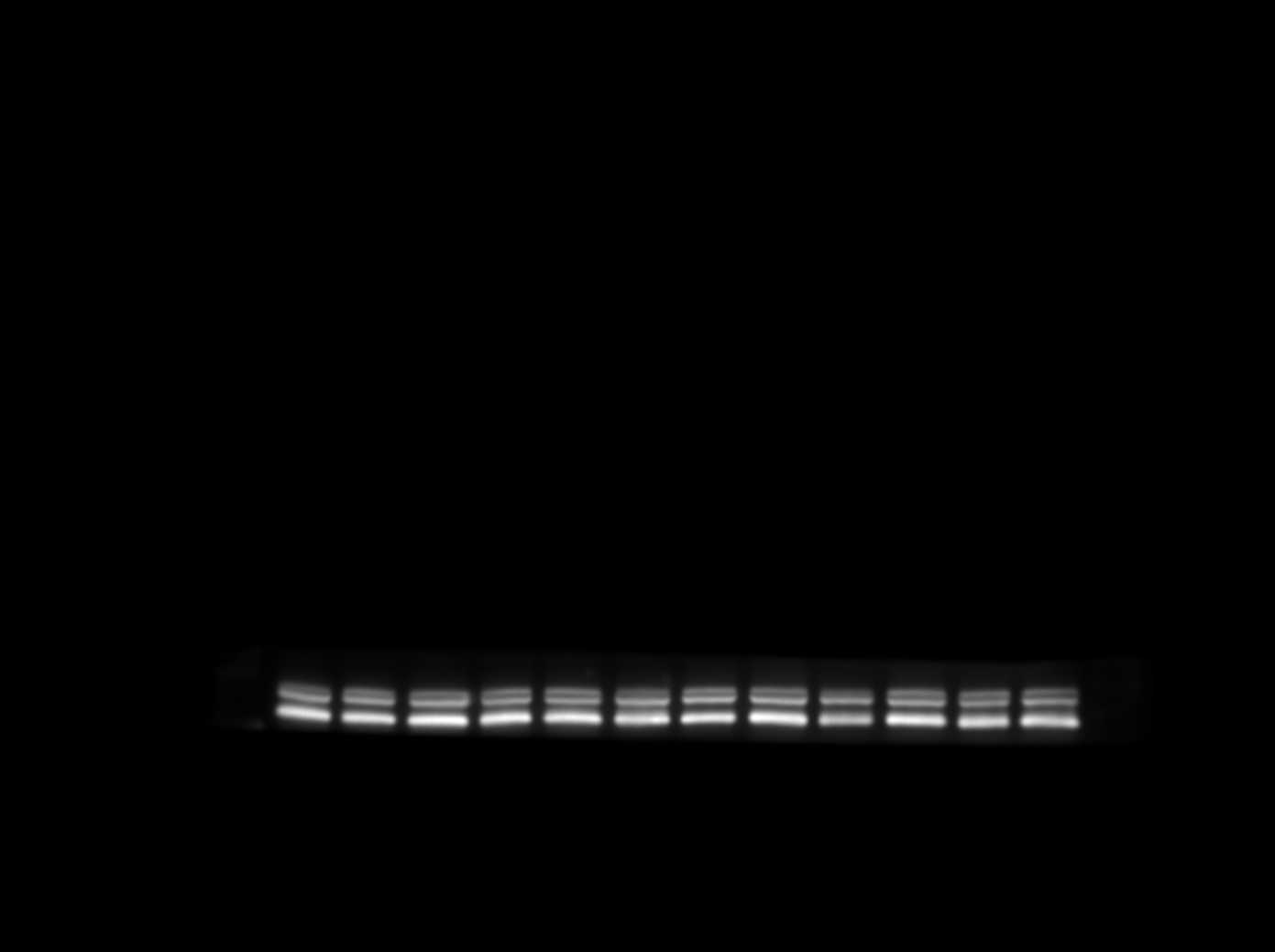

Supplement: Supplementary file 12 [file DataSheet5.ZIP › Figure2/Figure2D/COLO205/Erk.tif]

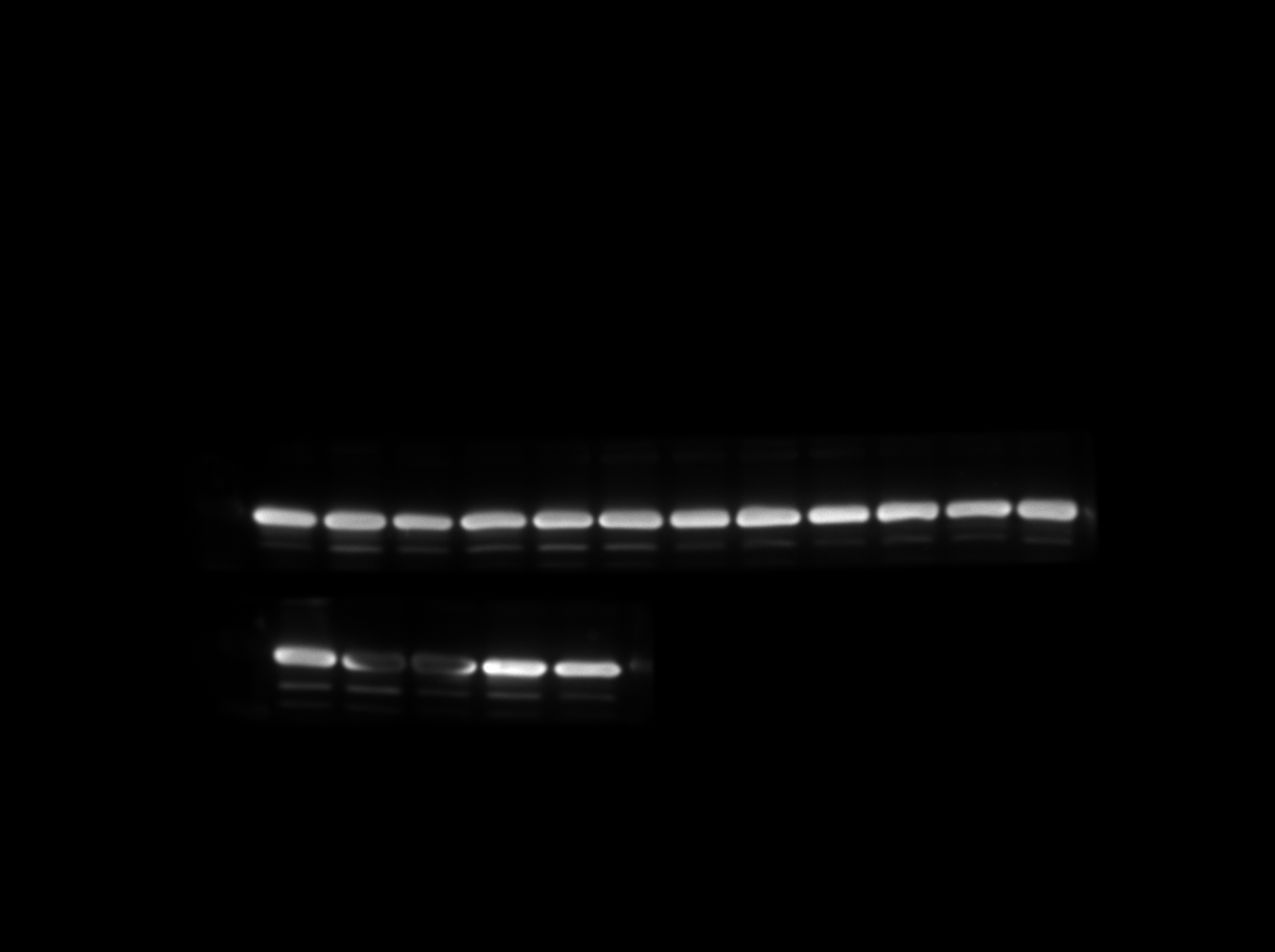

Supplement: Supplementary file 12 [file DataSheet5.ZIP › Figure2/Figure2D/COLO205/GAPDH.tif]

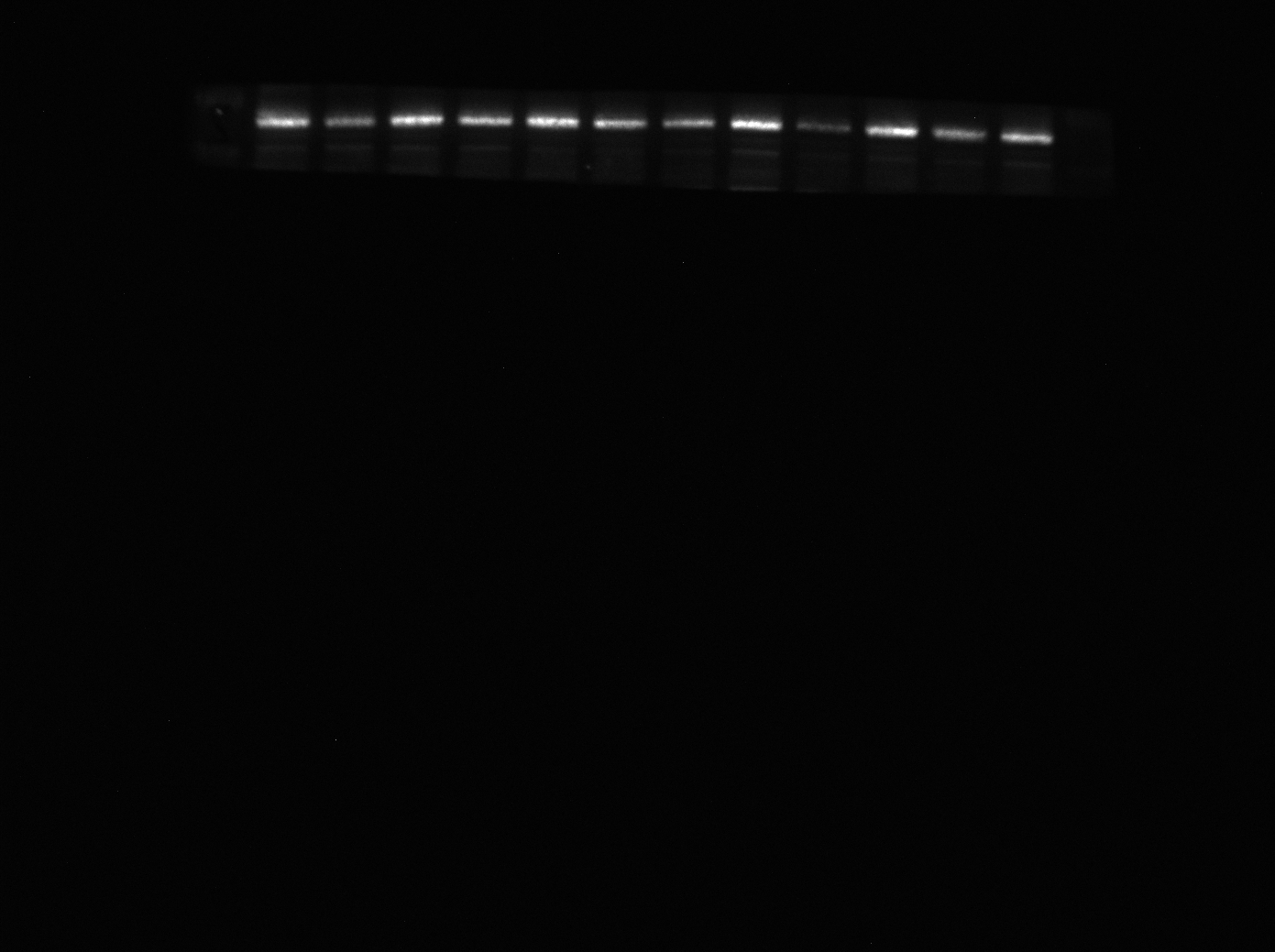

Supplement: Supplementary file 12 [file DataSheet5.ZIP › Figure2/Figure2D/COLO205/SHP2.tif]

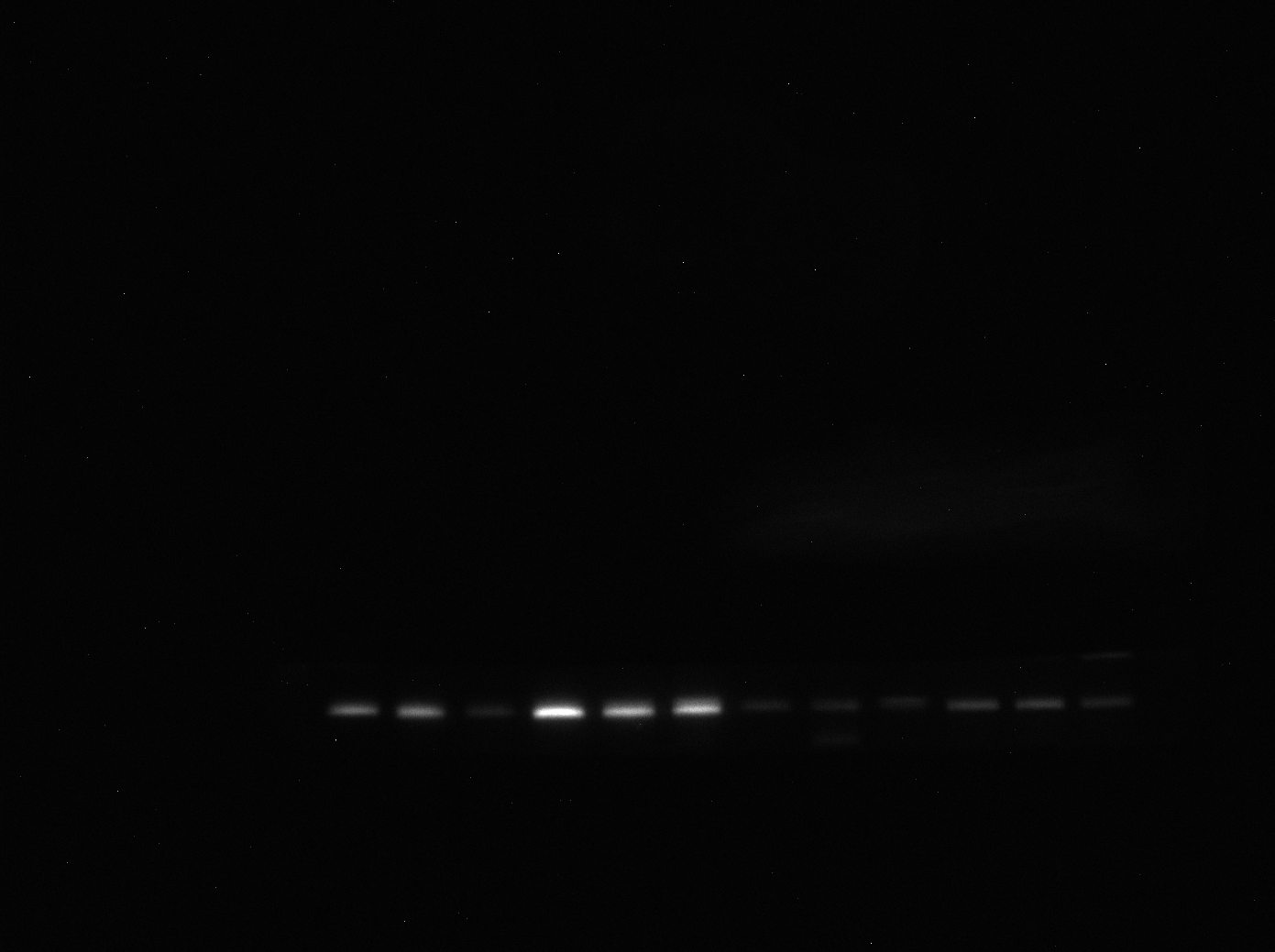

Supplement: Supplementary file 12 [file DataSheet5.ZIP › Figure2/Figure2D/COLO205/p-AKT.tif]

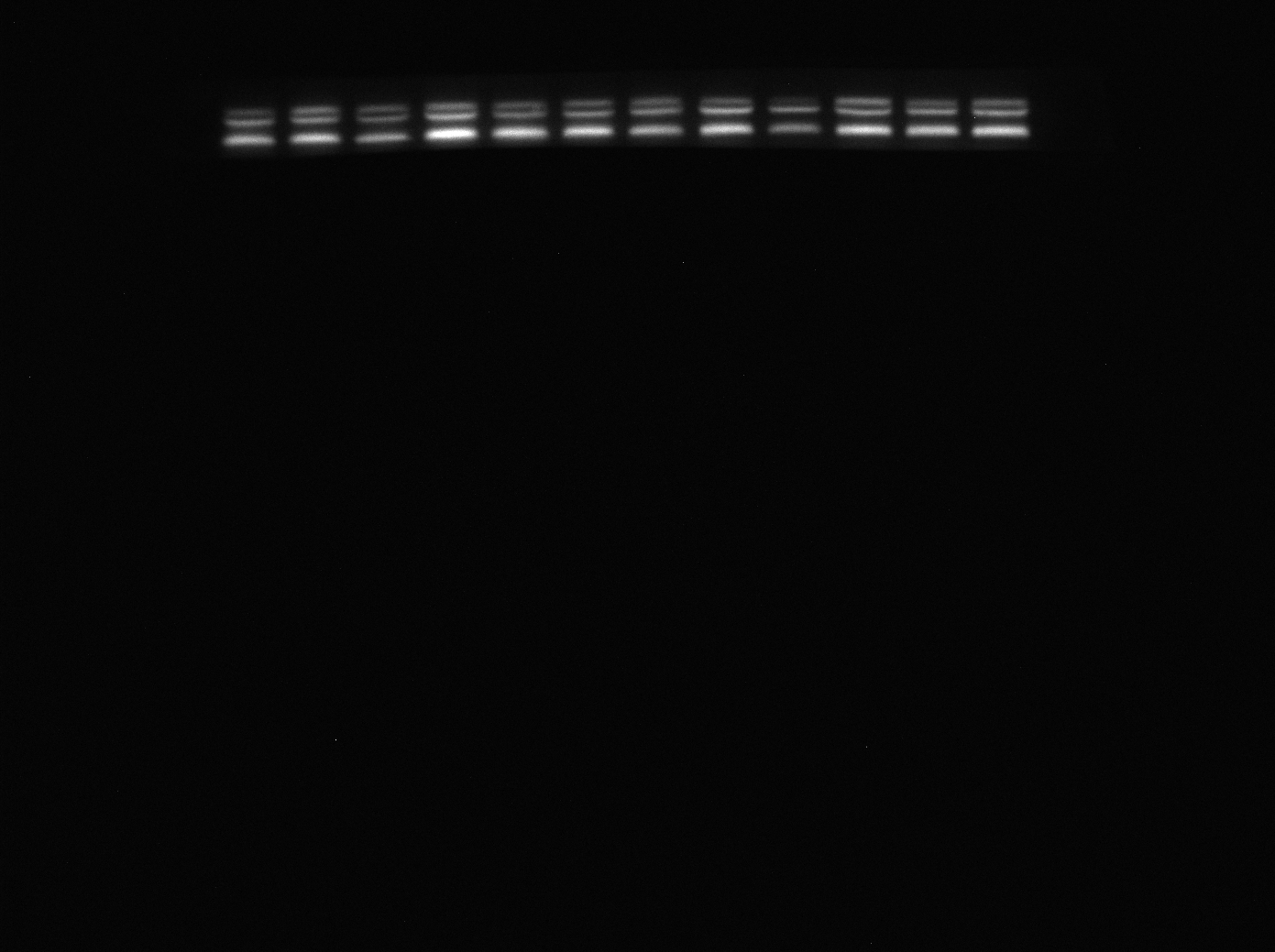

Supplement: Supplementary file 12 [file DataSheet5.ZIP › Figure2/Figure2D/COLO205/p-Erk.tif]

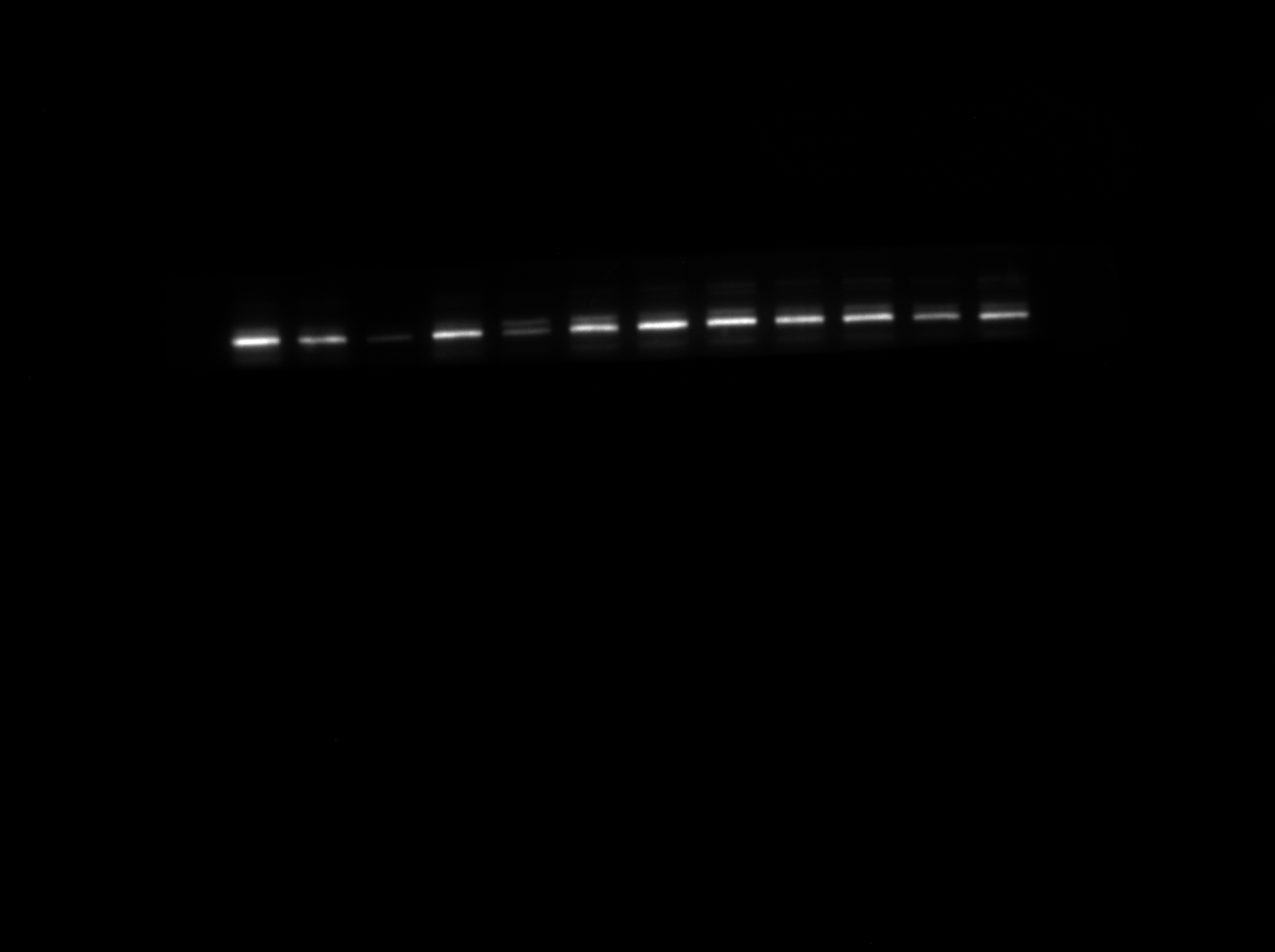

Supplement: Supplementary file 12 [file DataSheet5.ZIP › Figure2/Figure2D/COLO205/p-SHP2.tif]

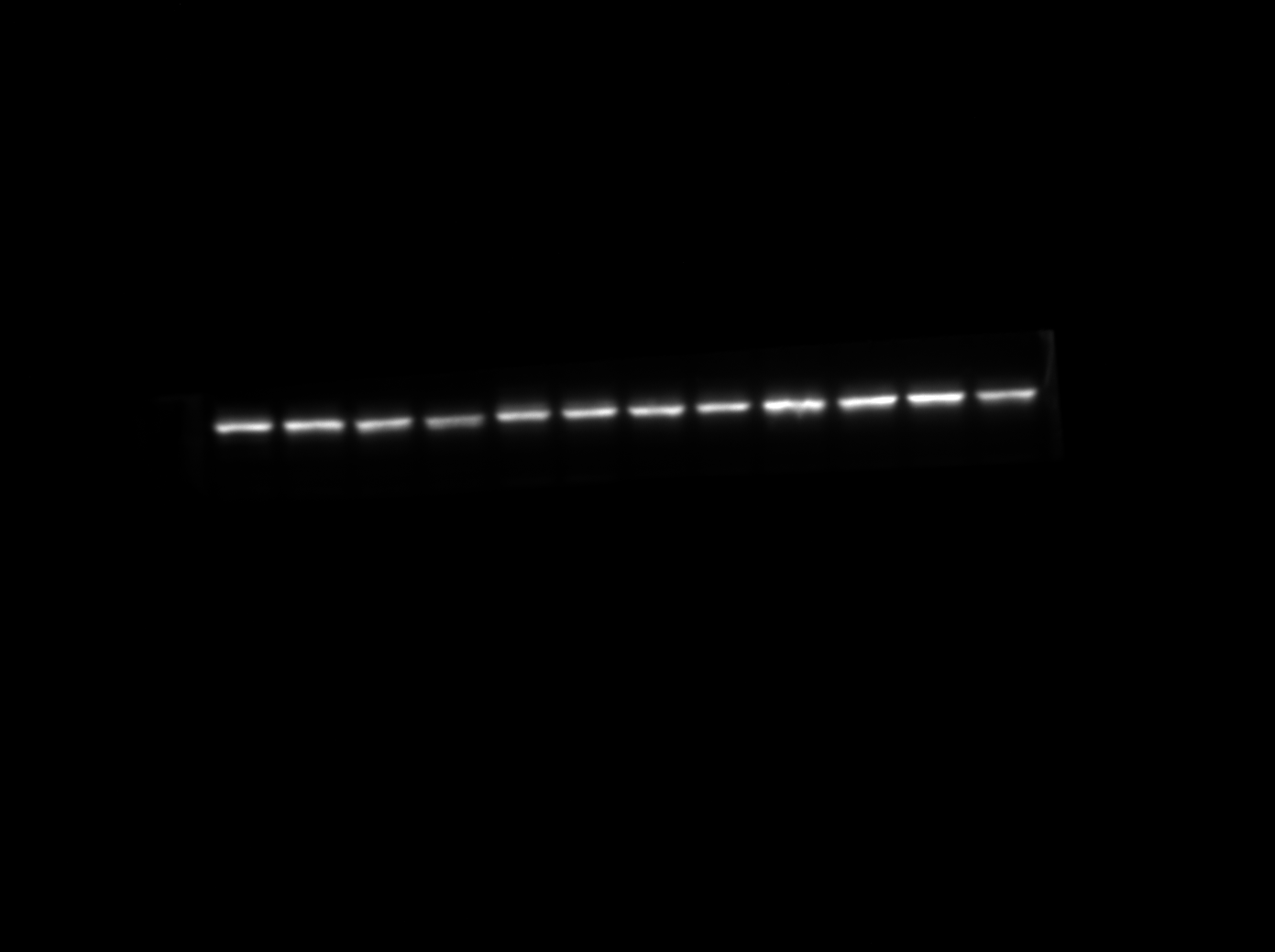

Supplement: Supplementary file 12 [file DataSheet5.ZIP › Figure2/Figure2D/SW620/AKT.tif]

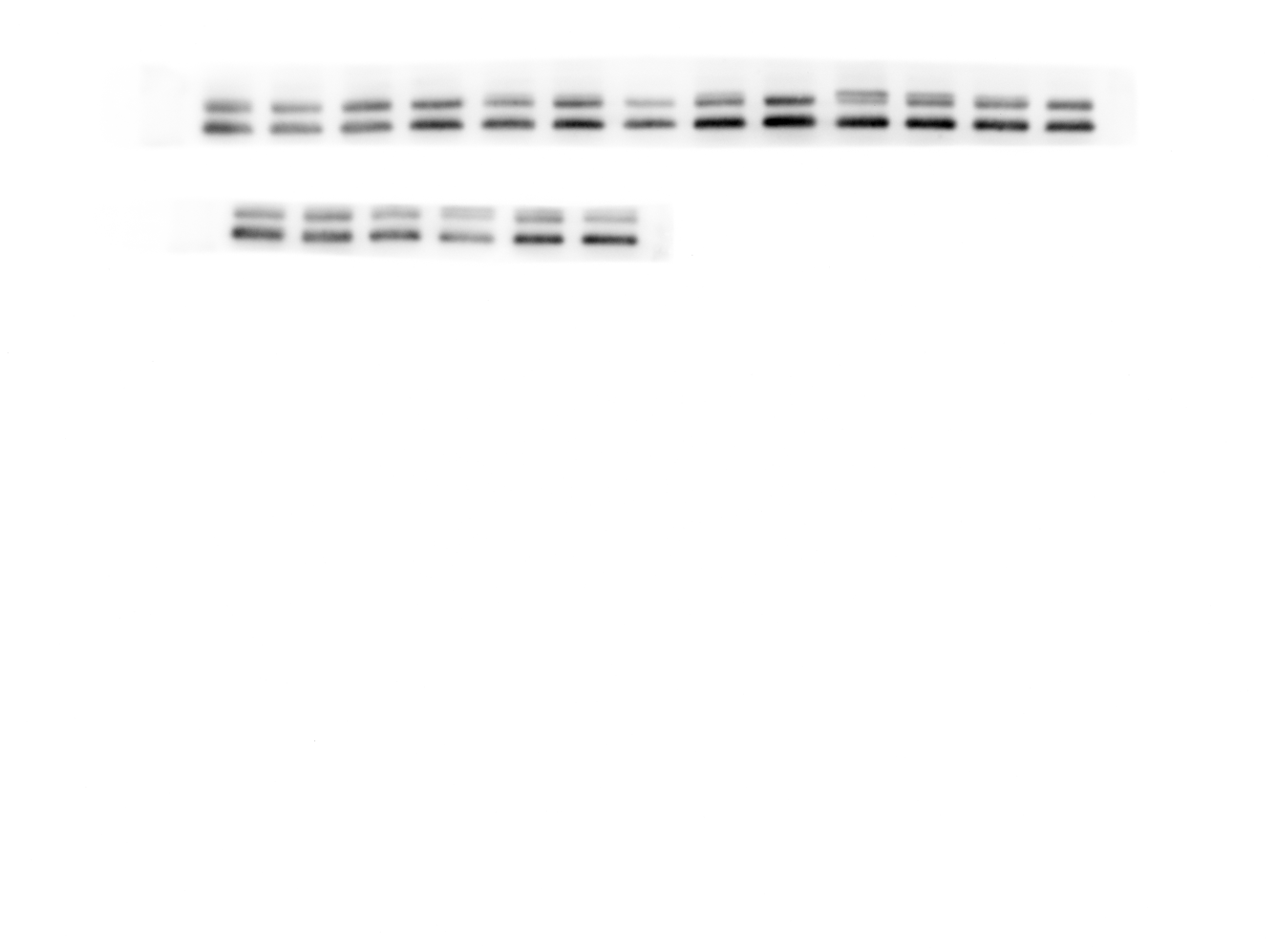

Supplement: Supplementary file 12 [file DataSheet5.ZIP › Figure2/Figure2D/SW620/Erk.tif]

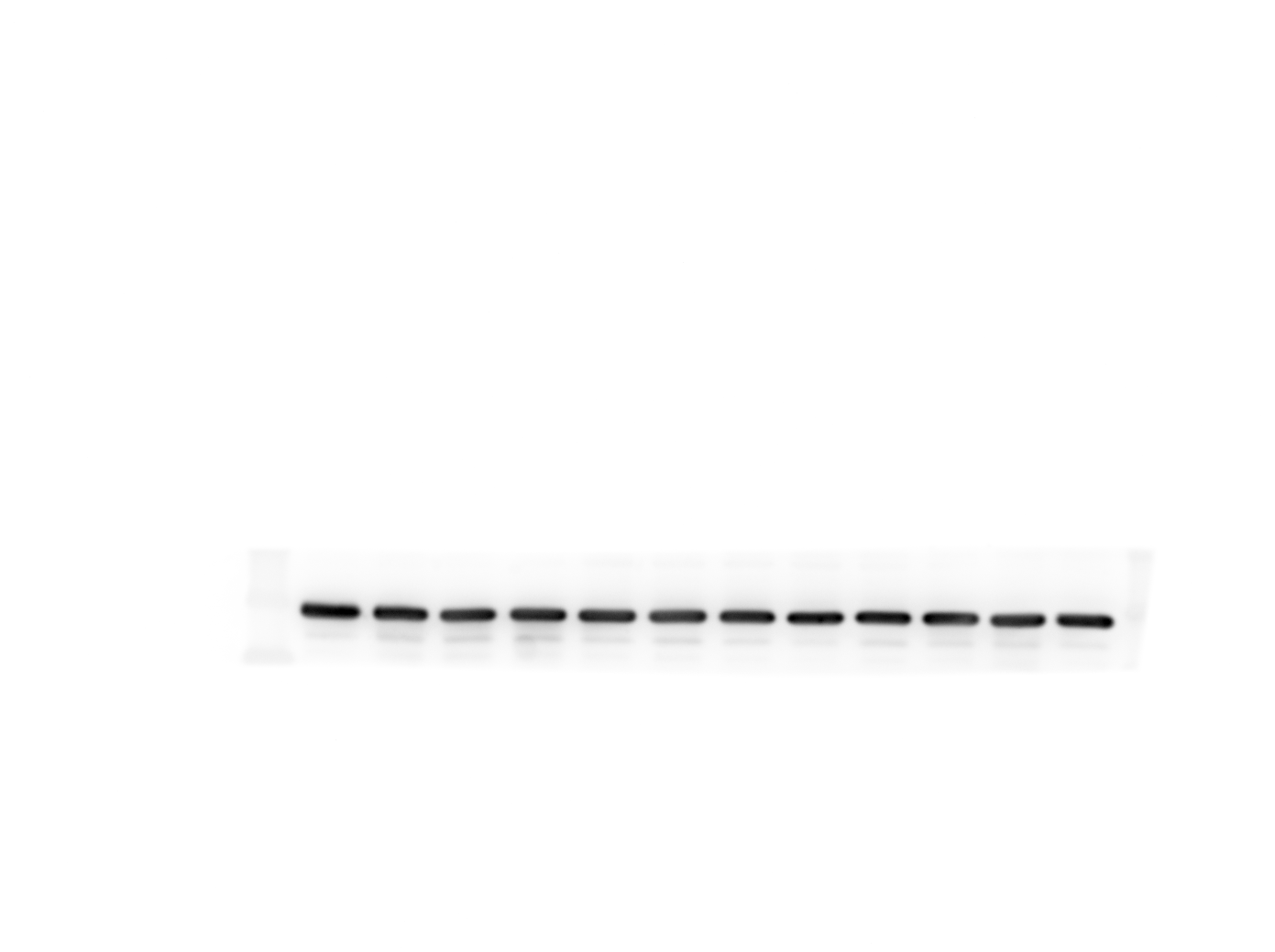

Supplement: Supplementary file 12 [file DataSheet5.ZIP › Figure2/Figure2D/SW620/GAPDH.tif]

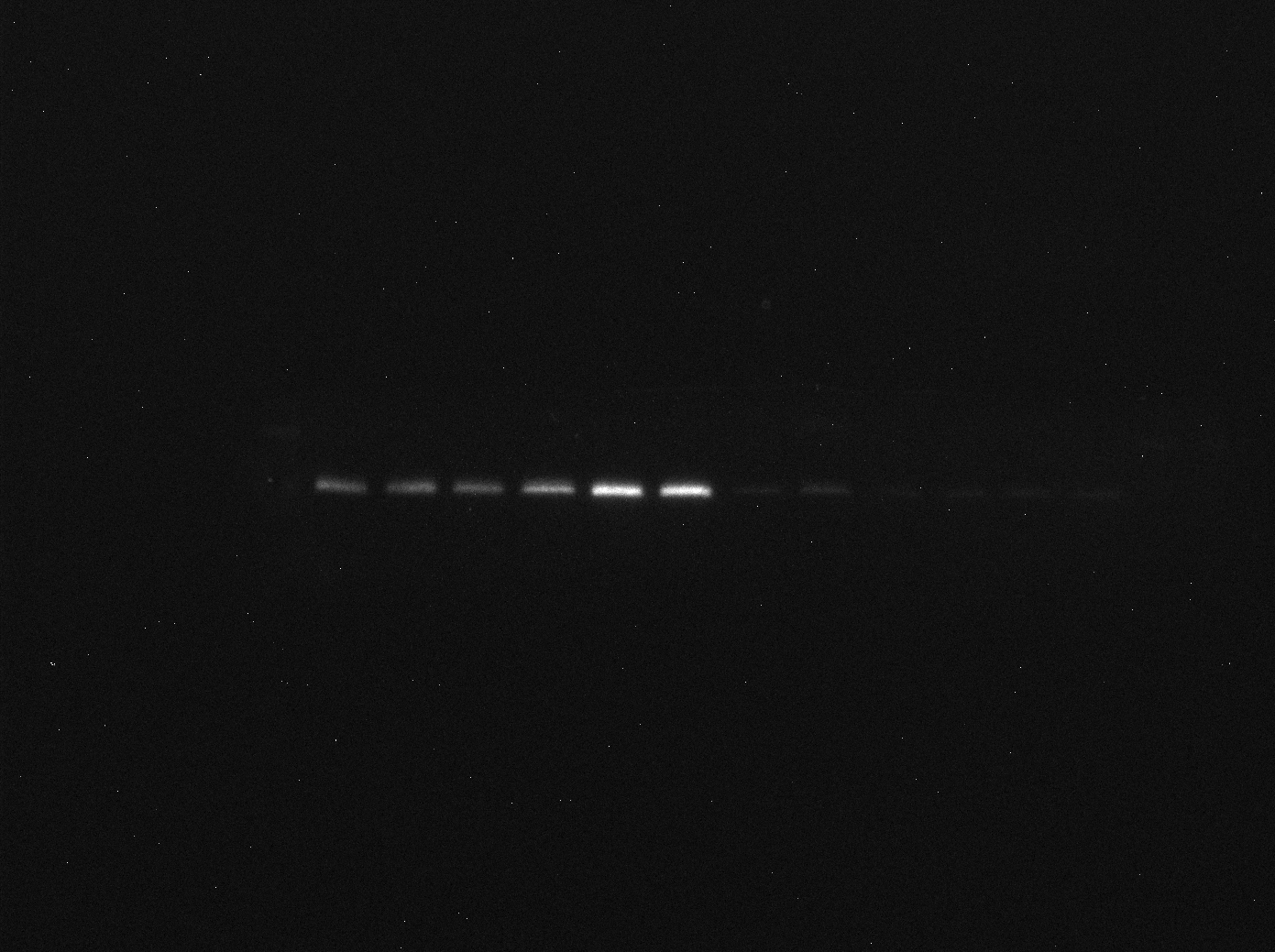

Supplement: Supplementary file 12 [file DataSheet5.ZIP › Figure2/Figure2D/SW620/p-AKT.tif]

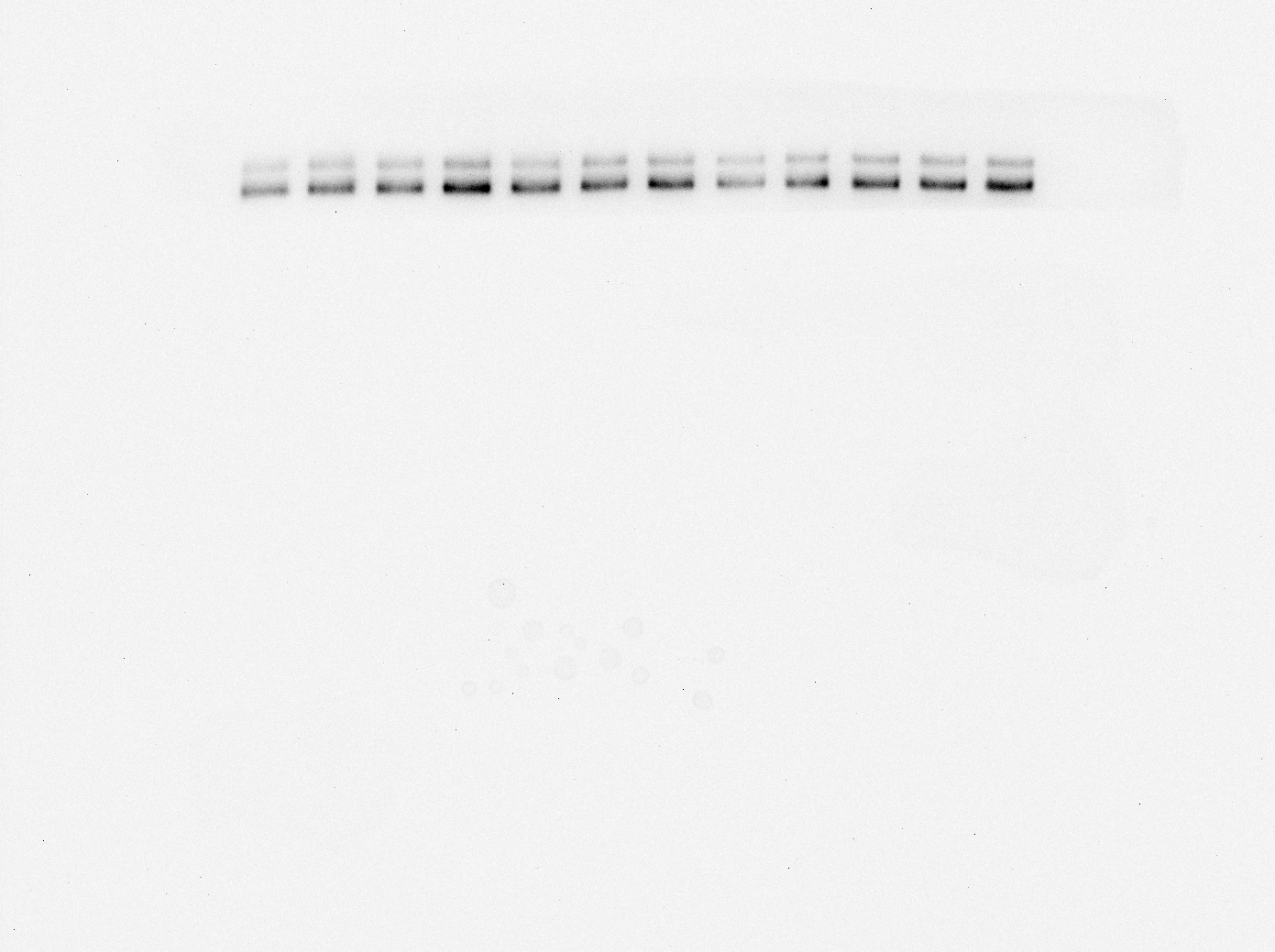

Supplement: Supplementary file 12 [file DataSheet5.ZIP › Figure2/Figure2D/SW620/p-Erk.tif]

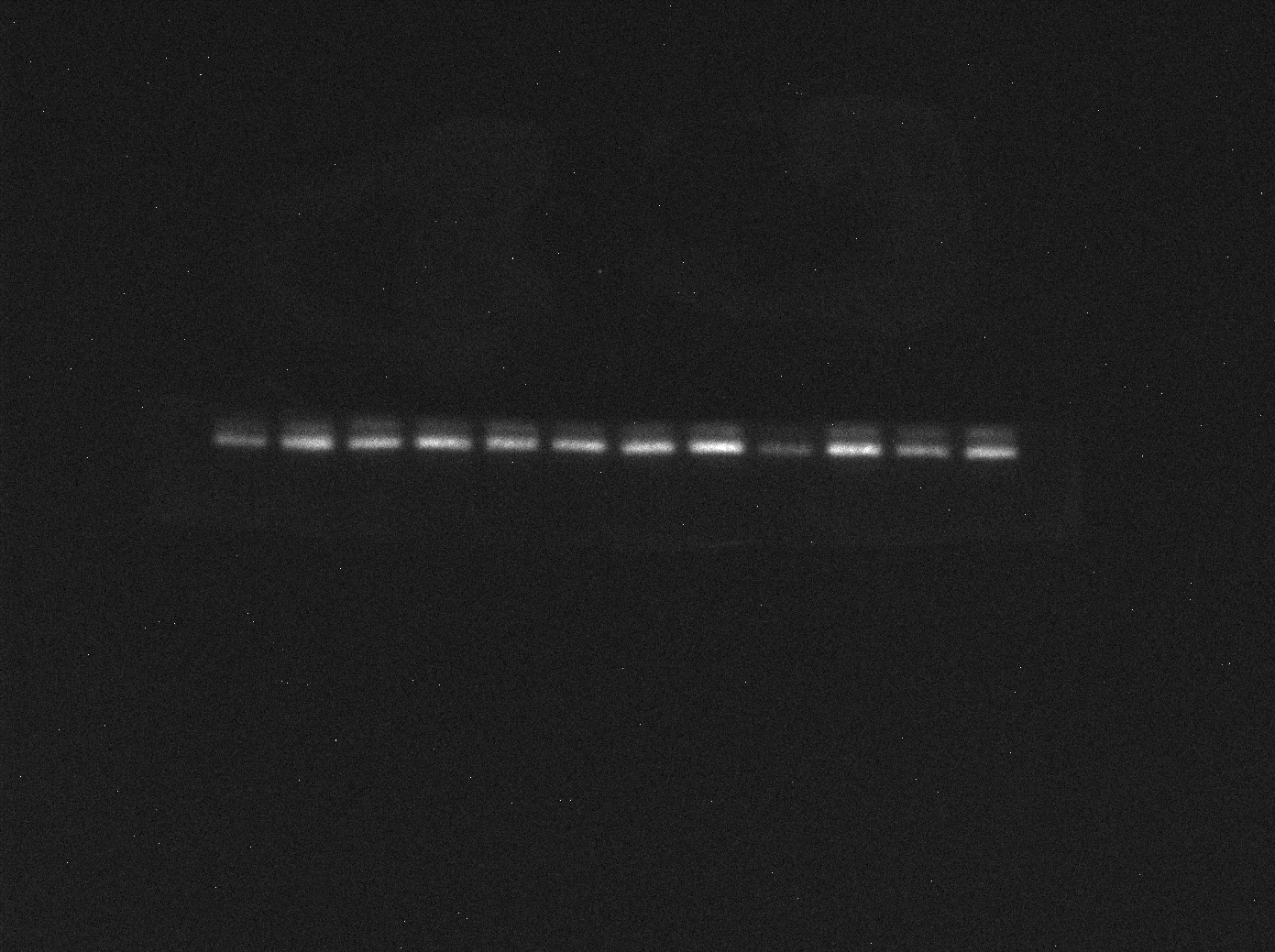

Supplement: Supplementary file 12 [file DataSheet5.ZIP › Figure2/Figure2D/SW620/p-shp2.tif]

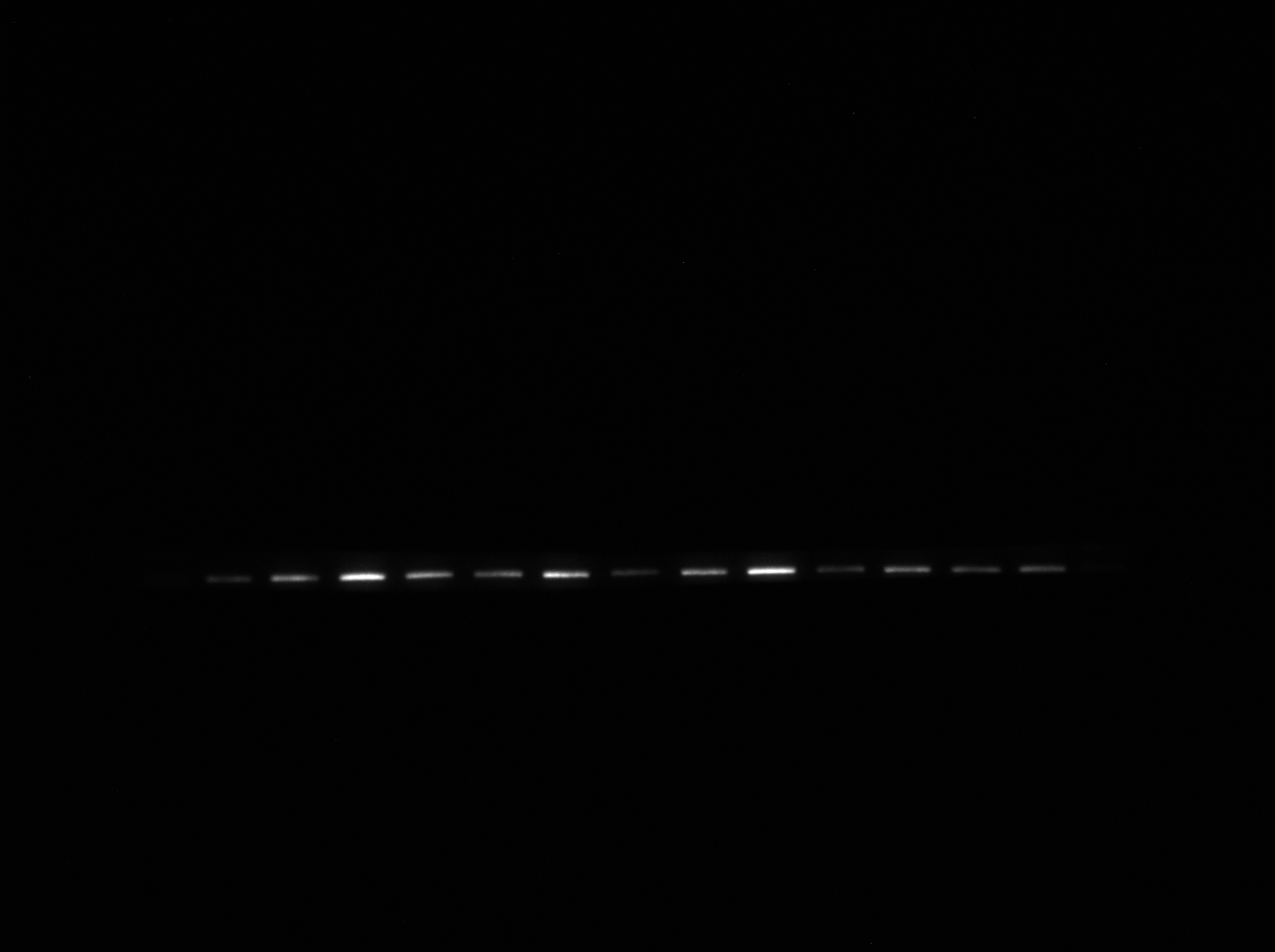

Supplement: Supplementary file 12 [file DataSheet5.ZIP › Figure2/Figure2D/SW620/shp2.tif]

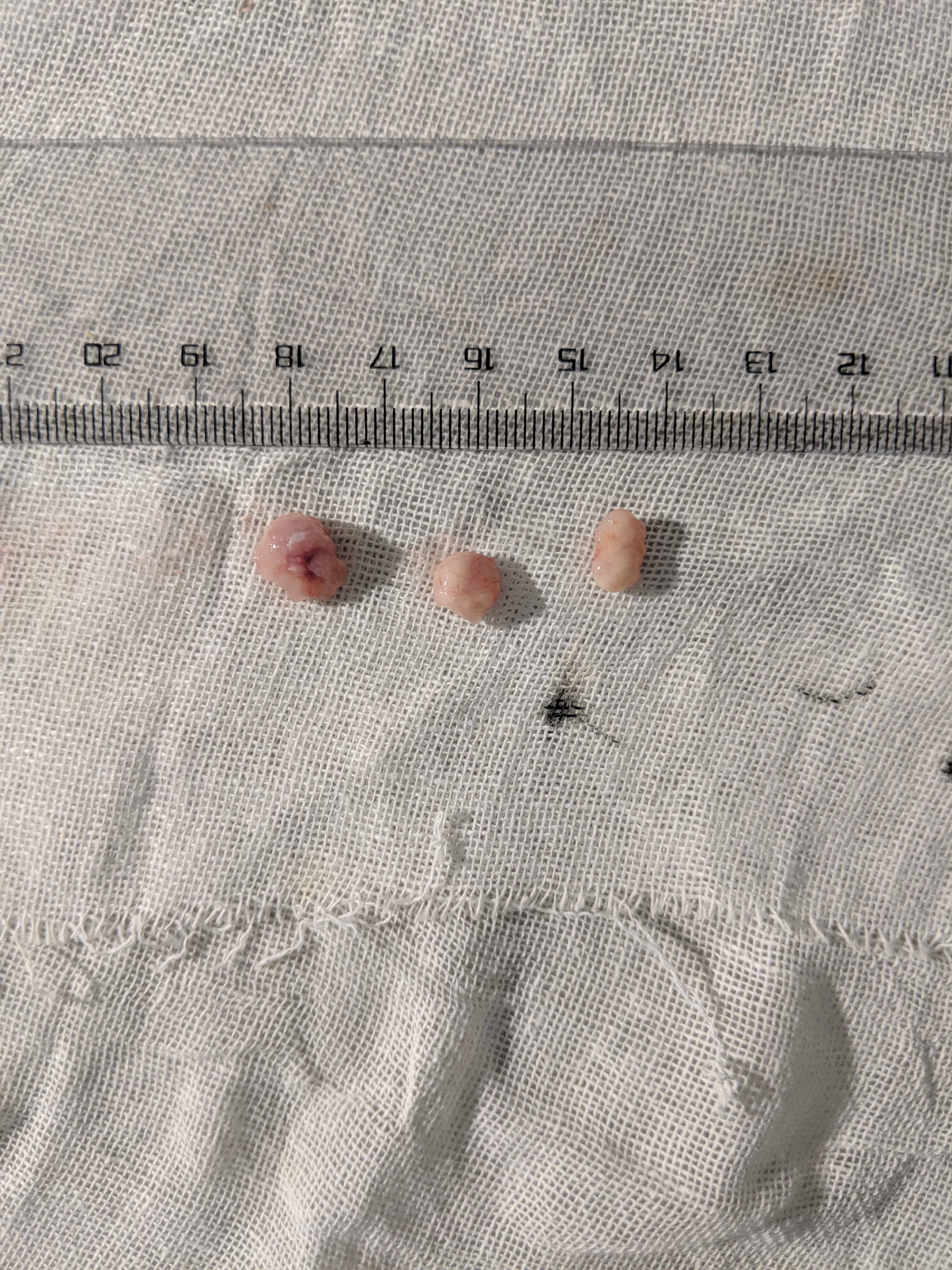

Supplement: Supplementary file 13 [file DataSheet7.ZIP › Figure4/B/Combination.jpg]

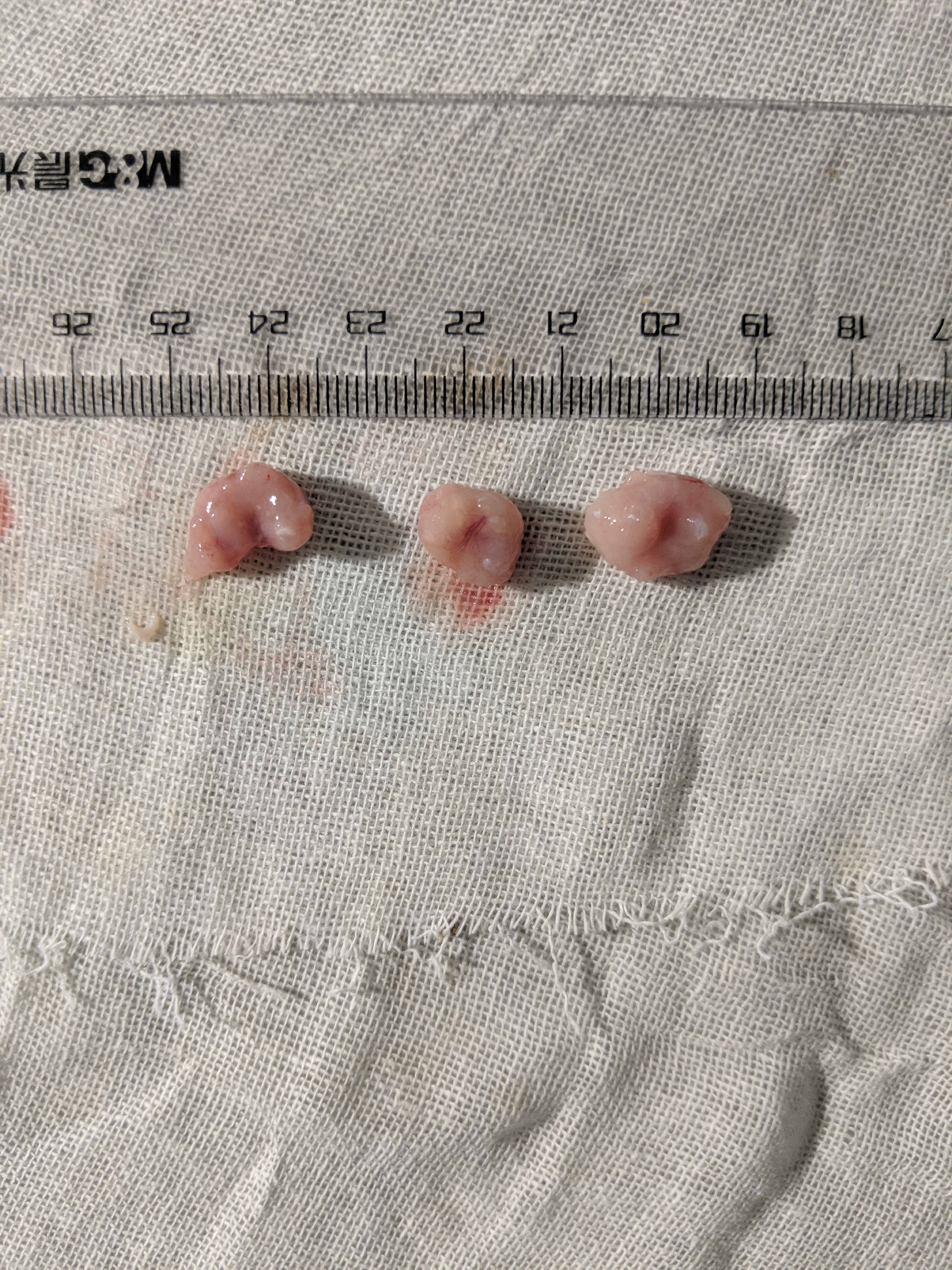

Supplement: Supplementary file 13 [file DataSheet7.ZIP › Figure4/B/MK2206.jpg]

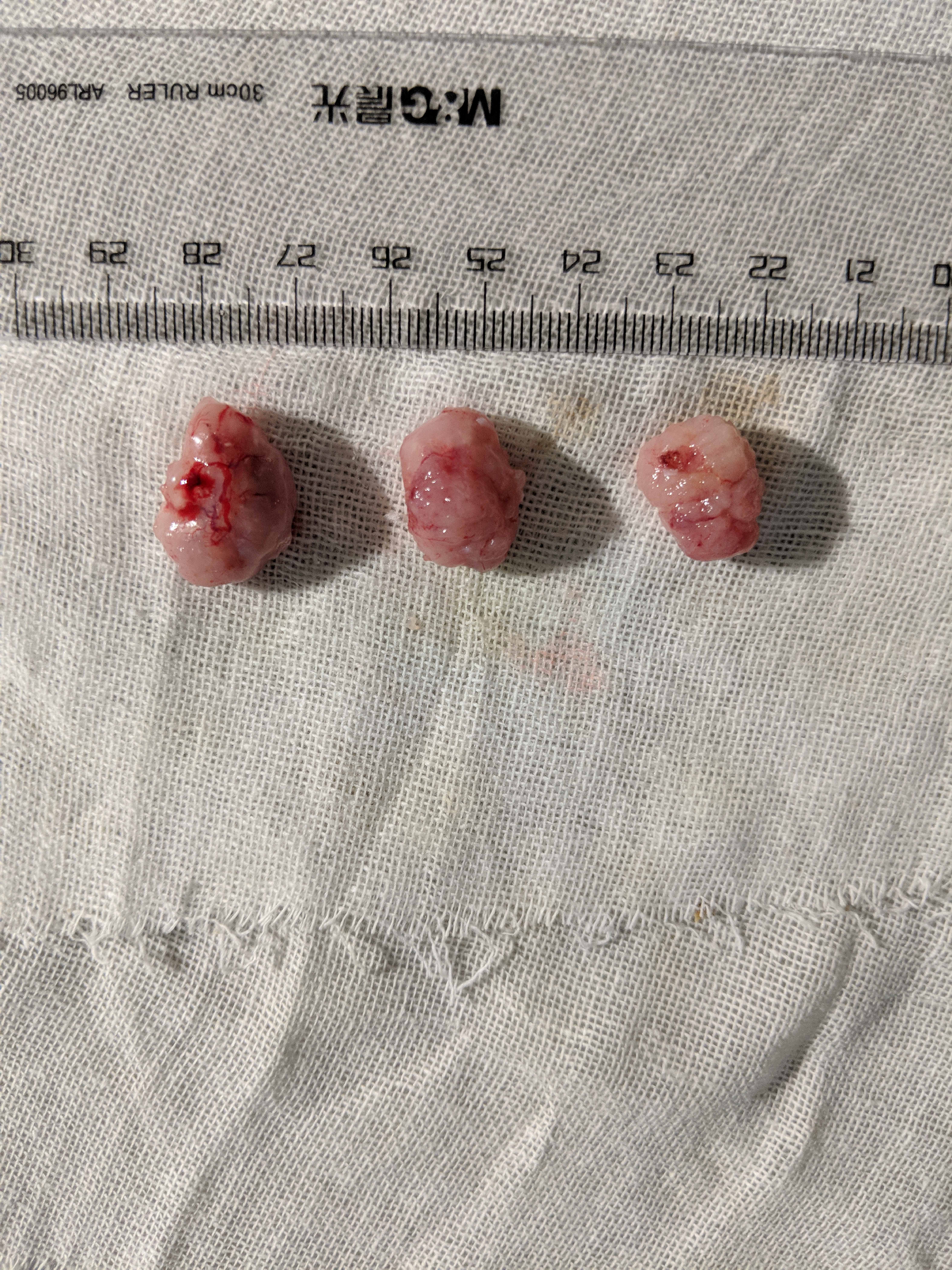

Supplement: Supplementary file 13 [file DataSheet7.ZIP › Figure4/B/NC.jpg]

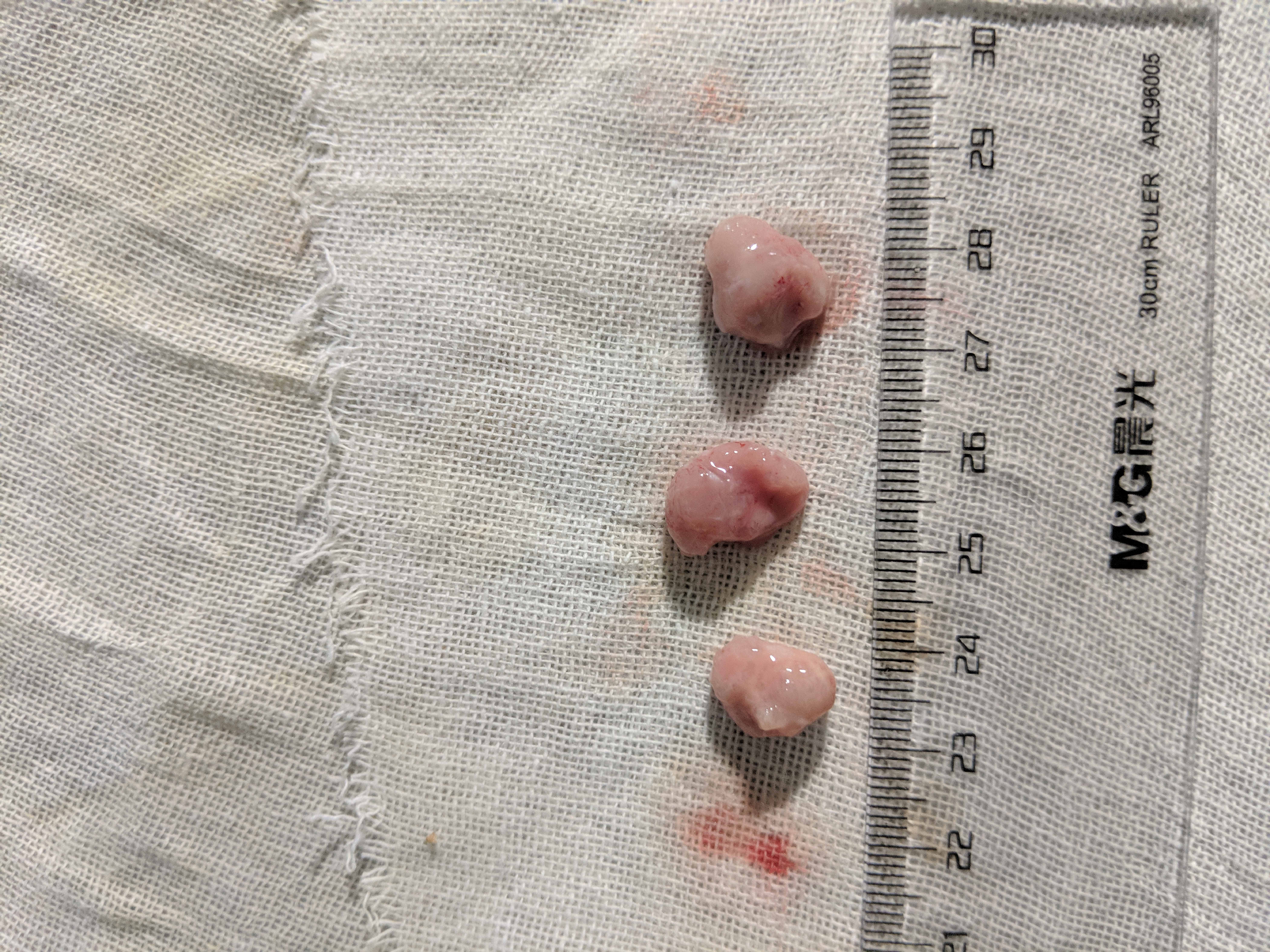

Supplement: Supplementary file 13 [file DataSheet7.ZIP › Figure4/B/SHP099.jpg]

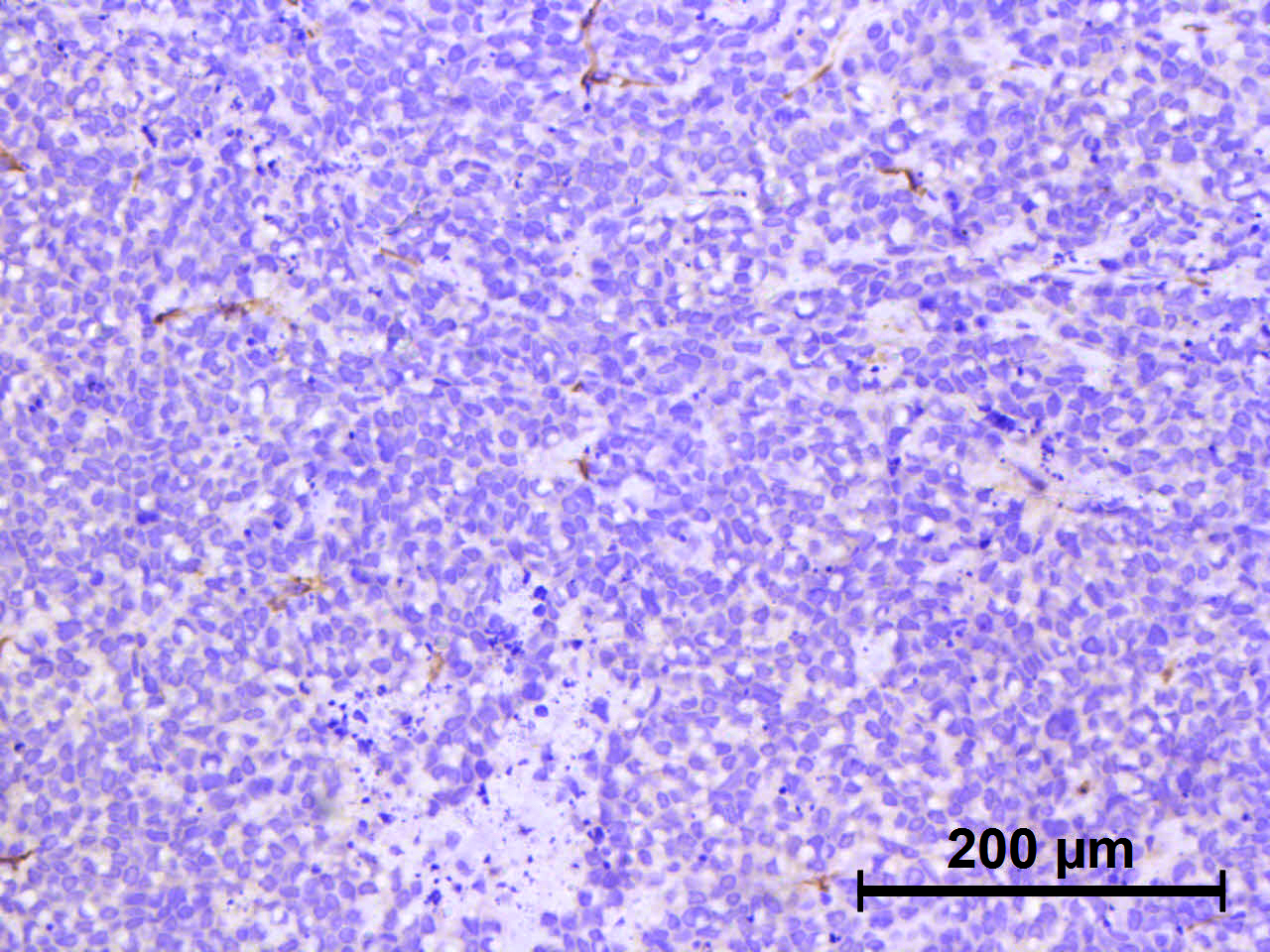

Supplement: Supplementary file 13 [file DataSheet7.ZIP › Figure4/D/CD31/Combination-C2-CD31-200X0320.jpg]

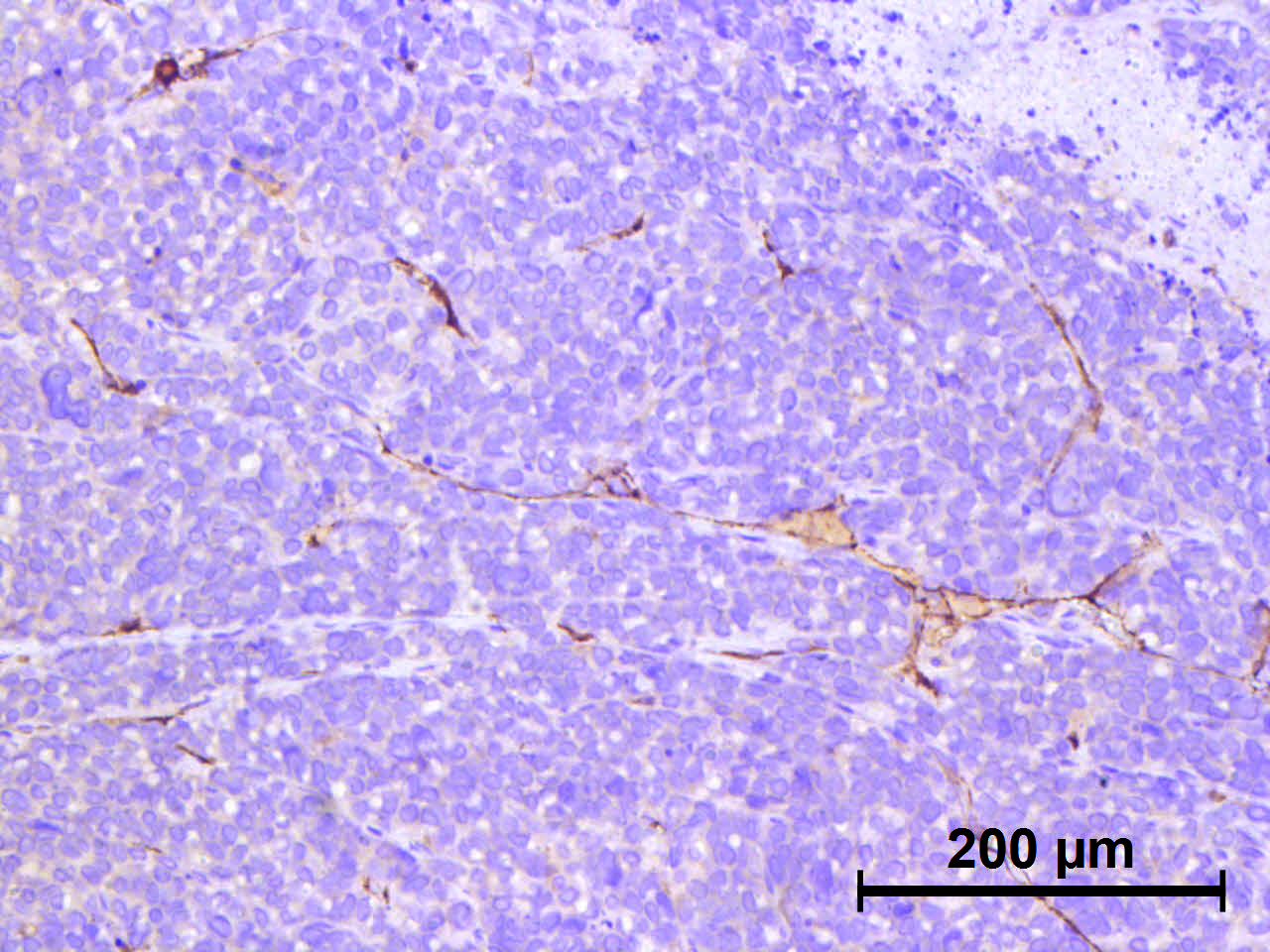

Supplement: Supplementary file 13 [file DataSheet7.ZIP › Figure4/D/CD31/Mk2206-B5-CD31-200X0284.jpg]

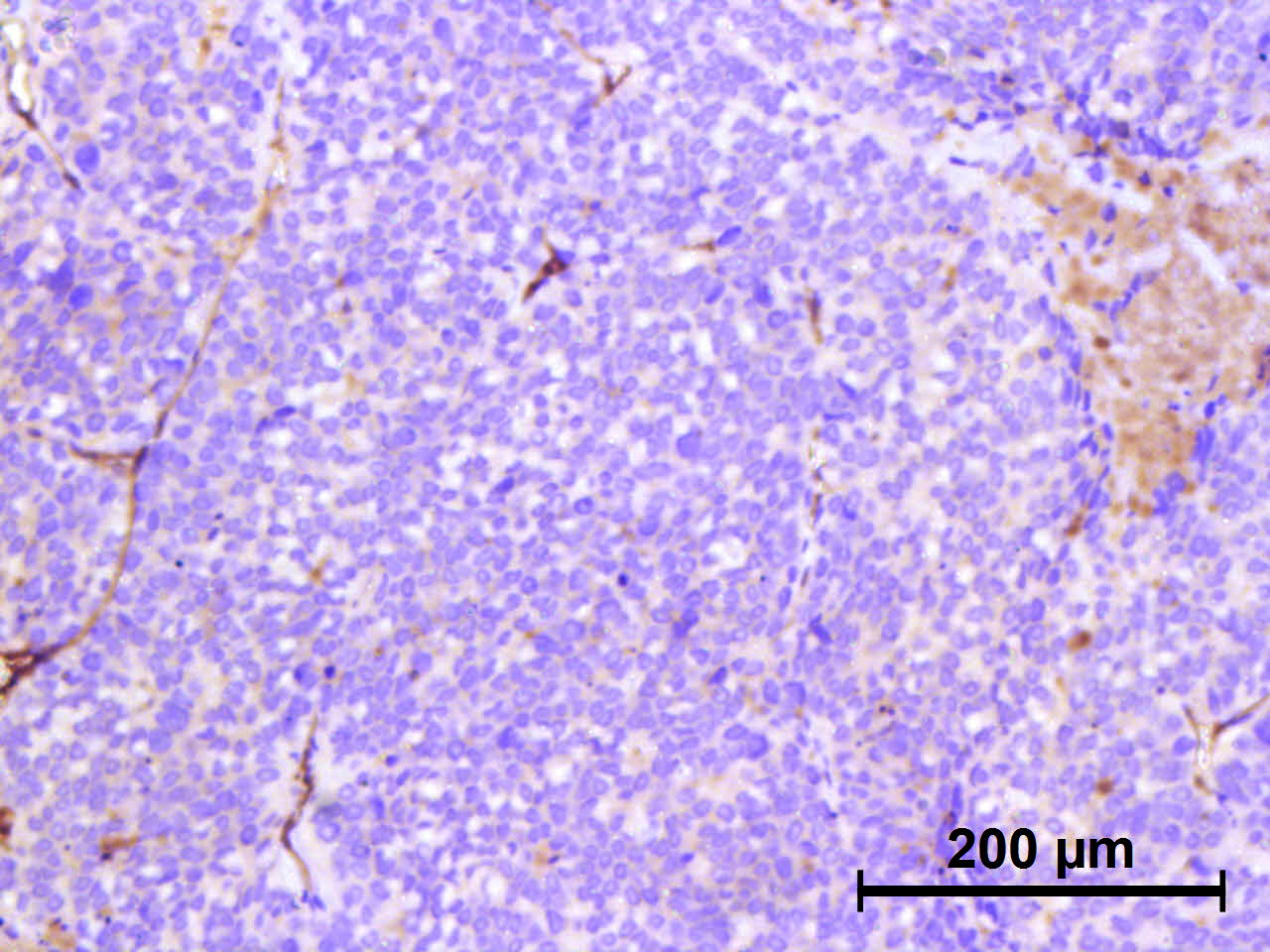

Supplement: Supplementary file 13 [file DataSheet7.ZIP › Figure4/D/CD31/SHP099-C6-CD31-200X0336.jpg]

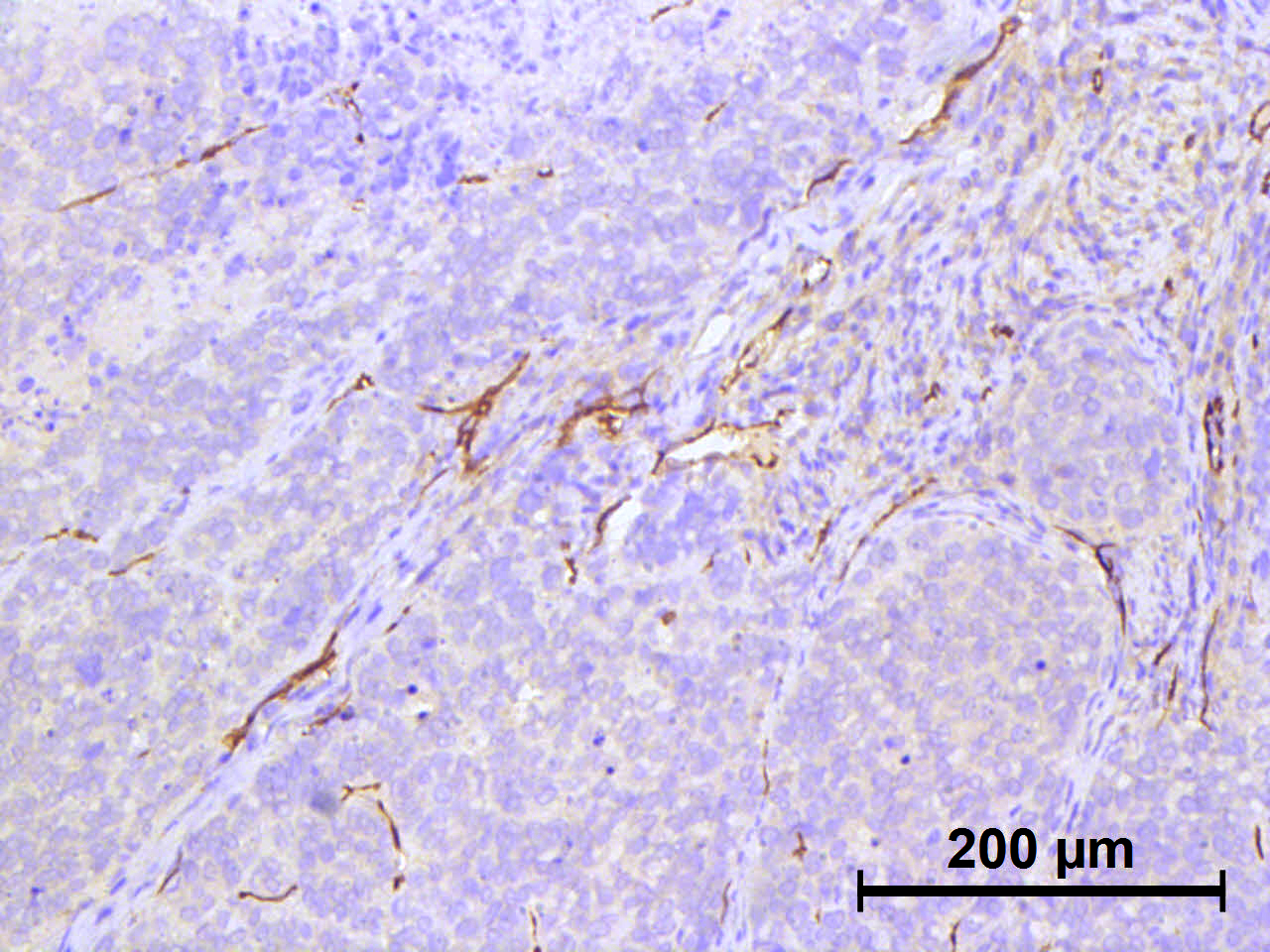

Supplement: Supplementary file 13 [file DataSheet7.ZIP › Figure4/D/CD31/Vehicle-B9-CD31-200X0311.jpg]

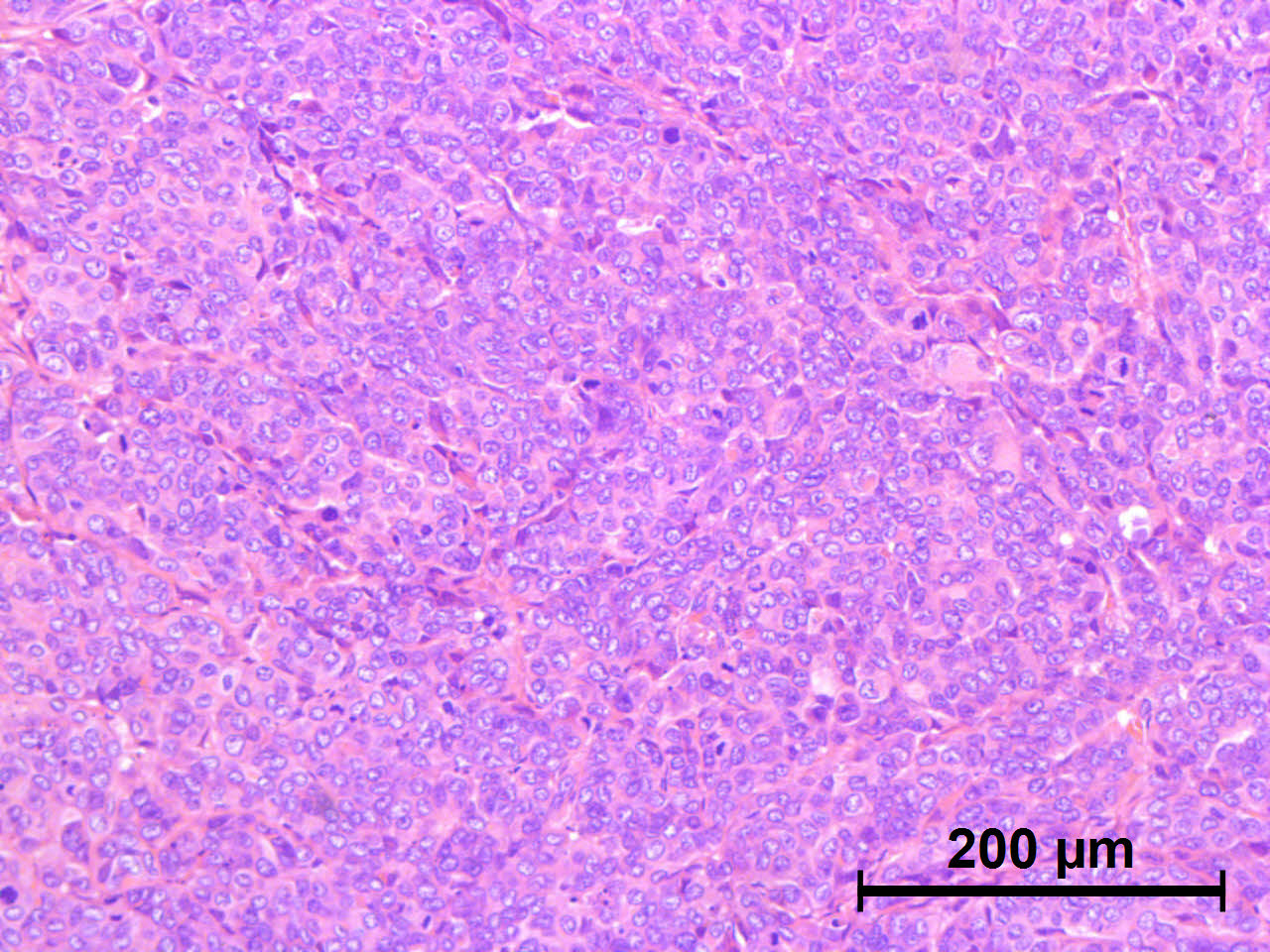

Supplement: Supplementary file 13 [file DataSheet7.ZIP › Figure4/D/HE/205-combination-C3-HE-200X.jpg]

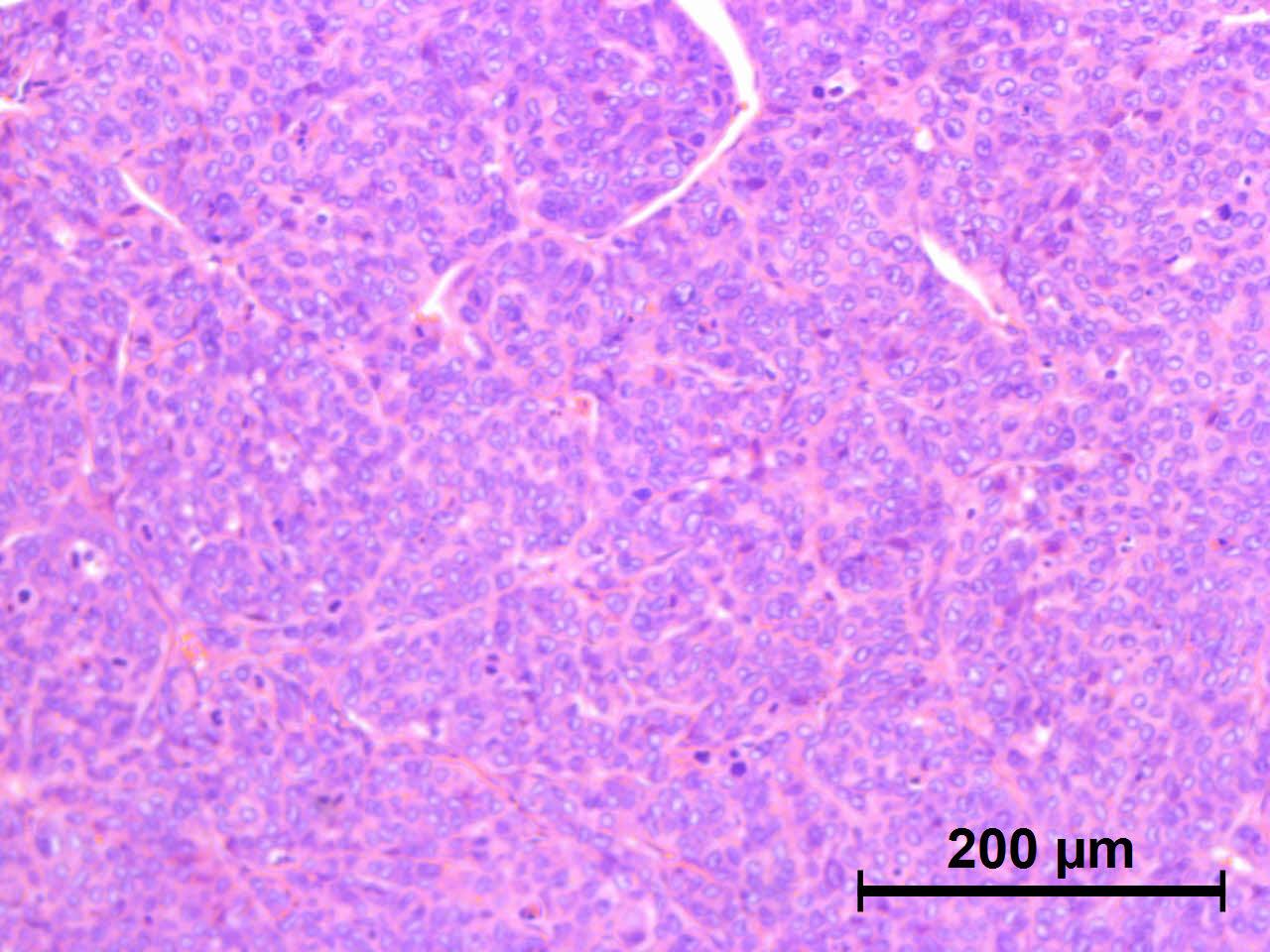

Supplement: Supplementary file 13 [file DataSheet7.ZIP › Figure4/D/HE/205-mk2206-B5-HE-200X.jpg]

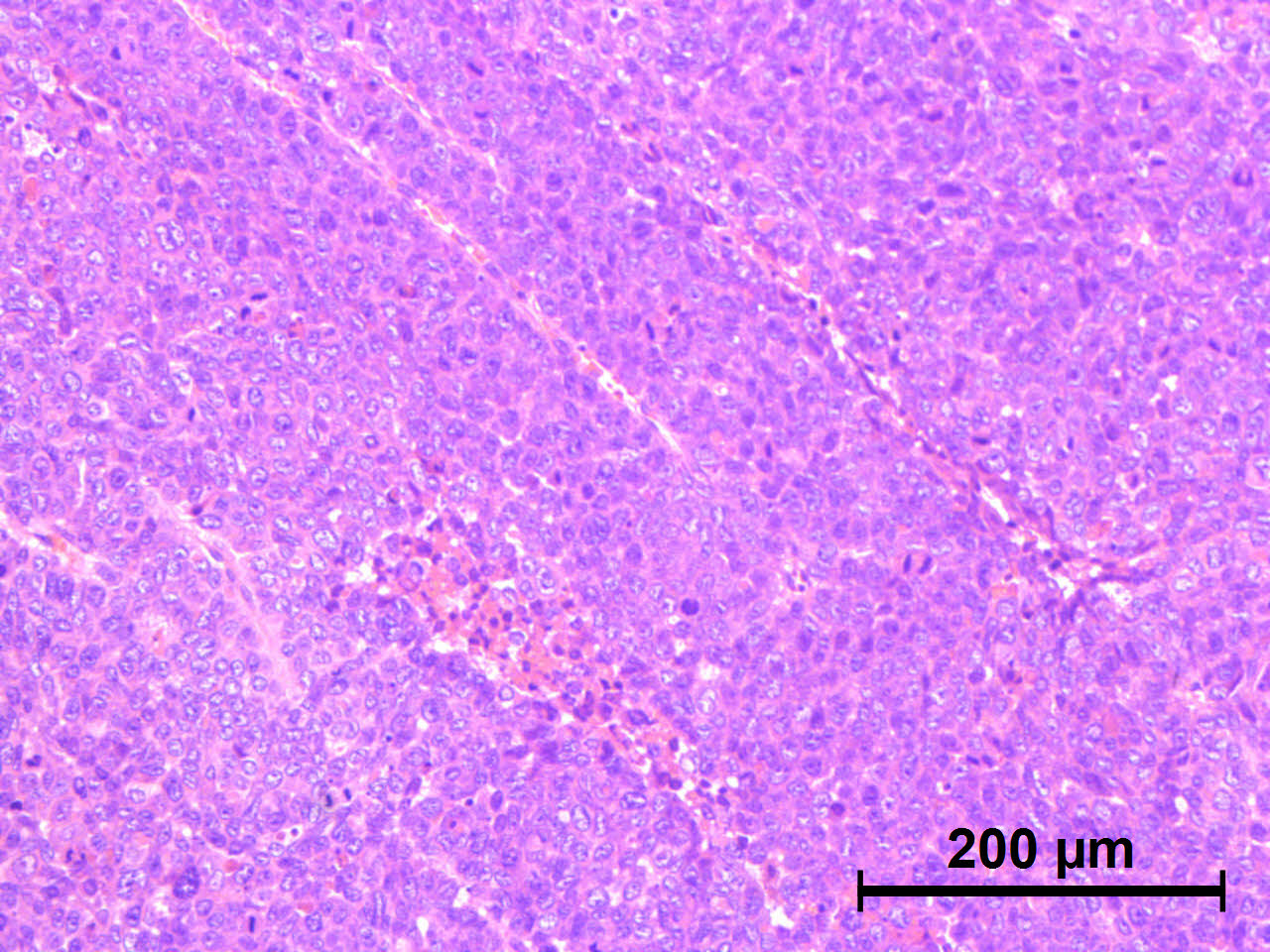

Supplement: Supplementary file 13 [file DataSheet7.ZIP › Figure4/D/HE/205-shp099-C6-HE-200X.jpg]

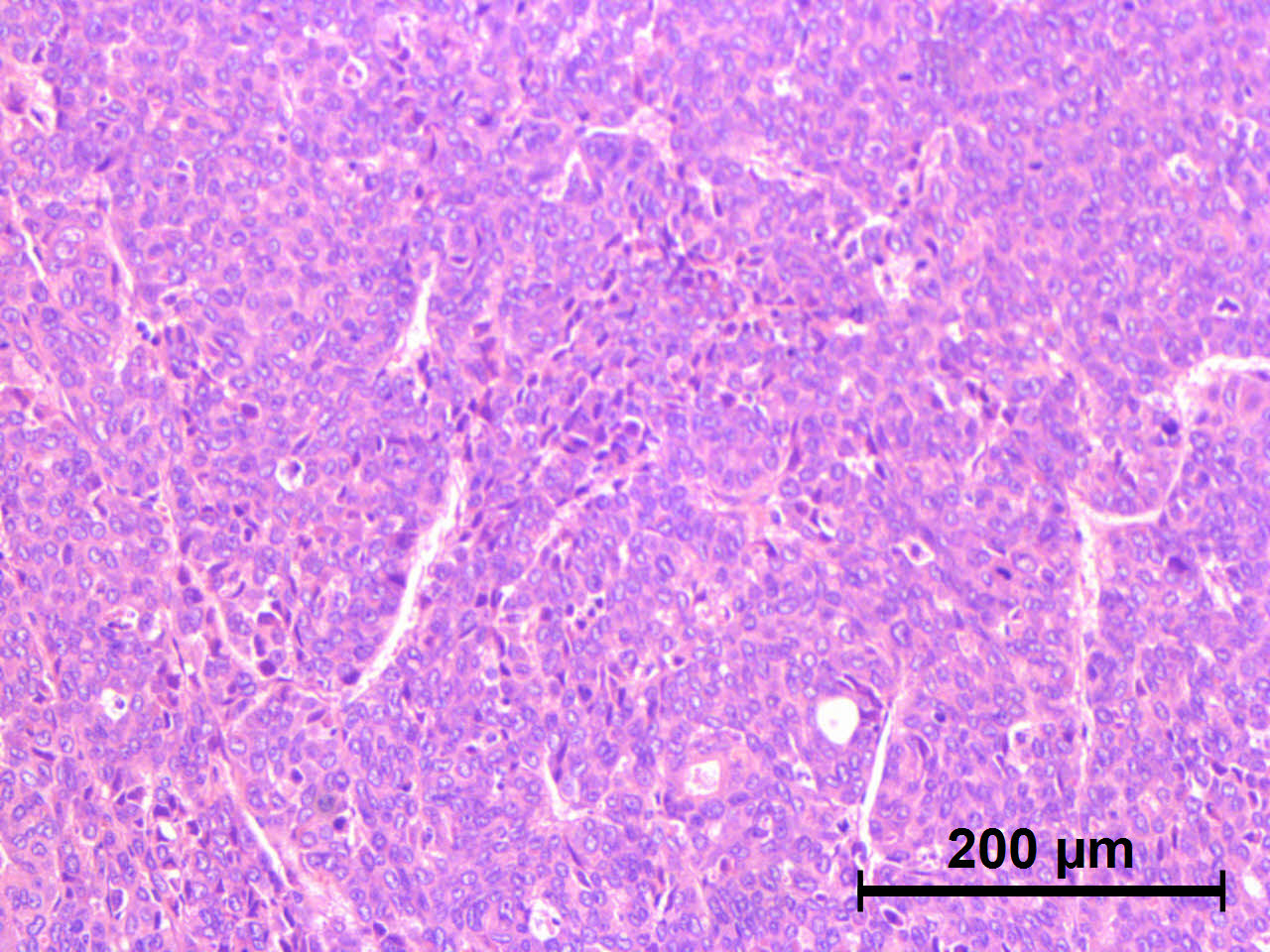

Supplement: Supplementary file 13 [file DataSheet7.ZIP › Figure4/D/HE/205-vehicle-C1-HE-200X.jpg]

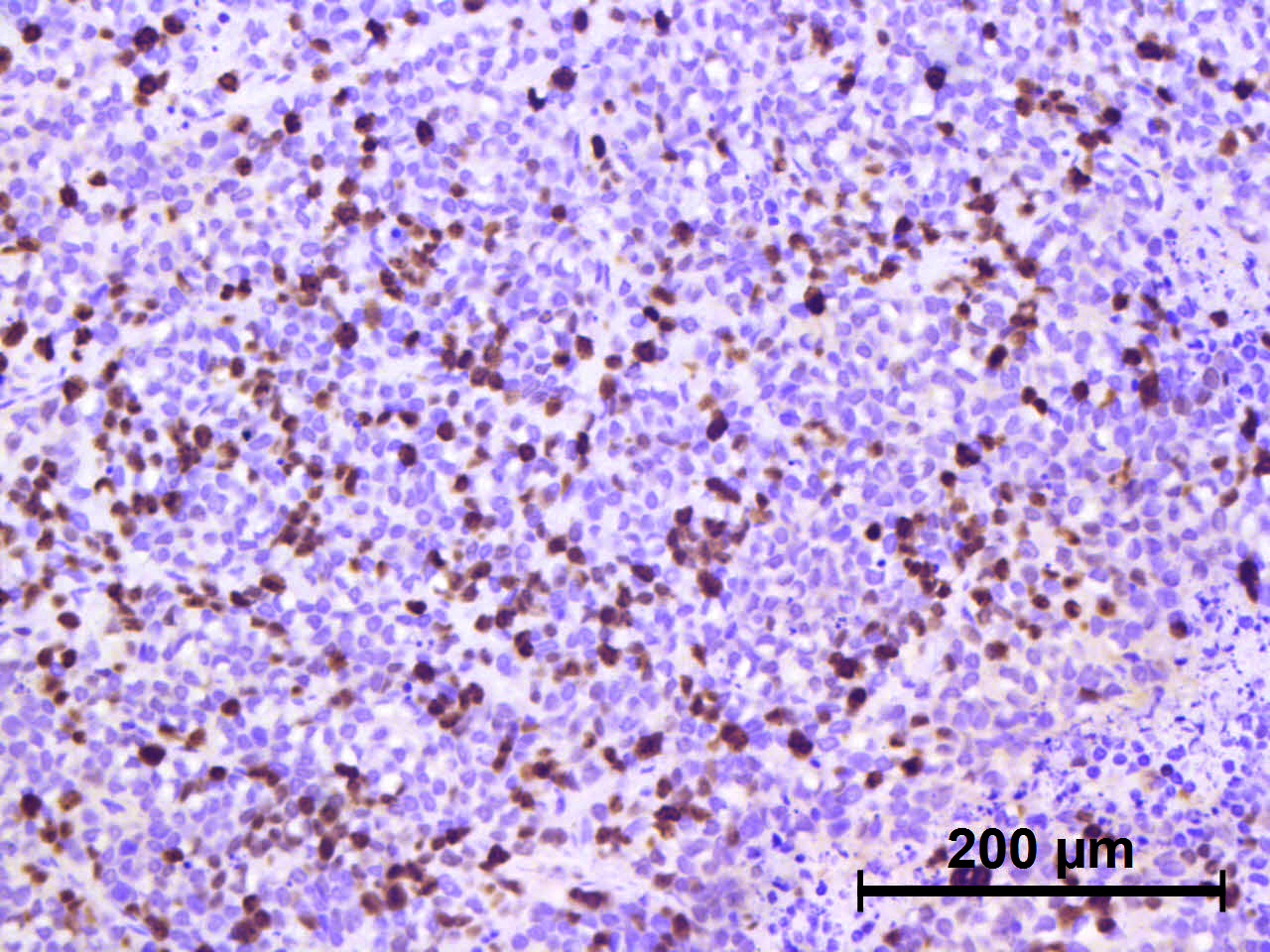

Supplement: Supplementary file 13 [file DataSheet7.ZIP › Figure4/D/KI67/205-combination-C1-KI67-3-200X0510.jpg]

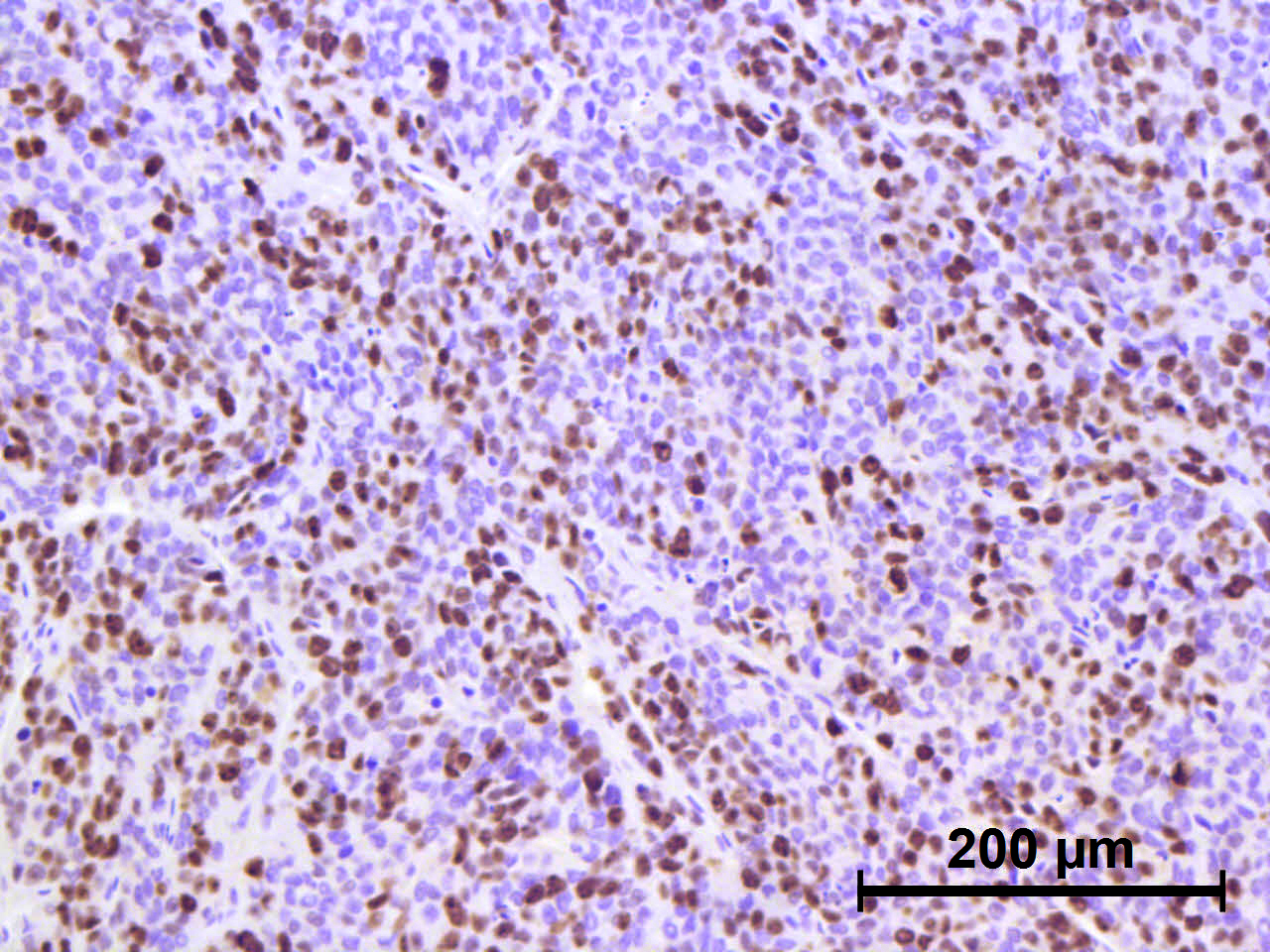

Supplement: Supplementary file 13 [file DataSheet7.ZIP › Figure4/D/KI67/205-mk2206-B5-KI67-3-200X0503.jpg]

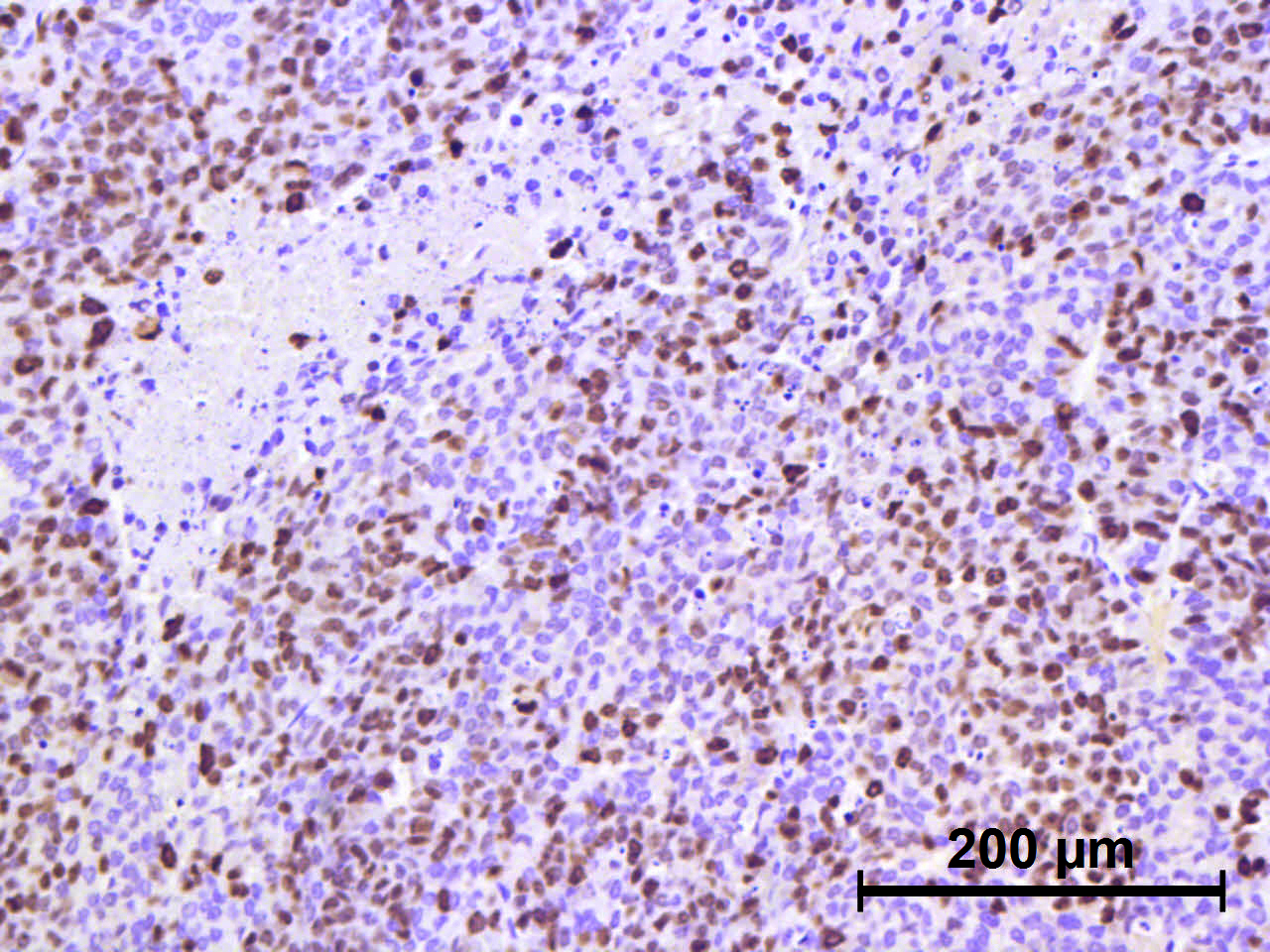

Supplement: Supplementary file 13 [file DataSheet7.ZIP › Figure4/D/KI67/205-shp099-C6-KI67-3-200X0515.jpg]

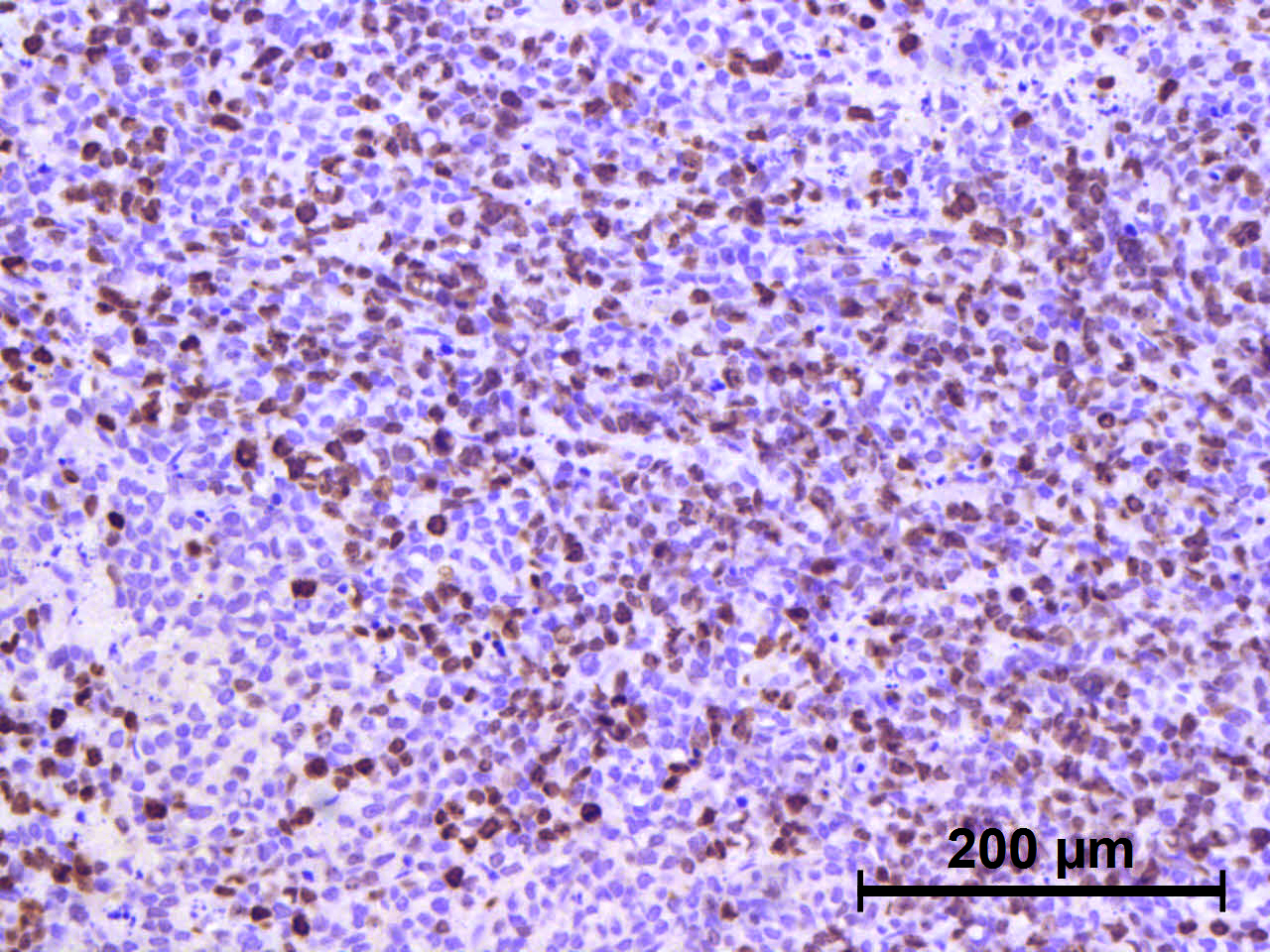

Supplement: Supplementary file 13 [file DataSheet7.ZIP › Figure4/D/KI67/205-vehicle-B8-KI67-3-200X0507.jpg]
